# Validation of proposed prostate cancer biomarkers with gene expression data: a long road to travel

**DOI:** 10.1007/s10555-013-9470-4

**Published:** 2014-01-30

**Authors:** Adriana Amaro, Alessia Isabella Esposito, Anna Gallina, Matthias Nees, Giovanna Angelini, Adriana Albini, Ulrich Pfeffer

**Affiliations:** 1 Functional Genomics, IRCCS A.O.U. San Martino – IST Istituto Nazionale per la Ricerca sul Cancro, Genoa, Italy; 2 Medical Biotechnology, VTT Technical Research Centre of Finland and University of Turku, Turku, Finland; 3 Research Infrastructure, IRCCS Arcispedale Santa Maria Nuova, Viale Umberto I°, 50 42100 Reggio Emilia, Italy

**Keywords:** Prostate cancer, Biomarkers, Multivariate model, PSA, Prognostic signature

## Abstract

Biomarkers are important for early detection of cancer, prognosis, response prediction, and detection of residual or relapsing disease. Special attention has been given to diagnostic markers for prostate cancer since it is thought that early detection and surgery might reduce prostate cancer-specific mortality. The use of prostate-specific antigen, PSA (KLK3), has been debated on the base of cohort studies that show that its use in preventive screenings only marginally influences mortality from prostate cancer. Many groups have identified alternative or additional markers, among which PCA3, in order to detect early prostate cancer through screening, to distinguish potentially lethal from indolent prostate cancers, and to guide the treatment decision. The large number of markers proposed has led us to the present study in which we analyze these indicators for their diagnostic and prognostic potential using publicly available genomic data. We identified 380 markers from literature analysis on 20,000 articles on prostate cancer markers. The most interesting ones appeared to be claudin 3 (CLDN3) and alpha-methysacyl-CoA racemase highly expressed in prostate cancer and filamin C (FLNC) and keratin 5 with highest expression in normal prostate tissue. None of the markers proposed can compete with PSA for tissue specificity. The indicators proposed generally show a great variability of expression in normal and tumor tissue or are expressed at similar levels in other tissues. Those proposed as prognostic markers distinguish cases with marginally different risk of progression and appear to have a clinically limited use. We used data sets sampling 152 prostate tissues, data sets with 281 prostate cancers analyzed by microarray analysis and a study of integrated genomics on 218 cases to develop a multigene score. A multivariate model that combines several indicators increases the discrimination power but does not add impressively to the information obtained from Gleason scoring. This analysis of 10 years of marker research suggests that diagnostic and prognostic testing is more difficult in prostate cancer than in other neoplasms and that we must continue to search for better candidates.

**Electronic supplementary material:**

The online version of this article (doi:10.1007/s10555-013-9470-4) contains supplementary material, which is available to authorized users.

## Introduction

Prostate cancer is the most commonly diagnosed non-skin cancer and the second leading cause of cancer death for males in the USA. More than 240,000 men were diagnosed with the disease and more than 33,000 died of it in 2011 [1]. If the current prostate-specific antigen-based screening schemes will be applied in the future, it can be estimated that 16.2 % of American men alive today will be diagnosed with the neoplasm and approximately 3 % will die of it. There is a general epidemiological trend towards growing incidence while mortality is stable. The increasing incidence is particularly evident for the period between 1980 and 1995 in affluent countries and at present in emerging countries [2]. This trend is probably at least in part due to the introduction of prostate cancer screening using prostate-specific antigen (PSA) as a marker. Introduction of PSA [3] has led to a drastic increase in the early detection of prostate cancer resulting in an increased reported incidence, in part due to indolent cancers.

PSA, a marker for prostate cells [4], is not specific for prostate cancer. Currently, PSA is used both as a diagnostic marker for early detection of prostate cancers and for follow-up after surgery or during prostate cancer therapy. PSA is expressed almost exclusively by the prostate; therefore, its expression is tightly linked to the presence of prostatic cells. After the initial clearance of residual PSA from the serum of patients who had their prostate surgically removed, increasing PSA levels indicate the presence of disseminated and eventually growing cells. During chemical castration, growing PSA levels can indicate failure of the therapy. While it is recognized an undisputable value as a follow-up marker, there is a longstanding discussion on its use as a diagnostic marker. The U.S. Preventive Services Task Force has reviewed the existing evidence for the benefit of PSA screening and has issued a recommendation against PSA screening in men over 65 years old in 2008 [5] that has been extended to younger men as a draft in 2011 [6] confirmed in 2012 [7]. The recommendation is based on two clinical trials that come to opposite conclusions: the US Prostate, Lung, Colorectal, and Ovarian cancer screening trial [8] showed an increased absolute risk of prostate cancer-specific mortality of 0.2/1,000 men associated with screening, whereas in the European Randomized Study of Screening for Prostate Cancer (ERSPC) [9], screening was associated with a decreased absolute risk of prostate cancer mortality of 0.6/1,000 men. The two associations were not statistically significant but, applying the statistical analyses planned a priori, a significant reduction of mortality (0.7/1,000 men; 1,410 screenings to prevent 1 death) was detected for the subgroup of men aged 55 to 65 years only in ERSPC.

PSA velocity, the increase over time of PSA serum levels, has been proposed as a more specific marker for cancer [10] but there is contrasting evidence [11]. The increase of PSA levels in the year before surgery identifies more aggressively growing cancers [12]. Most of PSA is bound to serum proteins and a minor part is free in the serum. The use of free PSA or the ratio between free and serum protein-bound PSA as markers may reduce unnecessary biopsies for men with relatively low PSA levels between 4 and 10 ng/ml [13].

A more recent addition to the urologist's toolbox is the prostate cancer antigen 3, PCA3, identified by Bussemaker and colleagues in 1999 [14] under the name DD3 using digital display screening for prostate cancer-specific RNAs (for a recent review see [15]). PCA3 is a non-coding RNA of unknown function. It is analyzed by RNA amplification methods from urine sediments after prostate massage [16]. In contrast to PSA, PCA3 is specifically expressed by prostate cancer cells [17]. The increased specificity is contrasted by a reduced sensitivity and PCA3 is therefore applied in association with PSA where it can reduce the number of unnecessary biopsies after a negative biopsy in men with elevated PSA levels [18, 19]. PCA3 may also have some prognostic potential inasmuch as its expression correlates with the Gleason score [20, 21], yet it has not been reported whether the combination of PCA3 and Gleason can improve prognostic power.

While the discussion on the appropriateness of PSA (and PCA3) screening is still open, it is widely held that improved biomarkers, especially biomarkers that distinguish normal prostate tissue from prostate cancer and markers associated with aggressive disease, could greatly improve prostate cancer screening results and deliver the benefit of early diagnosis and appropriate treatment to many men.

Cancer biomarkers are invaluable for early detection of cancer. An ideal marker would be expressed by tumor cells but not by the normal counterpart or other tissues. Diagnostic markers are applied to screening of healthy people, in particular of those with an elevated specific risk and must therefore be measurable in the least invasive manner possible. Although screening programs invariably lead to some degree of overdiagnosis, early detection has led to a reduction in cancer mortality for breast cancer (mammography, echography) [22], cervical cancer (Papanicolaou test, HPV screening) [23], and colon cancer (occult fecal blood, colonoscopy) [24].

Prognostic biomarkers can help to distinguish relatively benign cancers from aggressive ones and might orient treatment decisions. These markers are useful post-detection where they should be able to distinguish aggressive disease so as to direct the surgical/therapeutic intervention that might be unnecessary for non-aggressive cancers. Prognostic histopathological analyses are standard for many cancers, and prognostic gene expression signatures are being applied to the treatment decision for breast cancer [25–27].

Predictive markers can detect drug sensitivity or resistance guiding the treatment choice. These markers are useful after diagnosis and most often are detected in tissue samples obtained by biopsy or surgery. Examples of predictive markers are epidermal growth factor receptor (EGFR) and k-RAS mutations in non-small cell lung cancer that guide the use of EGFR-specific tyrosine kinase inhibitors [28] or HER2 overexpression or amplification that indicates the treatment with anti-HER2 antibodies [29] or adjuvant anthracyclines [30].

Follow-up markers allow for the screening of residual or relapsing disease and should be measured in a noninvasive way, by the analysis of sera, plasma, or urine. Serum PSA, in particular free PSA, is widely used as a follow-up marker after surgery and during therapy given its specificity for prostate tissue [31]. In addition, specific radiation response markers for prostate cancer have been proposed [32, 33]. New markers are therefore mostly needed for screening, for early diagnosis, and for prognosis.

PSA is almost exclusively produced by the prostate and released into the serum. Yet, its use as a diagnostic marker is limited by the fact that it is also expressed in healthy prostate tissue and that circulating levels can be elevated in subjects with prostatitis, inflammation, benign prostatic hyperplasia [34], and after recent ejaculation [35]. In addition, the PSA screening trials show that many diagnosed prostate cancers do not develop into a life-threatening disease. Further, prostate cancer can develop in individuals whose PSA levels remain low.

Biomarker research today can rely on a large number of publicly available data that allow for in-depth analyses of the association between gene expression and clinical and histopathological variables. Our aim in this study is to review the state of the art and eventually to restrict the number of candidate markers to those with molecular characteristics and expression profiles compatible with a selective marker function. We have analyzed the literature over the last 10 years identifying a large number of markers that have been proposed as diagnostic or prognostic markers for prostate cancer. We analyzed these markers using several datasets for their ability to discriminate healthy and neoplastic prostate tissue and for their capability to predict the clinical behavior of the tumors. Finally, we tried to develop prognostic signatures on the base of the published markers. Our results show that the proposed markers either taken alone or combined in a signature have a limited diagnostic or prognostic power and that further studies need to be done across an increasing range of potential marker sources.

## Methods

### Identification of prostate cancer markers

PubMed was screened for scientific articles published from 2001 to 2011 with the terms “prostate” AND “marker*” or “prostate” AND “biomarker*” in any field. The articles identified were manually analyzed for genes encoding the prostate cancer biomarkers reported. For markers that were reported in more than one paper, the paper publishing the marker for the first time was used as a reference. No further filtering was applied. All markers were considered without regard to the nature of the originally proposed markers (protein or mRNA) or the analysis method used. The official gene symbol and the Ensembl accession number of the genes encoding the markers were identified using the gene ID conversion tool of DAVID Bioinformatics Resources 6.7 (http://david.abcc.ncifcrf.gov/) [36], and the resulting list was manually managed in order to obtain the gene IDs for all markers. All markers considered are listed in Supplementary Table 1.

### Datasets used

General gene expression data have been obtained from the GeneSapiens database. Briefly, GeneSapiens (http://www.genesapiens.org/) [37] is a collection of 9,873 Affymetrix microarray gene expression profiling experiments. All samples are reannotated and normalized with a custom algorithm. The data are collected from various publicly available sources, including Gene Expression Omnibus and ArrayExpress and cover 175 different tissue types. Mean expression of each gene was determined in prostate cancer (*n* = 349) and healthy prostate (*n* = 147).

For the evaluation of the prognostic potential of markers, we used gene expression data of prostate cancers of the Swedish Watchful Waiting cohort with up to 30 years of clinical follow-up data set sampling 281 prostate cancers analyzed by microarray analysis of formaldehyde-fixed formalin-embedded specimens (GSE16560) [38]. We used the GSE21034 dataset [39] for external validation. This dataset derives from a study of integrated genomics of 218 prostate cancers. The gene expression analysis was performed using Affymetrix Exon 1.0 microarrays.

For additional analyses of marker expression in normal and tumoral tissue, we used the GSE6919 dataset containing 152 human samples including prostate cancer tissues, prostate tissues adjacent to tumor, and organ donor prostate tissues, obtained from men of various ages [40, 41].

### Statistical analyses

All biomarkers extracted from the literature for which a corresponding probe set was present on the two array platforms used were used for all analyses irrespective of the scope for which they have been designed (diagnostic or prognostic markers). Thus, we should be able to detect eventual prognostic power of diagnostic markers and vice versa as well as the original application. For gene expression analyses in prostate cancer versus normal prostate tissue, Student's *t* test was used.

Correlations with survival were performed using the GSE16560 and GSE21034 datasets. All markers for which probe sets were present were analyzed using the complete dataset. The prognostic value of the signature was tested by Kaplan–Meier survival analysis and Cox regression analysis. As endpoints, we used survival (“indolent” = over 10 years survival after diagnosis and “lethal” = death within 10 years after diagnosis) for GSE16560 and distant metastasis for GSE21034 since the latter contained only few disease-specific deaths.

## Results

**Fig. 1:**
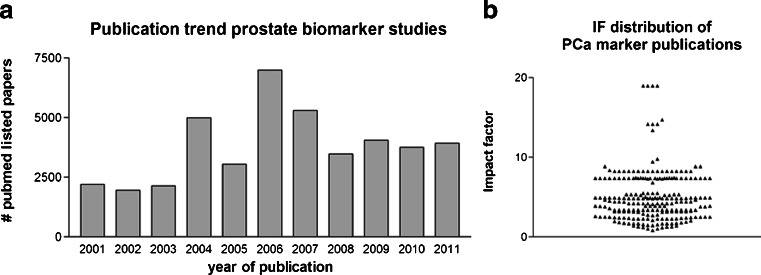
Publications on prostate cancer biomarkers 2001–2011. **a** Publications per year. **b** Distribution of publications according to impact factor

The analysis of the literature has led to the identification of over 20,000 articles on prostate markers published between January 1, 2001 and June 1, 2011. Articles published in journals not indexed in the Journal Citation Reports and articles reporting on markers that are not measured as mRNA or protein expression were omitted from further analyses. Two hundred forty-four articles report for the first time at least one new mRNA or protein marker for a total of 380 markers. There is a trend towards slightly increasing numbers of articles reporting prostate cancer markers over time (Fig. 1a). The studies have been published in journals with a wide range of impact factors from 0.822 (*Ca. J. Urol*) to 18.97 (*J. Clin. Oncol.*) (Fig. 1b). The complete bibliography containing the list of references for all studies included in this analysis is available as Supplementary Table 1.

**Fig. 2:**
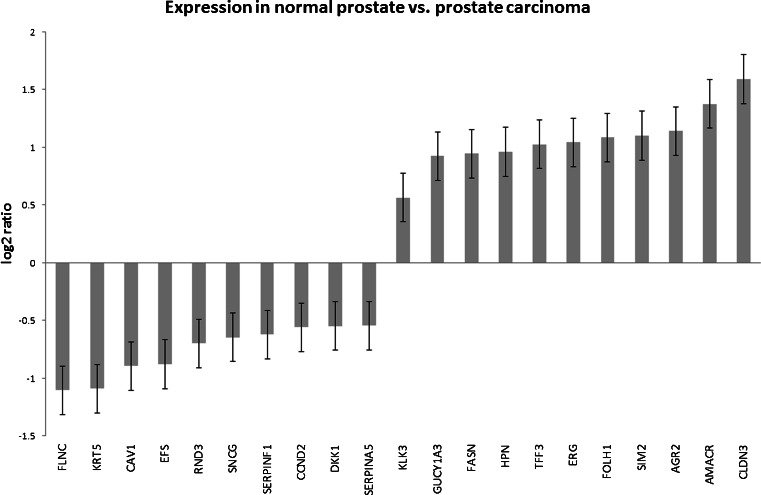
Expression of prostate cancer biomarkers in healthy and tumoral prostate tissues. The ratios of expression in healthy and tumoral prostate tissues of the 20 prostate cancer biomarkers that are most significantly differentially expressed are reported. KLK3 (PSA) has been added for comparison

We analyzed all markers together irrespective of the potential application (diagnostic, prognostic, or follow-up) of the marker claimed in the original publication. We evaluated the markers for their diagnostic potential using a set of microarray data of the GeneSapiens database containing 147 normal prostate and 329 prostate cancer tissues. Of the 287 markers identified in the microarray data, 143 markers had significantly different expression values when normal and cancer tissues were compared (*p* < 0.01). Figure 2 shows the ten markers with highest (downregulated in cancer) and ten with the lowest (overexpressed in cancer) expression ratio and, for comparison, kallikrein-related peptidase 3 (KLK3), the gene encoding PSA. The lowest score, 0.46, is attained by filamin C (FLNC) [42] and the highest score, 3.00, by claudin 3 (CLDN3) [43] (KLK3/PSA = 0.56). The data for all markers are reported in Supplementary Table 2.

**Fig. 3:**
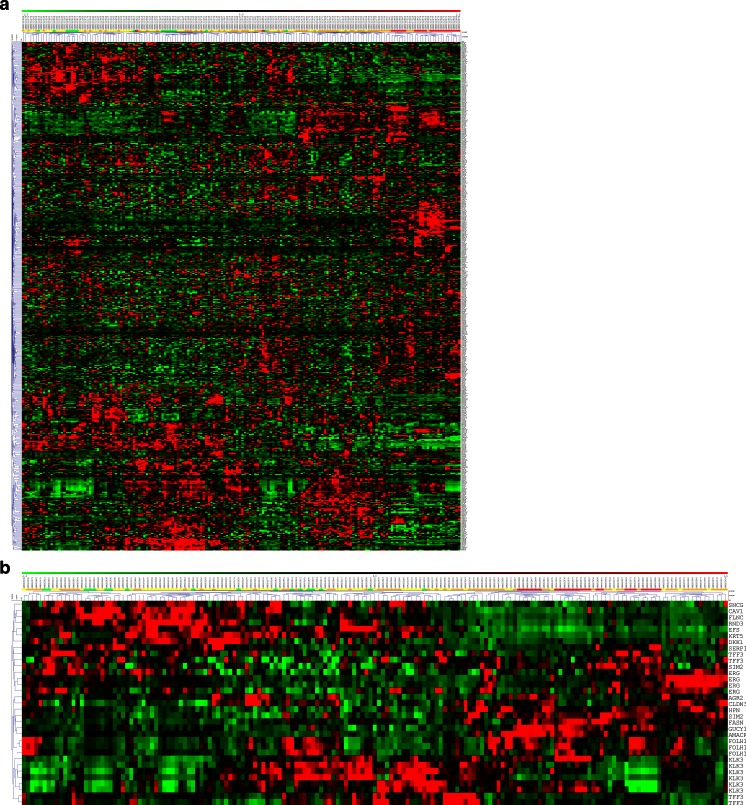
Hierarchical clustering marker gene expression in human prostate tissues from dataset GSE6919 using Euclidean distance measures and average linkage. The state of the tissue is indicated by a *color code* in the *bar above the dendrogram* (*green* = prostate tissues from healthy donors, *yellow* = peritumoral tissue, *orange* = tumor tissue, *red* = metastases). For markers represented by more than one probe set on the array, all probe sets were included in the analysis. **a** All prostate cancer biomarkers. **b** The 20 best markers from Fig. 2

We investigated the expression of the markers in an independent dataset (GSE6919) containing data from 152 human prostate tissues including normal prostate tissue from healthy donors, prostate cancers, peritumoral tissues, and prostate metastases. Hierarchical clustering of the expression data for the 380 markers reveals that almost all of the metastases and many of the tumor tissues cluster together in a cluster distinct from the clusters containing mainly peritumoral and normal tissues, indicating that the combination of markers distinguishes to some extent healthy and tumor tissues. However, the clusters formed are not strongly distinct as the distances in the dendrogram are short (Fig. 3a). When the same analysis is limited to the 20 markers from Fig. 2 whose expression is most different in normal versus tumor tissues, the clusters formed become slightly more robust and all the metastases and the majority of tumors are in one cluster, yet the clusters are still not very distinct (Fig. 3b).

**Fig. 4:**
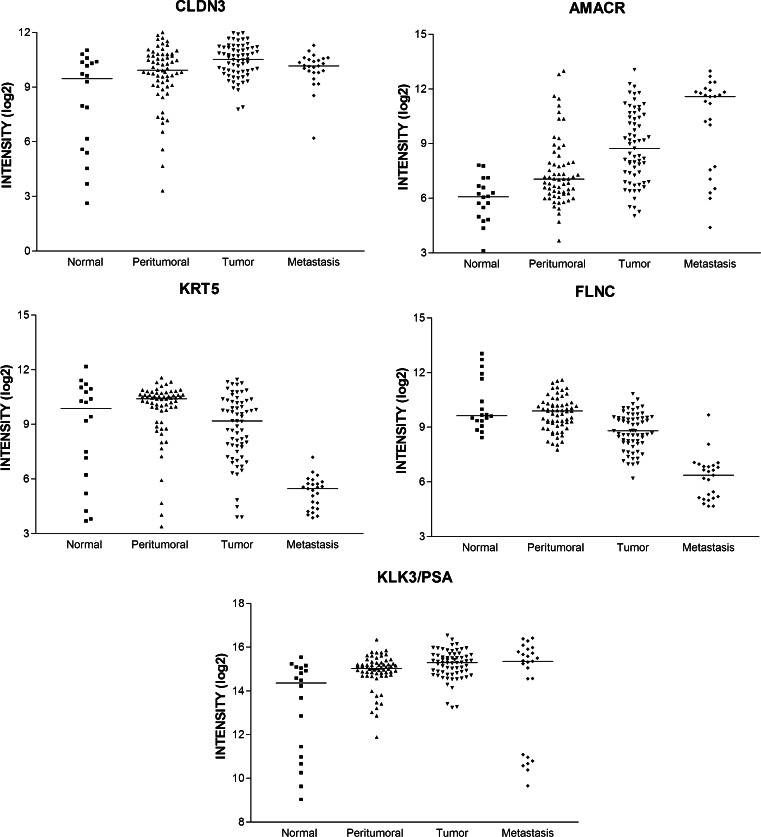
Expression scatter plots of the four best prostate cancer biomarkers (claudin 3 (CLDN3), alpha-methylacyl-CoA racemase (AMACR), keratin 5 (KRT5), filamin C (FLNC)) in comparison to KLK3 (PSA). Expression data for healthy, peritumoral, tumoral, and metastatic prostate tissues are shown for the four most differentially expressed markers in comparison to KLK3 (PSA)

Figure 4 reports the expression scatter plots of the two markers with the strongest overexpression in cancer (alpha-methylacyl-CoA racemase/AMACR [44] and CLDN3 [43]) and in normal (FLNC [42] and keratin 5/KRT5 [45]) tissue and KLK3/PSA for comparison. The new markers do not appear to be clearly superior to KLK3/PSA inasmuch as their expression is not drastically different in normal and cancerous tissues and their expression in normal and cancer tissues varies widely not allowing for the classification of single patients according to the expression levels, although the expression differences are statistically significant.

**Fig. 5:**
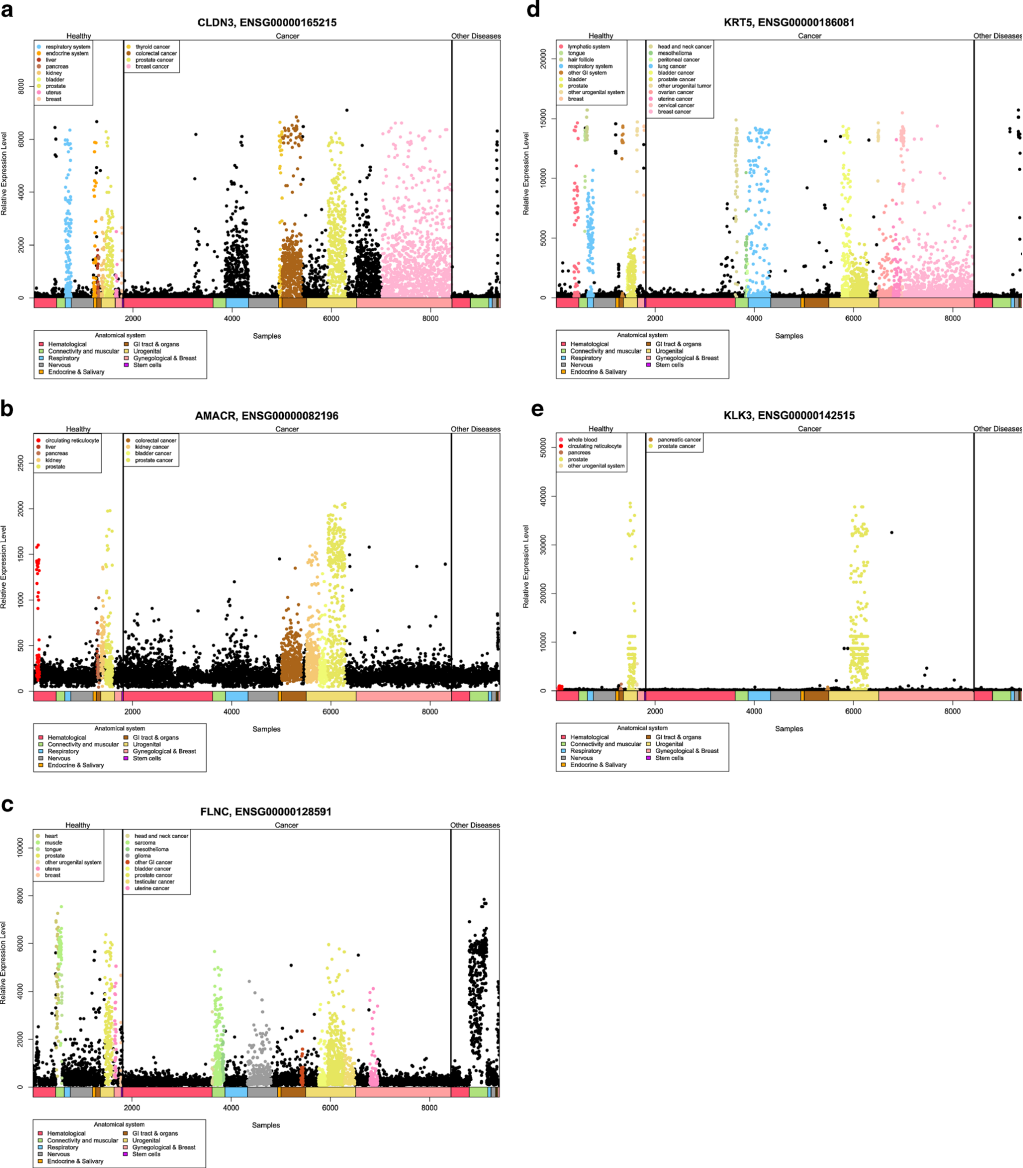
Relative expression of prostate cancer biomarkers in various tissues **a** claudin 3 (CLDN3), **b** alpha-methylacyl-CoA racemase (AMACR), **c** filamin C (FLNC), **d** keratin 5 (KRT5), **e** KLK3/PSA. Note that only KLK3/PSA is highly specific for prostate tissues

The different significance level of the “new” markers as compared to KLK3/PSA (*p* = 1.2 × 10^−16^ for FLNC, 1.67 × 10^−9^ for CLDN3, 1.89 × 10^−3^ for KLK3/PSA) could indicate that these markers are more powerful for the discrimination of cancer and normal tissue. The potential as a diagnostic marker depends, however, at least for serum markers, also on the prostate-specific expression as compared to other tissues. We therefore used the GeneSapiens set of microarray data to monitor tissue-specific expression of the new markers as compared to KLK3/PSA. Figure 5 reports the expression patterns for the four best new markers and KLK3/PSA. This analysis shows that despite the greater expression difference in normal versus cancer tissues, the new markers are unlikely to be superior to KLK3/PSA given their widespread expression in other normal and neoplastic tissues as well as in tissues affected by other diseases. The prostate specificity of KLK3/PSA is unmet.

**Fig. 6:**
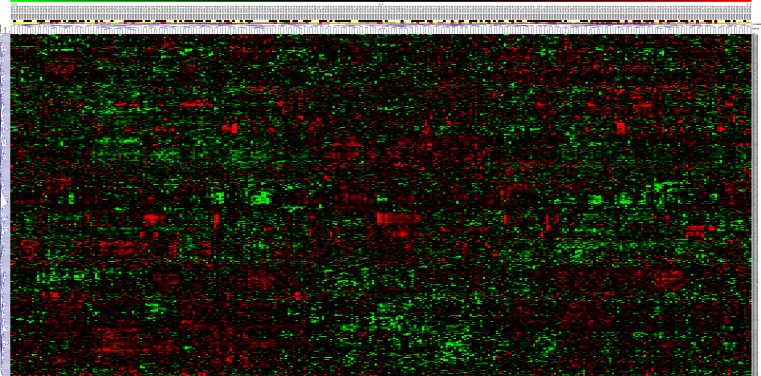
Hierarchical clustering of 281 prostate cancer tissues. Gene expression values of the genes encoding potential prostate cancer biomarkers in 281 prostate cancers from dataset GSE16560 were clustered using Euclidean distance measure and average linkage. Cancer status (indolent = *white*, lethal = *black*) and Gleason score (5 = *green*, 6 = *yellow*, 7 = *orange*, 8 = *pink*, 9 = *red*) are indicated in the *bars above the dendrogram*

We next asked whether the markers identified have any prognostic potential. We used the GSE16560 dataset of 281 prostate cancers. The samples are derived from FFPE material from transurethral resection of prostate at the time of the initial diagnosis. Patients who died of the disease within 10 years (*n* = 140) and patients who survived at least 10 years (*n* = 141) were selected for the analysis allowing for a clear-cut distinction. For 280 of the 380 proposed markers, a corresponding probeset could be identified on the arrays used for this study. Hierarchical clustering of the gene expression data of these markers did not show strong associations of gene expression values with status (lethal or indolent) or Gleason score (Fig. 6).

**Fig. 7:**
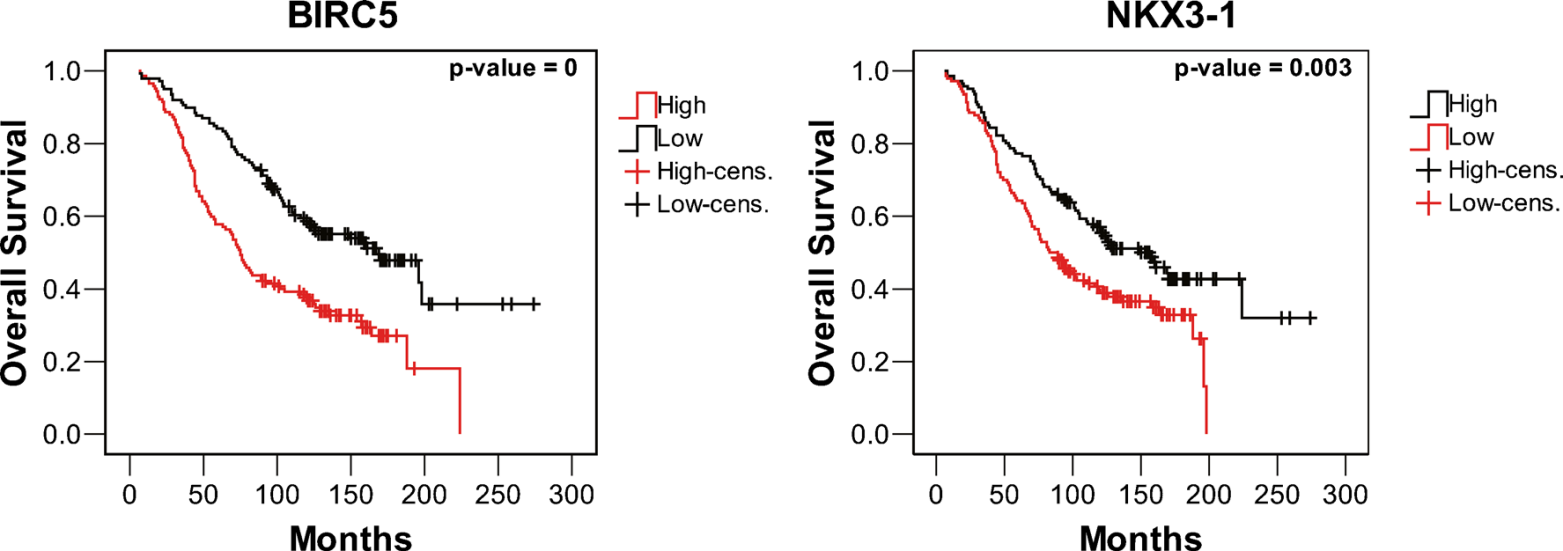
Kaplan–Meier survival analysis of prostate cancer biomarkers. Kaplan–Meier curves for the two markers with the most significant prognostic potential based on data from dataset GSE16560 are shown

In order to identify the prognostic potential of single markers, we performed Cox regression analyses using the same dataset. Figure 7 shows Kaplan–Meier survival curves for two markers, BIRC5/survivin [46] and NKX3-1 [47, 48], among those with the lowest logrank test *p* value (*p* = 0). Low-risk and high-risk cases show a significantly different survival, yet it is unlikely that differences as observed here could guide treatment decisions or follow-up screenings. The collection of Kaplan–Meier curves for all markers analyzed is available as Supplementary Fig. 1.

**Fig. 8:**
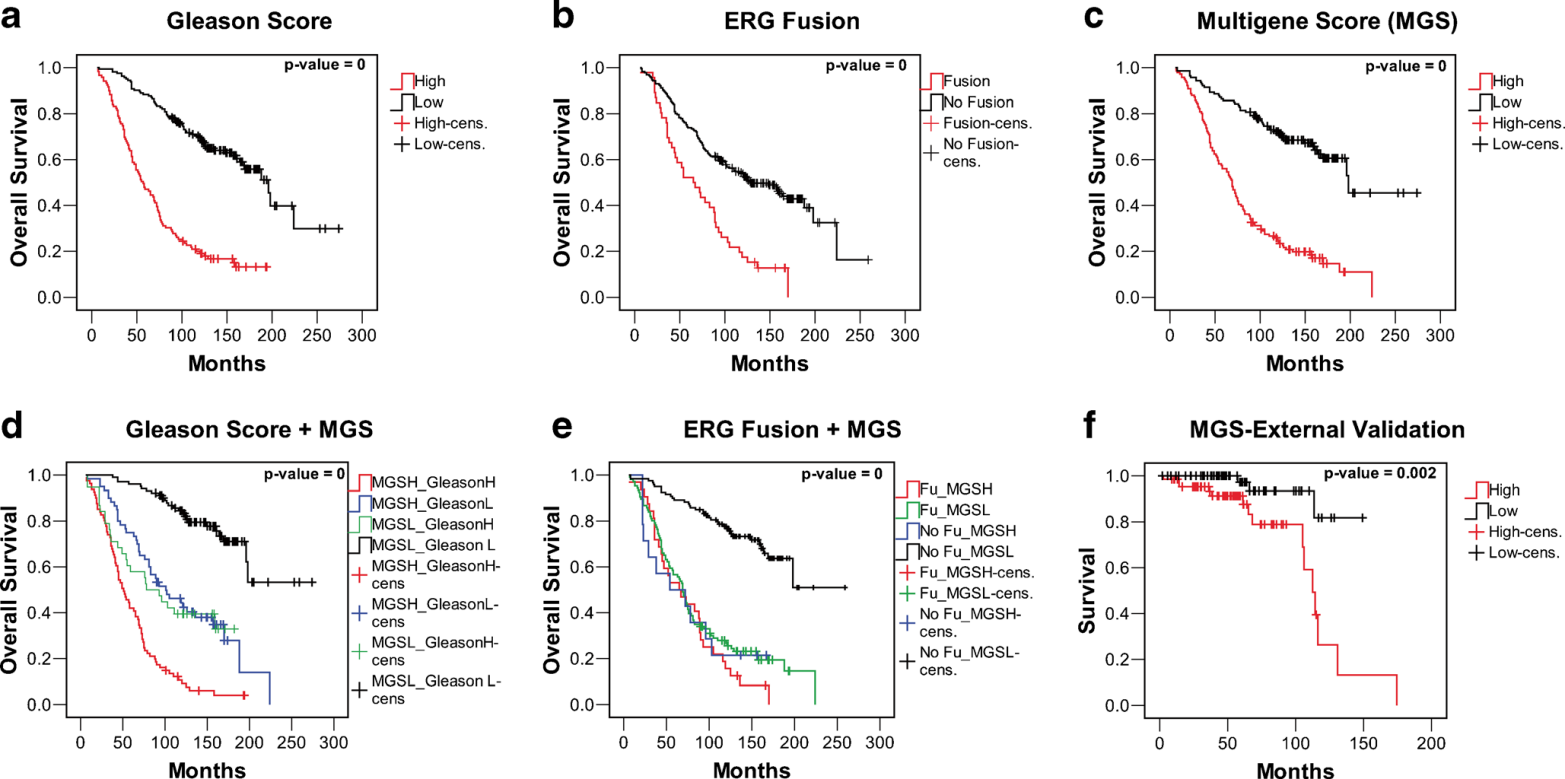
Multivariate models for prostate cancer prognosis. Prognostic prostate cancer biomarkers were combined in a prognostic multigene model using multivariate Cox regression analysis and dataset GSE16560 (see also Table 1). **a** Kaplan–Meier survival analysis applying Gleason score (low risk = <7 or 7 (=3 + 4), high risk = >7 or 7(=4 + 3)). **b** Kaplan–Meier survival analysis for cases with and without rearrangements of the gene ERG. **c** Kaplan–Meier survival analysis for the multigene score; cases are assigned according to the median of the score. **d** Combination of Gleason score with the multigene score (assignment of cases as above). **e** Combination of ERG fusion status and multigene score. **f** Application of the model to the external dataset GSE21034. The scores calculated on GSE16560 were directly applied

Multigene signatures have been shown to have a considerable prognostic power for several cancers [49]. We therefore asked whether a multivariate score of the markers that are significantly differentially expressed between low- and high-risk cases has a clinically relevant prognostic power. The multivariate model was calculated in a backward manner in order to leave as many genes in the model as possible. The genes selected for the model are the ATP-binding cassette (ABC) transporter with unknown substrate and function (ABCA5) [50], the engrailed homeobox gene 2 (EN2) [51], the 17-beta-hydroxysteroid dehydrogenase type 3 that converts androstenedione to testosterone (HSD17B3) [52], the NK3 homeobox 1, a negative regulator of epithelial cell growth in prostate tissue, (NKX3-1) [47], the signal transducer and activator of transcription 6 that mediates the anti-apoptotic effects of interleukin 4 (STAT6) [53], the E2F transcription factor 1 that is involved in the control of cell cycle progression (E2F1) [54], the folate hydrolase (prostate-specific membrane antigen) 1, a glutamate carboxypeptidase (FOLH1) [47], the proteasome (prosome, macropain) subunit, alpha type, 7 that plays a role in the cellular stress response by regulating hypoxia-inducible factor 1 alpha (PSMA7) [55], and the topoisomerase (DNA) II alpha (TOP2A) [54]. Table 1 shows the results of the multivariate analysis. A score indicating the strength of correlation between the expression of the given gene and survival (column B) is calculated for each gene. The global multigene score (MGS) is obtained by the sum of the expression values multiplied by the score assigned. The median value of the score is then used to classify the samples in low and high risk. Kaplan–Meier survival curves for the commonly used Gleason scoring system (Fig. 8a), the presence or absence of the fusions involving the V-Ets erythroblastosis virus E26 oncogene homolog (ERG; Fig. 8b), and the multigene score are plotted (Fig. 8c). All three prognostic measures yield risk classes with significantly different risk of death from prostate cancer (logrank test *p* = 0). The discrimination of high- and low-risk groups using the multigene score (Fig. 8c) is clearly superior to that observed for single genes (see Fig. 7 and Supplementary Fig. 1). The survival differences of the low- and high-risk groups are evident from the very beginning of follow-up. After 5 years, approximately 86 % of the low-risk cases and 56 % of the high-risk cases are alive, and after 10 years, these figures become 73 and 24 %, respectively. This analysis shows that the combined score can distinguish prostate cancer patients with a significantly different risk of death of prostate cancer, similar to what is obtained by Gleason scoring (Fig. 8a). ERG fusions found in 46 cases (226 cases without fusion, for 9 cases the fusion status is unknown) also confer a bad prognosis, yet the absence of a fusion is not a good indicator of an indolent evolution of the cancer (Fig. 8b).

**Table 1:** Description of the multivariate model of prognostic prostate cancer biomarkers

Cox multivariate model of prognostic markers for prostate cancer								
	*B*	SE	Wald	*df*	Sig.	Exp(*B*)	95.0 % CI for Exp(*B*)	
							Lower	Upper
ABCA5	−0.29815	0.121503	6.021354	1	0.014134	0.742191	0.584914	0.941757
EN2	−0.27537	0.128319	4.605327	1	0.031873	0.759288	0.590447	0.97641
HSD17B3	−0.22517	0.08482	7.047193	1	0.007939	0.798383	0.676102	0.942779
NKX3-1	−0.84309	0.259302	10.57136	1	0.001149	0.43038	0.258901	0.715436
STAT6	−0.31482	0.104022	9.159404	1	0.002474	0.729922	0.595296	0.894994
E2F1	0.232565	0.099174	5.499088	1	0.019026	1.261832	1.038925	1.532565
FOLH1	0.384885	0.142412	7.304128	1	0.00688	1.469446	1.111558	1.942562
PSMA7	0.789574	0.381355	4.286744	1	0.038411	2.202458	1.043033	4.650688
TOP2A	0.181014	0.070785	6.539388	1	0.010551	1.198432	1.043184	1.376784

The Gleason scoring system [56] is commonly used for prostate cancer prognosis. The combination of new molecular markers with the Gleason score must be assessed. We therefore calculated Kaplan–Meier survival curves for cases with a Gleason score below 7 or equal to 7 (=3 + 4) and cases with Gleason > 7 or equal to 7 (=4 + 3). The application of the combined marker score to the former cases shows an improved distinction of low- and high-risk cases (Fig. 8d) and cases with low Gleason score and low-risk multigene score show a clear difference in survival from the very beginning of follow-up as compared to cases where both scores indicate high risk. Cases with low and high Gleason score are further divided by the application of the MGS. This creates two intermediate groups (Gleason low, MGS high; Gleason high, MGS low) with similar Kaplan–Meier curves (Fig. 8d). Combination of the MGS with ERG fusion status shows that MGS low-risk cases without fusion have a good prognosis and cases with MGS high risk or the ERG fusion, or both, have a bad prognosis (Fig. 8e). Cases can be classified considering at high risk those cases that receive an indication of high risk by either the Gleason score or the MGS thus considering the intermediate cases as high risk. In this way, an additional 40 of 162 cases with Gleason 6 or 7 (=3 + 4) would be correctly identified as high-risk cases at the expense of 20 indolent cases that would be considered at high risk (Table 2). If instead Gleason 7 would be considered high risk with no regard of the status of the major and minor components of the tumor, additional 39 would be correctly identified as high risk yet 40 actually indolent cases would be classified at high risk. The combination of the MGS with Gleason can therefore be expected to slightly improve the assessment of cases with Gleason score of 6 or 7 (see also Table 2).

**Table 2:** Multigene score classification in relation to Gleason score

Multigene score classification in relation to Gleason score				
Total (281)	Indolent (116)		Lethal (165)	
Gleason score	MGS low	MGS high	MGS low	MGS high
6 (83)	47	*10*	14	**12**
7 = 3 + 4 (79)	30	*10*	11	**28**
7 = 4 + 3 (38)	7	3	9	19
8 (27)	7	1	5	14
9 (49)	0	1	10	38
10 (5)	0	0	0	5

Lethal cases with low Gleason score that are correctly classified as “high risk” using the MGS are indicated in bold, and indolent cases with low Gleason score that are incorrectly classified as “high risk” by MGS are indicated in italics

Calculation of multigene scores often leads to over-fitting yielding scores that strongly depend on the specific dataset on which they have been calculated. To avoid over-fitting, the dataset must be randomly divided into a training set on which the score is calculated and a test set to which the score is applied [57]. When we applied this strategy to the selected prostate biomarkers, the resulting risk classes show a significantly different risk (logrank test *p* = 0) in the training set. The same score yields a similar distinction when applied to the test set that also is statistically significant (logrank test *p* = 0.005) (Supplementary Fig. 2).

We further validated the MGS on an external dataset (GSE21034). The application of the MGS to this dataset also yielded risk classes with significantly different risks (*p* = 0.002; Fig. 8f). This dataset is based on a completely different array type (exon arrays) and probe design. Thus, the application of the score calculated on the data derived from a different platform can lead to an underestimation of the discrimination power of the classifier.

**Fig. 9:**
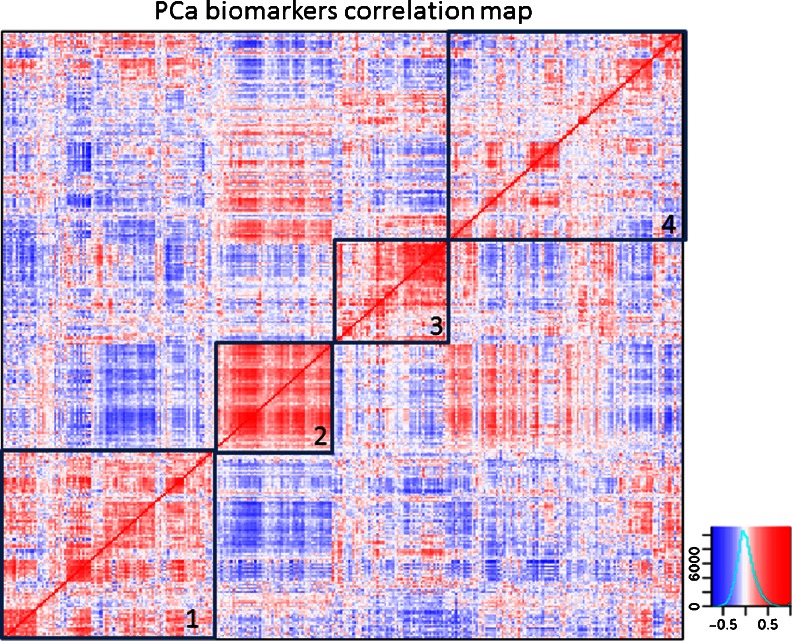
Correlation map of prostate cancer biomarkers. The expression correlation of the prostate cancer biomarkers is calculated and plotted as a heat map. Strong correlations are indicated by a color code (*blue*, <−0.5; *red*, >0.5). Arbitrarily selected clusters containing markers with high correlation are indicated by *black squares* (1–4): Cluster 1 – enrichment of angiogenesis-related genes; Cluster 2 – enrichment of extracellular matrix- and matrix metalloproteinases; Cluster 3 – enhanced peptidase activity and kallikreins; Cluster 4 – enrichment for epithelial–mesenchymal transition

Finally, we asked whether the many biomarkers identified are functionally interrelated or independent. For this purpose, we performed a correlation analysis using more than 10,000 microarray gene expression data sets. Figure 9 shows the correlation heat map. There is generally a considerable correlation (*r* > 0.5) of any marker with several others. In order to understand whether correlated markers belong to groups of genes that exert similar functions or participate in similar biological processes, we analyzed the enrichment of gene ontology terms using the Database for Annotation, Visualization and Integrated Discovery [58] for the four predominant, yet arbitrarily selected clusters of the correlation map. Cluster 1 (Fig. 9) shows enrichment of several angiogenesis-related gene ontology (GO) annotations; most of which contain the angiogenic factors VEGFA and VEGFC as well as HGF. Cluster 2 shows an enrichment of extracellular matrix-related GO terms dominated by several matrix metalloproteinases (MMP2, MMP9, and MMP13). Cluster 3 shows GO terms related to peptidase activity containing several kallikreins and in cluster 4 epithelial–mesenchymal transition-related GO categories predominate (see Supplementary Table 3 for complete data). These four biological processes are clearly related to cancer development and progression. Interestingly, cell growth and proliferation are not among the most enriched GO terms despite the important role of cell proliferation in cancer prognosis.

## Discussion

The identification of prostate cancer biomarkers is a very active field of research. The mean impact factor of the journals in which the prostate cancer biomarkers analyzed here were published is 5.41 (range 0–18.97) reflecting the relatively high attention that the scientific community is giving to this research. The markers that show some value in this meta-analysis are published in journals with a mean impact factor of 4.63 (range 0–8.234) and 5.72 (range 4.411–7.338) for potential diagnostic and prognostic markers, respectively. Hence, there is no evidence of valid markers being published in journals with an impact factor higher than the mean.

The interest in prostate cancer biomarkers derives from the high incidence of this disease and the considerable variability in aggressiveness that ranges from almost benign to life threatening. Yet, even more emphasis in the field stems from the debate on the value of PSA screening that has culminated in the recent recommendation against screening issued by U.S. Preventive Services Task Force [7]. The balance to be found between benefits (saved lives) and costs (unnecessary surgery) could be greatly influenced by better markers. More effective biomarkers would improve the discrimination between healthy and tumor tissue and, perhaps even more importantly, allow for improved prognostication that could identify indolent cancers and limit surgery/intervention to patients who are at risk to die from the disease.

We have extracted 380 markers from the literature of 10 years and we analyzed these markers using publicly available datasets for prostate cancer. This approach has clear limits: (a) only gene expression data are used, (b) not all the data have been raised in controlled studies aimed at the identification of prostate cancer biomarkers, and (c) not all markers are represented in the dataset used (in particular, the non-coding RNA marker PCA3/DD3 [14] that is widely used in combination with PSA was not present in the dataset analyzed). Our study can therefore not exclude that some of the markers might perform much better if analyzed at the protein level since mRNA expression and protein expression are separated by several levels of regulation or that the markers not represented in the dataset used might perform better than those present. The aim of our study is to verify whether the efforts of 10 years of research can be condensed in a multigene prognostic classifier. In addition, this study raises concern on markers that are widely expressed and whose expression levels, even when protein expression is analyzed, are likely to be influenced by expression in other tissues. Finally, this study indicates how in silico analyses should be integrated in early phases of biomarker development in order to avoid unnecessary laboratory work.

The evaluation of the markers for a potential application in the diagnosis of prostate cancer did not yield evidence of any new marker that might substitute or complement PSA. Several markers show a more differential expression between normal and neoplastic prostate tissue than PSA yet, in contrast to the latter, they are expressed by several other normal and neoplastic tissues as well. The wide variability of expression of these markers with overlapping ranges for normal and tumor tissue makes the diagnostic assessment of the single patient difficult. It can therefore not be expected that any of these markers can resolve the problems associated with PSA-based early diagnosis of prostate cancer.

The validation of these mRNAs as prognostic markers yields a series of candidates that can discriminate cases with higher and lower risk, yet none of them appears to be clinically relevant. In addition to statistical significance, the marker sought should discriminate risk classes that deserve different therapeutic approaches. Minor yet statistically significant differences are irrelevant for the treatment decision. The combination of those markers that contribute independently to the risk assessment in a multivariate model appears to yield a discrimination of high- and low-risk cases that could be helpful in the clinics. But if the model is combined with the Gleason score, most of its potential disappears since there are few cases where Gleason score and the multigene model are strongly discordant (i.e., Gleason 6 and multigene high risk). The addition of the MGS mainly affects classification of Gleason score 7 cases.

Chen and coworkers recently reported on a seven-gene prognostic classifier for prostate cancer that they developed applying a preselection of samples mainly composed of tumor cells [59]. Similar to what we observe here, the seven-gene signature adds little to Gleason classification. The preselection procedure excludes many samples since prostate cancers typically contain a large stromal component. This also reduces the clinical applicability. Concordance of gene expression signatures with the prognostic Gleason score that we observe here has been observed by several groups [59, 60]. A nine-gene signature has been developed for the analysis of mRNAs isolated from whole blood cells [61]. This signature distinguishes rapidly progressing cancers among already castration-resistant prostate cancers and can therefore not be considered a general prognostic signature. Most of the patients in this cohort died during the follow-up of 36 months [61].

The question arises of why multigene signatures should work for breast cancer [62] but, at least so far, not or much less so for prostate cancer [38]? Several aspects of prostate cancer biology could contribute to an answer:

1. The main discriminator of high- and low-risk breast cancers is proliferation, and in fact, the simple assessment of the proliferative potential of breast cancers using KI-67 or aurora kinase A as markers performs almost as well as current multigene signatures [63]. Most prostate cancers have a particularly slow progression, and proliferation might be less prognostic in prostate cancer than in breast cancer.
2. Multifocal presentation and focal heterogeneity of prostate cancer may lead to sampling errors for prognostic assessment much more frequently in prostate cancer as compared to breast cancer.
3. Breast cancers derive from two different cell populations, luminal or basal cells, and the cell type they derive from determines most of the metastatic risk [64]. Perhaps prostate cancers derive from a more homogenous cell population, giving rise to a more homogeneous progression scheme.
4. The introduction of PSA screening has led to the identification of many low-risk cases that would not have been detected without screening. The numeric imbalance between low- and high-risk cases can make the identification of the latter more difficult (yet, this is not the case for the dataset on which we validated the prognostic power here).
5. Prostate cancer therapy is relatively successful leading to extended survival even of cases with largely dedifferentiated cells (high Gleason score). Death from prostate cancer is due to resistance to therapy (i.e., androgen-independent growth) that depends on acquired molecular alterations not present at the time of first diagnosis.
6. Gene expression profiles are dominated by transcription events that determine cell morphology. The influence of cell morphology on tumor progression might already be optimally assessed by the Gleason scoring system.
7. Tumor progression and metastasis are intrinsically stochastic. In the absence of other determinants, prostate cancer progression follows probability.
8. Important determinants of prostate cancer progression are not or not reliably reflected by gene expression. Identification of new mutations might allow for the identification of high-risk classes in analogy to the effect of ERG fusion genes [65, 66].

The identification of prostate cancer markers remains a challenge to be pursued by adding new technological approaches. Array comparative genome hybridization has revealed structural and numerical genomic alterations that correlate with outcome independently of Gleason scoring [39], and next generation sequencing (exome sequencing) has revealed a series of new mutations in prostate cancer whose prognostic value has yet to be determined [39, 65, 66]. Further, expression of new potential markers such as microRNAs and other non-coding RNAs may provide new avenues of investigation [67] as well as use of novel approaches for metabolic markers [68].

## Electronic supplementary material

Below is the link to the electronic supplementary material.

Supplementary Fig. 1 Kaplan–Meier survival analysis of all prostate cancer biomarkers on dataset GSE16560 (PDF 839 kb)

Supplementary Fig. 2 Kaplan–Meier curves for the multigene score on a random split of the dataset GSE16560 to create training and test sets (JPEG 284 kb)

High-resolution image (TIFF 573 kb)

Supplementary Table 1 List of prostate cancer biomarkers considered for the present study with bibliographic references (PDF 632 kb)

Supplementary Table 2 Expression of prostate cancer markers in healthy and tumoral prostate tissue (XLSX 56 kb)

Supplementary Table 3 Functional categories of the markers in clusters of expression correlated markers (XLSX 110 kb)
