# Supplementary material for: Validation of proposed prostate cancer biomarkers with gene expression data: a long road to travel
Source: Cancer Metastasis Rev. 2014 Jan 30;33(2):657–71. doi: 10.1007/s10555-013-9470-4 (PMC4113682; doi:10.1007/s10555-013-9470-4)
Supplement: Supplementary file 1 — Kaplan–Meier survival analysis of all prostate cancer biomarkers on dataset GSE16560 (PDF 839 kb) [file 10555_2013_9470_MOESM1_ESM.pdf]

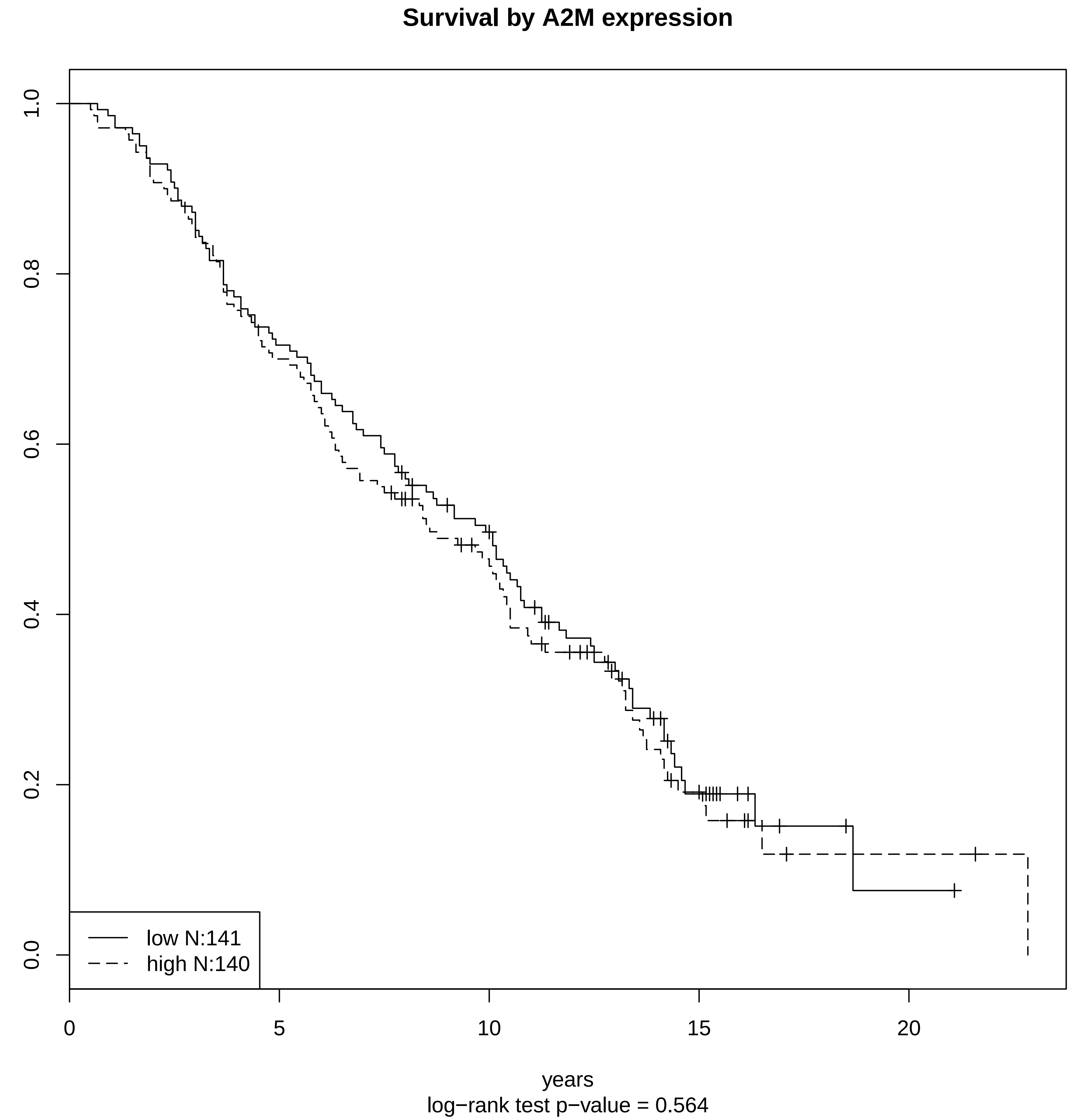

Survival by A2M expression
1.0
0.8
0.6
0.4
0.2
0.0
0
5
10
15
20
low N:141
high N:140
years
log−rank test p−value = 0.564

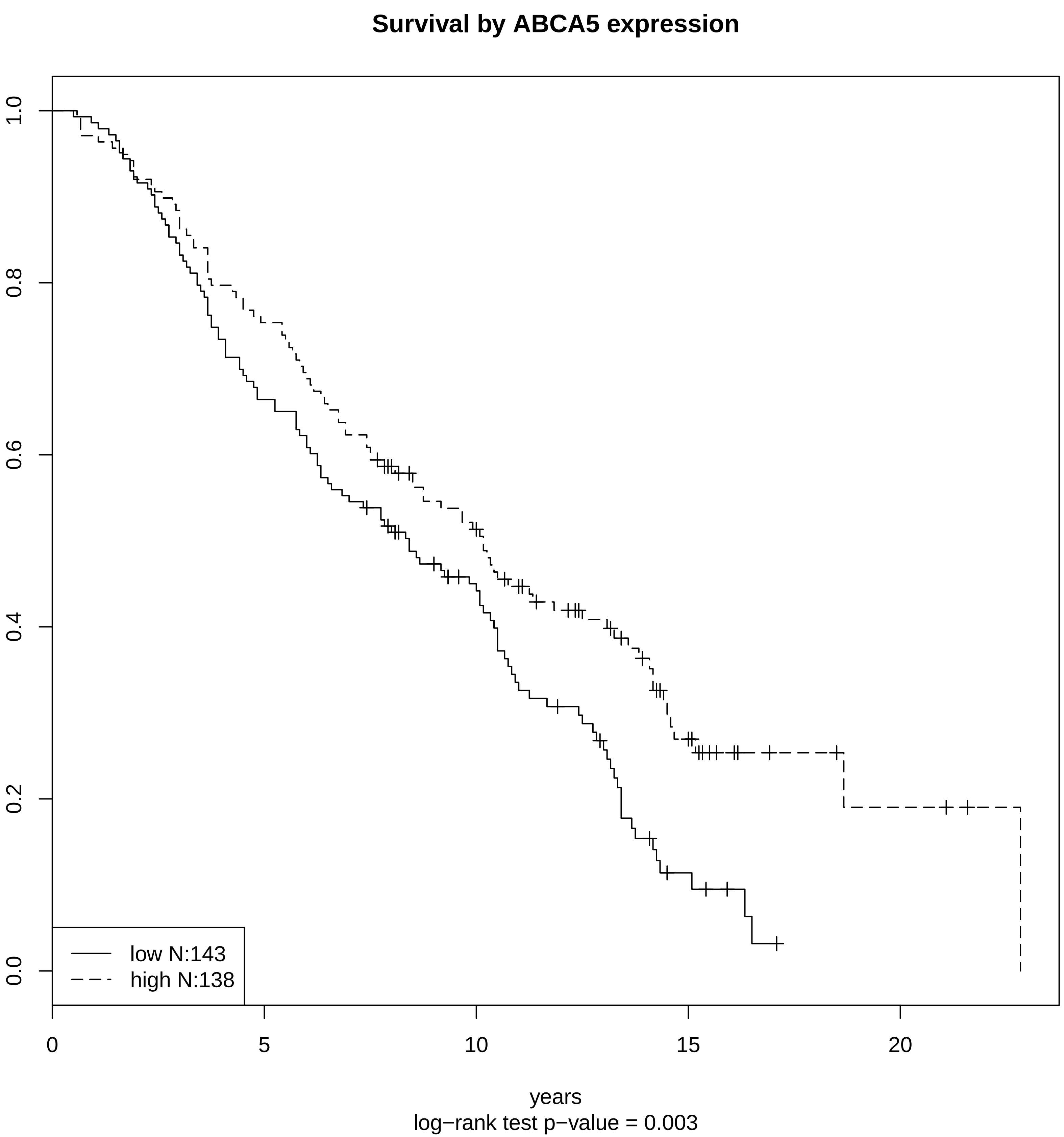
Survival by ABCA5 expression
1.0
0.8
0.6
0.4
0.2
0.0
0
5
10
15
20
years
log−rank test p−value = 0.003
low N:143
high N:138

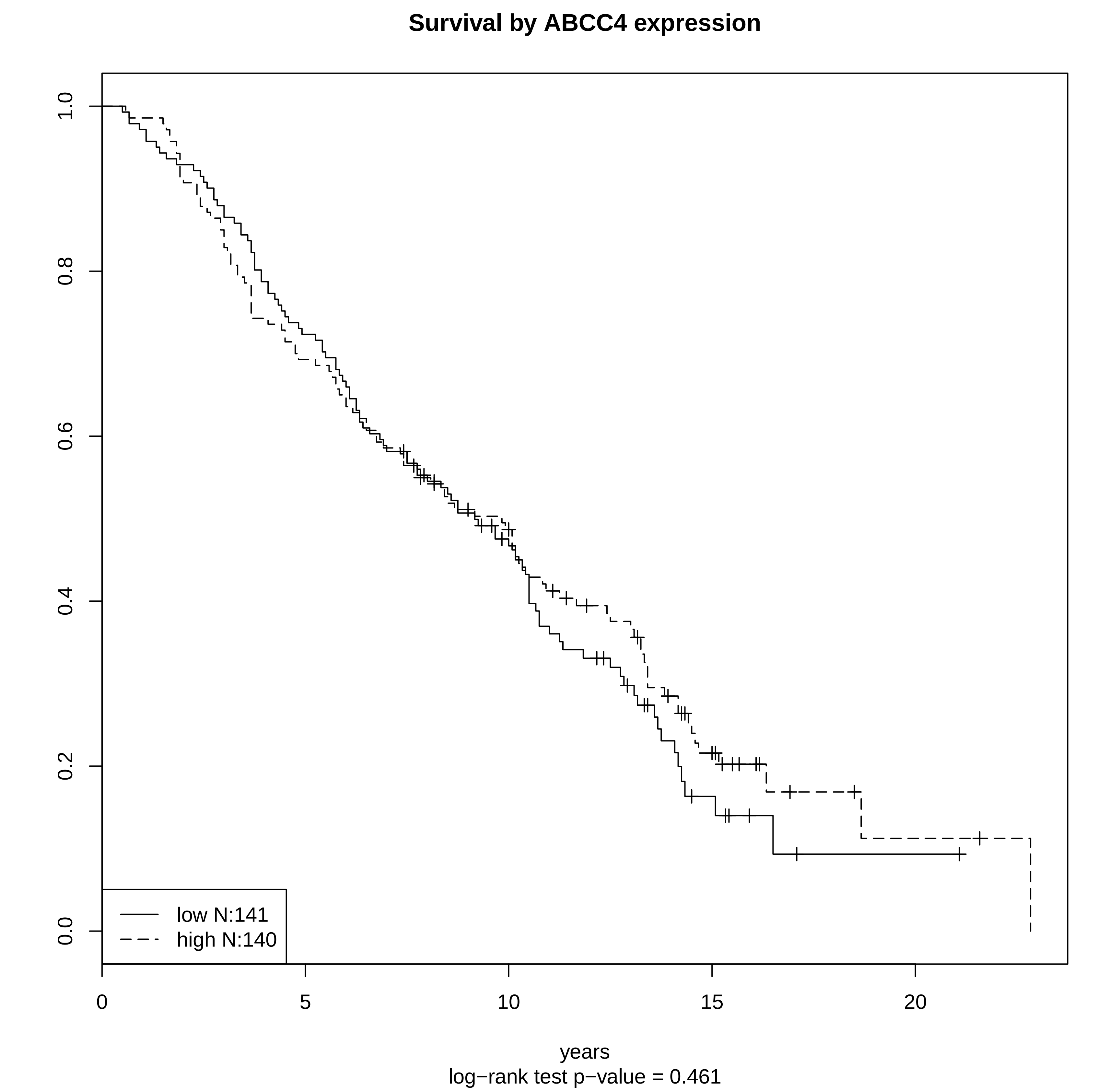
Survival by ABCC4 expression
1.0
0.8
0.6
0.4
0.2
0.0
0
5
10
15
20
low N:141
high N:140
years
log−rank test p−value = 0.461

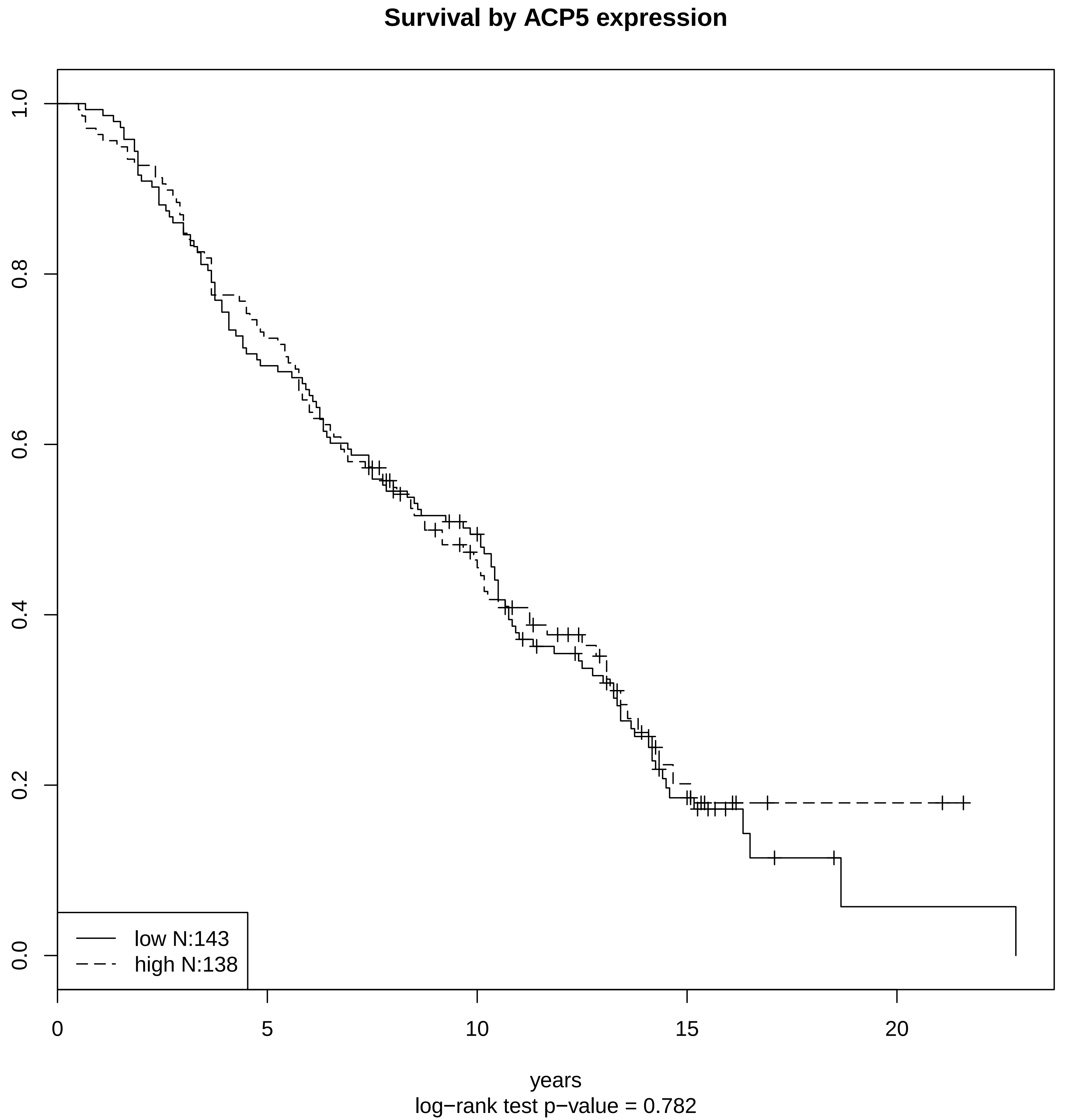

Survival by ACP5 expression
1.0
0.8
0.6
0.4
0.2
0.0
0
5
10
15
20
low N:143
high N:138
years
log−rank test p−value = 0.782

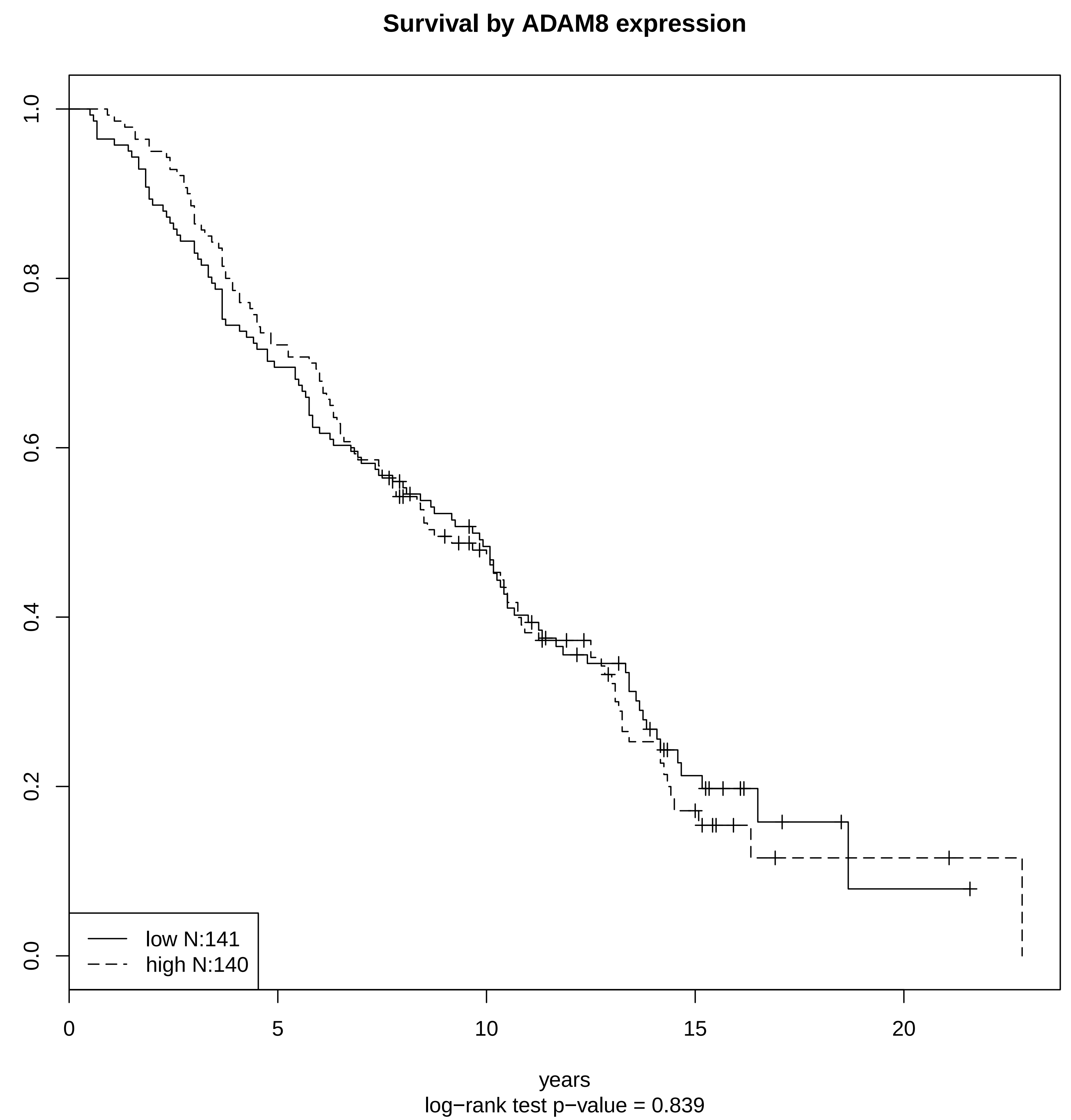
Survival by ADAM8 expression
1.0
0.8
0.6
0.4
0.2
0.0
0
5
10
15
20
years
log−rank test p−value = 0.839
low N:141
high N:140

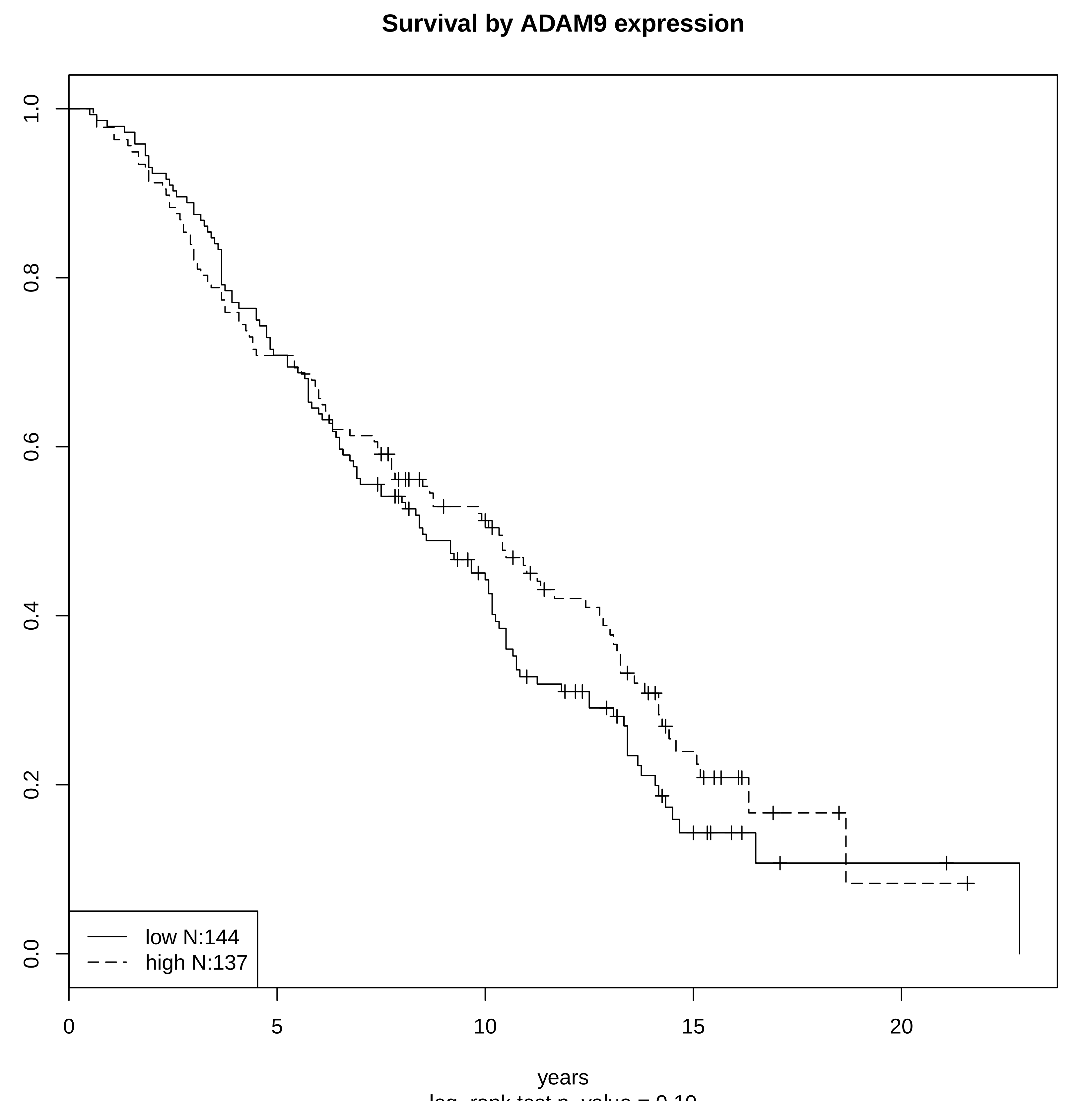
Survival by ADAM9 expression
1.0
0.8
0.6
0.4
0.2
0.0
0
5
10
15
20
low N:144
high N:137
years
log−rank test p−value = 0.19

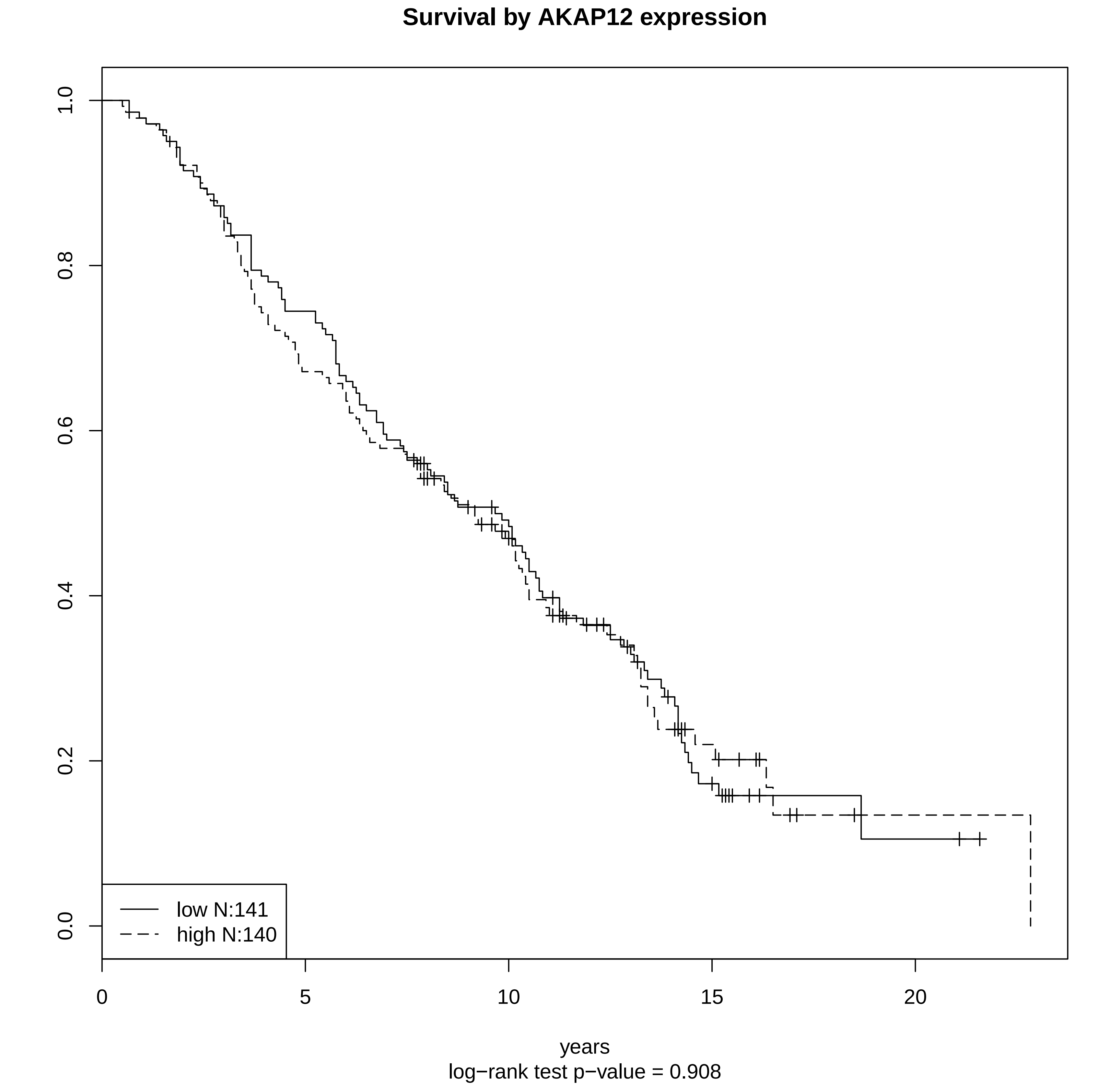

Survival by AKAP12 expression
1.0
0.8
0.6
0.4
0.2
0.0
0
5
10
15
20
low N:141
high N:140
years
log−rank test p−value = 0.908

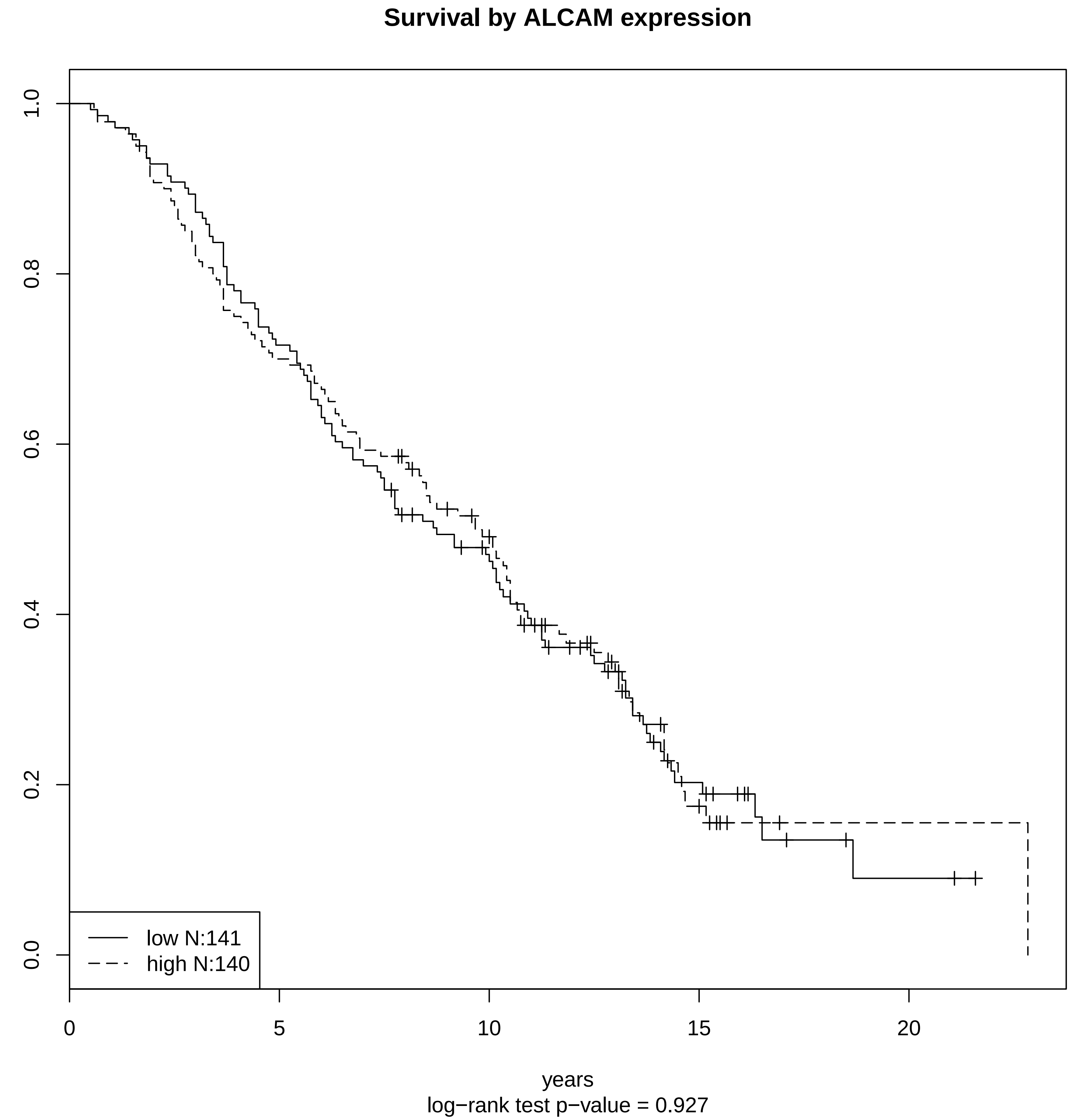

Survival by ALCAM expression
1.0
0.8
0.6
0.4
0.2
0.0
0
5
10
15
20
low N:141
high N:140
years
log−rank test p−value = 0.927

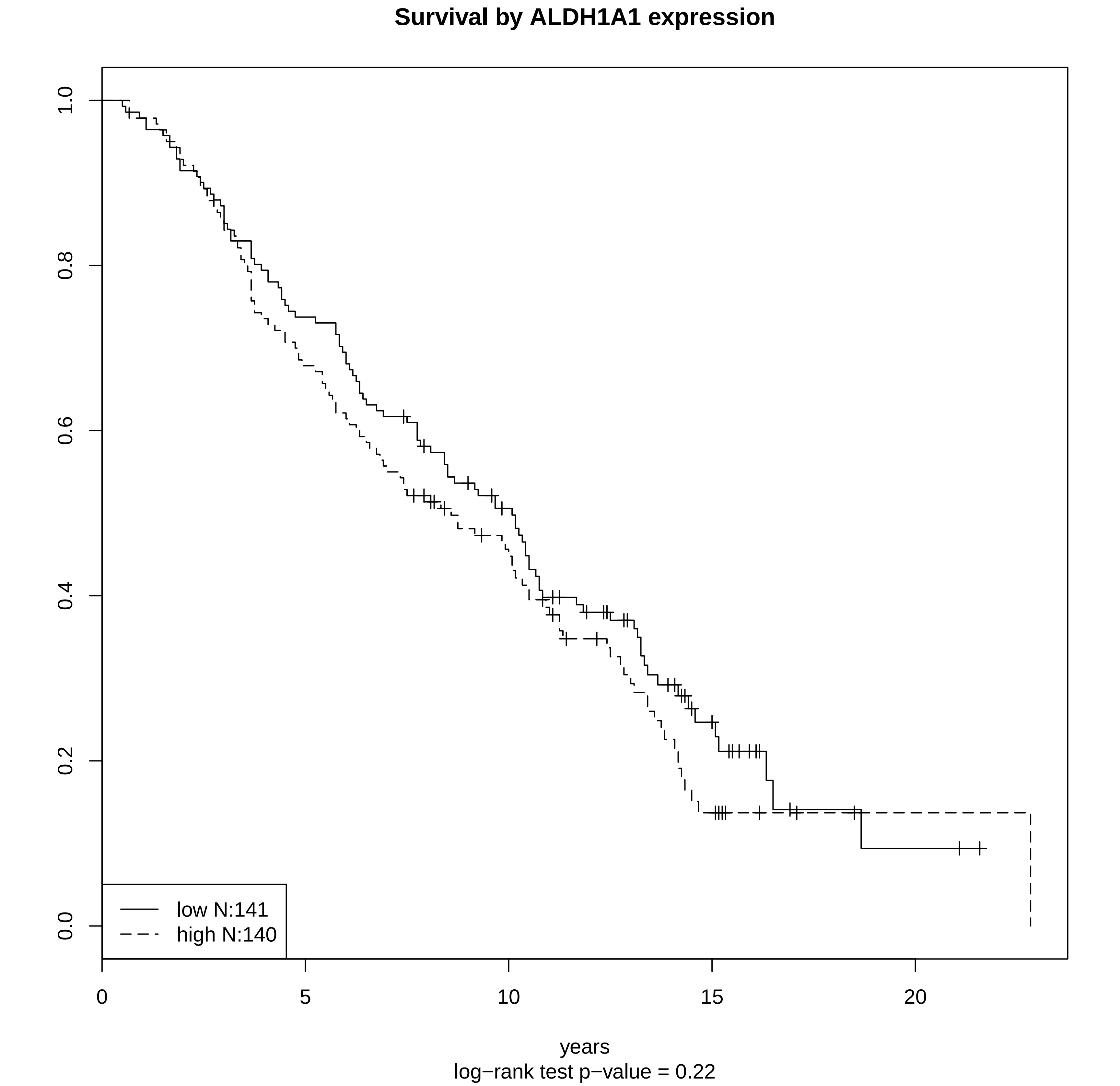

Survival by ALDH1A1 expression
1.0
0.8
0.6
0.4
0.2
0.0
0
5
10
15
20
low N:141
high N:140
years
log−rank test p−value = 0.22

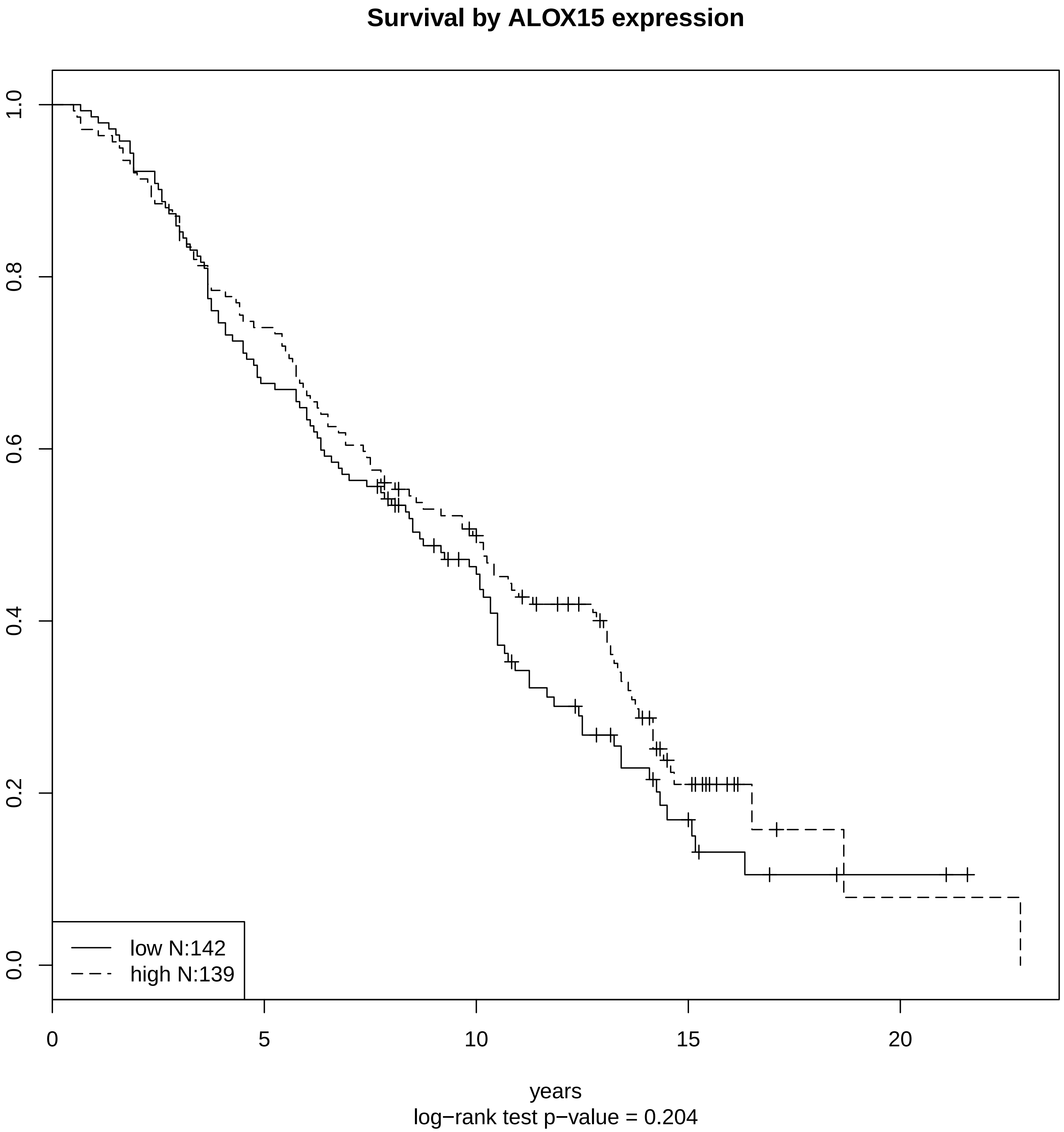
Survival by ALOX15 expression
1.0
0.8
0.6
0.4
0.2
0.0
0
5
10
15
20
years
log−rank test p−value = 0.204
low N:142
high N:139

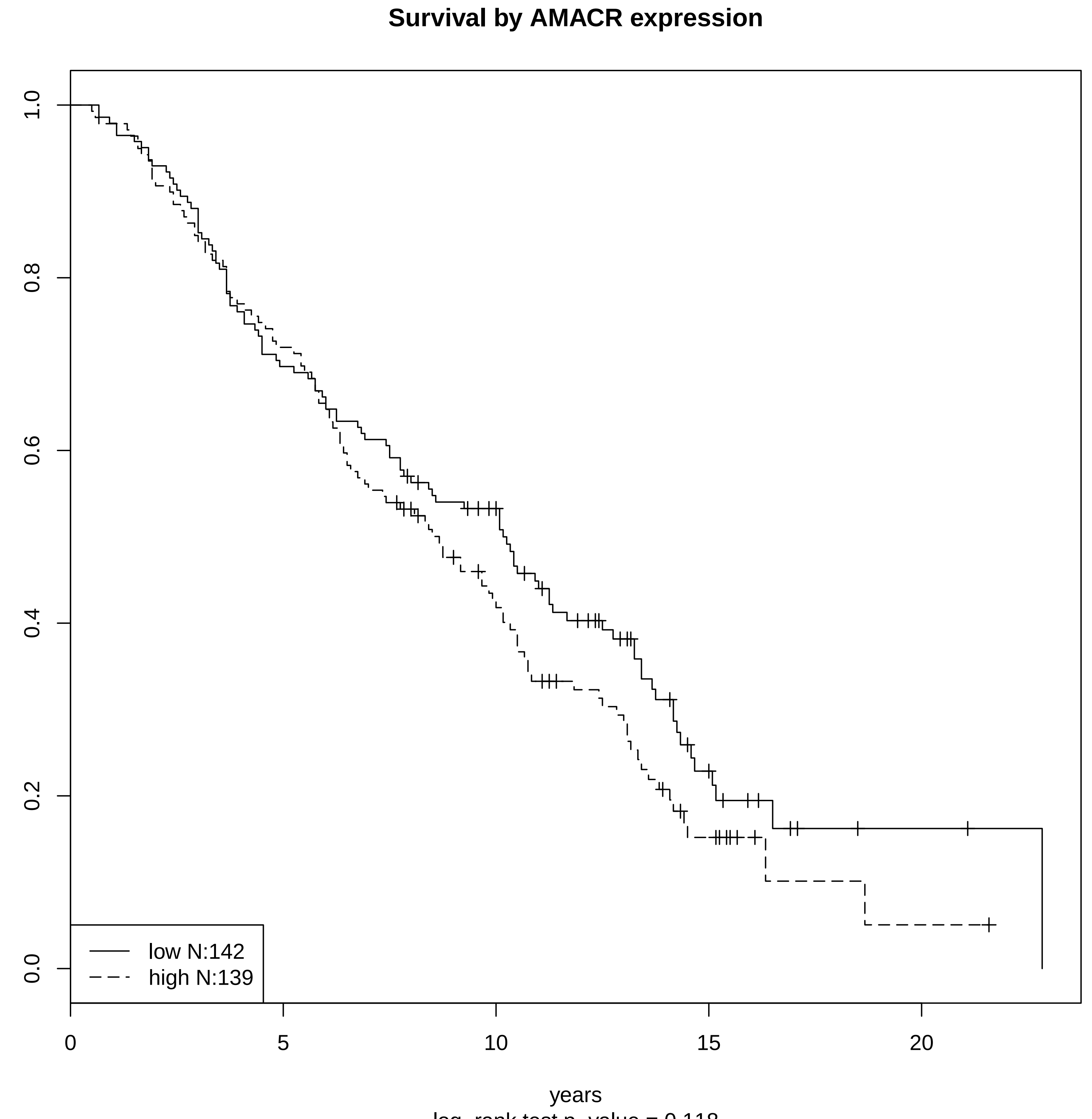

Survival by AMACR expression
1.0
0.8
0.6
0.4
0.2
0.0
0
5
10
15
20
low N:142
high N:139
years
log−rank test p−value = 0.118

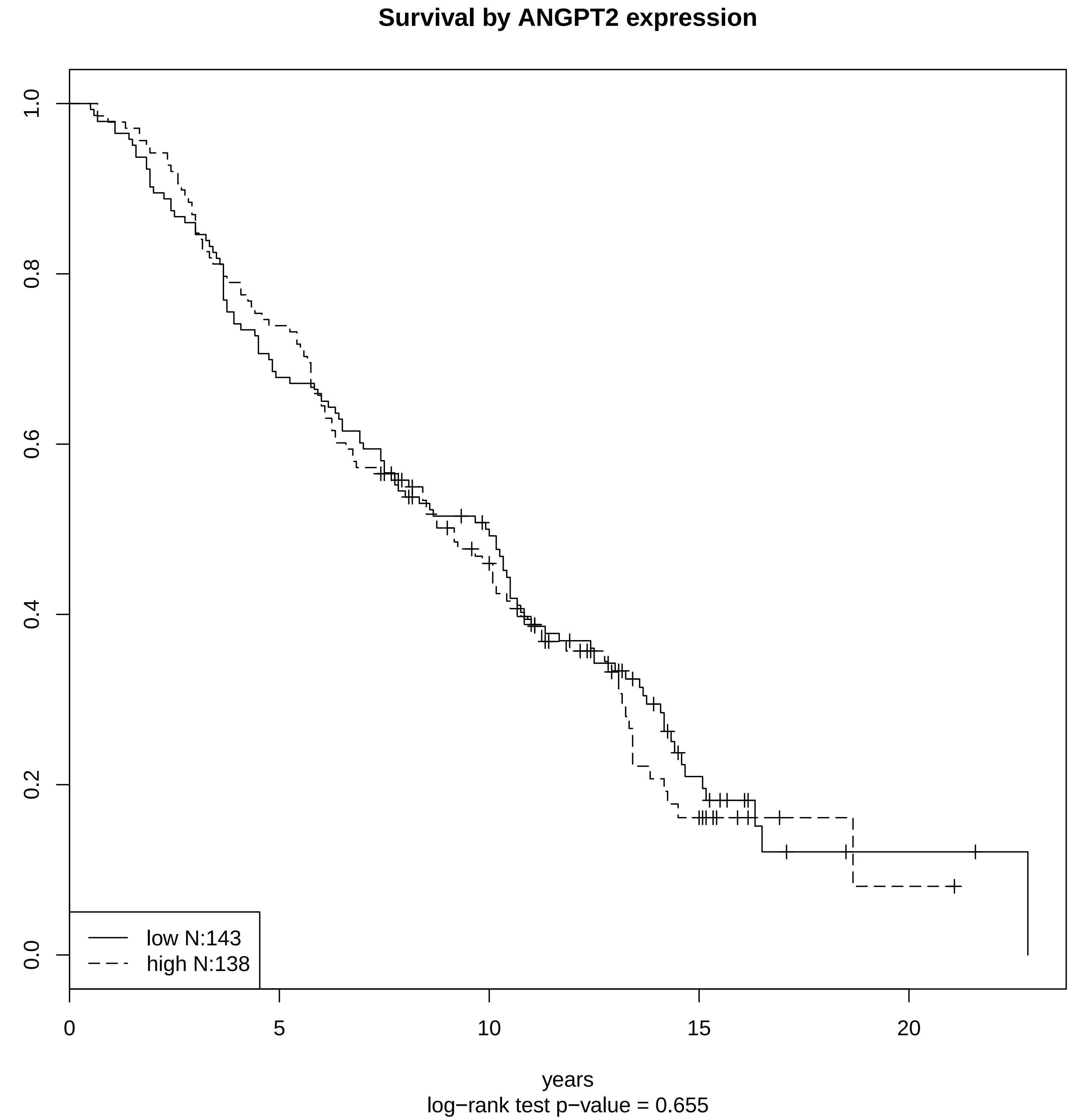
Survival by ANGPT2 expression
1.0
0.8
0.6
0.4
0.2
0.0
0
5
10
15
20
low N:143
high N:138
years
log−rank test p−value = 0.655

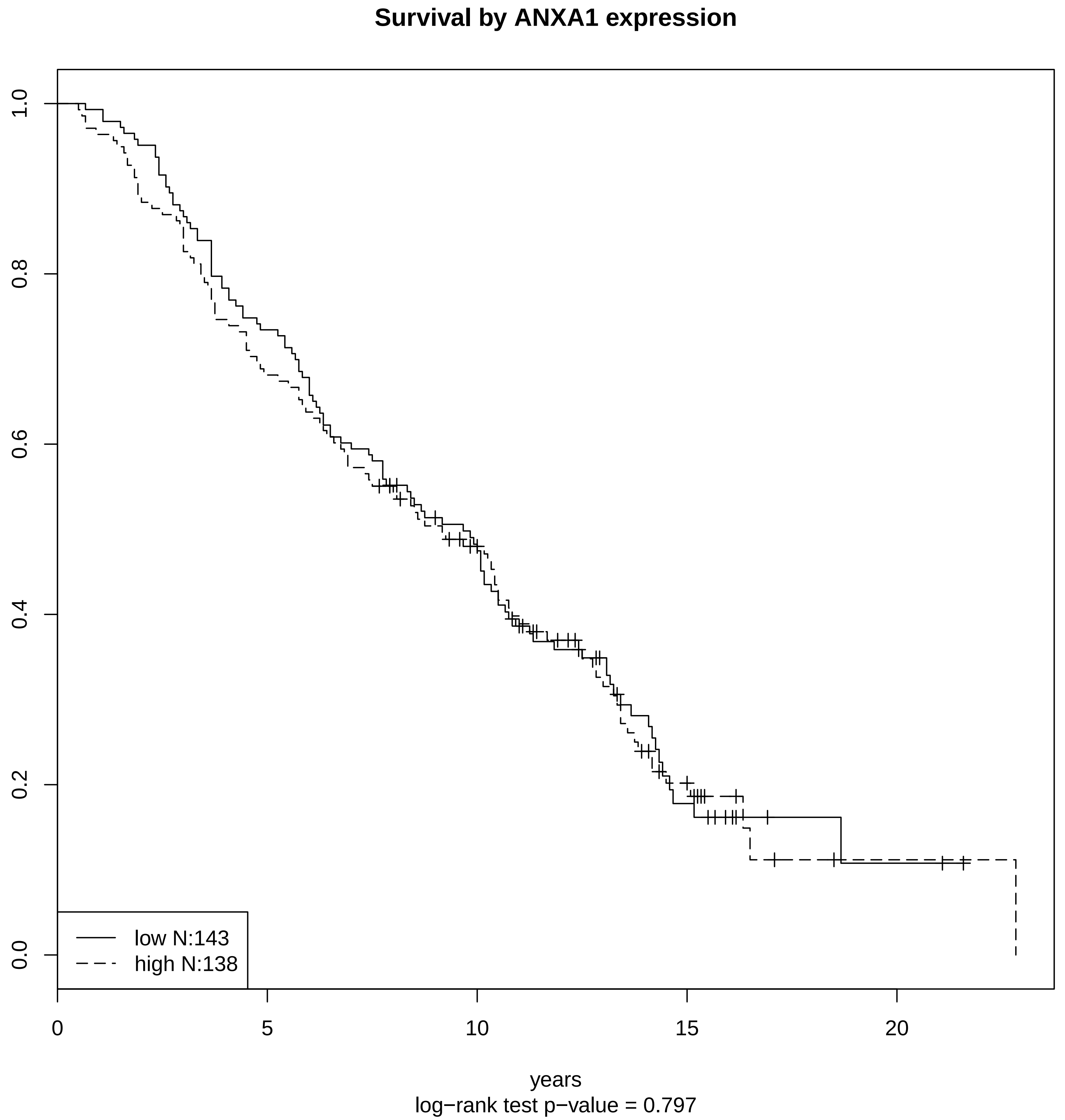
Survival by ANXA1 expression
1.0
0.8
0.6
0.4
0.2
0.0
0
5
10
15
20
years
low N:143
high N:138
log−rank test p−value = 0.797

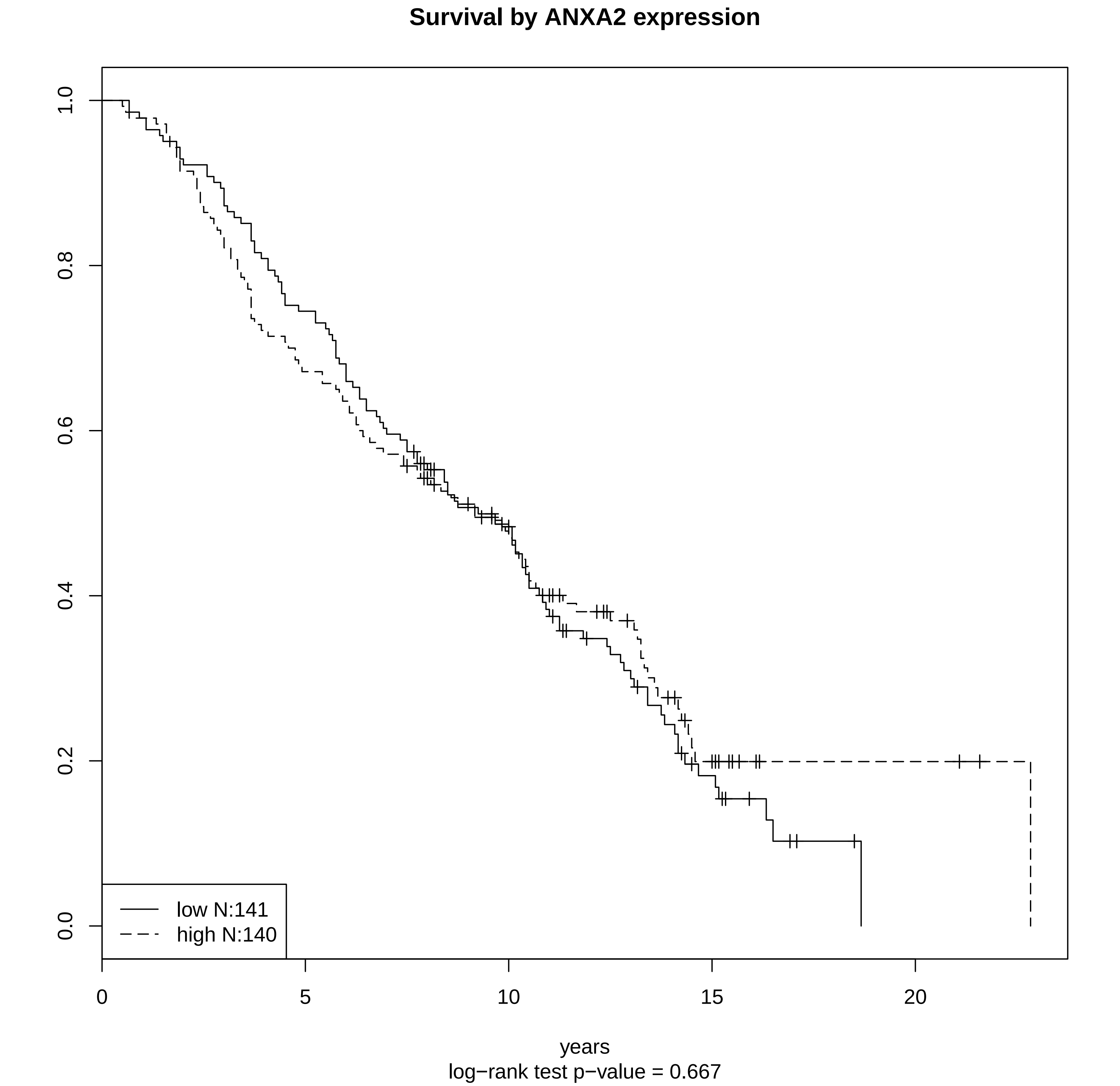

Survival by ANXA2 expression
0.0
0.2
0.4
0.6
0.8
1.0
0
5
10
15
20
years
log−rank test p−value = 0.667
low N:141
high N:140

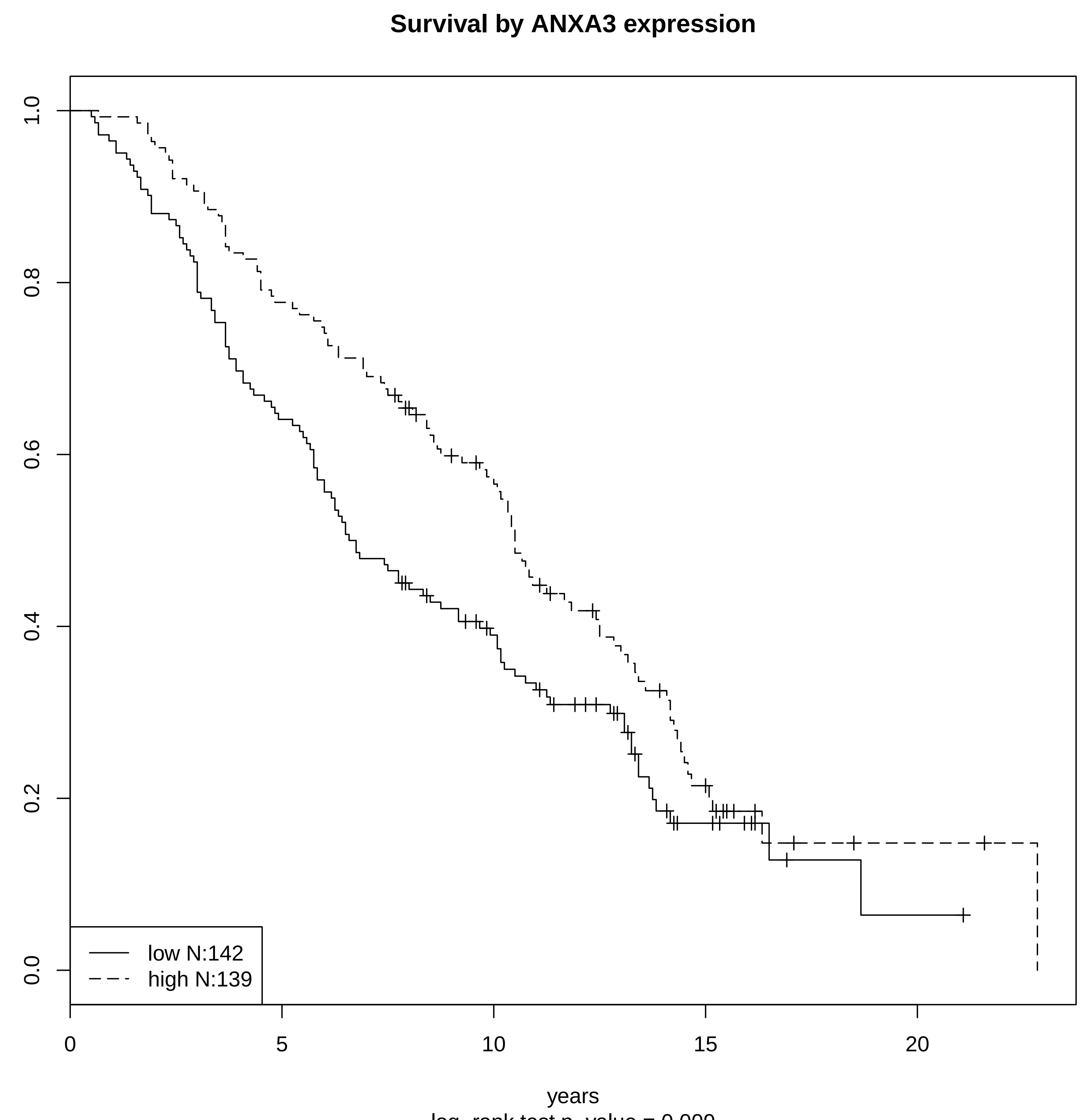

Survival by ANXA3 expression
1.0
0.8
0.6
0.4
0.2
0.0
0
5
10
15
20
low N:142
high N:139
years
log−rank test p−value = 0.009

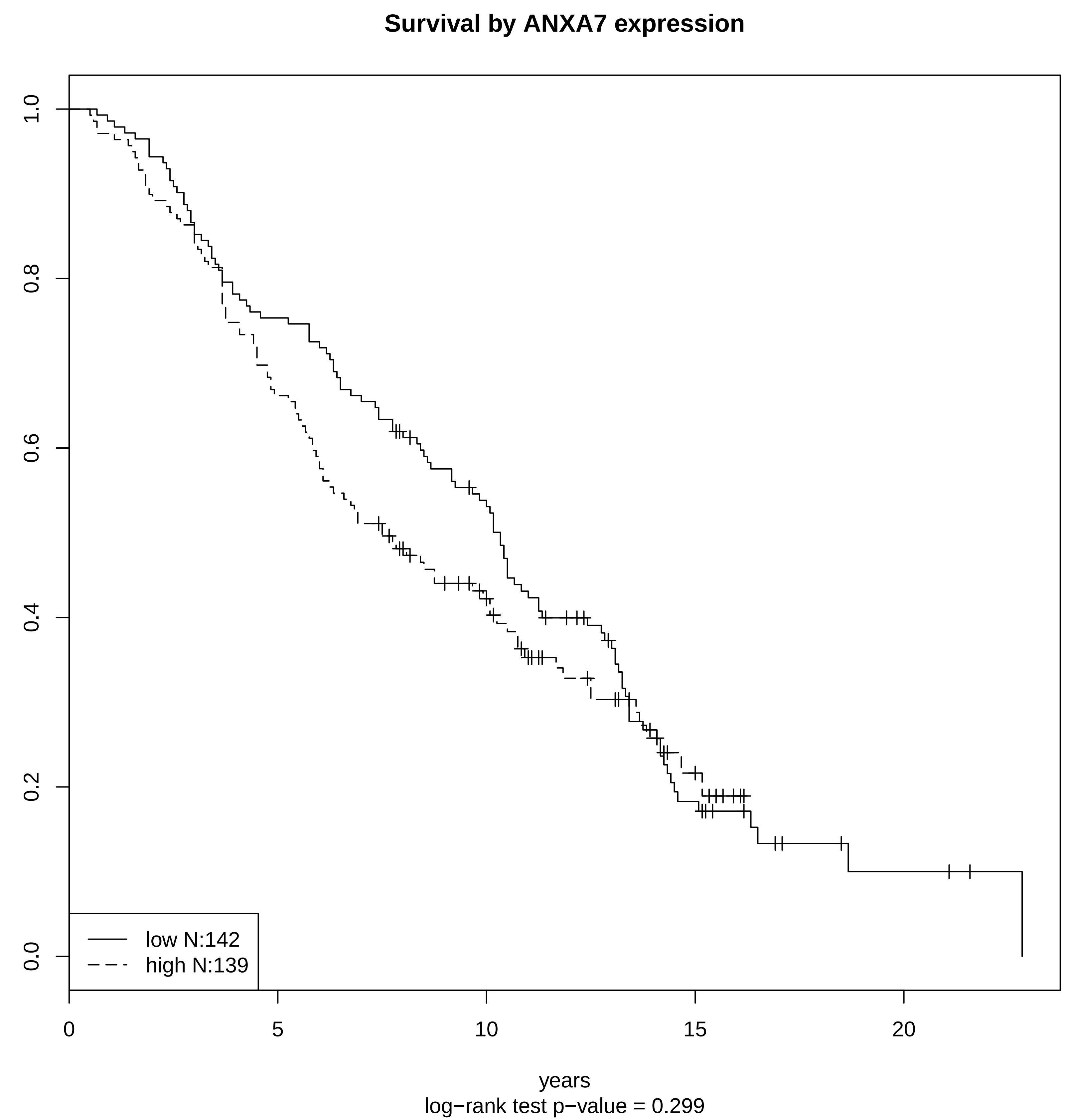

Survival by ANXA7 expression
1.0
0.8
0.6
0.4
0.2
0.0
0
5
10
15
20
low N:142
high N:139
years
log−rank test p−value = 0.299

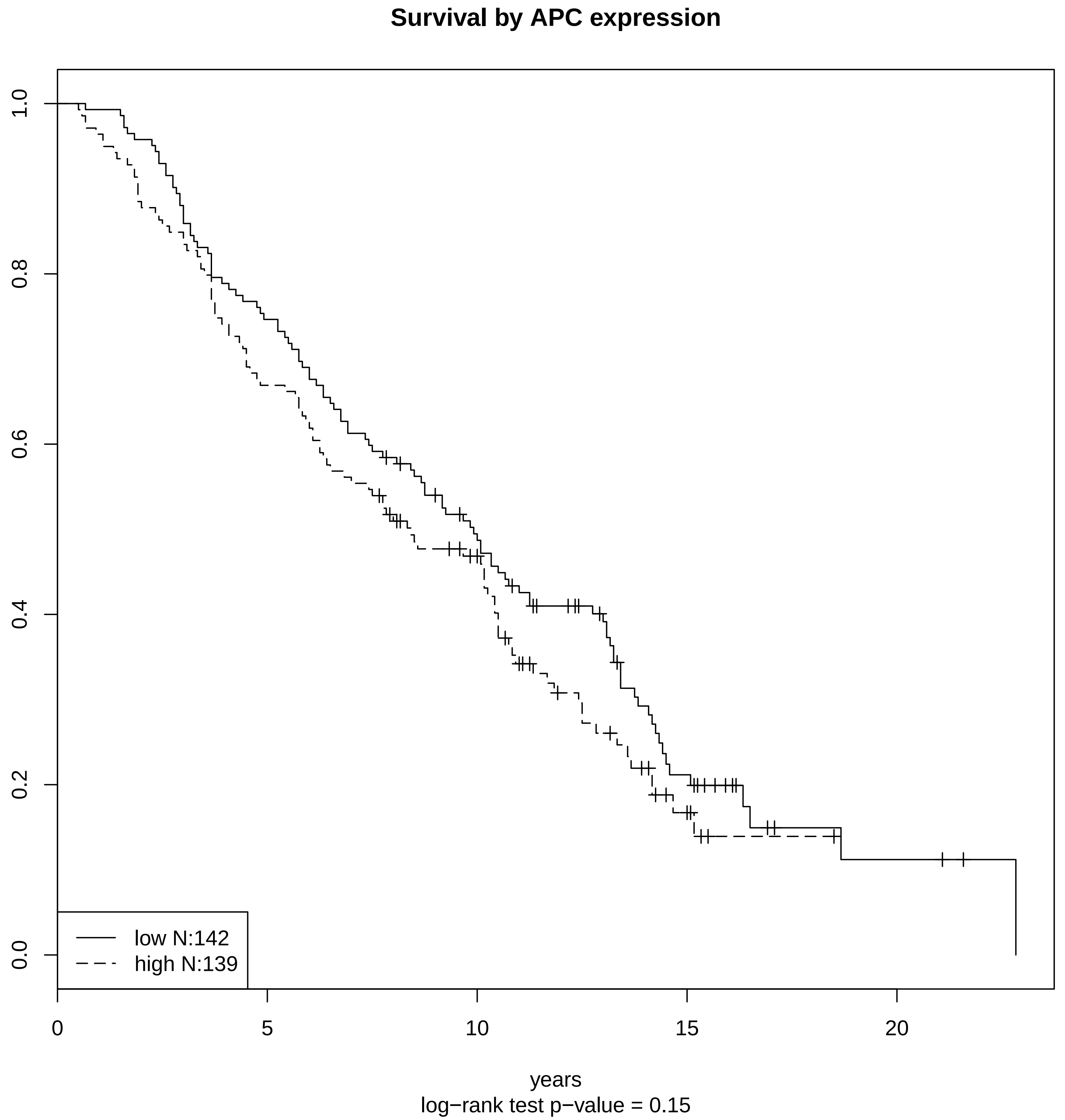

Survival by APC expression
1.0
0.8
0.6
0.4
0.2
0.0
0
5
10
15
20
years
log−rank test p−value = 0.15
low N:142
high N:139

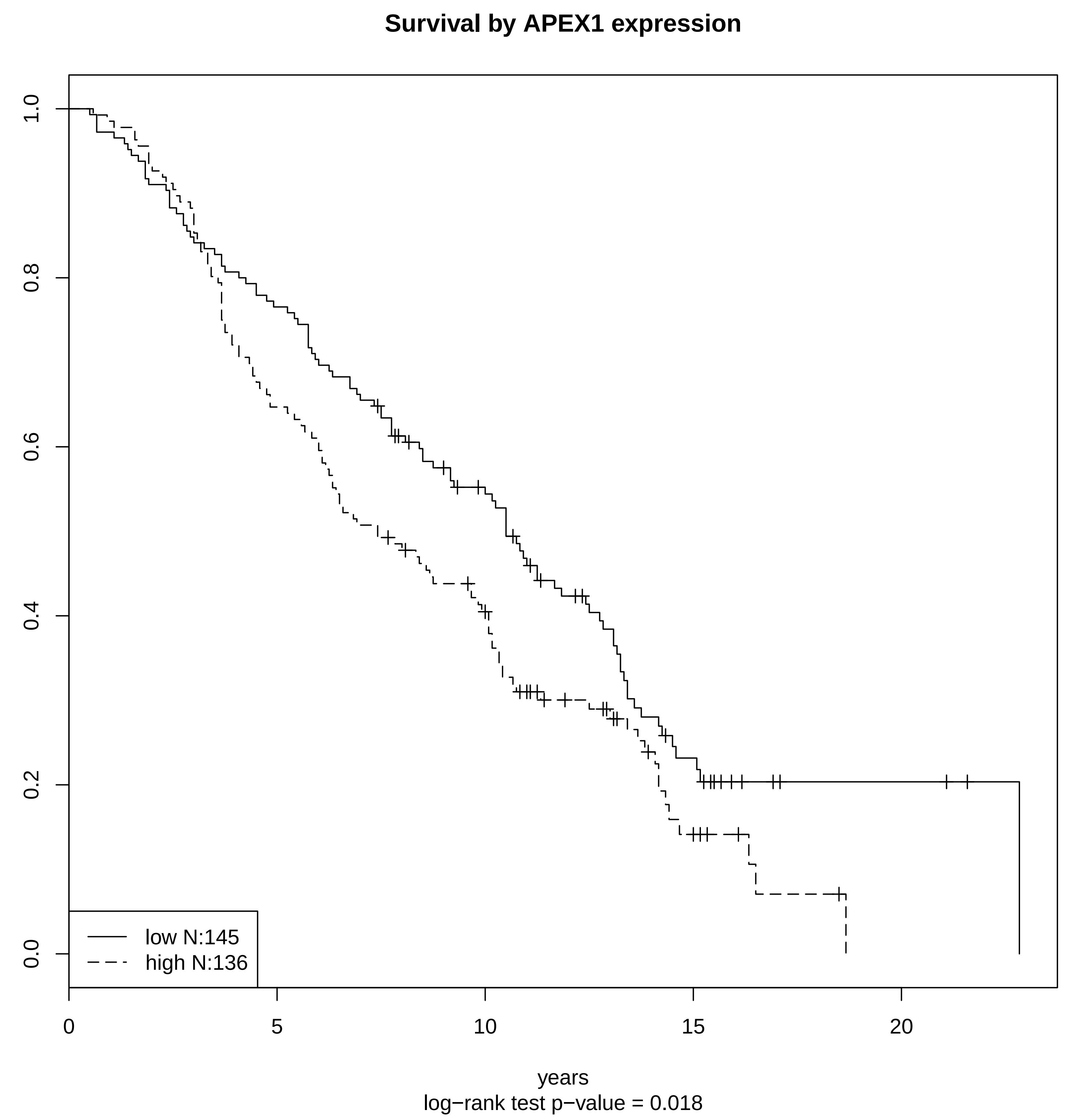

Survival by APEX1 expression
1.0
0.8
0.6
0.4
0.2
0.0
0
5
10
15
20
low N:145
high N:136
years
log−rank test p−value = 0.018

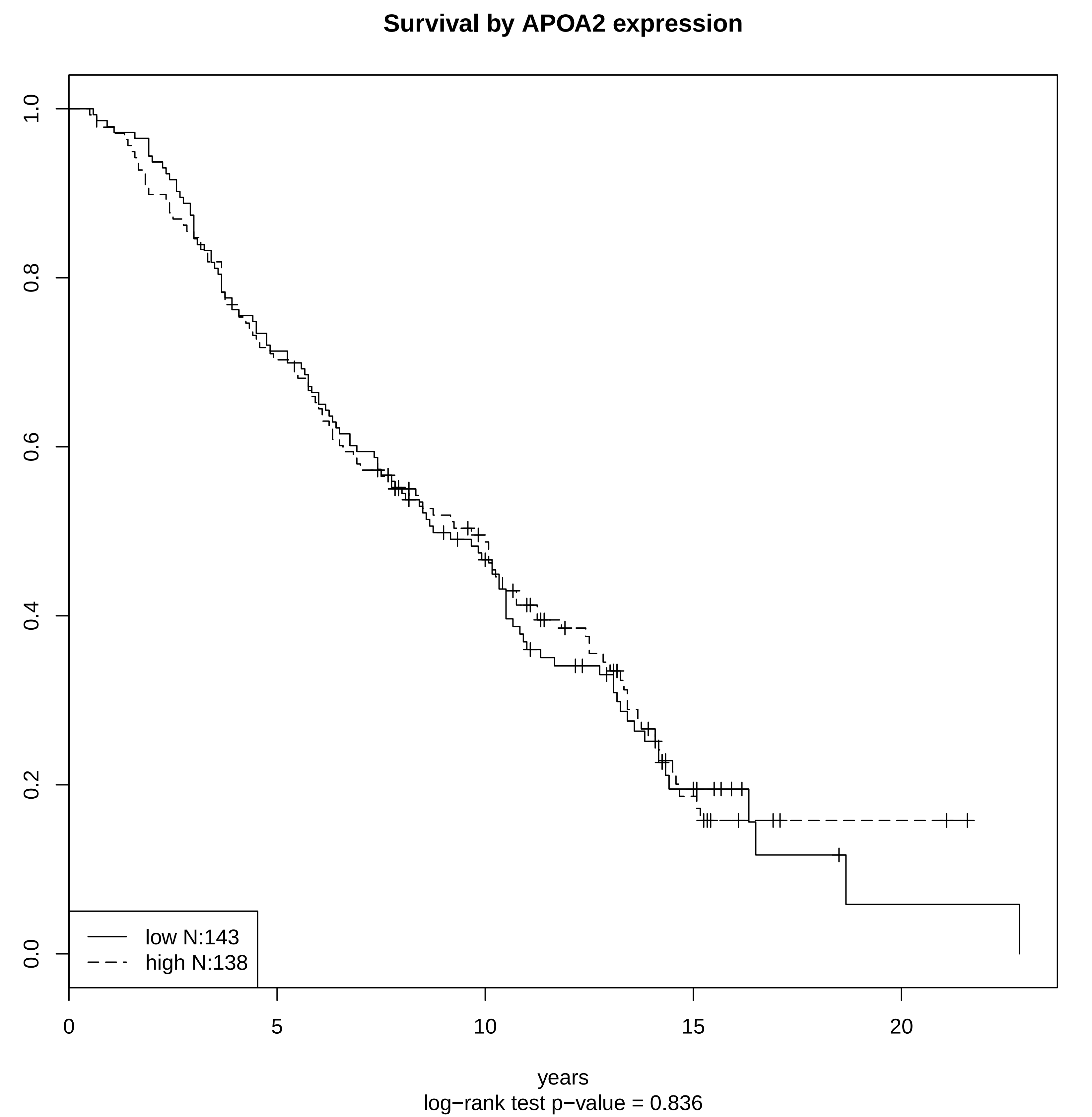
Survival by APOA2 expression
1.0
0.8
0.6
0.4
0.2
0.0
0
5
10
15
20
low N:143
high N:138
years
log−rank test p−value = 0.836

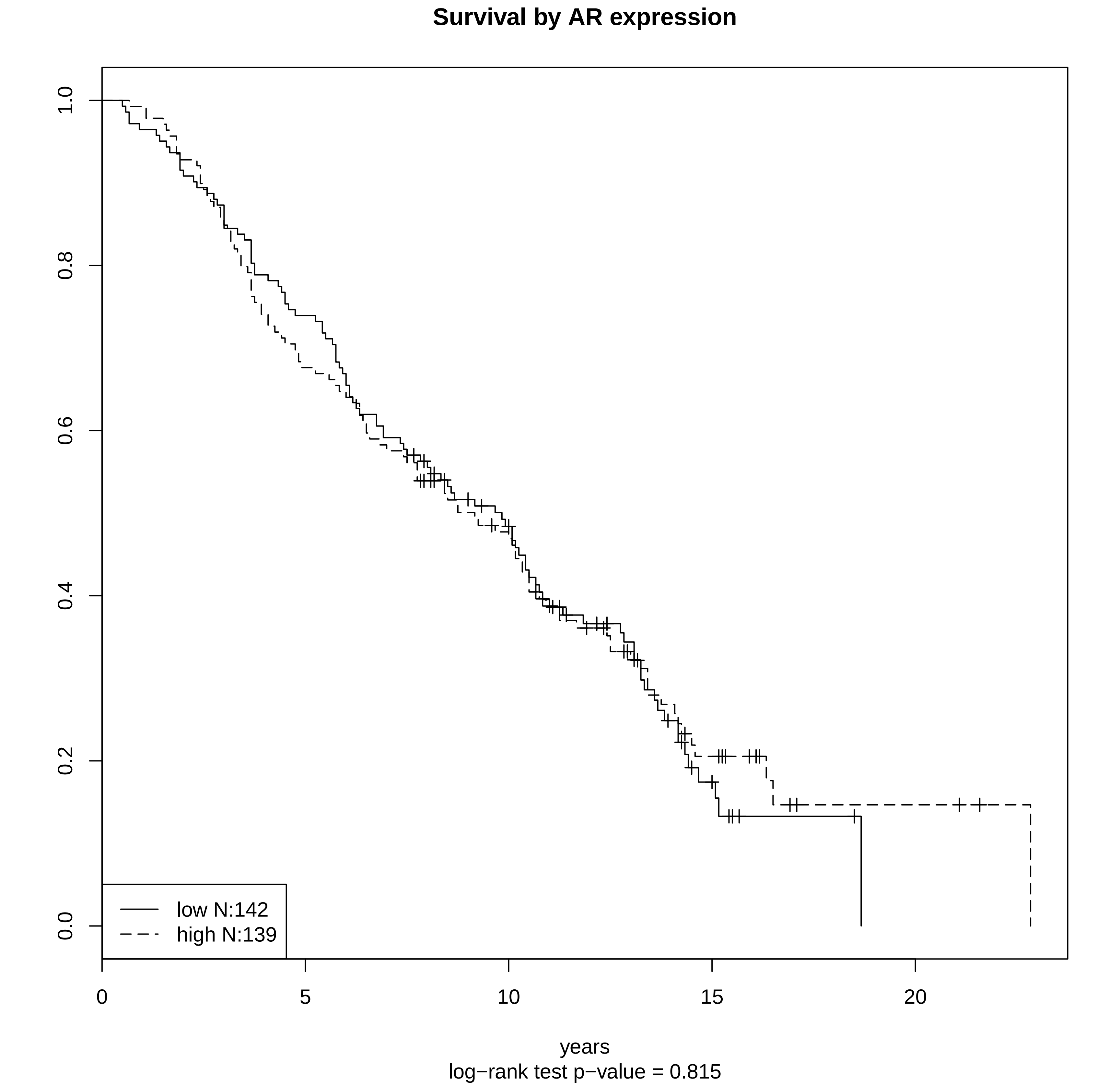
Survival by AR expression
1.0
0.8
0.6
0.4
0.2
0.0
0
5
10
15
20
years
low N:142
high N:139
log−rank test p−value = 0.815

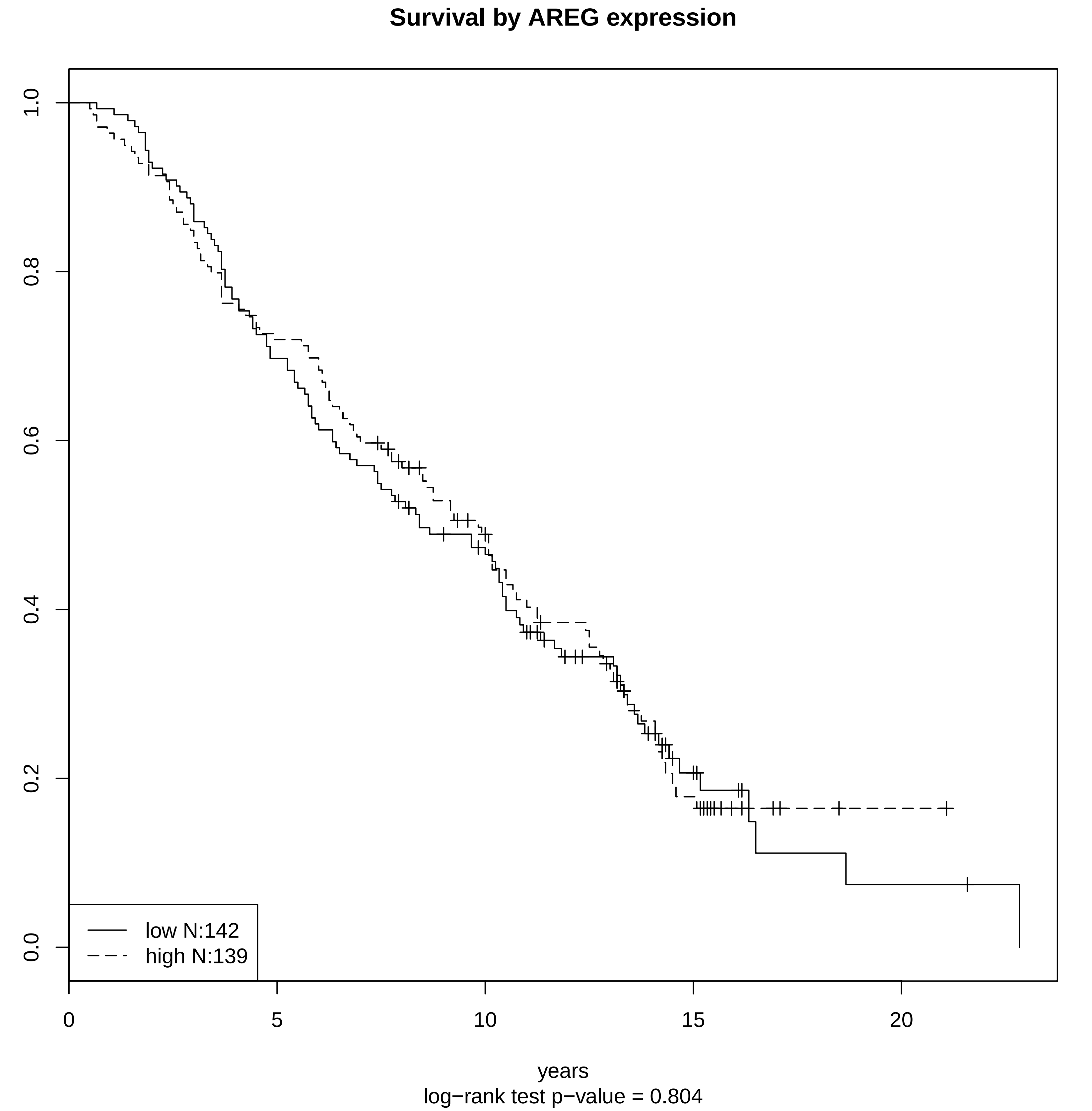

Survival by AREG expression
1.0
0.8
0.6
0.4
0.2
0.0
0
5
10
15
20
low N:142
high N:139
years
log−rank test p−value = 0.804

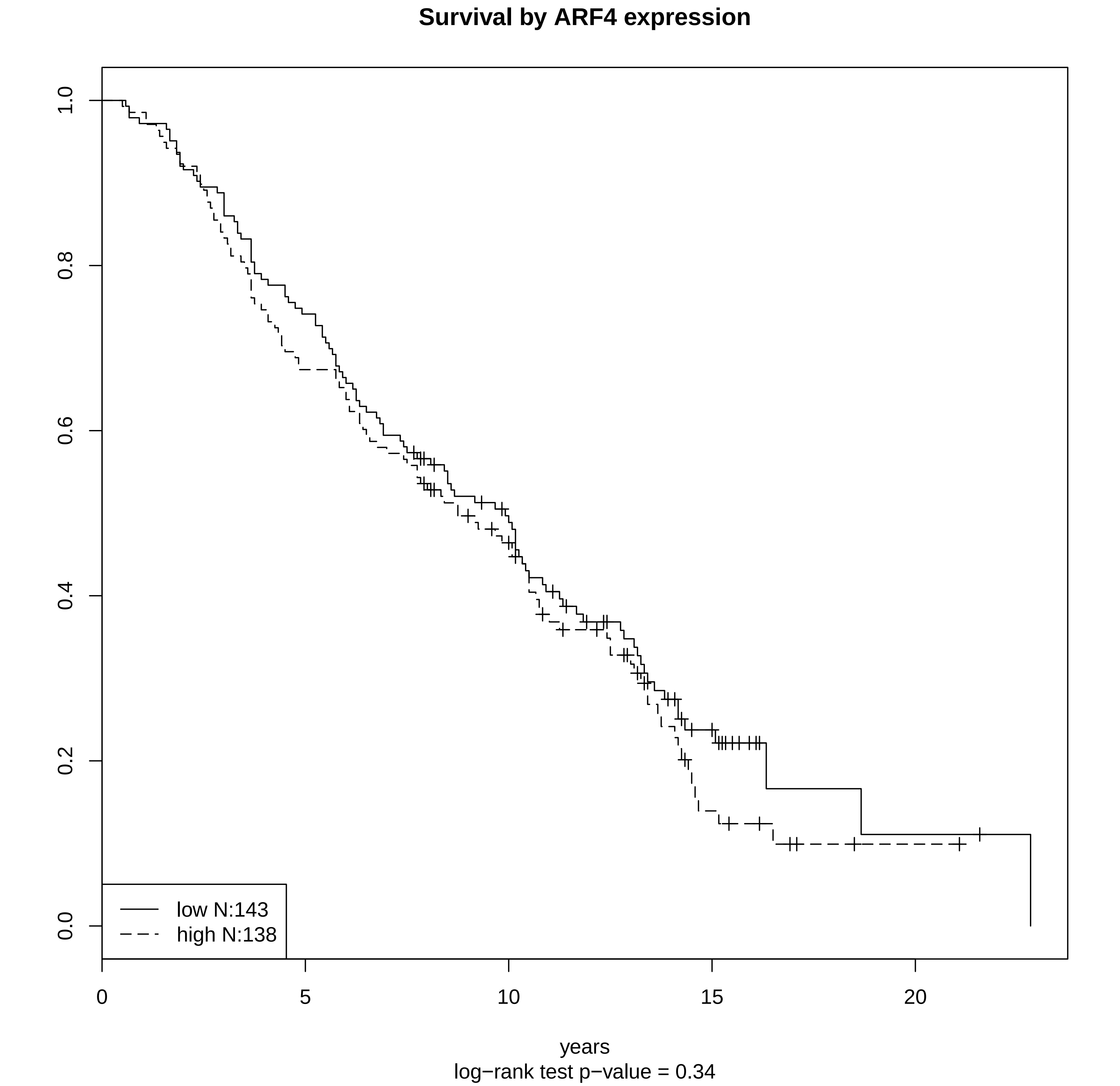
Survival by ARF4 expression
1.0
0.8
0.6
0.4
0.2
0.0
0
5
10
15
20
years
low N:143
high N:138
log−rank test p−value = 0.34

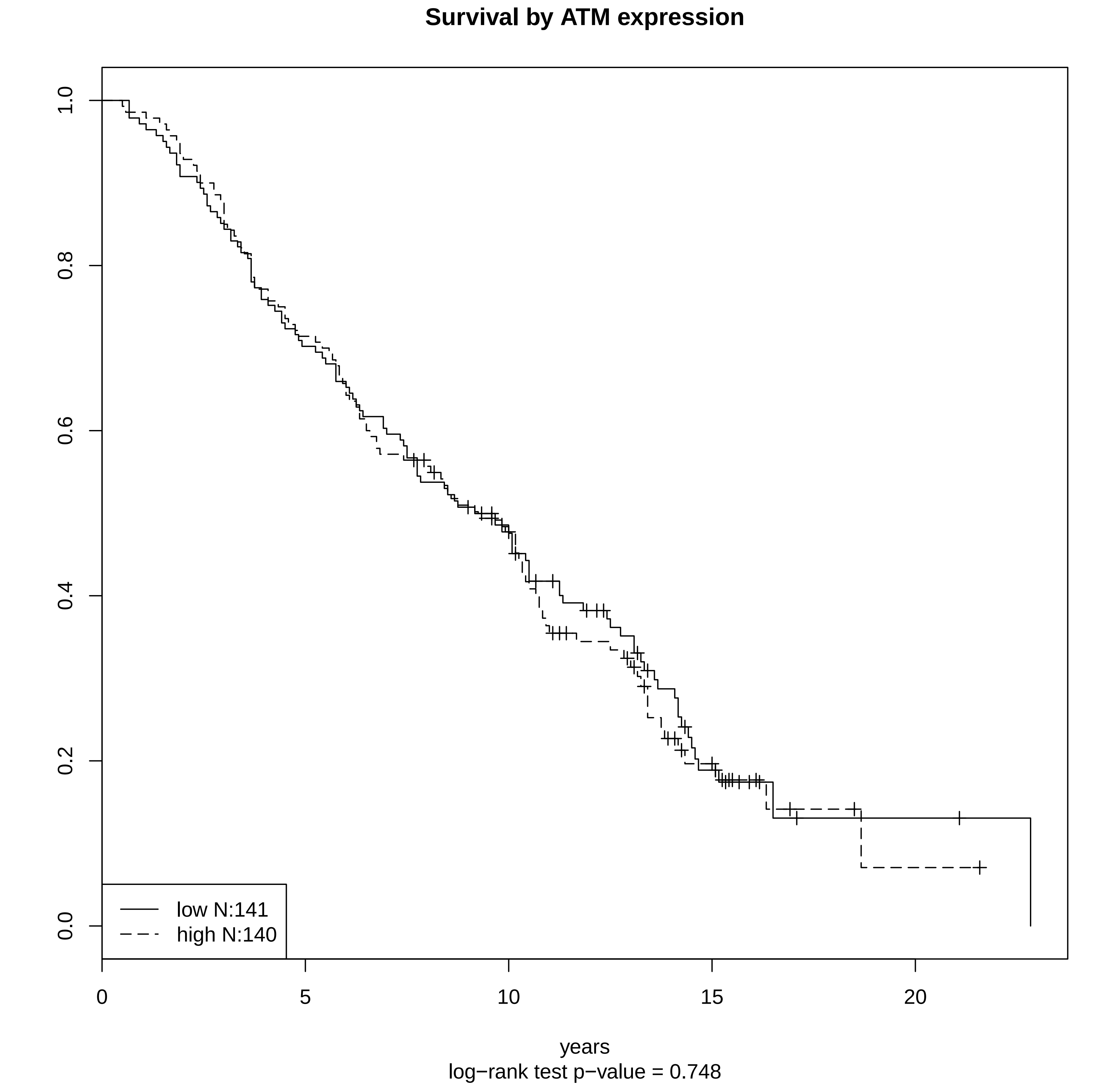

Survival by ATM expression
1.0
0.8
0.6
0.4
0.2
0.0
0
5
10
15
20
years
log−rank test p−value = 0.748
low N:141
high N:140

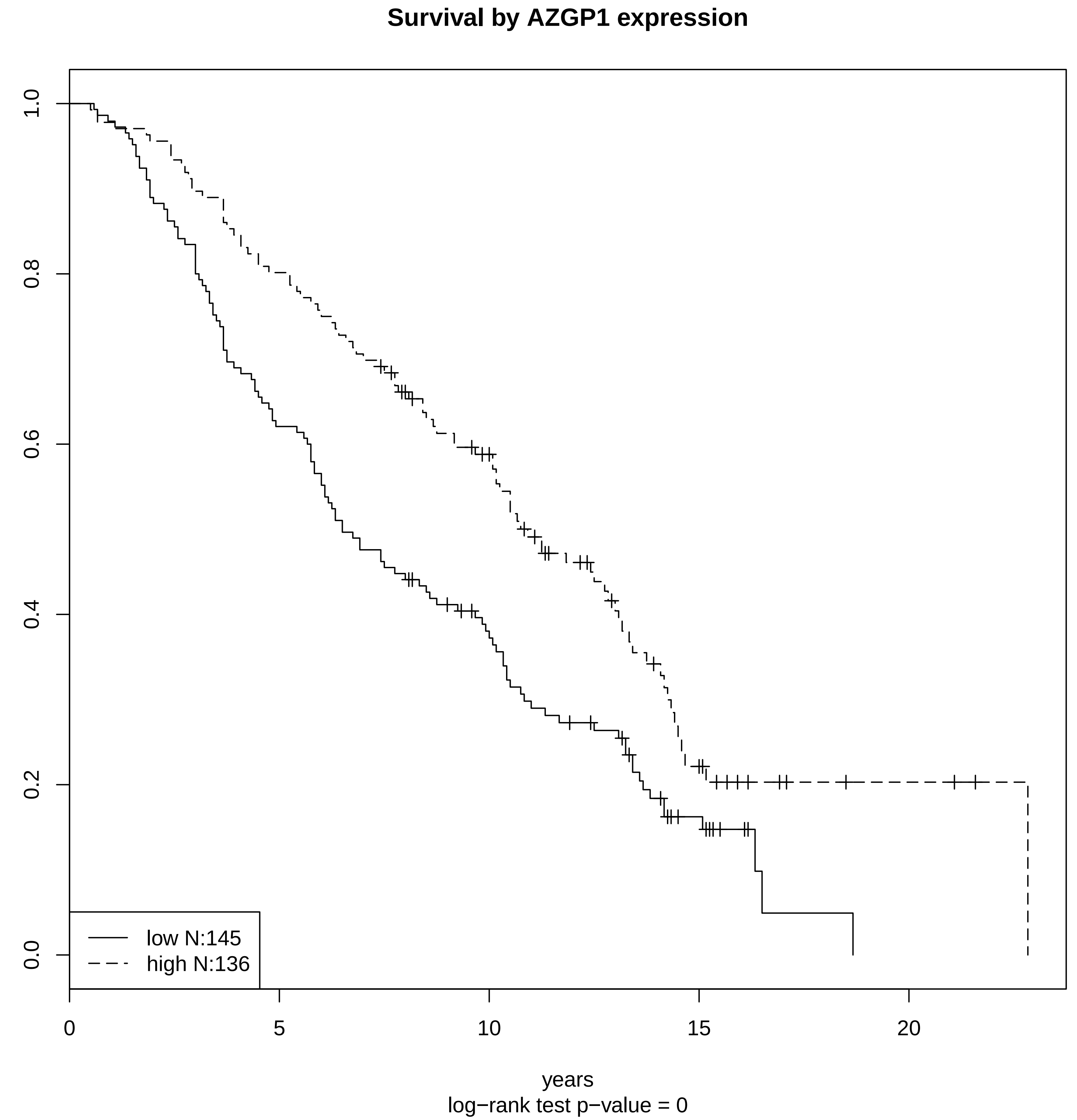

Survival by AZGP1 expression
1.0
0.8
0.6
0.4
0.2
0.0
0
5
10
15
20
low N:145
high N:136
years
log−rank test p−value = 0

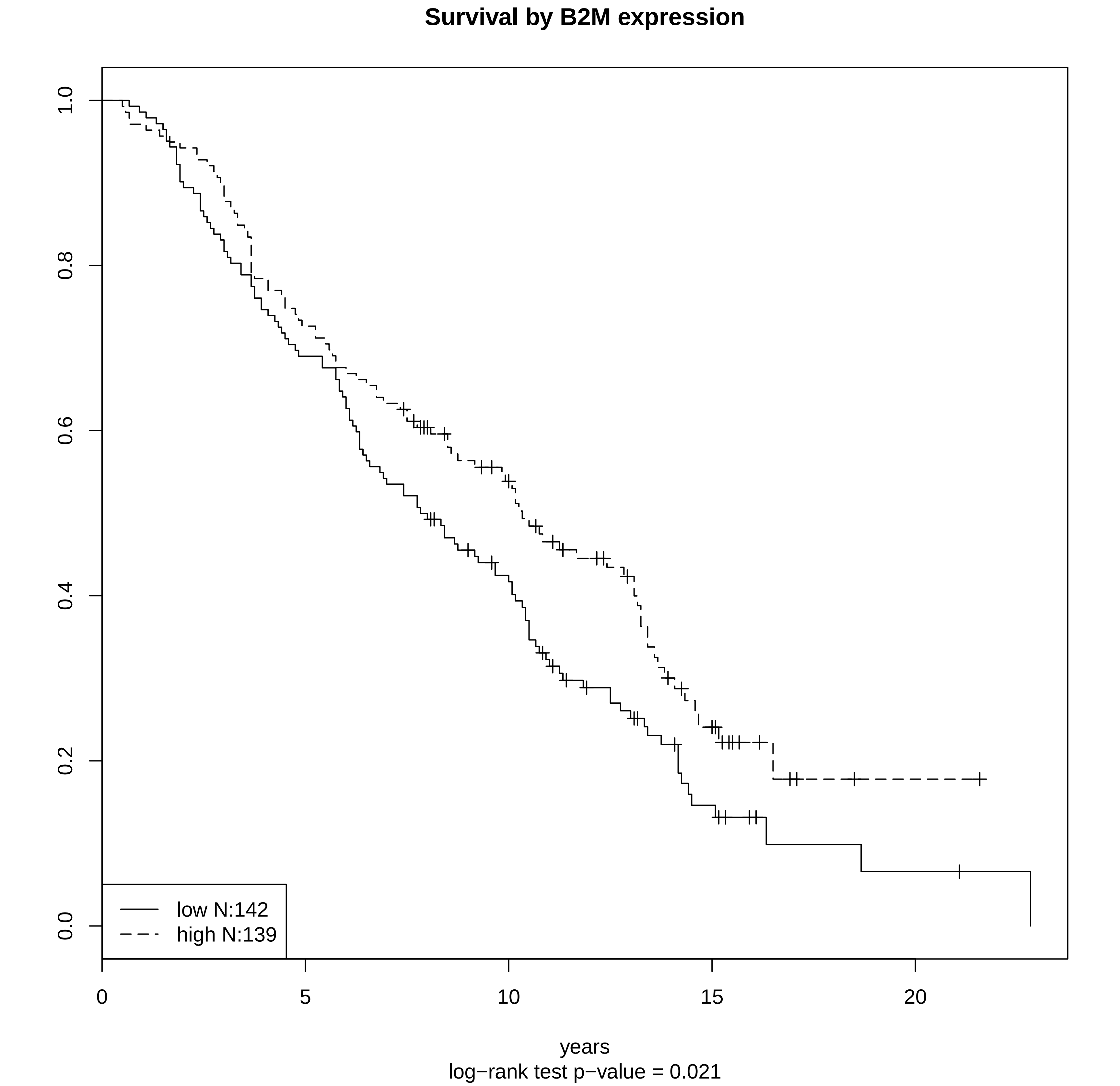
Survival by B2M expression
1.0
0.8
0.6
0.4
0.2
0.0
0
5
10
15
20
low N:142
high N:139
years
log−rank test p−value = 0.021

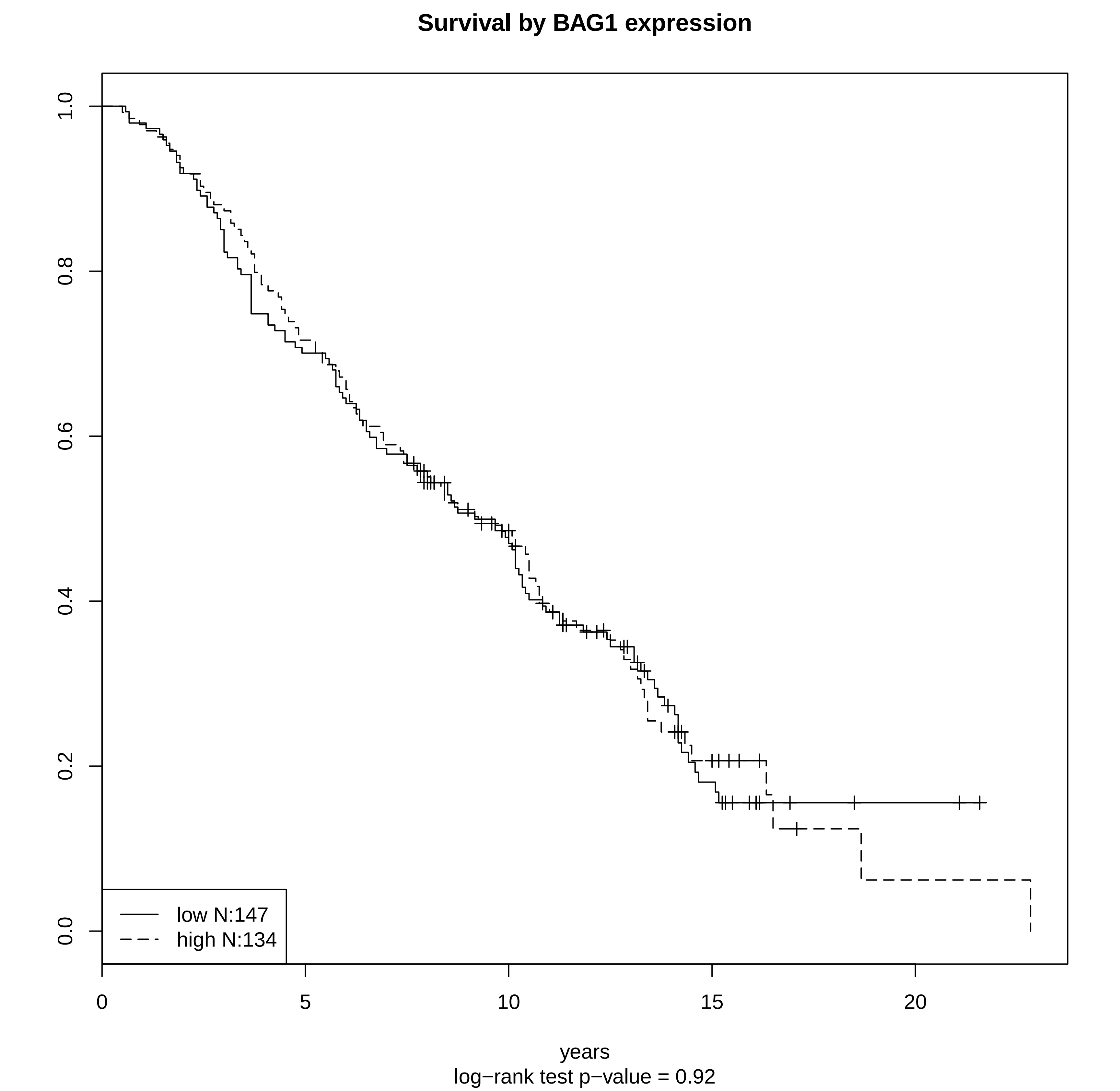
Survival by BAG1 expression
1.0
0.8
0.6
0.4
0.2
0.0
0
5
10
15
20
years
log−rank test p−value = 0.92
low N:147
high N:134

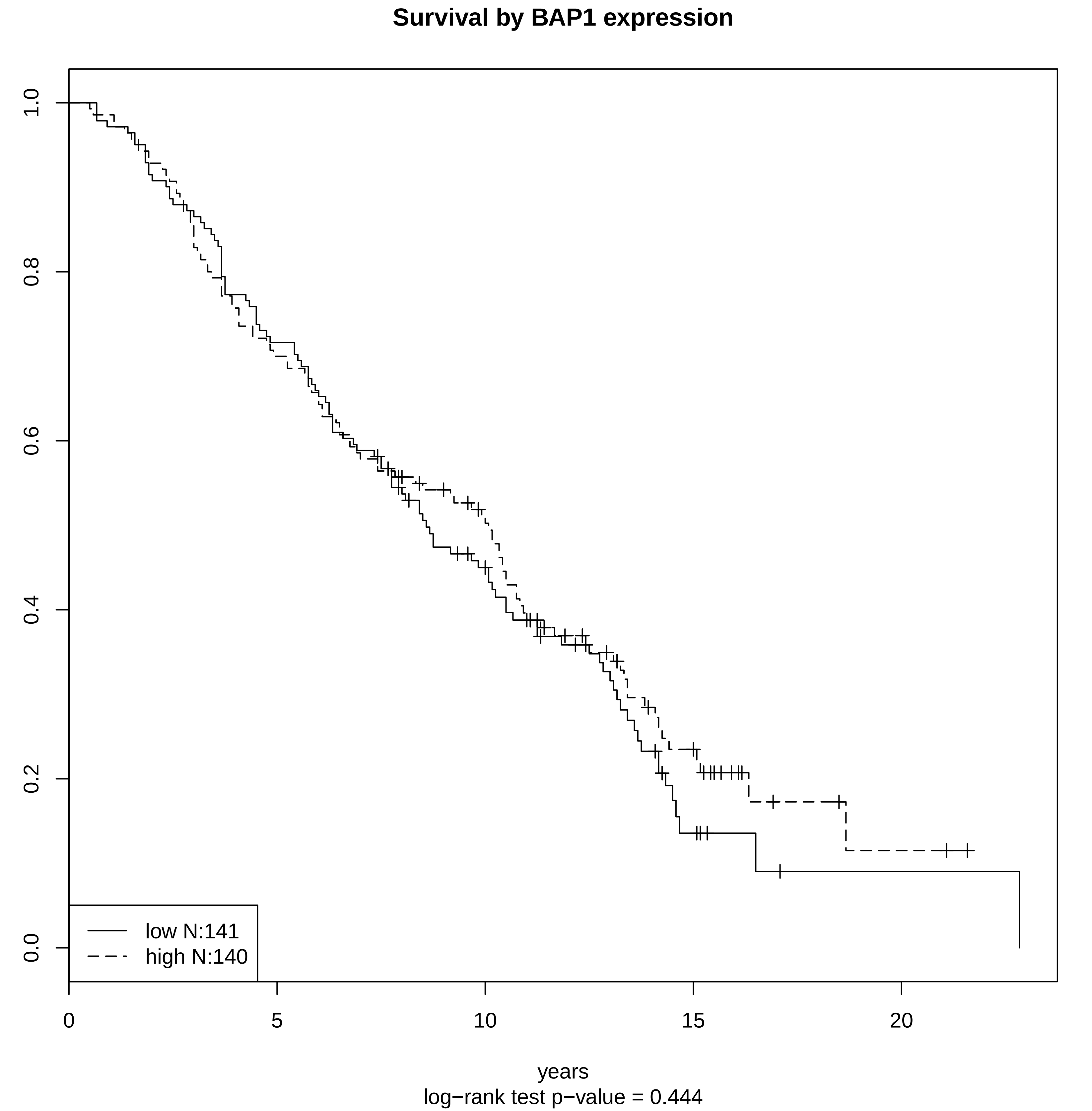
Survival by BAP1 expression
1.0
0.8
0.6
0.4
0.2
0.0
0
5
10
15
20
low N:141
high N:140
years
log−rank test p−value = 0.444

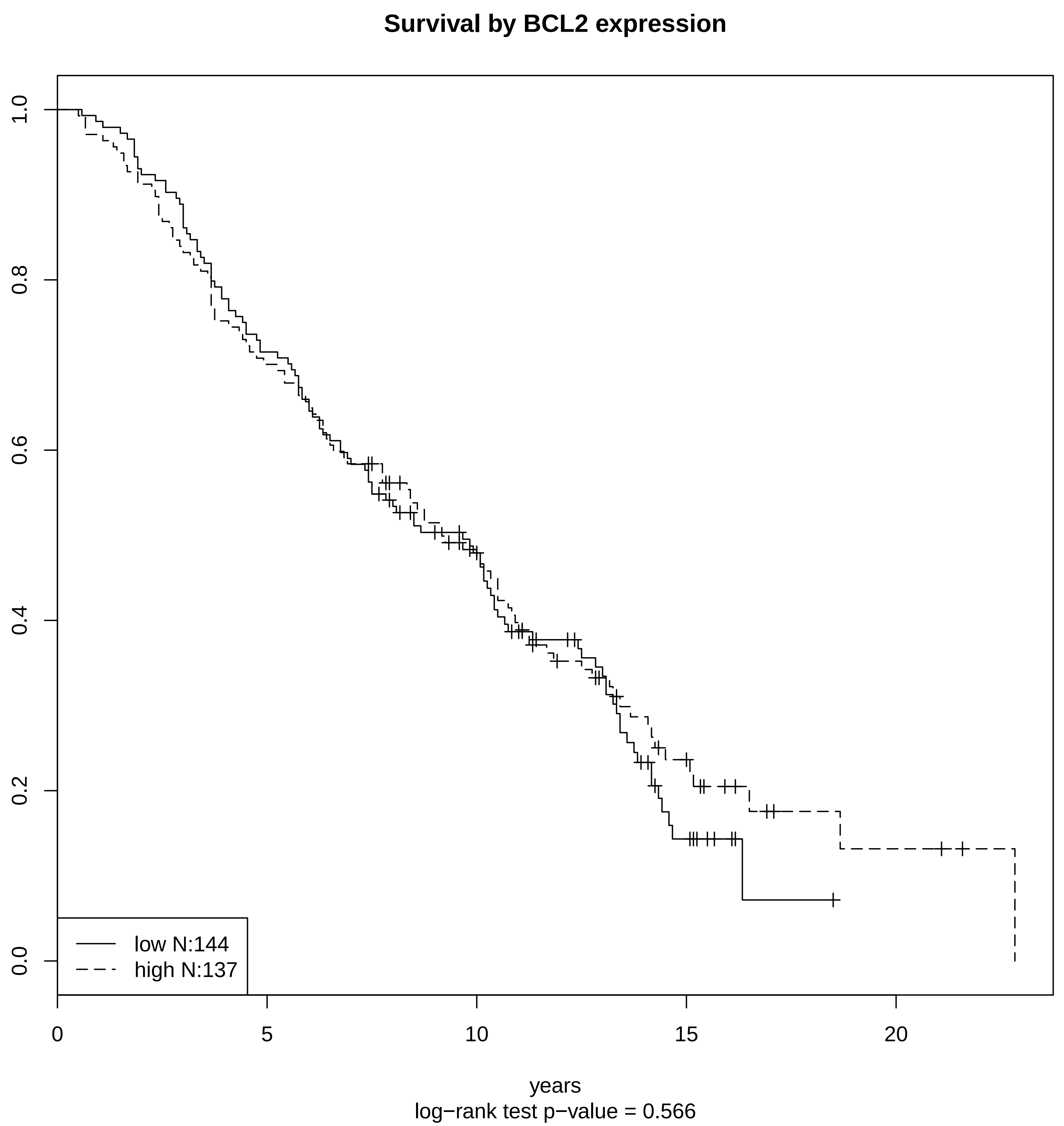

Survival by BCL2 expression
1.0
0.8
0.6
0.4
0.2
0.0
0
5
10
15
20
low N:144
high N:137
years
log−rank test p−value = 0.566

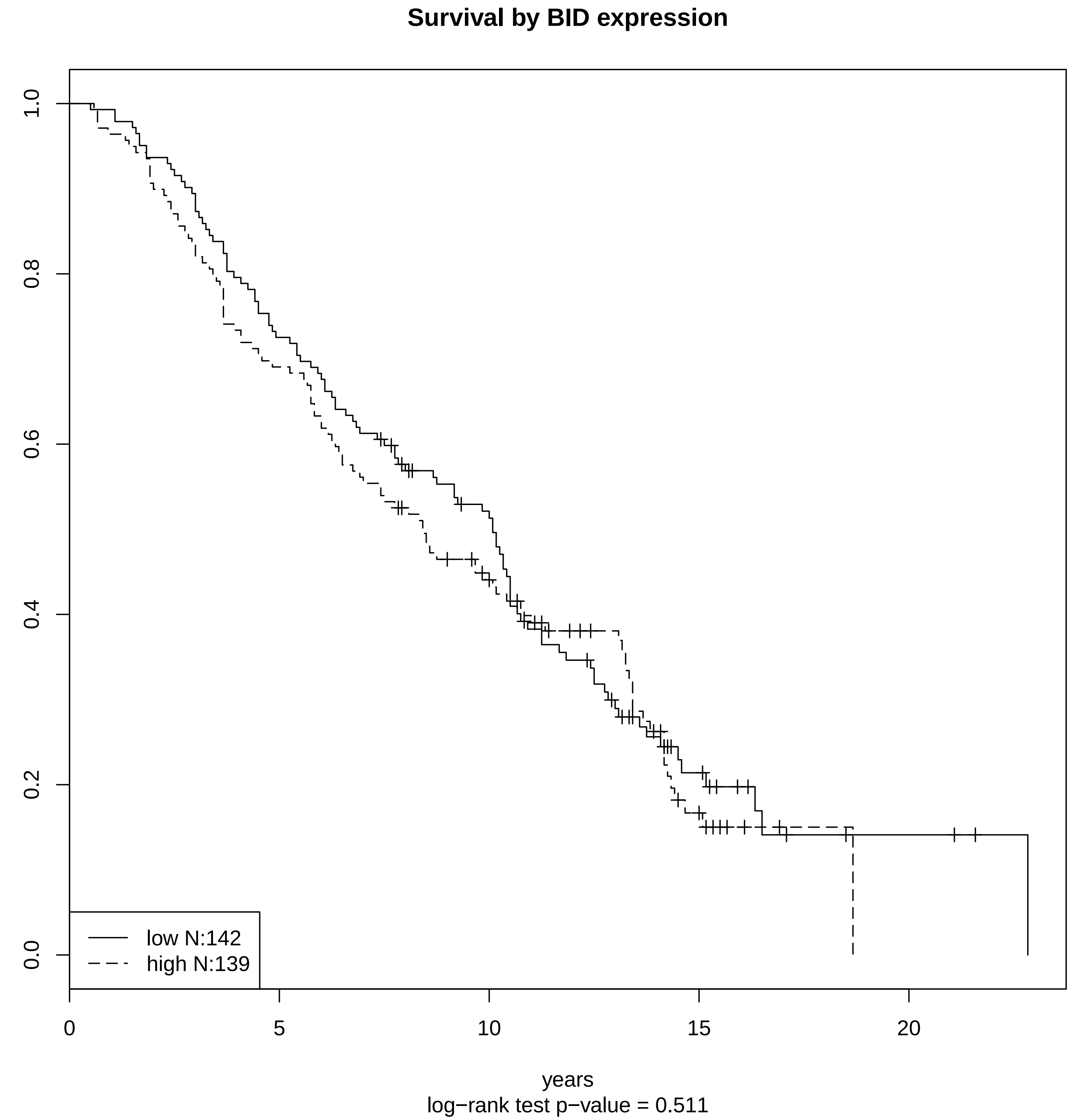
Survival by BID expression
1.0
0.8
0.6
0.4
0.2
0.0
0
5
10
15
20
low N:142
high N:139
years
log−rank test p−value = 0.511

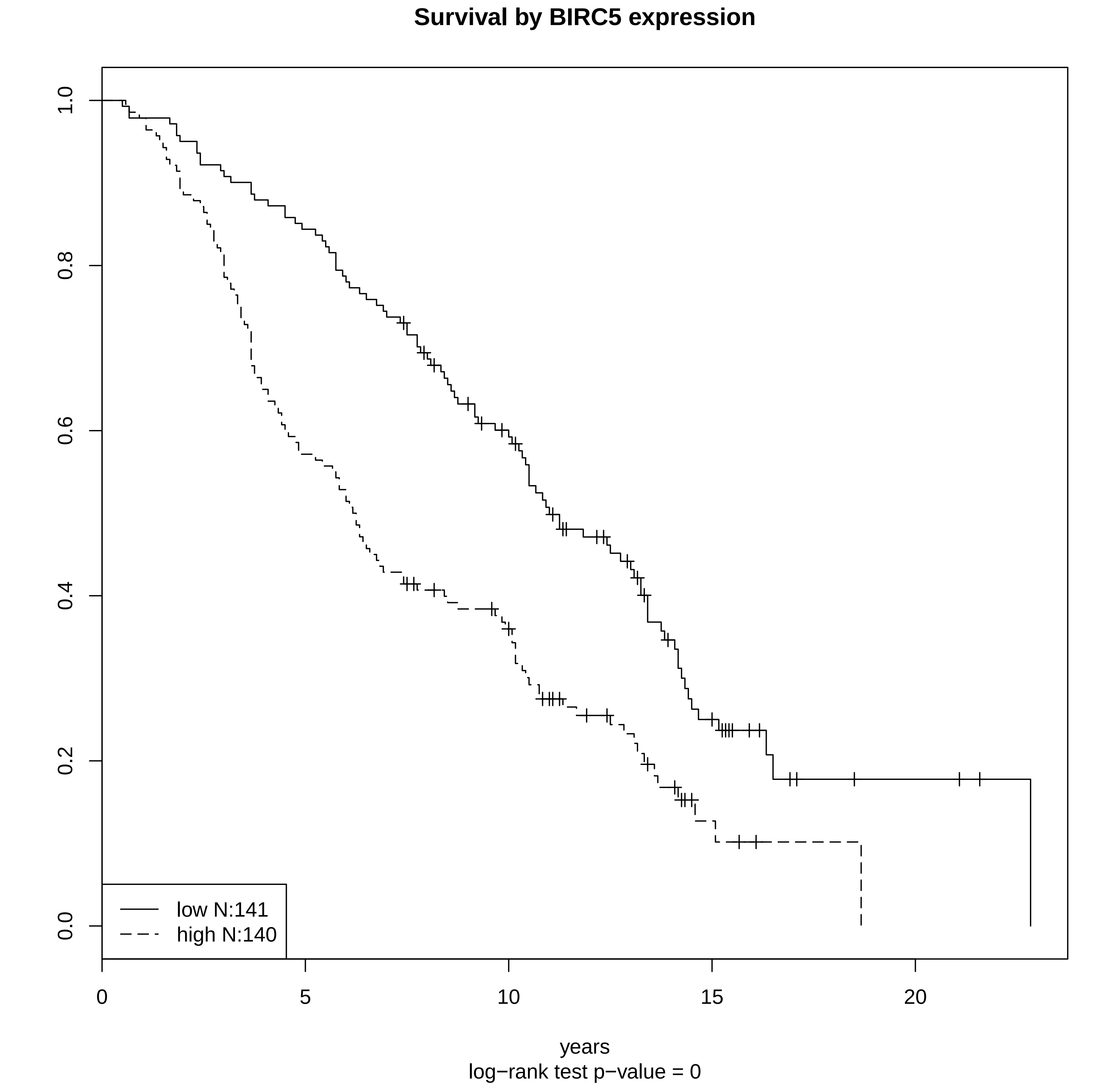

Survival by BIRC5 expression
1.0
0.8
0.6
0.4
0.2
0.0
0
5
10
15
20
low N:141
high N:140
years
log−rank test p−value = 0

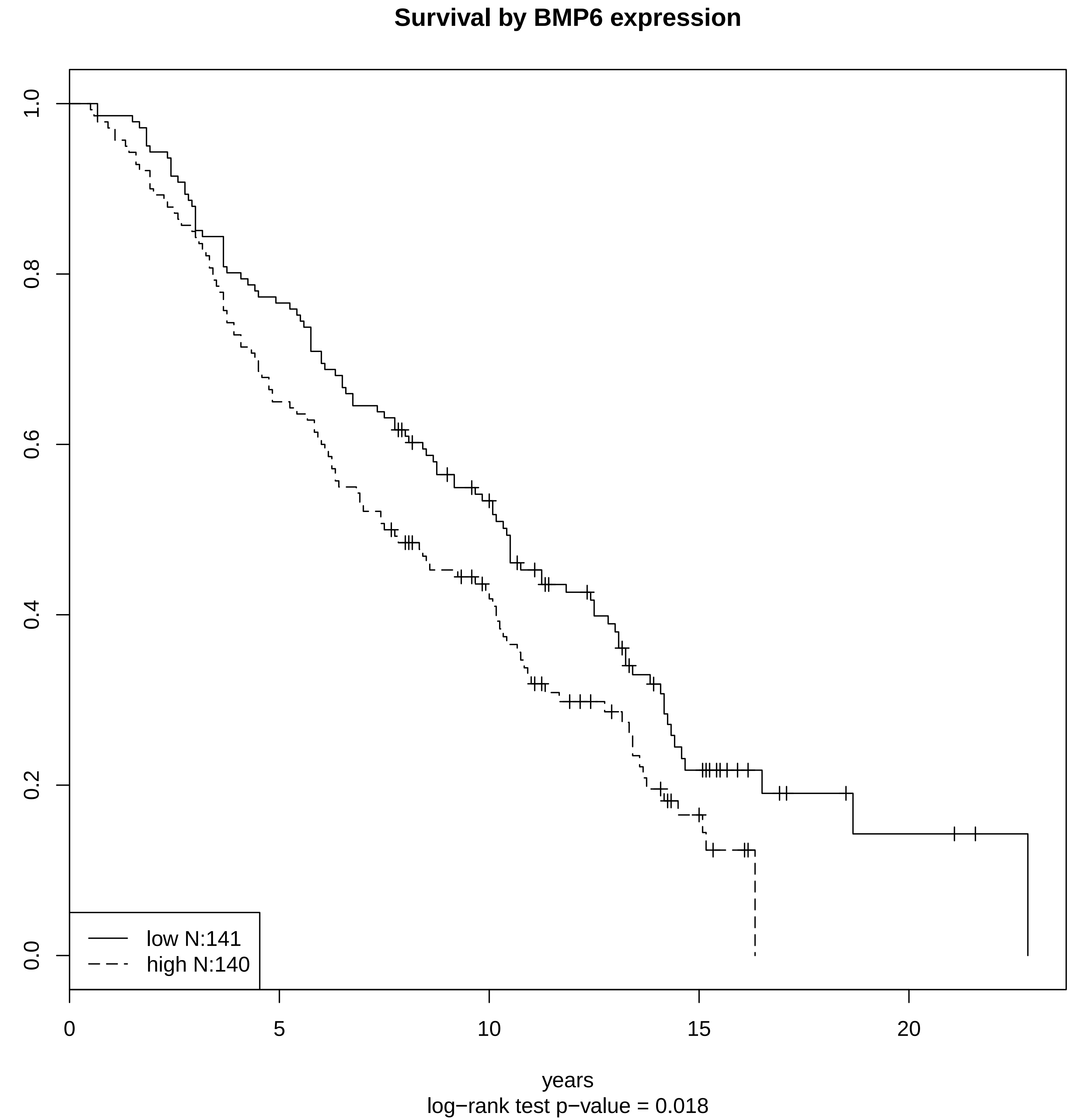
Survival by BMP6 expression
1.0
0.8
0.6
0.4
0.2
0.0
0
5
10
15
20
low N:141
high N:140
years
log−rank test p−value = 0.018

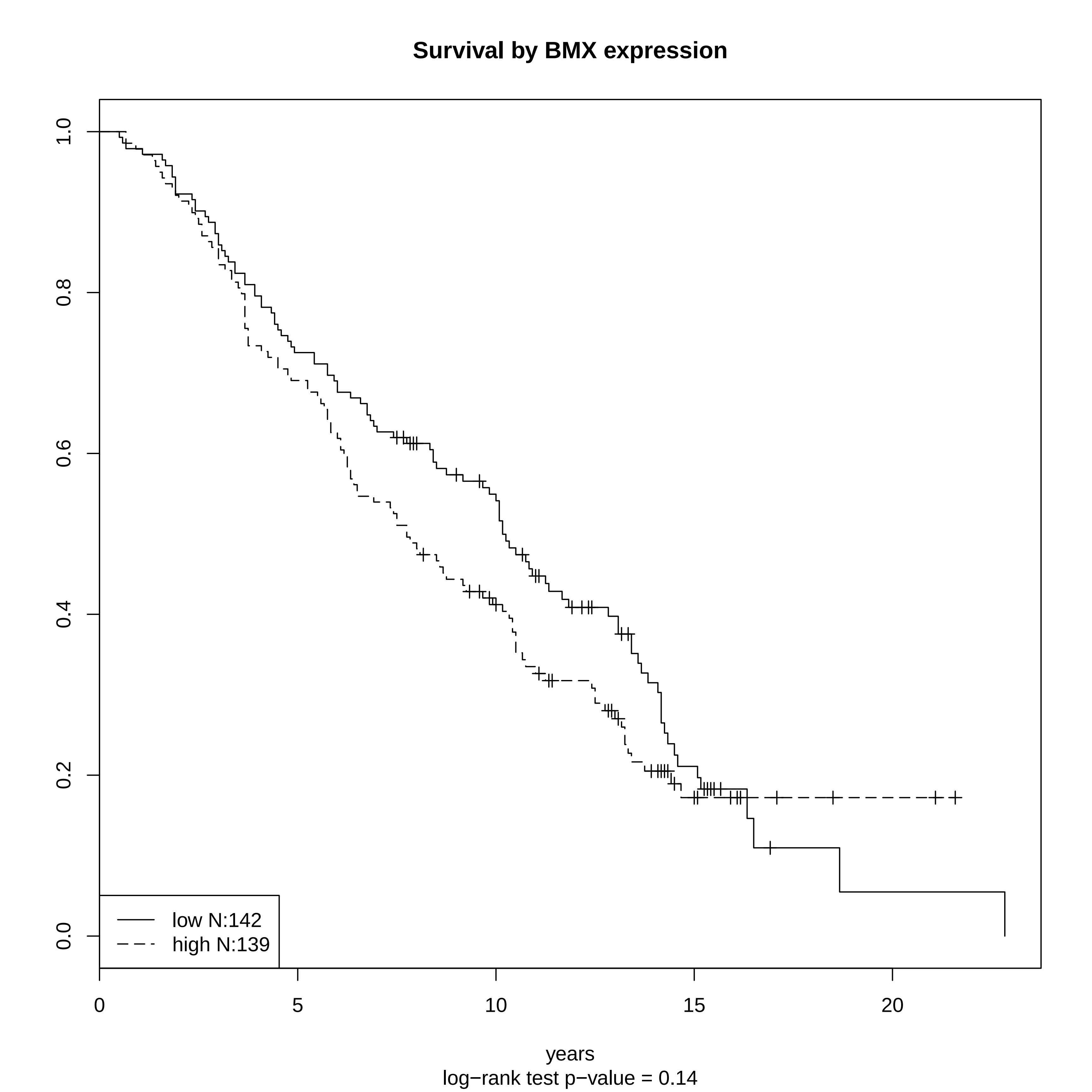

Survival by BMX expression
1.0
0.8
0.6
0.4
0.2
0.0
0
5
10
15
20
low N:142
high N:139
years
log−rank test p−value = 0.14

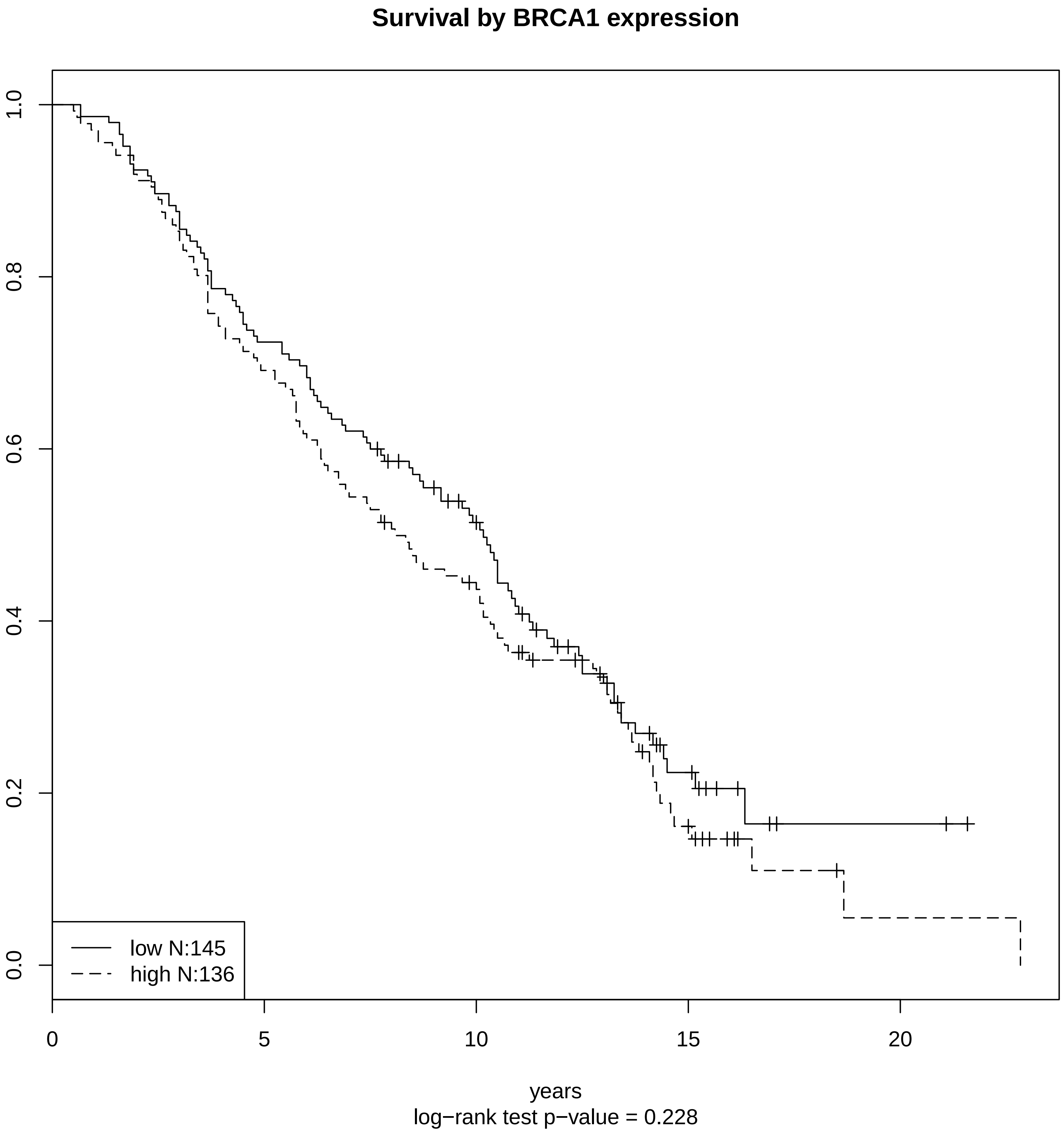

Survival by BRCA1 expression
1.0
0.8
0.6
0.4
0.2
0.0
0
5
10
15
20
low N:145
high N:136
years
log−rank test p−value = 0.228

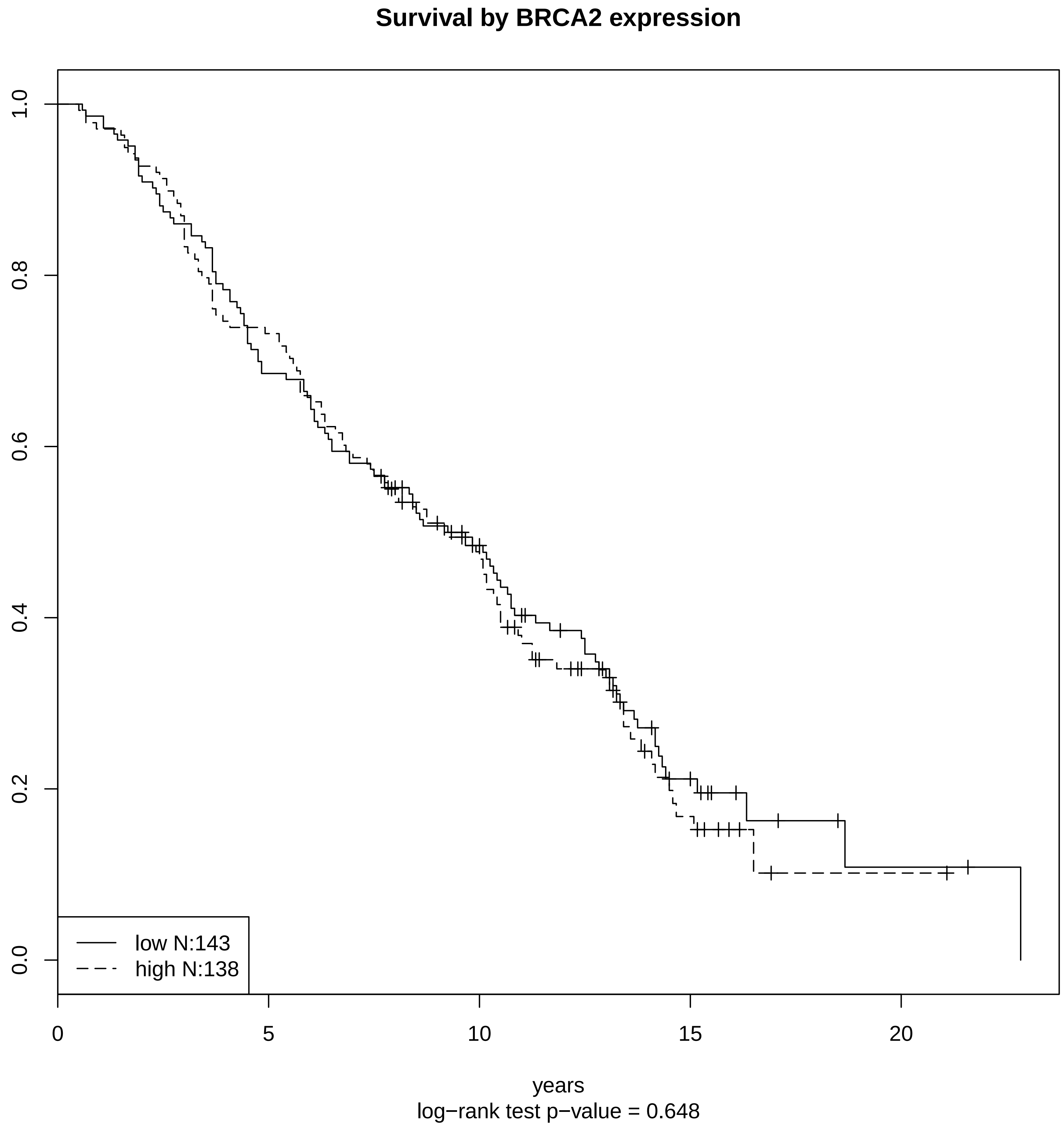
Survival by BRCA2 expression
1.0
0.8
0.6
0.4
0.2
0.0
0
5
10
15
20
low N:143
high N:138
years
log−rank test p−value = 0.648

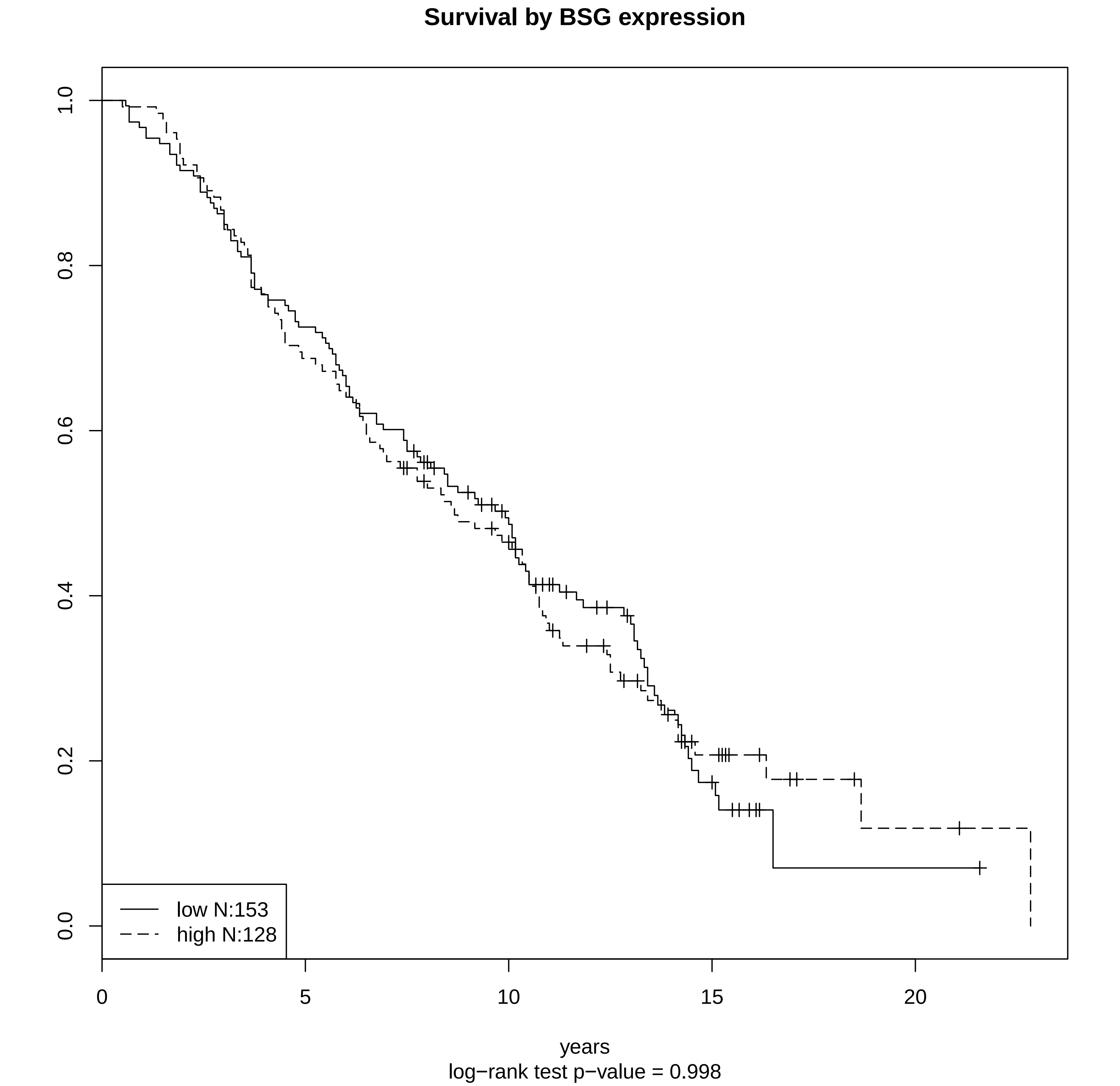
Survival by BSG expression
1.0
0.8
0.6
0.4
0.2
0.0
0
5
10
15
20
low N:153
high N:128
years
log−rank test p−value = 0.998

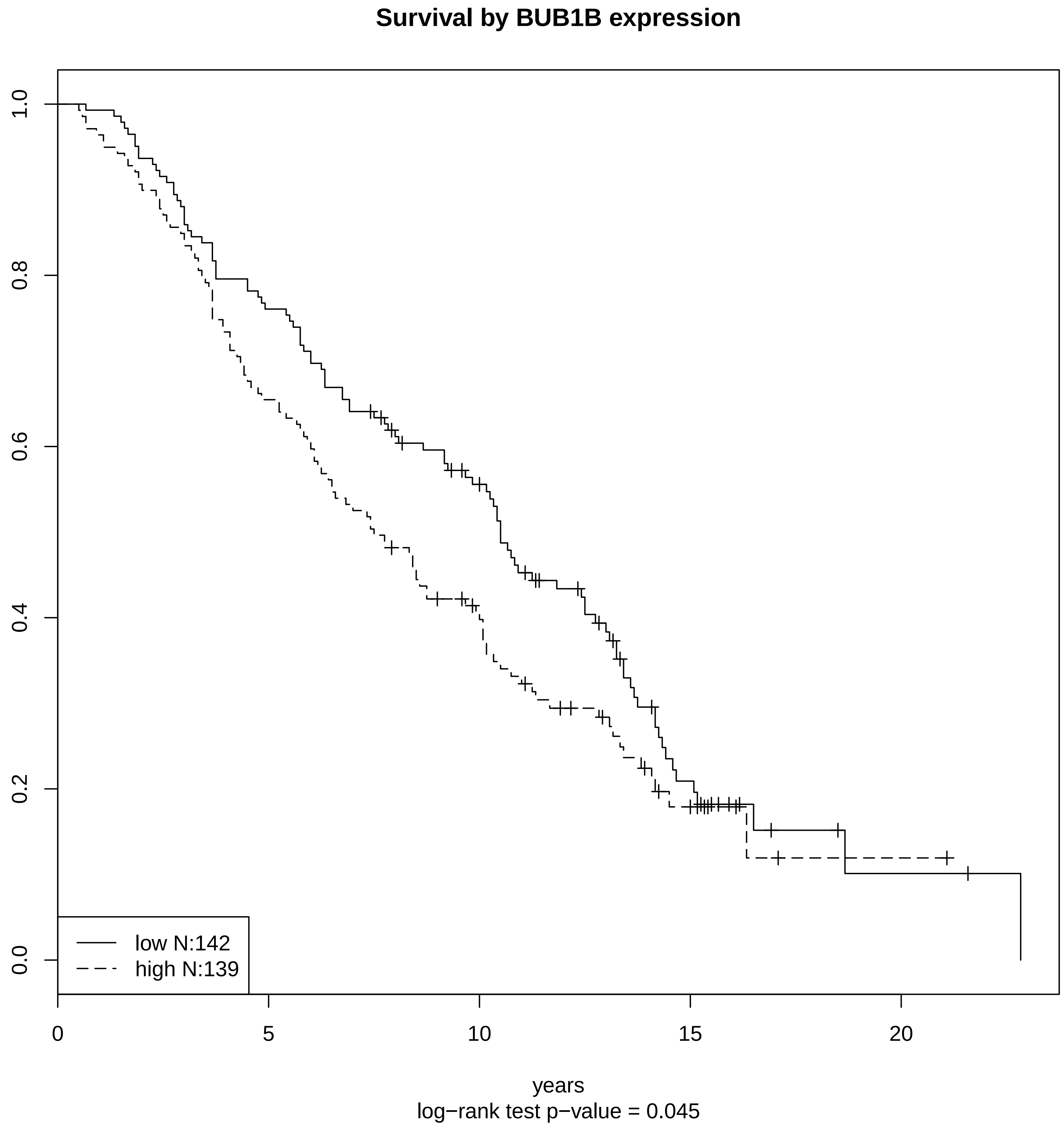

Survival by BUB1B expression
1.0
0.8
0.6
0.4
0.2
0.0
0
5
10
15
20
low N:142
high N:139
years
log−rank test p−value = 0.045

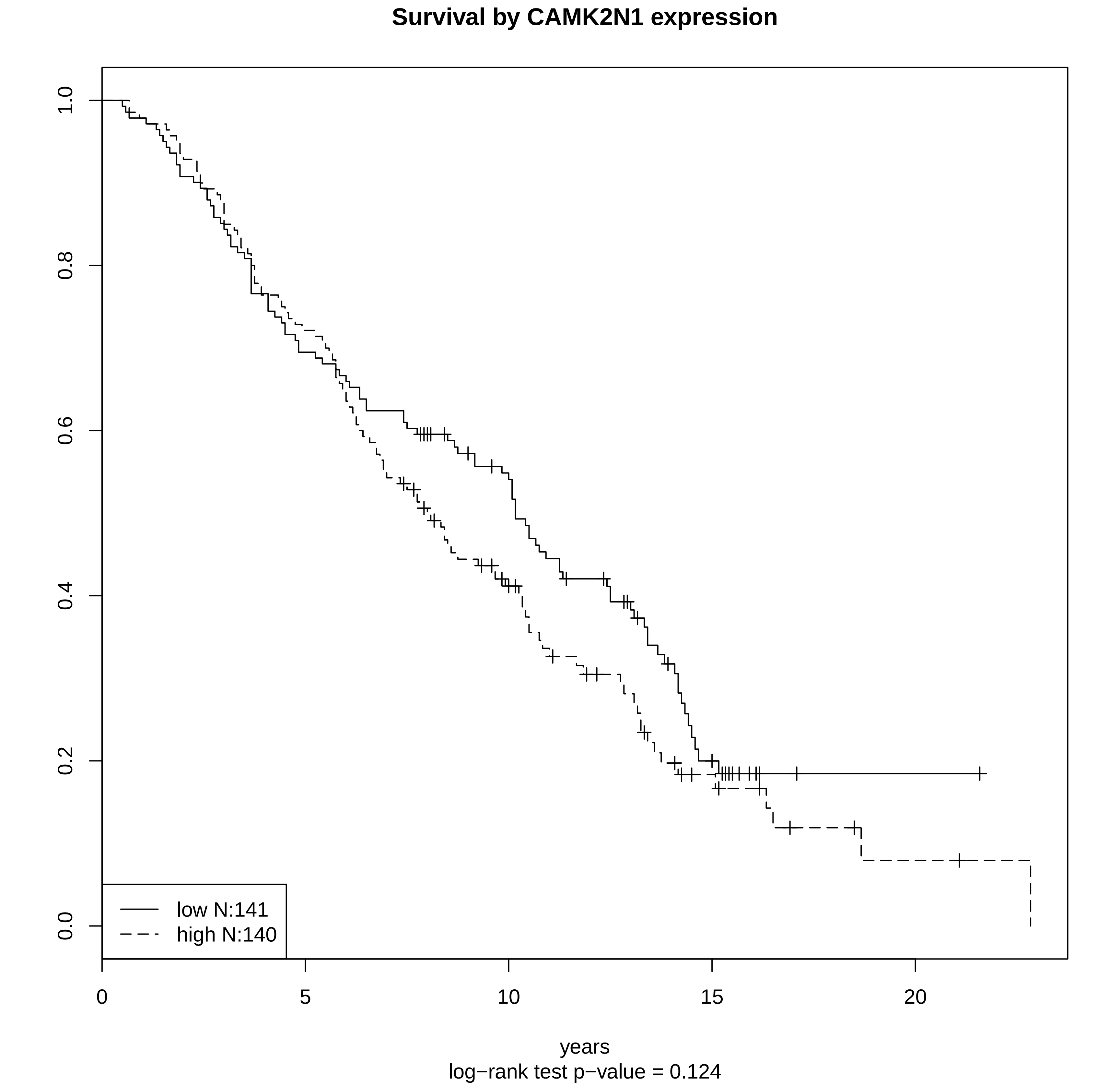

Survival by CAMK2N1 expression
1.0
0.8
0.6
0.4
0.2
0.0
0
5
10
15
20
low N:141
high N:140
years
log−rank test p−value = 0.124

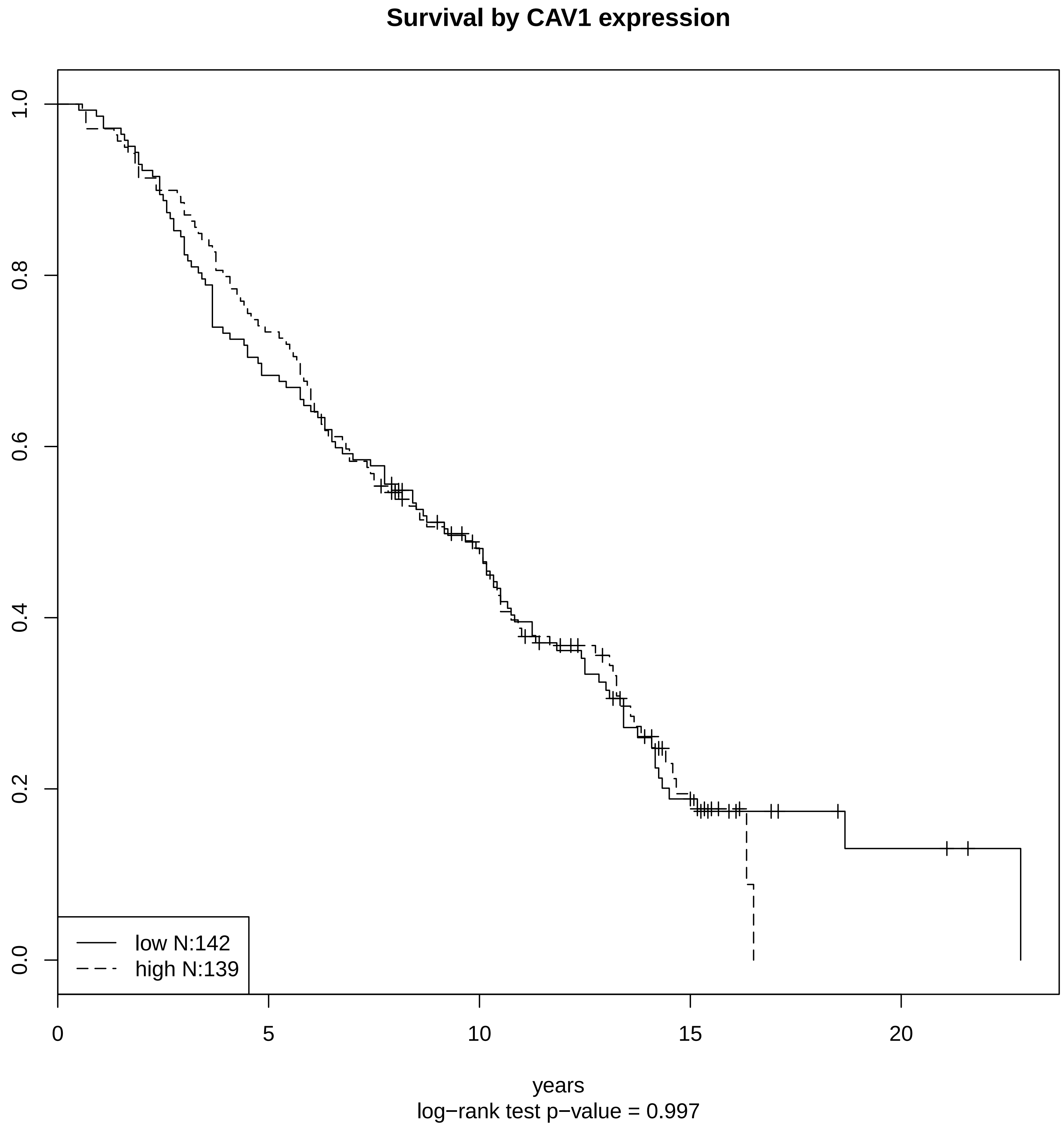
Survival by CAV1 expression
1.0
0.8
0.6
0.4
0.2
0.0
low N:142
high N:139
0
5
10
15
20
years
log−rank test p−value = 0.997

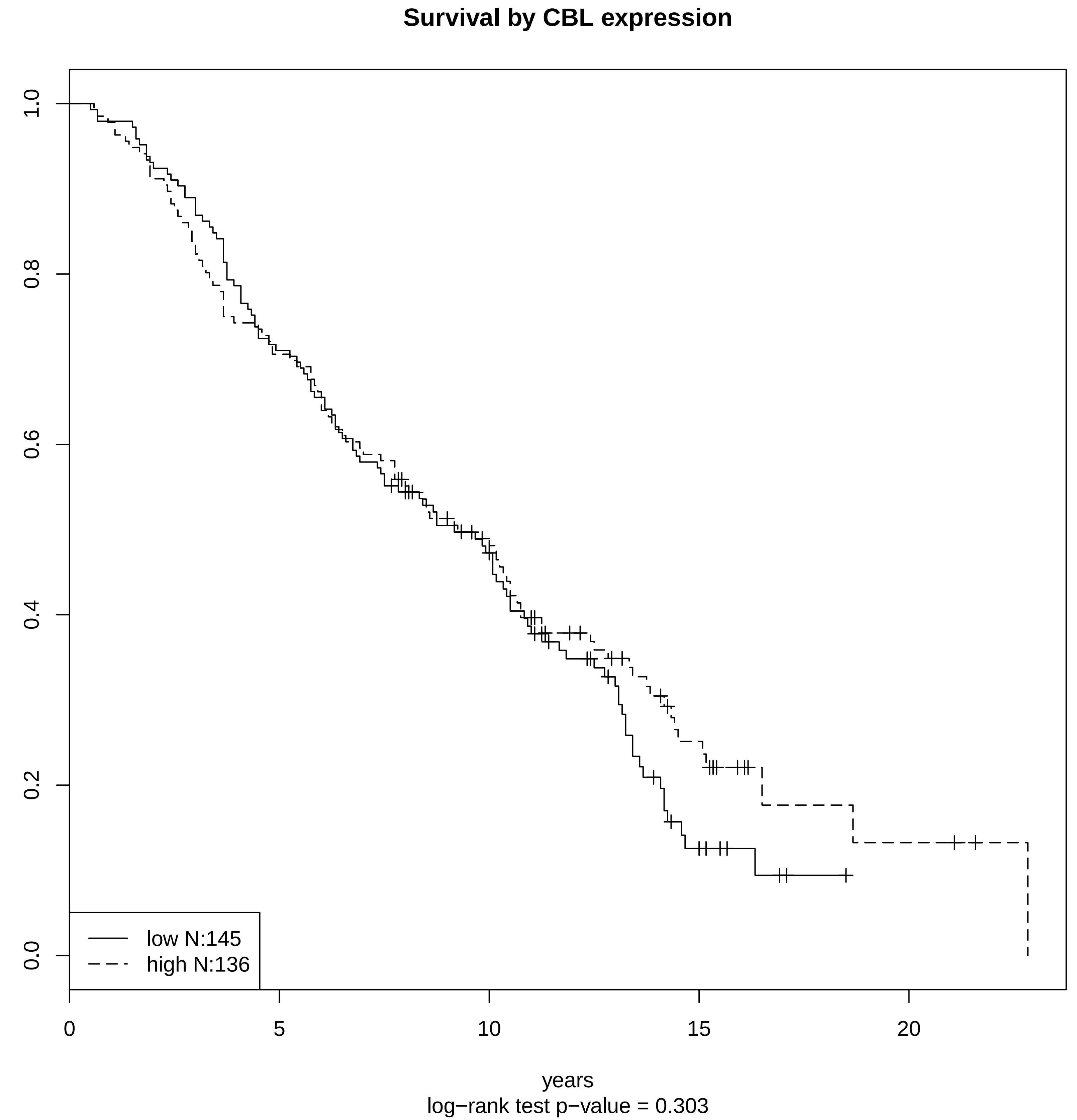
Survival by CBL expression
1.0
0.8
0.6
0.4
0.2
0.0
0
5
10
15
20
low N:145
high N:136
years
log−rank test p−value = 0.303

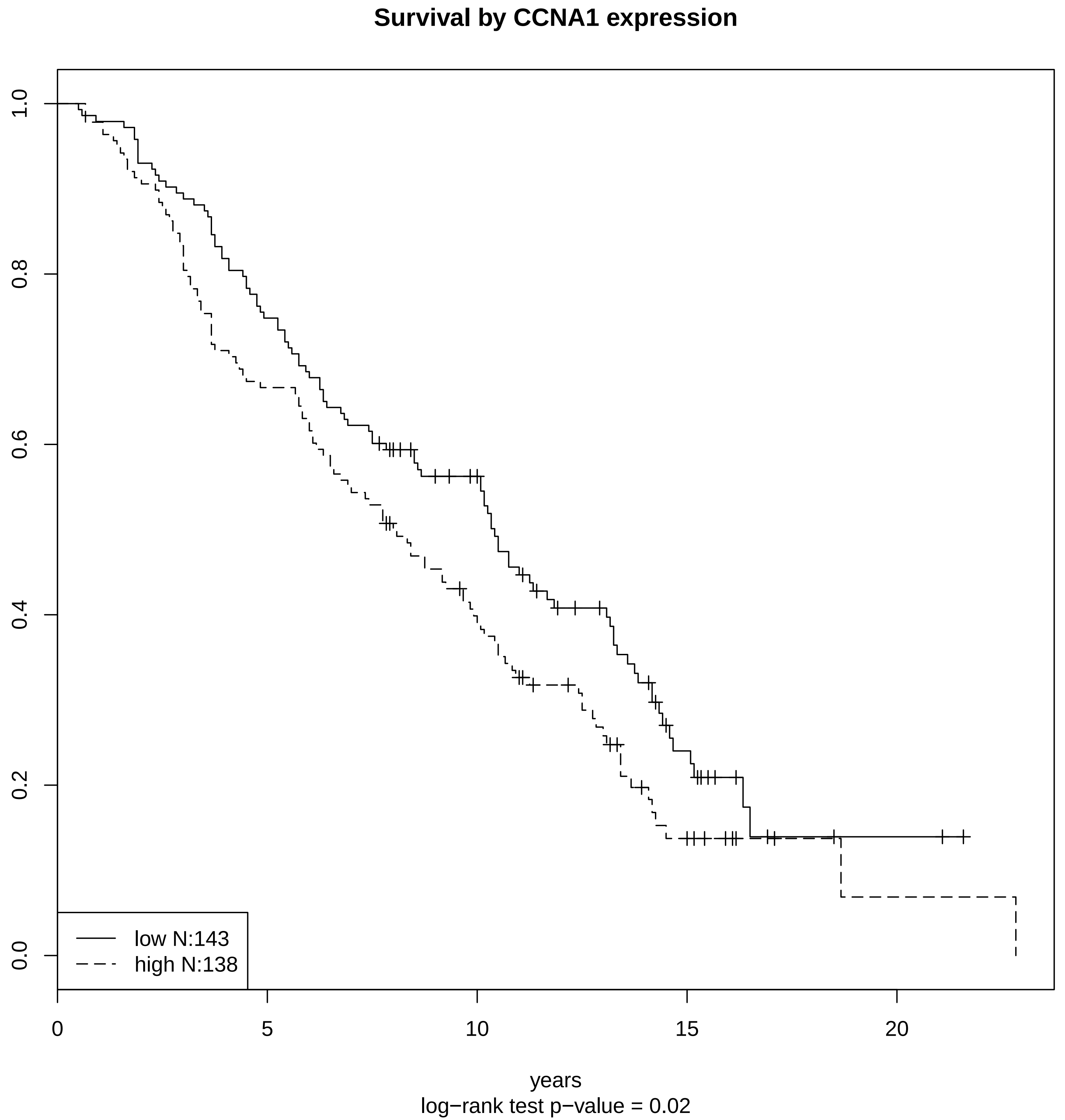

Survival by CCNA1 expression
1.0
0.8
0.6
0.4
0.2
0.0
0
5
10
15
20
low N:143
high N:138
years
log−rank test p−value = 0.02

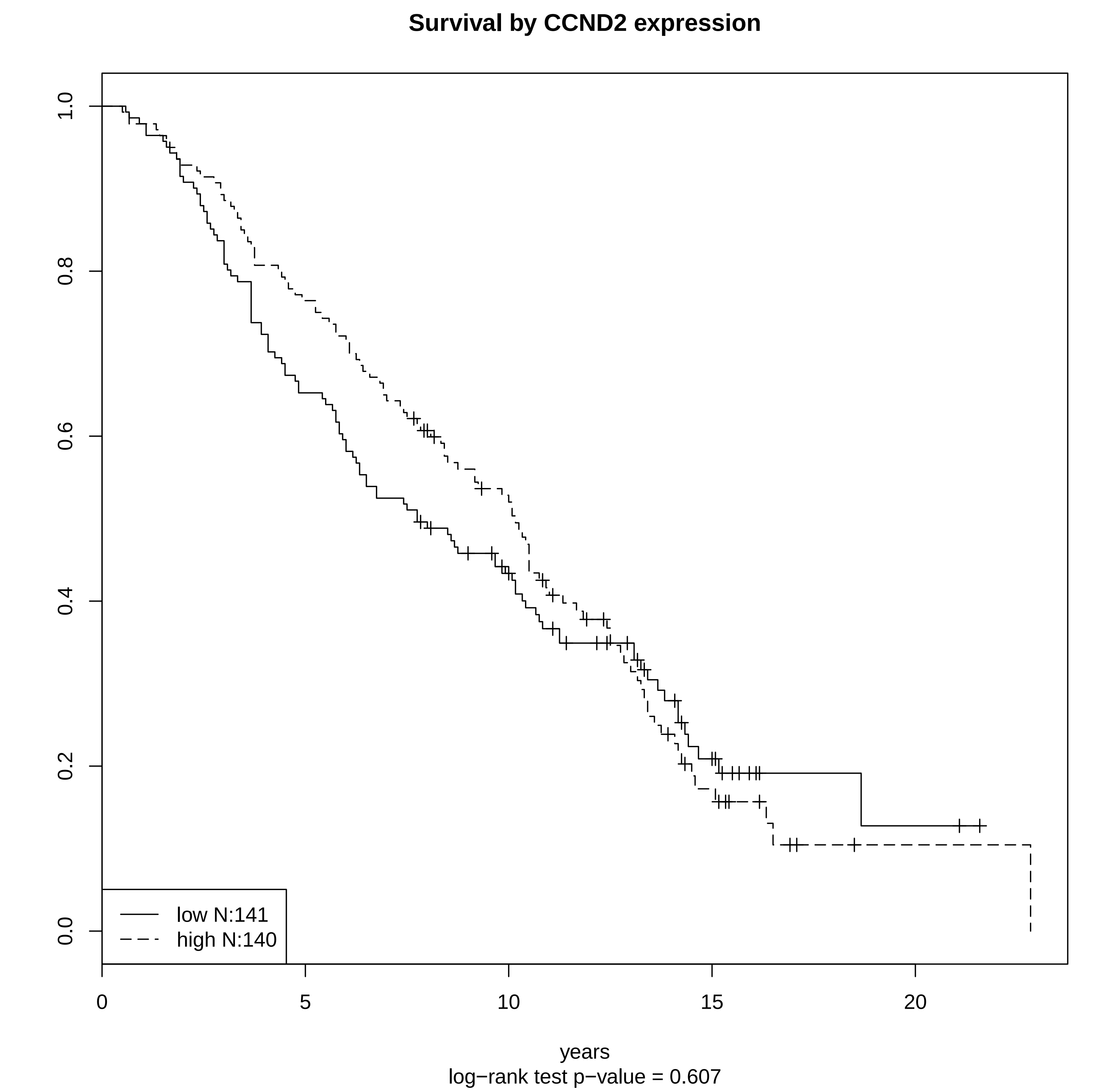
Survival by CCND2 expression
1.0
0.8
0.6
0.4
0.2
0.0
0
5
10
15
20
low N:141
high N:140
years
log−rank test p−value = 0.607

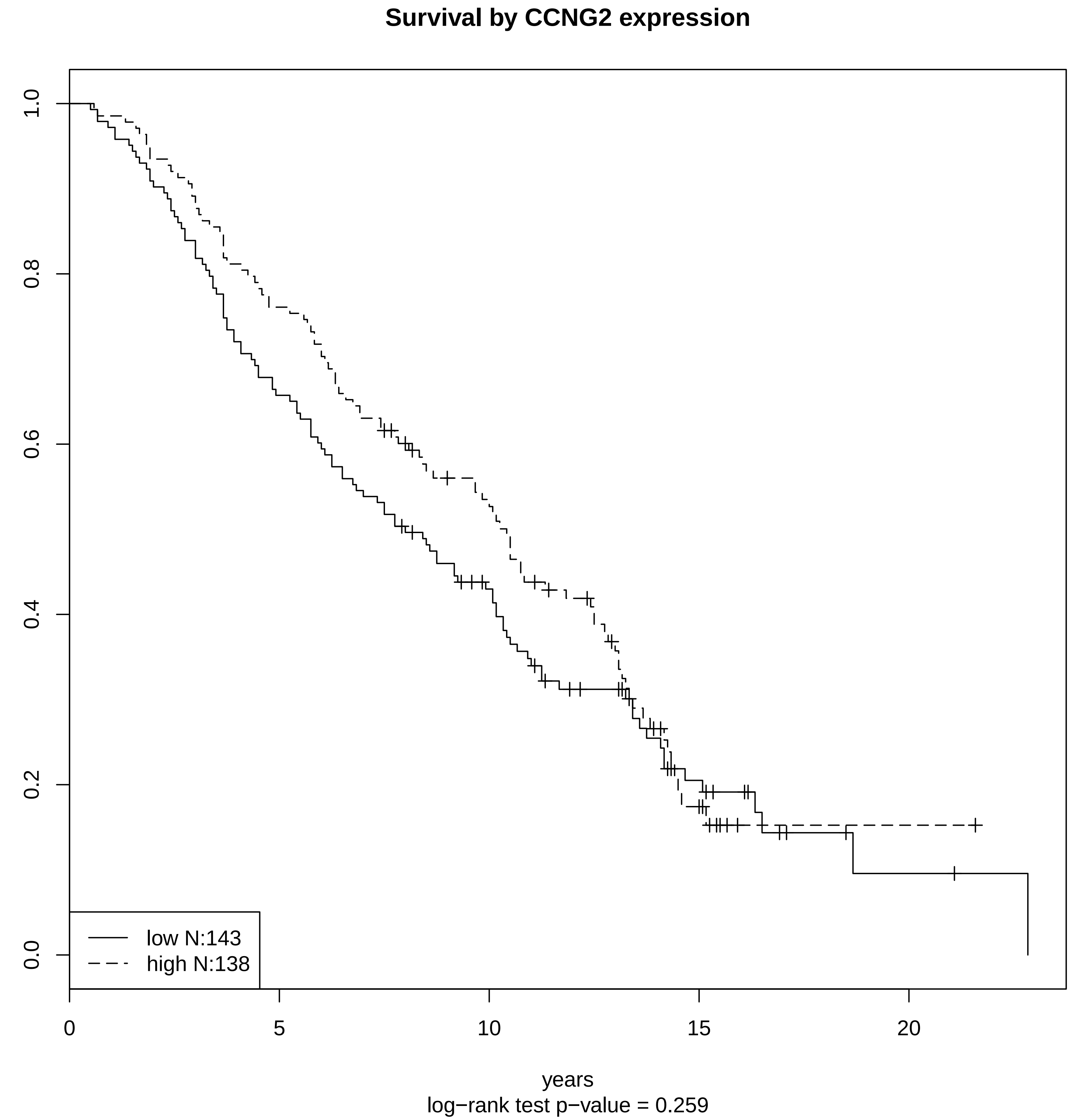
Survival by CCNG2 expression
1.0
0.8
0.6
0.4
0.2
0.0
0
5
10
15
20
low N:143
high N:138
years
log−rank test p−value = 0.259

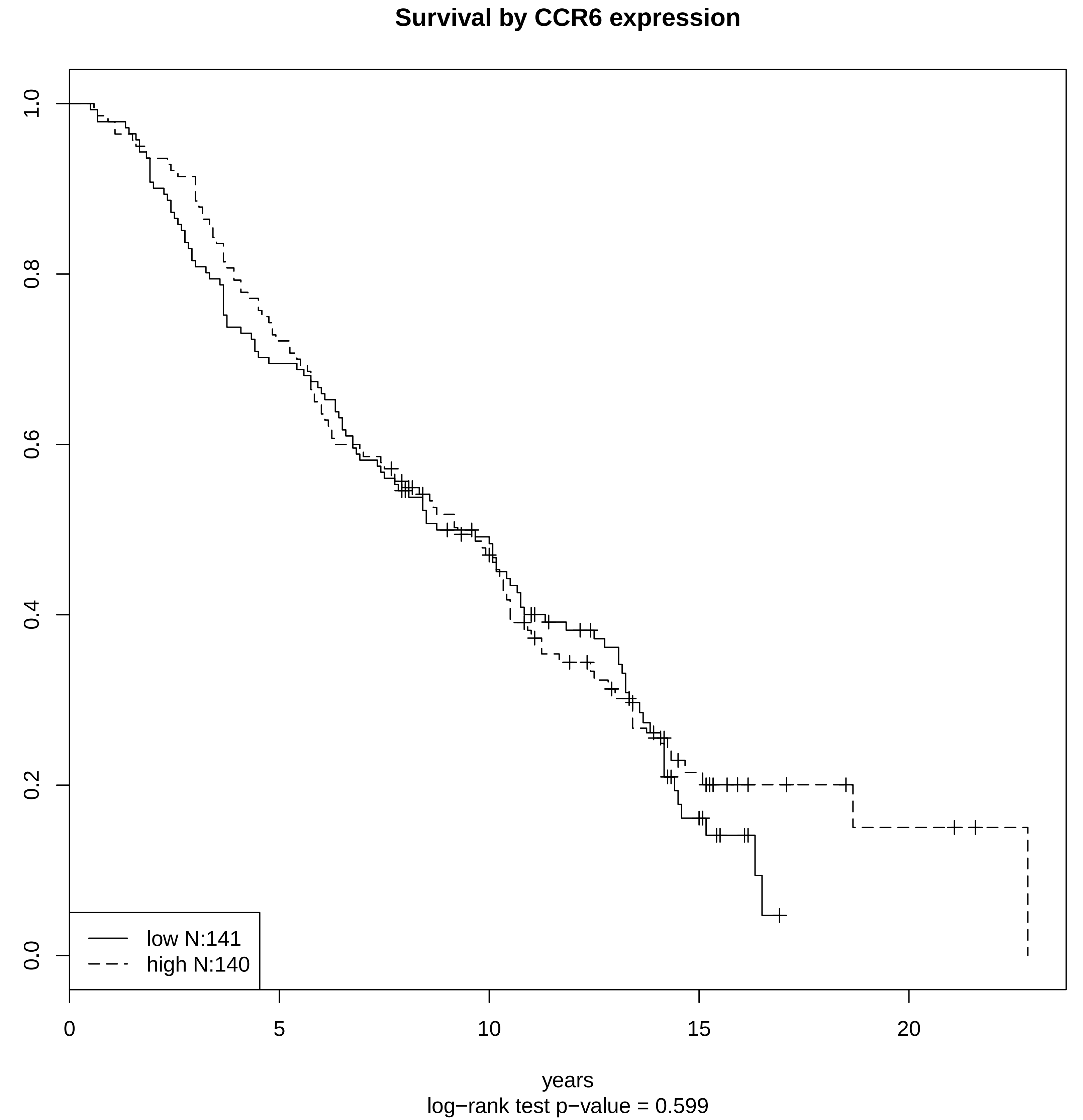

Survival by CCR6 expression
1.0
0.8
0.6
0.4
0.2
0.0
0
5
10
15
20
low N:141
high N:140
years
log−rank test p−value = 0.599

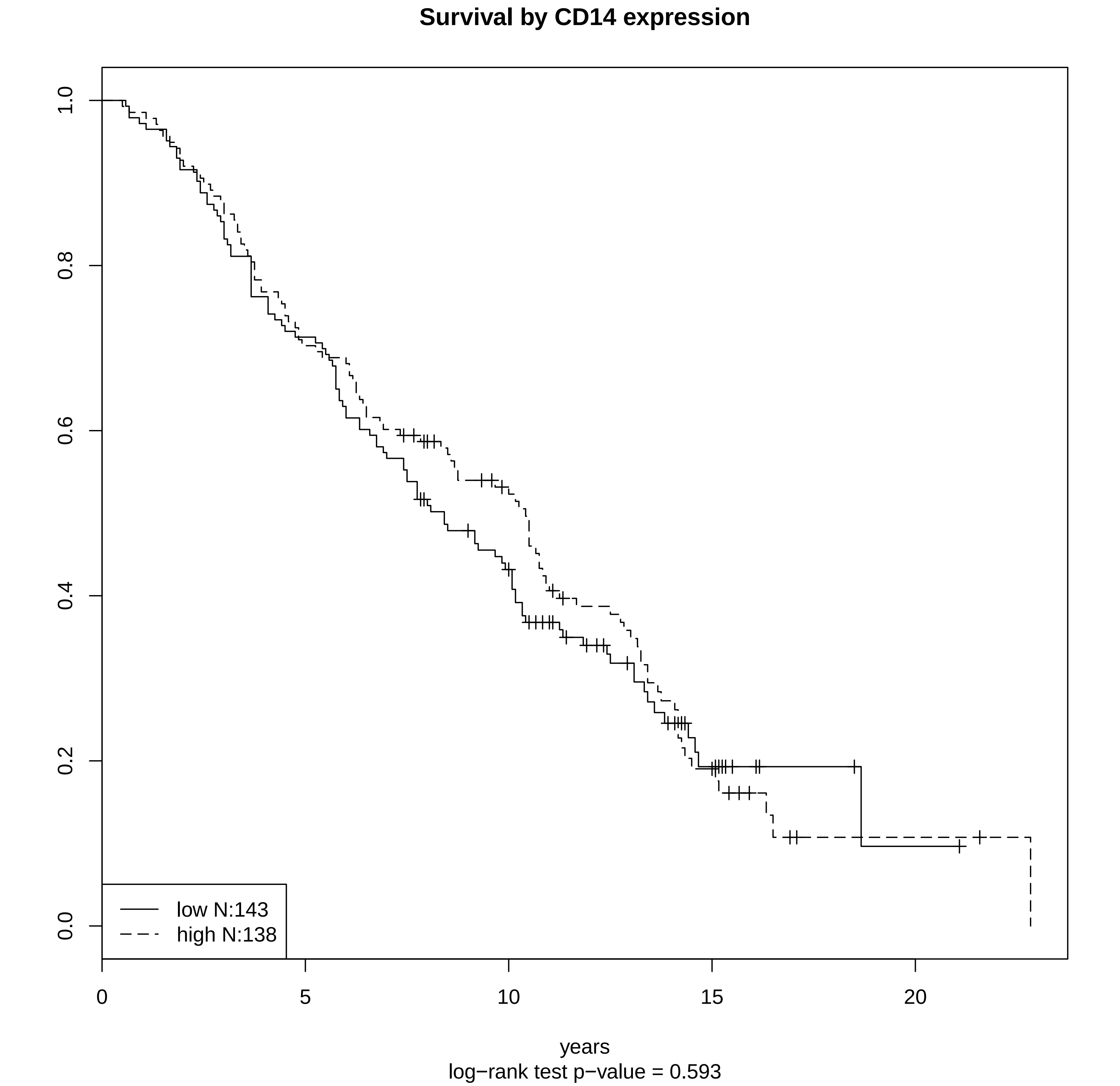
Survival by CD14 expression
1.0
0.8
0.6
0.4
0.2
0.0
0
5
10
15
20
low N:143
high N:138
years
log−rank test p−value = 0.593

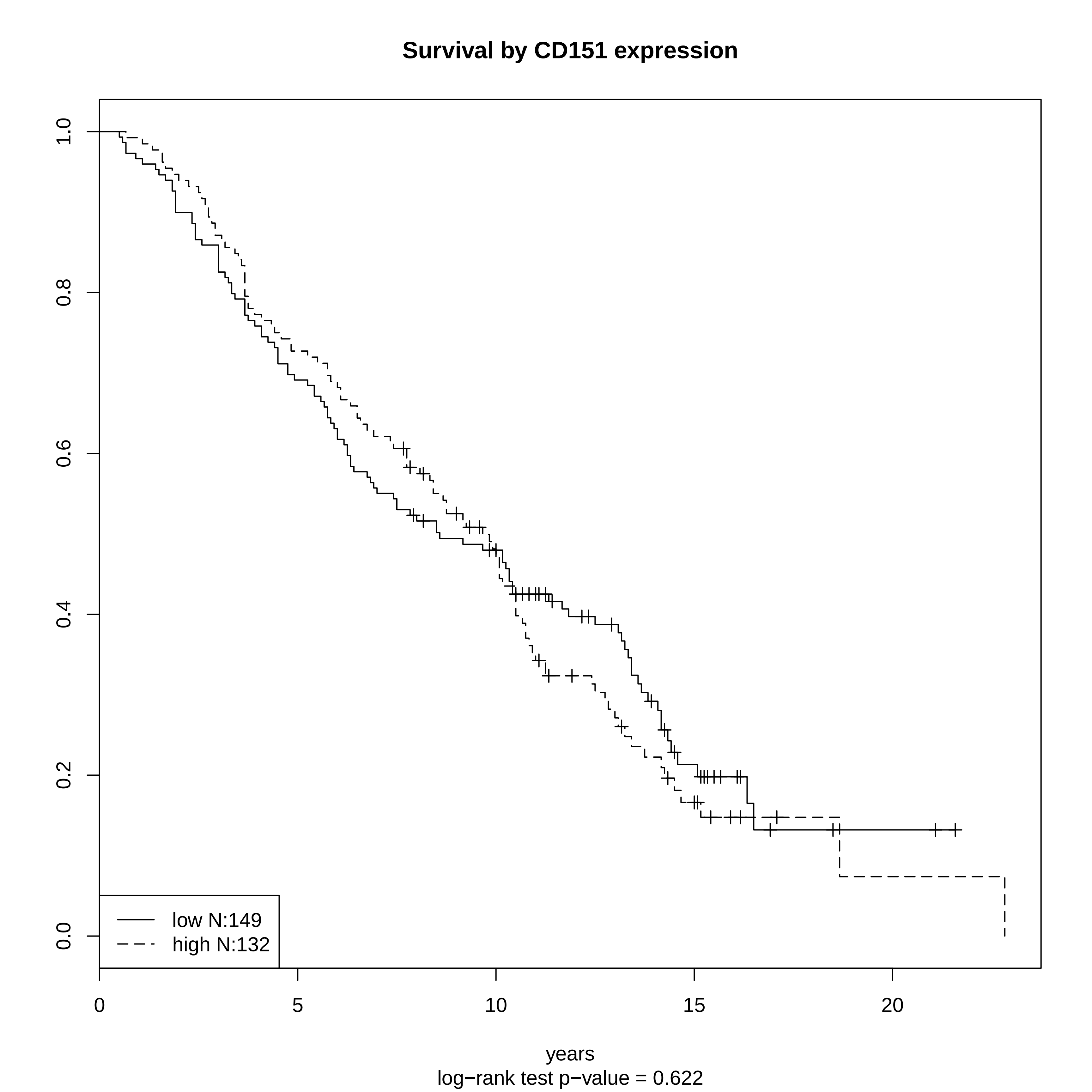

Survival by CD151 expression
1.0
0.8
0.6
0.4
0.2
0.0
0
5
10
15
20
low N:149
high N:132
years
log−rank test p−value = 0.622

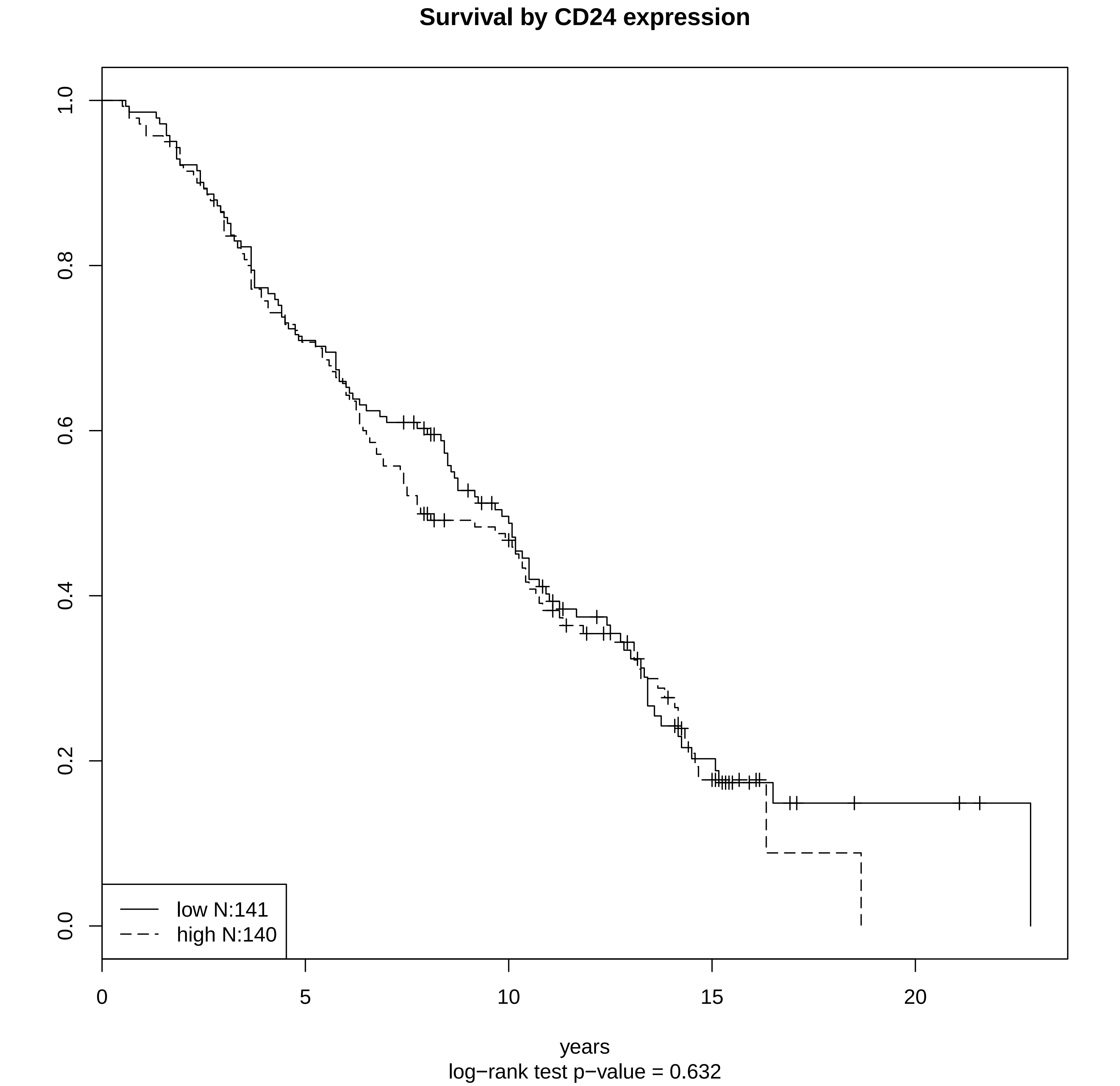

Survival by CD24 expression
1.0
0.8
0.6
0.4
0.2
0.0
0
5
10
15
20
low N:141
high N:140
years
log−rank test p−value = 0.632

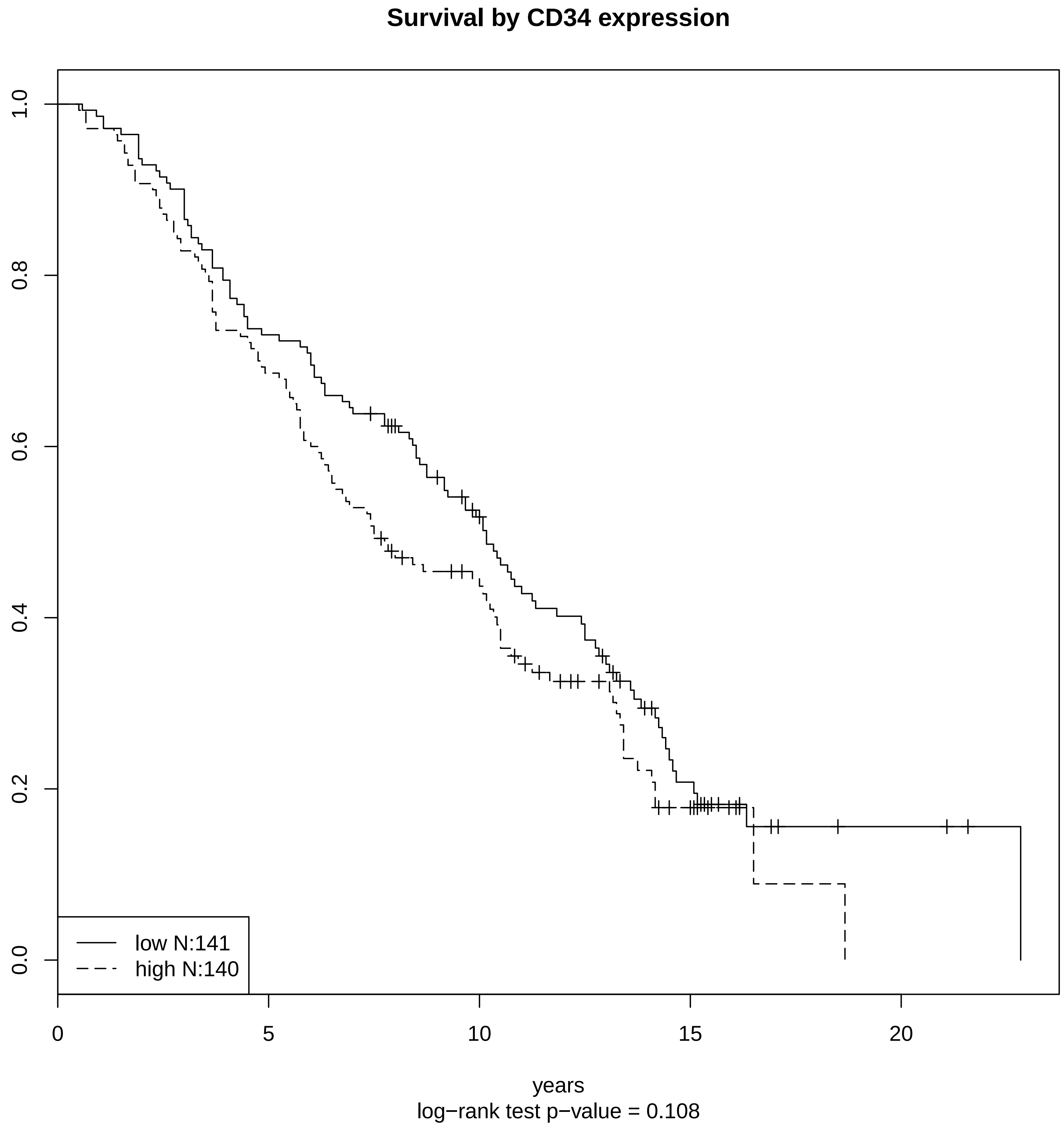
Survival by CD34 expression
1.0
0.8
0.6
0.4
0.2
0.0
0
5
10
15
20
years
low N:141
high N:140
log−rank test p−value = 0.108

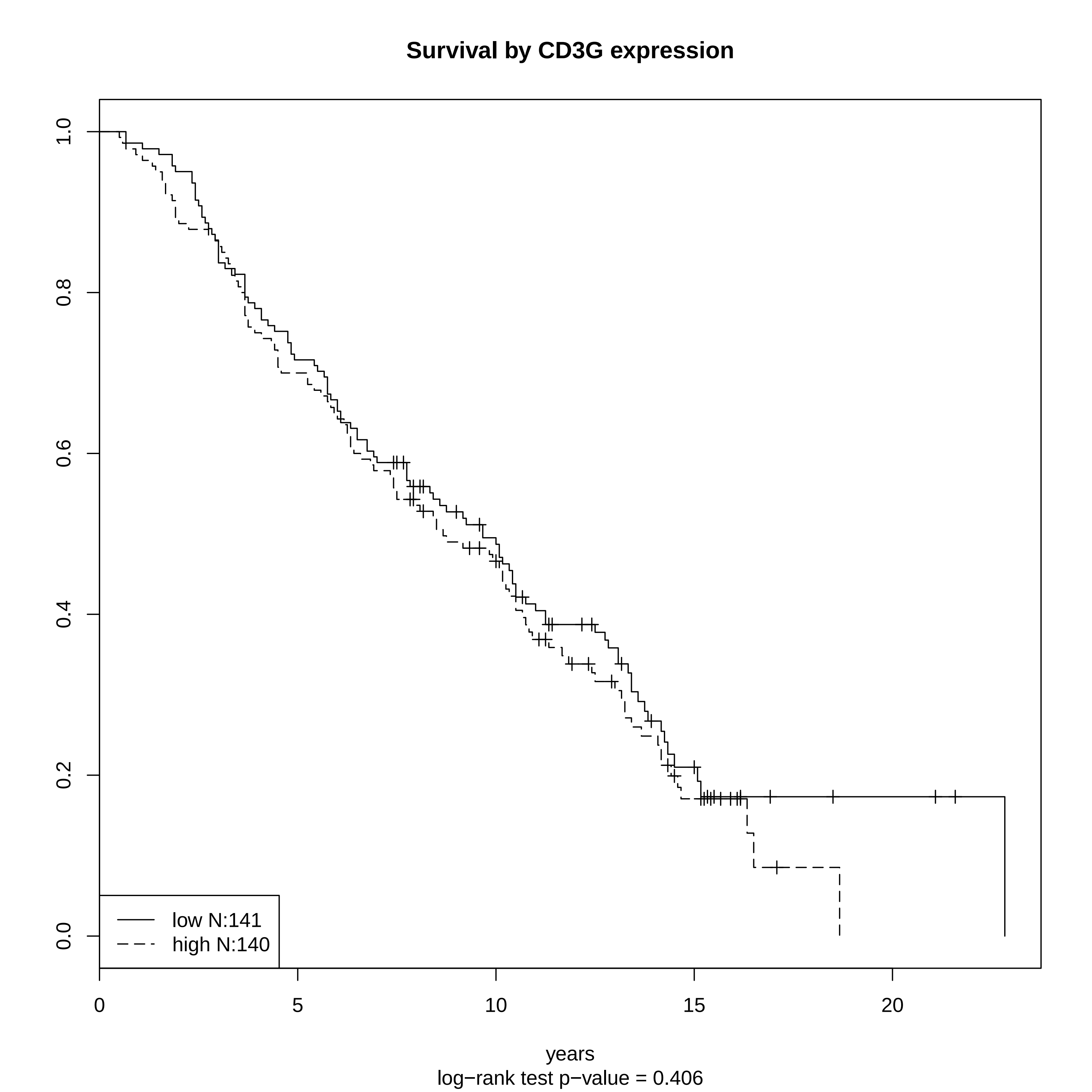

Survival by CD3G expression
1.0
0.8
0.6
0.4
0.2
0.0
0
5
10
15
20
low N:141
high N:140
years
log−rank test p−value = 0.406

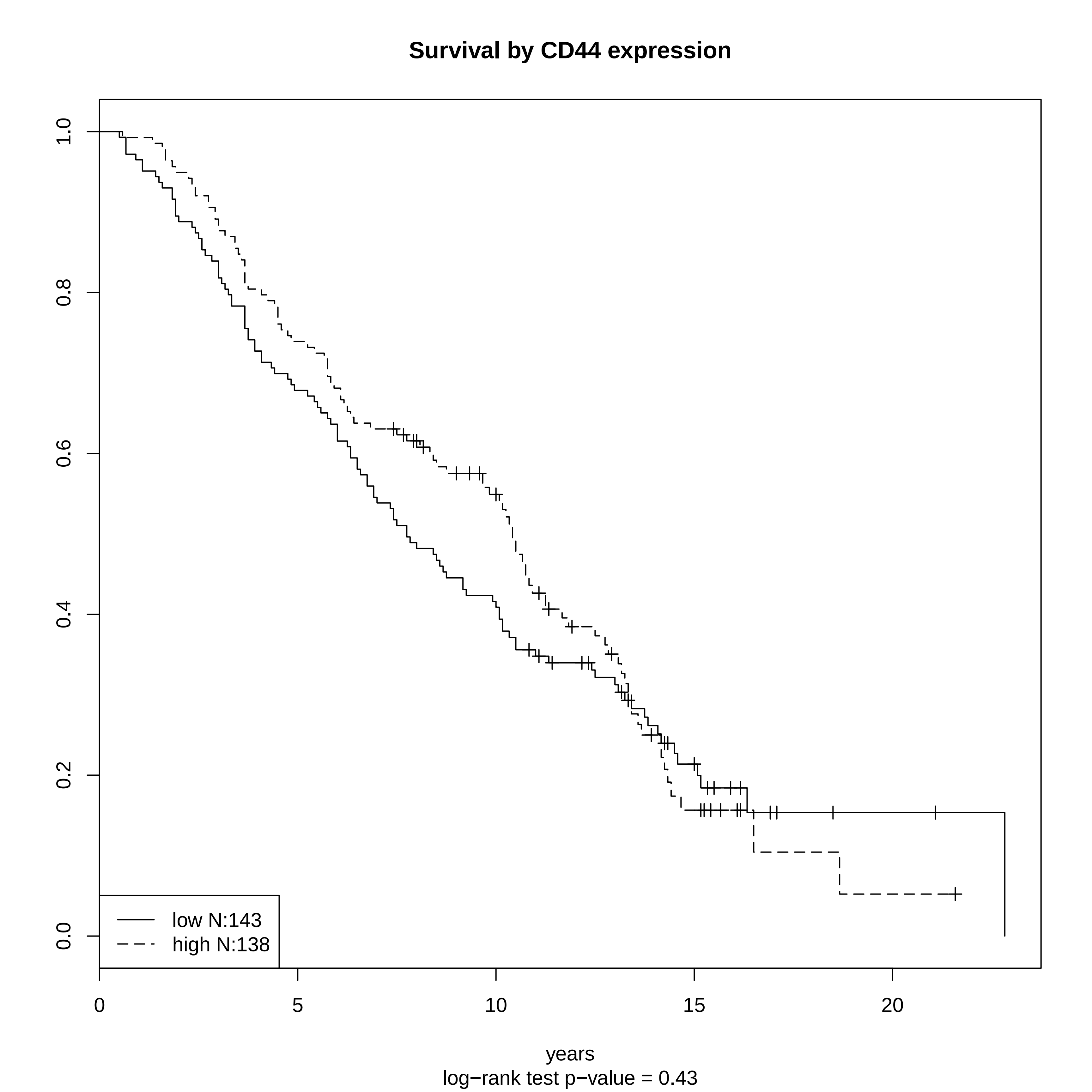
Survival by CD44 expression
1.0
0.8
0.6
0.4
0.2
0.0
0
5
10
15
20
years
log−rank test p−value = 0.43
low N:143
high N:138

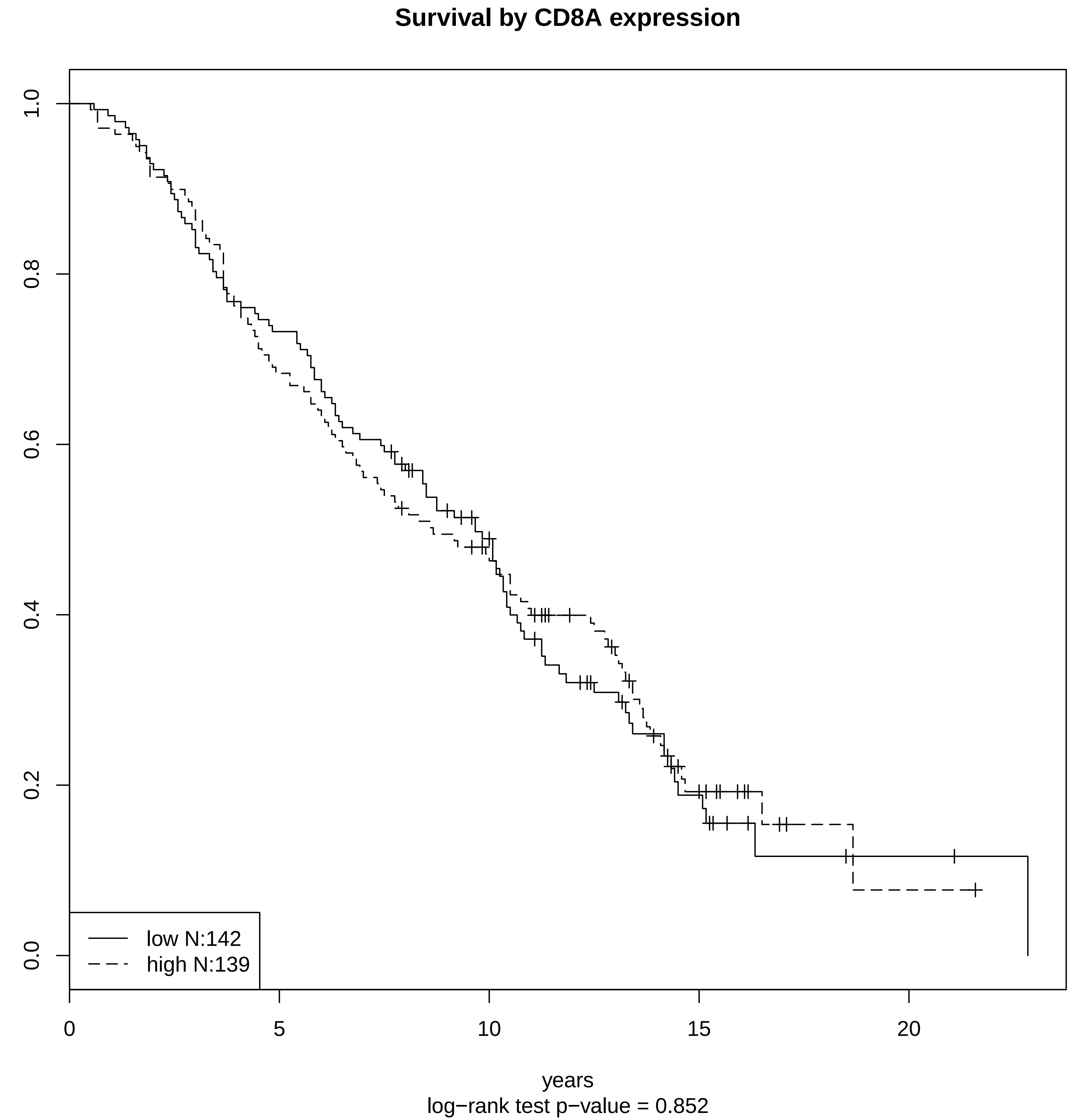

Survival by CD8A expression
1.0
0.8
0.6
0.4
0.2
0.0
0
5
10
15
20
low N:142
high N:139
years
log−rank test p−value = 0.852

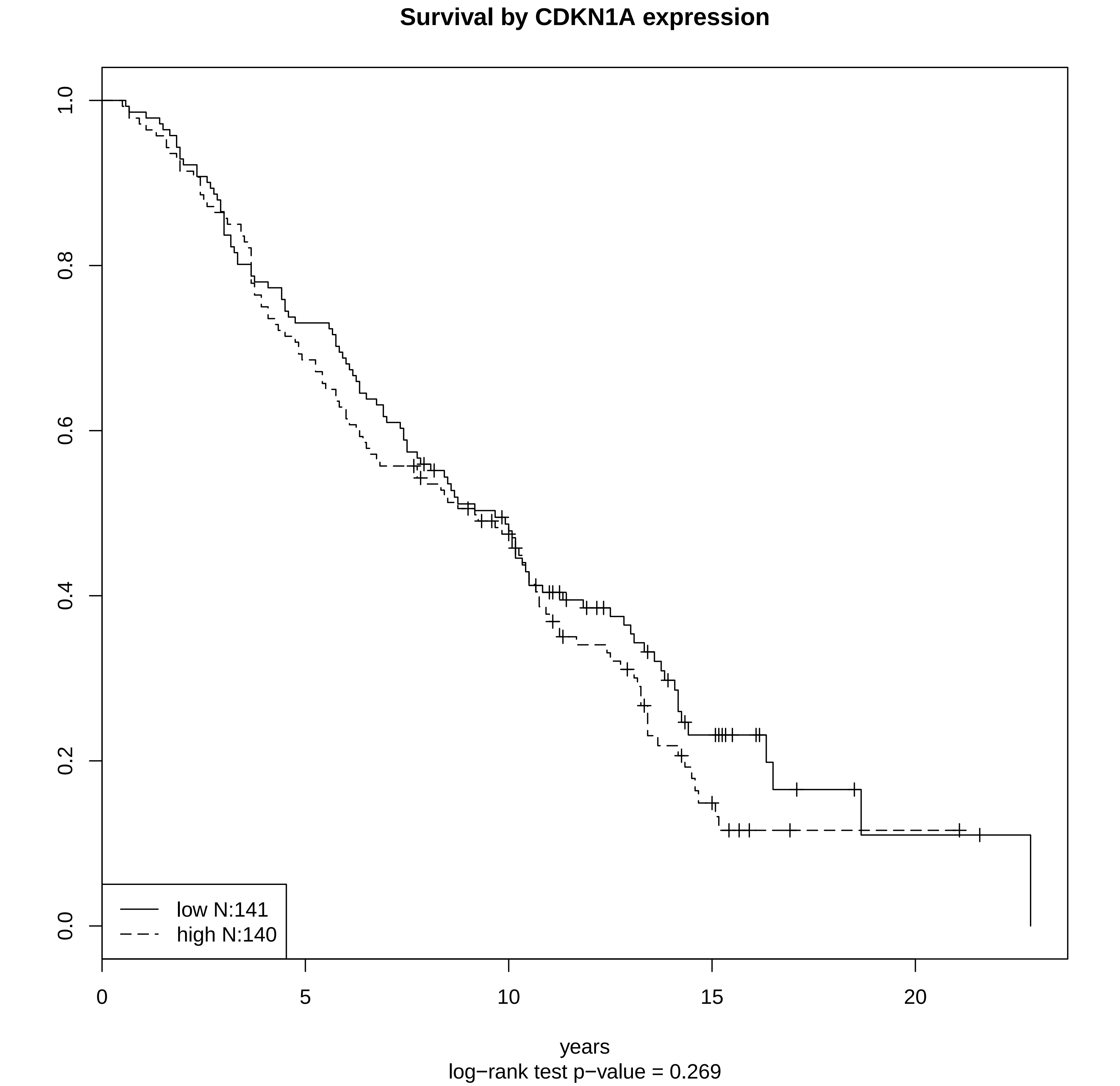

Survival by CDKN1A expression
1.0
0.8
0.6
0.4
0.2
0.0
0
5
10
15
20
low N:141
high N:140
years
log−rank test p−value = 0.269

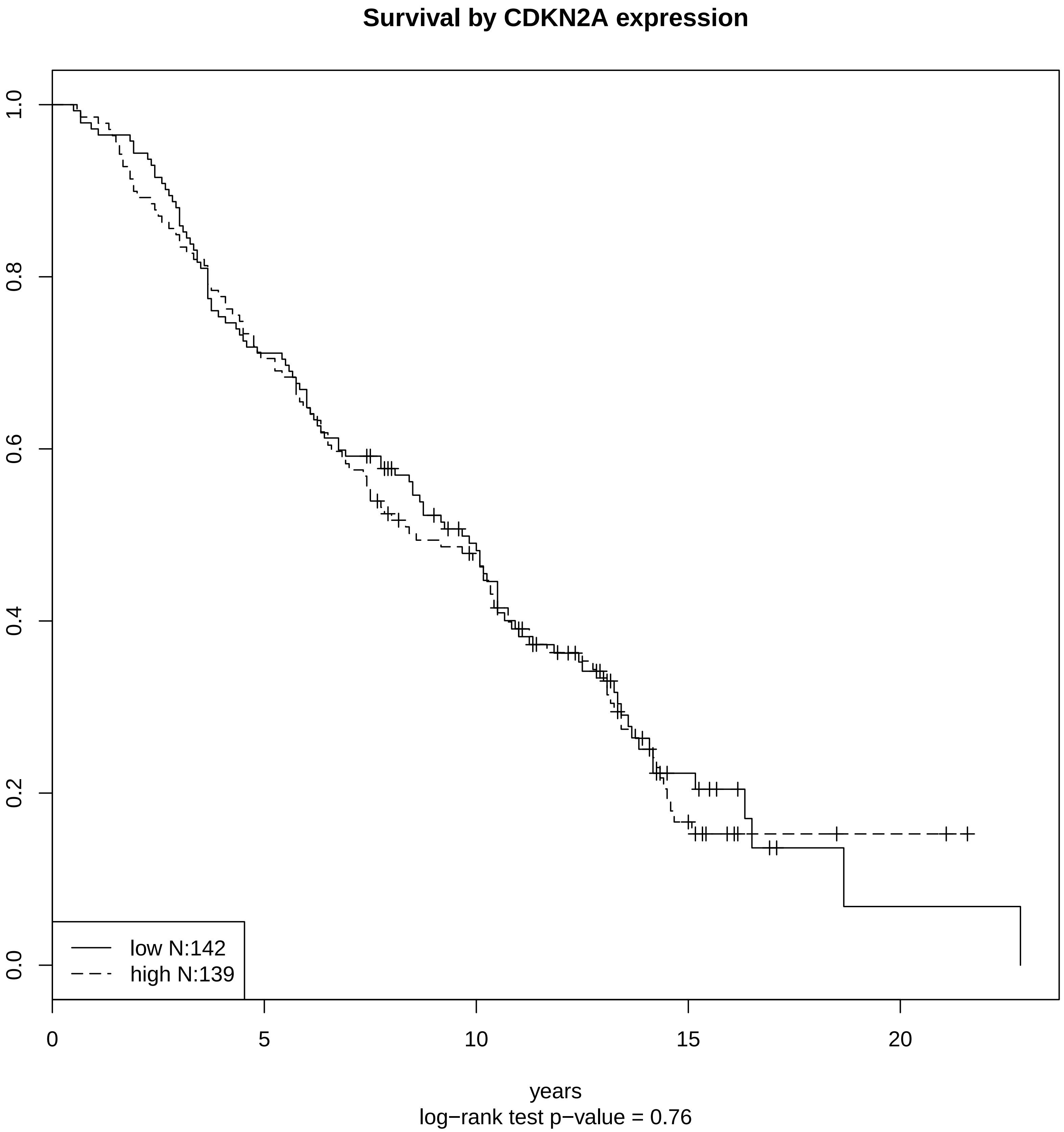
Survival by CDKN2A expression
1.0
0.8
0.6
0.4
0.2
0.0
0
5
10
15
20
years
log−rank test p−value = 0.76
low N:142
high N:139

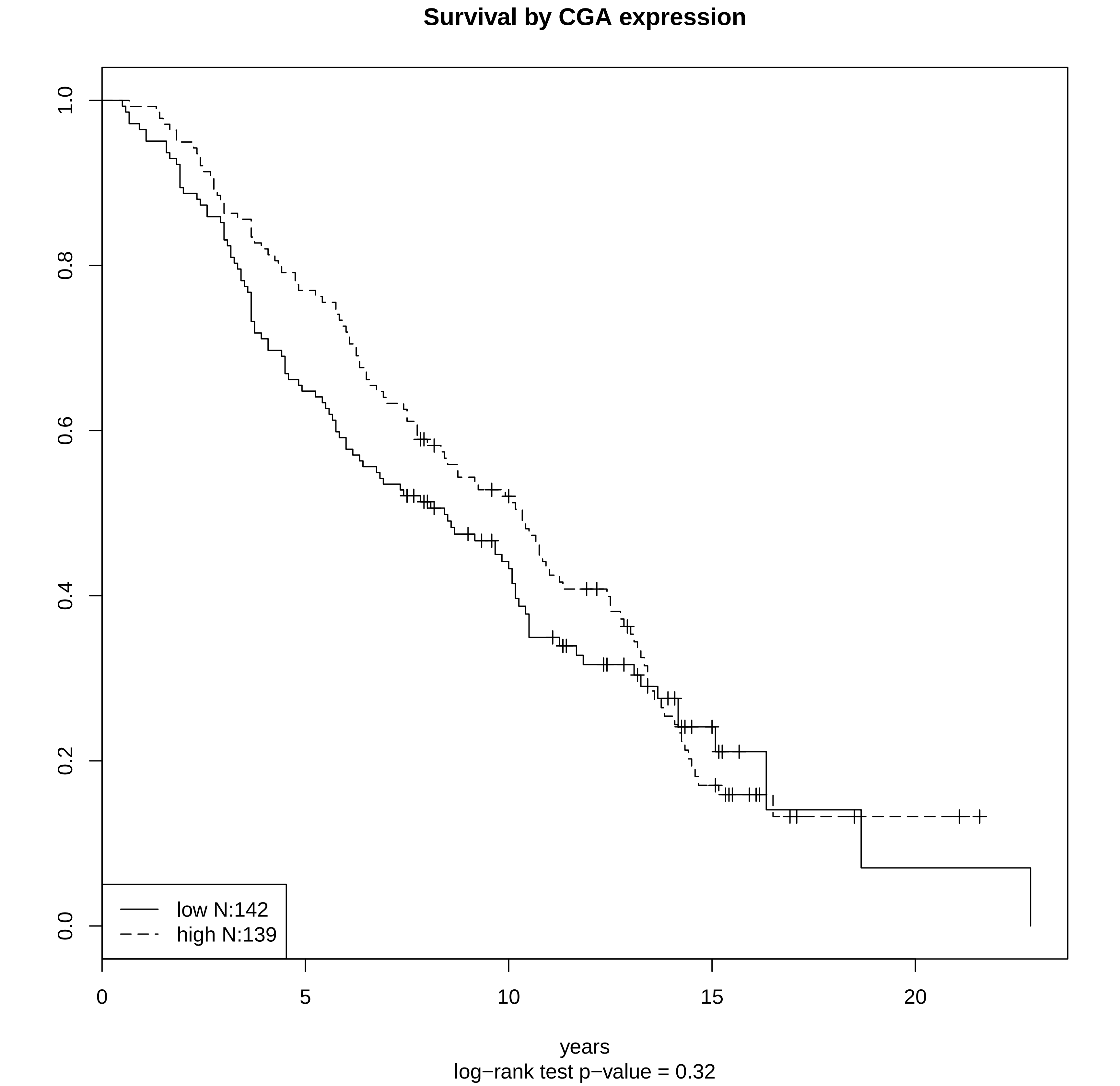

Survival by CGA expression
1.0
0.8
0.6
0.4
0.2
0.0
0
5
10
15
20
years
log−rank test p−value = 0.32
low N:142
high N:139

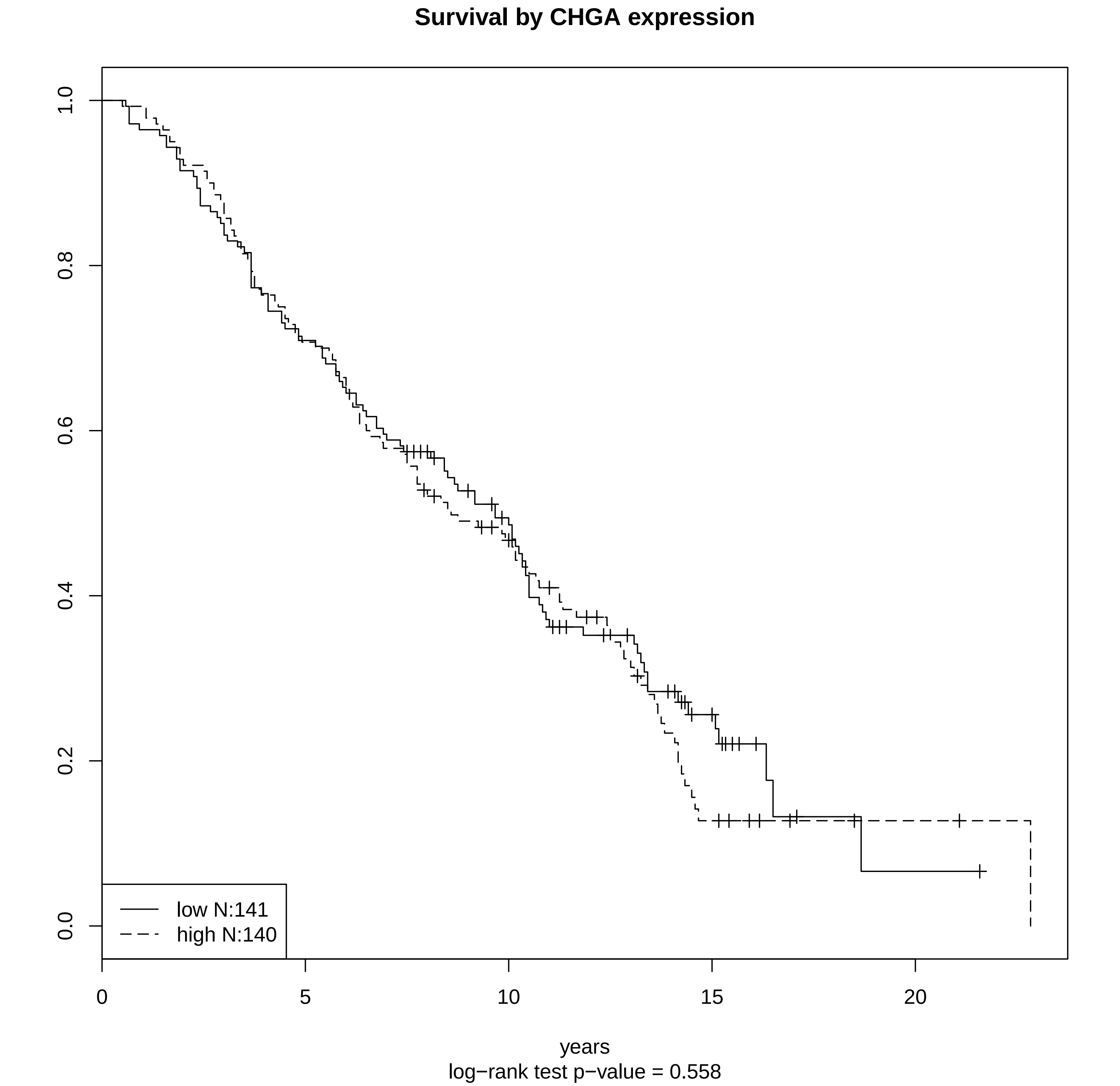

Survival by CHGA expression
1.0
0.8
0.6
0.4
0.2
0.0
0
5
10
15
20
low N:141
high N:140
years
log−rank test p−value = 0.558

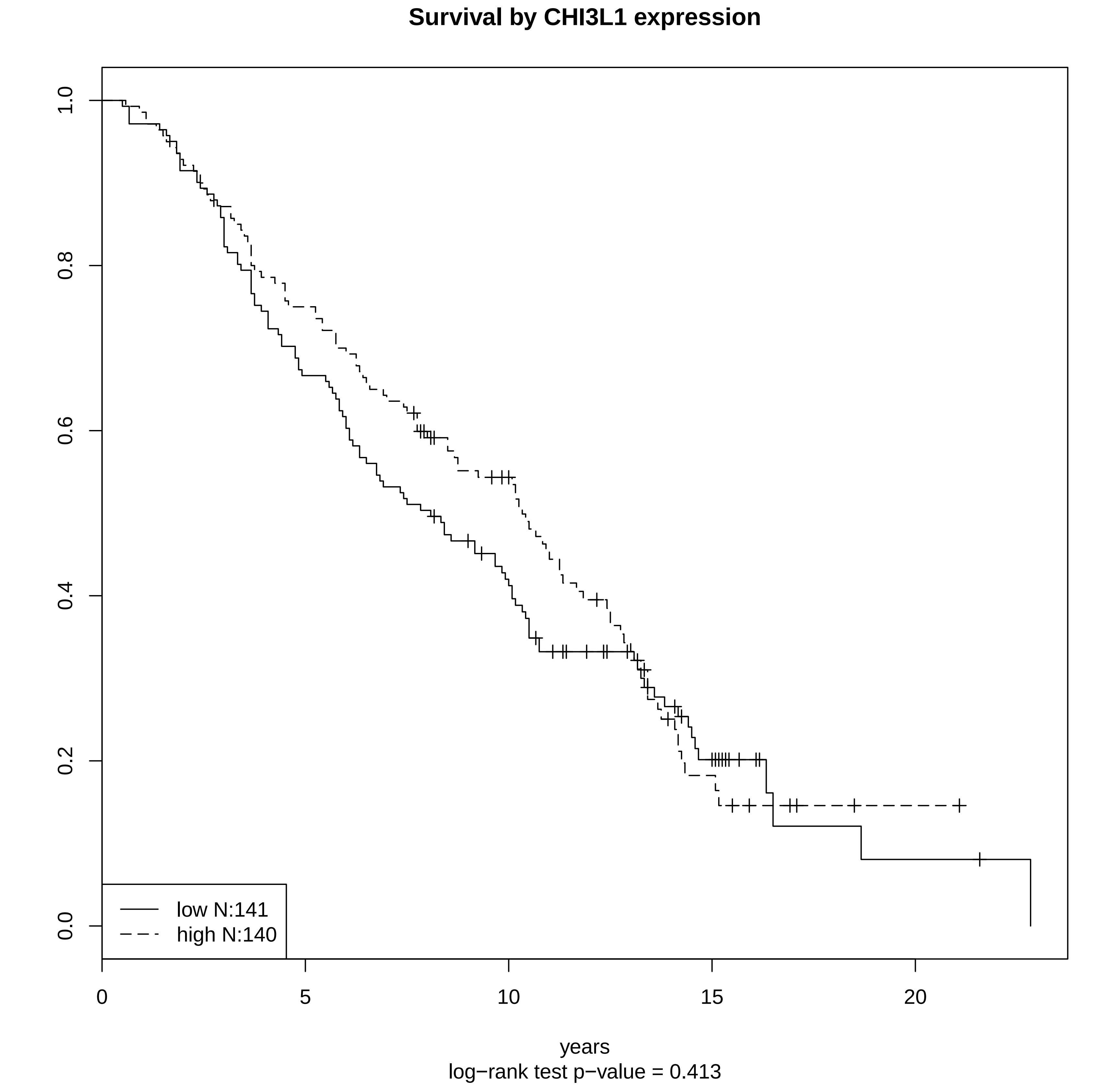

Survival by CHI3L1 expression
1.0
0.8
0.6
0.4
0.2
0.0
0
5
10
15
20
low N:141
high N:140
years
log−rank test p−value = 0.413

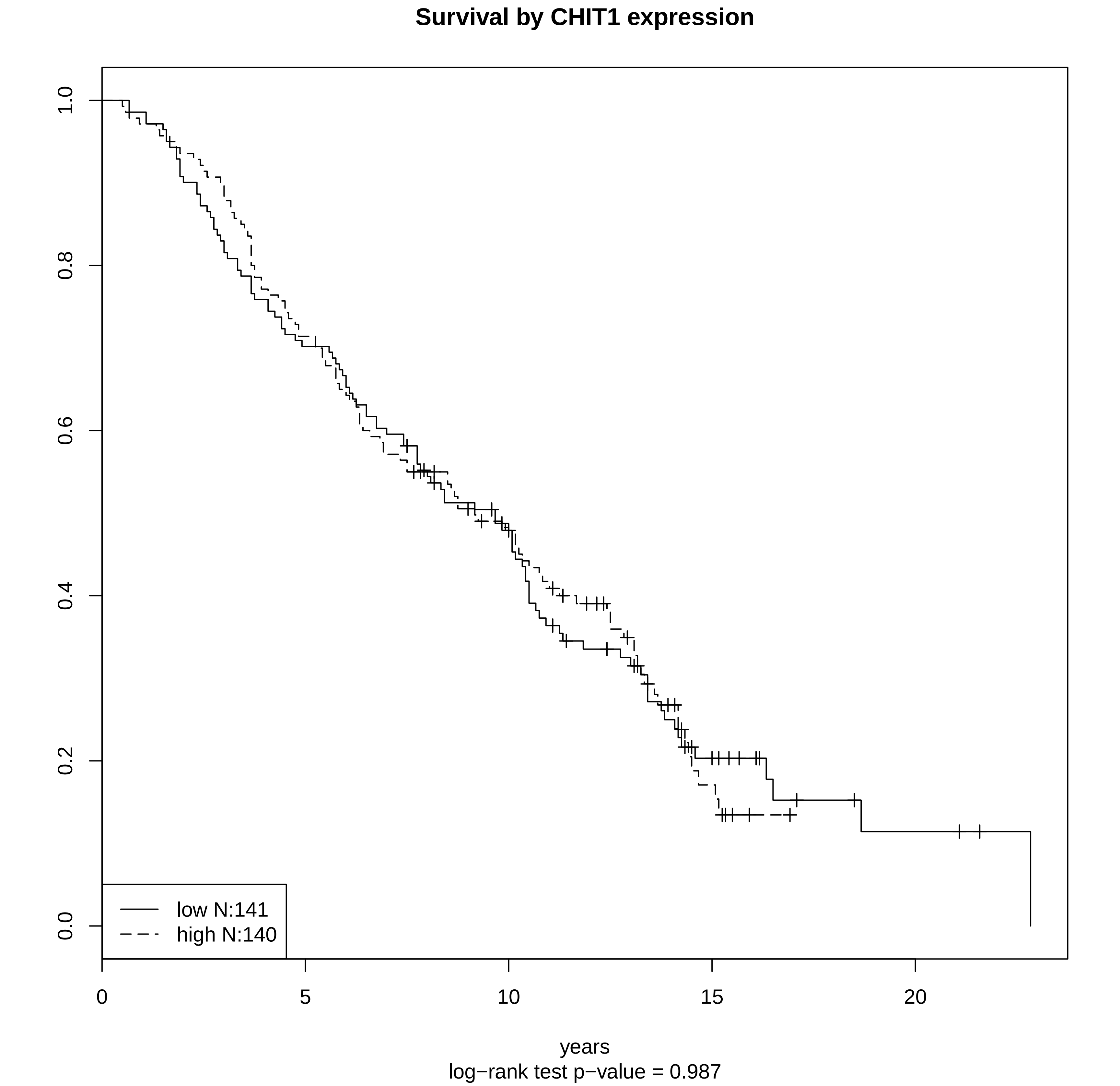
Survival by CHIT1 expression
1.0
0.8
0.6
0.4
0.2
0.0
0
5
10
15
20
low N:141
high N:140
years
log−rank test p−value = 0.987

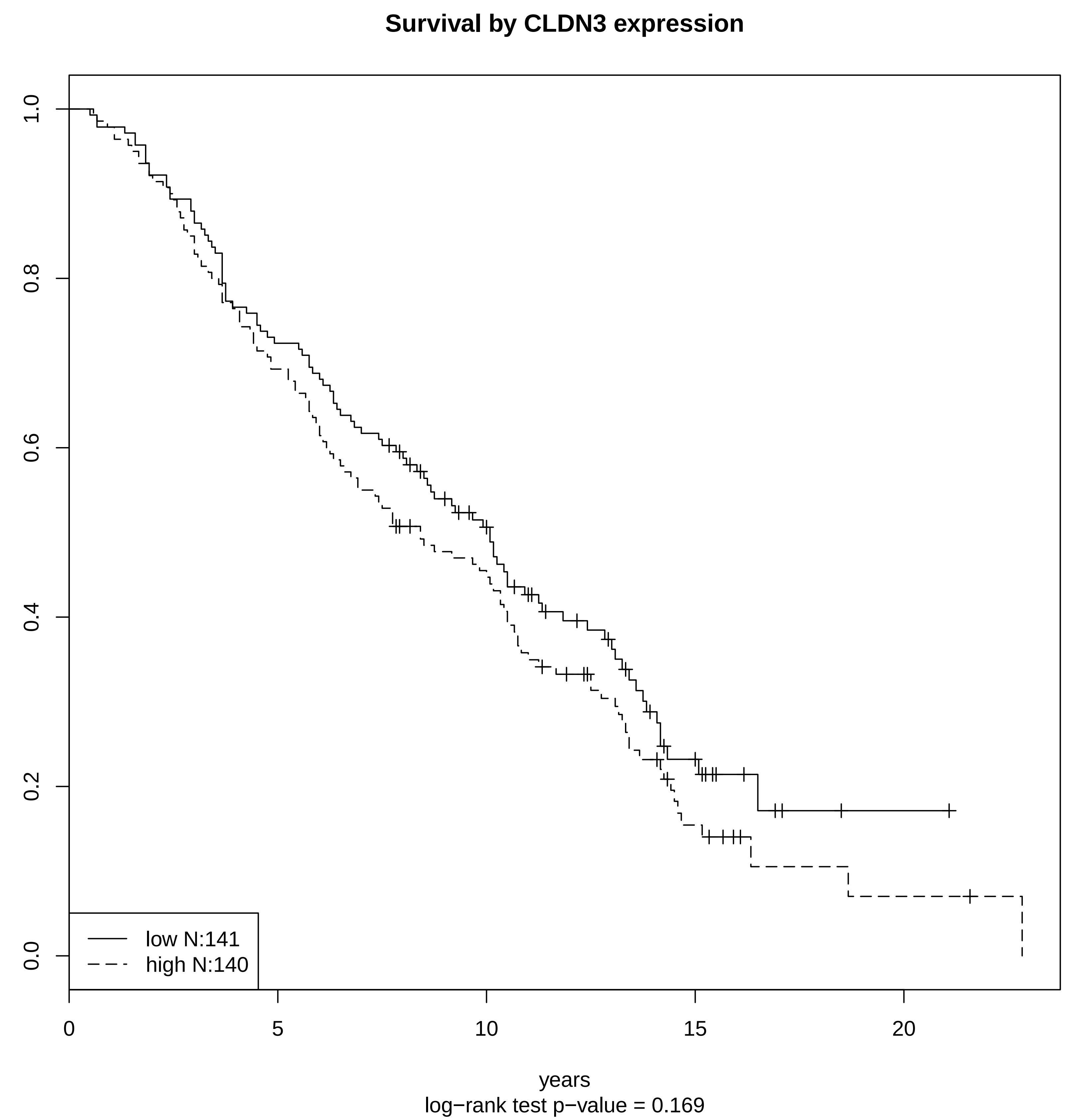
Survival by CLDN3 expression
1.0
0.8
0.6
0.4
0.2
0.0
0
5
10
15
20
years
log−rank test p−value = 0.169
low N:141
high N:140

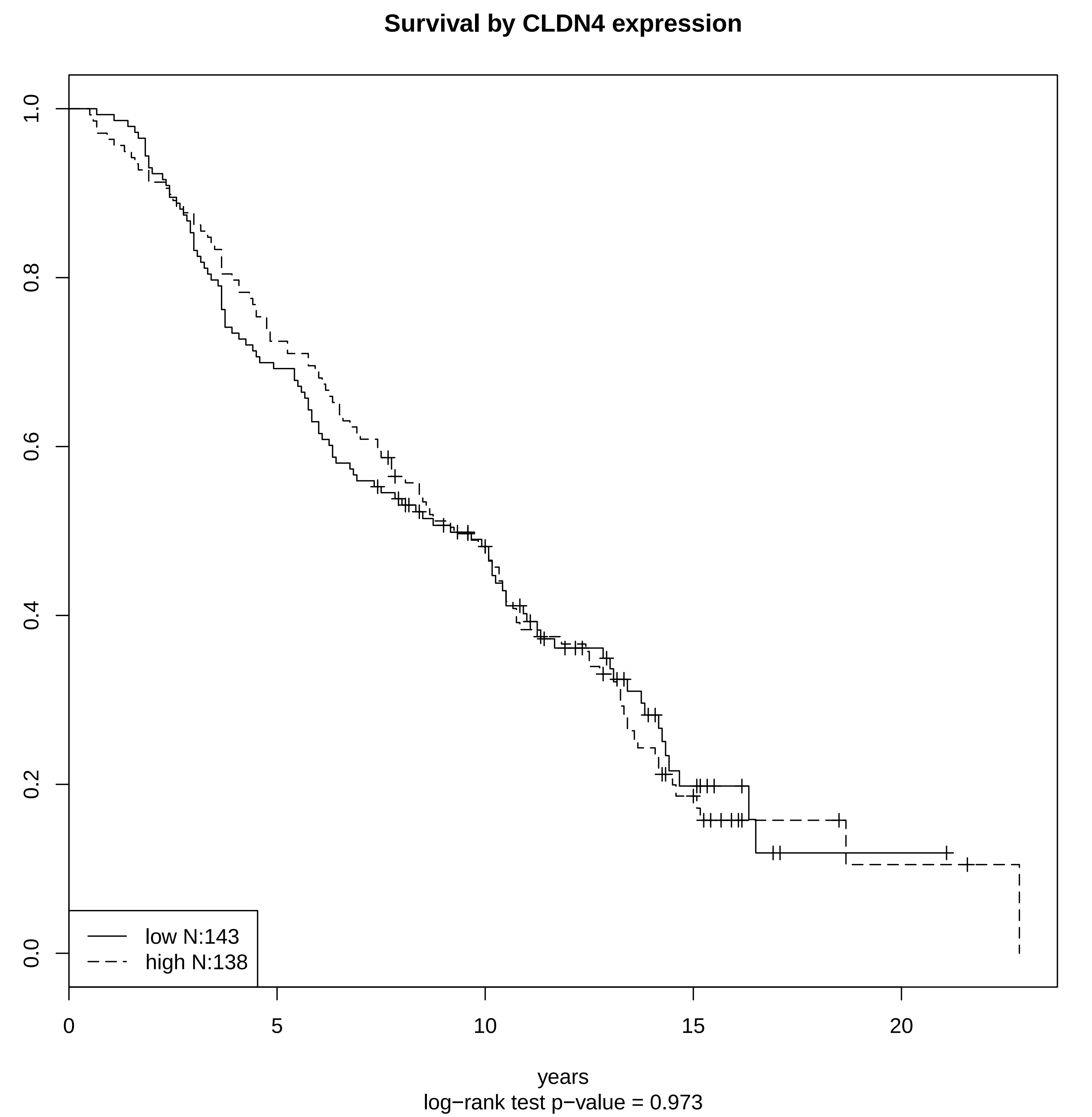

Survival by CLDN4 expression
1.0
0.8
0.6
0.4
0.2
0.0
0
5
10
15
20
years
log−rank test p−value = 0.973
low N:143
high N:138

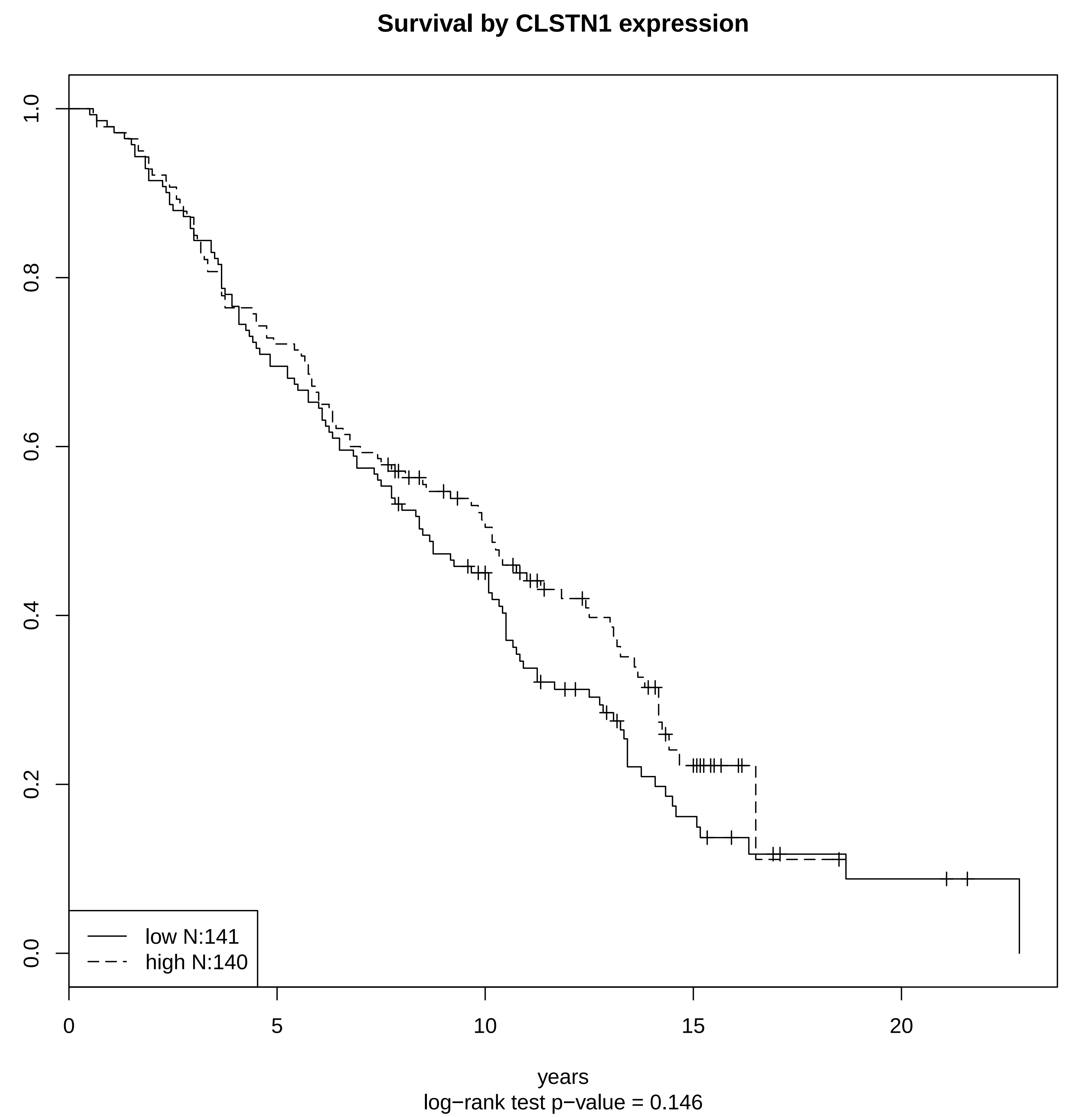
Survival by CLSTN1 expression
1.0
0.8
0.6
0.4
0.2
0.0
0
5
10
15
20
low N:141
high N:140
years
log−rank test p−value = 0.146

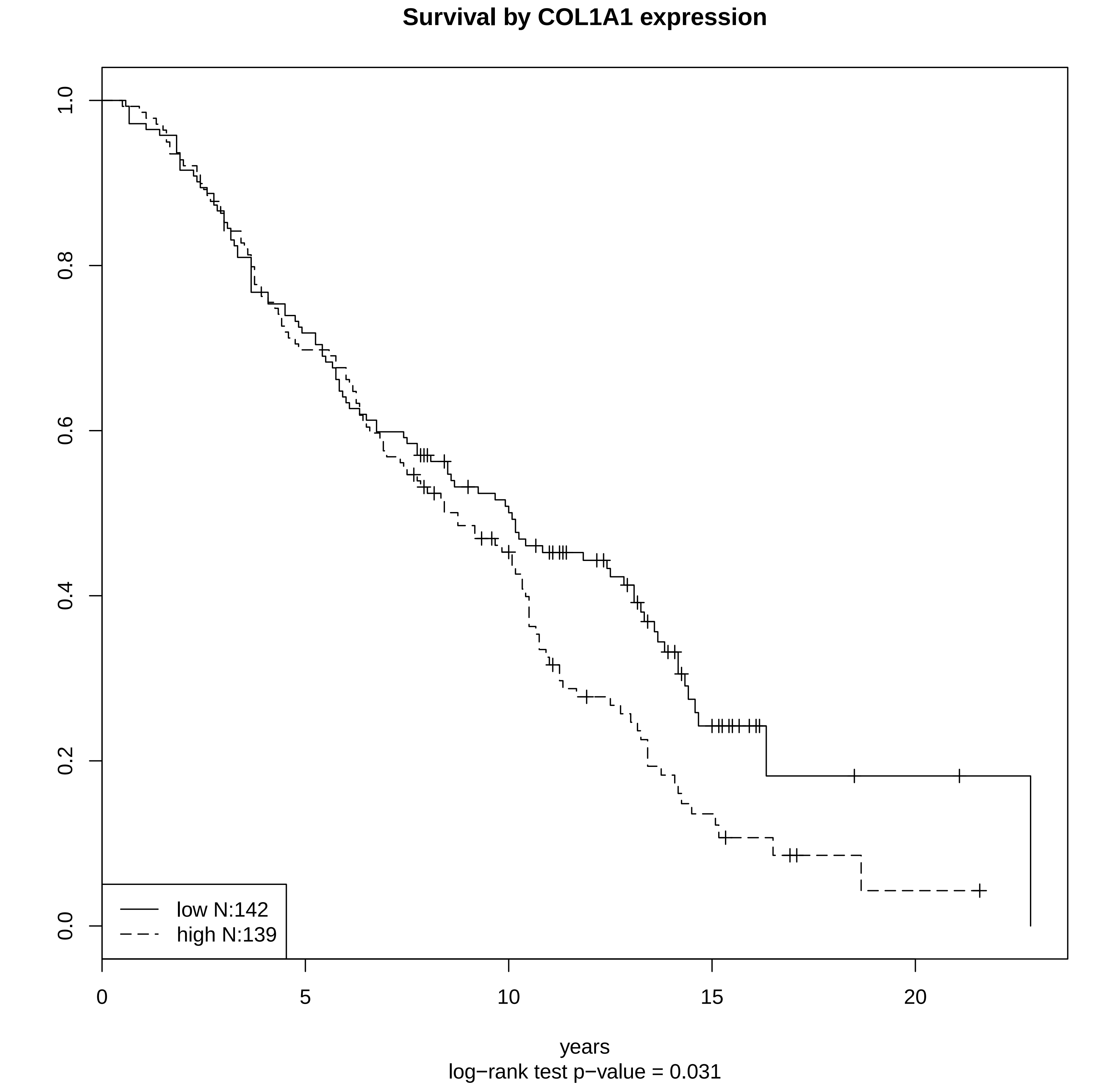

Survival by COL1A1 expression
1.0
0.8
0.6
0.4
0.2
0.0
0
5
10
15
20
low N:142
high N:139
years
log−rank test p−value = 0.031

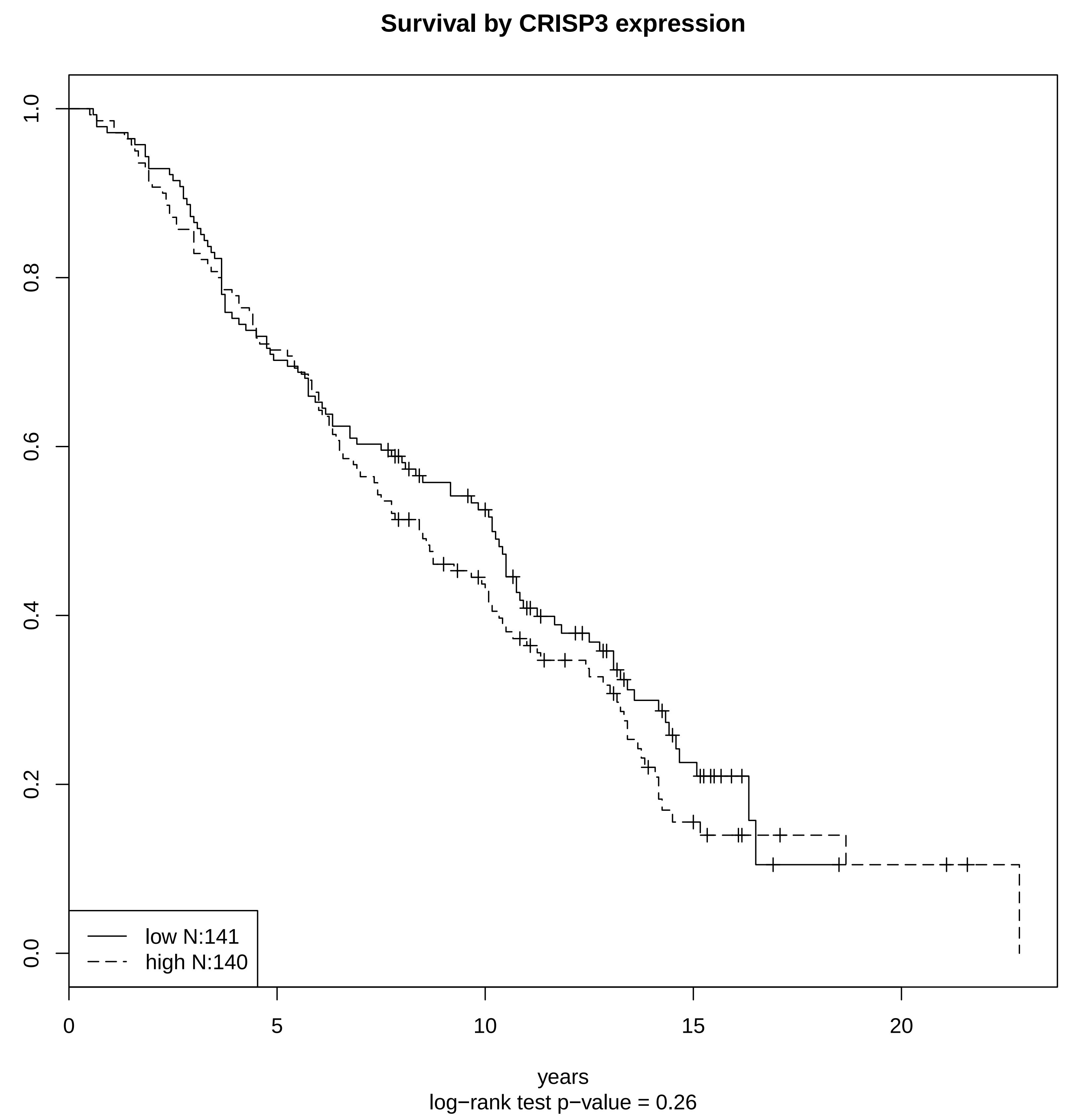

Survival by CRISP3 expression
1.0
0.8
0.6
0.4
0.2
0.0
0
5
10
15
20
low N:141
high N:140
years
log−rank test p−value = 0.26

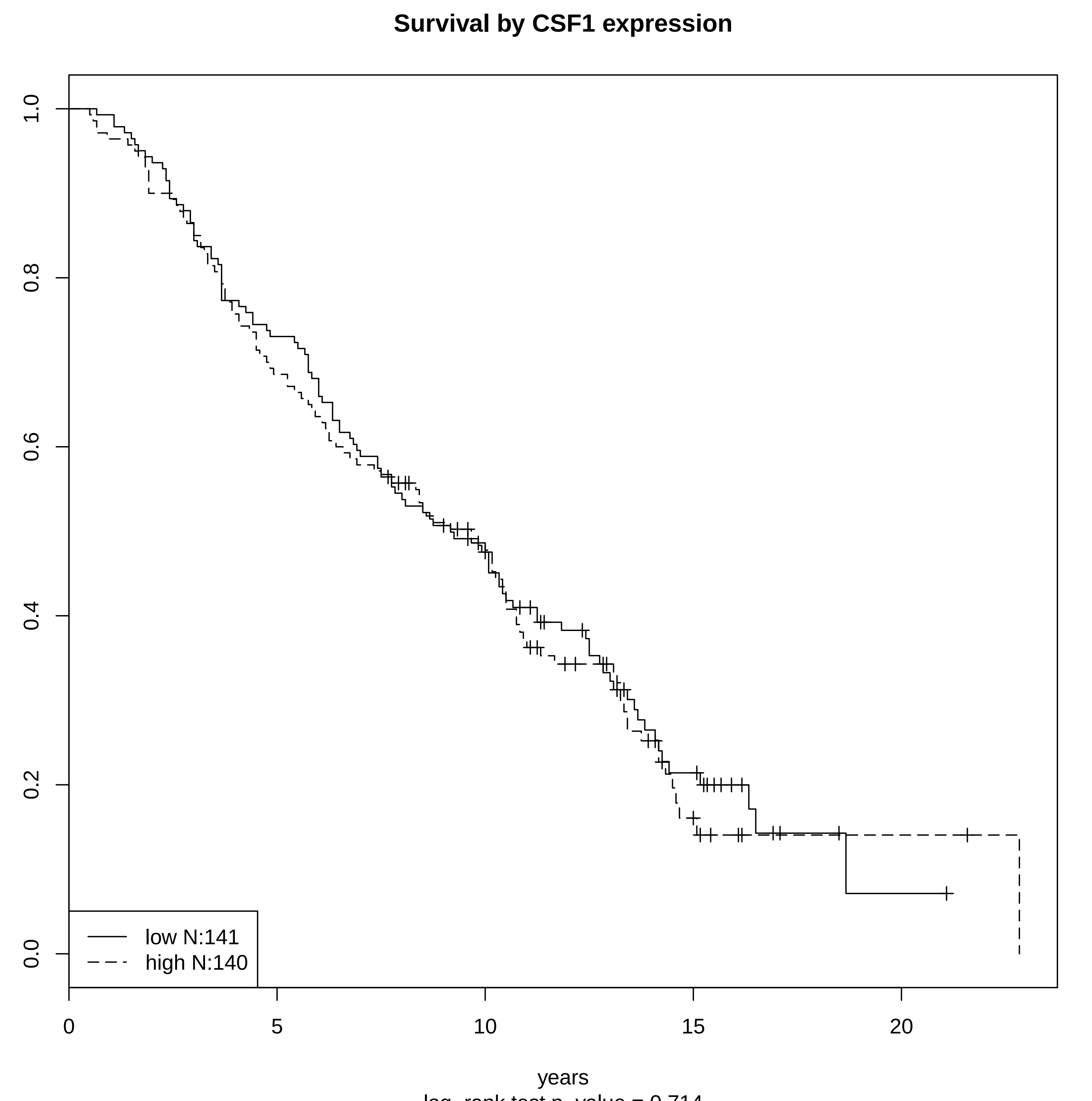

Survival by CSF1 expression
1.0
0.8
0.6
0.4
0.2
0.0
0
5
10
15
20
years
log−rank test p−value = 0.714
low N:141
high N:140

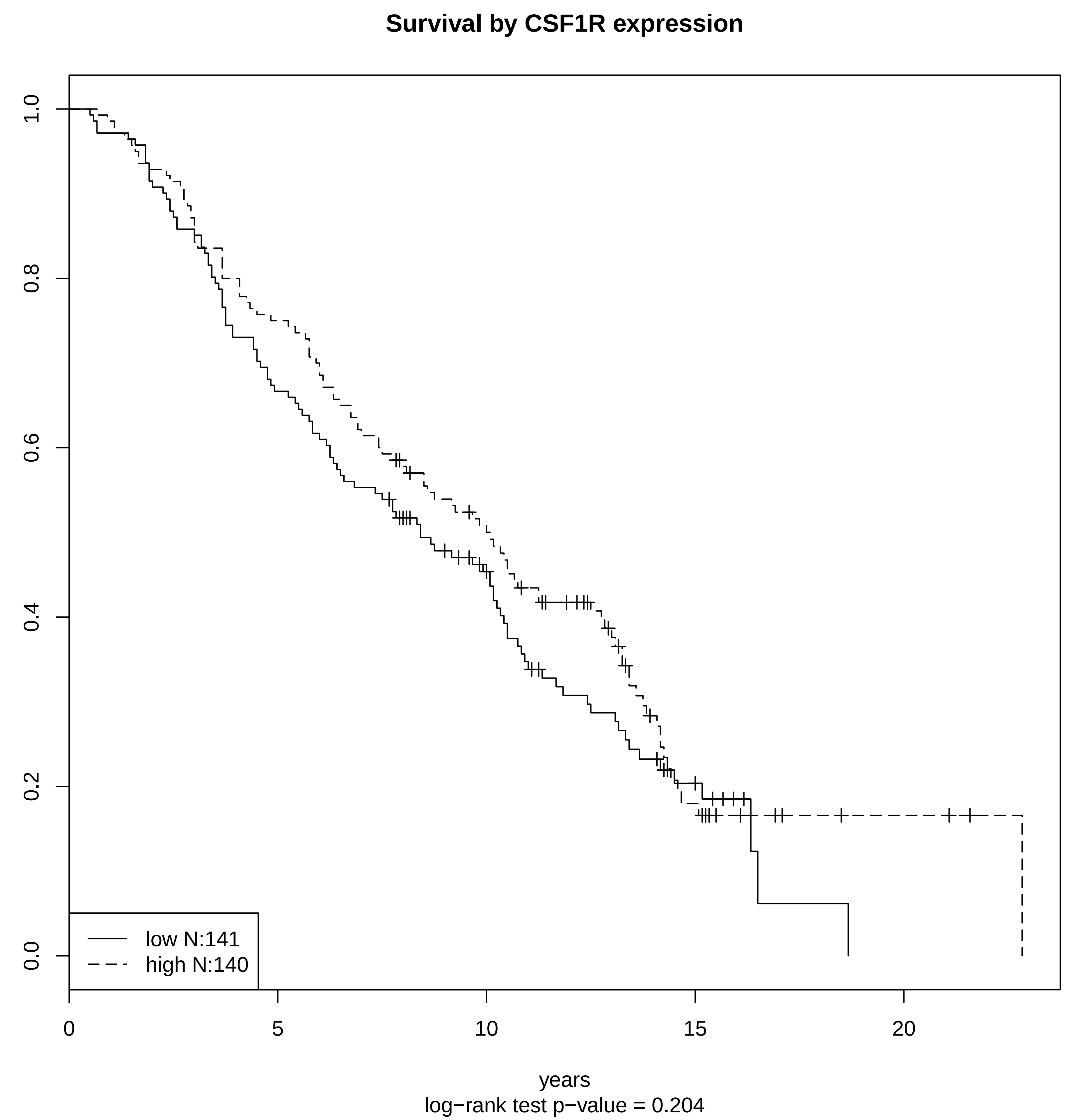
Survival by CSF1R expression
1.0
0.8
0.6
0.4
0.2
0.0
0
5
10
15
20
low N:141
high N:140
years
log−rank test p−value = 0.204

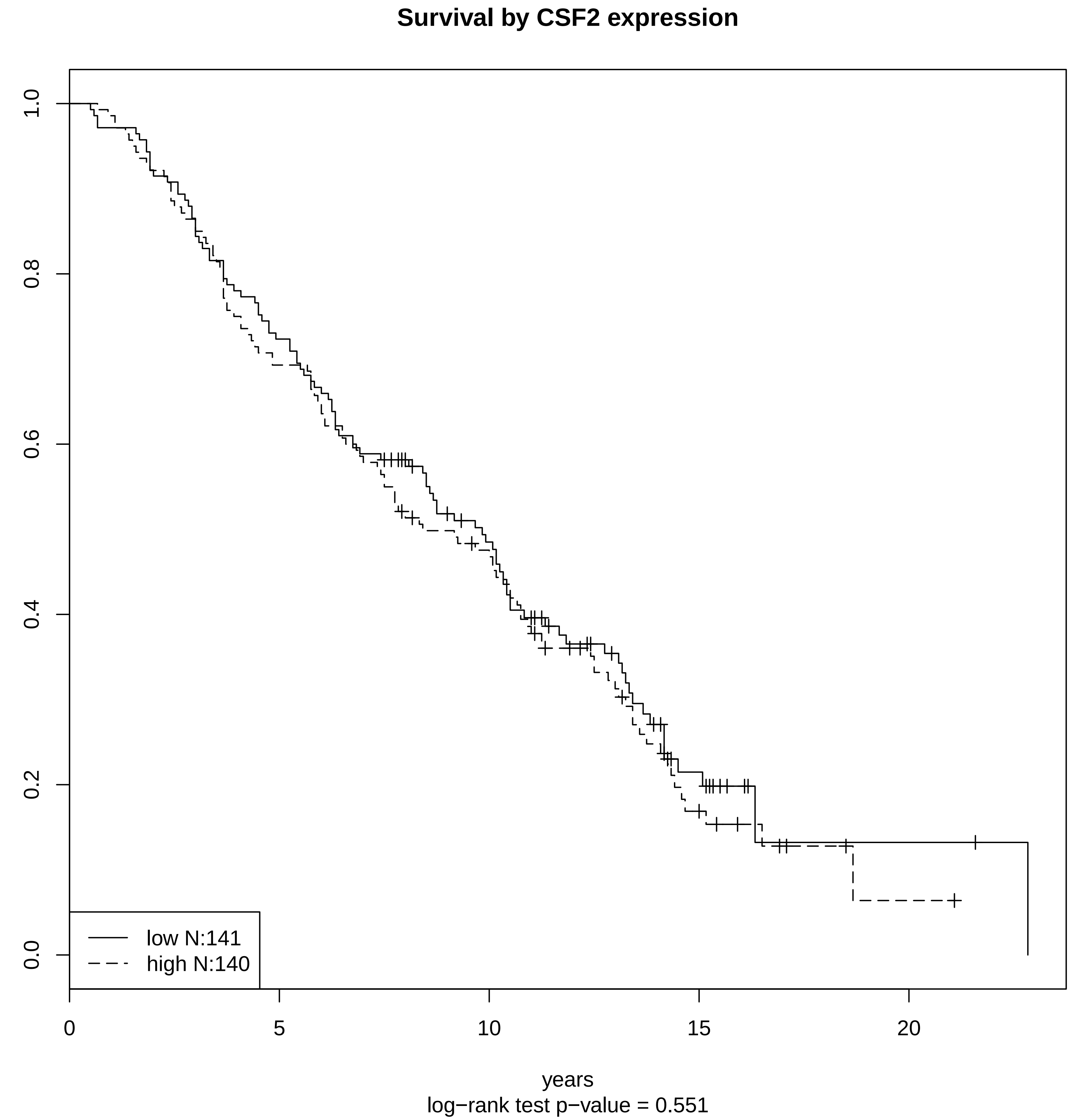
Survival by CSF2 expression
1.0
0.8
0.6
0.4
0.2
0.0
0
5
10
15
20
years
log−rank test p−value = 0.551
low N:141
high N:140

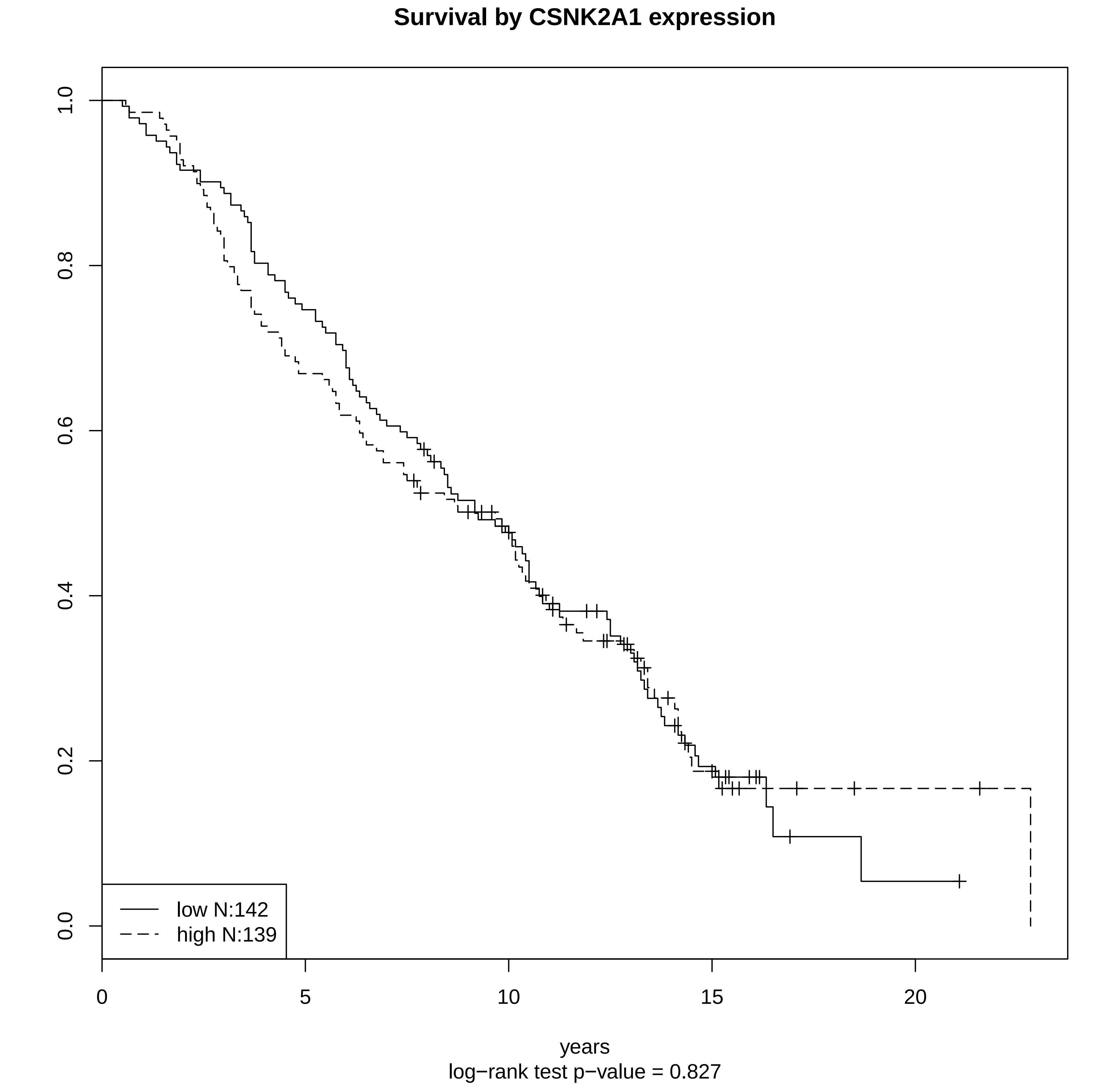
Survival by CSNK2A1 expression
1.0
0.8
0.6
0.4
0.2
0.0
0
5
10
15
20
years
log−rank test p−value = 0.827
low N:142
high N:139

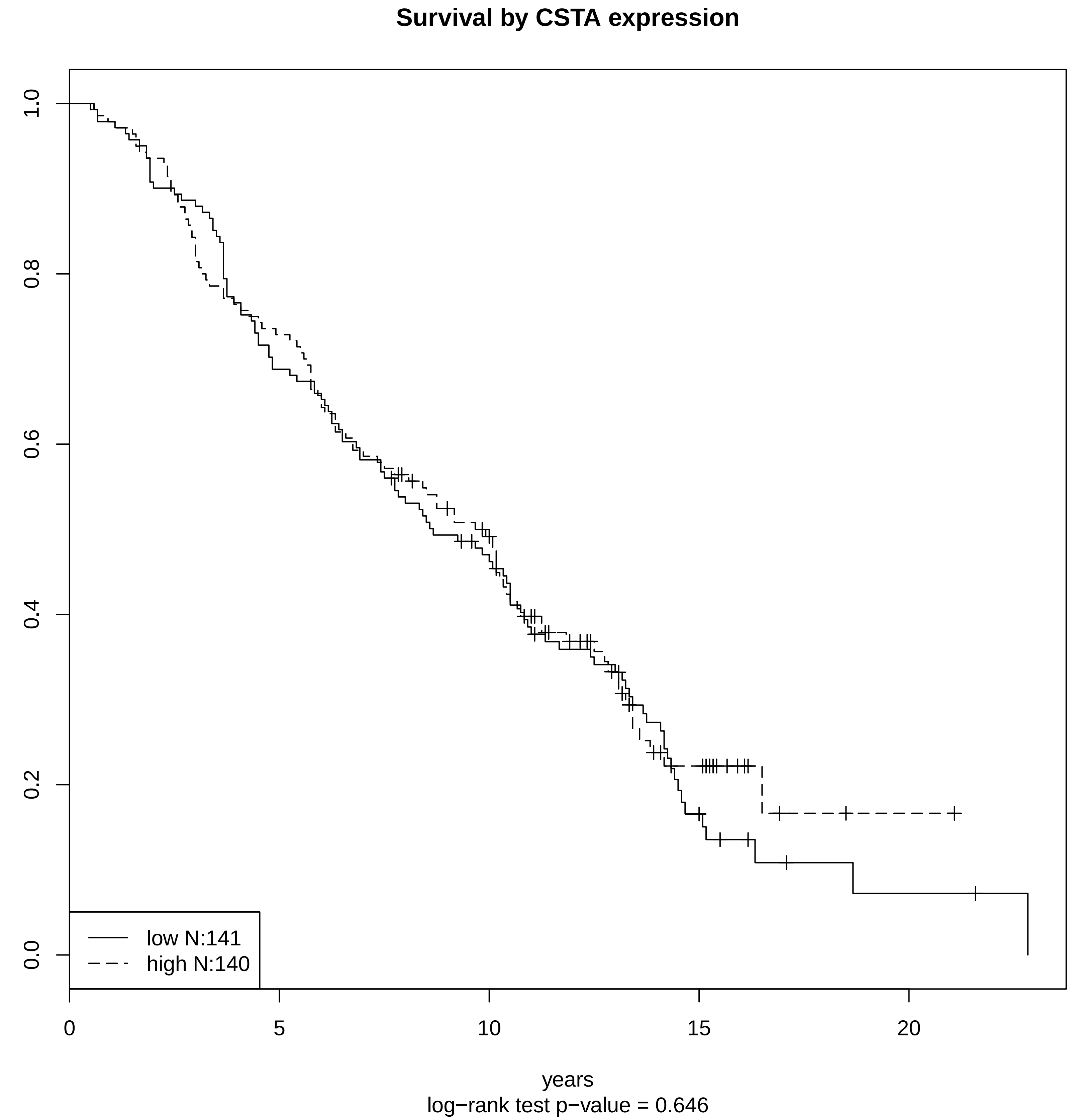
Survival by CSTA expression
1.0
0.8
0.6
0.4
0.2
0.0
0
5
10
15
20
low N:141
high N:140
years
log−rank test p−value = 0.646

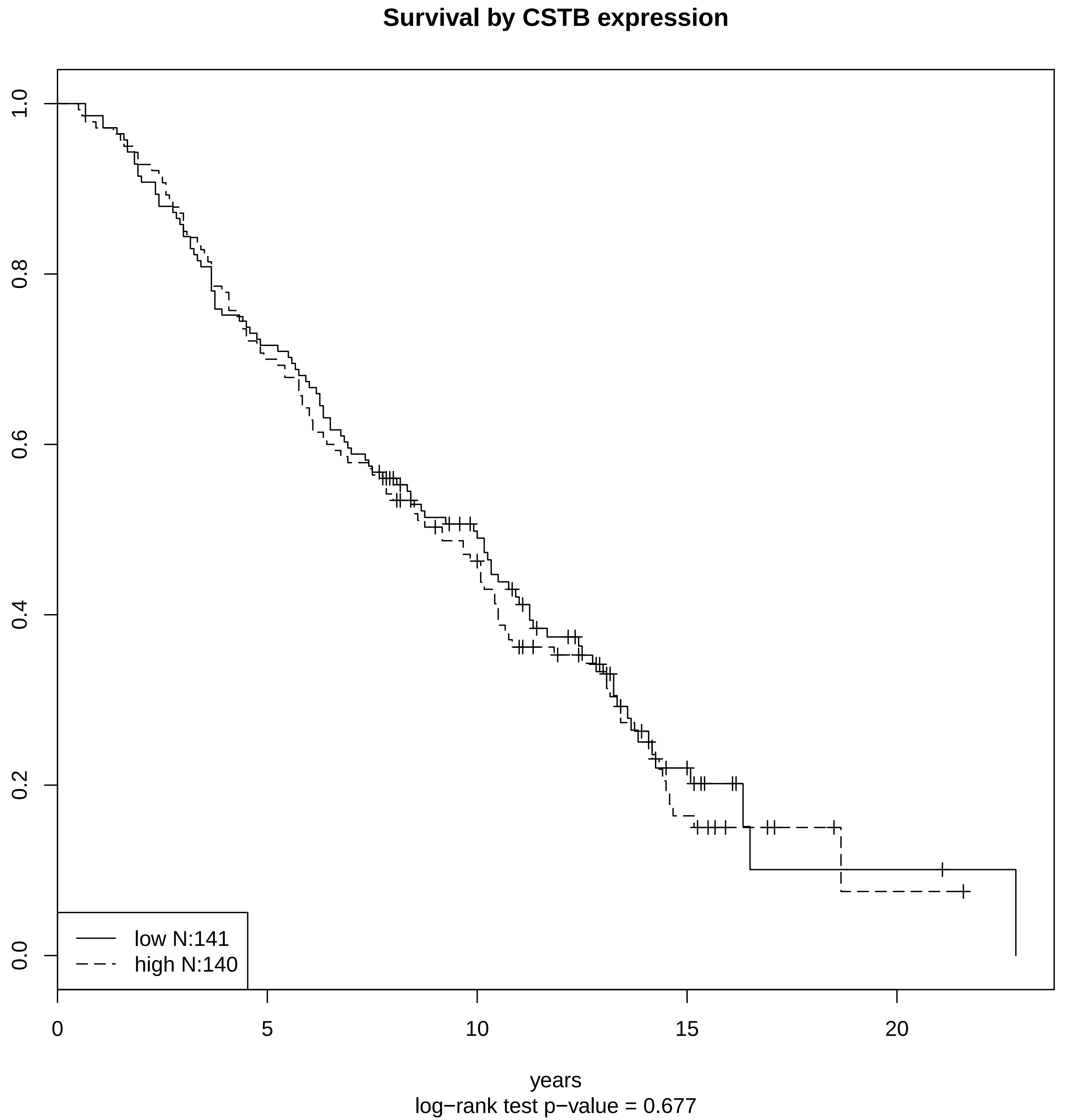
Survival by CSTB expression
1.0
0.8
0.6
0.4
0.2
0.0
0
5
10
15
20
years
log−rank test p−value = 0.677
low N:141
high N:140

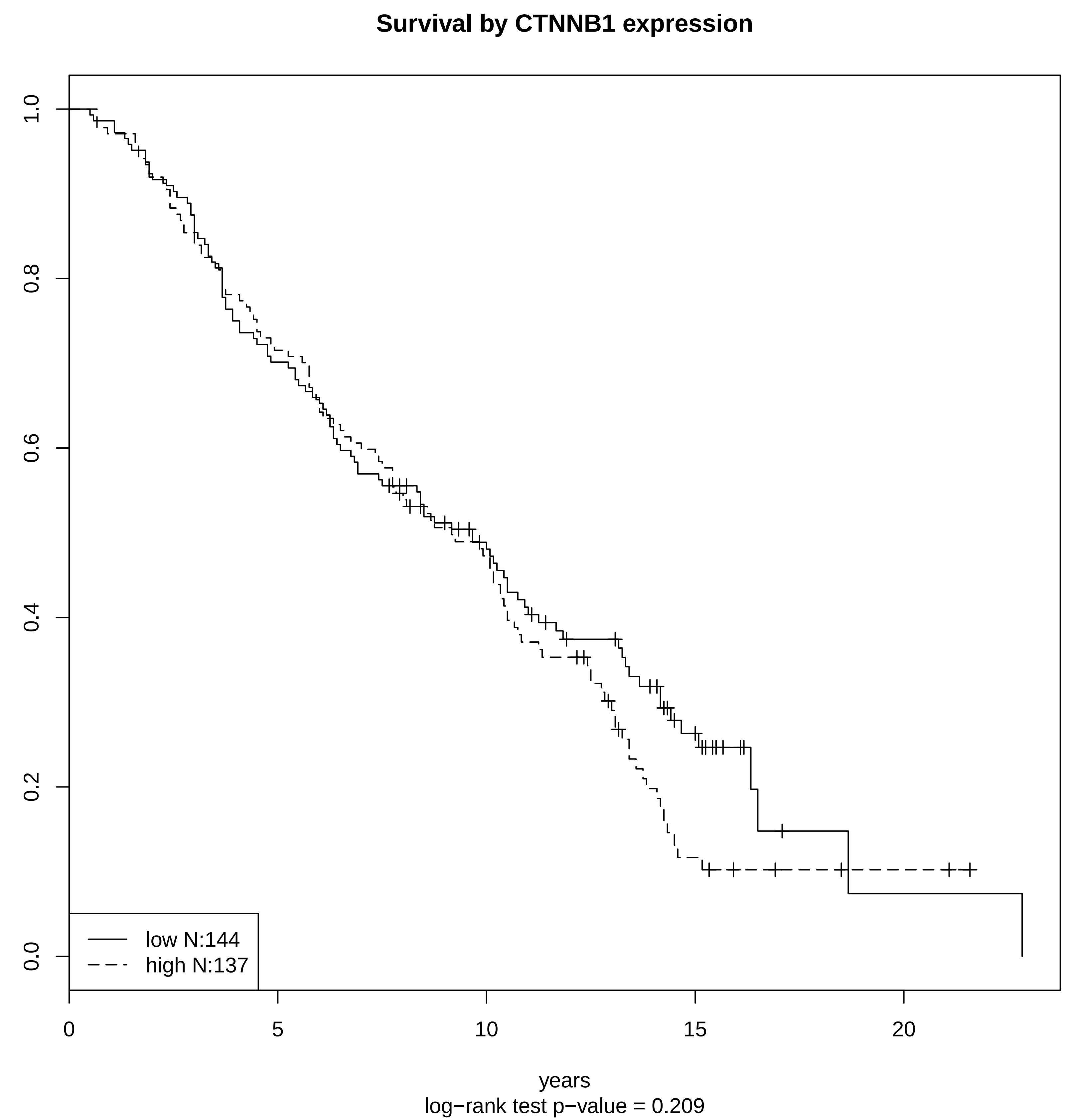

Survival by CTNNB1 expression
1.0
0.8
0.6
0.4
0.2
0.0
0
5
10
15
20
years
log−rank test p−value = 0.209
low N:144
high N:137

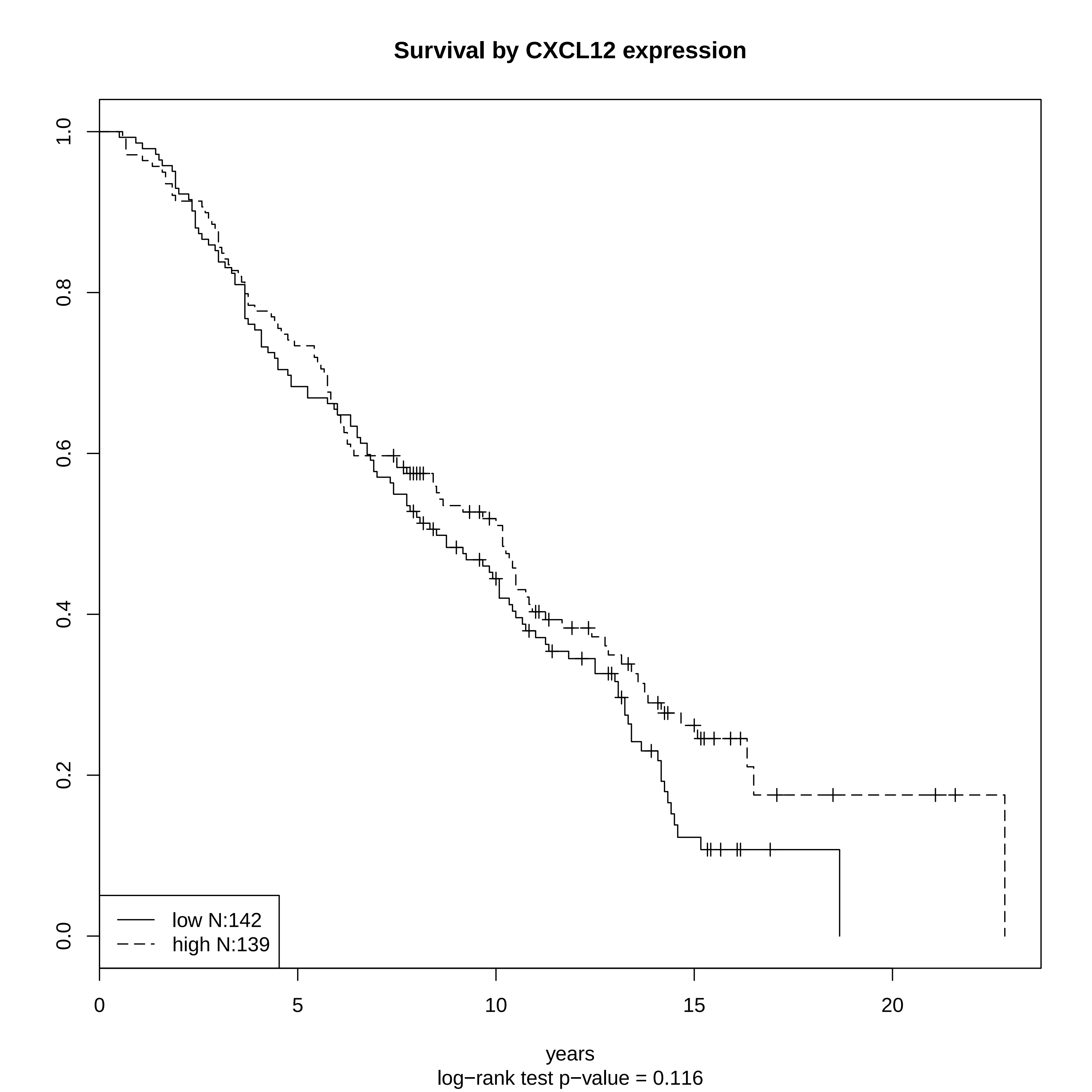
Survival by CXCL12 expression
1.0
0.8
0.6
0.4
0.2
0.0
0
5
10
15
20
low N:142
high N:139
years
log−rank test p−value = 0.116

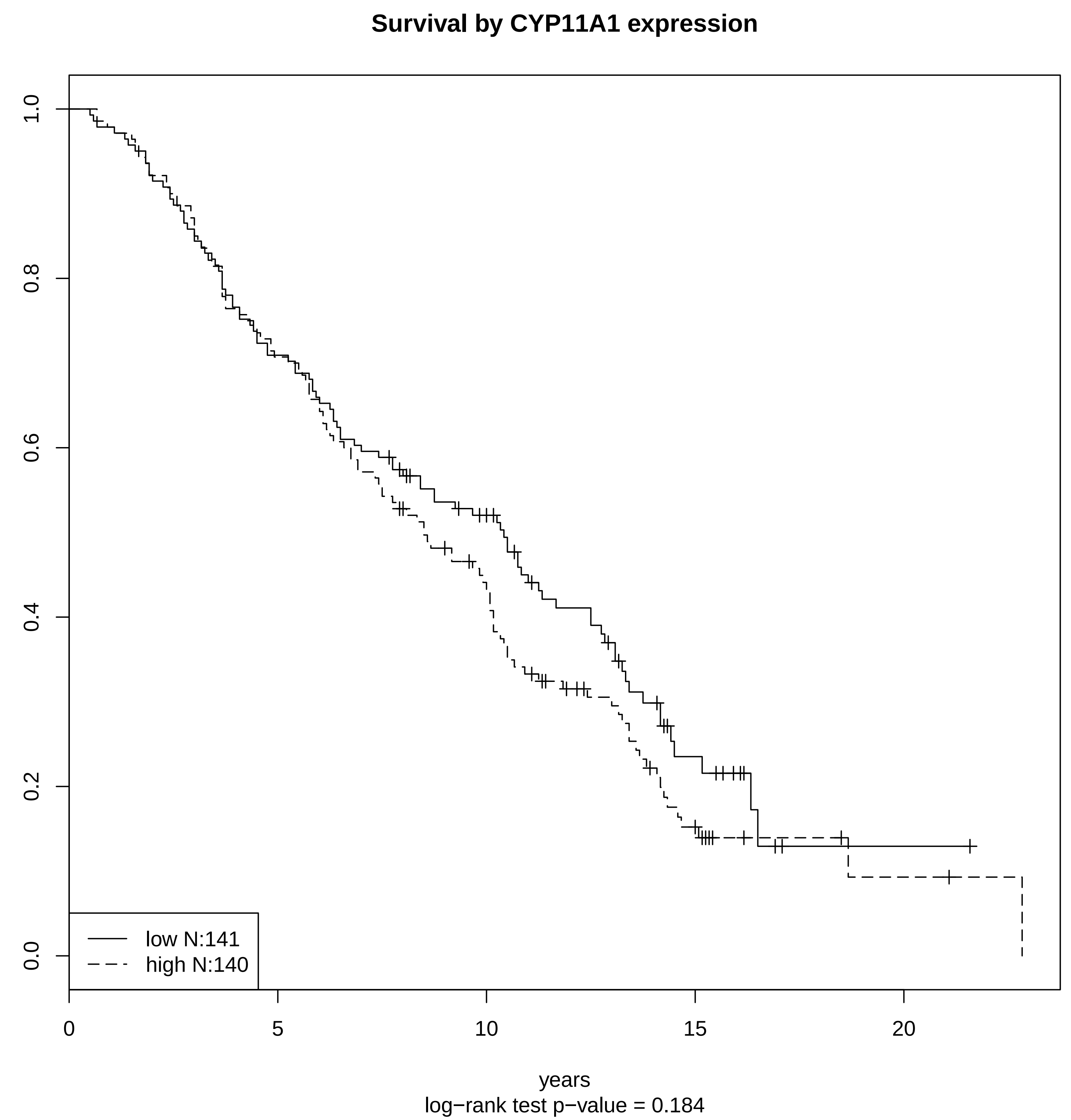
Survival by CYP11A1 expression
1.0
0.8
0.6
0.4
0.2
0.0
0
5
10
15
20
years
log−rank test p−value = 0.184
low N:141
high N:140

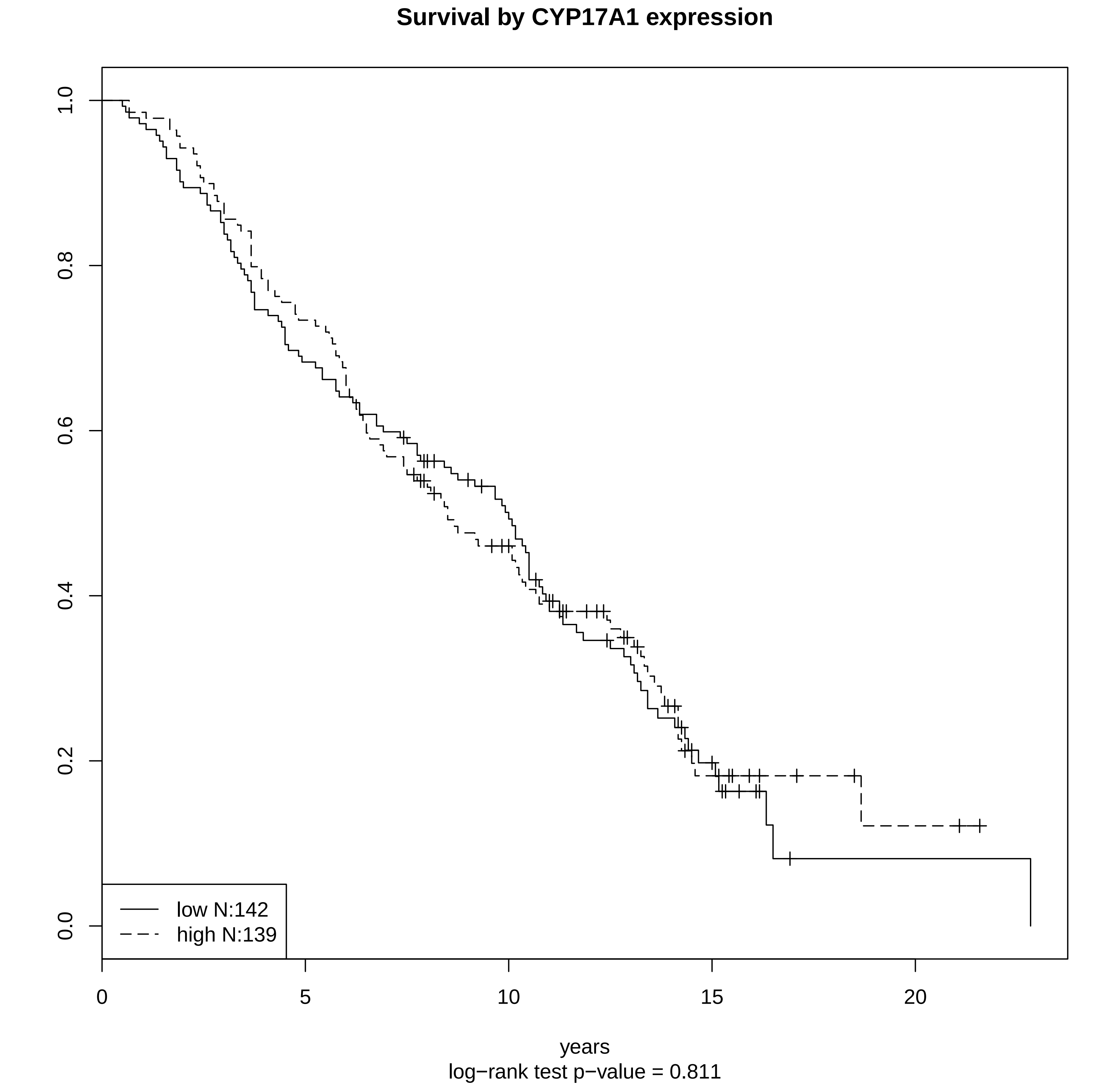
Survival by CYP17A1 expression
0.0
0.2
0.4
0.6
0.8
1.0
0
5
10
15
20
low N:142
high N:139
years
log−rank test p−value = 0.811

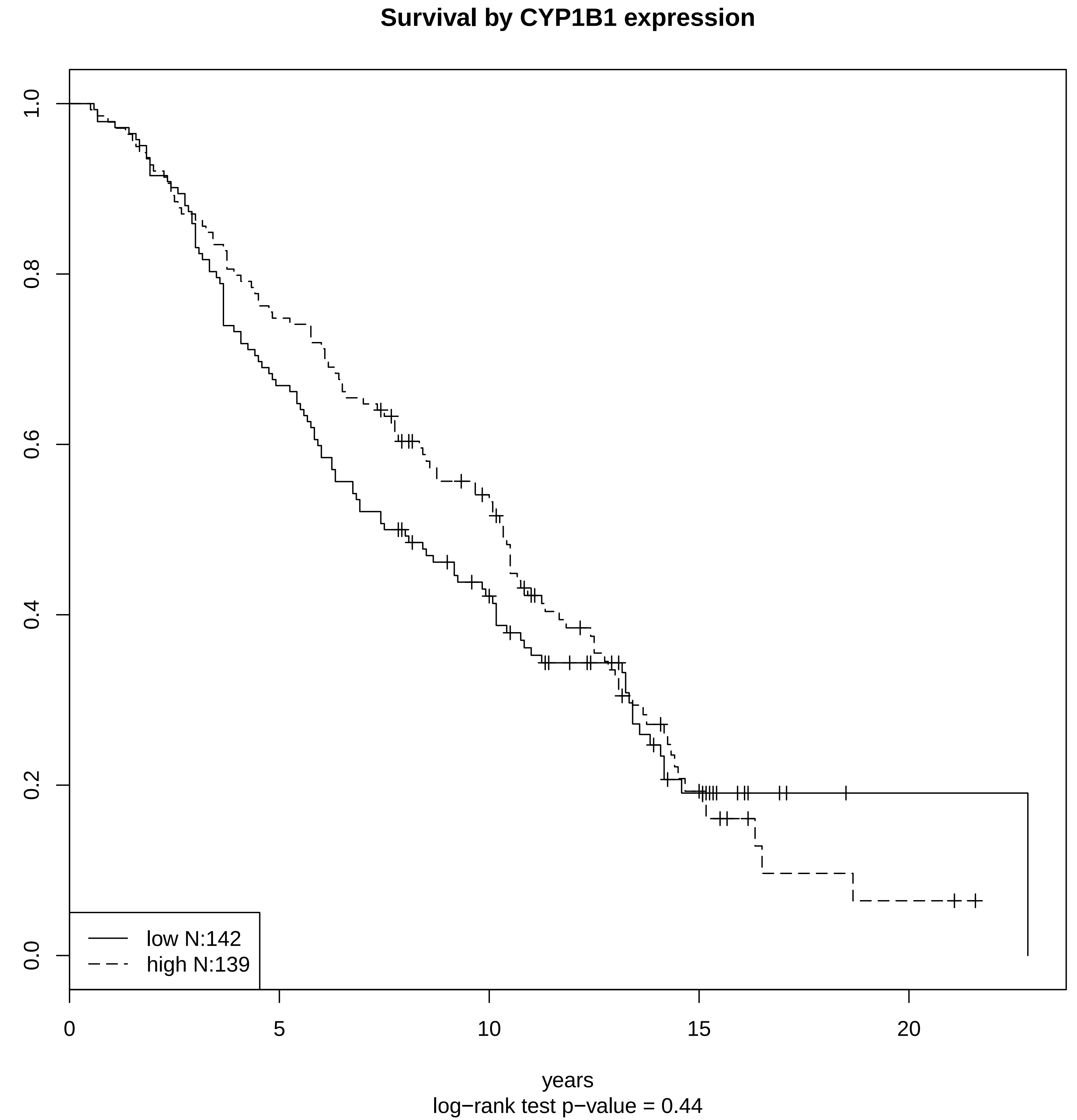

Survival by CYP1B1 expression
1.0
0.8
0.6
0.4
0.2
0.0
0
5
10
15
20
low N:142
high N:139
years
log−rank test p−value = 0.44

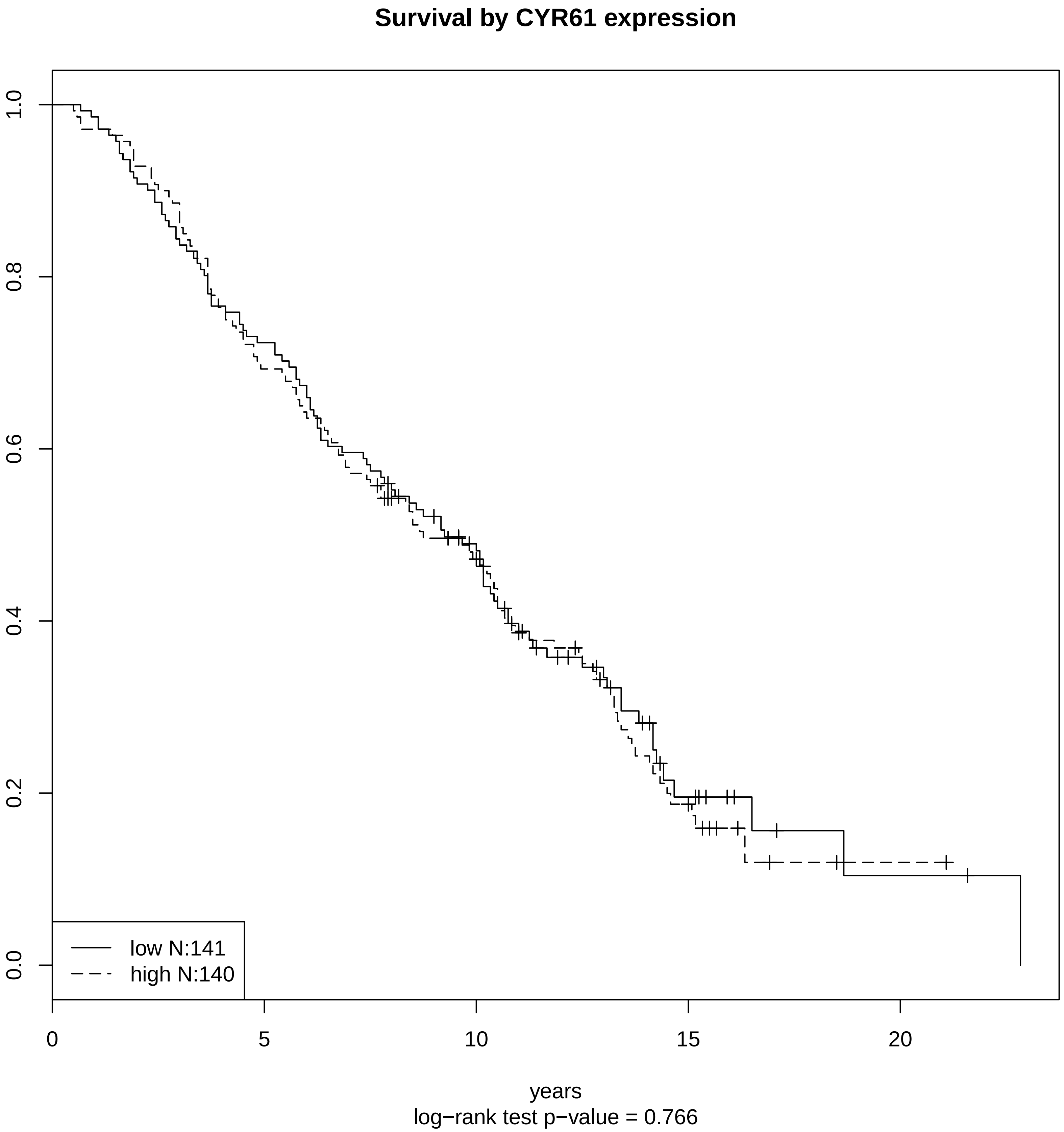

Survival by CYR61 expression
1.0
0.8
0.6
0.4
0.2
0.0
0
5
10
15
20
low N:141
high N:140
years
log−rank test p−value = 0.766

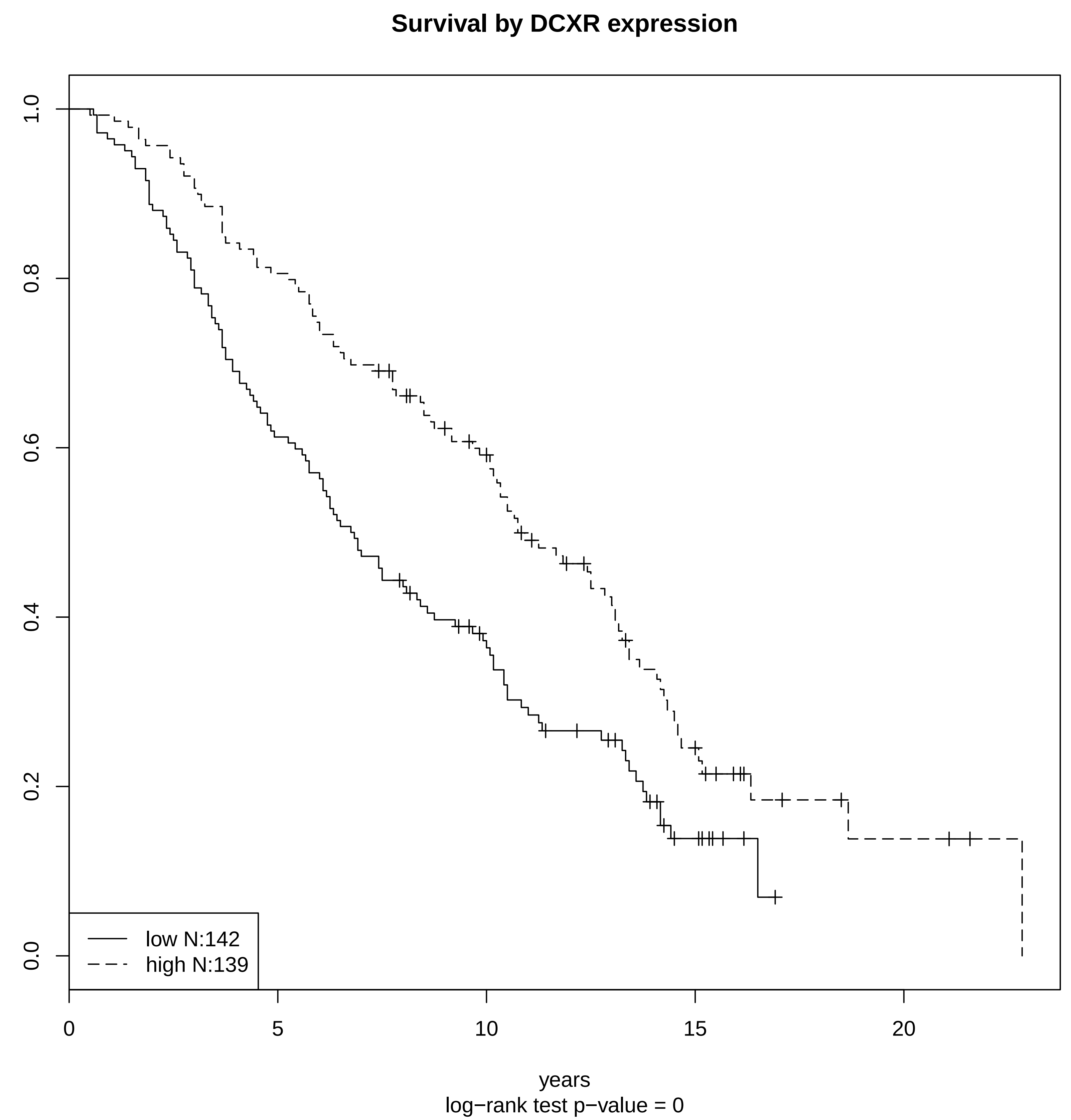
Survival by DCXR expression
1.0
0.8
0.6
0.4
0.2
0.0
0
5
10
15
20
low N:142
high N:139
years
log−rank test p−value = 0

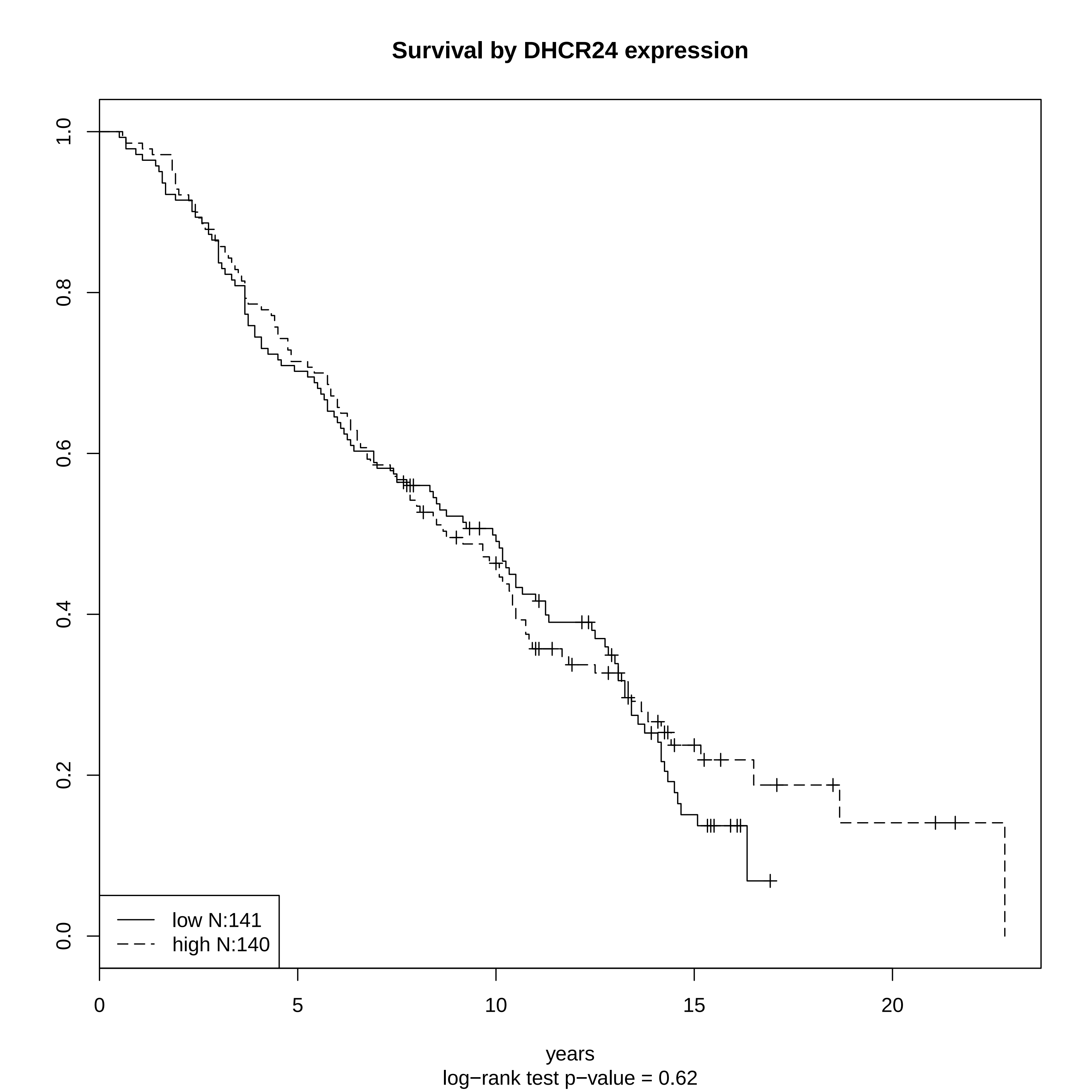
Survival by DHCR24 expression
1.0
0.8
0.6
0.4
0.2
0.0
low N:141
high N:140
0
5
10
15
20
years
log−rank test p−value = 0.62

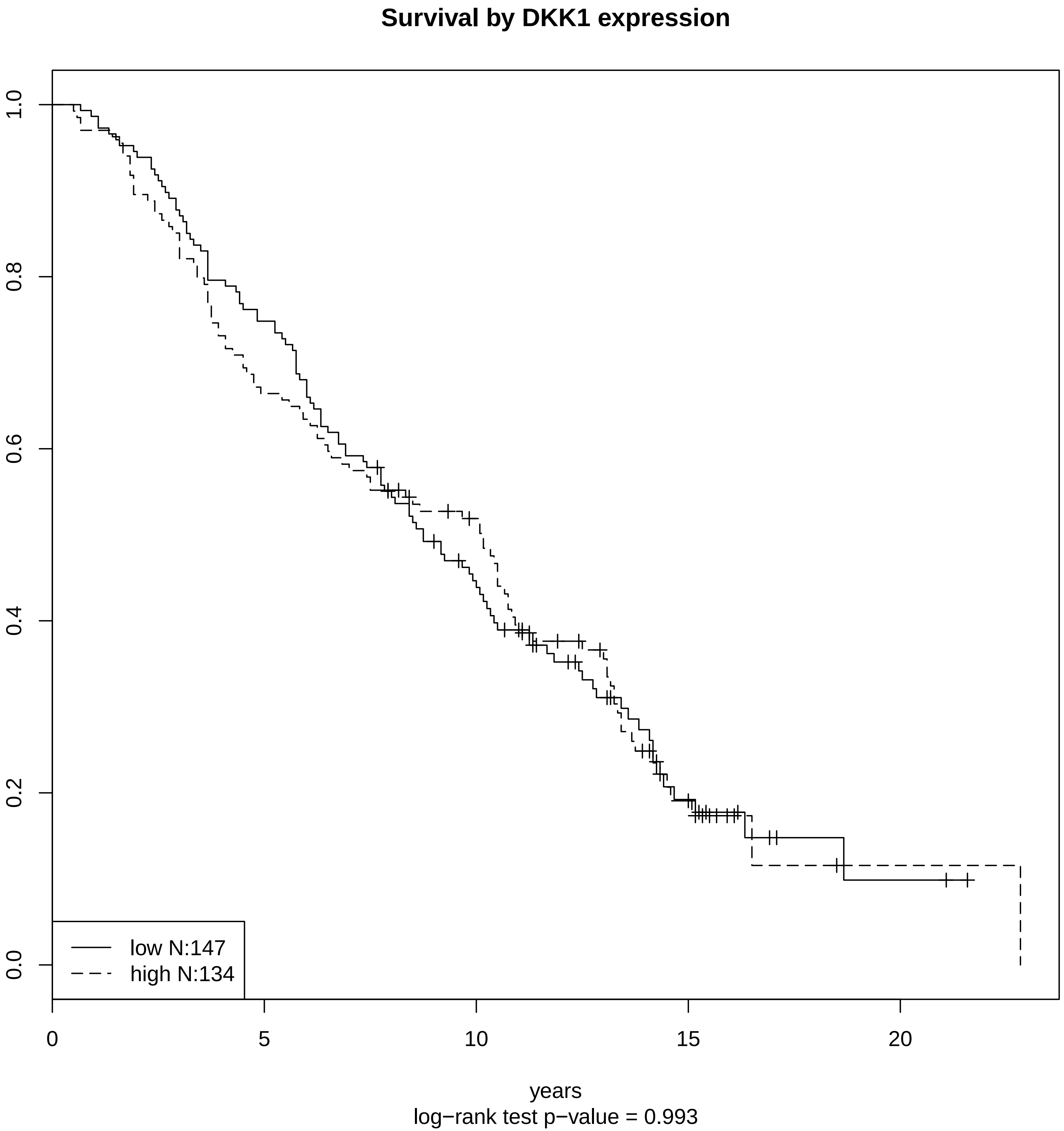

Survival by DKK1 expression
1.0
0.8
0.6
0.4
0.2
0.0
0
5
10
15
20
years
low N:147
high N:134
log−rank test p−value = 0.993

Survival by DPP4 expression

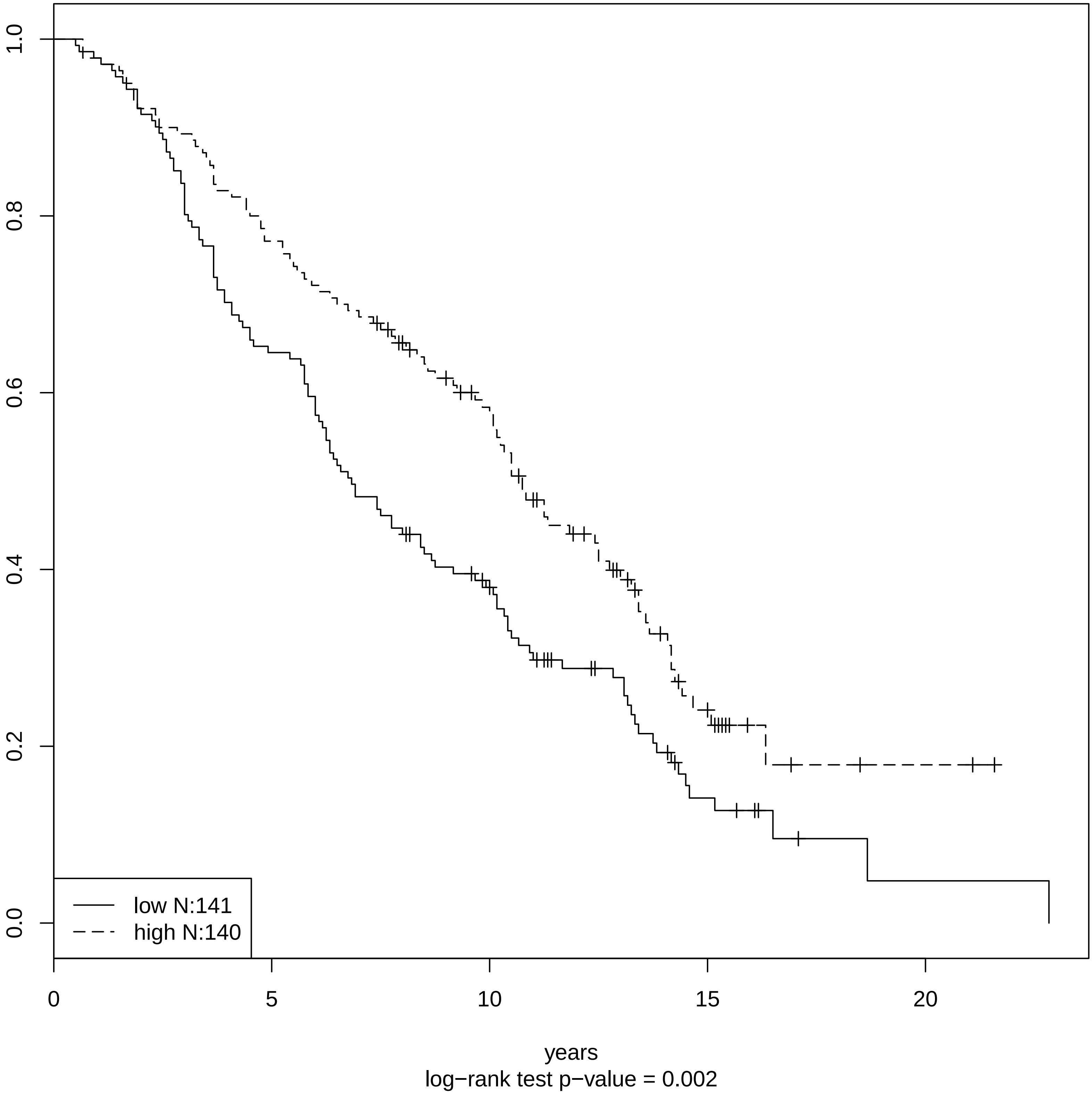

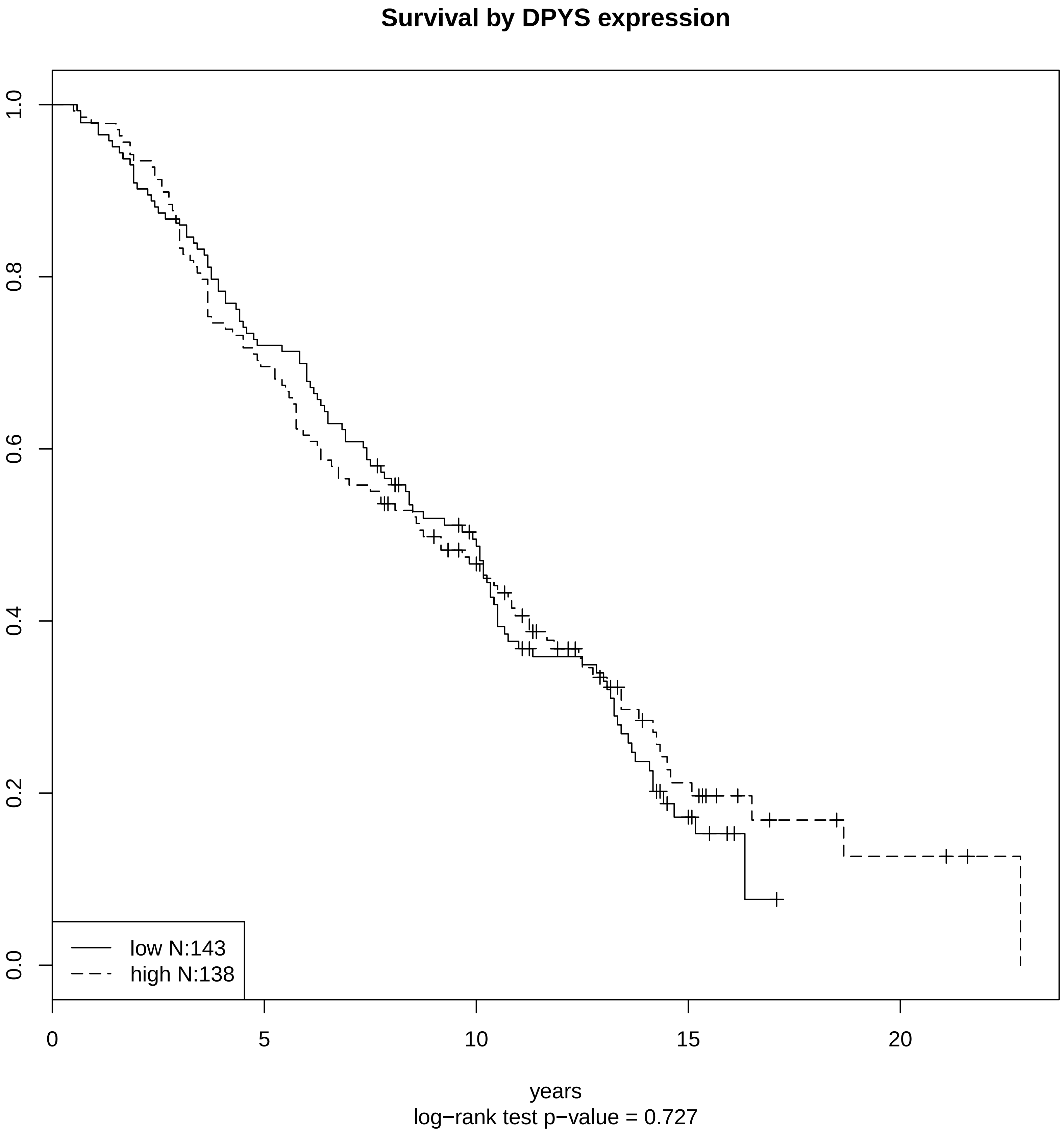

Survival by DPYS expression
1.0
0.8
0.6
0.4
0.2
0.0
0
5
10
15
20
low N:143
high N:138
years
log−rank test p−value = 0.727

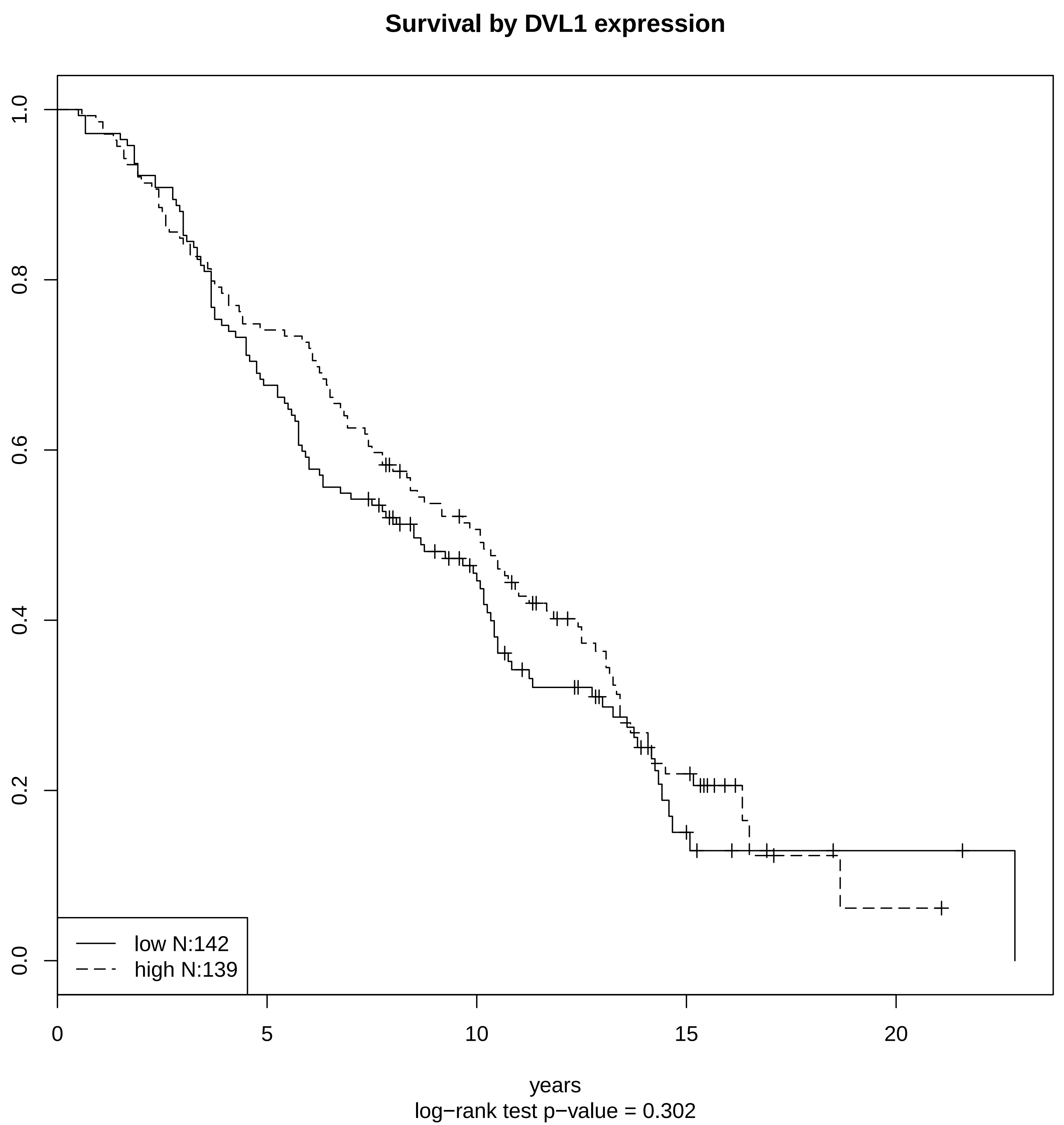
Survival by DVL1 expression
1.0
0.8
0.6
0.4
0.2
0.0
0
5
10
15
20
years
log−rank test p−value = 0.302
low N:142
high N:139

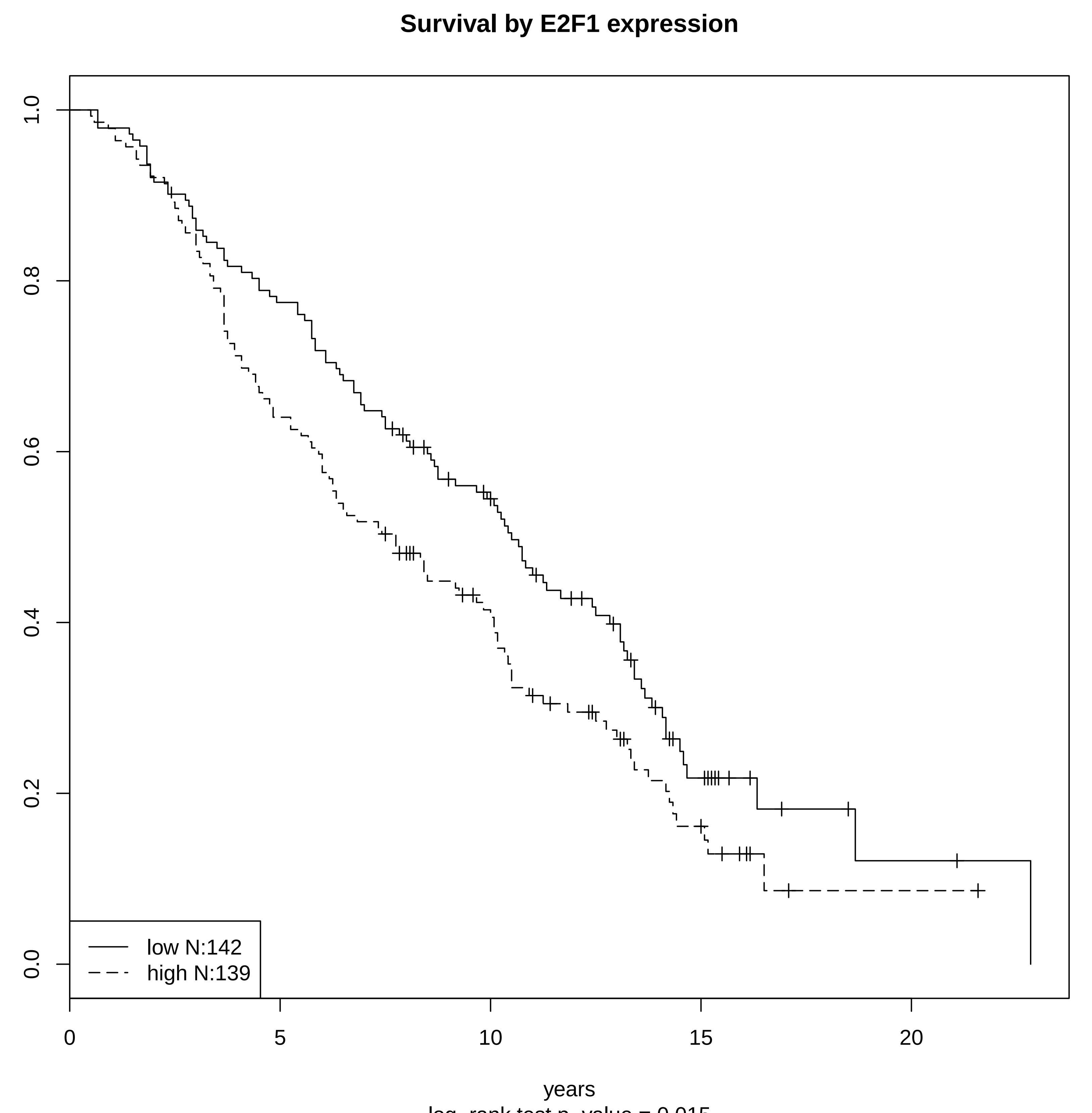
Survival by E2F1 expression
1.0
0.8
0.6
0.4
0.2
0.0
0
5
10
15
20
low N:142
high N:139
years
log−rank test p−value = 0.015

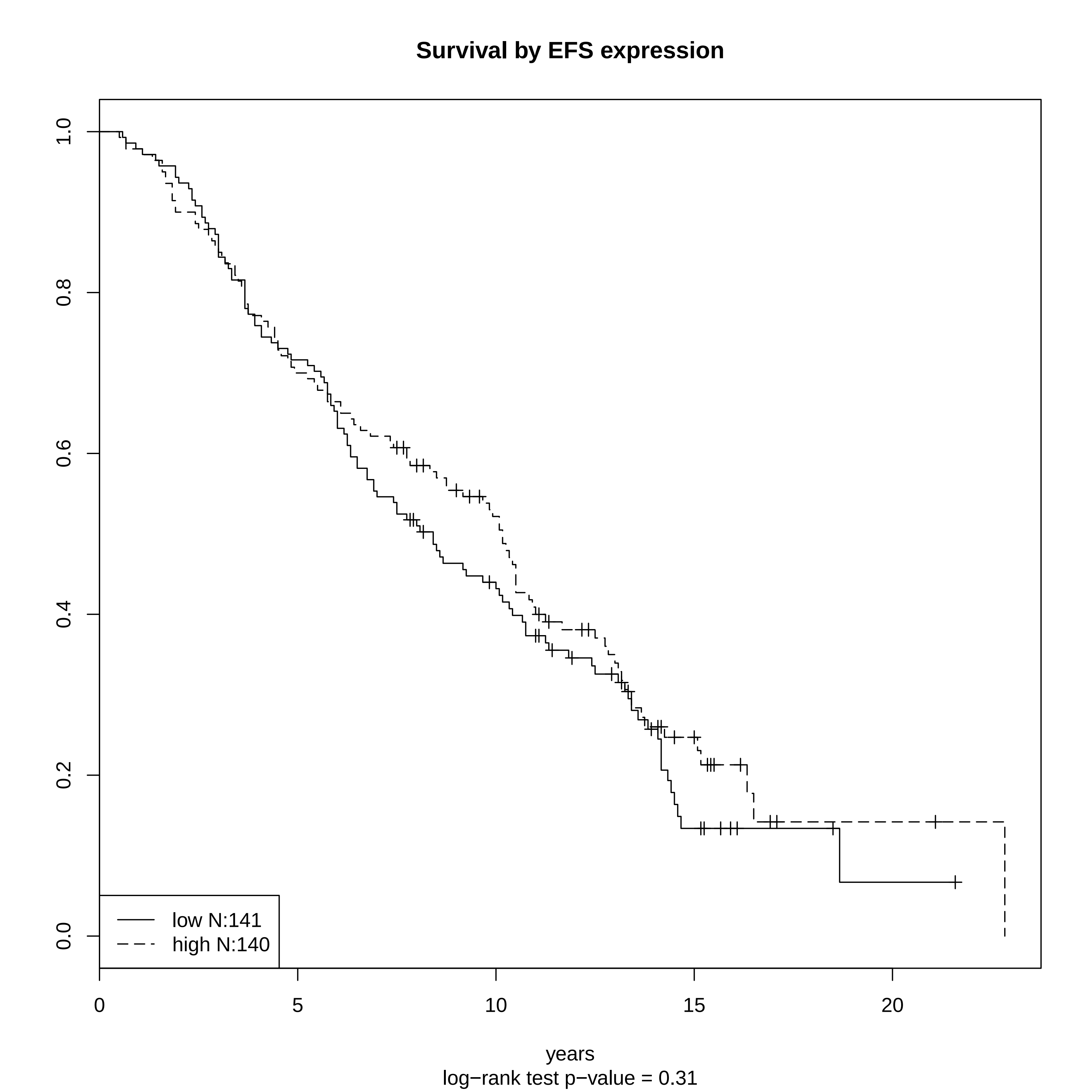
Survival by EFS expression
1.0
0.8
0.6
0.4
0.2
0.0
0
5
10
15
20
low N:141
high N:140
years
log−rank test p−value = 0.31

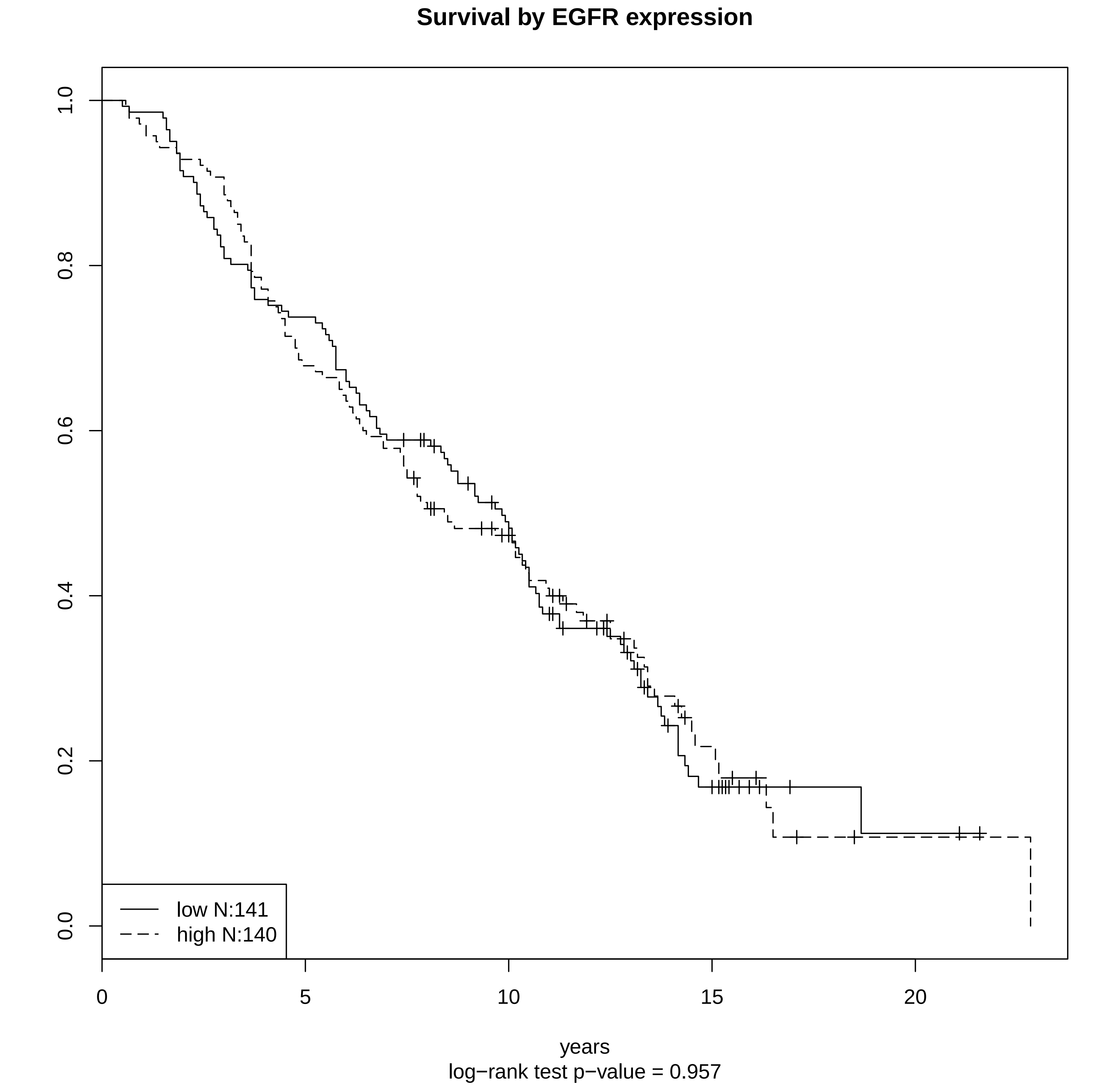
Survival by EGFR expression
1.0
0.8
0.6
0.4
0.2
0.0
0
5
10
15
20
years
log−rank test p−value = 0.957
low N:141
high N:140

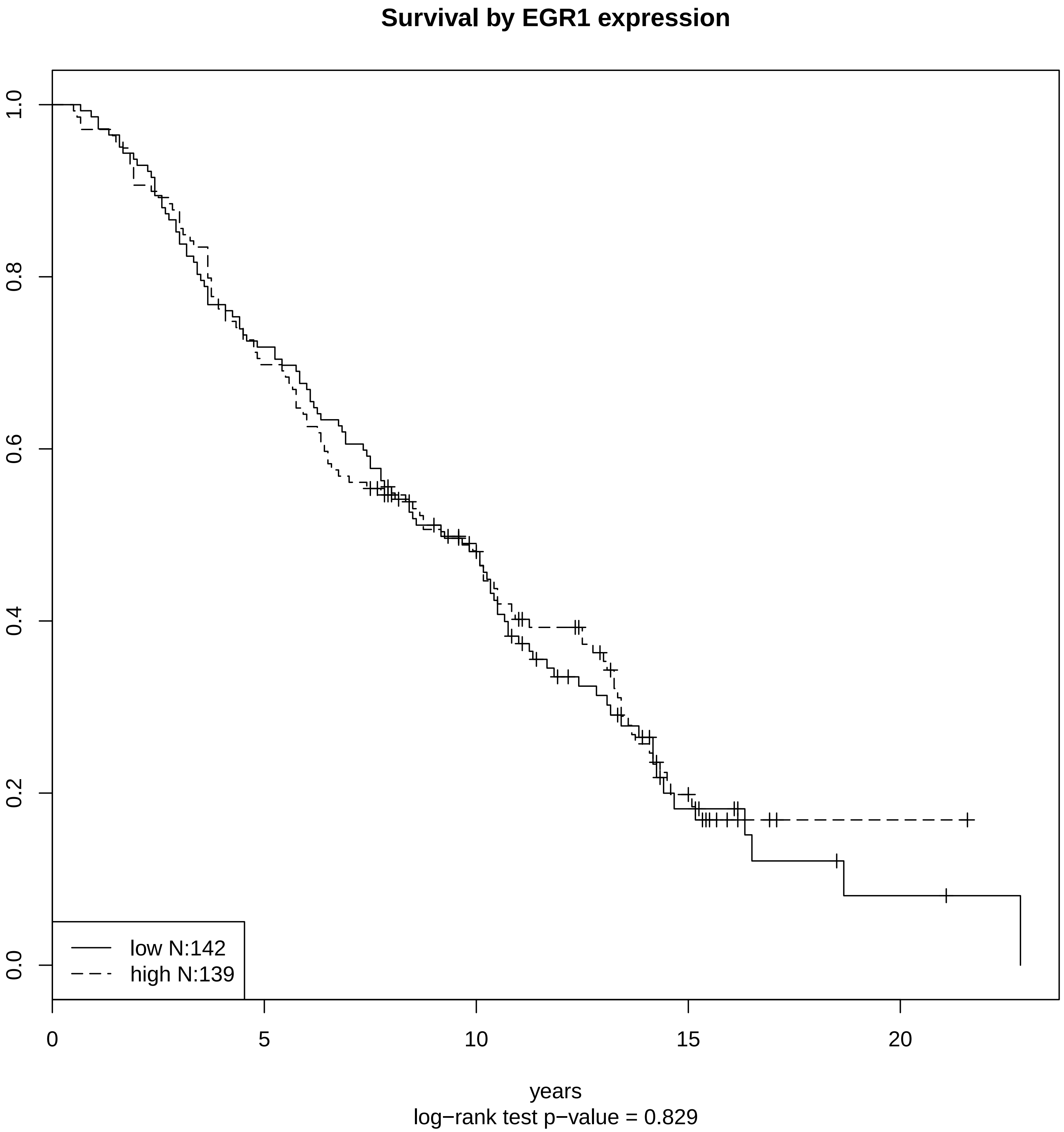

Survival by EGR1 expression
1.0
0.8
0.6
0.4
0.2
0.0
0
5
10
15
20
low N:142
high N:139
years
log−rank test p−value = 0.829

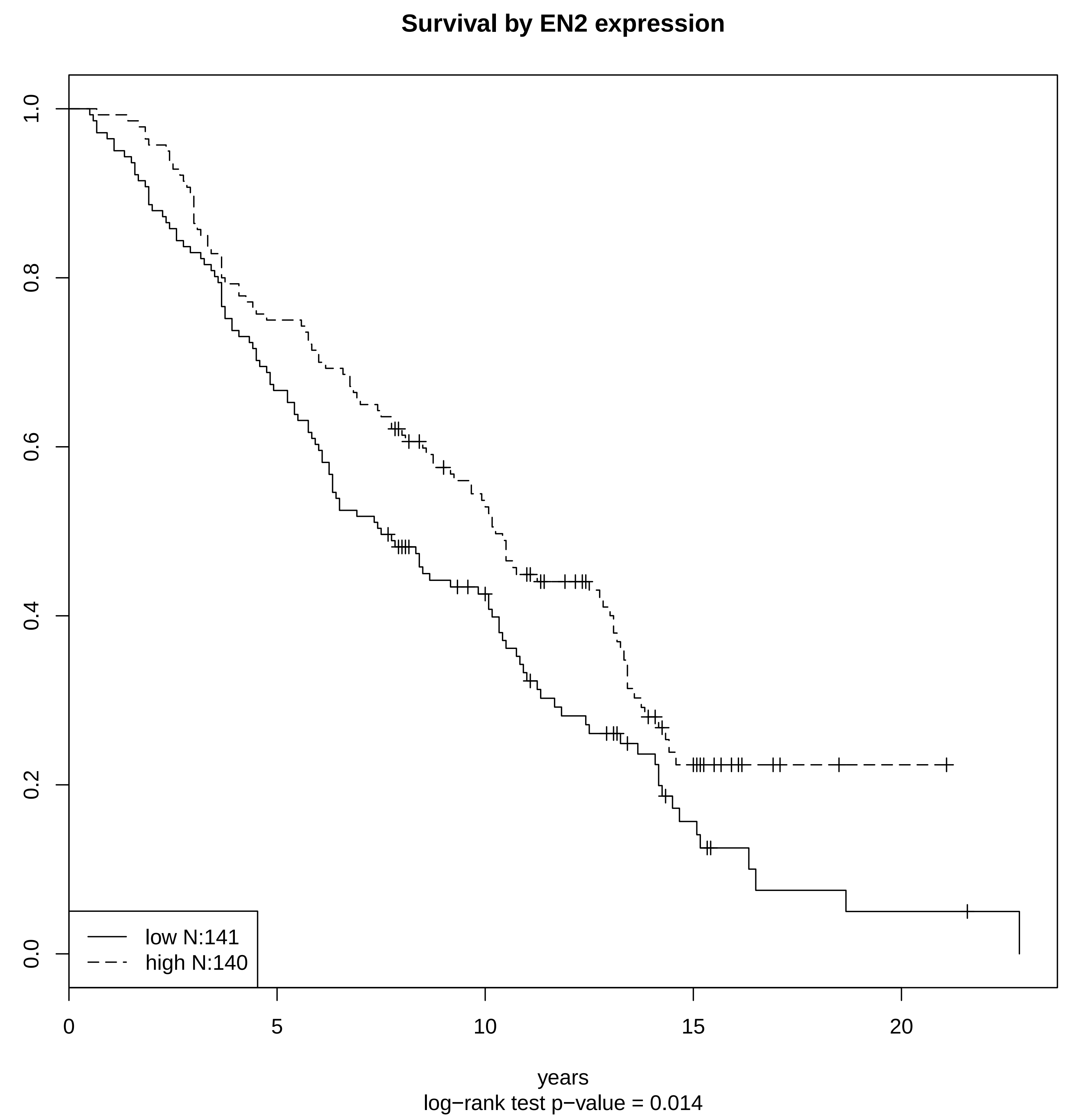
Survival by EN2 expression
1.0
0.8
0.6
0.4
0.2
0.0
0
5
10
15
20
low N:141
high N:140
years
log−rank test p−value = 0.014

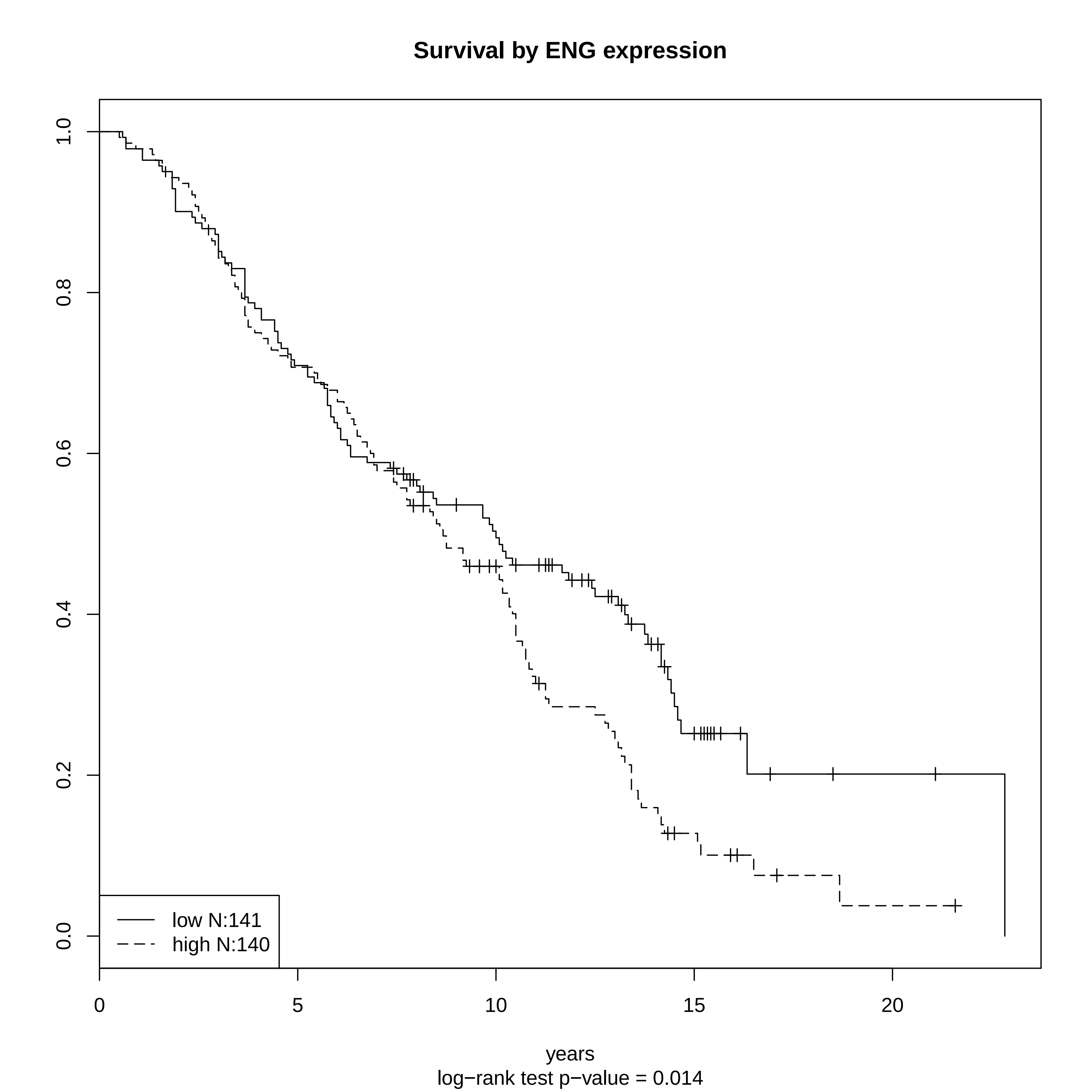
Survival by ENG expression
1.0
0.8
0.6
0.4
0.2
0.0
0
5
10
15
20
low N:141
high N:140
years
log−rank test p−value = 0.014

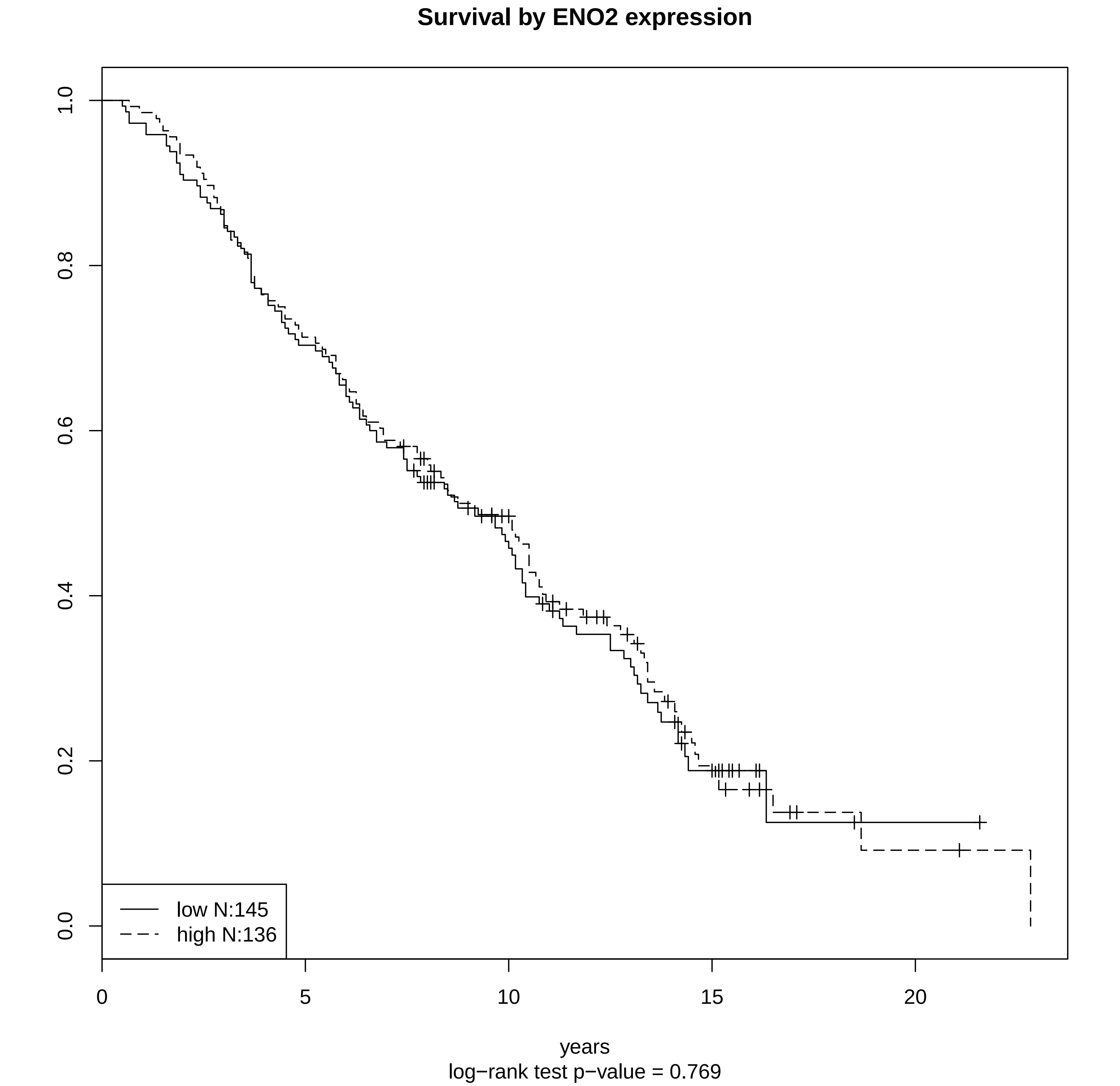
Survival by ENO2 expression
1.0
0.8
0.6
0.4
0.2
0.0
0
5
10
15
20
years
log−rank test p−value = 0.769
low N:145
high N:136

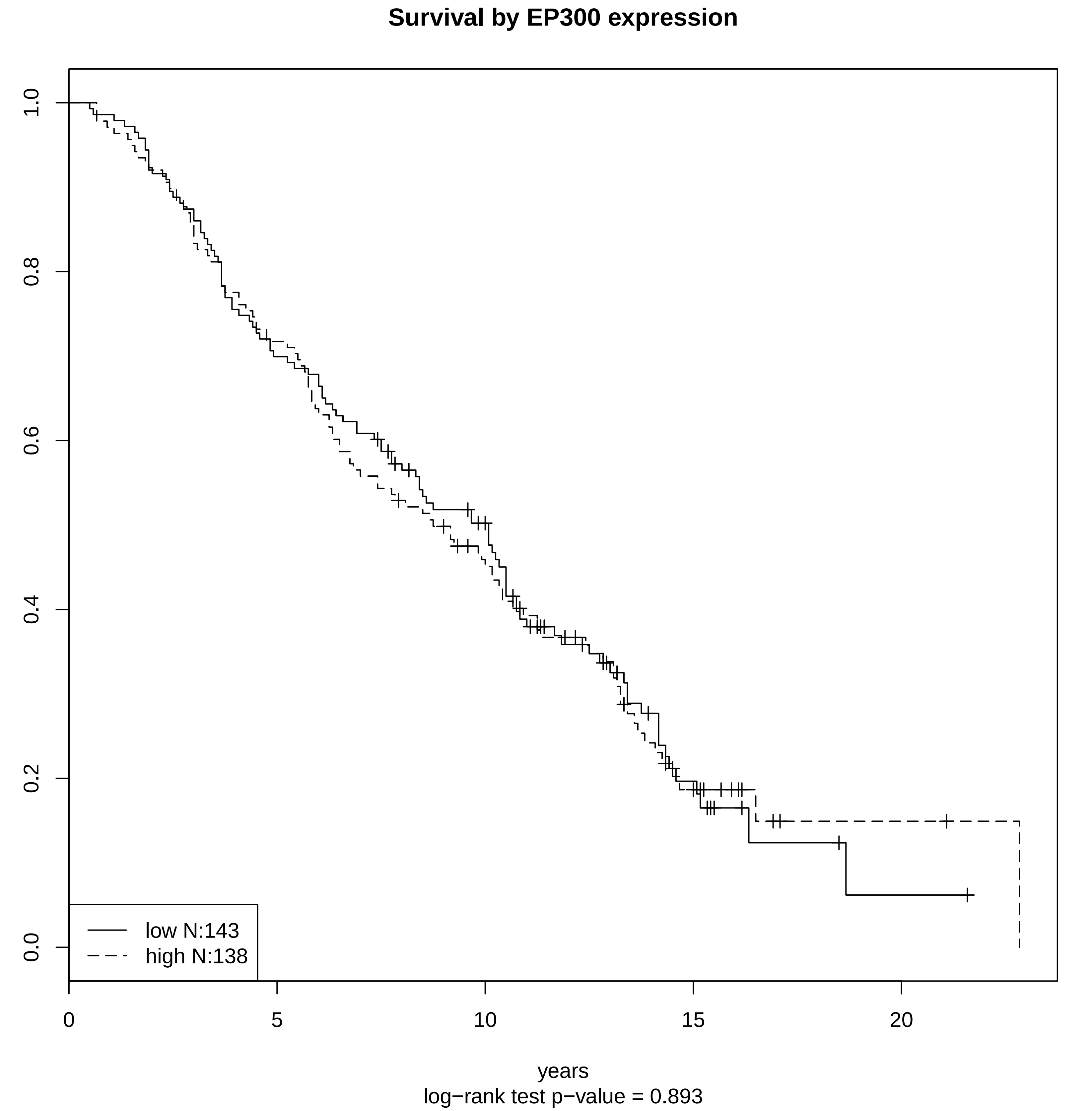

Survival by EP300 expression
1.0
0.8
0.6
0.4
0.2
0.0
low N:143
high N:138
0
5
10
15
20
years
log−rank test p−value = 0.893

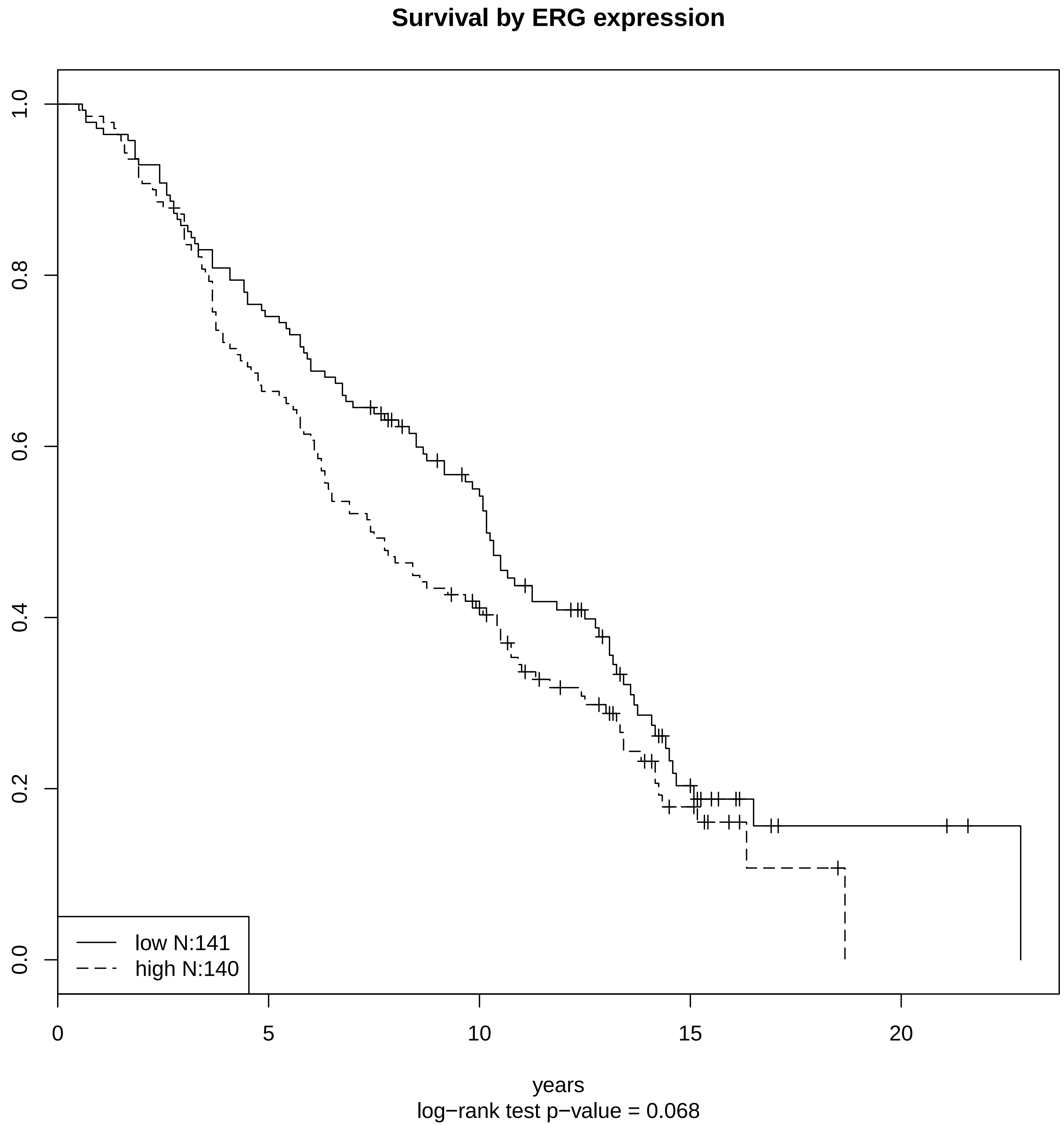

Survival by ERG expression
1.0
0.8
0.6
0.4
0.2
0.0
0
5
10
15
20
low N:141
high N:140
years
log−rank test p−value = 0.068

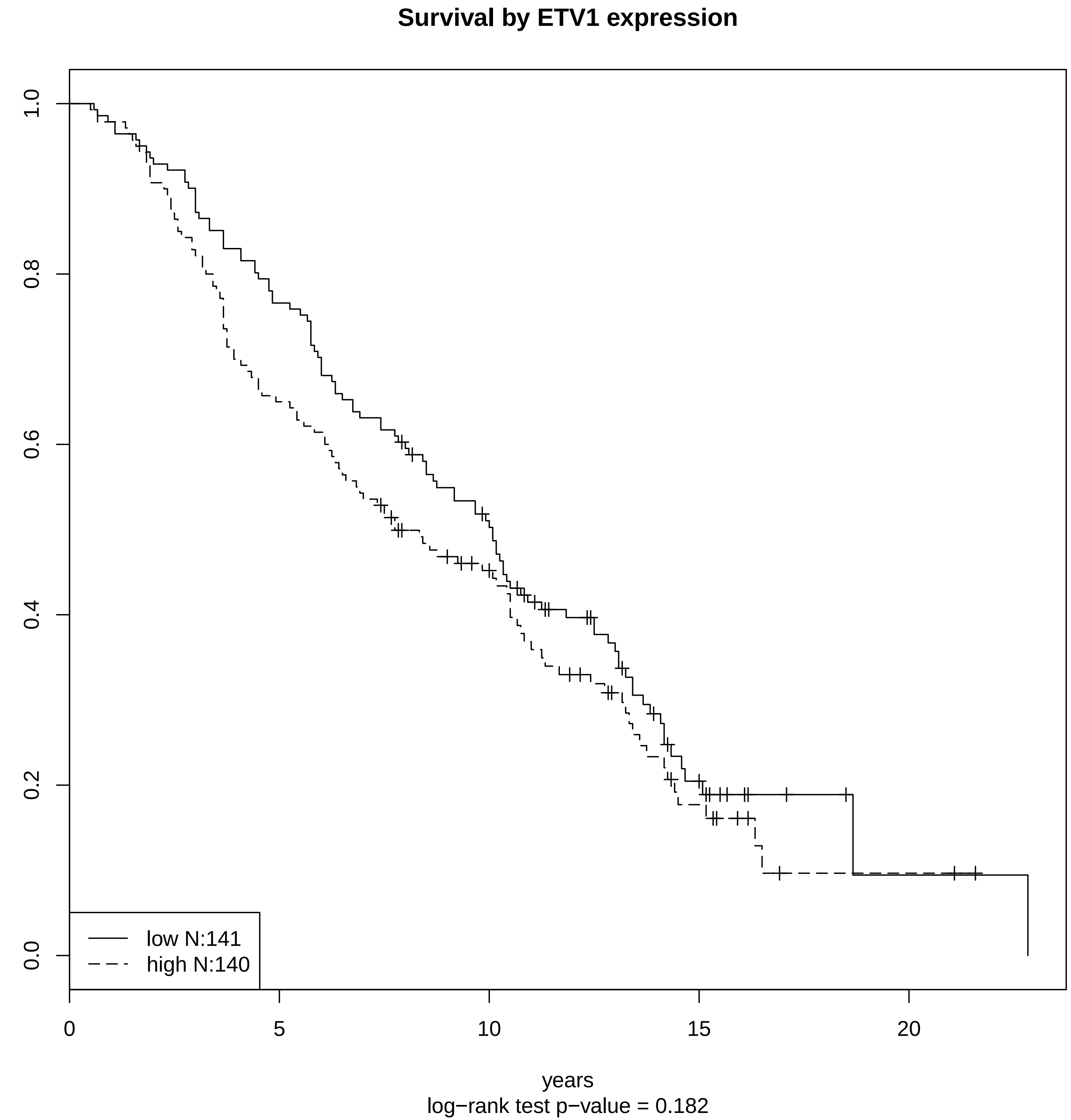

Survival by ETV1 expression
1.0
0.8
0.6
0.4
0.2
0.0
0
5
10
15
20
years
log−rank test p−value = 0.182
low N:141
high N:140

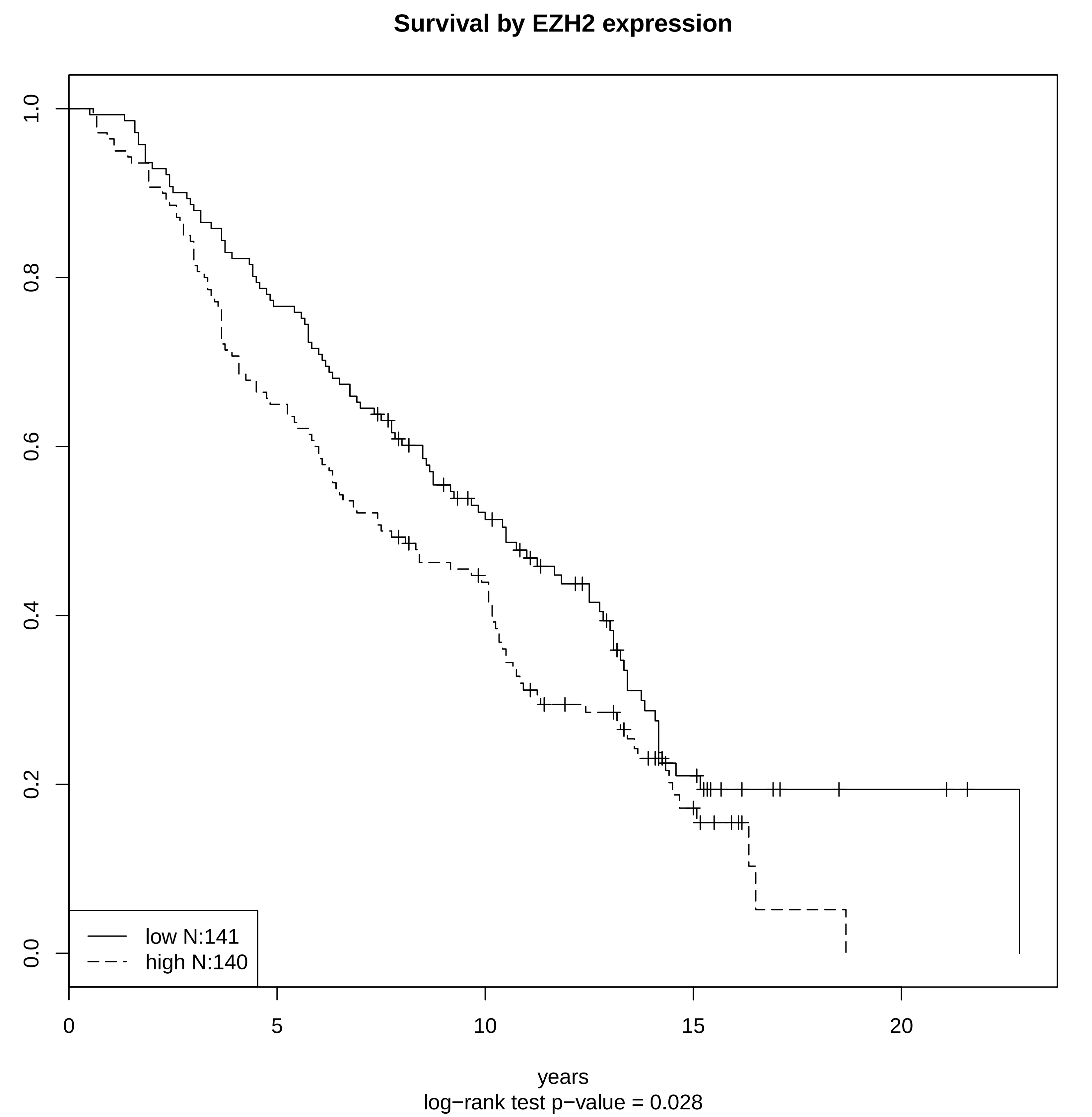
Survival by EZH2 expression
1.0
0.8
0.6
0.4
0.2
0.0
0
5
10
15
20
low N:141
high N:140
years
log−rank test p−value = 0.028

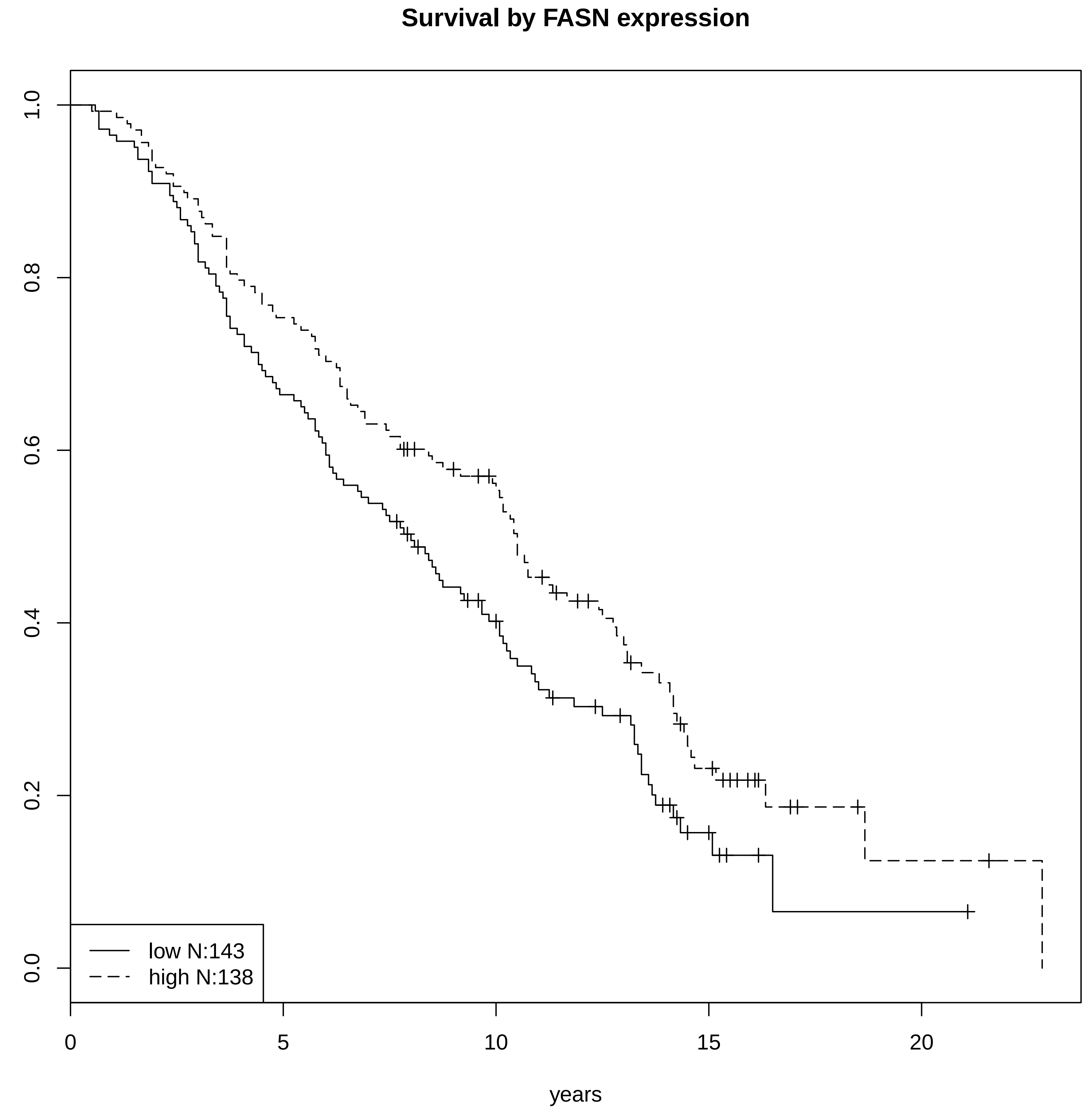
Survival by FASN expression
1.0
0.8
0.6
0.4
0.2
0.0
0
5
10
15
20
years
log−rank test p−value = 0.017
low N:143
high N:138

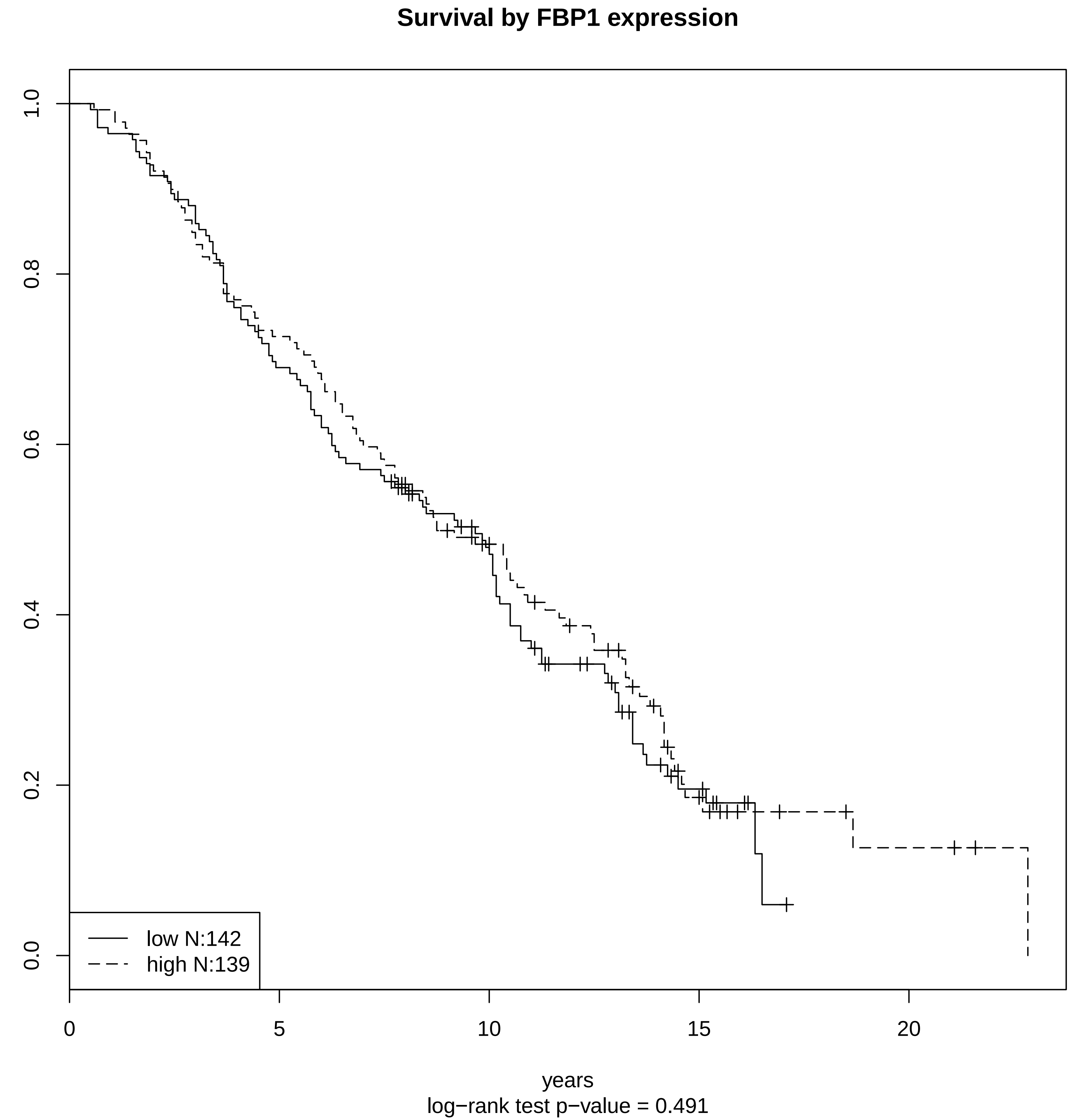
Survival by FBP1 expression
1.0
0.8
0.6
0.4
0.2
0.0
low N:142
high N:139
0
5
10
15
20
years
log−rank test p−value = 0.491

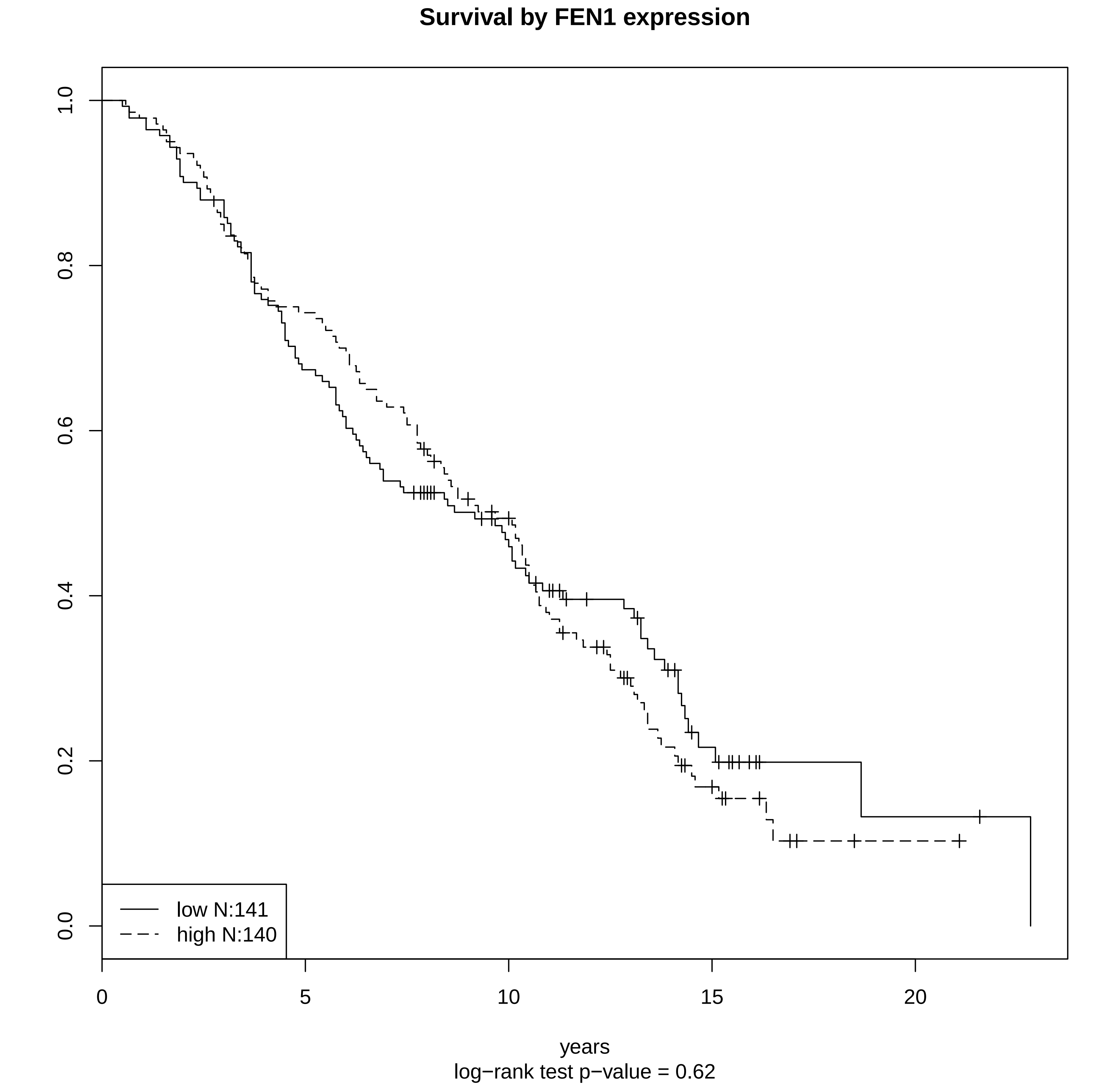
Survival by FEN1 expression
1.0
0.8
0.6
0.4
0.2
0.0
0
5
10
15
20
low N:141
high N:140
years
log−rank test p−value = 0.62

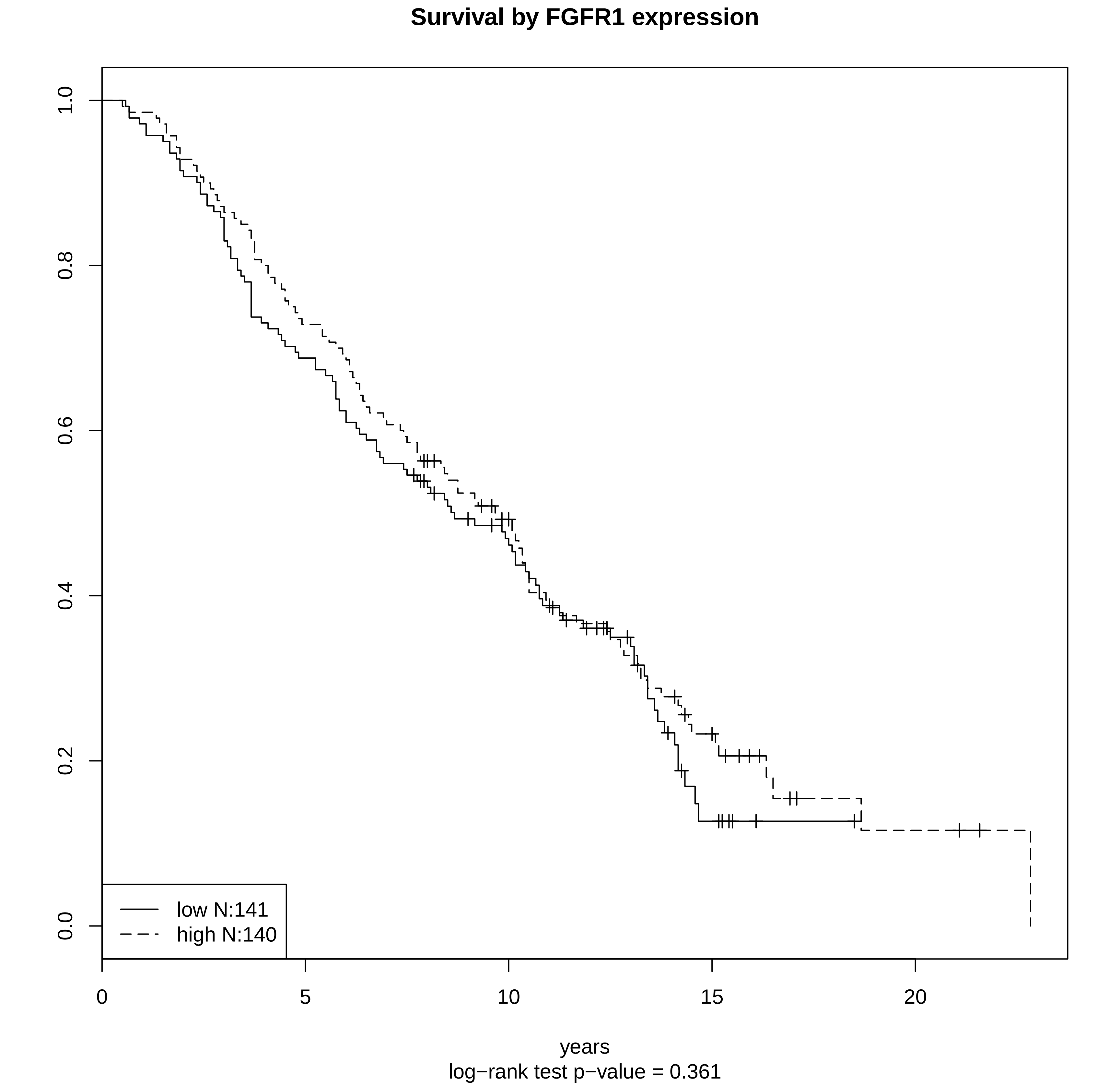

Survival by FGFR1 expression
1.0
0.8
0.6
0.4
0.2
0.0
0
5
10
15
20
years
log−rank test p−value = 0.361
low N:141
high N:140

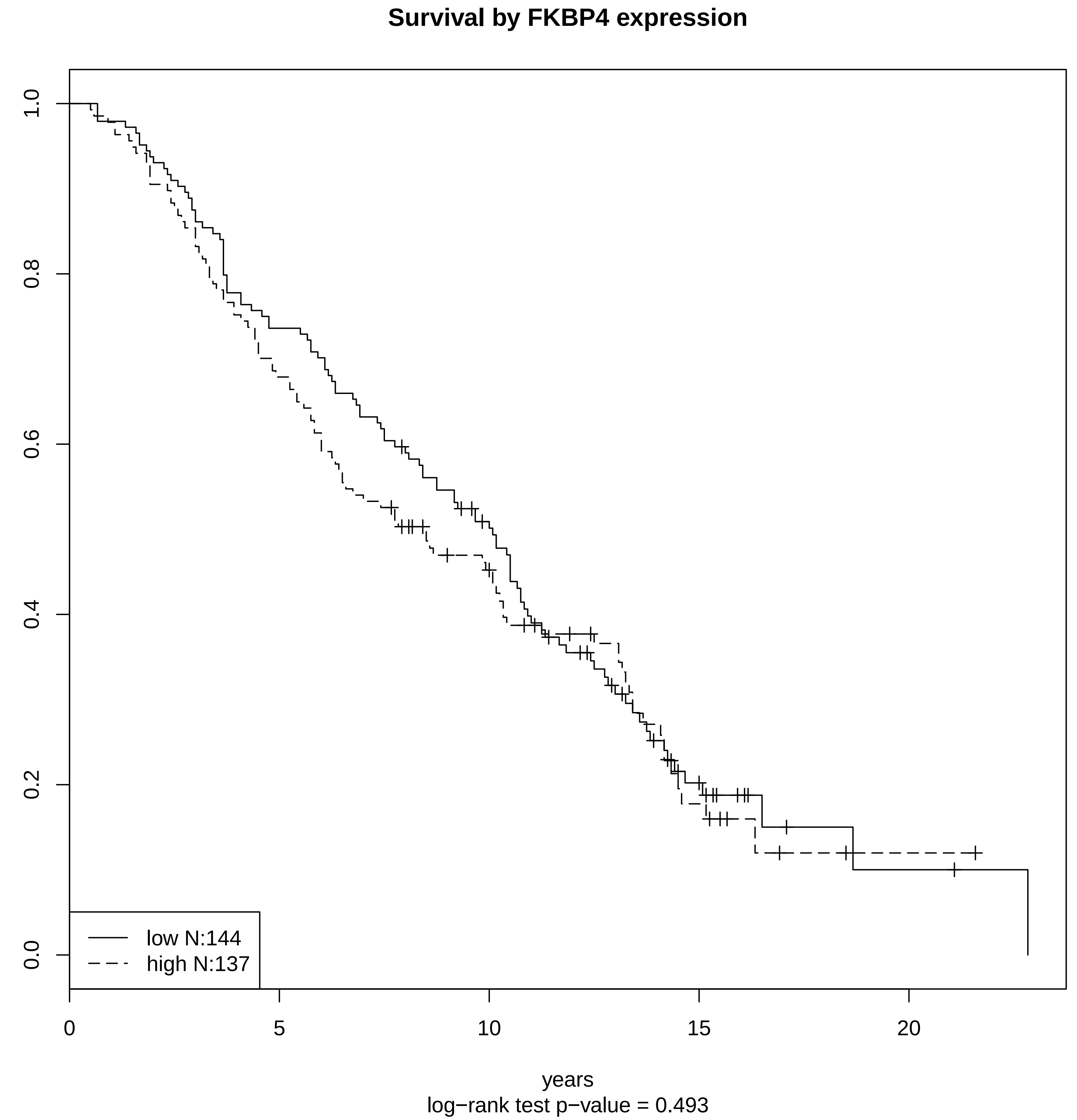
Survival by FKBP4 expression
1.0
0.8
0.6
0.4
0.2
0.0
0
5
10
15
20
years
log−rank test p−value = 0.493
low N:144
high N:137

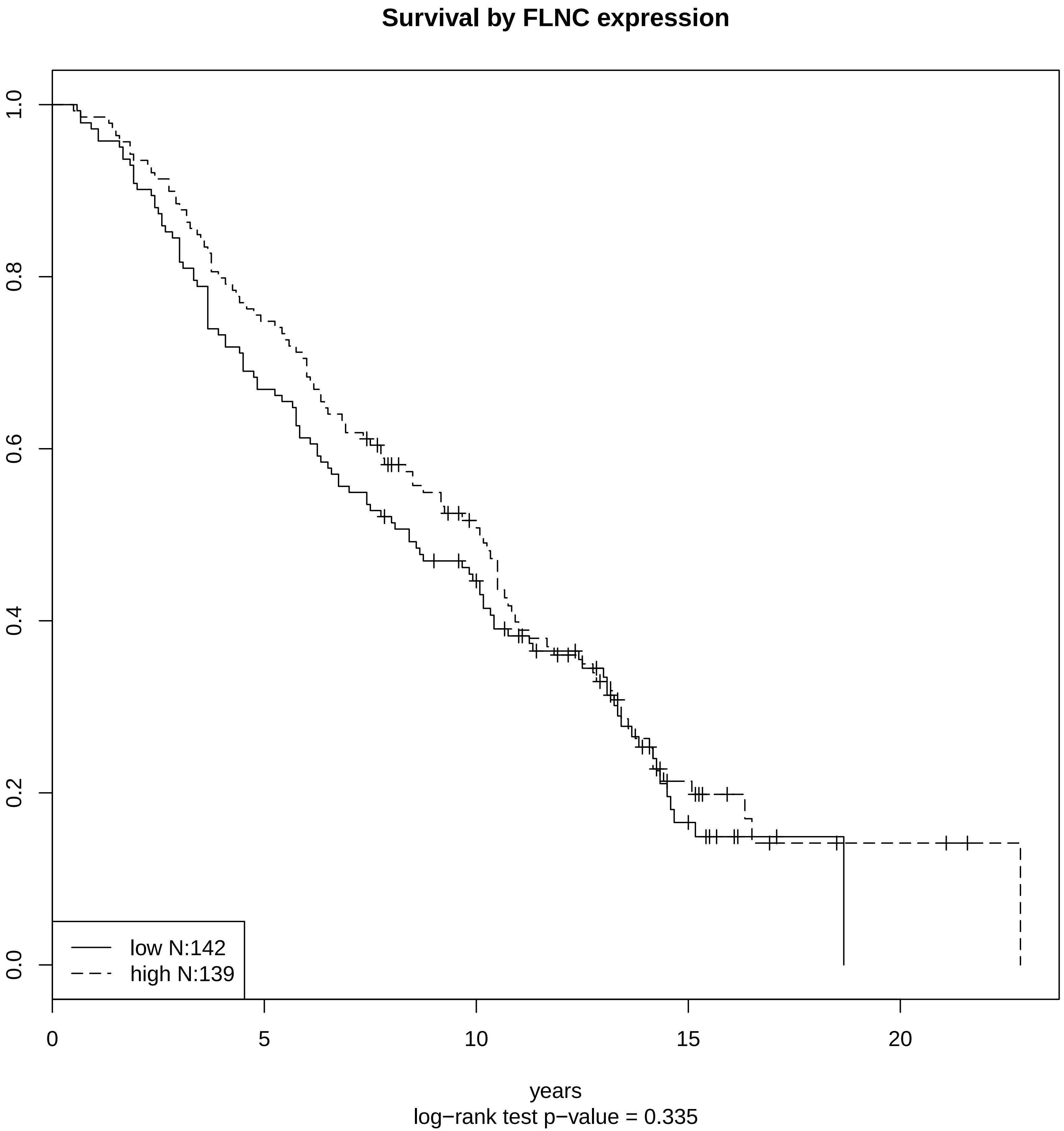

Survival by FLNC expression
1.0
0.8
0.6
0.4
0.2
0.0
0
5
10
15
20
low N:142
high N:139
years
log−rank test p−value = 0.335

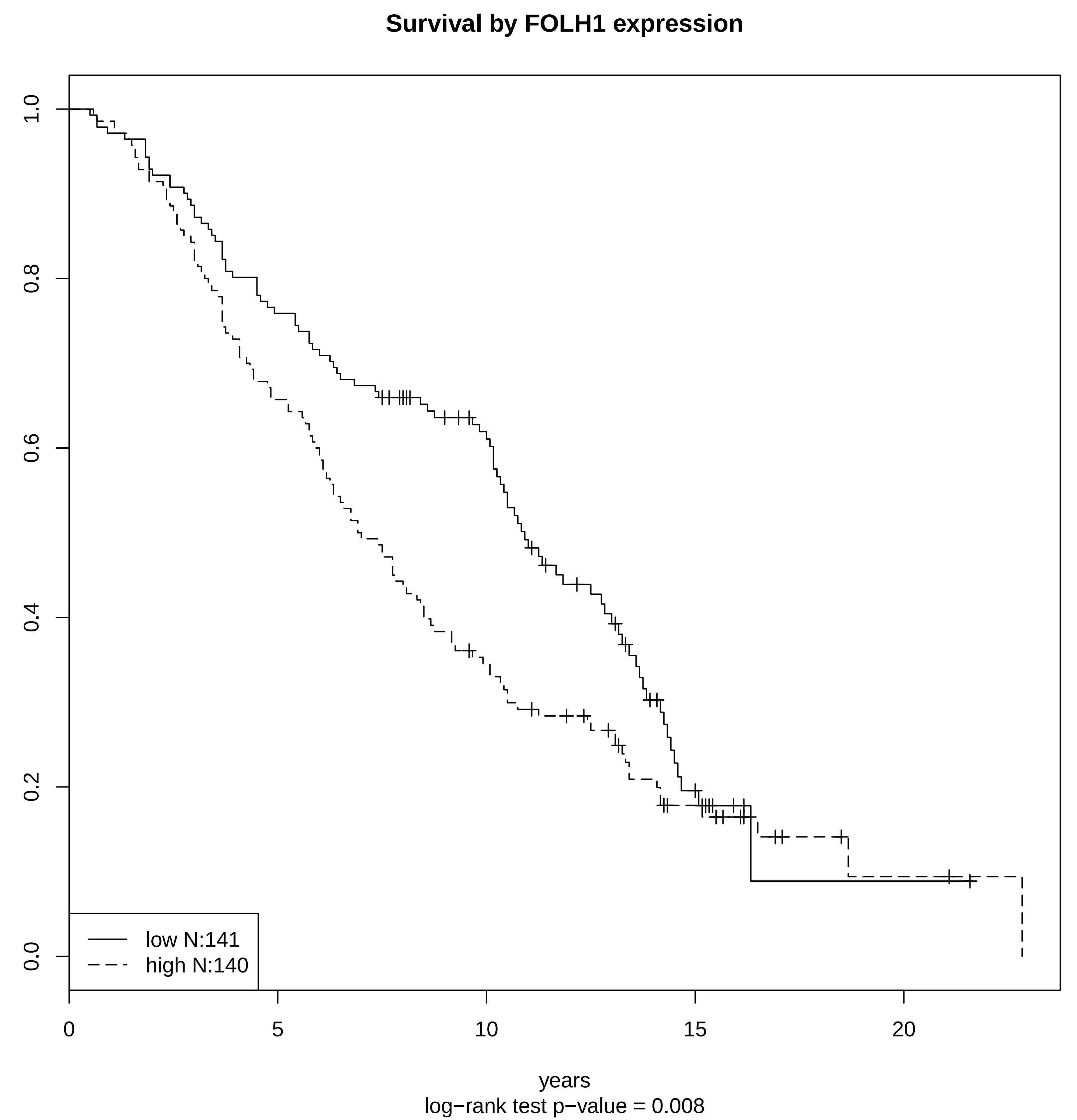
Survival by FOLH1 expression
1.0
0.8
0.6
0.4
0.2
0.0
0
5
10
15
20
years
log−rank test p−value = 0.008
low N:141
high N:140

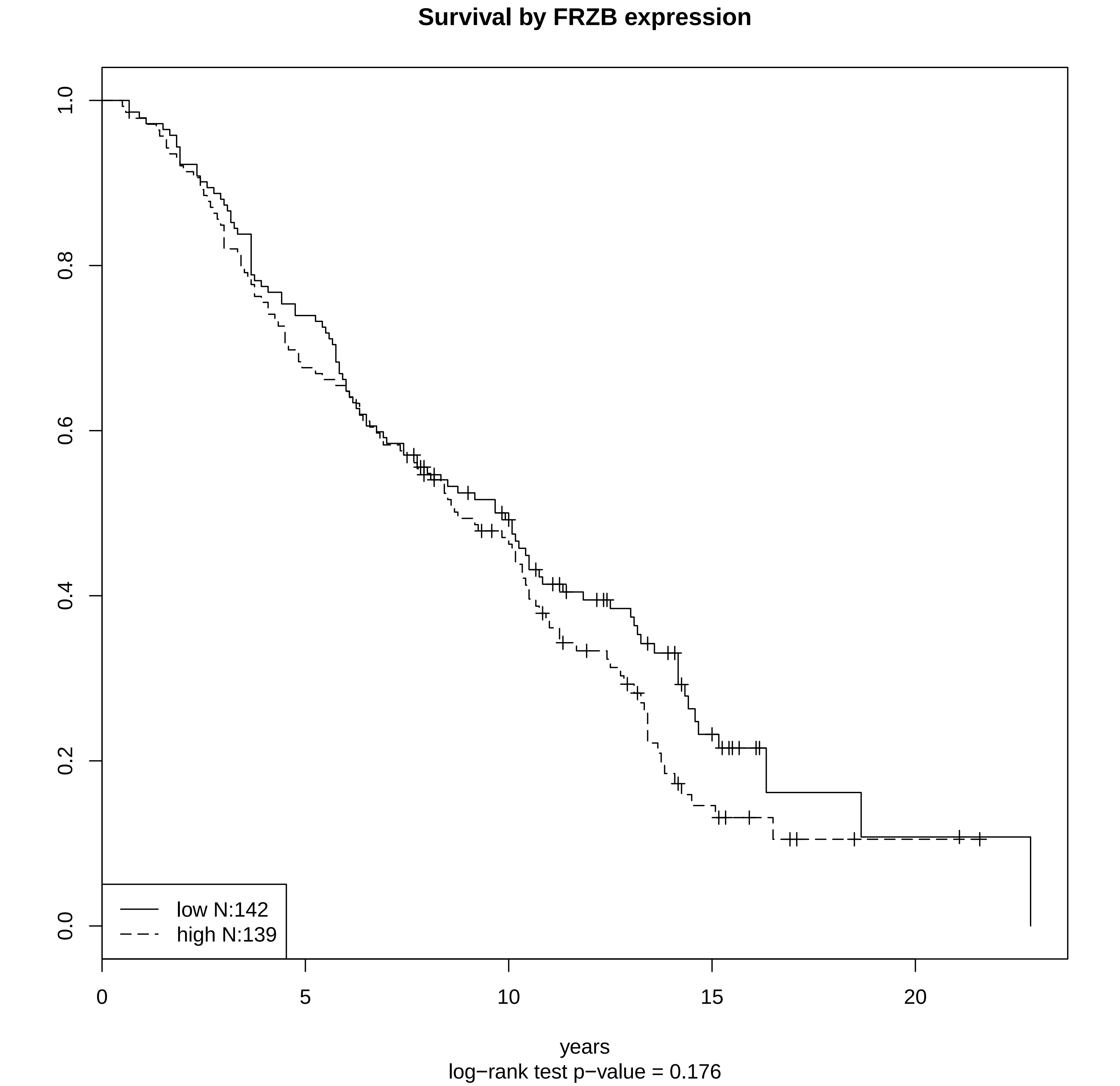

Survival by FRZB expression
1.0
0.8
0.6
0.4
0.2
0.0
0
5
10
15
20
low N:142
high N:139
years
log−rank test p−value = 0.176

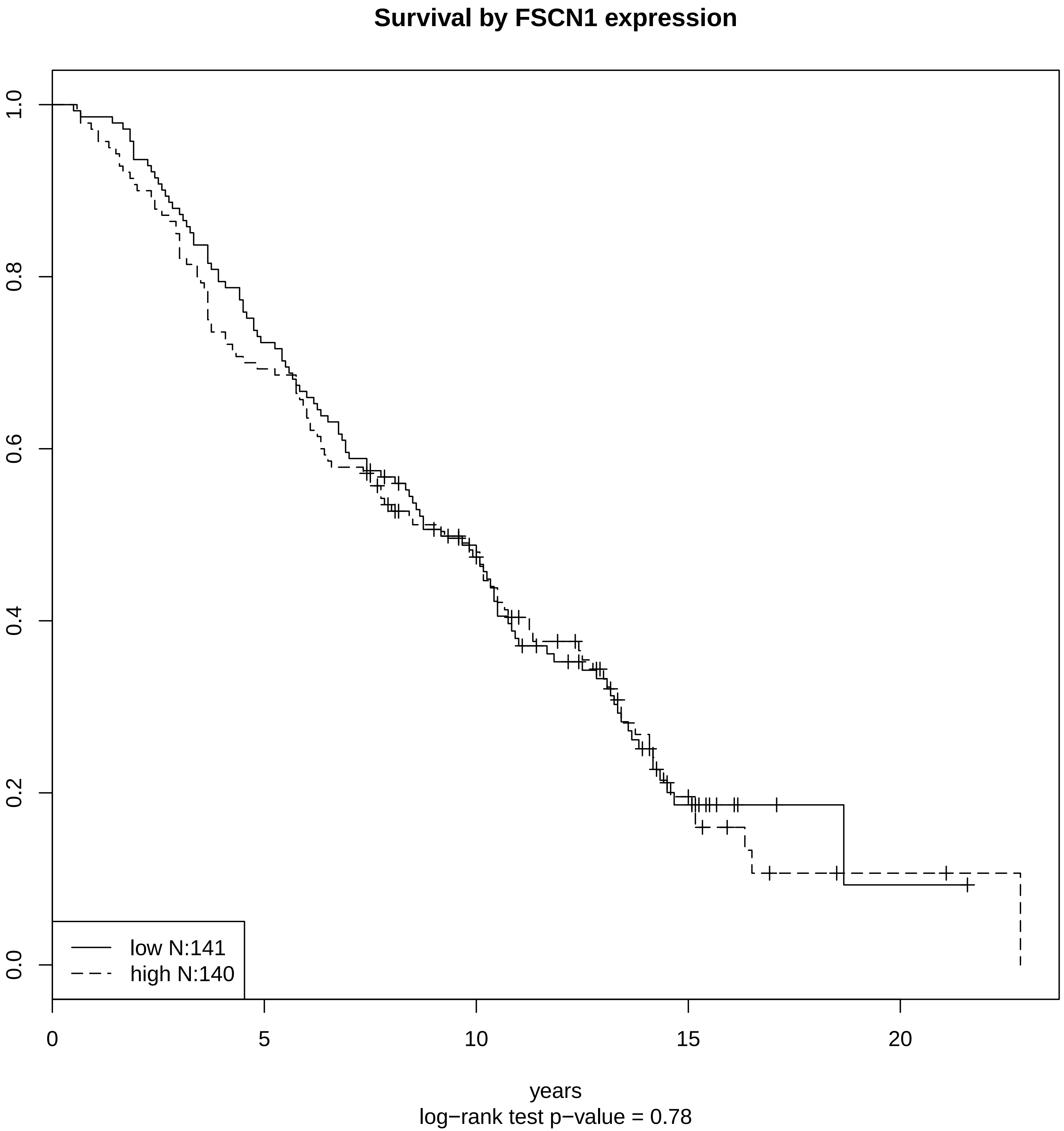

Survival by FSCN1 expression
1.0
0.8
0.6
0.4
0.2
0.0
0
5
10
15
20
years
log−rank test p−value = 0.78
low N:141
high N:140

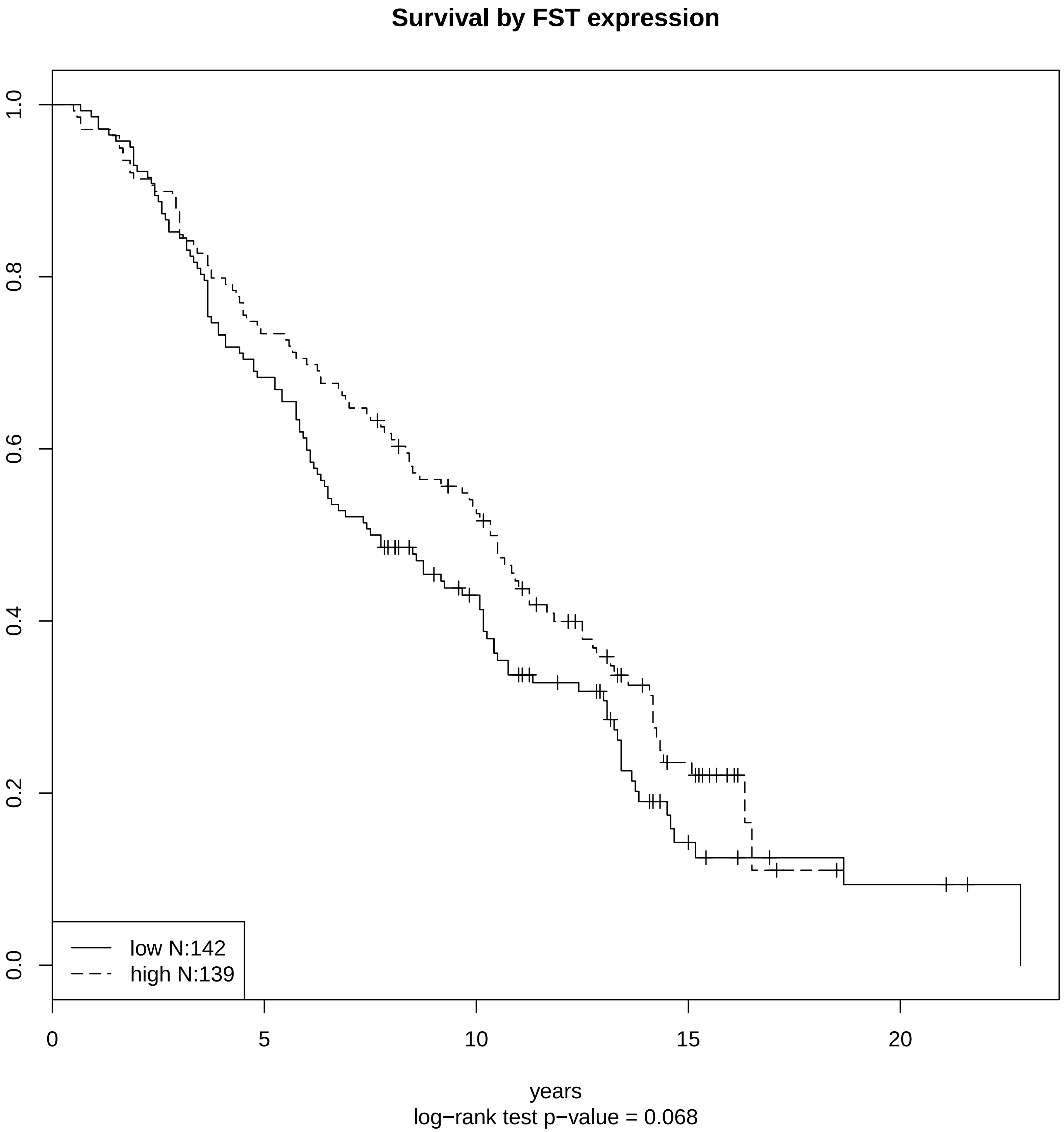

Survival by FST expression
1.0
0.8
0.6
0.4
0.2
0.0
low N:142
high N:139
0
5
10
15
20
years
log−rank test p−value = 0.068

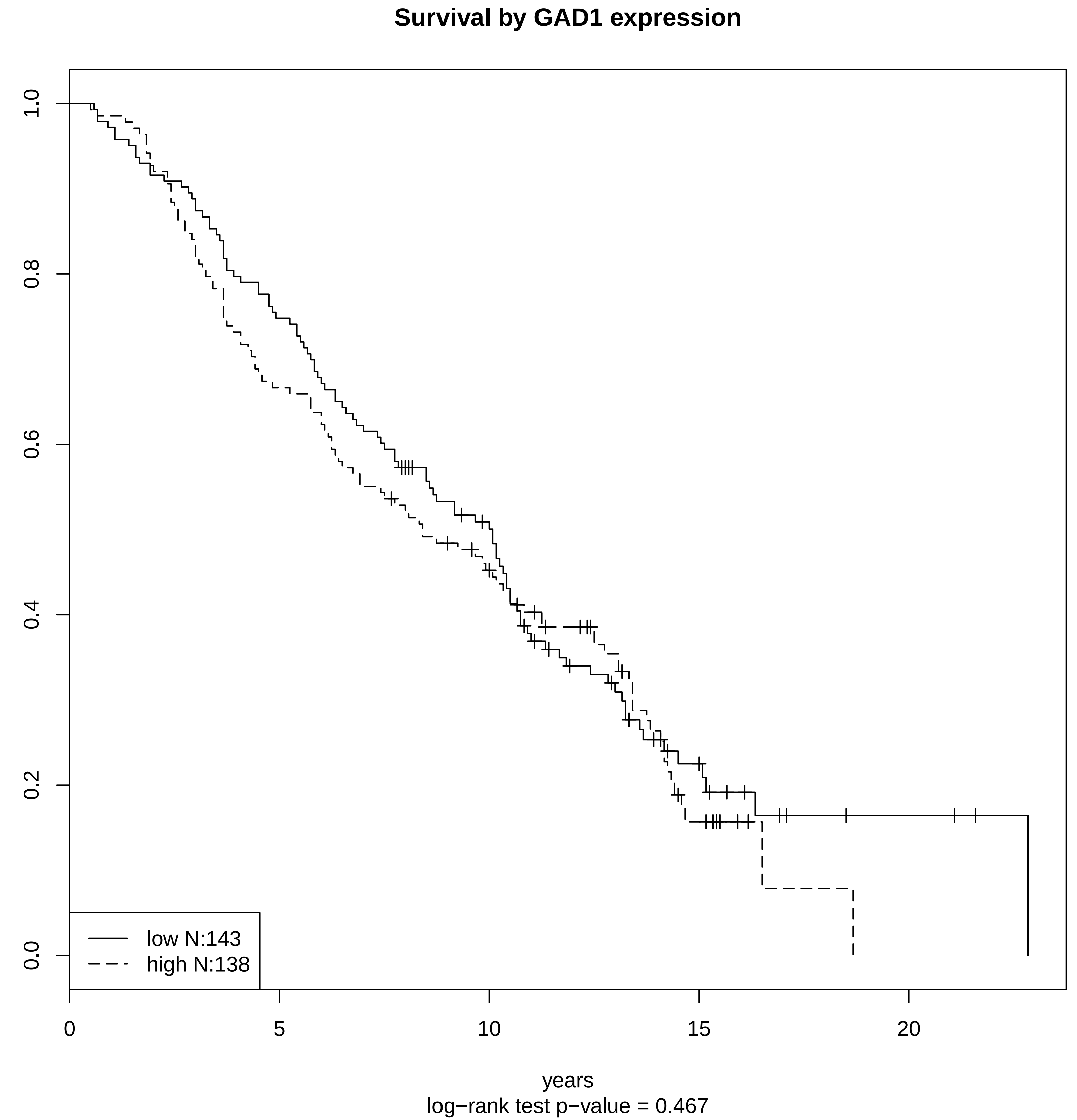

Survival by GAD1 expression
1.0
0.8
0.6
0.4
0.2
0.0
0
5
10
15
20
years
log−rank test p−value = 0.467
low N:143
high N:138

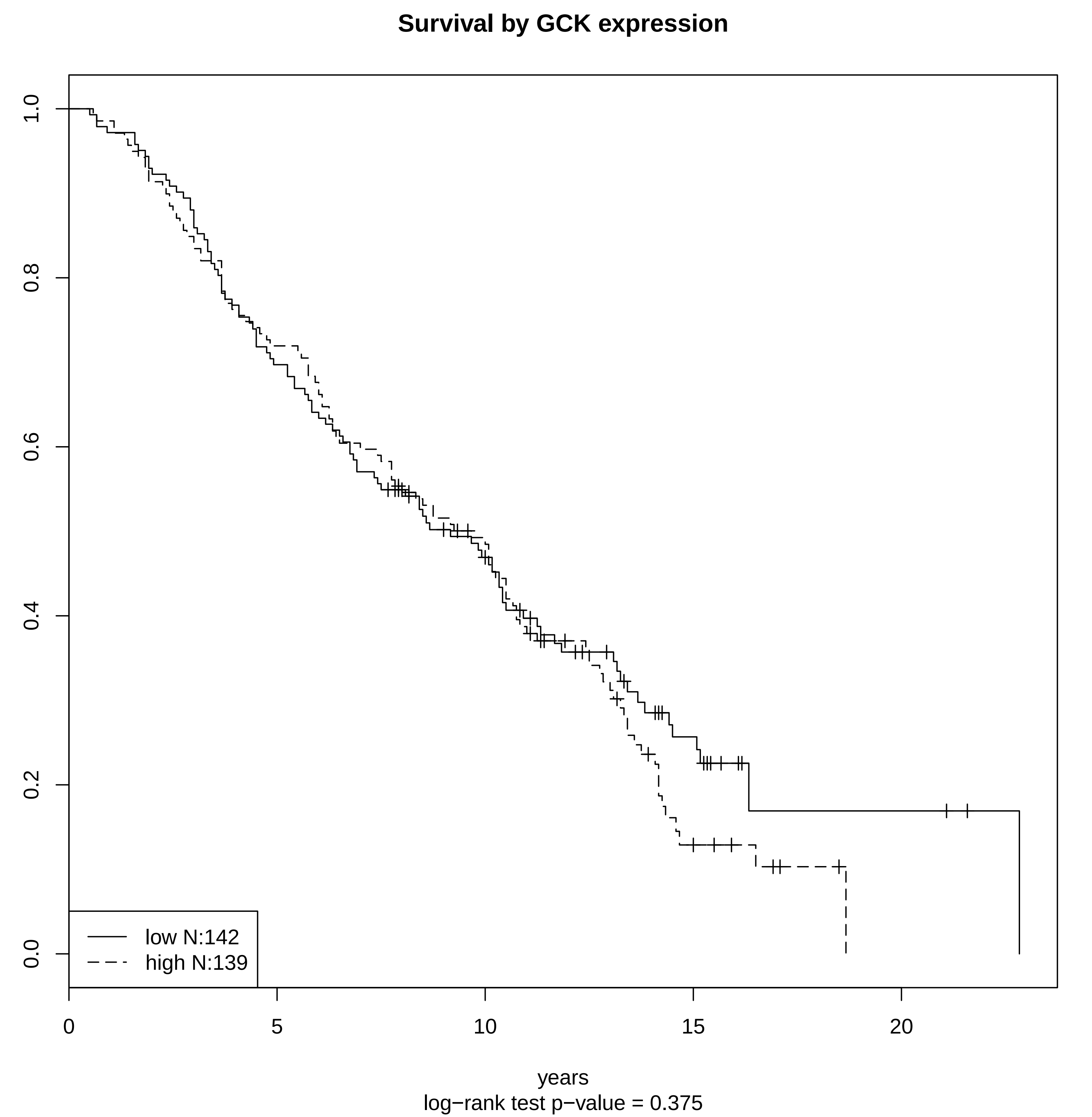
Survival by GCK expression
0.0
0.2
0.4
0.6
0.8
1.0
0
5
10
15
20
years
log−rank test p−value = 0.375
low N:142
high N:139

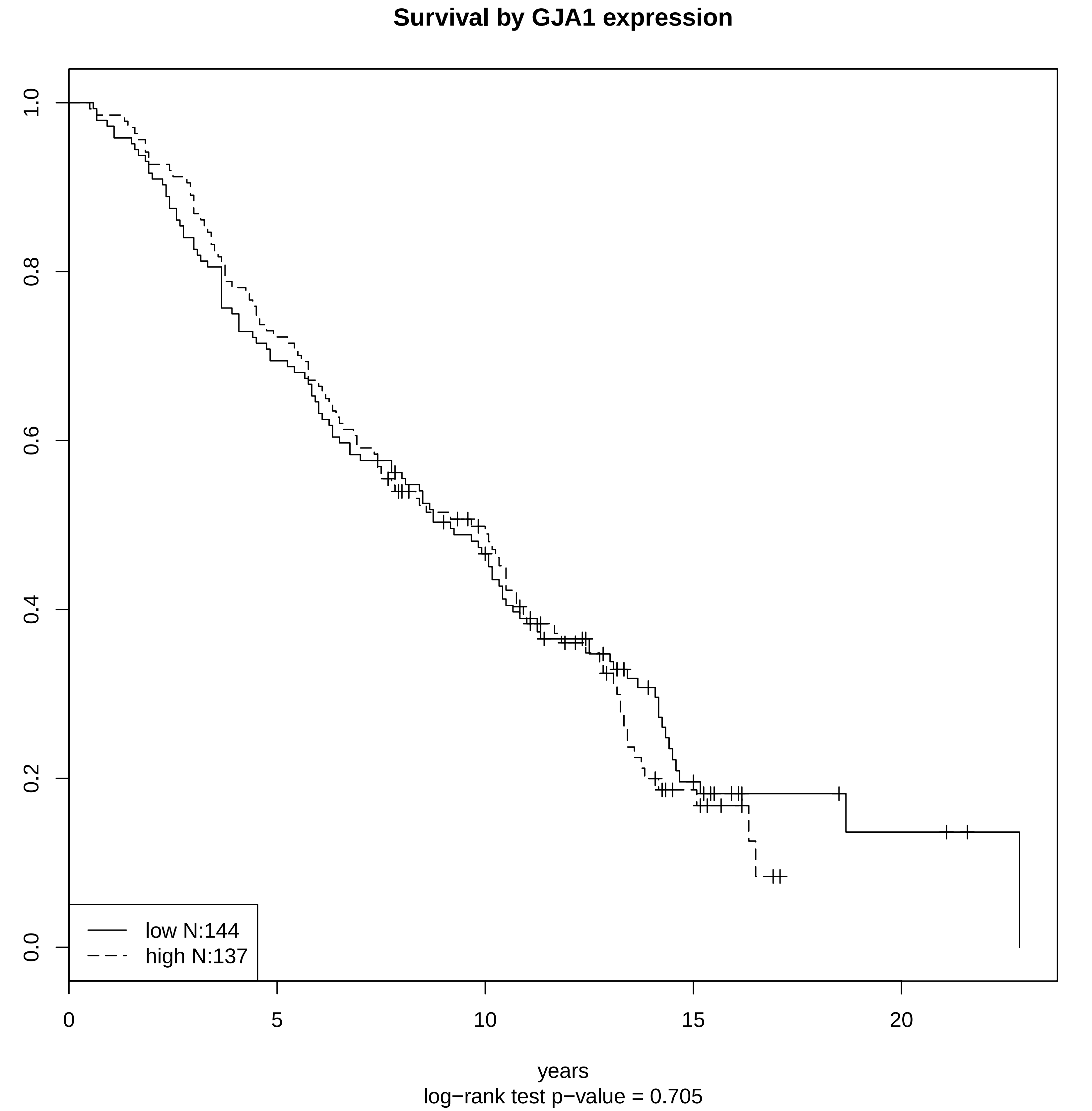
Survival by GJA1 expression
1.0
0.8
0.6
0.4
0.2
0.0
0
5
10
15
20
years
log−rank test p−value = 0.705
low N:144
high N:137

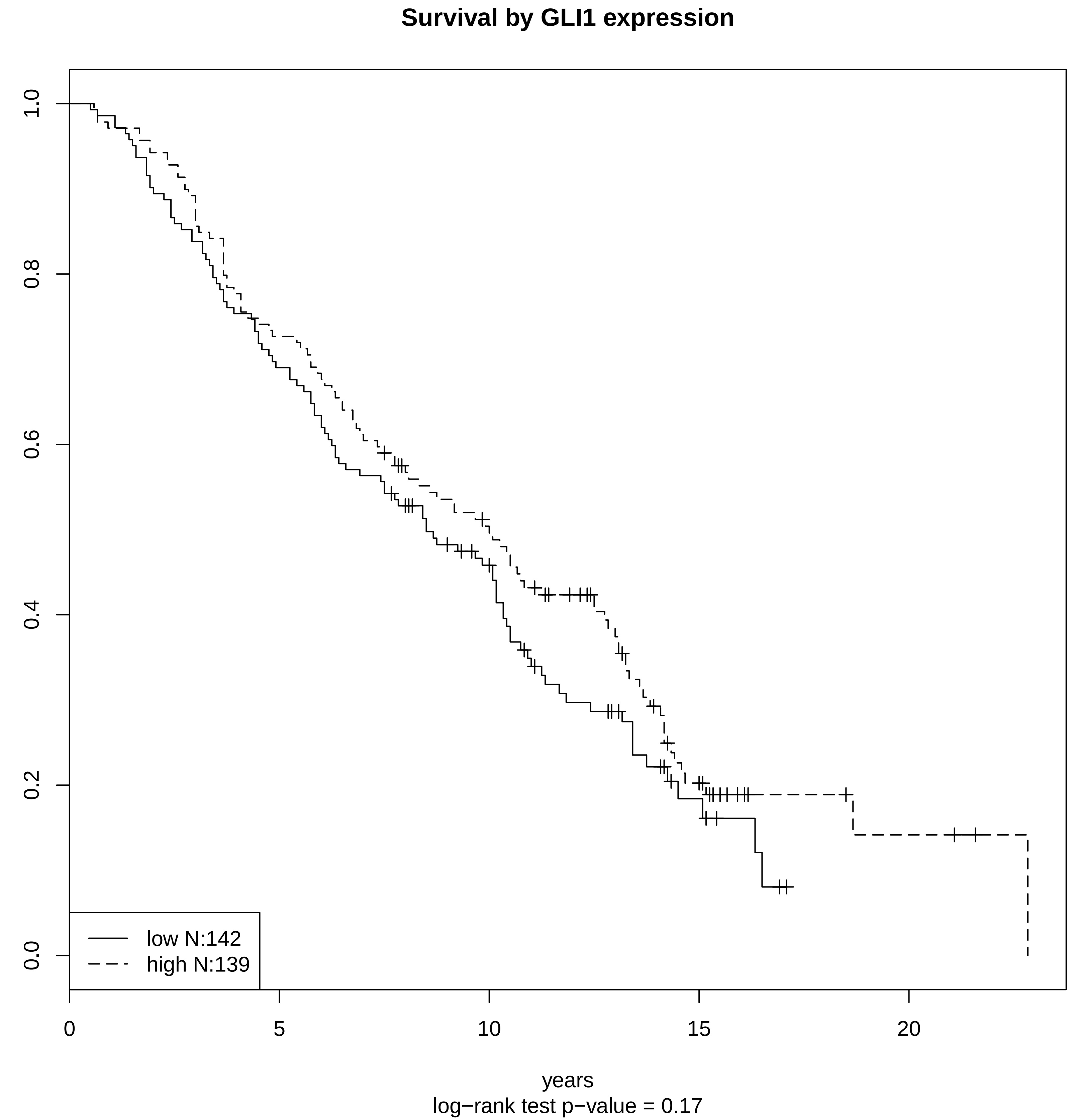
Survival by GLI1 expression
1.0
0.8
0.6
0.4
0.2
0.0
0
5
10
15
20
years
log−rank test p−value = 0.17
low N:142
high N:139

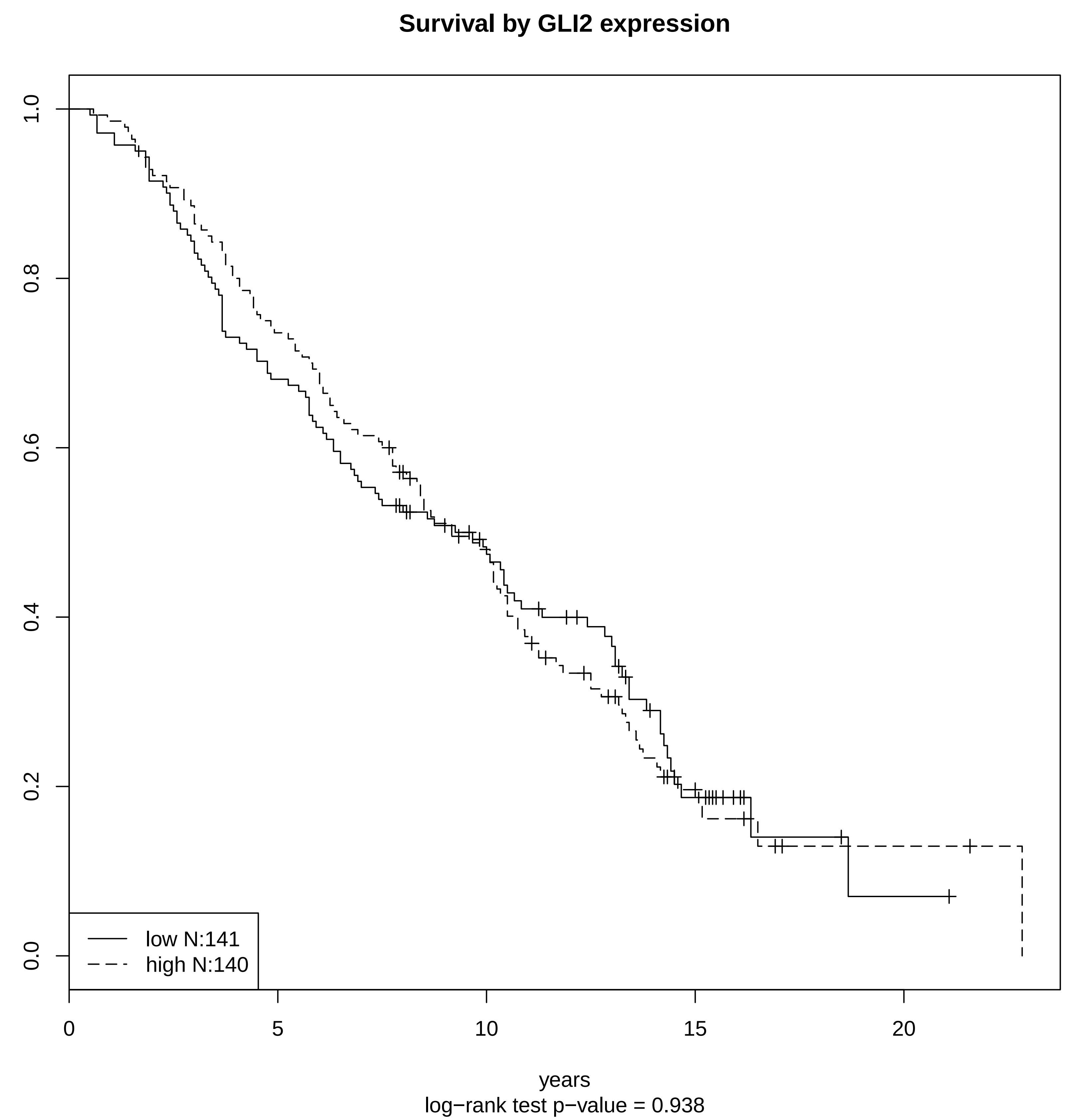

Survival by GLI2 expression
1.0
0.8
0.6
0.4
0.2
0.0
0
5
10
15
20
low N:141
high N:140
years
log−rank test p−value = 0.938

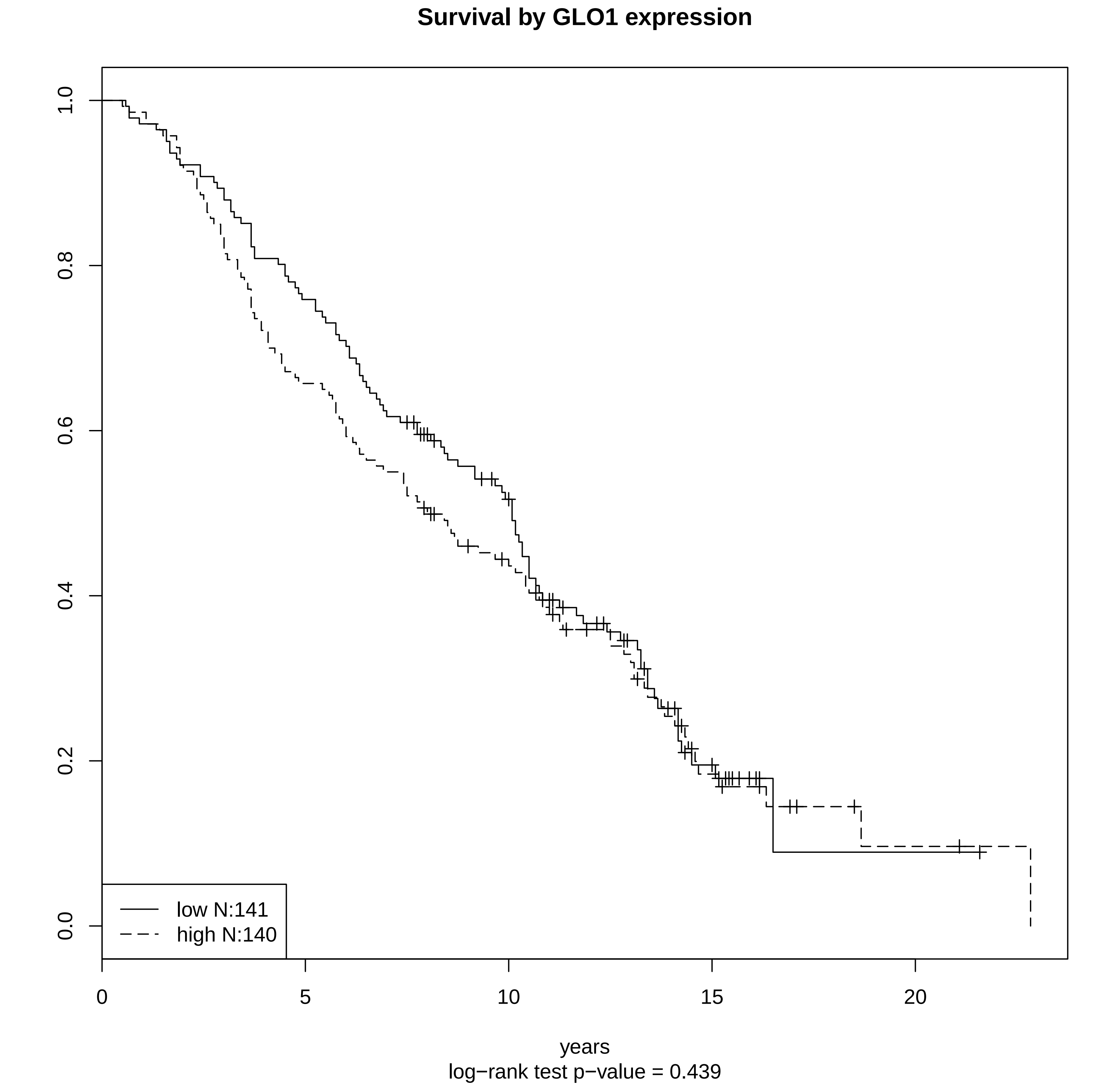
Survival by GLO1 expression
1.0
0.8
0.6
0.4
0.2
0.0
0
5
10
15
20
low N:141
high N:140
years
log−rank test p−value = 0.439

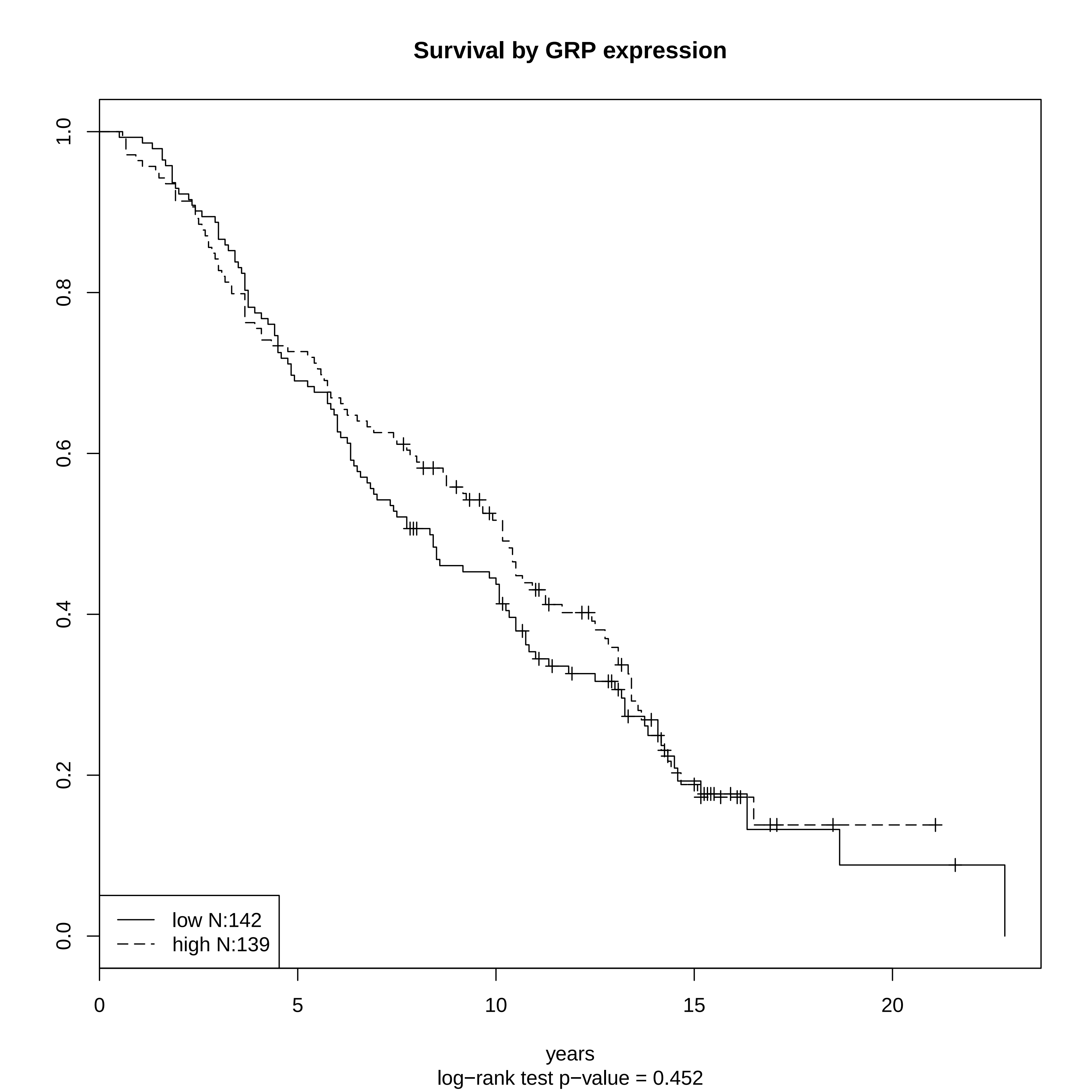

Survival by GRP expression
1.0
0.8
0.6
0.4
0.2
0.0
0
5
10
15
20
low N:142
high N:139
years
log−rank test p−value = 0.452

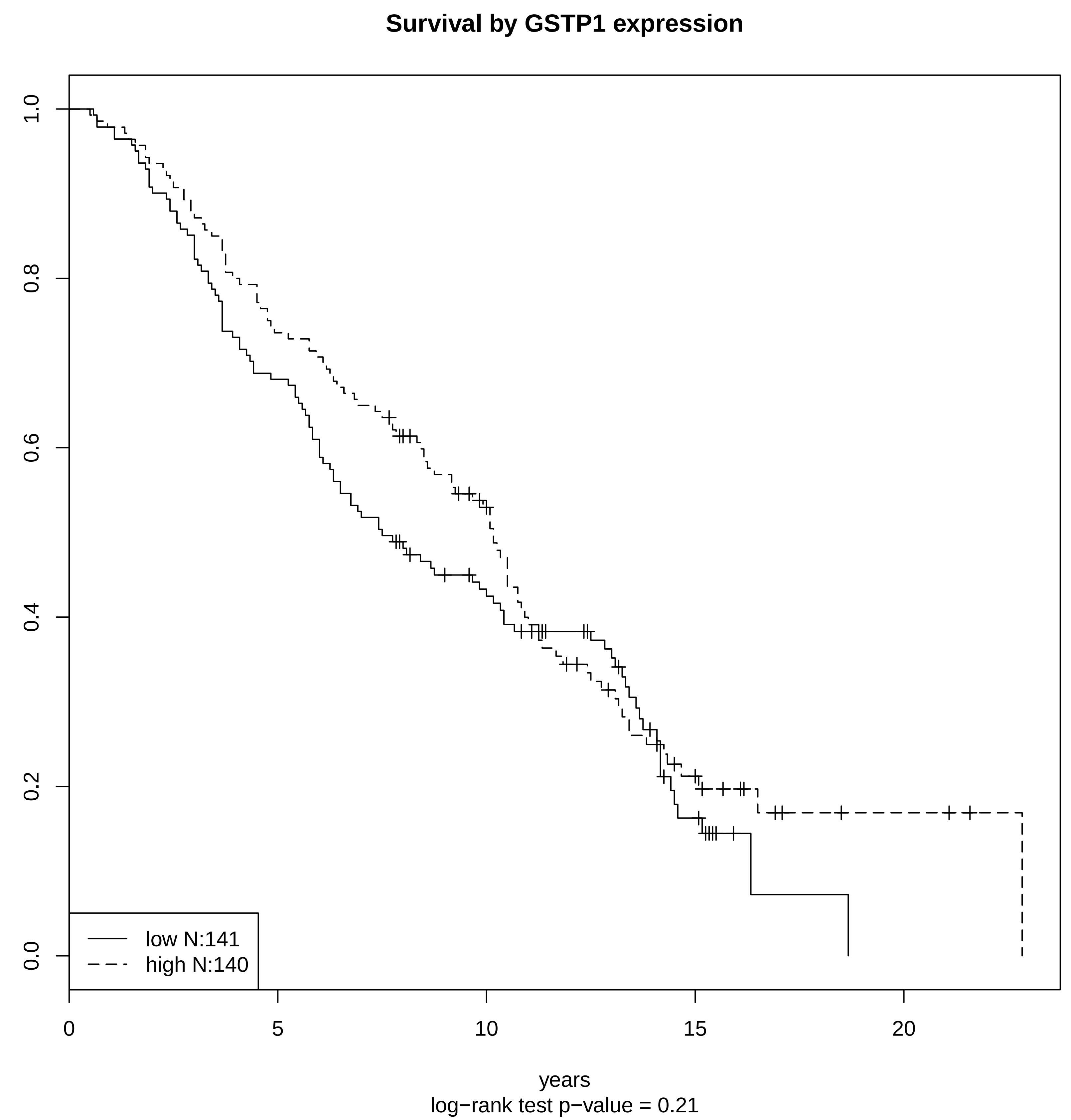

Survival by GSTP1 expression
1.0
0.8
0.6
0.4
0.2
0.0
0
5
10
15
20
low N:141
high N:140
years
log−rank test p−value = 0.21

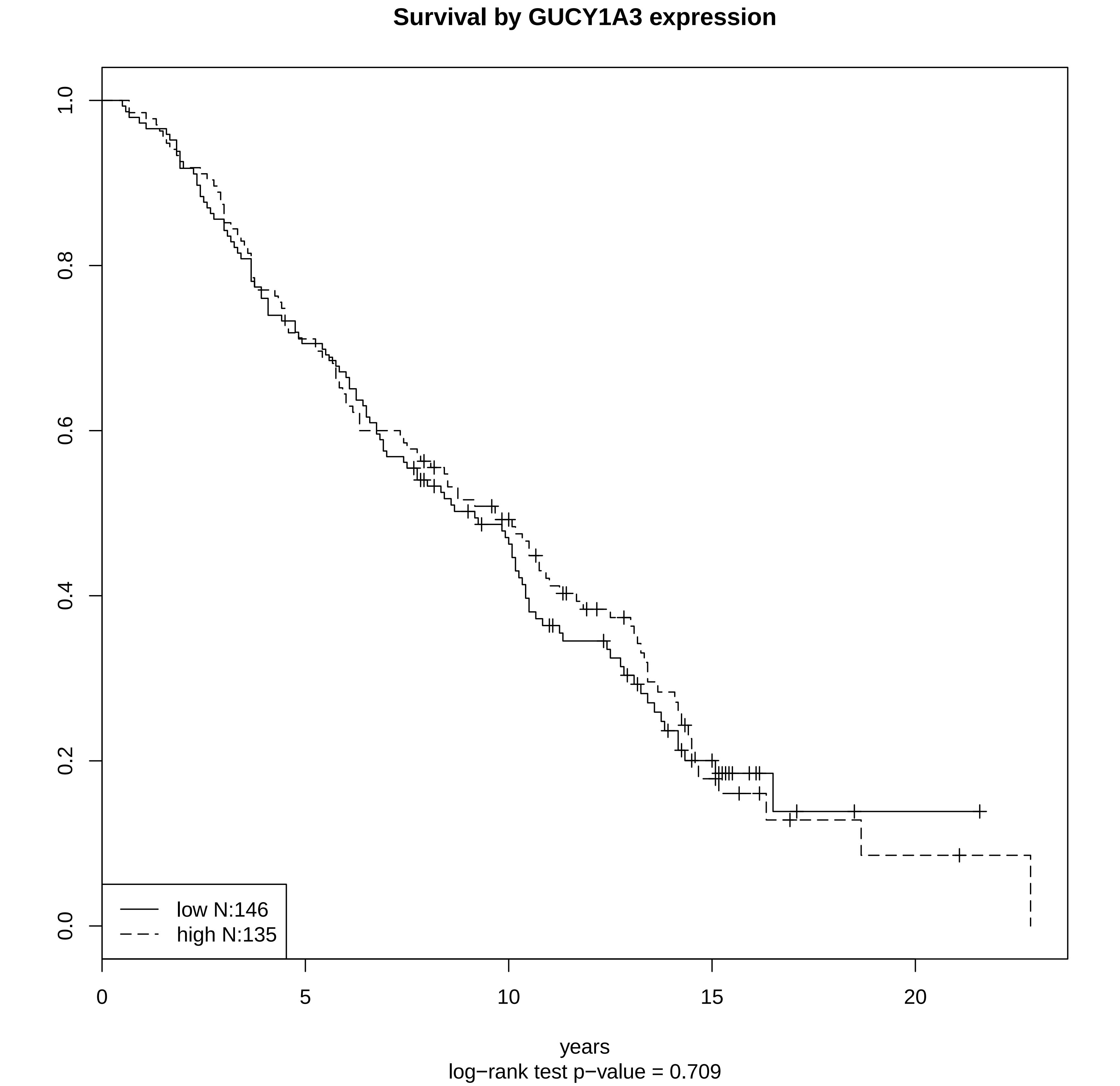
Survival by GUCY1A3 expression
1.0
0.8
0.6
0.4
0.2
0.0
0
5
10
15
20
years
low N:146
high N:135
log−rank test p−value = 0.709

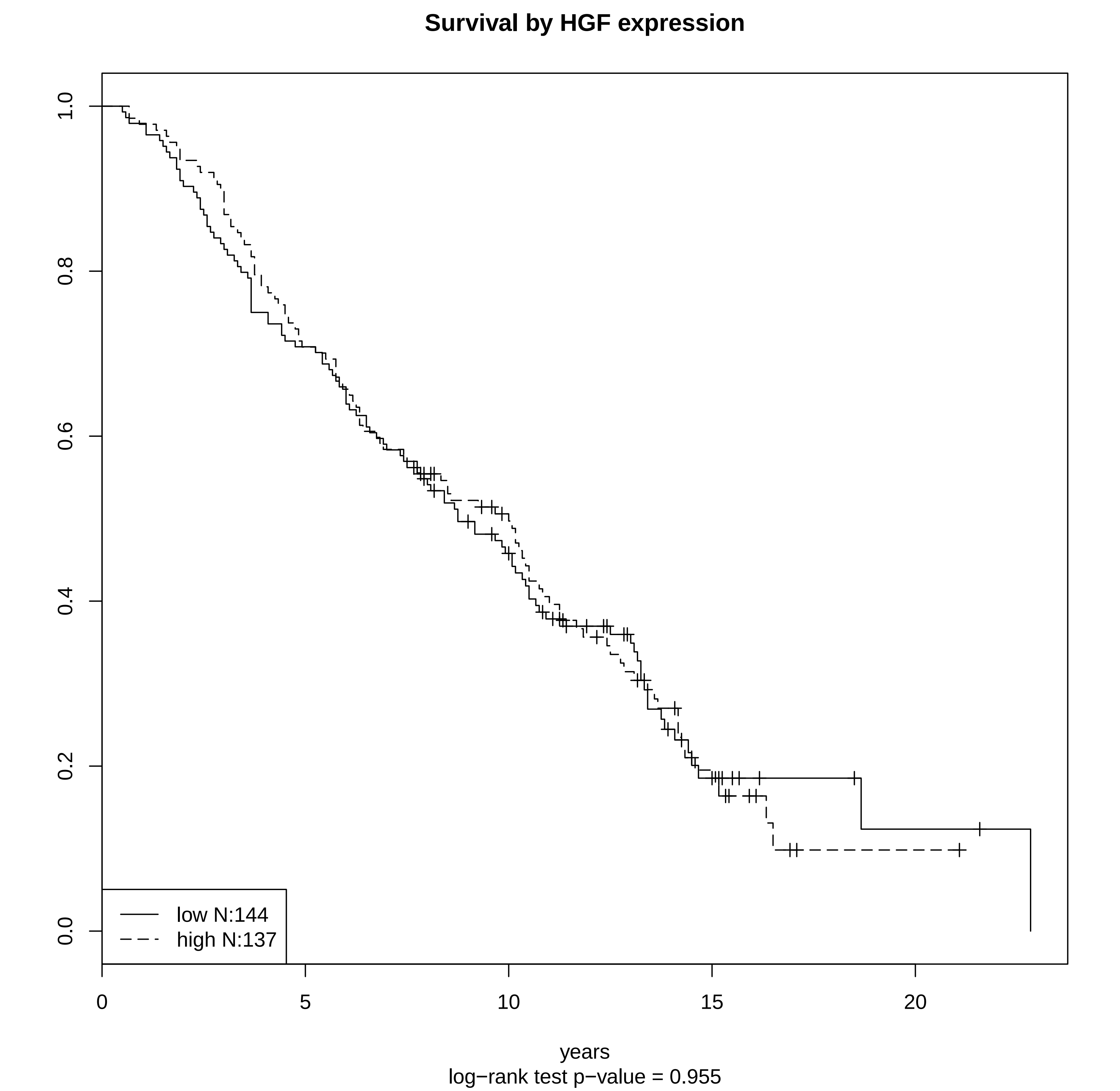
Survival by HGF expression
1.0
0.8
0.6
0.4
0.2
0.0
0
5
10
15
20
low N:144
high N:137
years
log−rank test p−value = 0.955

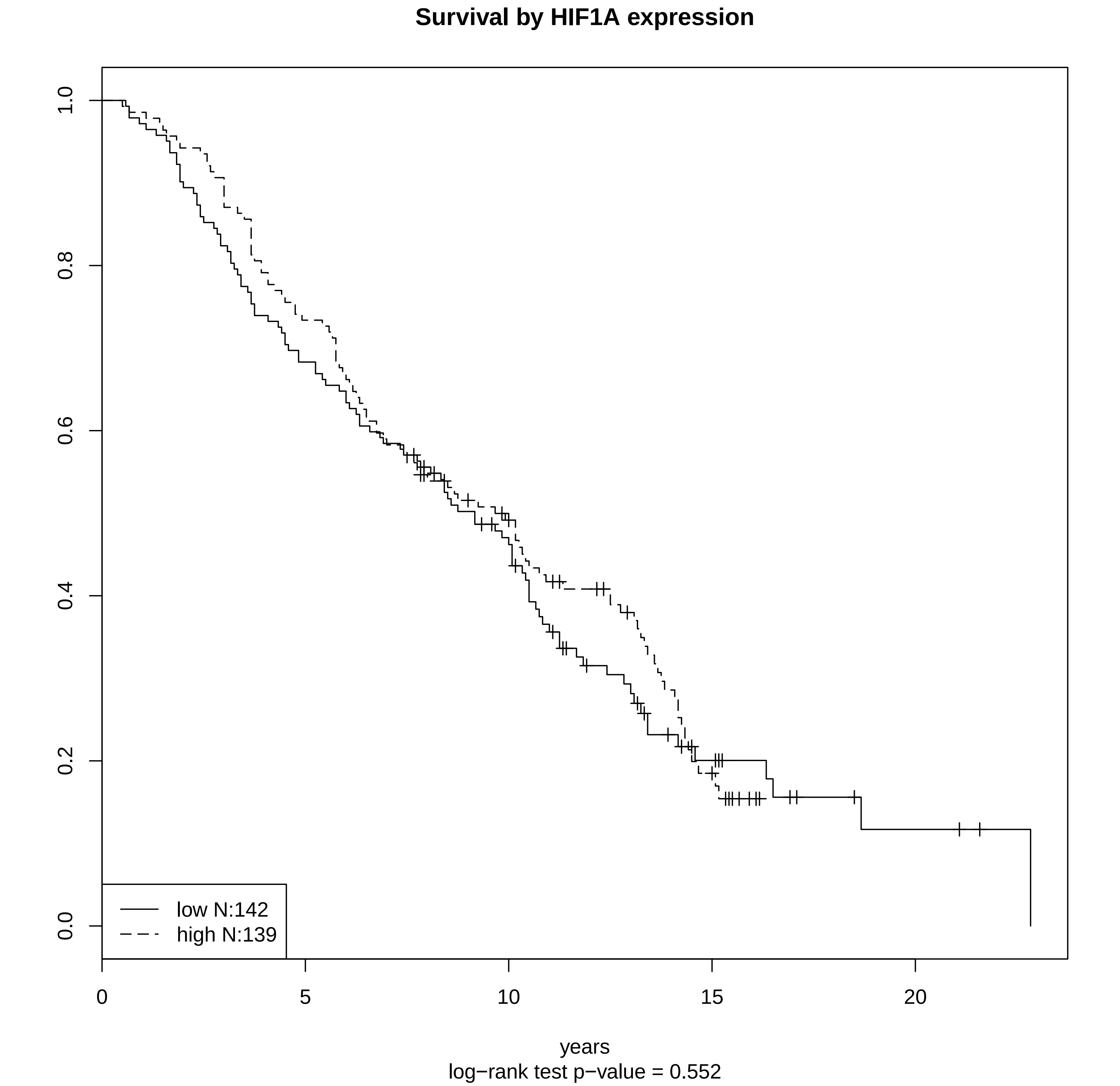

Survival by HIF1A expression
1.0
0.8
0.6
0.4
0.2
0.0
0
5
10
15
20
low N:142
high N:139
years
log−rank test p−value = 0.552

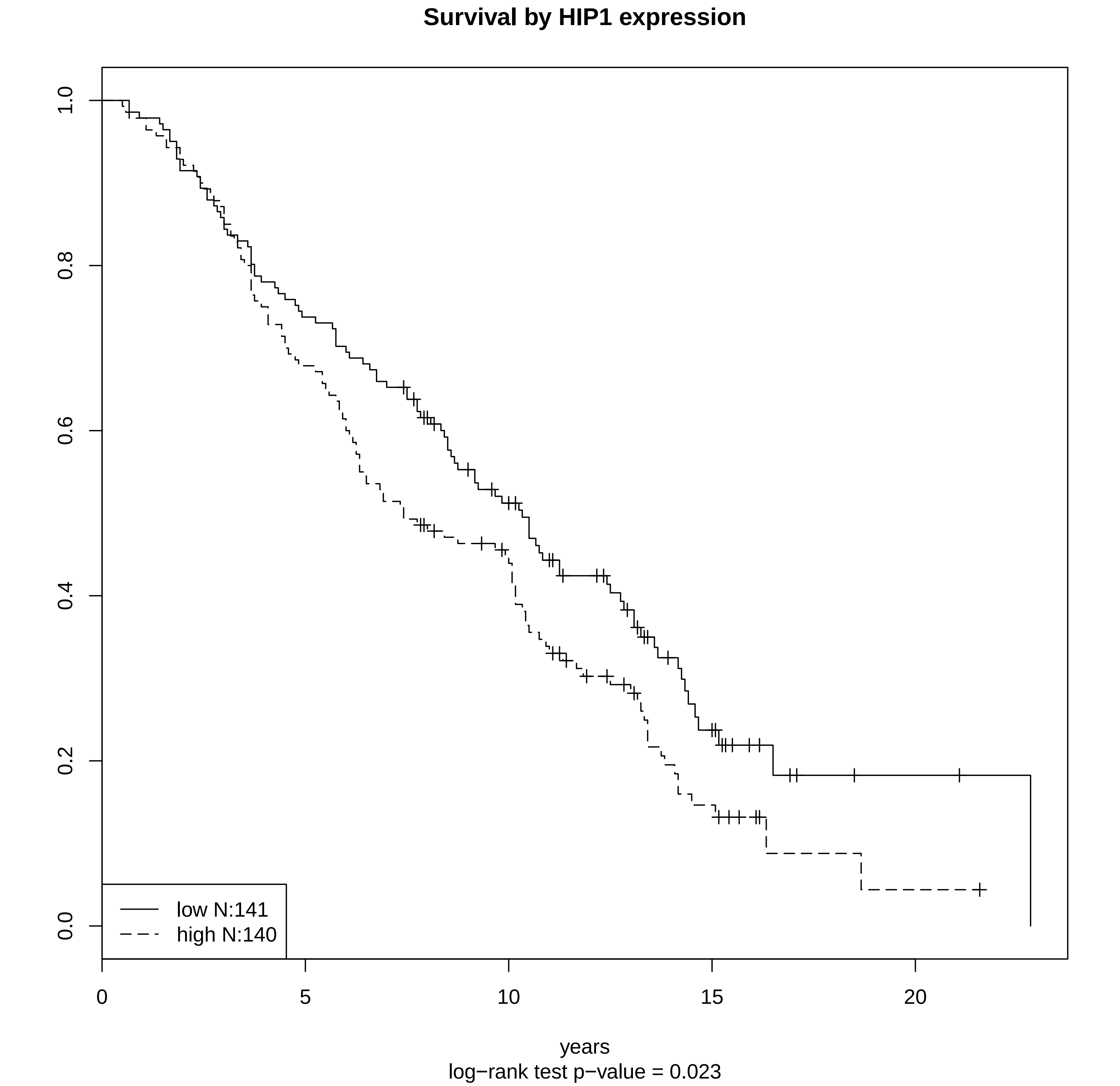
Survival by HIP1 expression
1.0
0.8
0.6
0.4
0.2
0.0
0
5
10
15
20
low N:141
high N:140
years
log−rank test p−value = 0.023

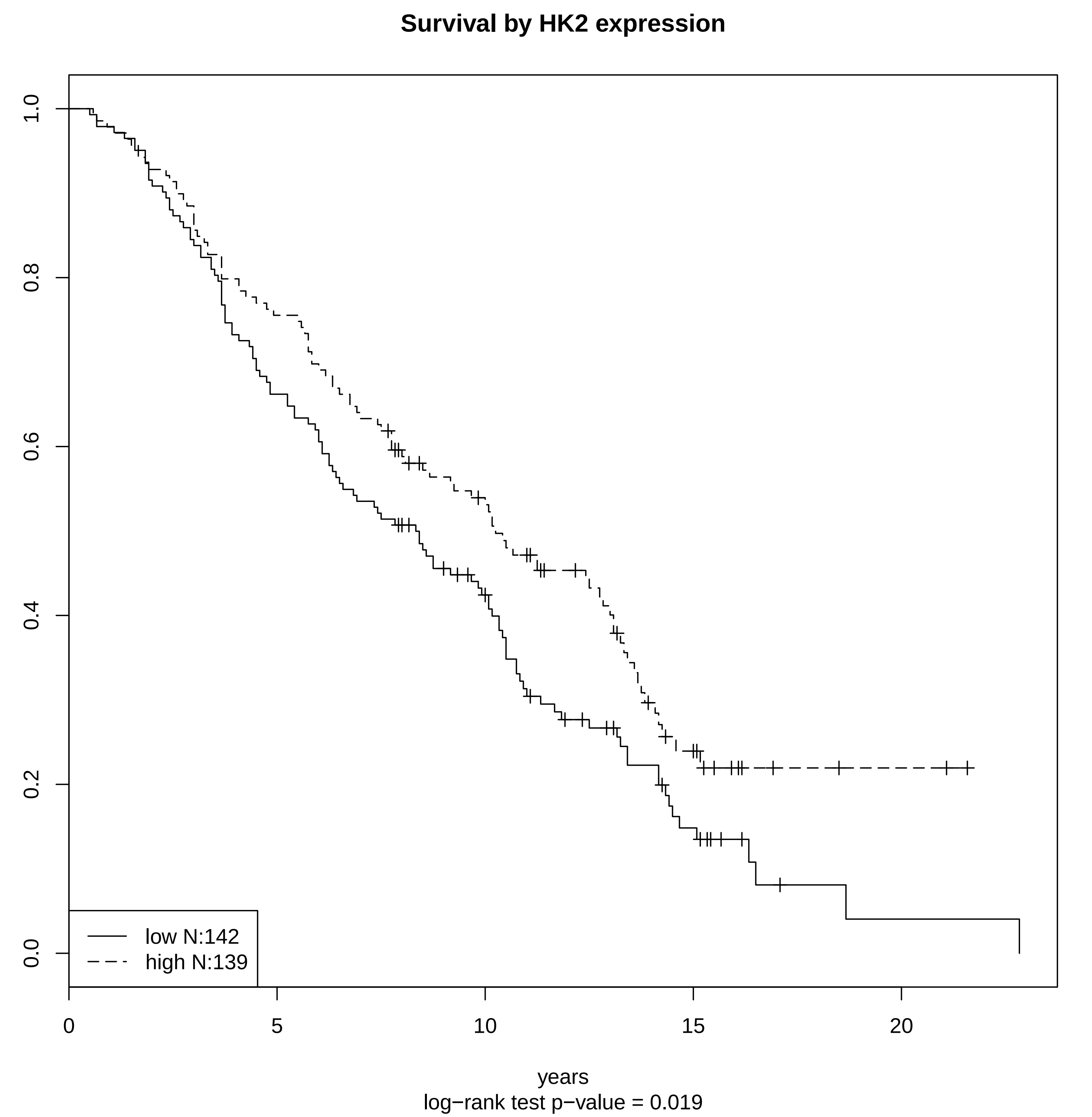
Survival by HK2 expression
1.0
0.8
0.6
0.4
0.2
0.0
0
5
10
15
20
low N:142
high N:139
years
log−rank test p−value = 0.019

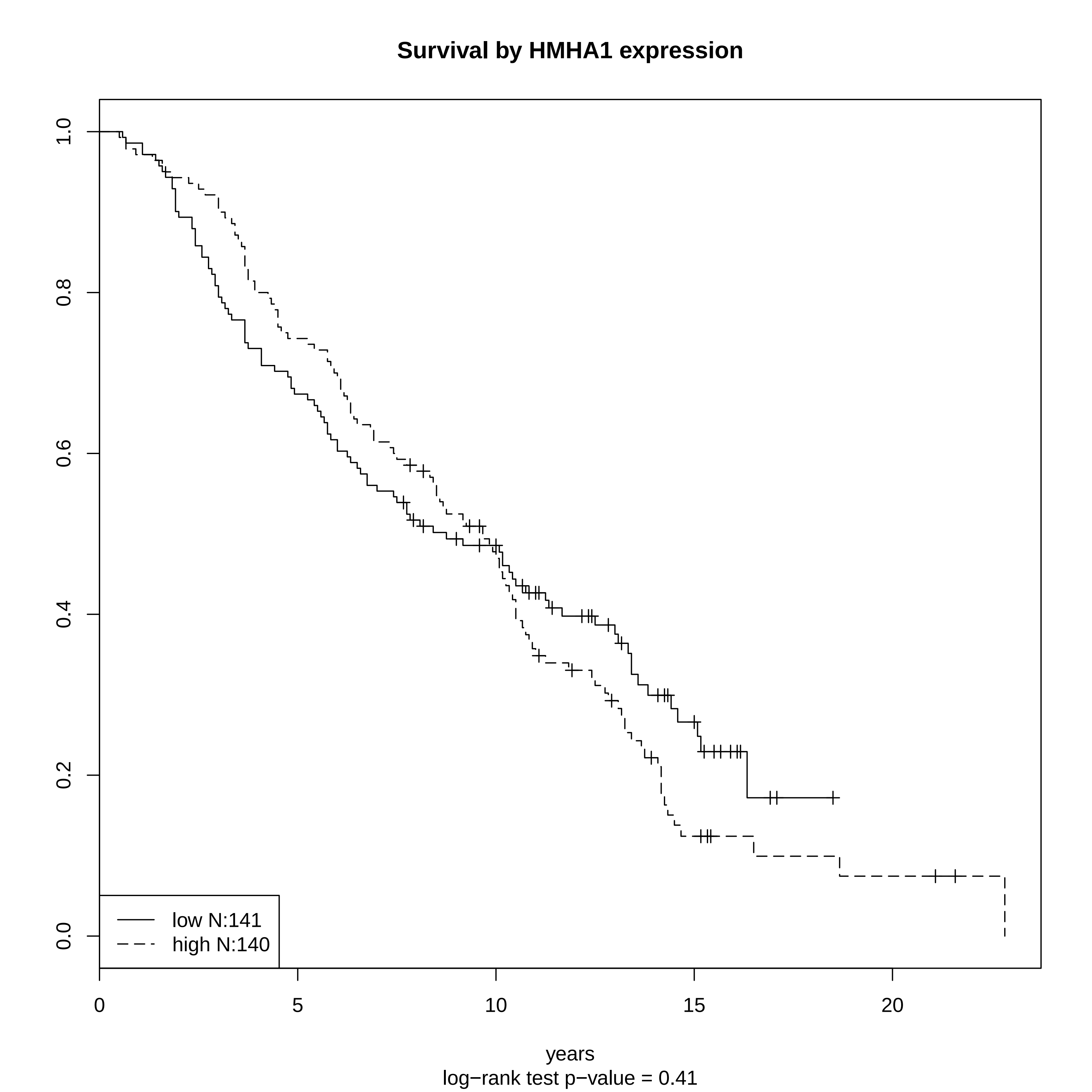

Survival by HMHA1 expression
1.0
0.8
0.6
0.4
0.2
0.0
0
5
10
15
20
low N:141
high N:140
years
log−rank test p−value = 0.41

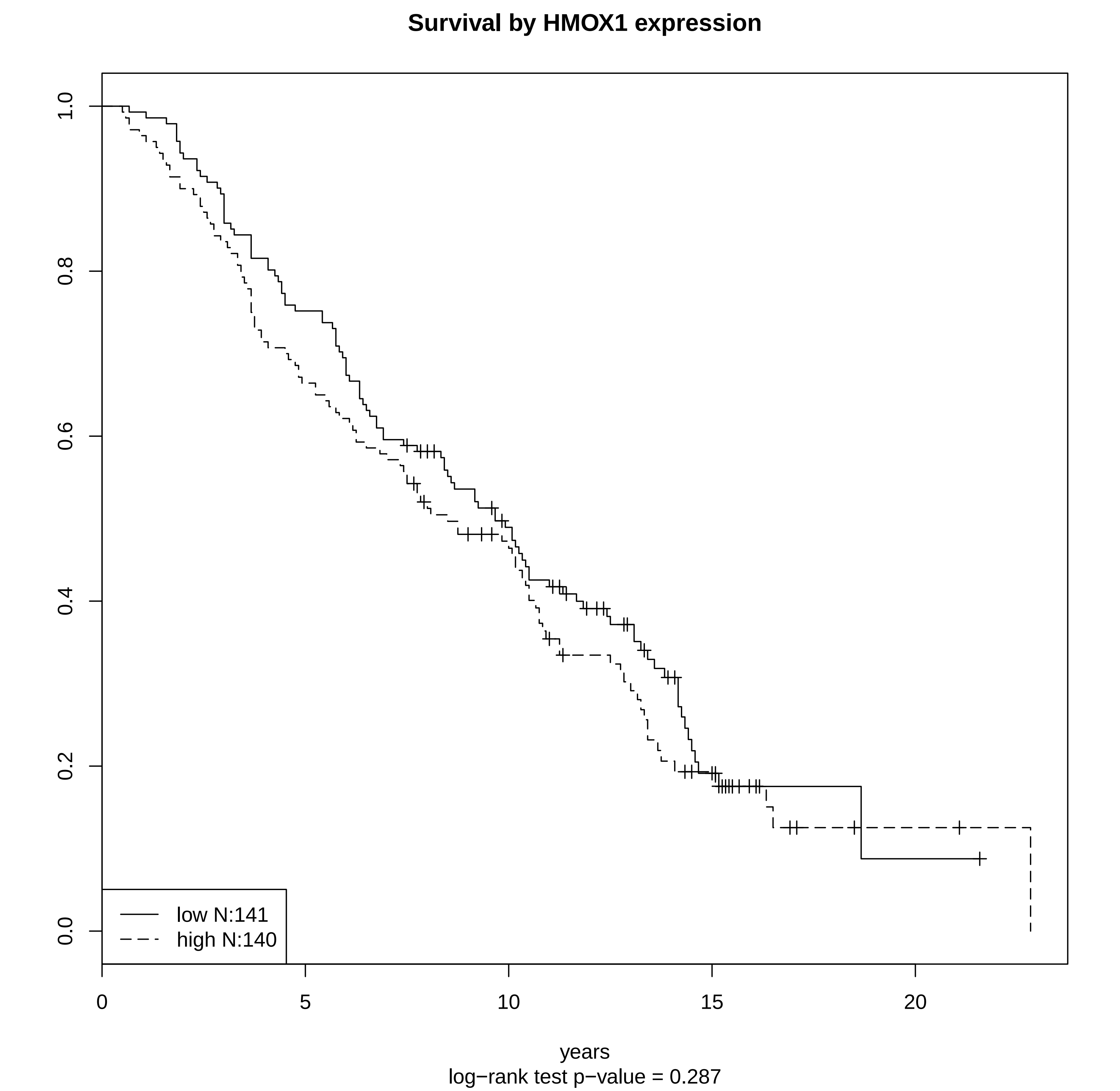
Survival by HMOX1 expression
1.0
0.8
0.6
0.4
0.2
0.0
0
5
10
15
20
low N:141
high N:140
years
log−rank test p−value = 0.287

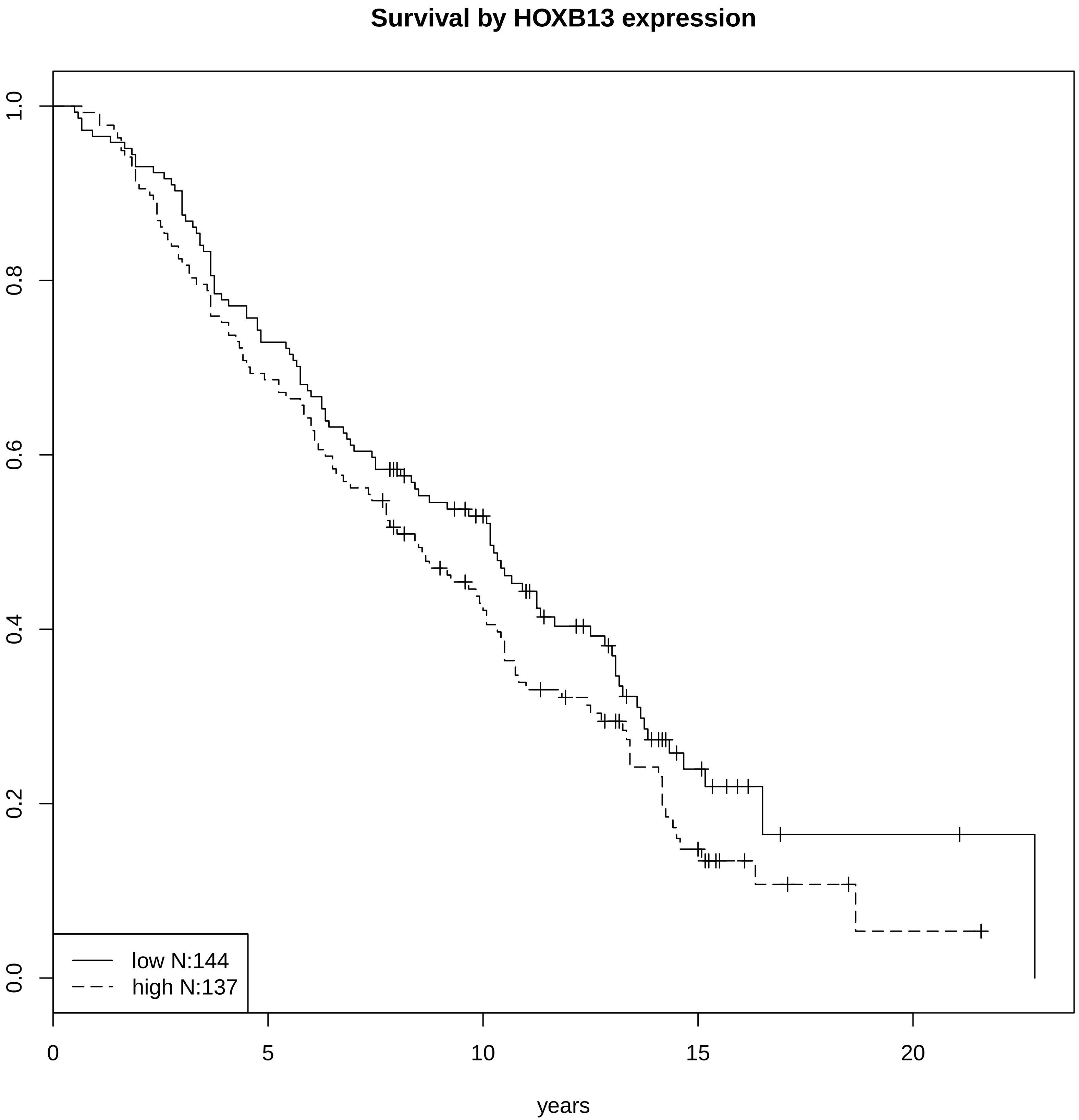
Survival by HOXB13 expression
1.0
0.8
0.6
0.4
0.2
0.0
0
5
10
15
20
low N:144
high N:137
years
log−rank test p−value = 0.093

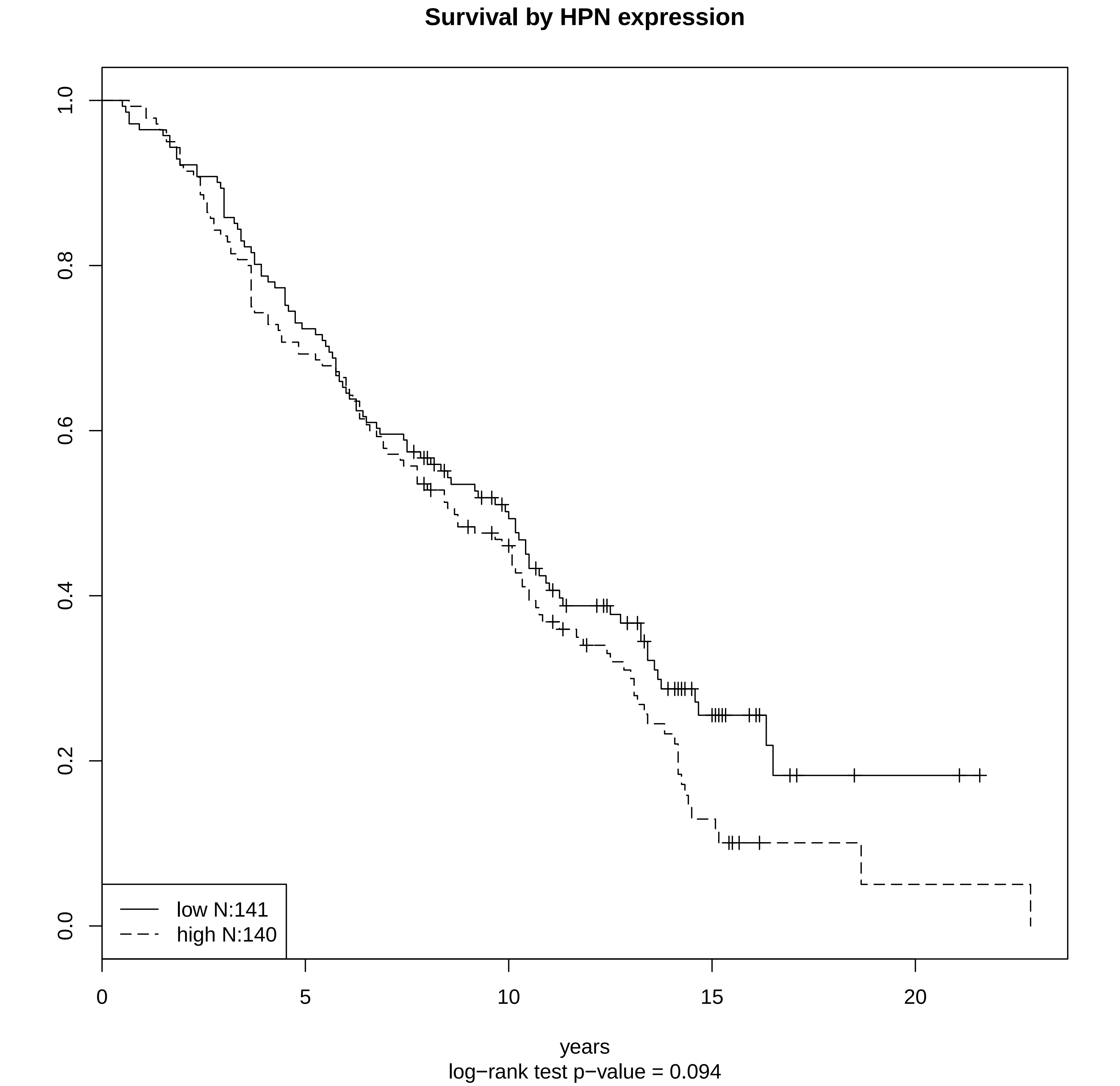

Survival by HPN expression
1.0
0.8
0.6
0.4
0.2
0.0
0
5
10
15
20
years
log−rank test p−value = 0.094
low N:141
high N:140

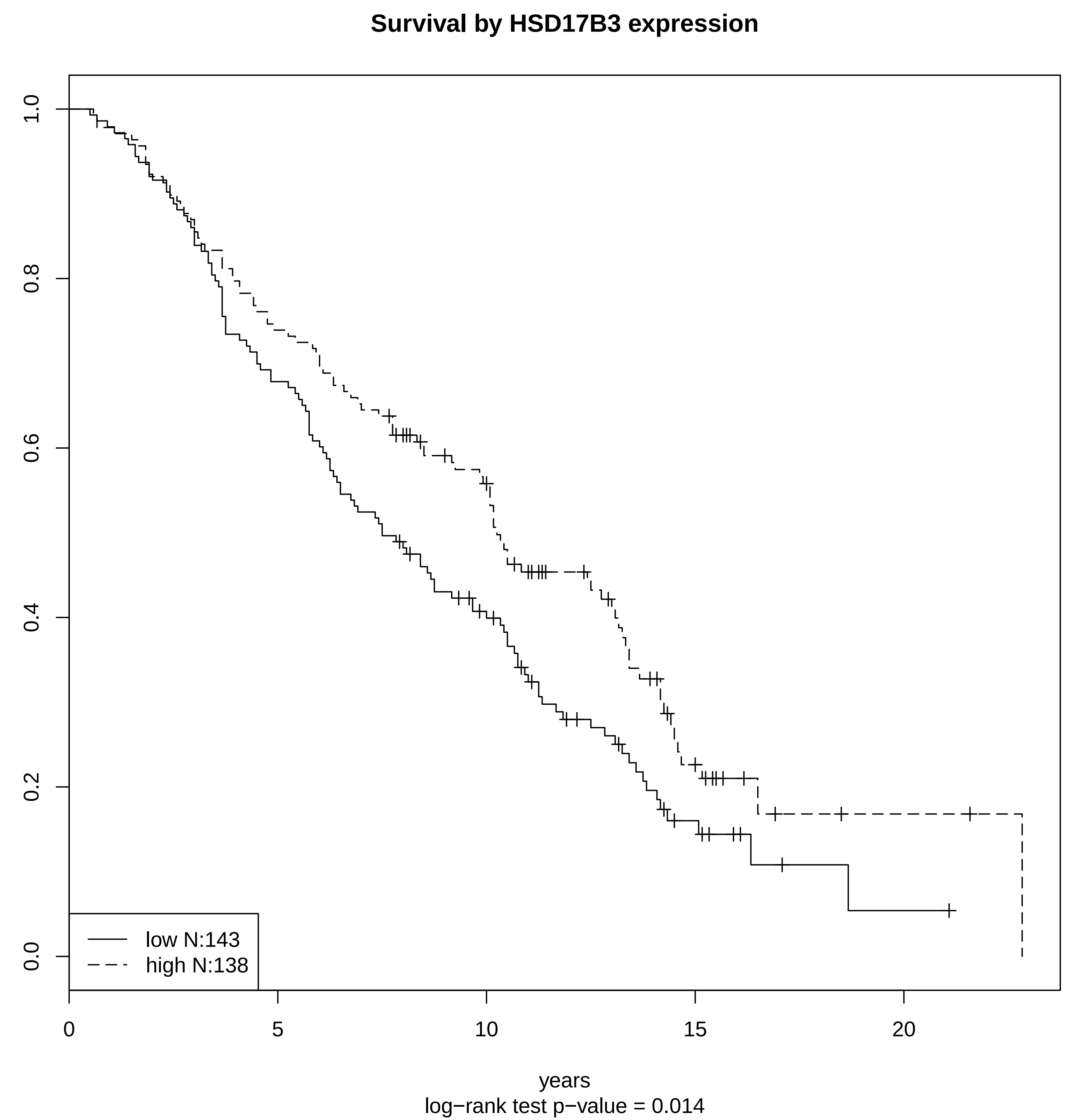

Survival by HSD17B3 expression
1.0
0.8
0.6
0.4
0.2
0.0
low N:143
high N:138
0
5
10
15
20
years
log−rank test p−value = 0.014

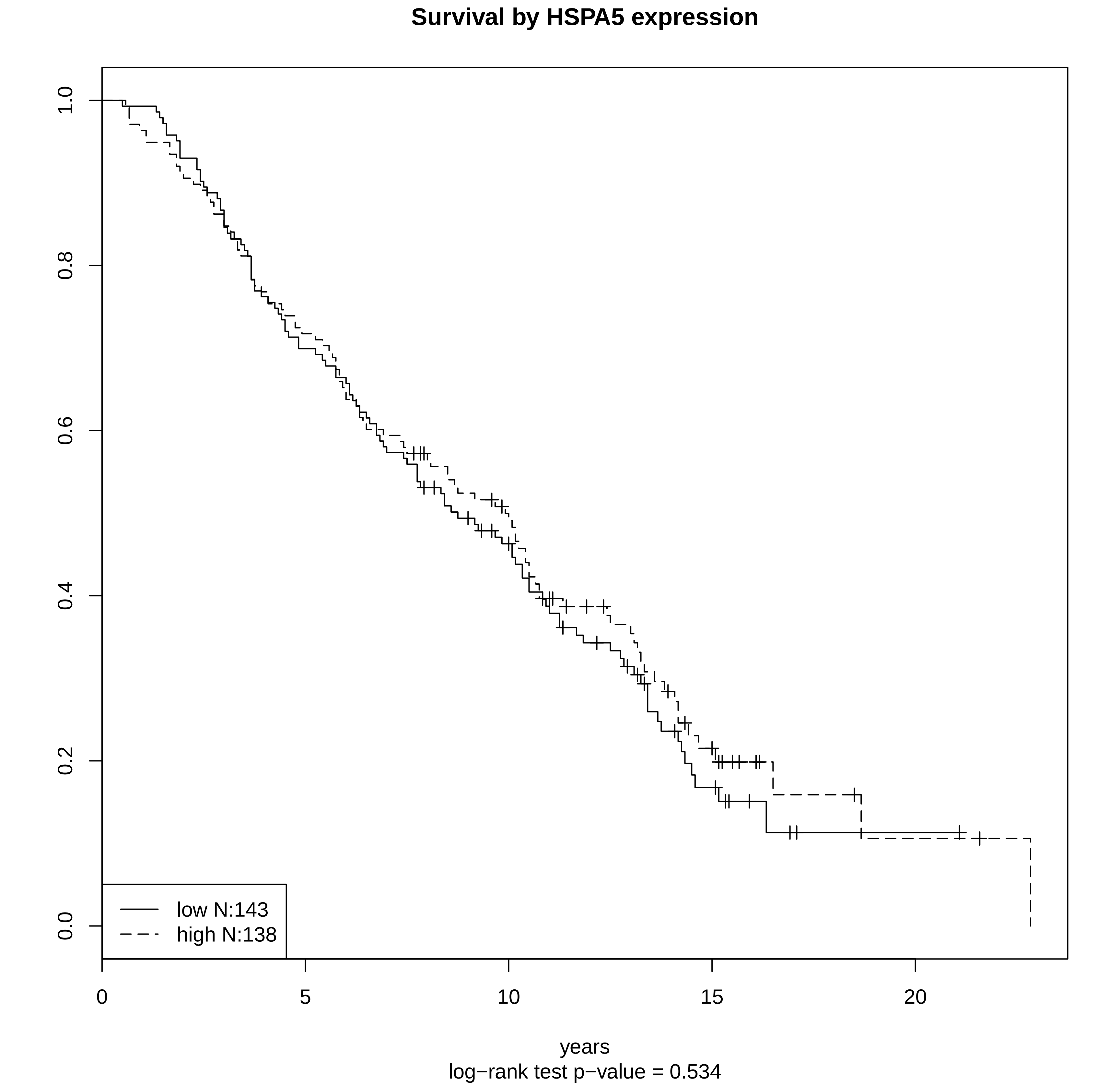

Survival by HSPA5 expression
1.0
0.8
0.6
0.4
0.2
0.0
0
5
10
15
20
years
log−rank test p−value = 0.534
low N:143
high N:138

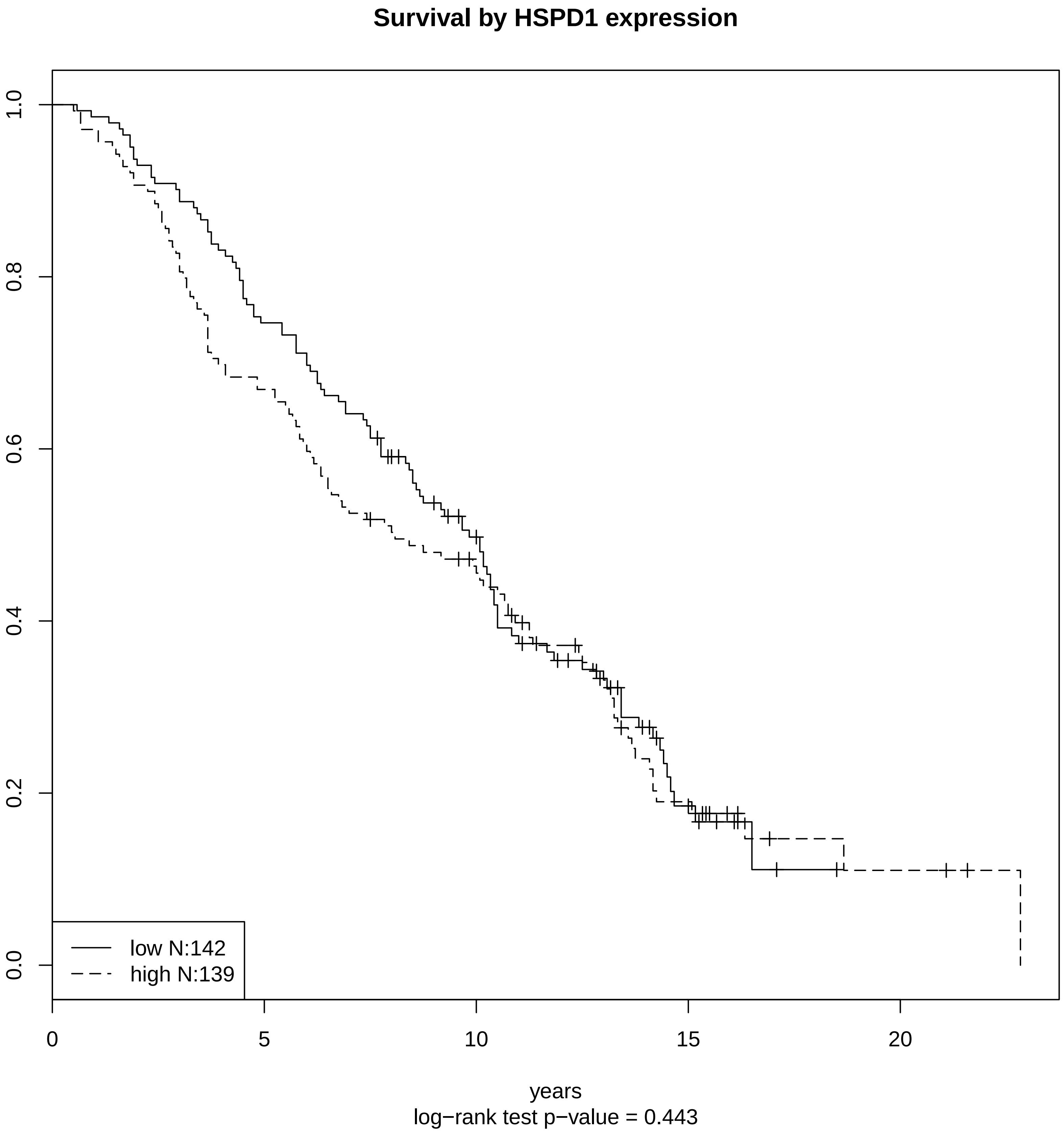
Survival by HSPD1 expression
1.0
0.8
0.6
0.4
0.2
0.0
0
5
10
15
20
low N:142
high N:139
years
log−rank test p−value = 0.443

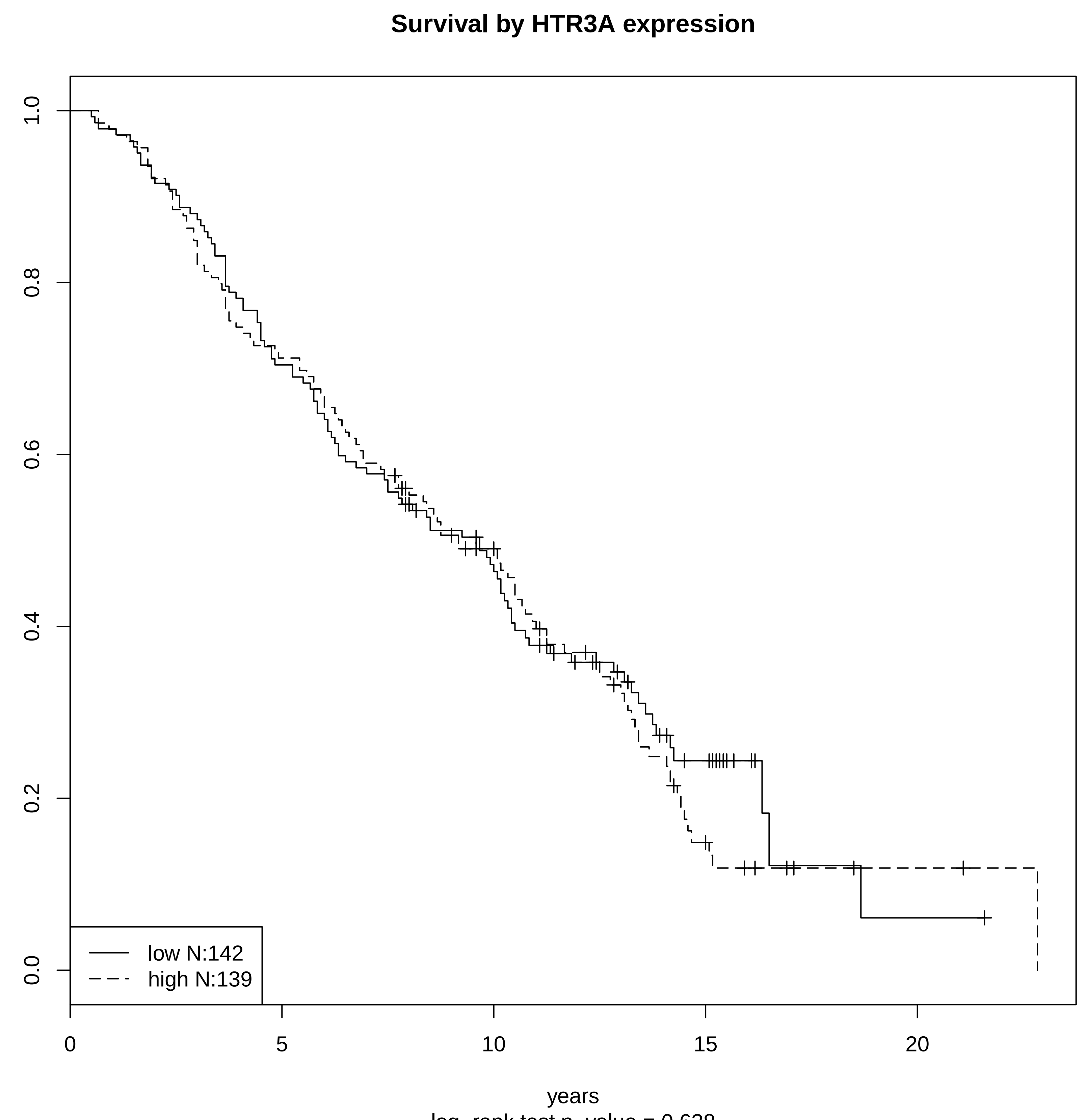

Survival by HTR3A expression
1.0
0.8
0.6
0.4
0.2
0.0
0
5
10
15
20
low N:142
high N:139
years
log−rank test p−value = 0.638

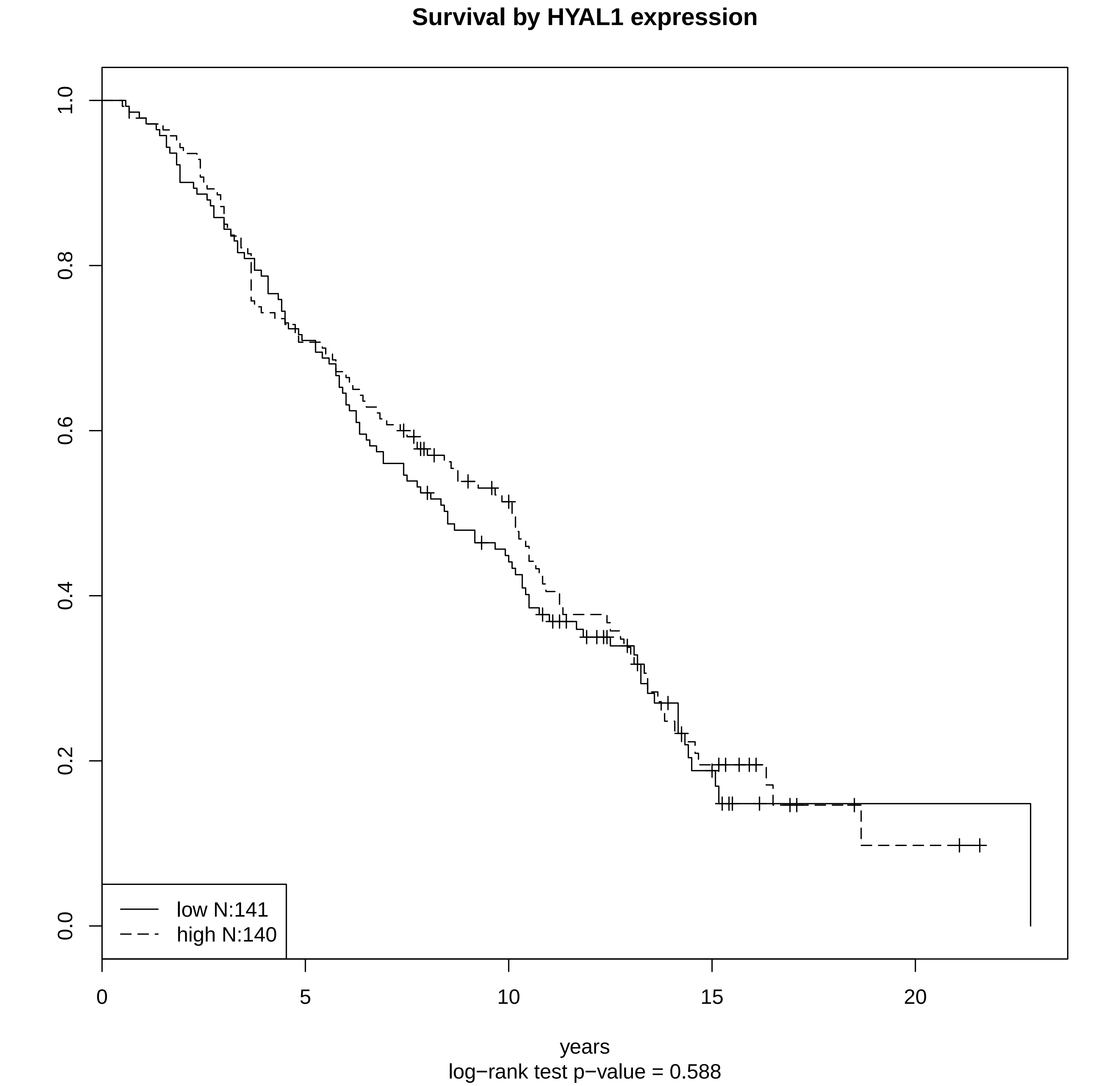
Survival by HYAL1 expression
1.0
0.8
0.6
0.4
0.2
0.0
0
5
10
15
20
years
log−rank test p−value = 0.588
low N:141
high N:140

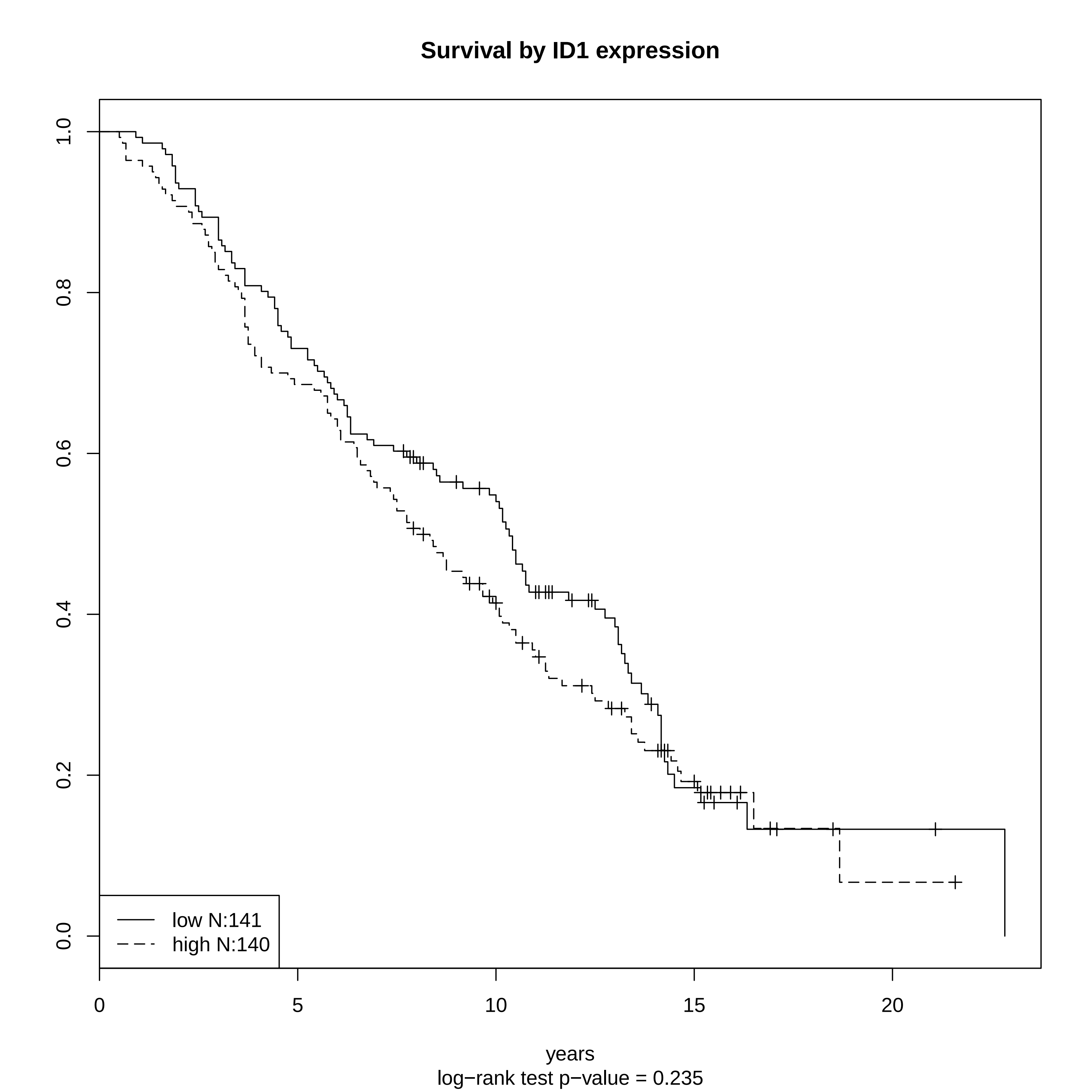
Survival by ID1 expression
1.0
0.8
0.6
0.4
0.2
0.0
0
5
10
15
20
low N:141
high N:140
years
log−rank test p−value = 0.235

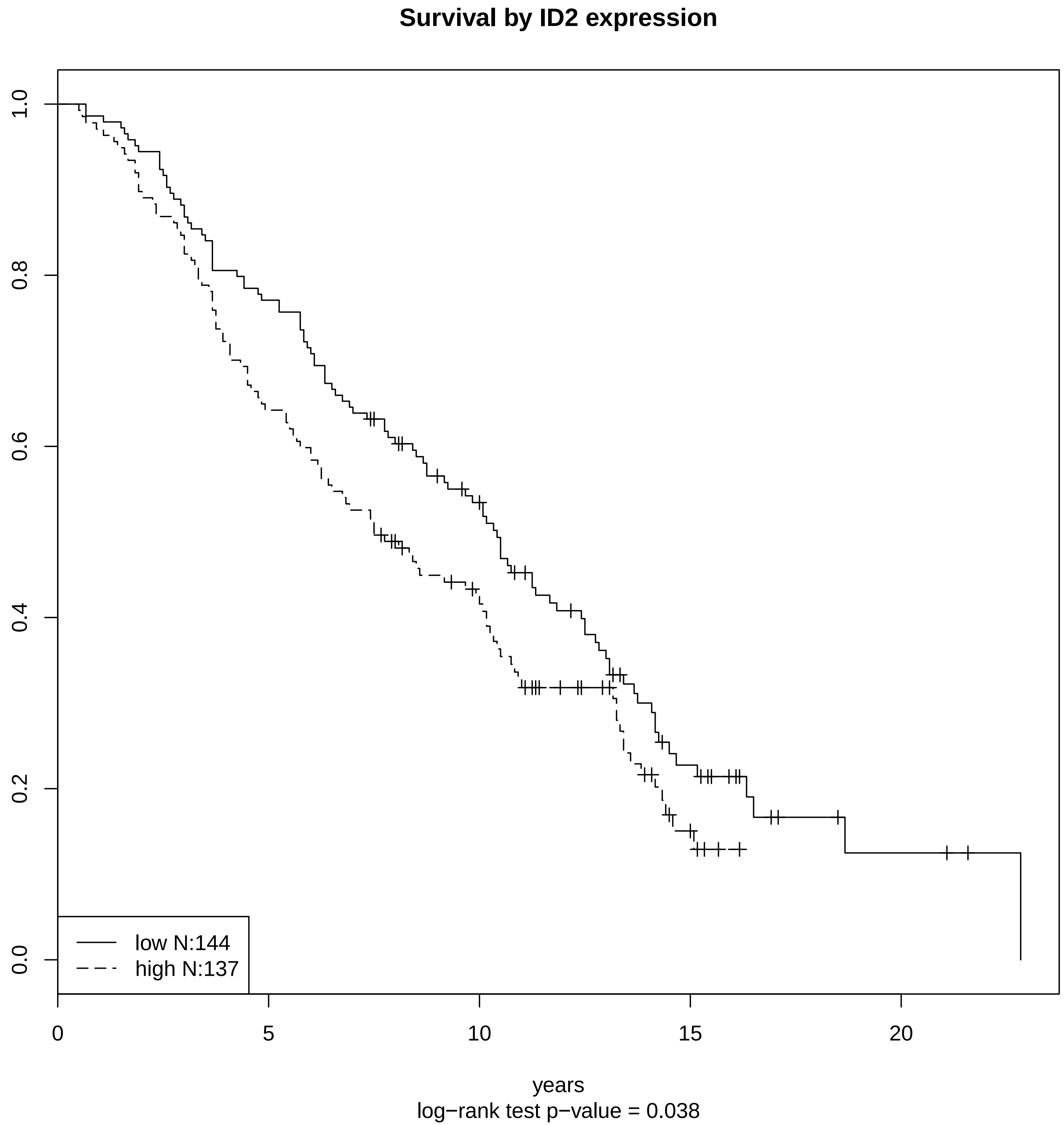
Survival by ID2 expression
1.0
0.8
0.6
0.4
0.2
0.0
0
5
10
15
20
years
log−rank test p−value = 0.038
low N:144
high N:137

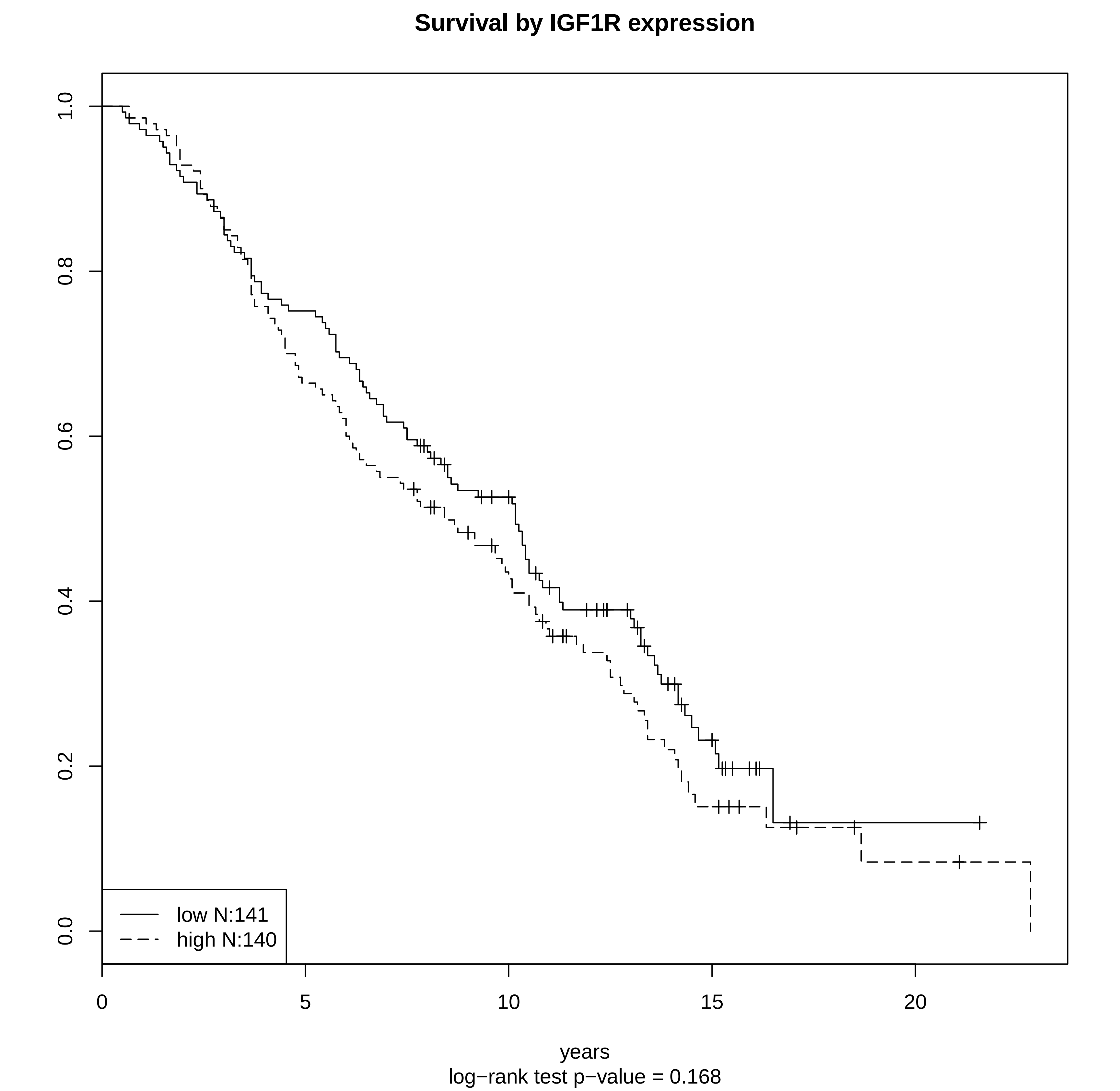
Survival by IGF1R expression
1.0
0.8
0.6
0.4
0.2
0.0
0
5
10
15
20
low N:141
high N:140
years
log−rank test p−value = 0.168

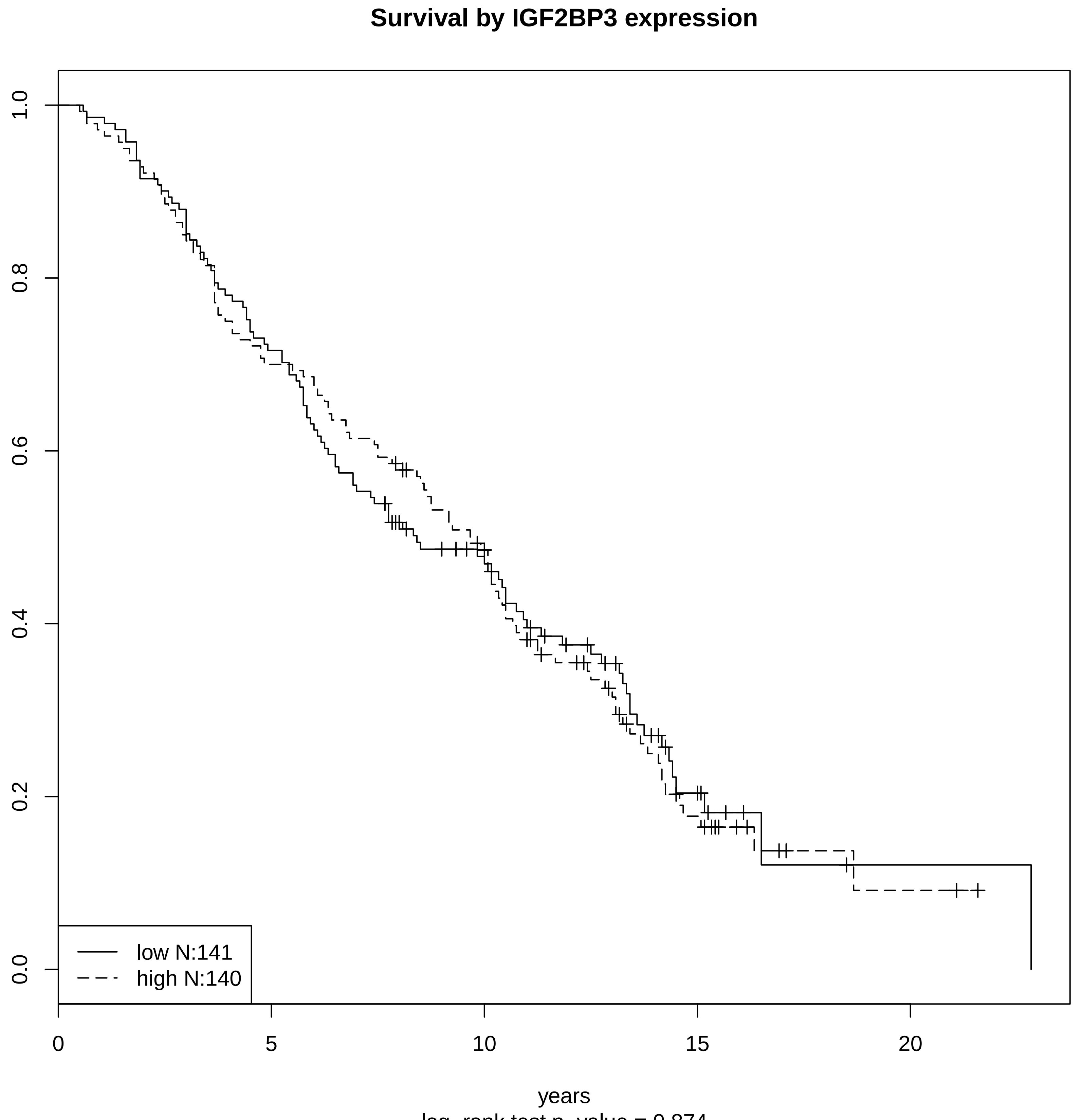
Survival by IGF2BP3 expression
1.0
0.8
0.6
0.4
0.2
0.0
0
5
10
15
20
years
low N:141
high N:140
log−rank test p−value = 0.874

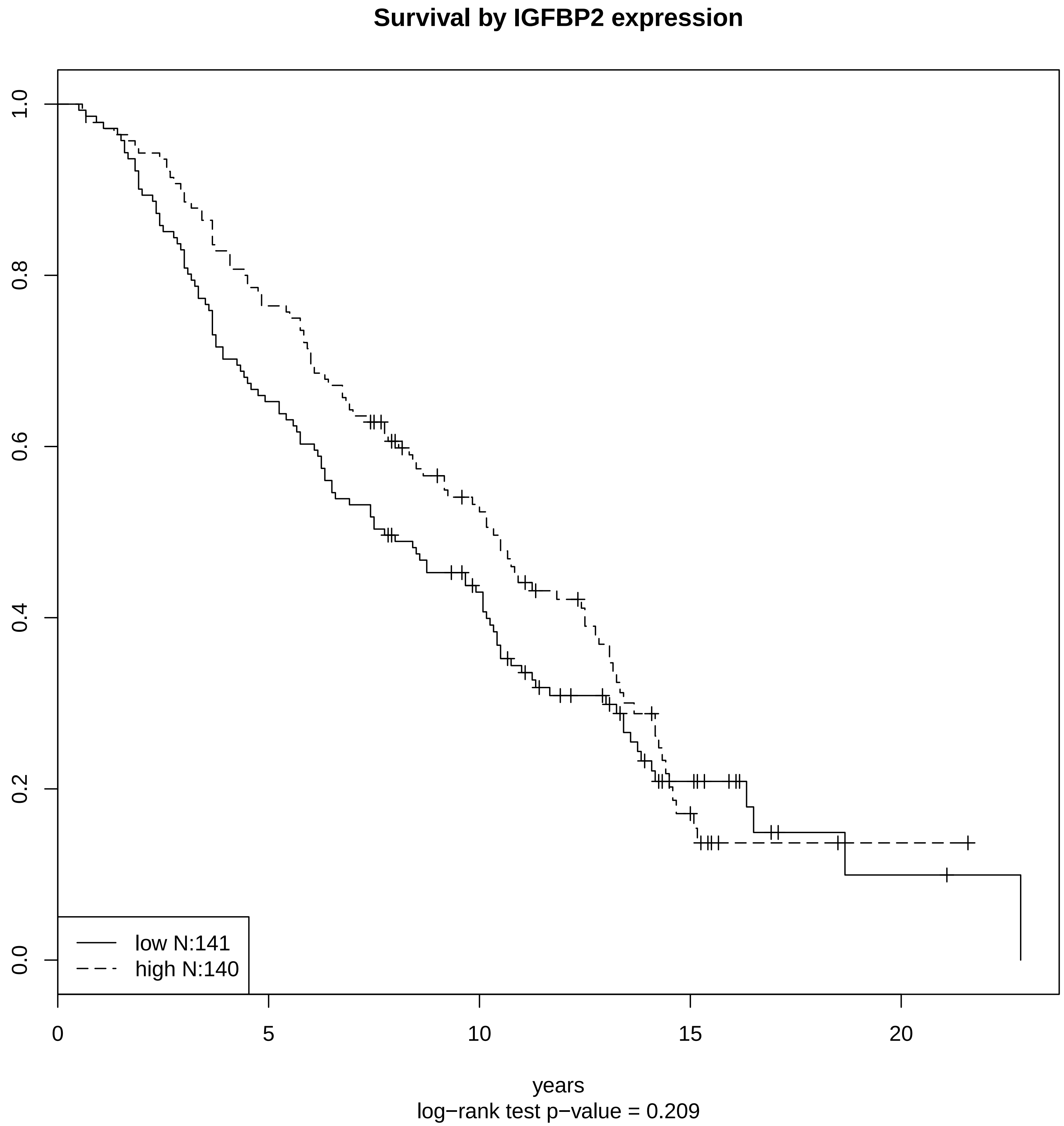

Survival by IGFBP2 expression
1.0
0.8
0.6
0.4
0.2
0.0
0
5
10
15
20
years
log−rank test p−value = 0.209
low N:141
high N:140

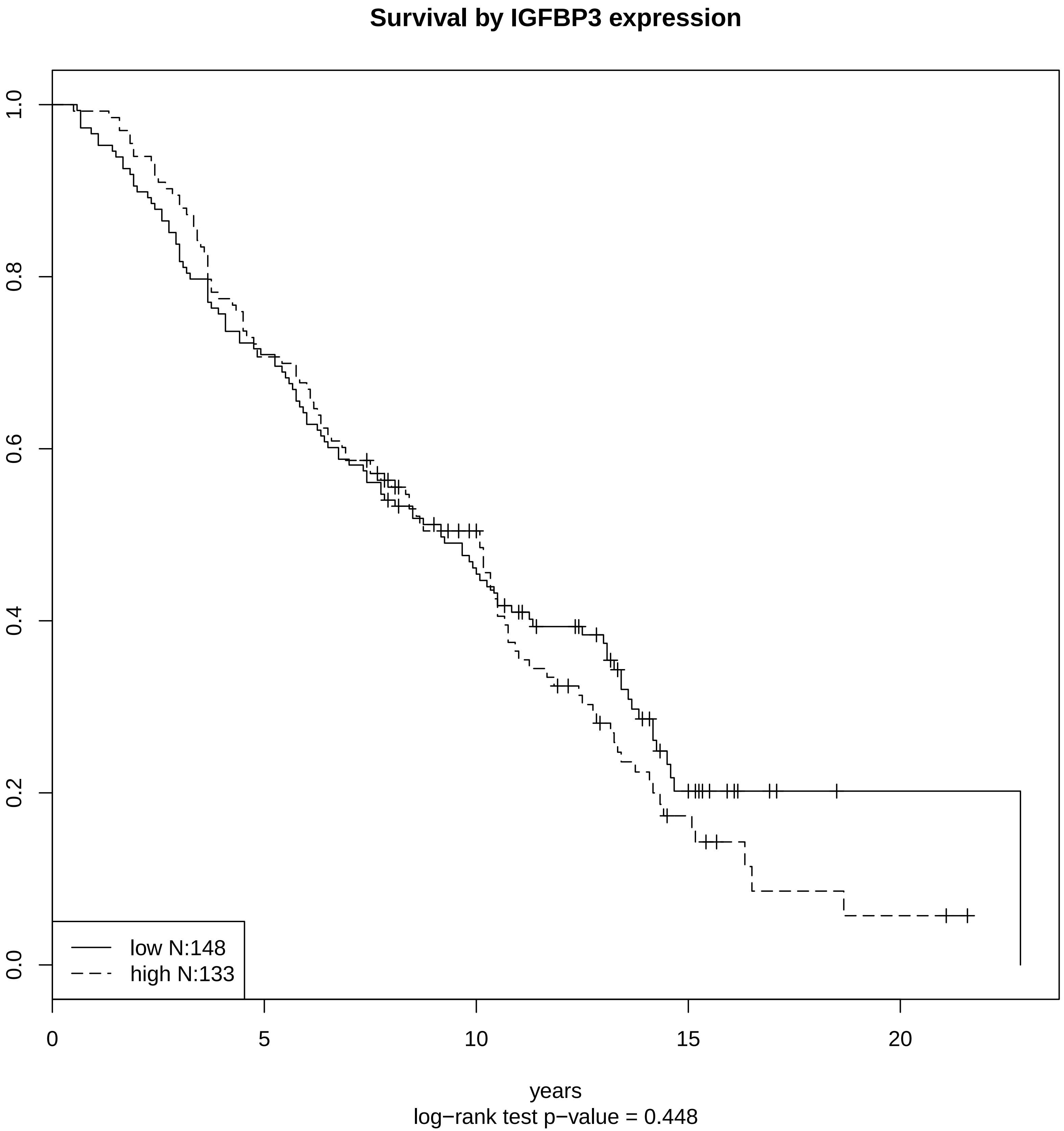

Survival by IGFBP3 expression
1.0
0.8
0.6
0.4
0.2
0.0
0
5
10
15
20
low N:148
high N:133
years
log−rank test p−value = 0.448

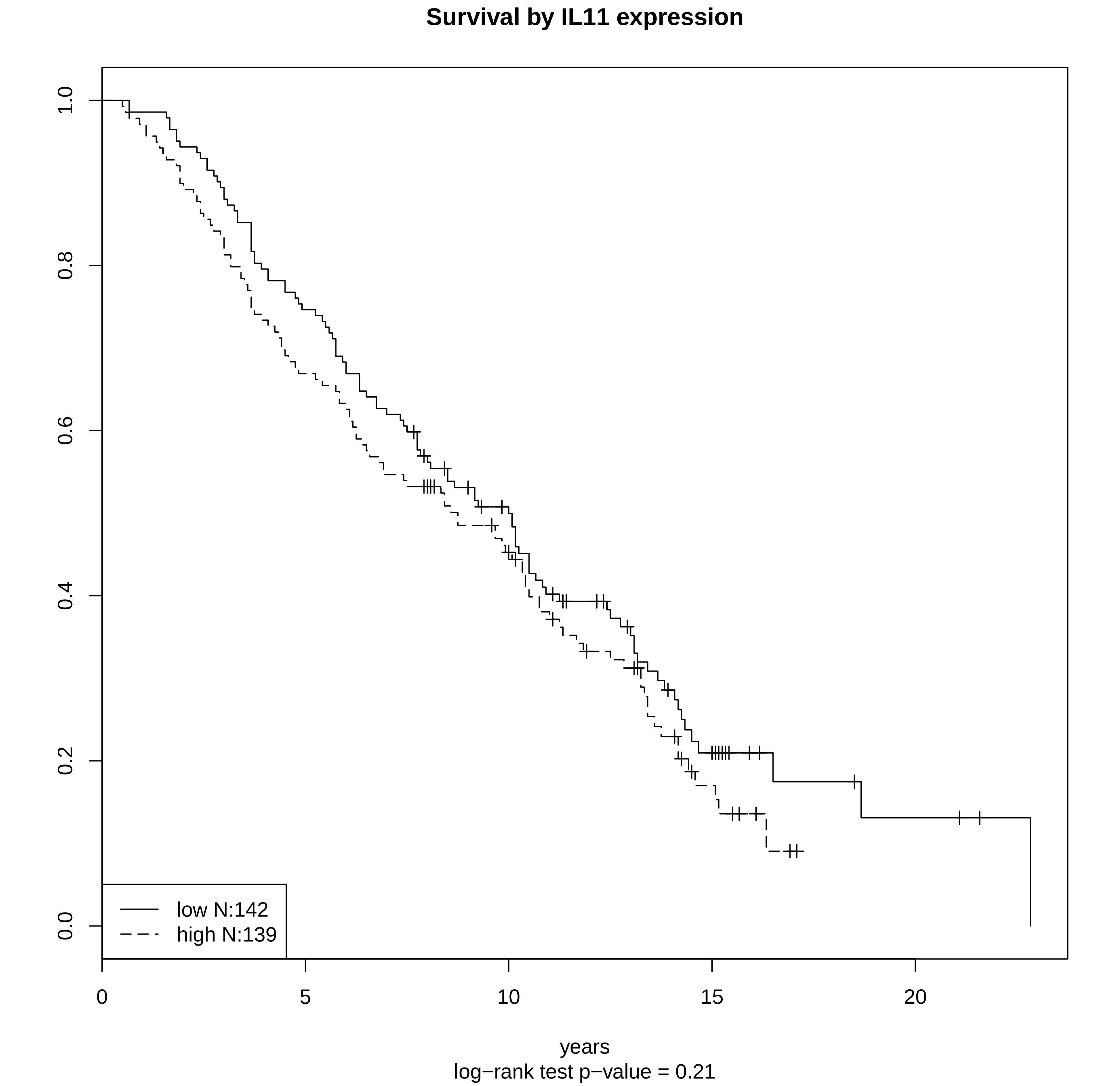

Survival by IL11 expression
1.0
0.8
0.6
0.4
0.2
0.0
0
5
10
15
20
low N:142
high N:139
years
log−rank test p−value = 0.21

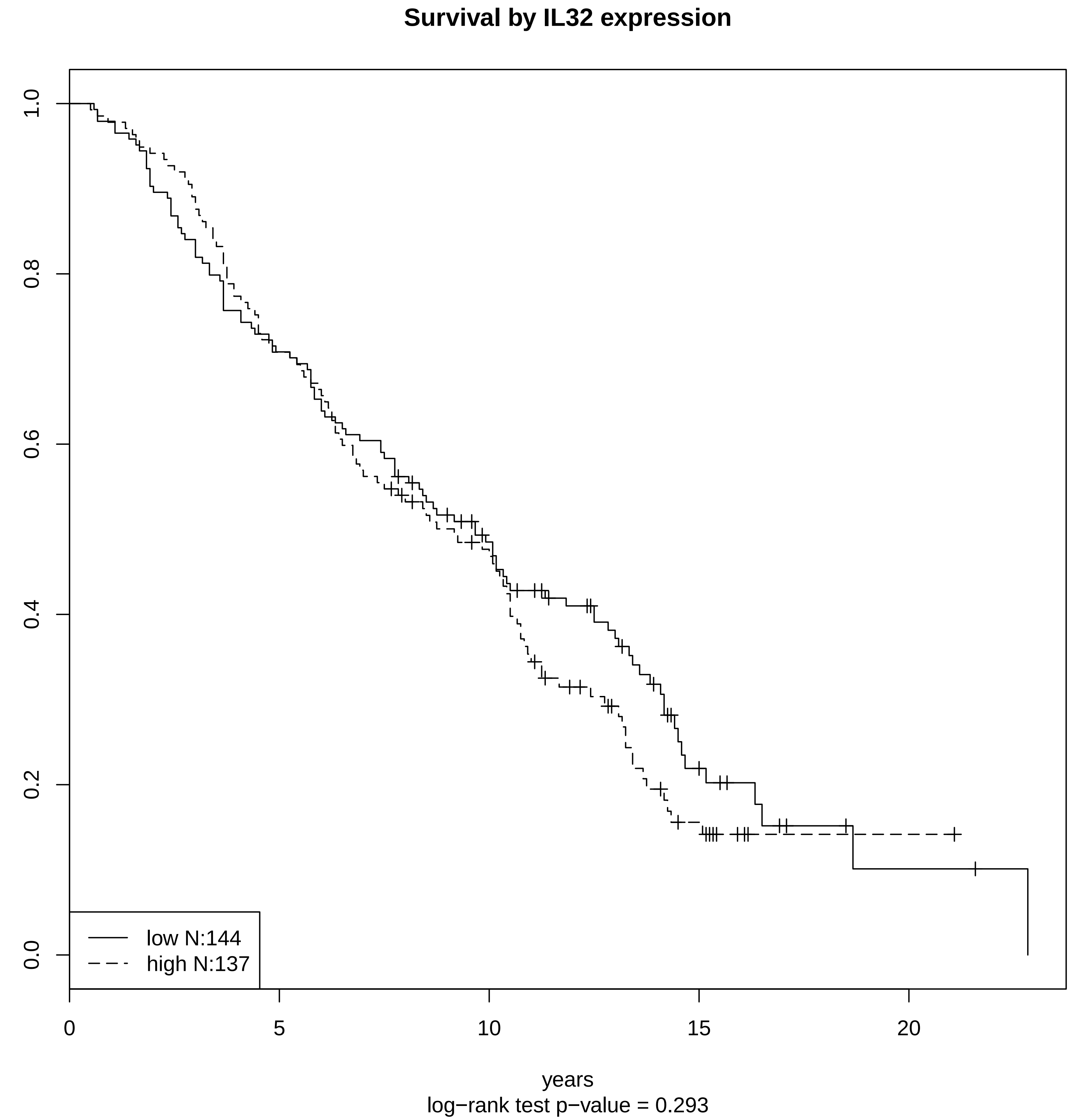
Survival by IL32 expression
1.0
0.8
0.6
0.4
0.2
0.0
low N:144
high N:137
0
5
10
15
20
years
log−rank test p−value = 0.293

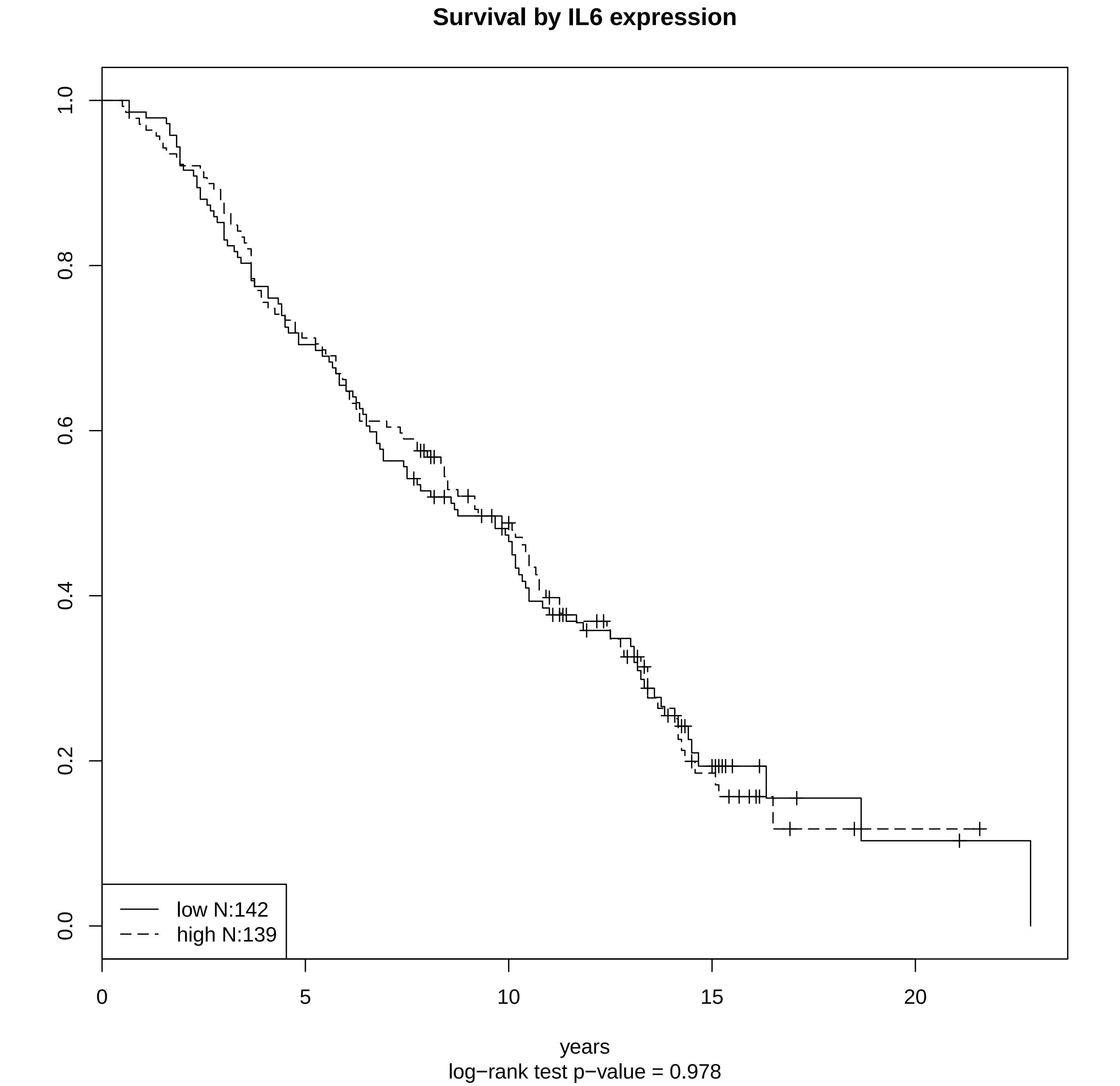

Survival by IL6 expression
1.0
0.8
0.6
0.4
0.2
0.0
0
5
10
15
20
low N:142
high N:139
years
log−rank test p−value = 0.978

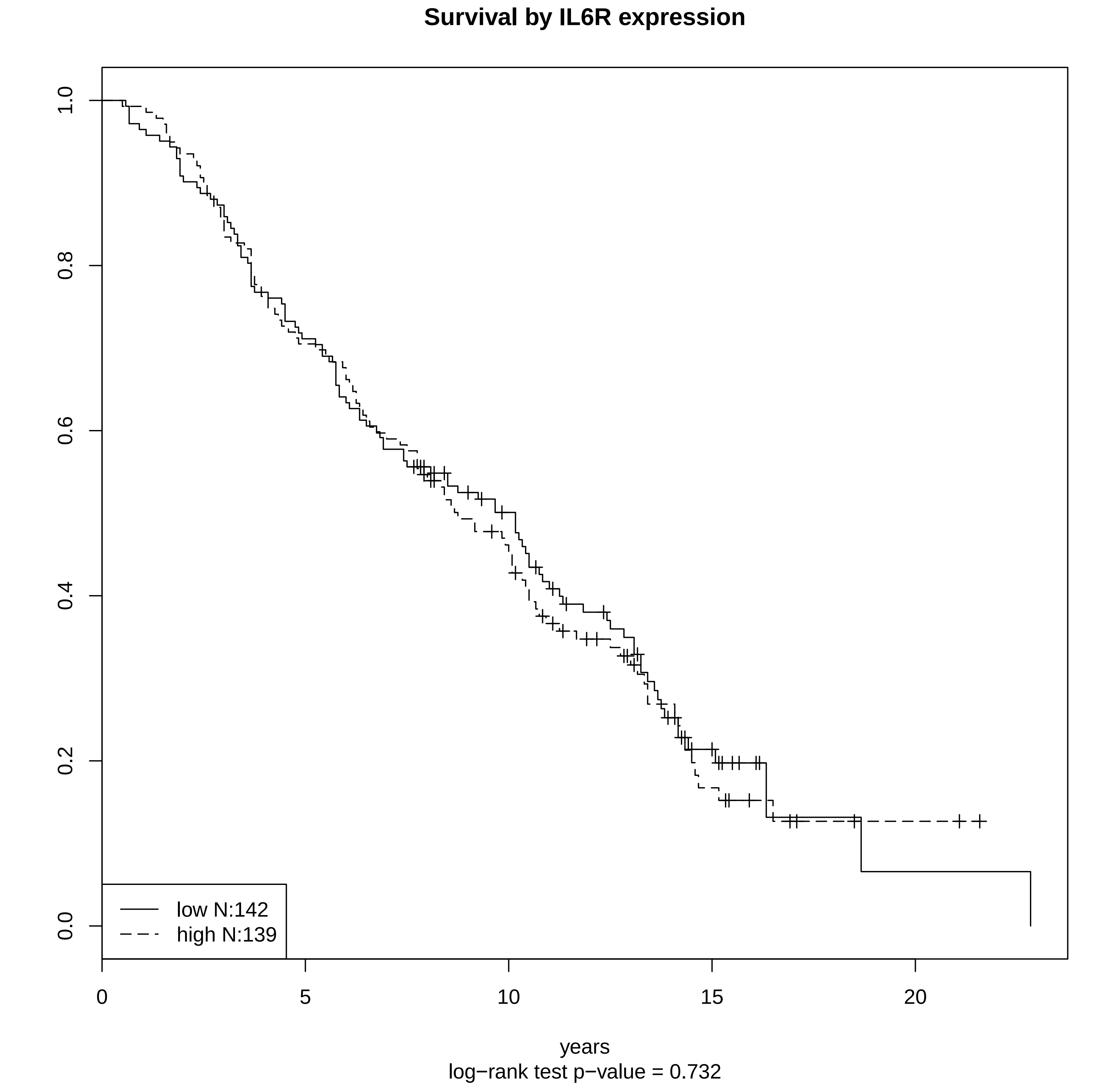

Survival by IL6R expression
1.0
0.8
0.6
0.4
0.2
0.0
0
5
10
15
20
years
log−rank test p−value = 0.732
low N:142
high N:139

Survival by IL8 expression

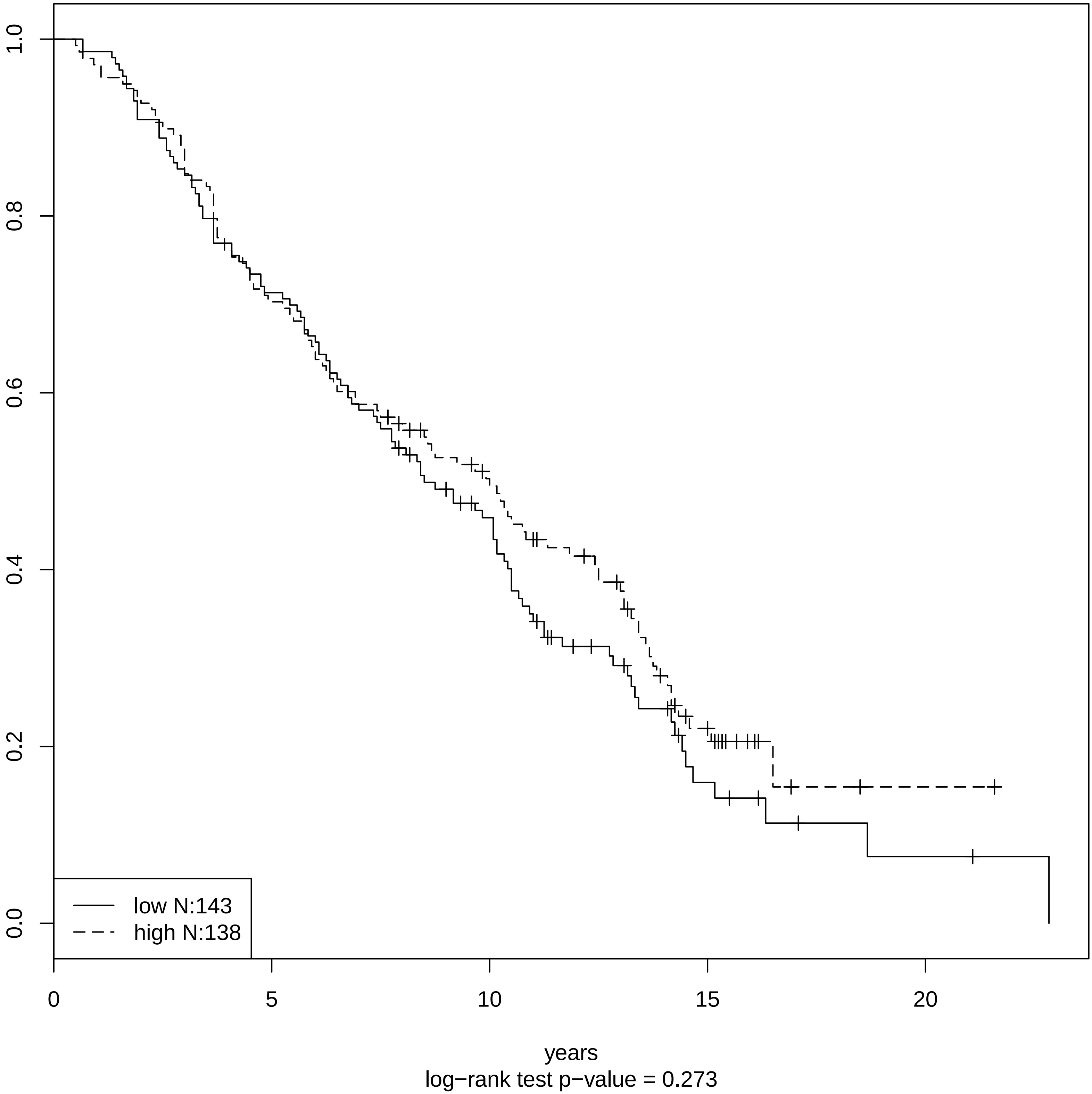

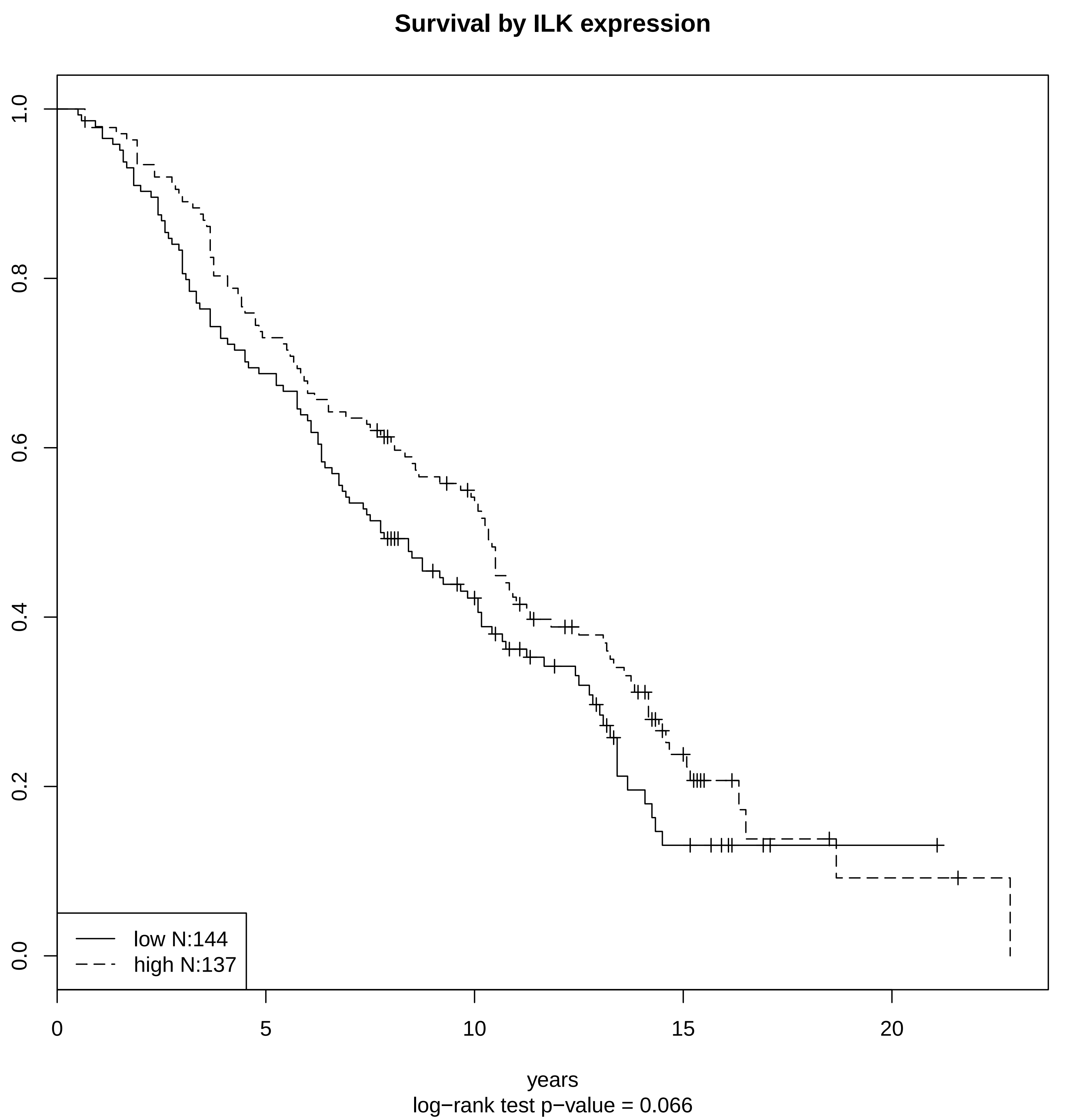
Survival by ILK expression
1.0
0.8
0.6
0.4
0.2
0.0
0
5
10
15
20
low N:144
high N:137
years
log−rank test p−value = 0.066

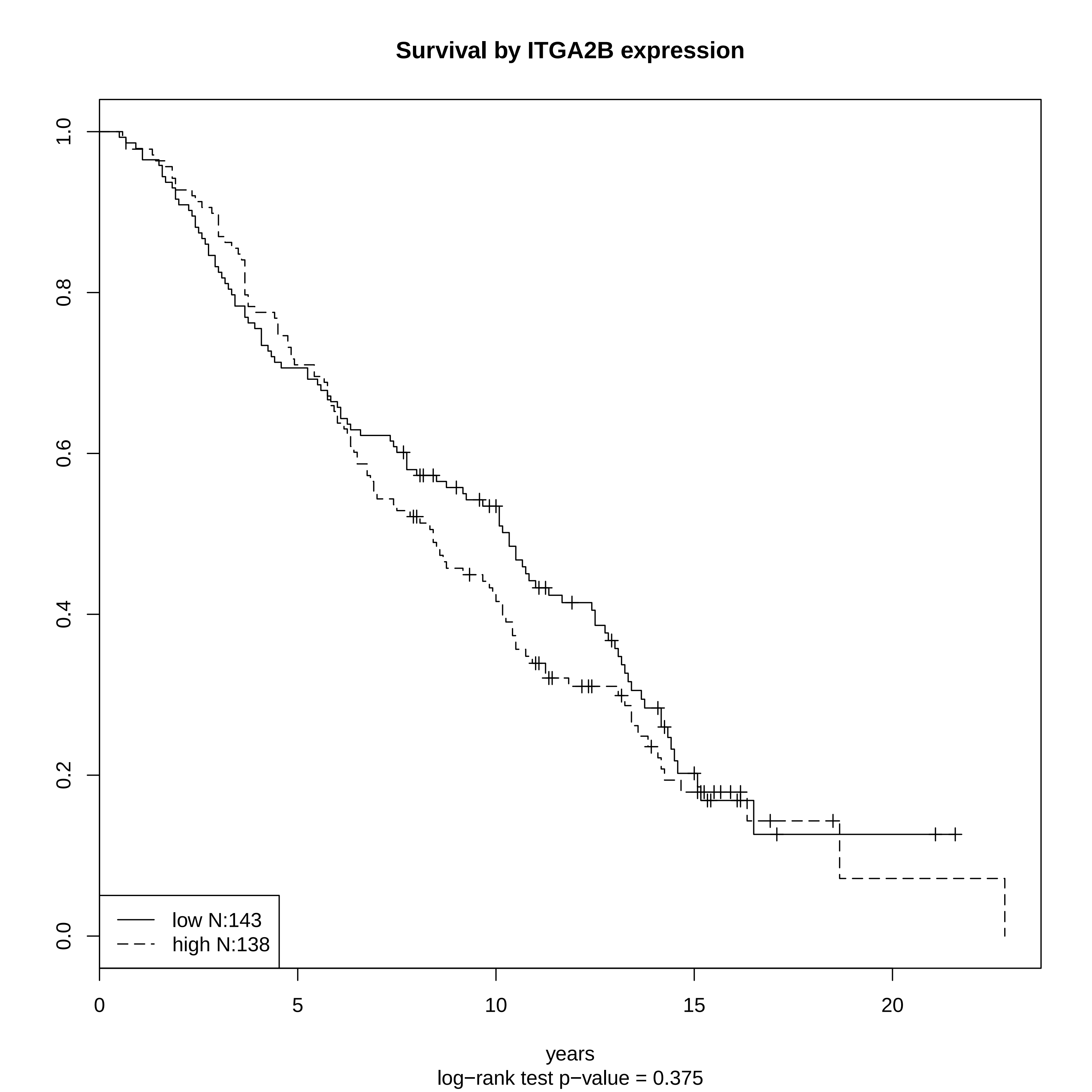
Survival by ITGA2B expression
1.0
0.8
0.6
0.4
0.2
0.0
0
5
10
15
20
low N:143
high N:138
years
log−rank test p−value = 0.375

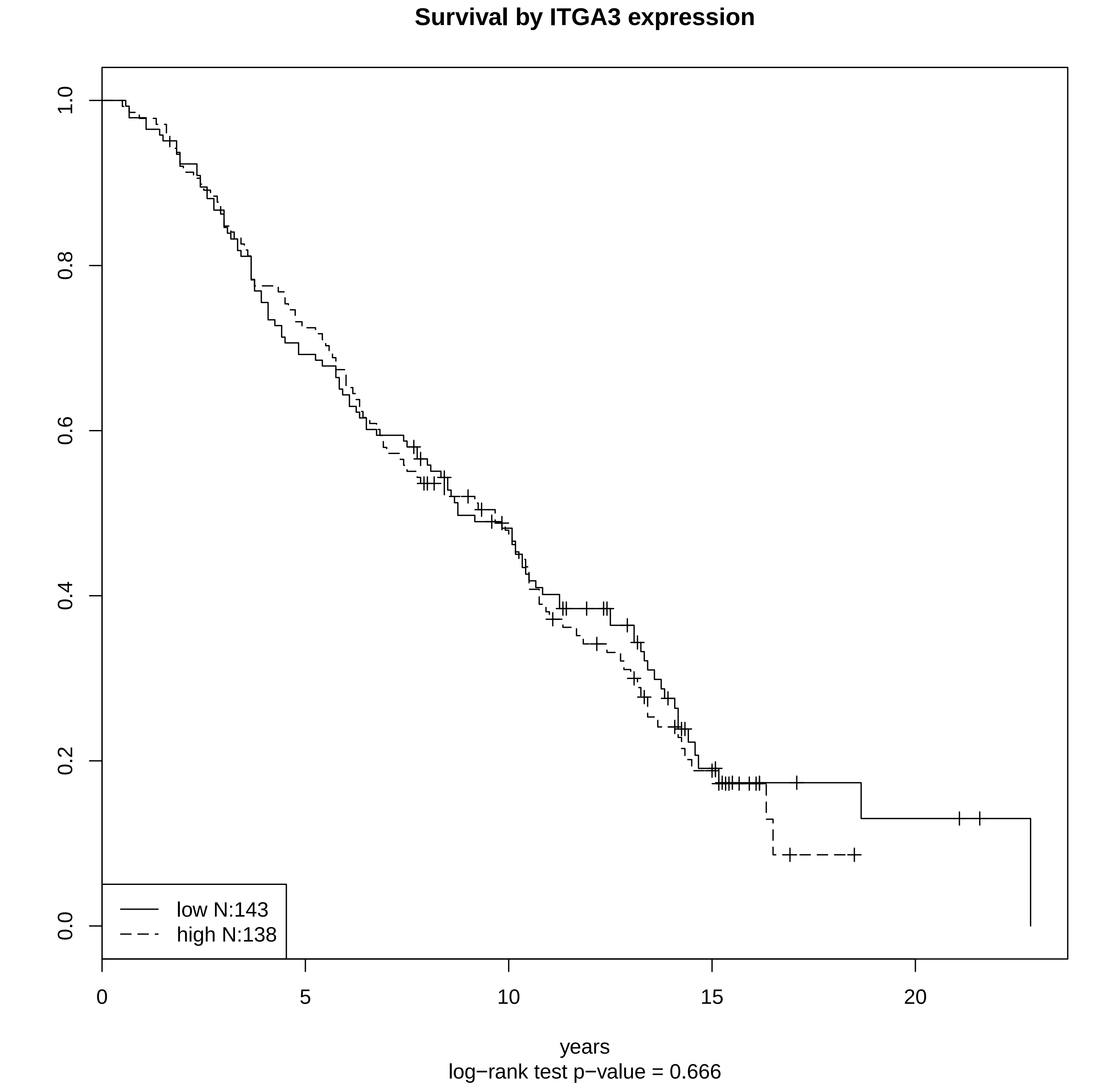
Survival by ITGA3 expression
1.0
0.8
0.6
0.4
0.2
0.0
0
5
10
15
20
low N:143
high N:138
years
log−rank test p−value = 0.666

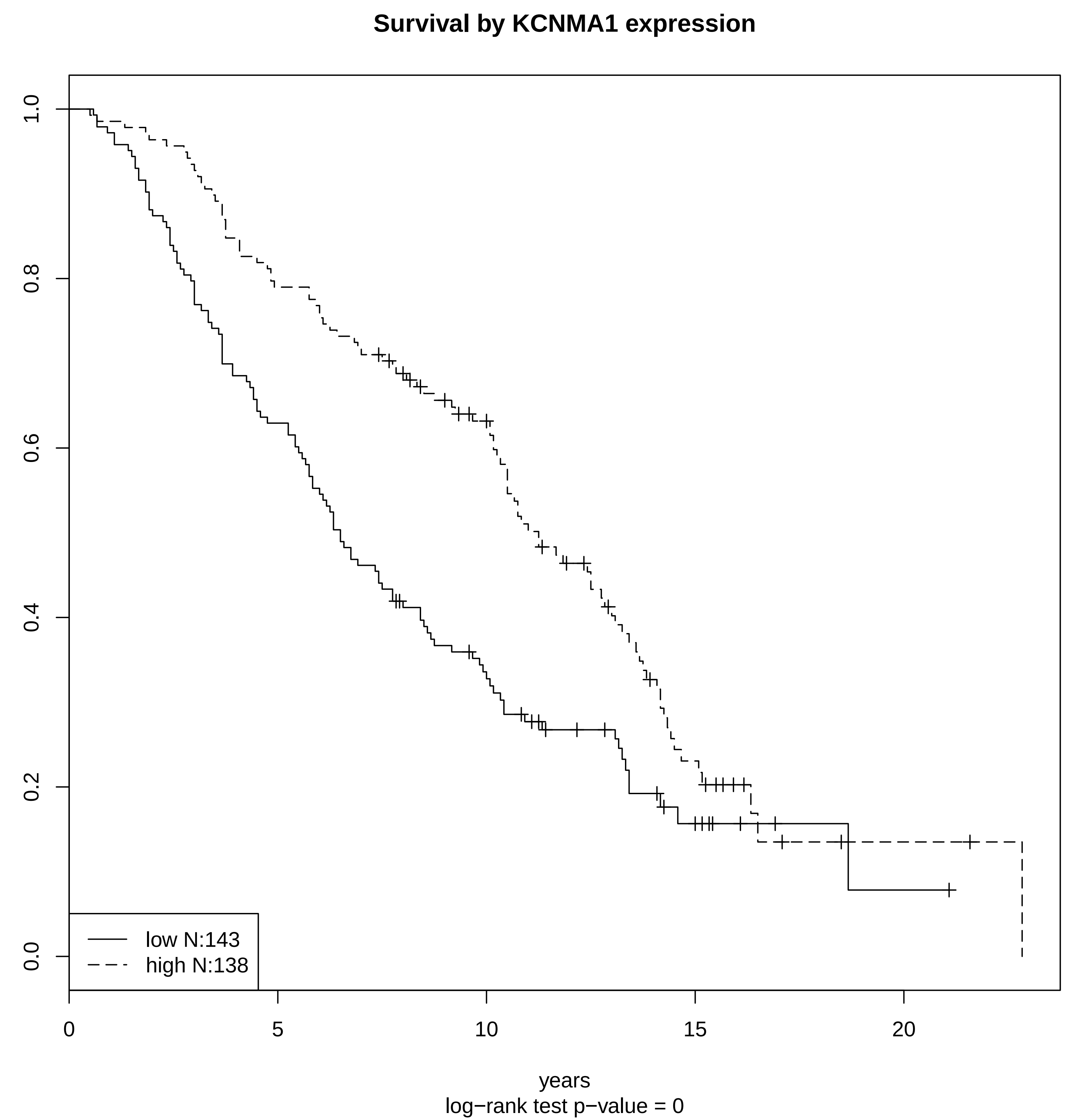
Survival by KCNMA1 expression
1.0
0.8
0.6
0.4
0.2
0.0
0
5
10
15
20
years
log−rank test p−value = 0
low N:143
high N:138

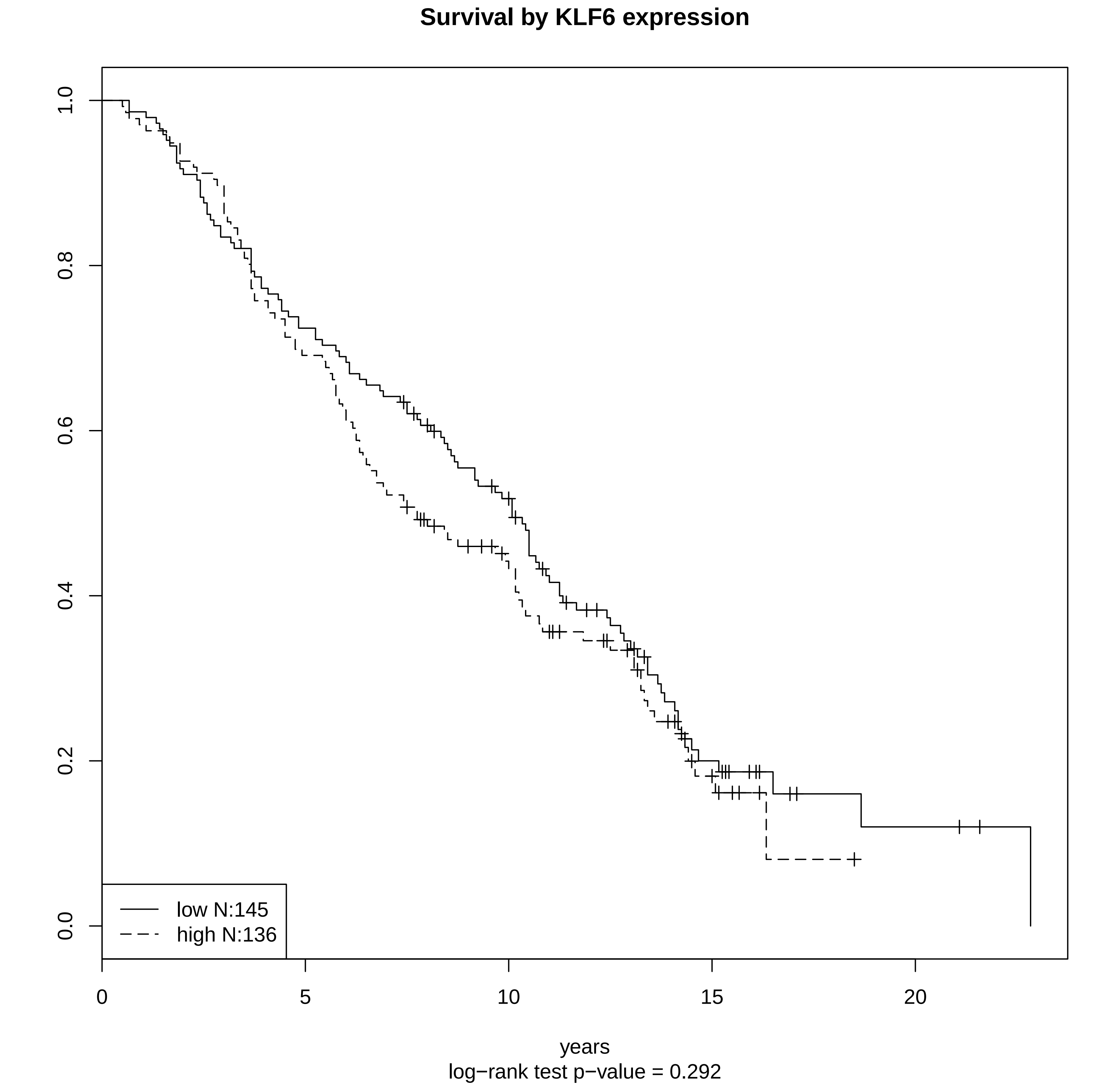

Survival by KLF6 expression
1.0
0.8
0.6
0.4
0.2
0.0
0
5
10
15
20
low N:145
high N:136
years
log−rank test p−value = 0.292

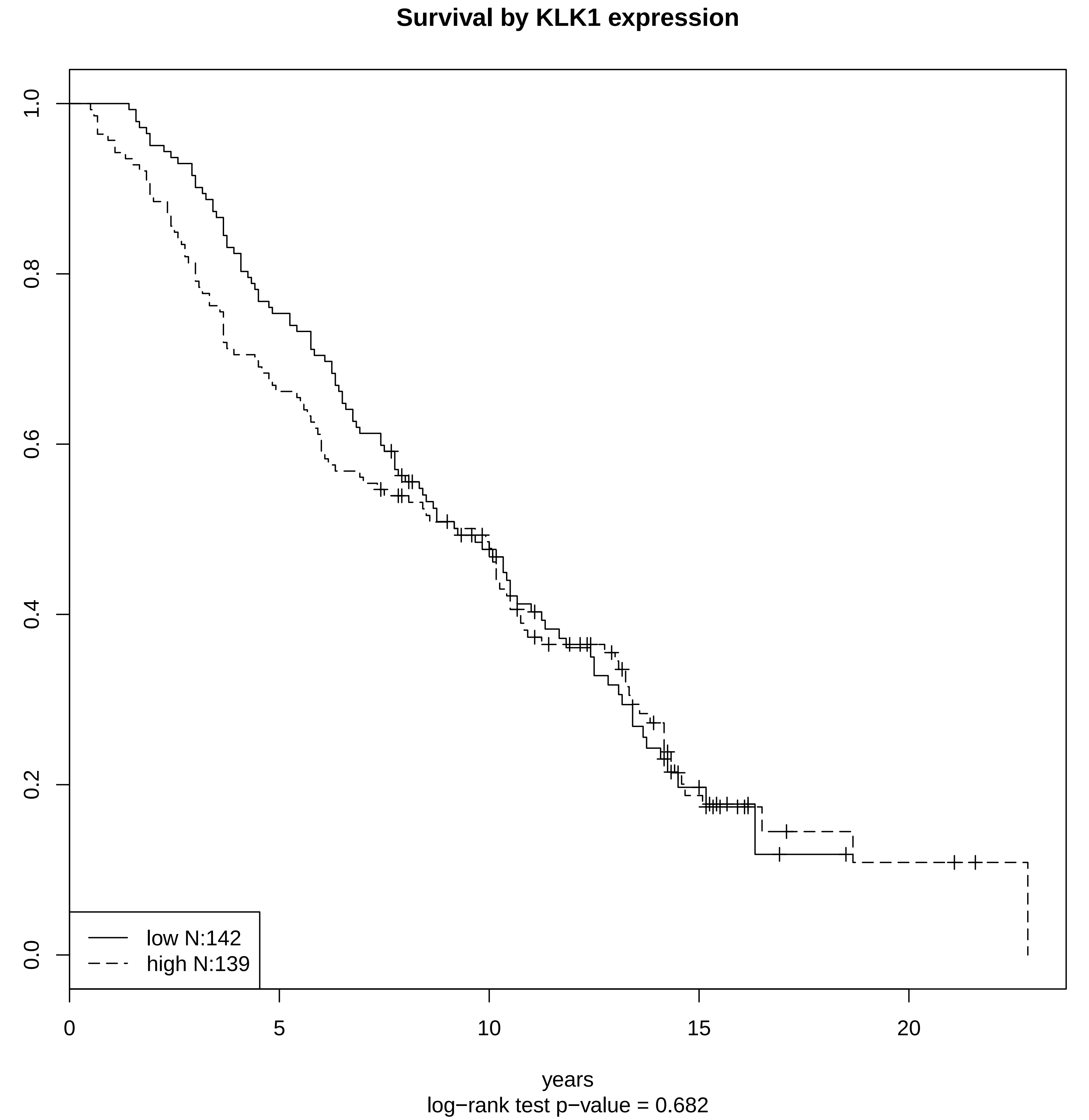

Survival by KLK1 expression
1.0
0.8
0.6
0.4
0.2
0.0
0
5
10
15
20
low N:142
high N:139
years
log−rank test p−value = 0.682

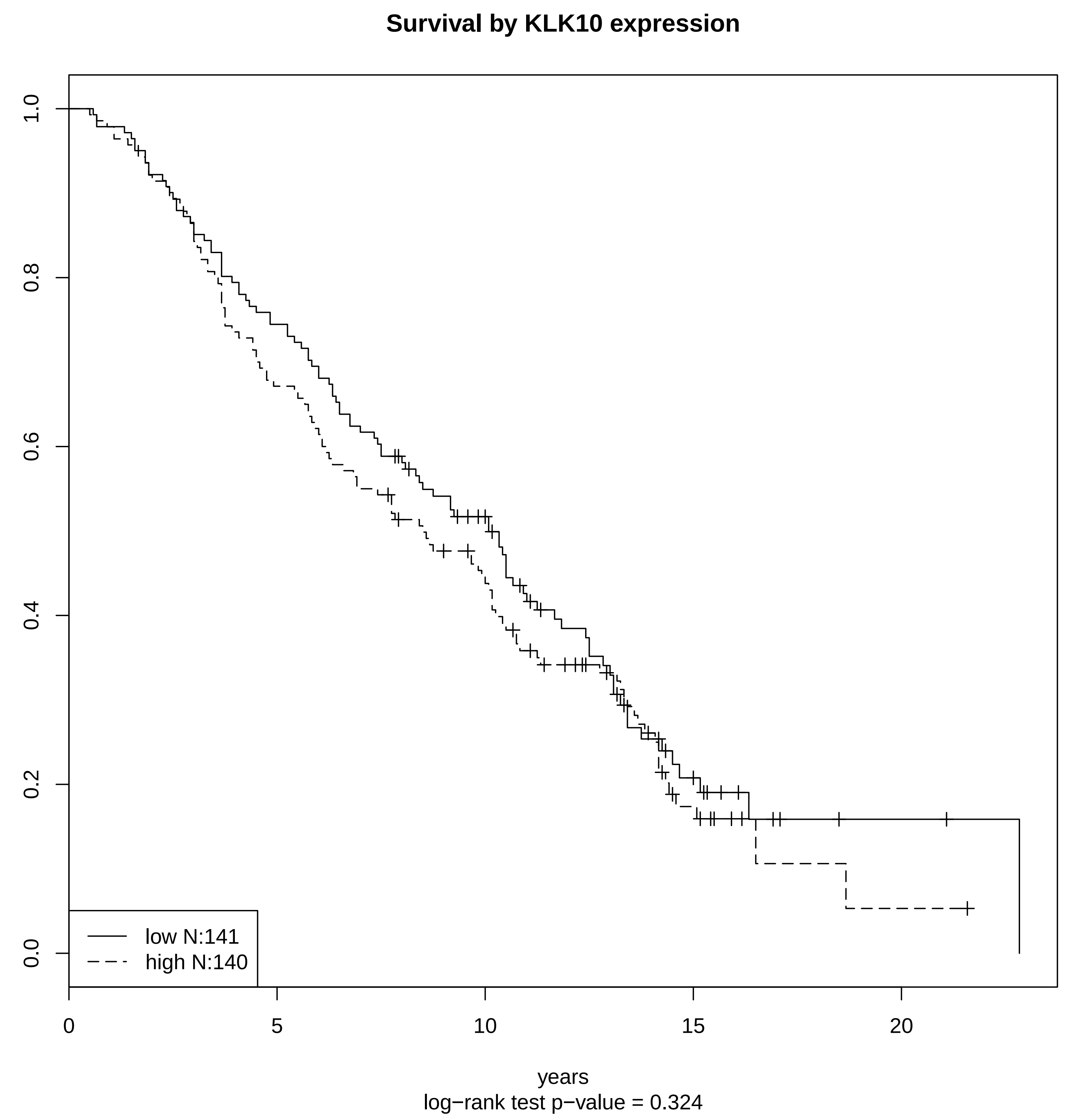

Survival by KLK10 expression
1.0
0.8
0.6
0.4
0.2
0.0
0
5
10
15
20
years
log−rank test p−value = 0.324
low N:141
high N:140

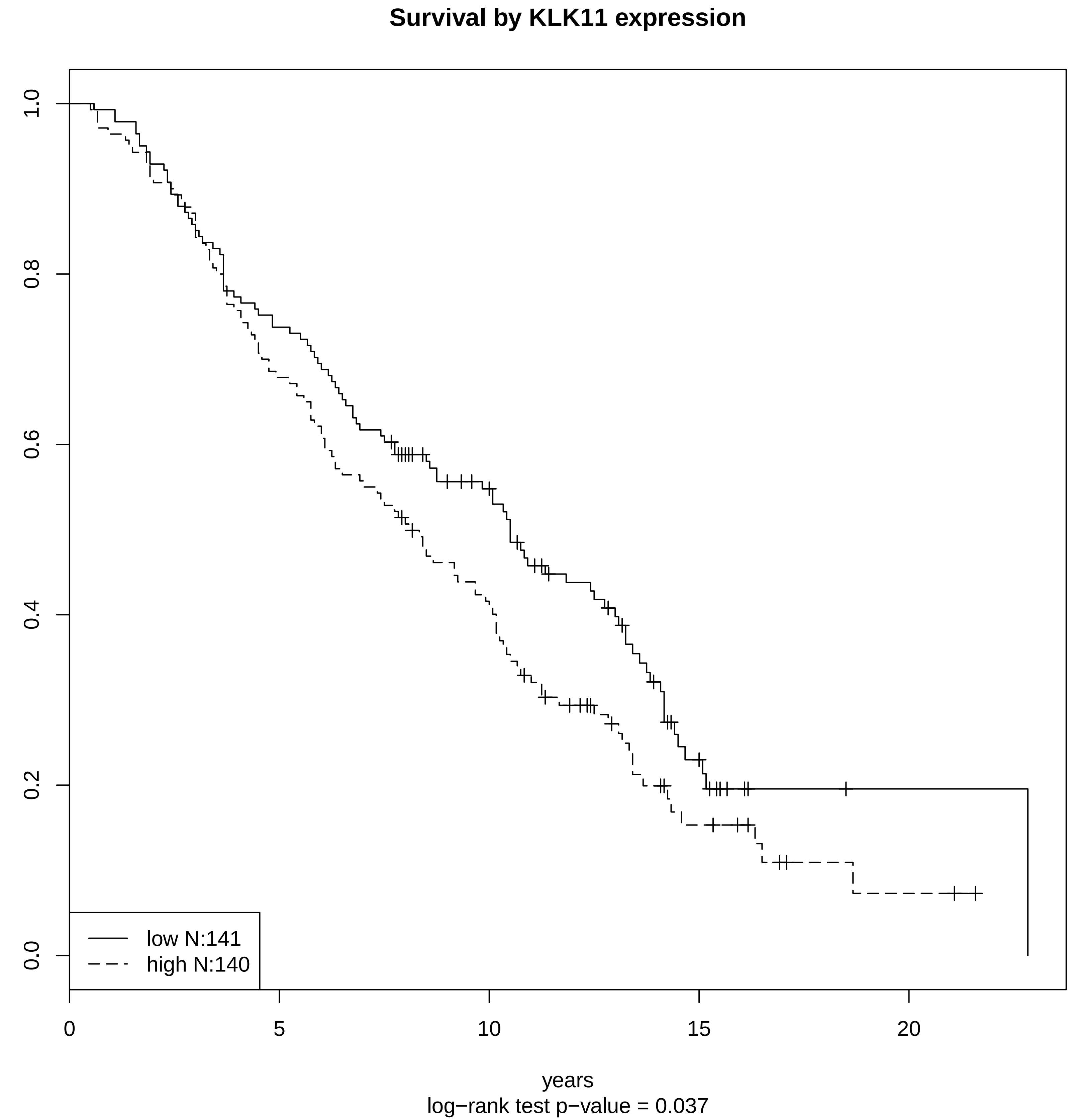

Survival by KLK11 expression
1.0
0.8
0.6
0.4
0.2
0.0
0
5
10
15
20
low N:141
high N:140
years
log−rank test p−value = 0.037

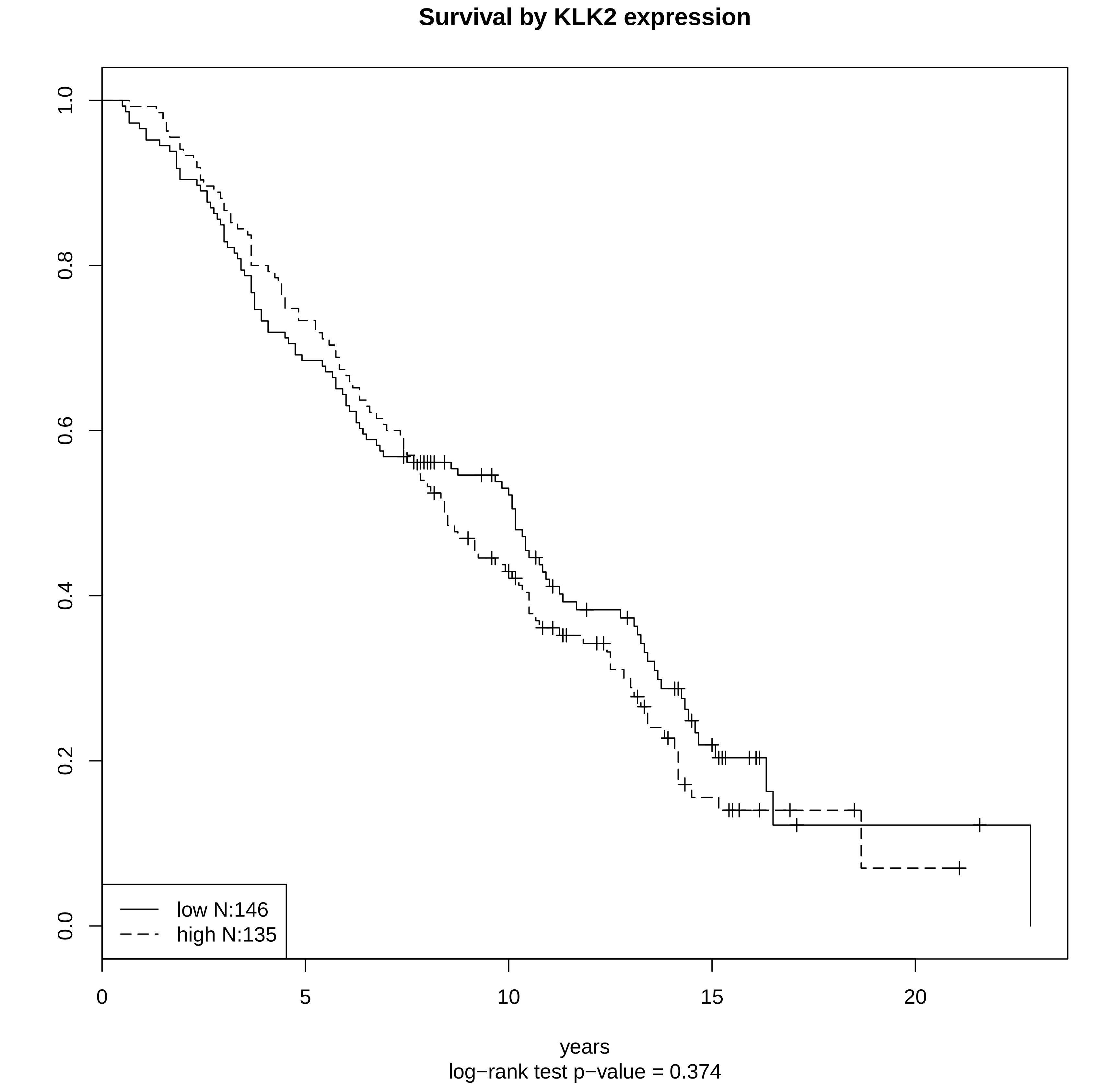

Survival by KLK2 expression
1.0
0.8
0.6
0.4
0.2
0.0
0
5
10
15
20
low N:146
high N:135
years
log−rank test p−value = 0.374

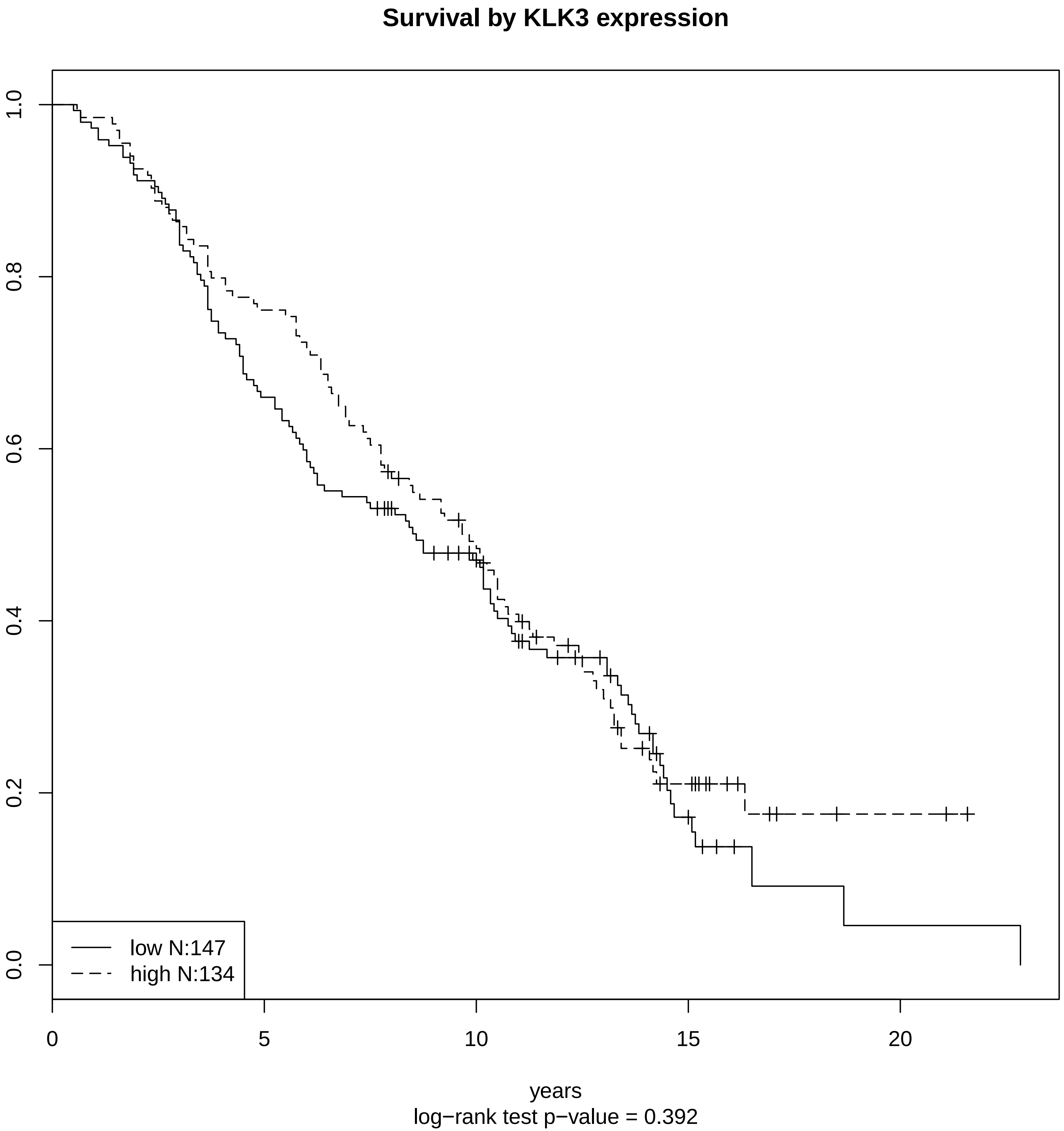
Survival by KLK3 expression
1.0
0.8
0.6
0.4
0.2
0.0
0
5
10
15
20
years
log−rank test p−value = 0.392
low N:147
high N:134

Survival by KLK4 expression

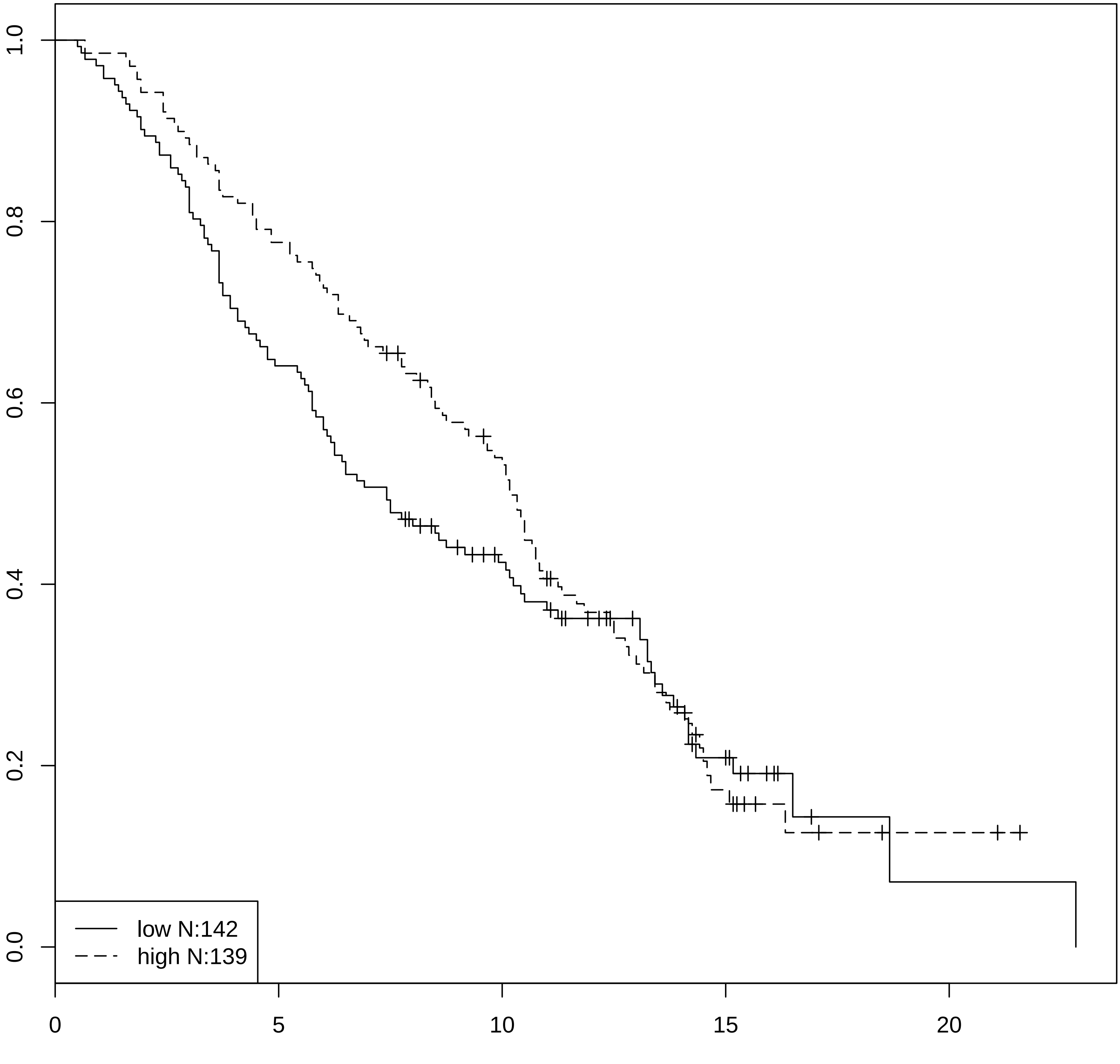

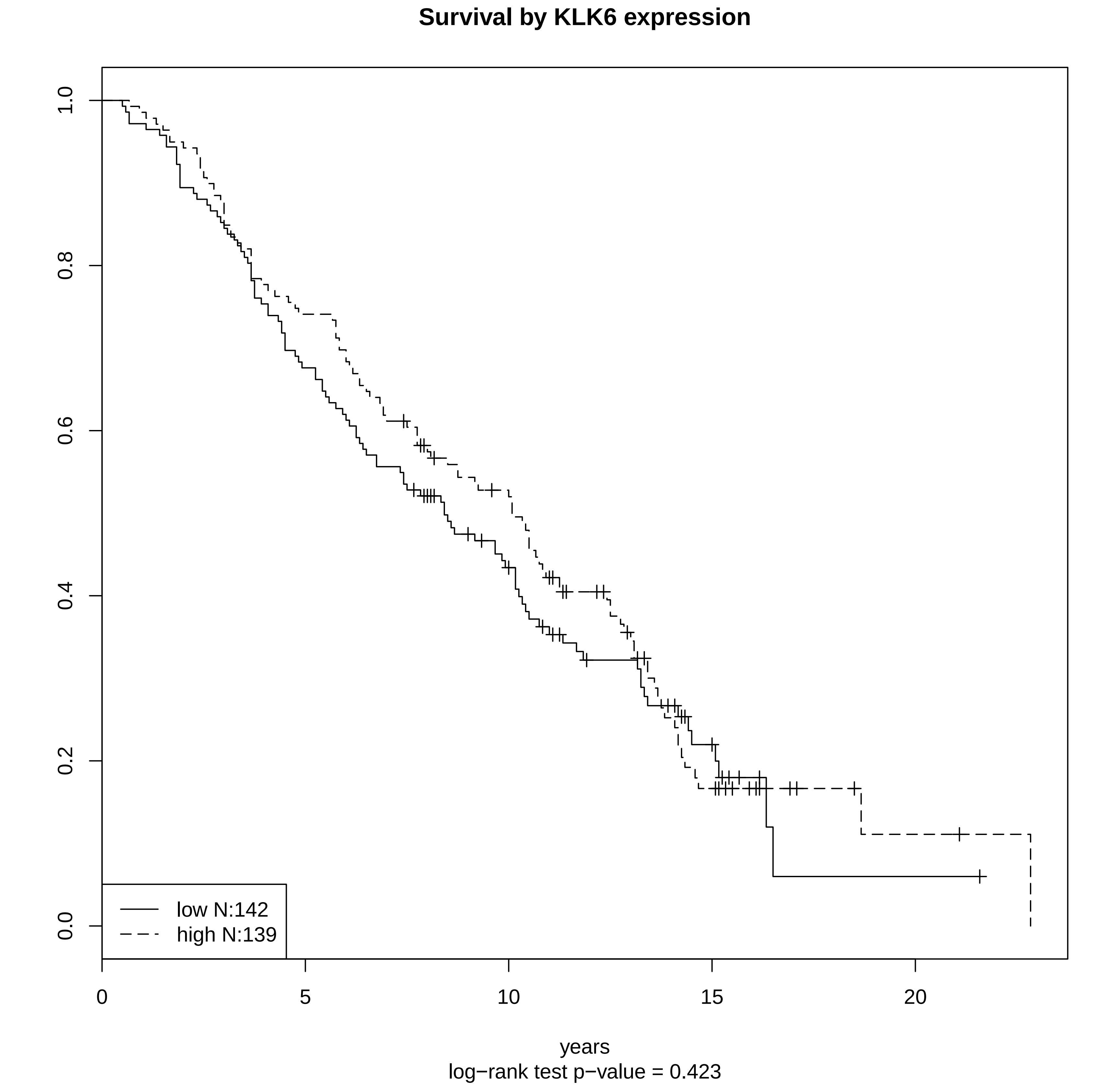
Survival by KLK6 expression
1.0
0.8
0.6
0.4
0.2
0.0
0
5
10
15
20
low N:142
high N:139
years
log−rank test p−value = 0.423

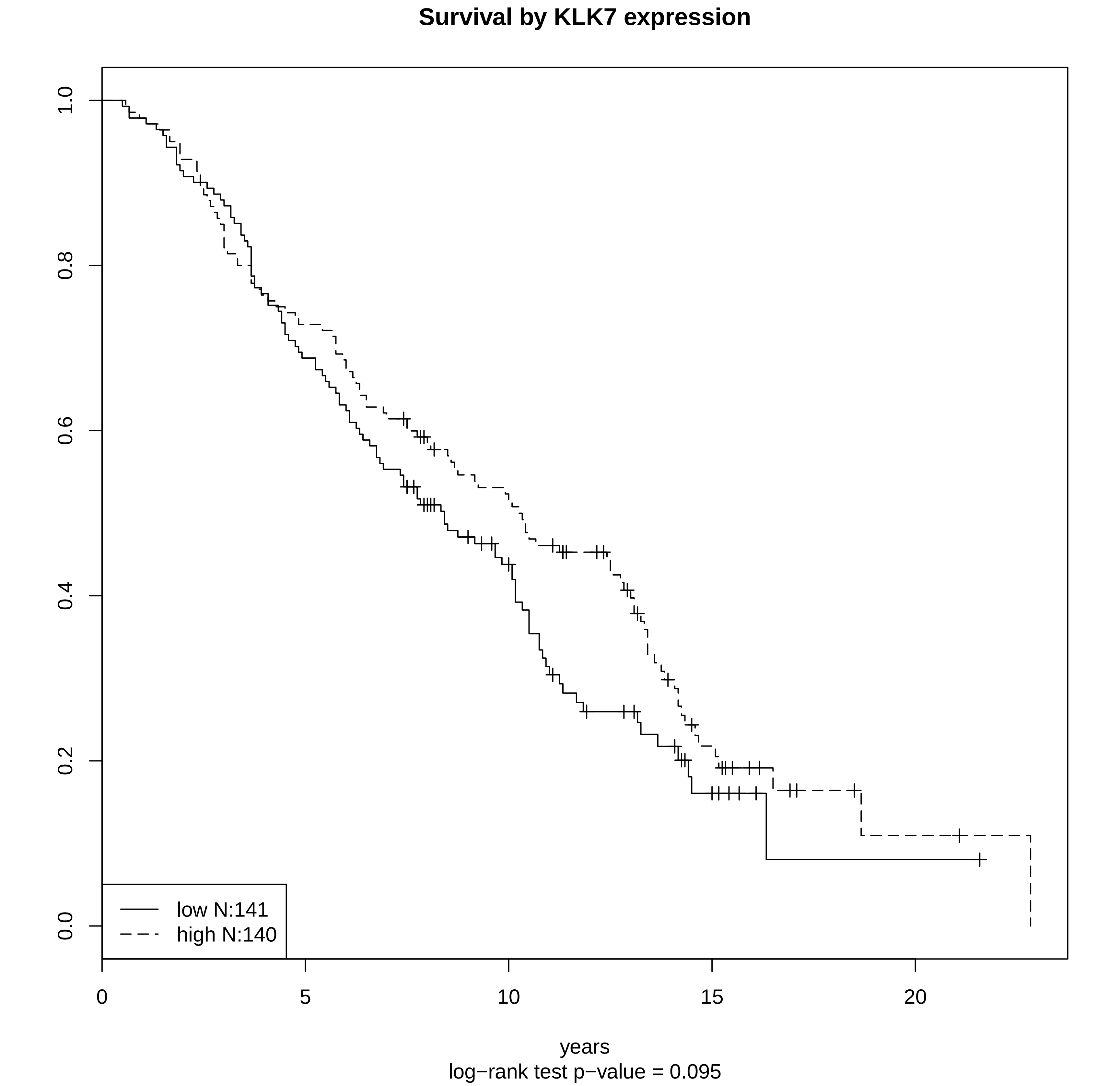

Survival by KLK7 expression
1.0
0.8
0.6
0.4
0.2
0.0
0
5
10
15
20
low N:141
high N:140
years
log−rank test p−value = 0.095

Survival by KLK8 expression

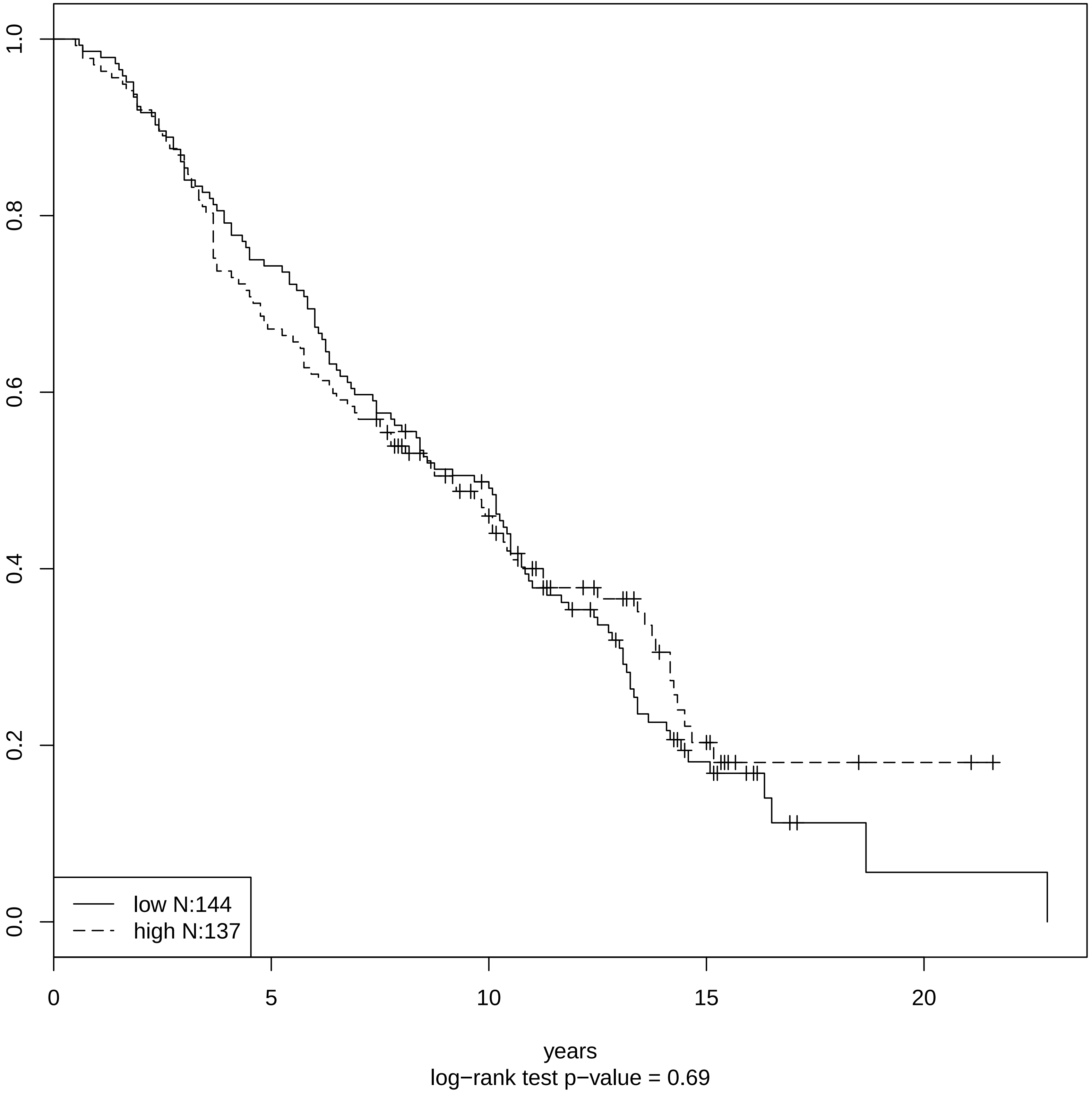

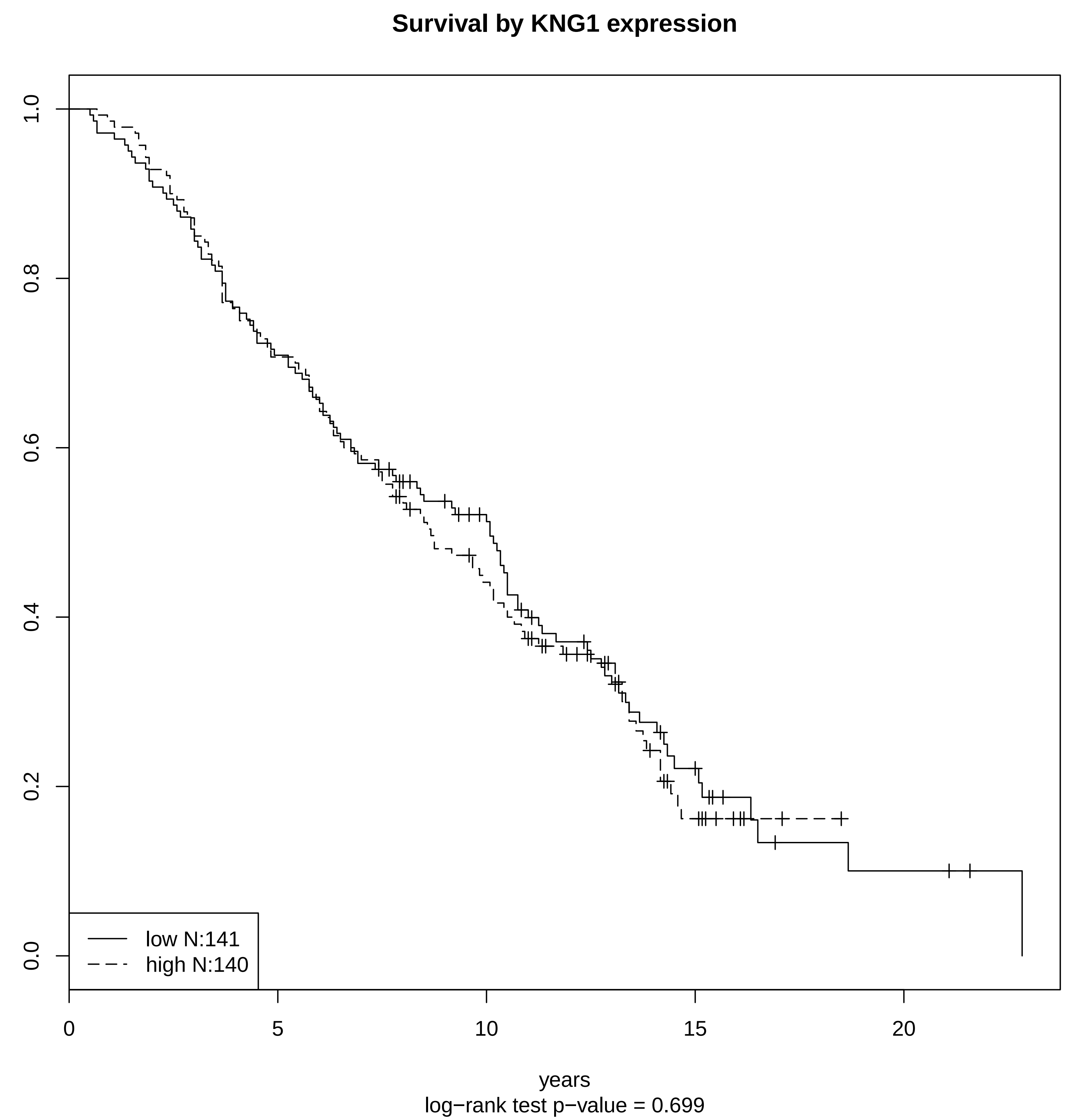
Survival by KNG1 expression
1.0
0.8
0.6
0.4
0.2
0.0
0
5
10
15
20
low N:141
high N:140
years
log−rank test p−value = 0.699

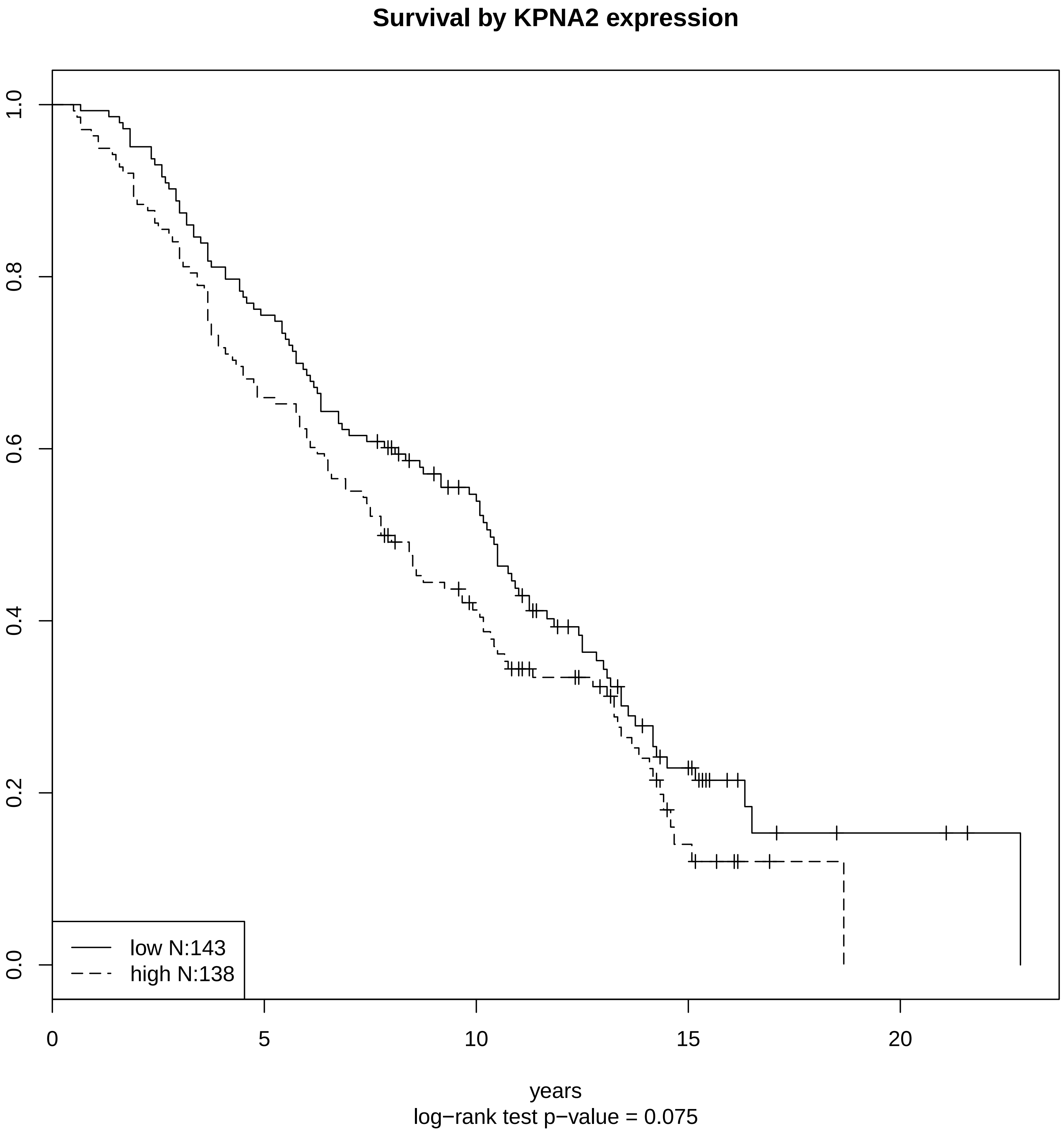
Survival by KPNA2 expression
1.0
0.8
0.6
0.4
0.2
0.0
low N:143
high N:138
0
5
10
15
20
years
log−rank test p−value = 0.075

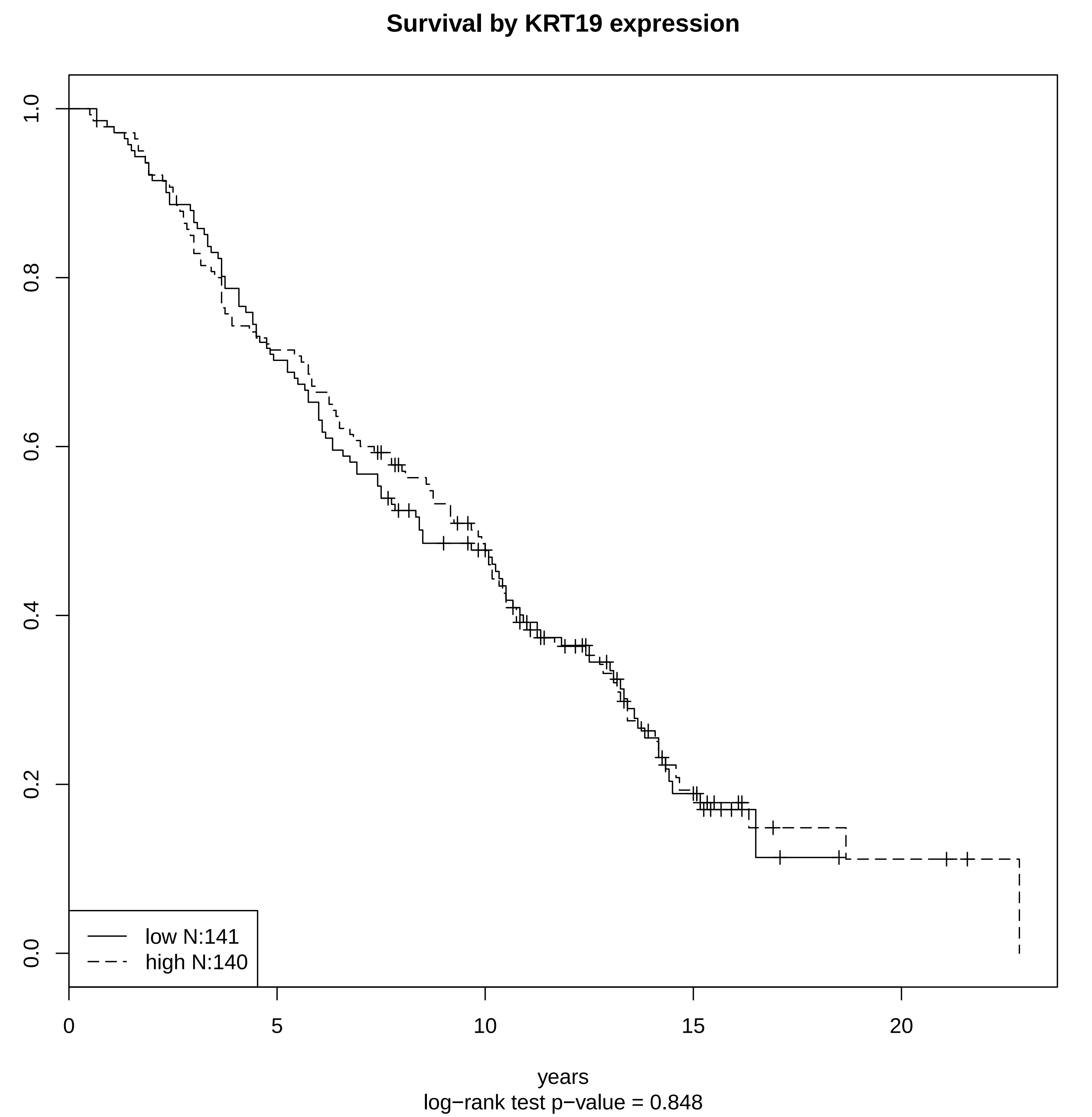
Survival by KRT19 expression
1.0
0.8
0.6
0.4
0.2
0.0
0
5
10
15
20
low N:141
high N:140
years
log−rank test p−value = 0.848

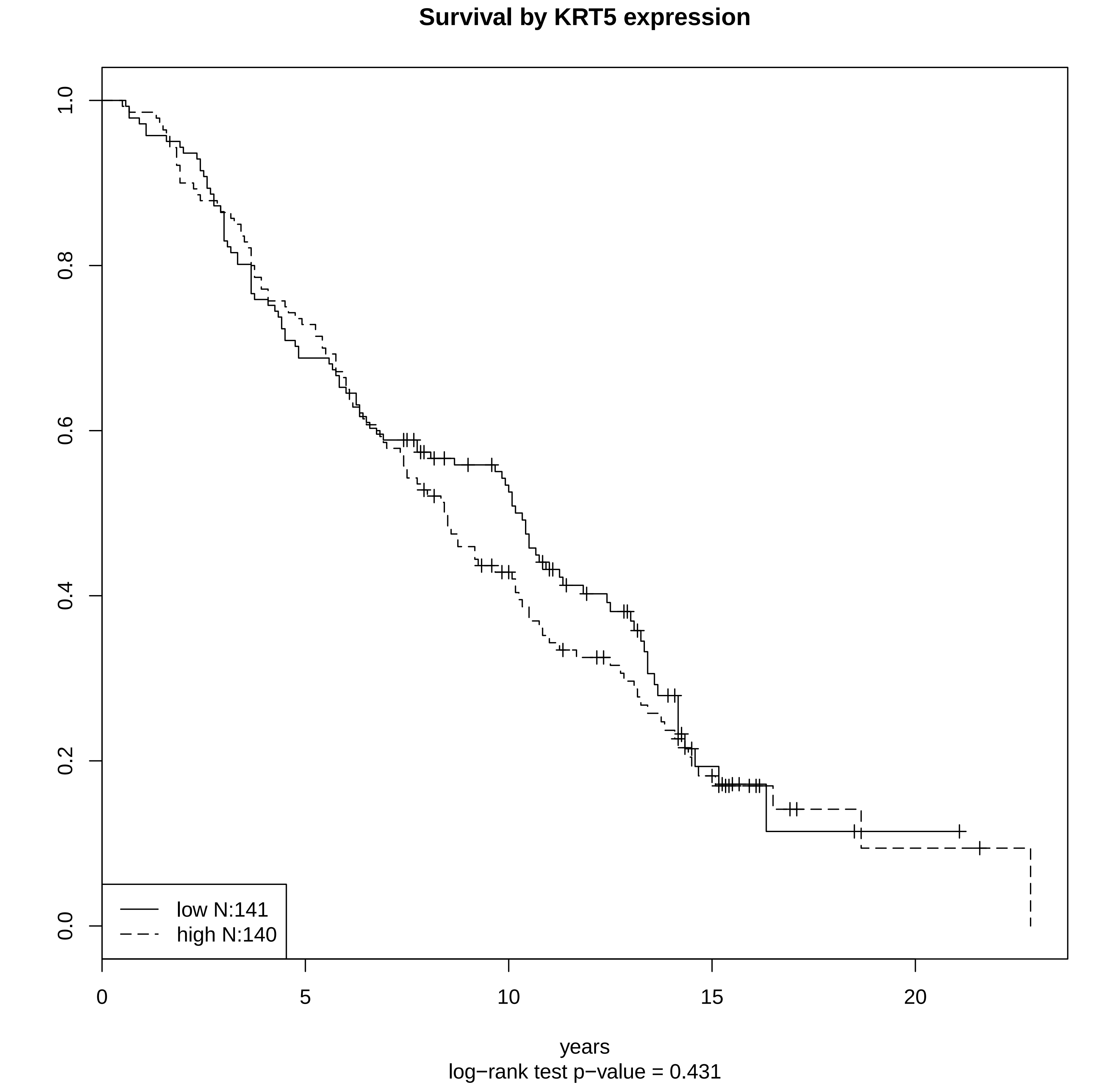

Survival by KRT5 expression
1.0
0.8
0.6
0.4
0.2
0.0
0
5
10
15
20
low N:141
high N:140
years
log−rank test p−value = 0.431

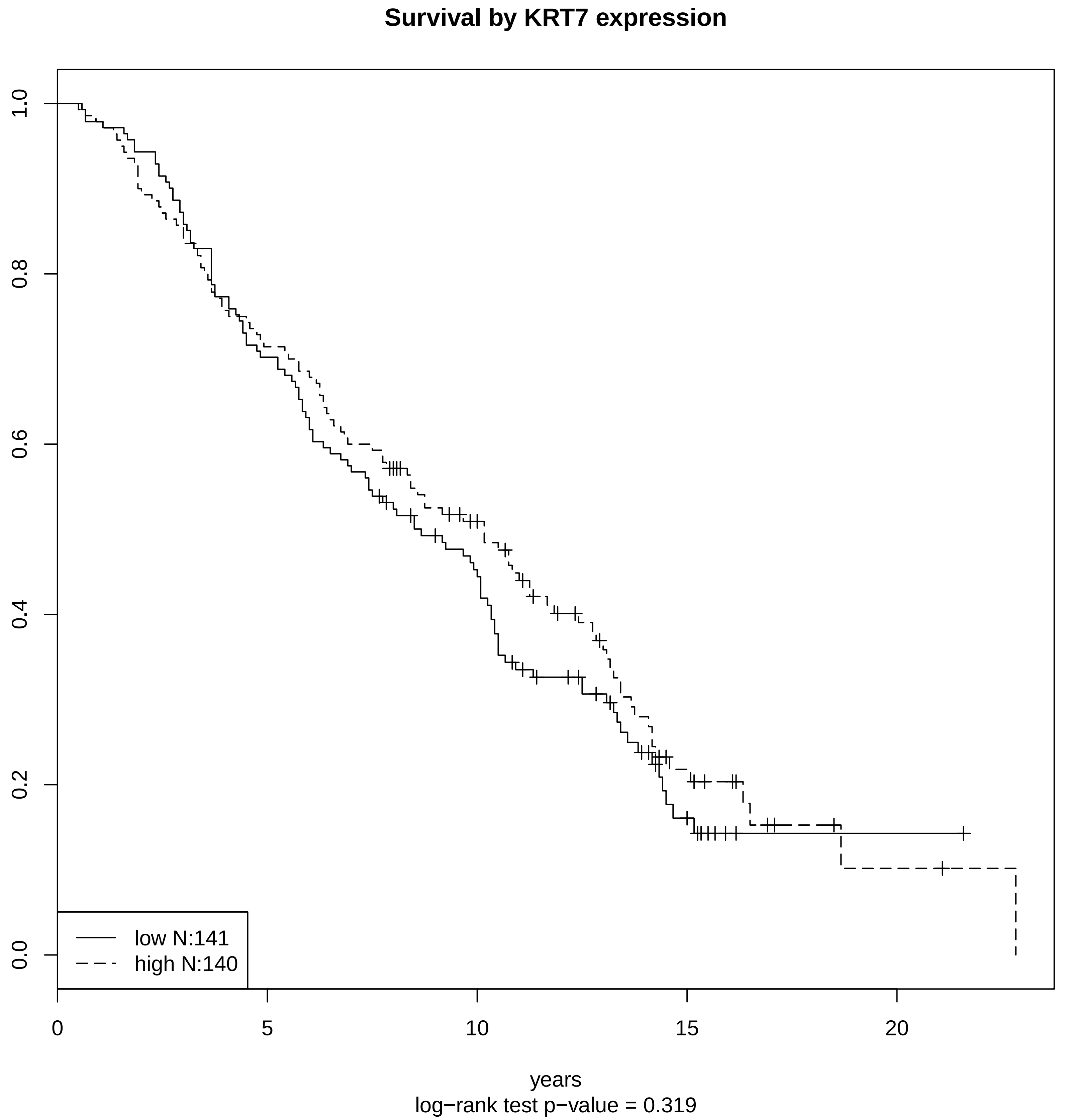
Survival by KRT7 expression
1.0
0.8
0.6
0.4
0.2
0.0
low N:141
high N:140
0
5
10
15
20
years
log−rank test p−value = 0.319

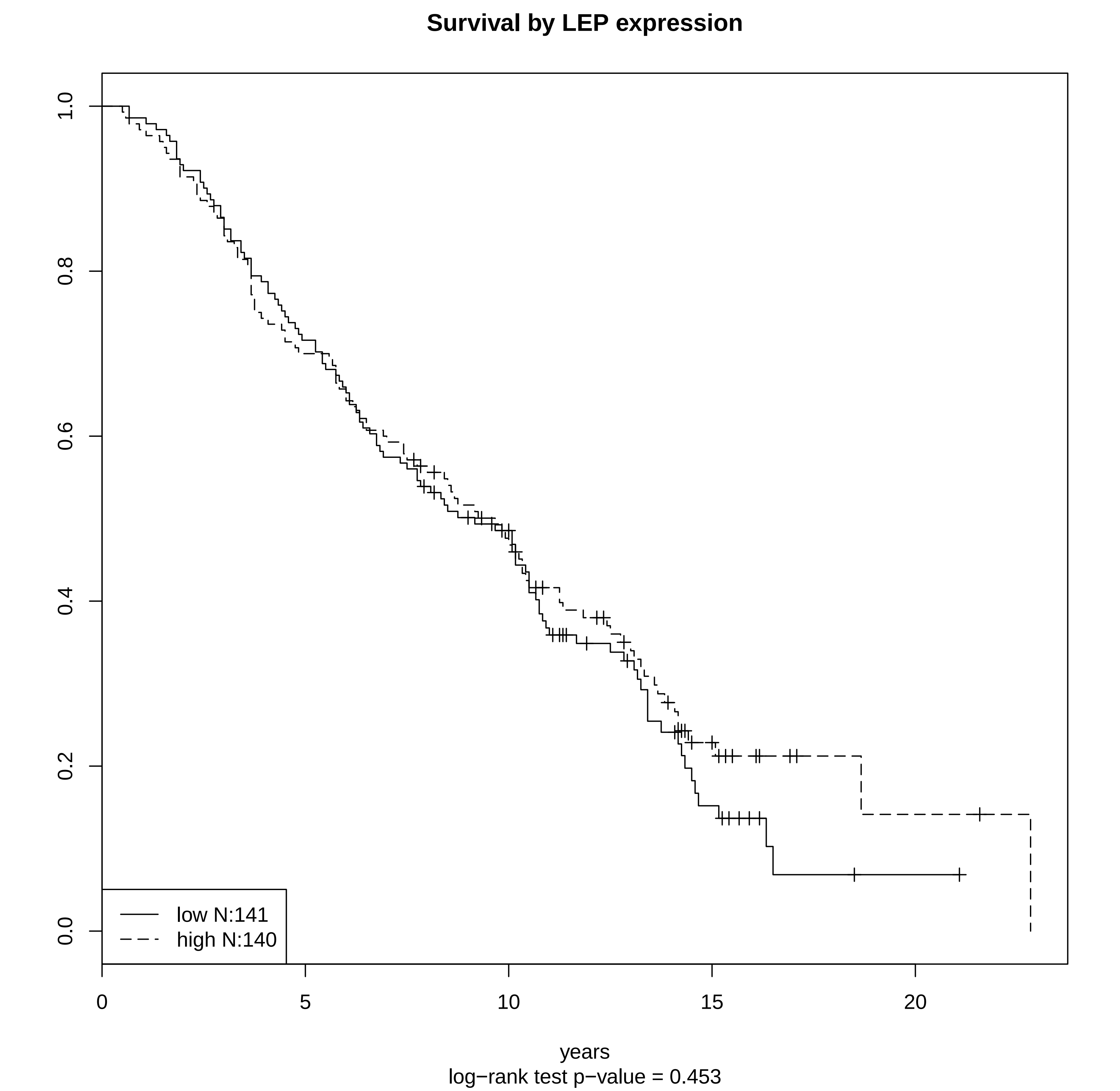
Survival by LEP expression
1.0
0.8
0.6
0.4
0.2
0.0
0
5
10
15
20
low N:141
high N:140
years
log−rank test p−value = 0.453

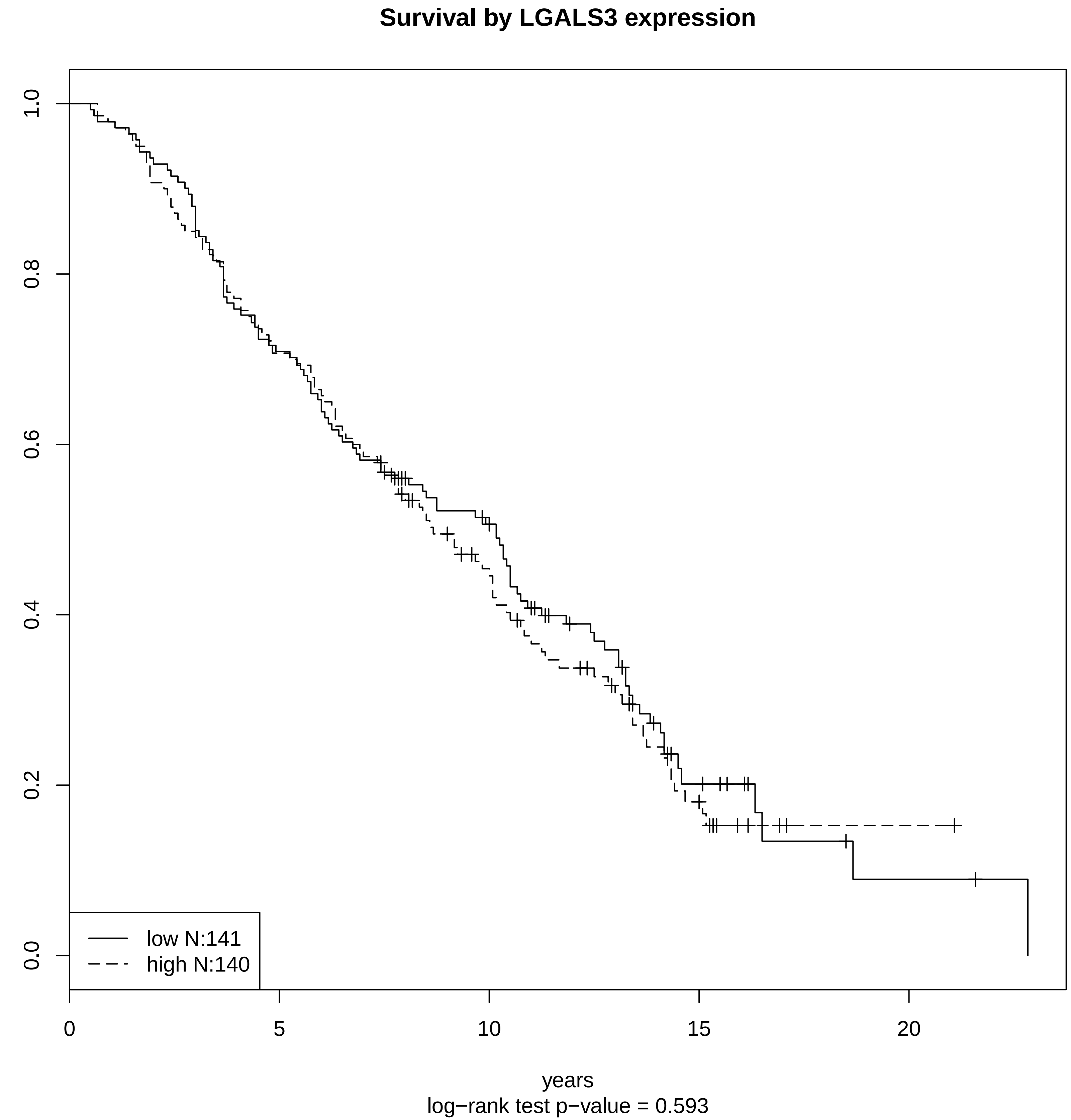

Survival by LGALS3 expression
1.0
0.8
0.6
0.4
0.2
0.0
0
5
10
15
20
low N:141
high N:140
years
log−rank test p−value = 0.593

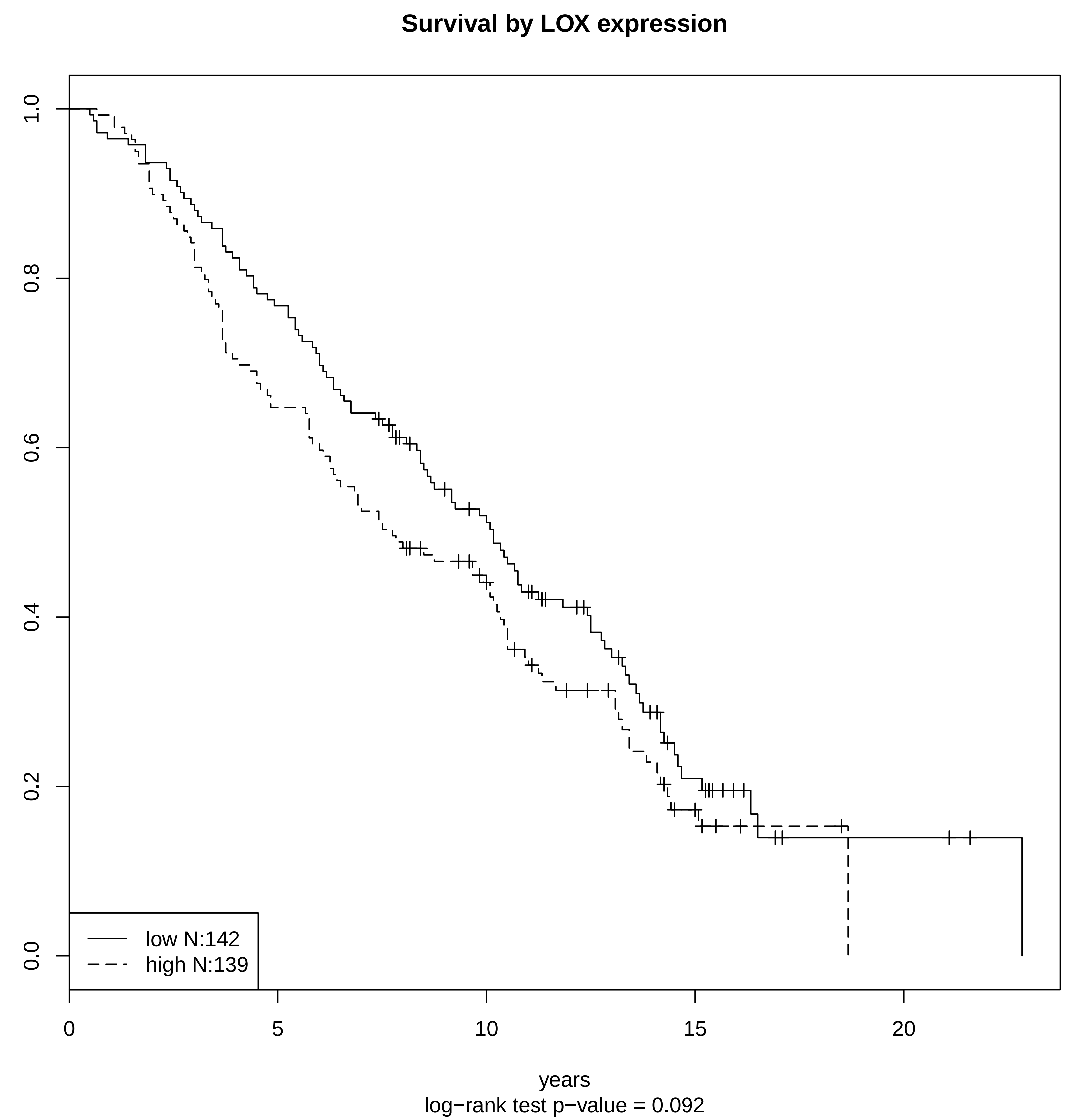
Survival by LOX expression
1.0
0.8
0.6
0.4
0.2
0.0
0
5
10
15
20
low N:142
high N:139
years
log−rank test p−value = 0.092

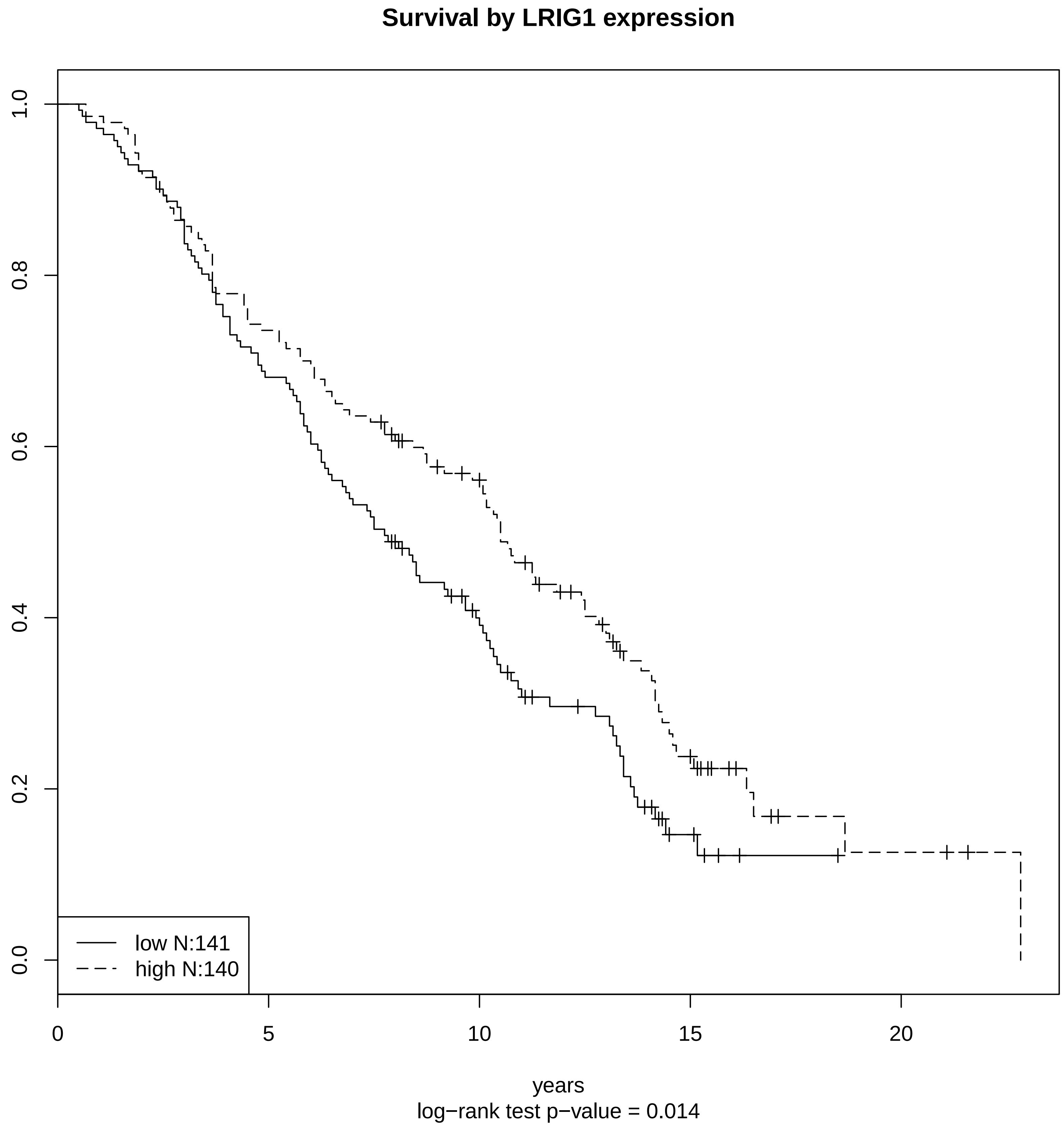
Survival by LRIG1 expression
1.0
0.8
0.6
0.4
0.2
0.0
0
5
10
15
20
years
low N:141
high N:140
log−rank test p−value = 0.014

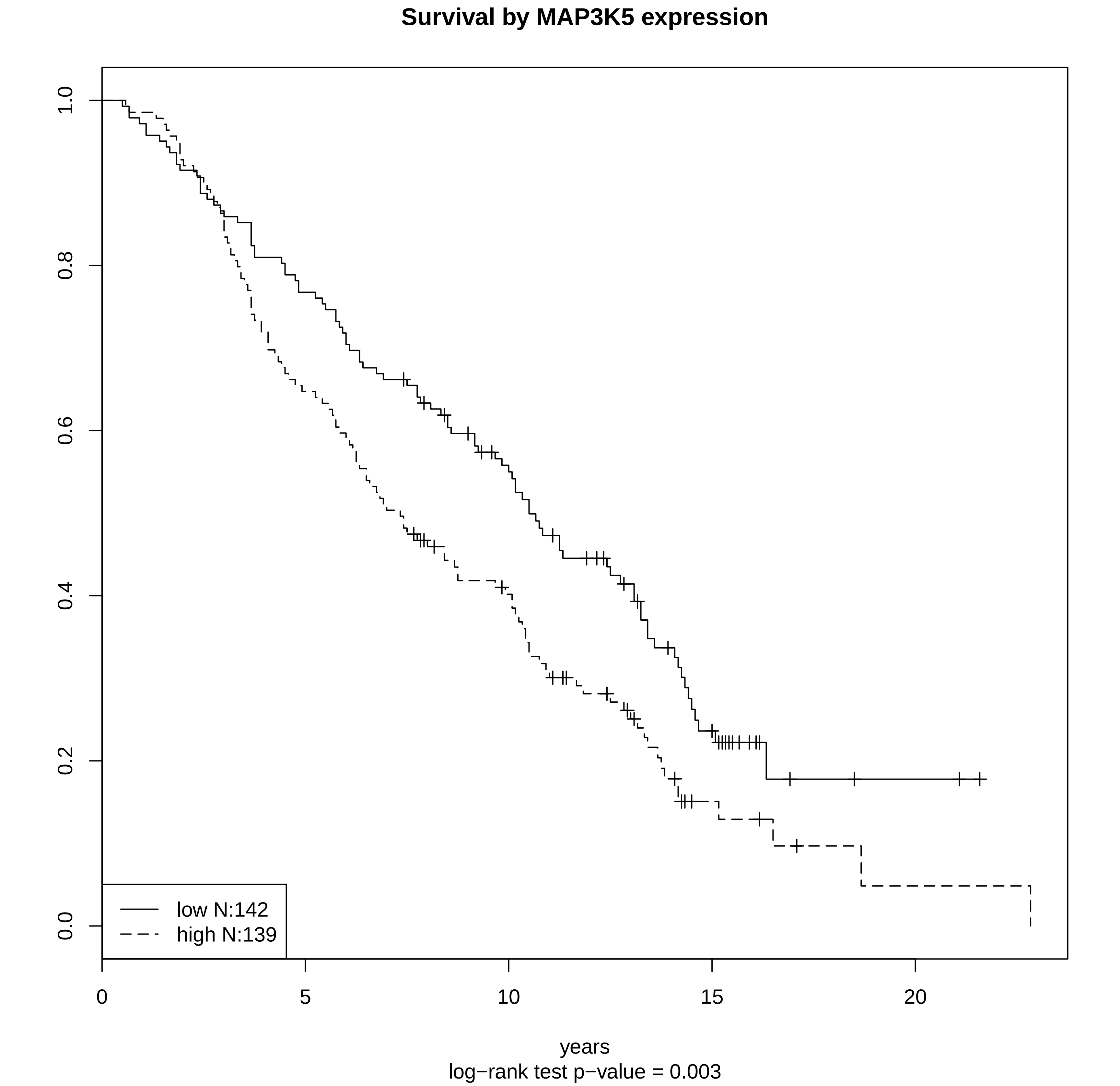
Survival by MAP3K5 expression
1.0
0.8
0.6
0.4
0.2
0.0
0
5
10
15
20
low N:142
high N:139
years
log−rank test p−value = 0.003

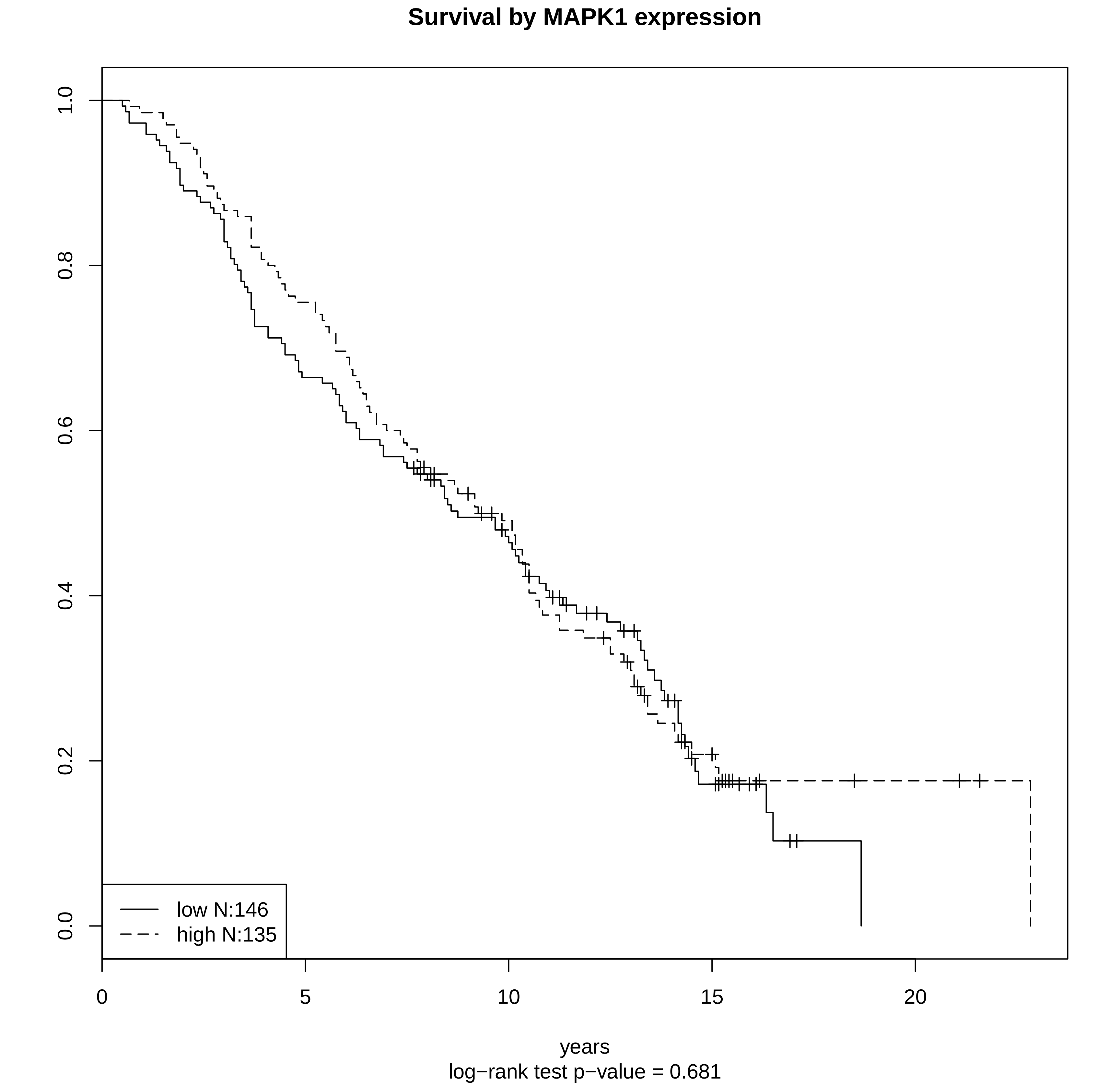
Survival by MAPK1 expression
1.0
0.8
0.6
0.4
0.2
0.0
0
5
10
15
20
low N:146
high N:135
years
log−rank test p−value = 0.681

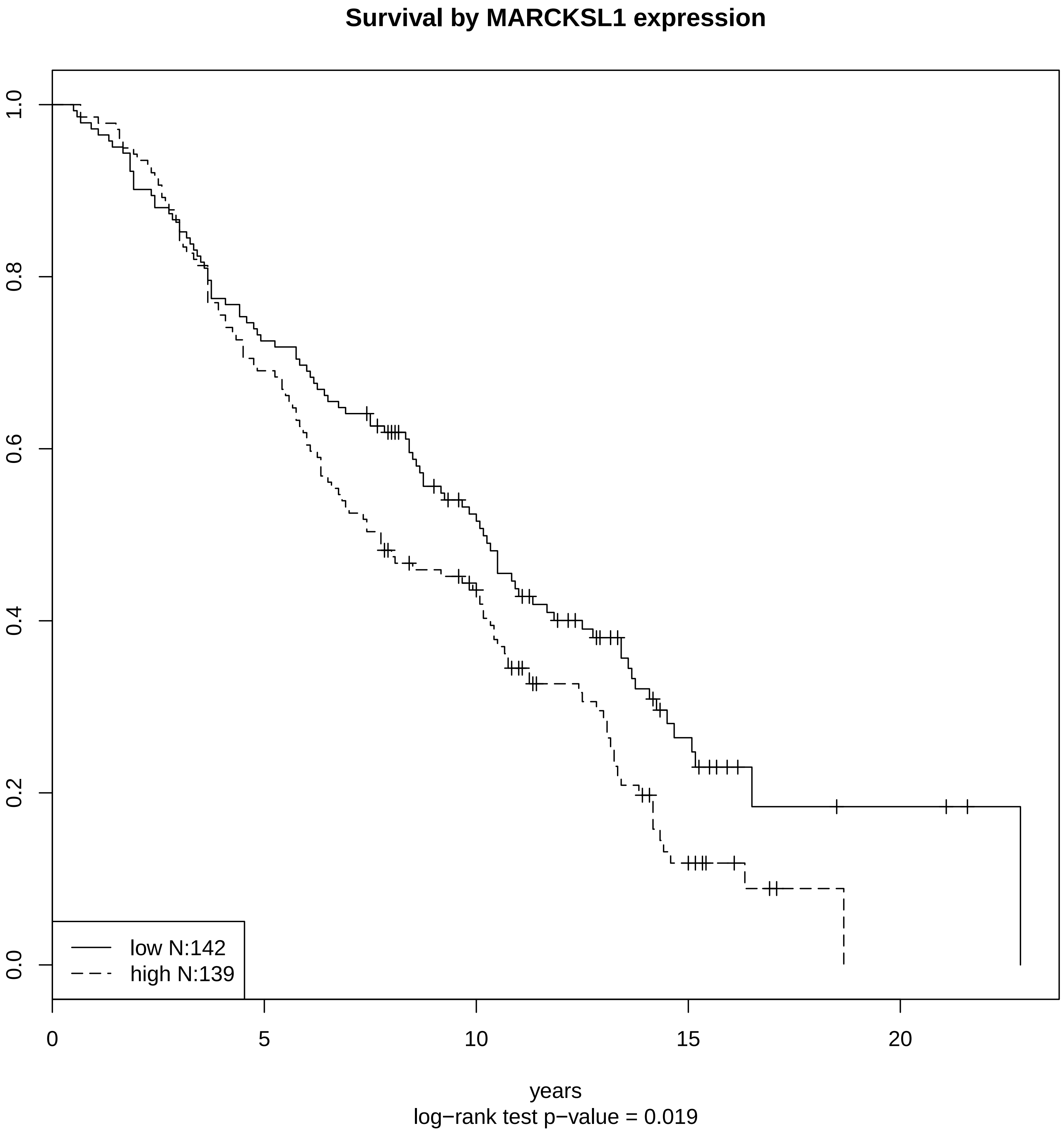

Survival by MARCKSL1 expression
1.0
0.8
0.6
0.4
0.2
0.0
0
5
10
15
20
years
log−rank test p−value = 0.019
low N:142
high N:139

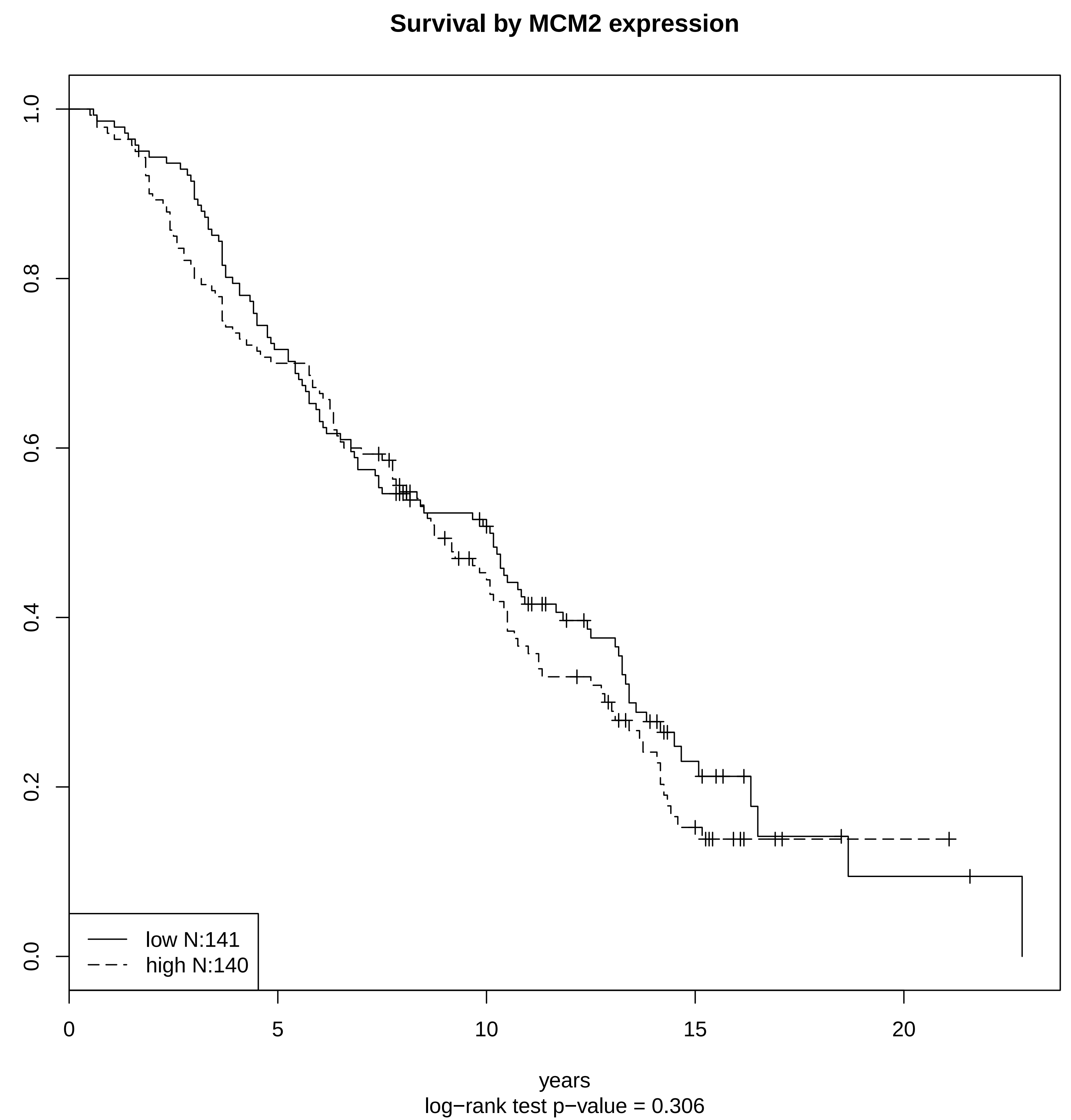
Survival by MCM2 expression
1.0
0.8
0.6
0.4
0.2
0.0
0
5
10
15
20
low N:141
high N:140
years
log−rank test p−value = 0.306

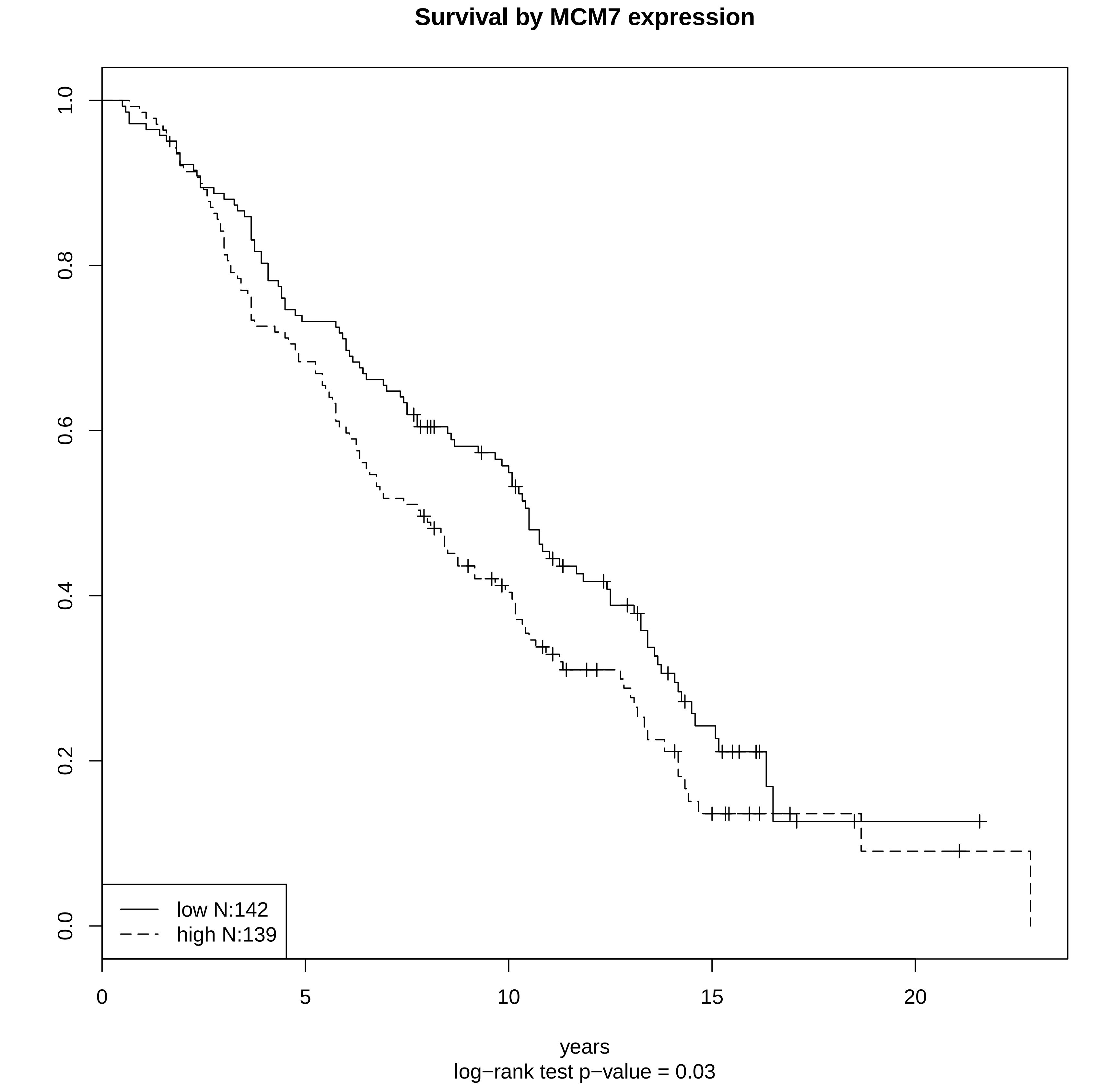

Survival by MCM7 expression
1.0
0.8
0.6
0.4
0.2
0.0
0
5
10
15
20
low N:142
high N:139
years
log−rank test p−value = 0.03

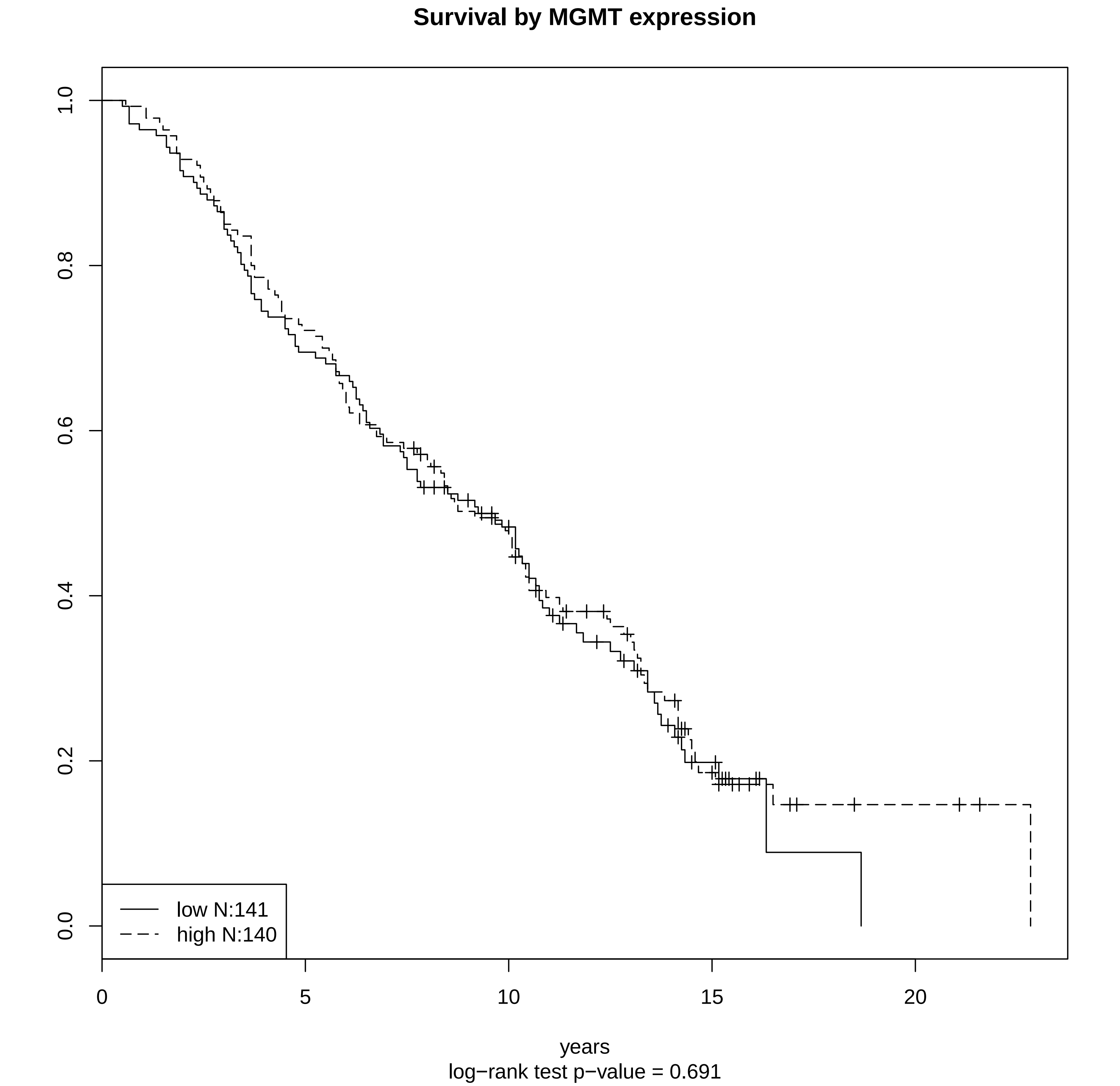
Survival by MGMT expression
1.0
0.8
0.6
0.4
0.2
0.0
0
5
10
15
20
low N:141
high N:140
years
log−rank test p−value = 0.691

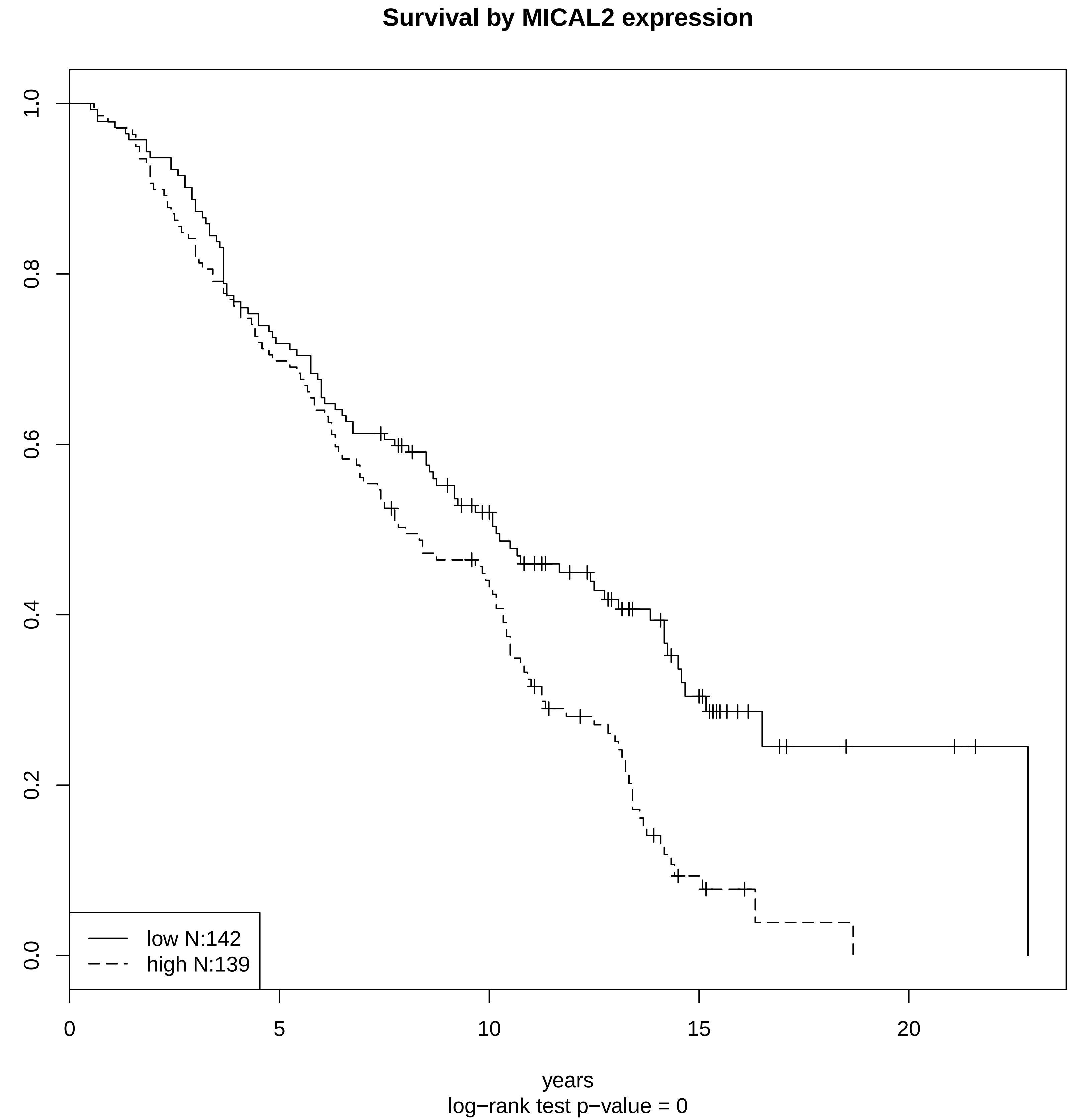
Survival by MICAL2 expression
1.0
0.8
0.6
0.4
0.2
0.0
0
5
10
15
20
low N:142
high N:139
years
log−rank test p−value = 0

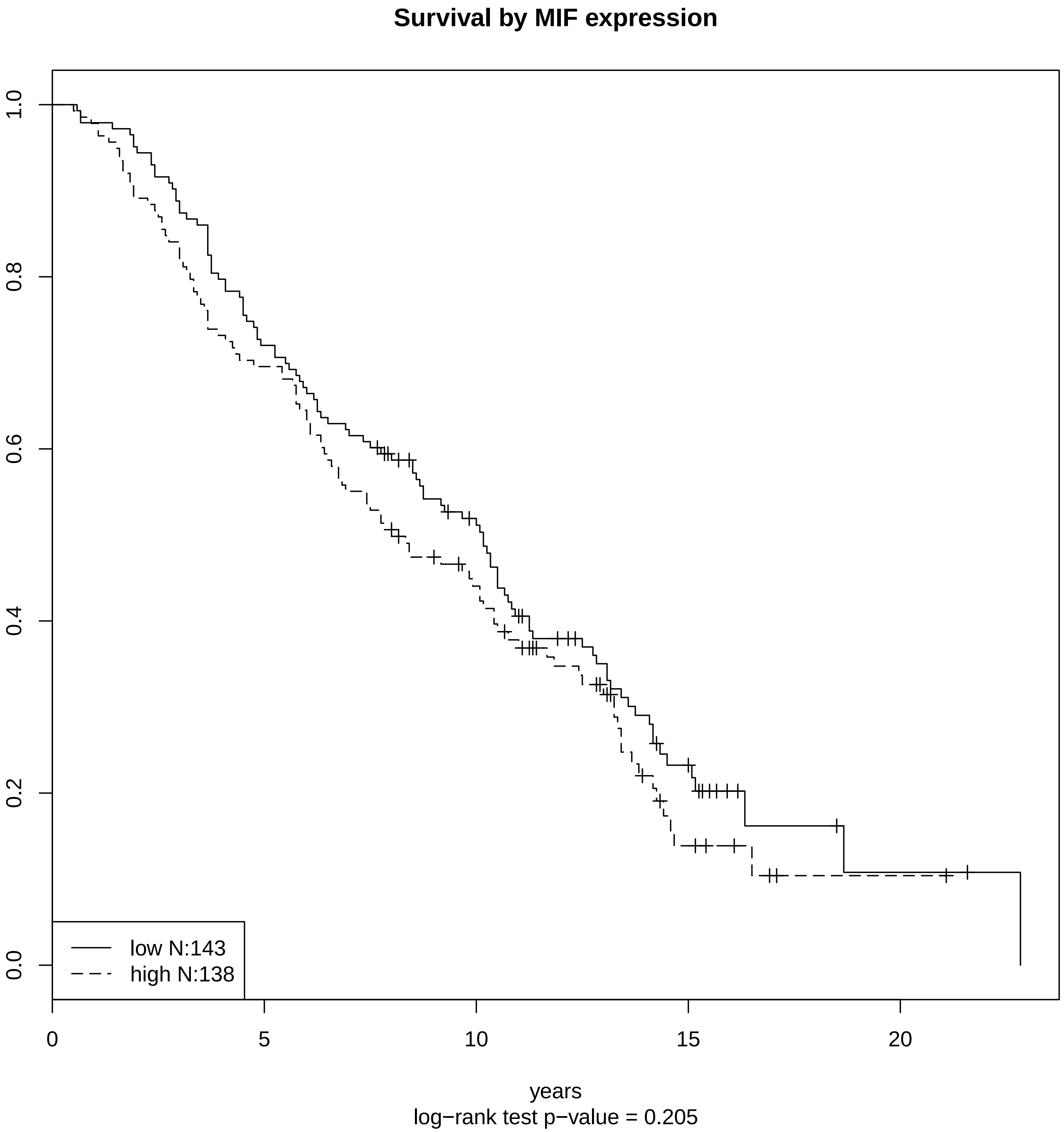
Survival by MIF expression
1.0
0.8
0.6
0.4
0.2
0.0
0
5
10
15
20
low N:143
high N:138
years
log−rank test p−value = 0.205

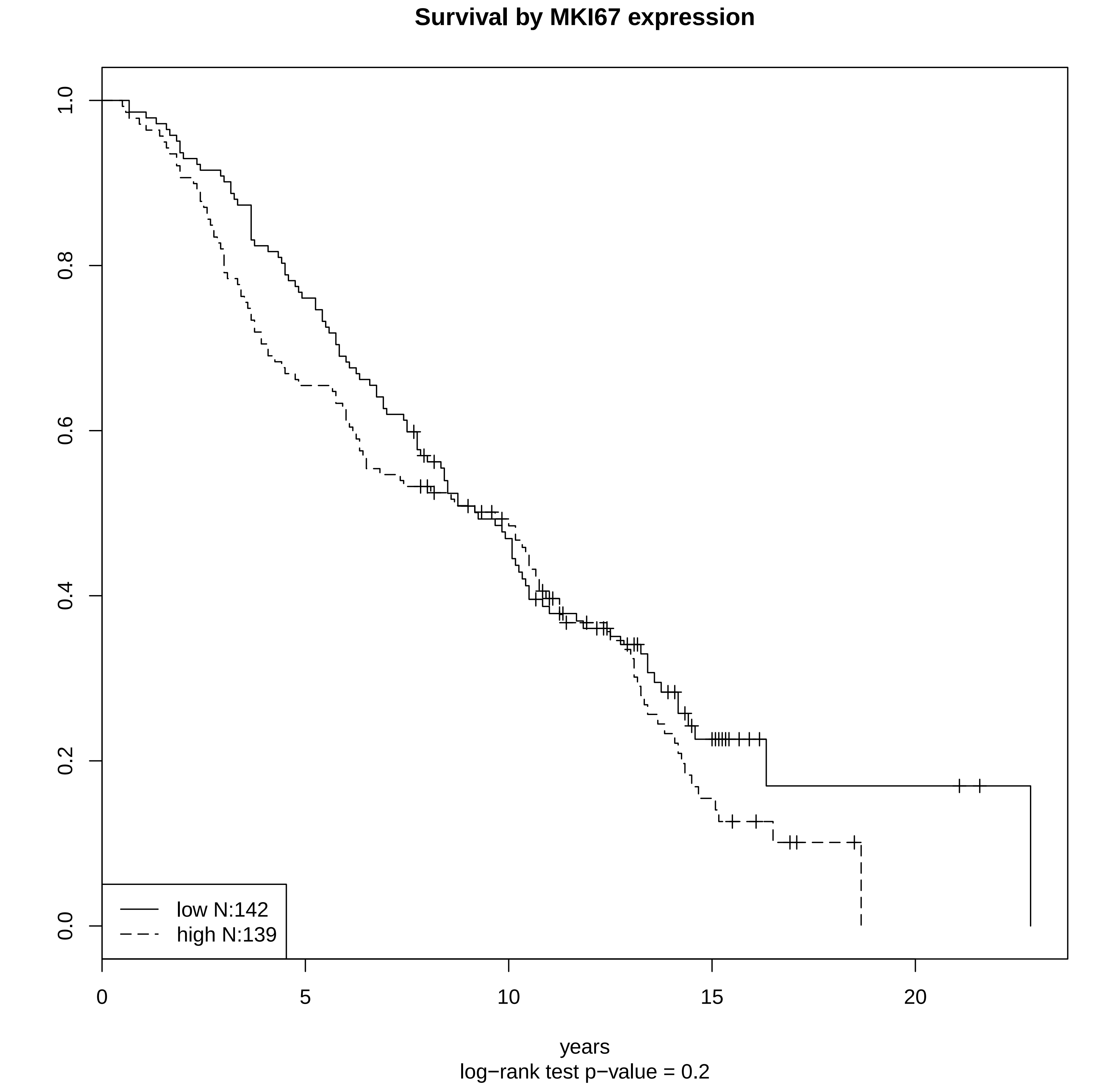
Survival by MKI67 expression
1.0
0.8
0.6
0.4
0.2
0.0
0
5
10
15
20
low N:142
high N:139
years
log−rank test p−value = 0.2

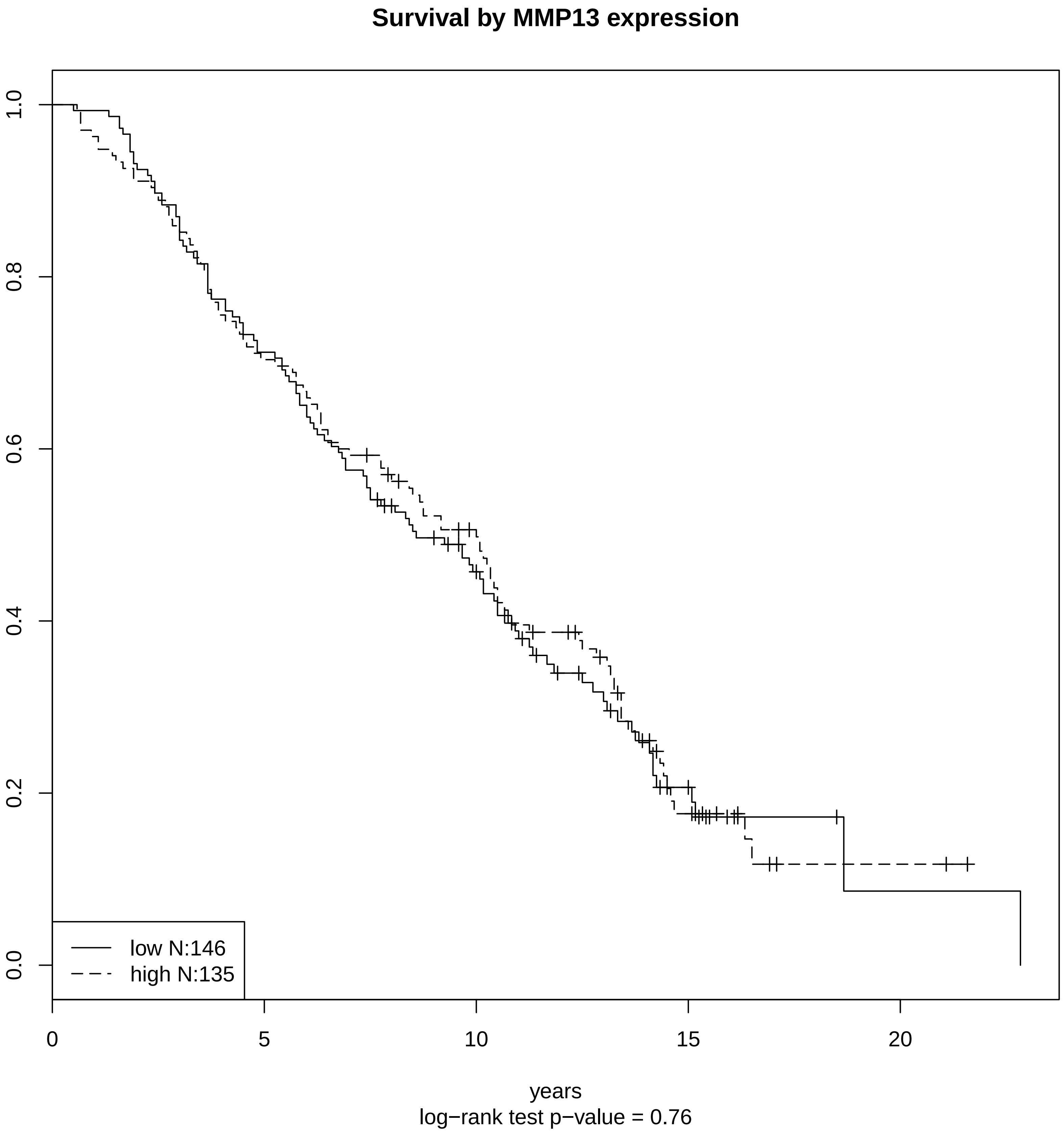
Survival by MMP13 expression
1.0
0.8
0.6
0.4
0.2
0.0
0
5
10
15
20
years
low N:146
high N:135
log−rank test p−value = 0.76

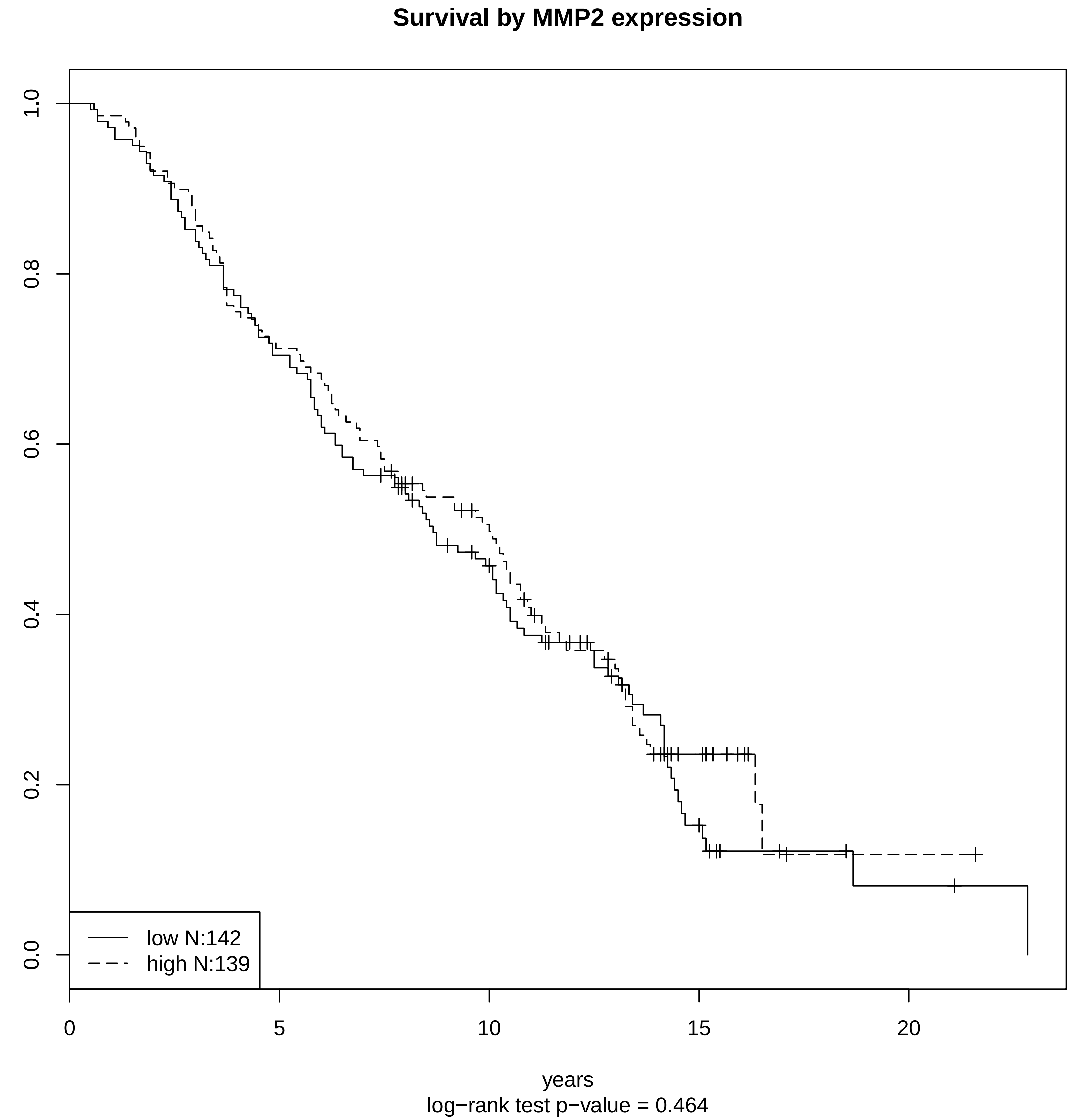
Survival by MMP2 expression
1.0
0.8
0.6
0.4
0.2
0.0
0
5
10
15
20
low N:142
high N:139
years
log−rank test p−value = 0.464

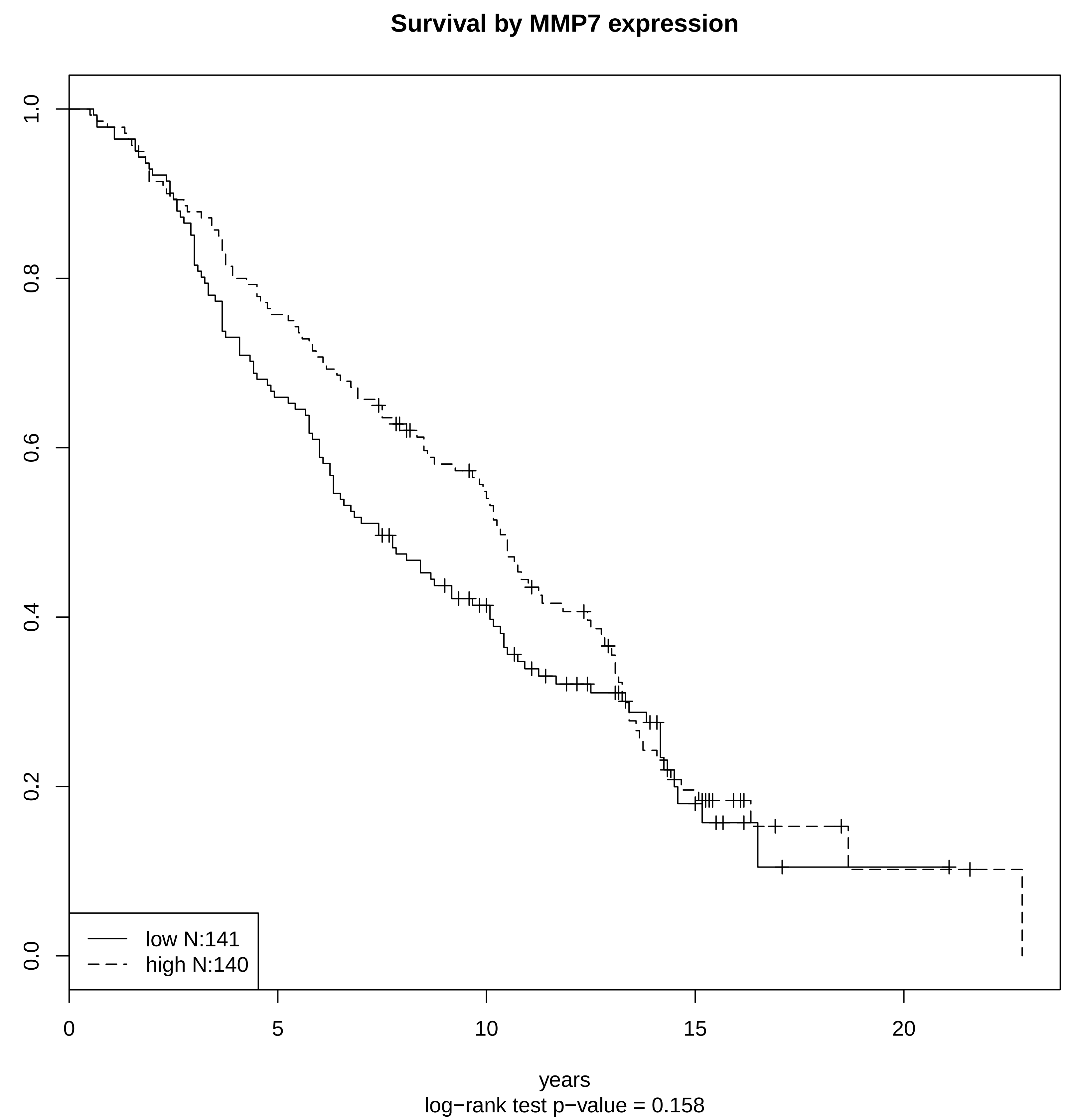
Survival by MMP7 expression
1.0
0.8
0.6
0.4
0.2
0.0
0
5
10
15
20
low N:141
high N:140
years
log−rank test p−value = 0.158

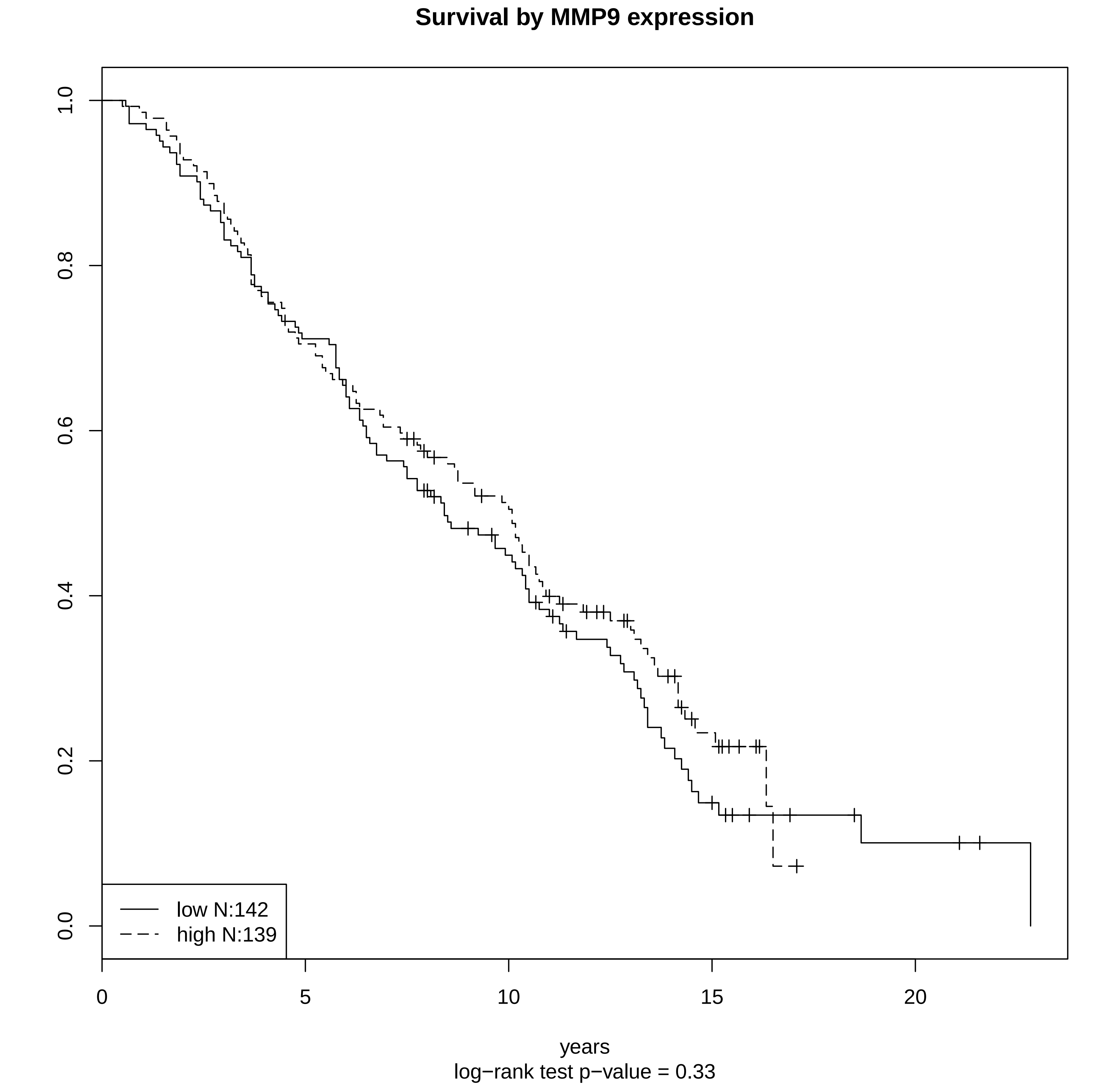
Survival by MMP9 expression
1.0
0.8
0.6
0.4
0.2
0.0
0
5
10
15
20
low N:142
high N:139
years
log−rank test p−value = 0.33

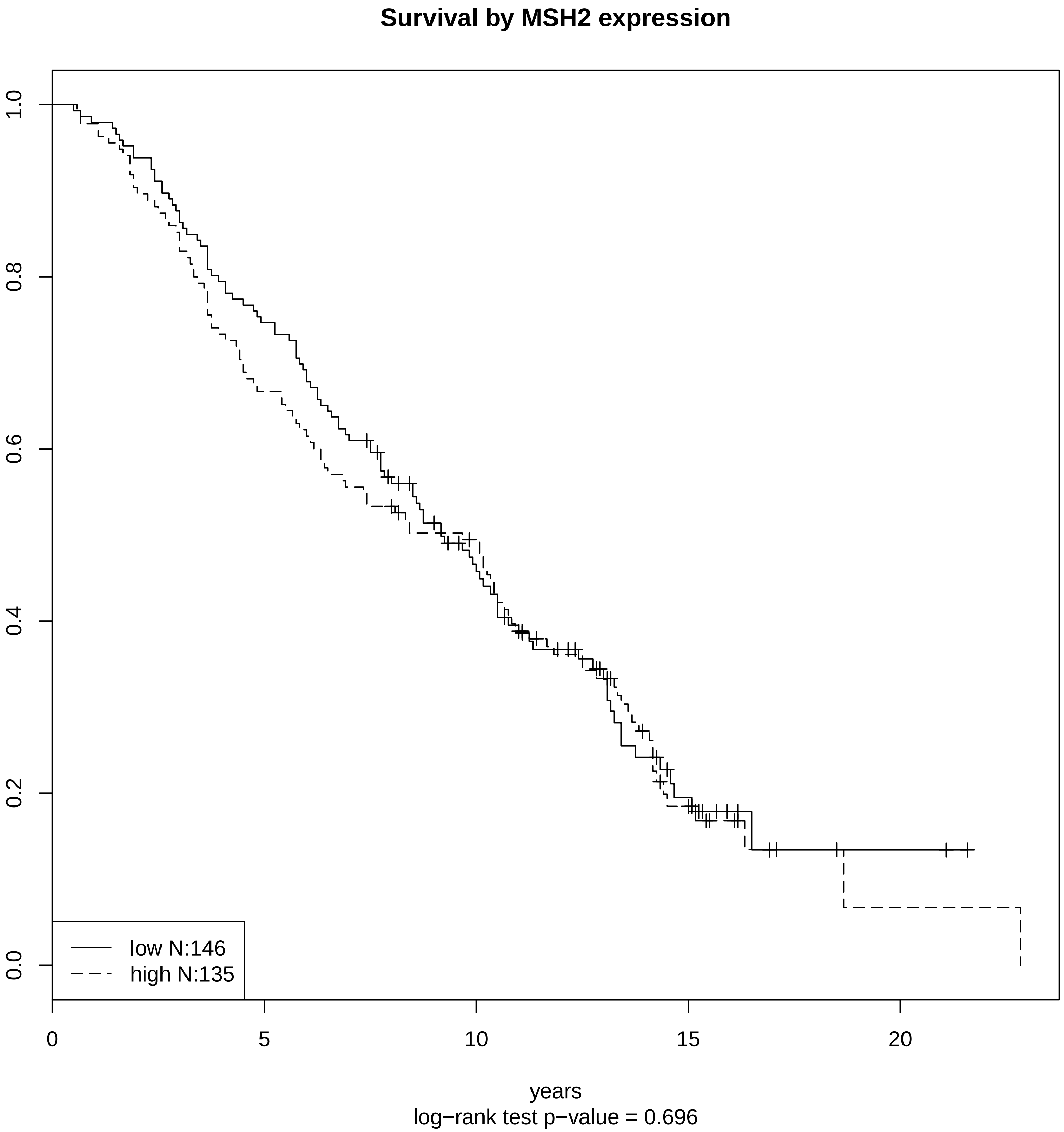
Survival by MSH2 expression
1.0
0.8
0.6
0.4
0.2
0.0
0
5
10
15
20
years
log−rank test p−value = 0.696
low N:146
high N:135

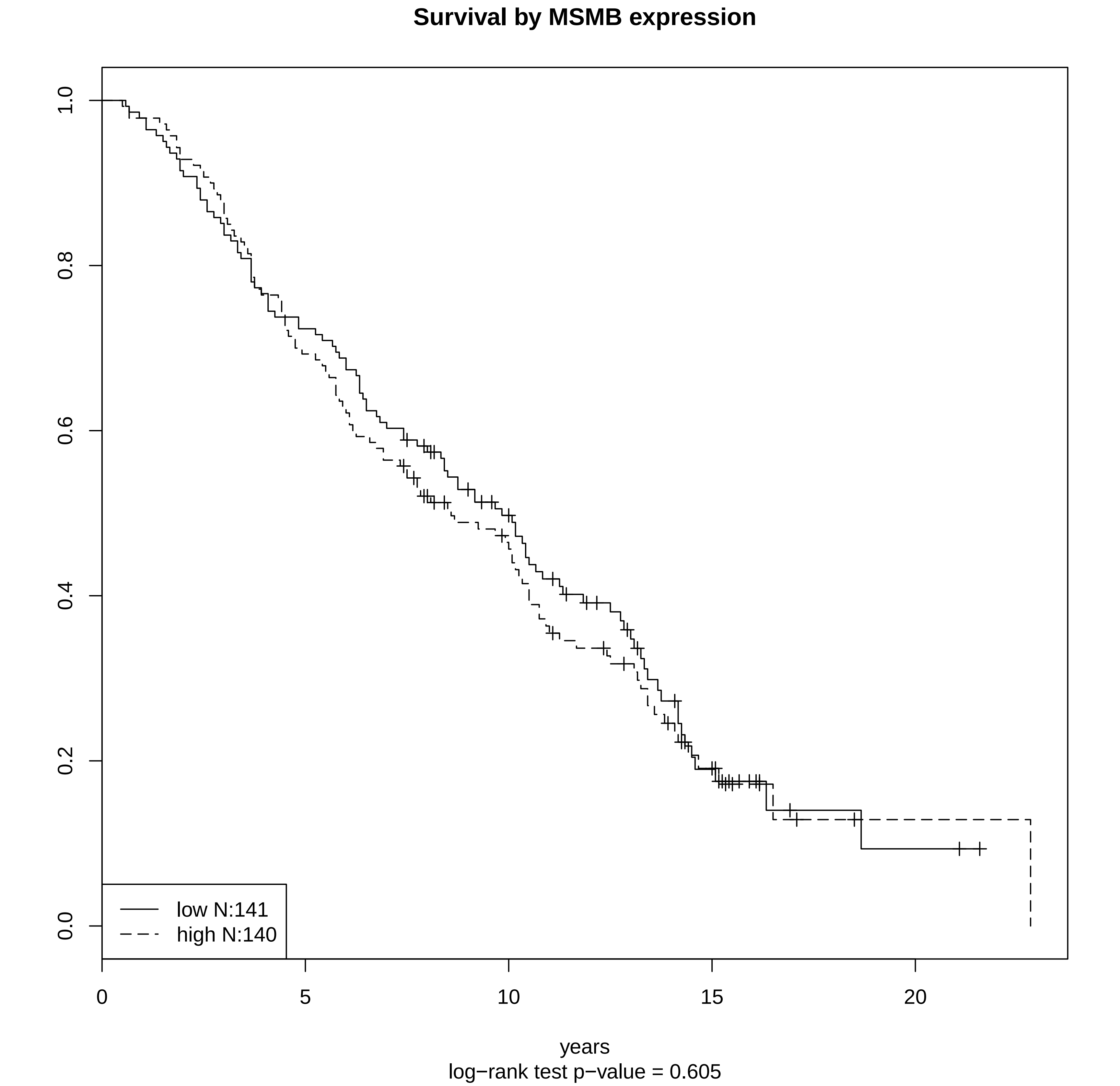
Survival by MSMB expression
1.0
0.8
0.6
0.4
0.2
0.0
0
5
10
15
20
years
log−rank test p−value = 0.605
low N:141
high N:140

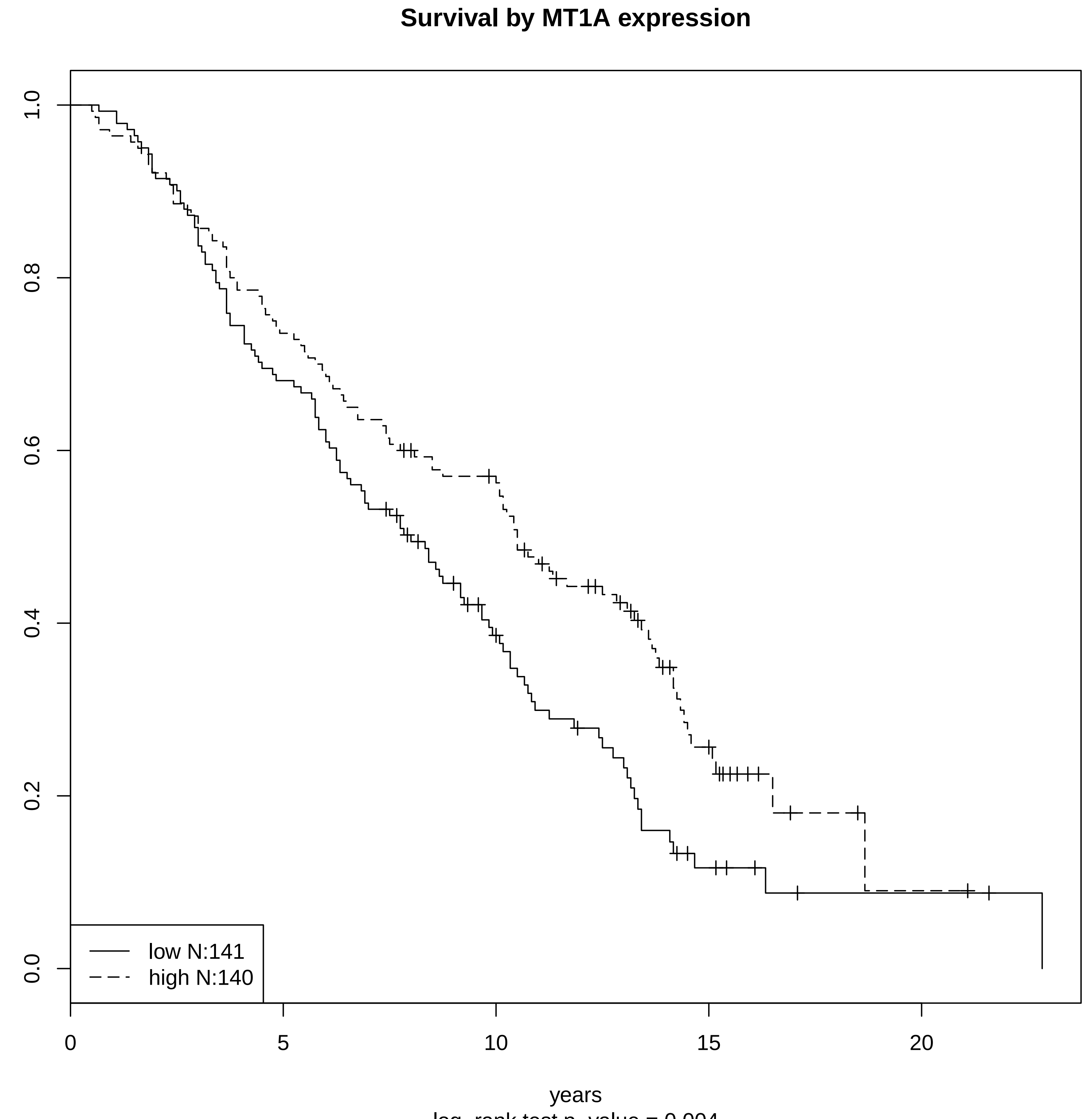

Survival by MT1A expression
1.0
0.8
0.6
0.4
0.2
0.0
0
5
10
15
20
years
log−rank test p−value = 0.004
low N:141
high N:140

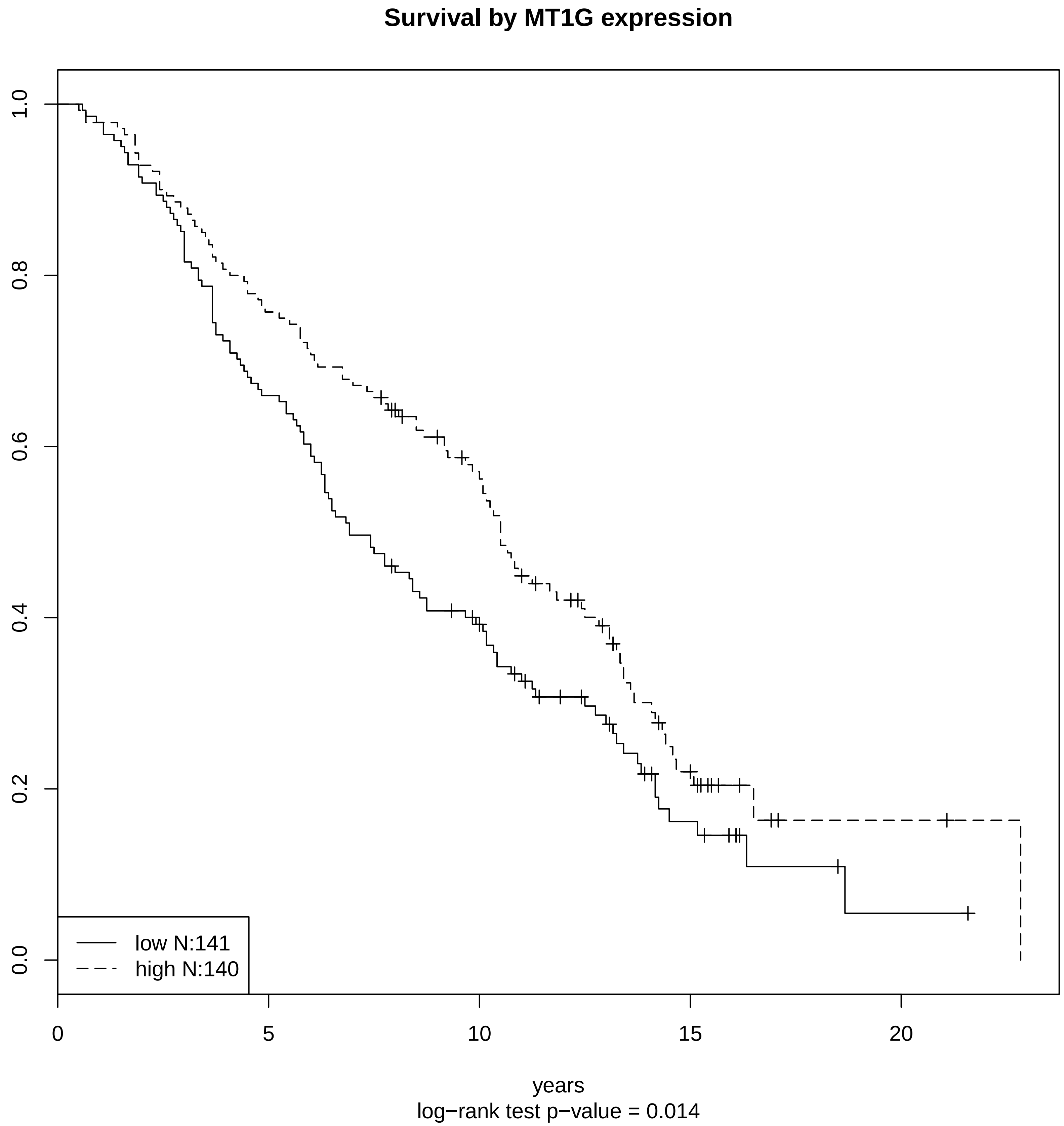
Survival by MT1G expression
1.0
0.8
0.6
0.4
0.2
0.0
0
5
10
15
20
low N:141
high N:140
years
log−rank test p−value = 0.014

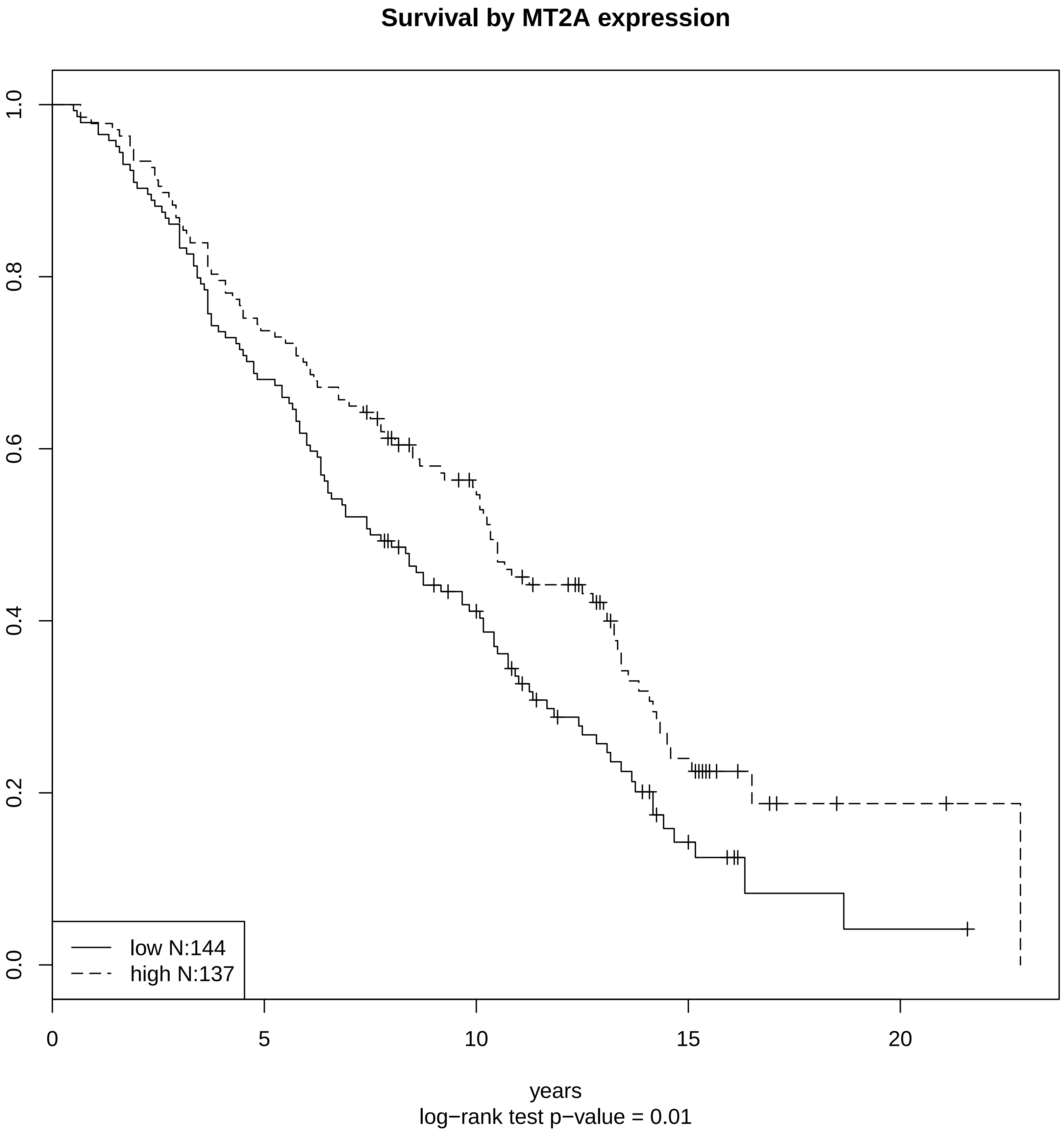
Survival by MT2A expression
1.0
0.8
0.6
0.4
0.2
0.0
0
5
10
15
20
years
log−rank test p−value = 0.01
low N:144
high N:137

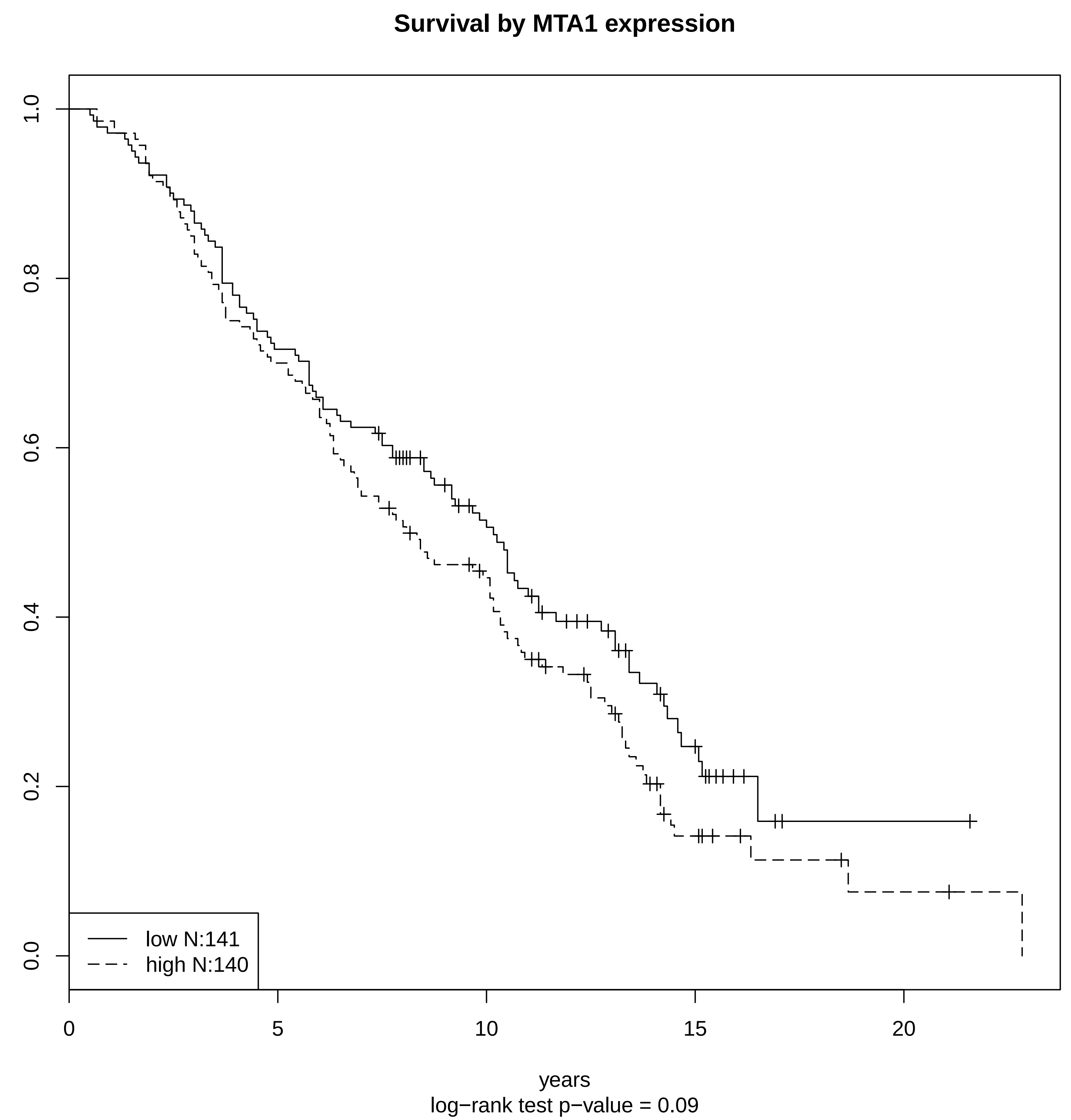
Survival by MTA1 expression
1.0
0.8
0.6
0.4
0.2
0.0
0
5
10
15
20
years
low N:141
high N:140
log−rank test p−value = 0.09

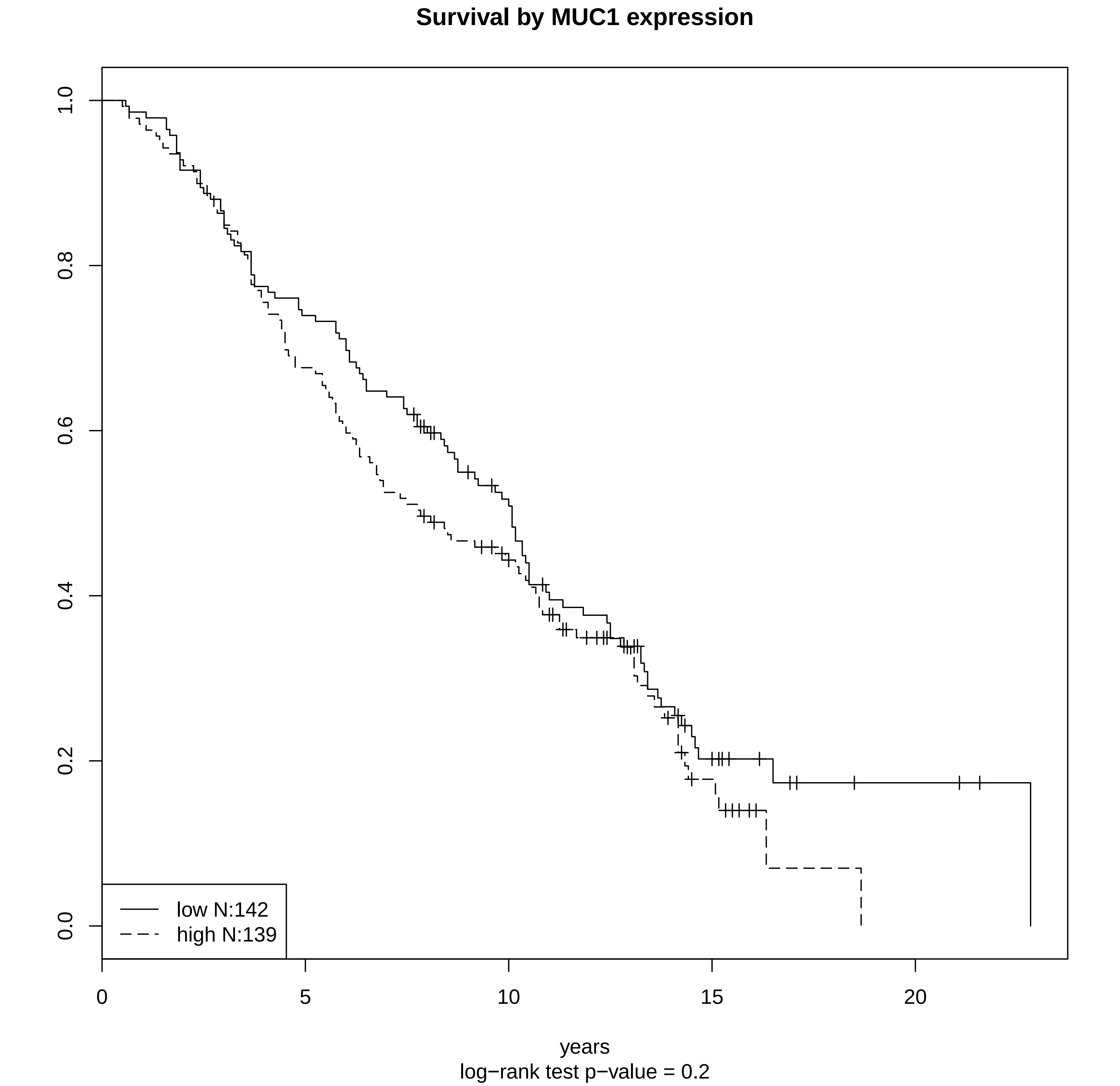

Survival by MUC1 expression
1.0
0.8
0.6
0.4
0.2
0.0
0
5
10
15
20
low N:142
high N:139
years
log−rank test p-value = 0.2

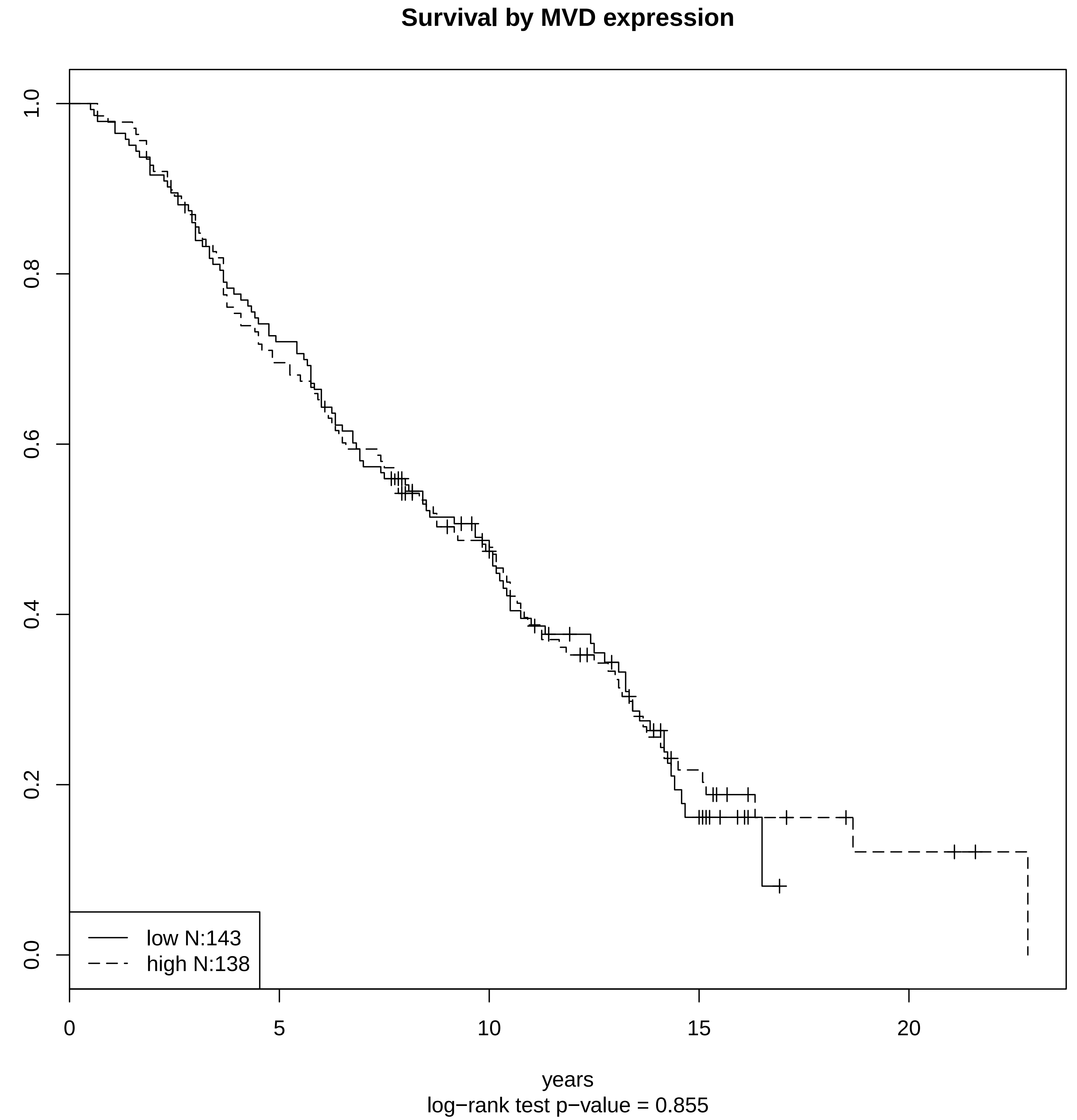
Survival by MVD expression
1.0
0.8
0.6
0.4
0.2
0.0
0
5
10
15
20
low N:143
high N:138
years
log−rank test p−value = 0.855

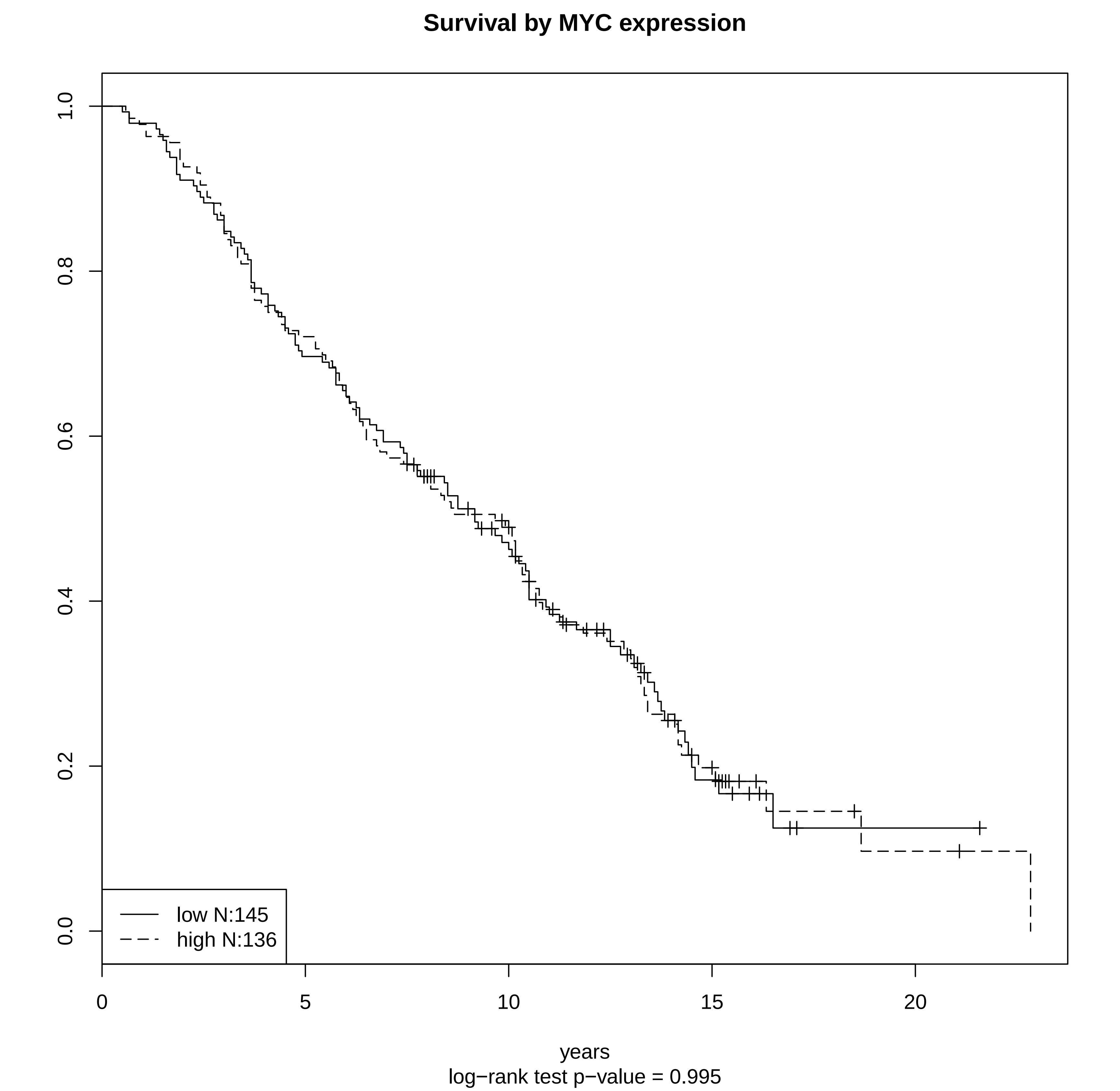

Survival by MYC expression
1.0
0.8
0.6
0.4
0.2
0.0
0
5
10
15
20
years
log−rank test p−value = 0.995
low N:145
high N:136

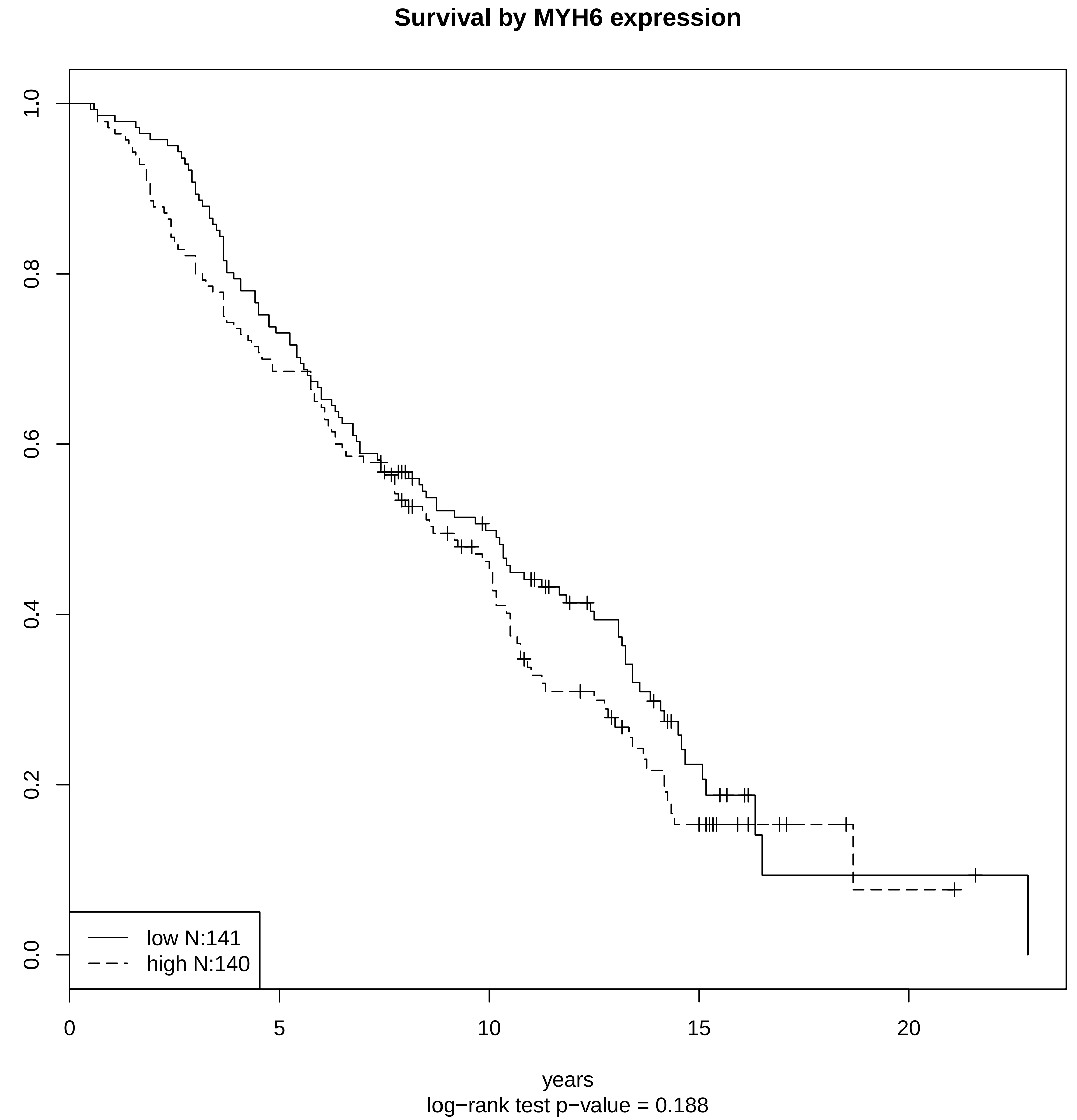
Survival by MYH6 expression
1.0
0.8
0.6
0.4
0.2
0.0
0
5
10
15
20
years
low N:141
high N:140
log−rank test p−value = 0.188

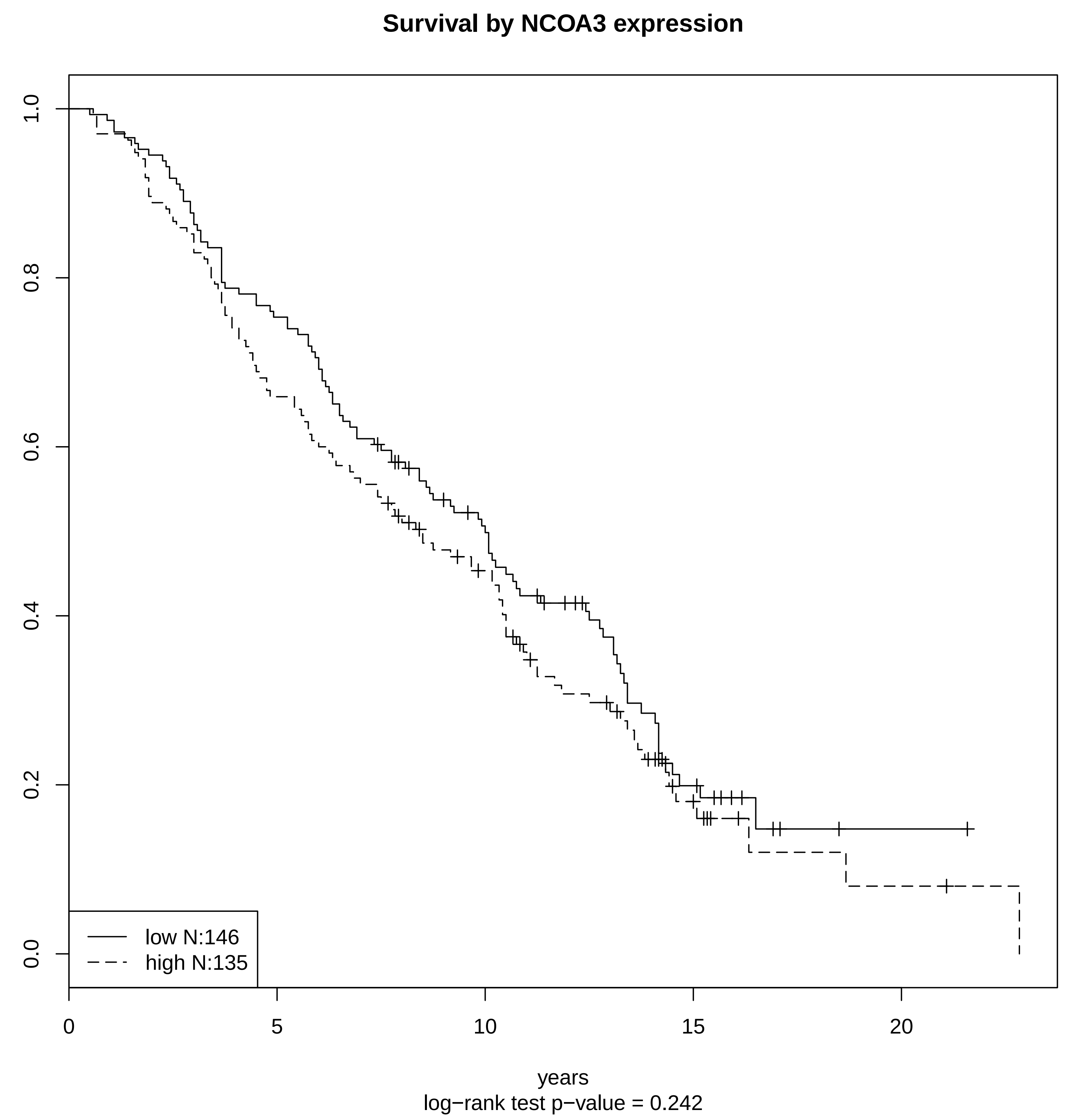
Survival by NCOA3 expression
1.0
0.8
0.6
0.4
0.2
0.0
0
5
10
15
20
years
low N:146
high N:135
log−rank test p−value = 0.242

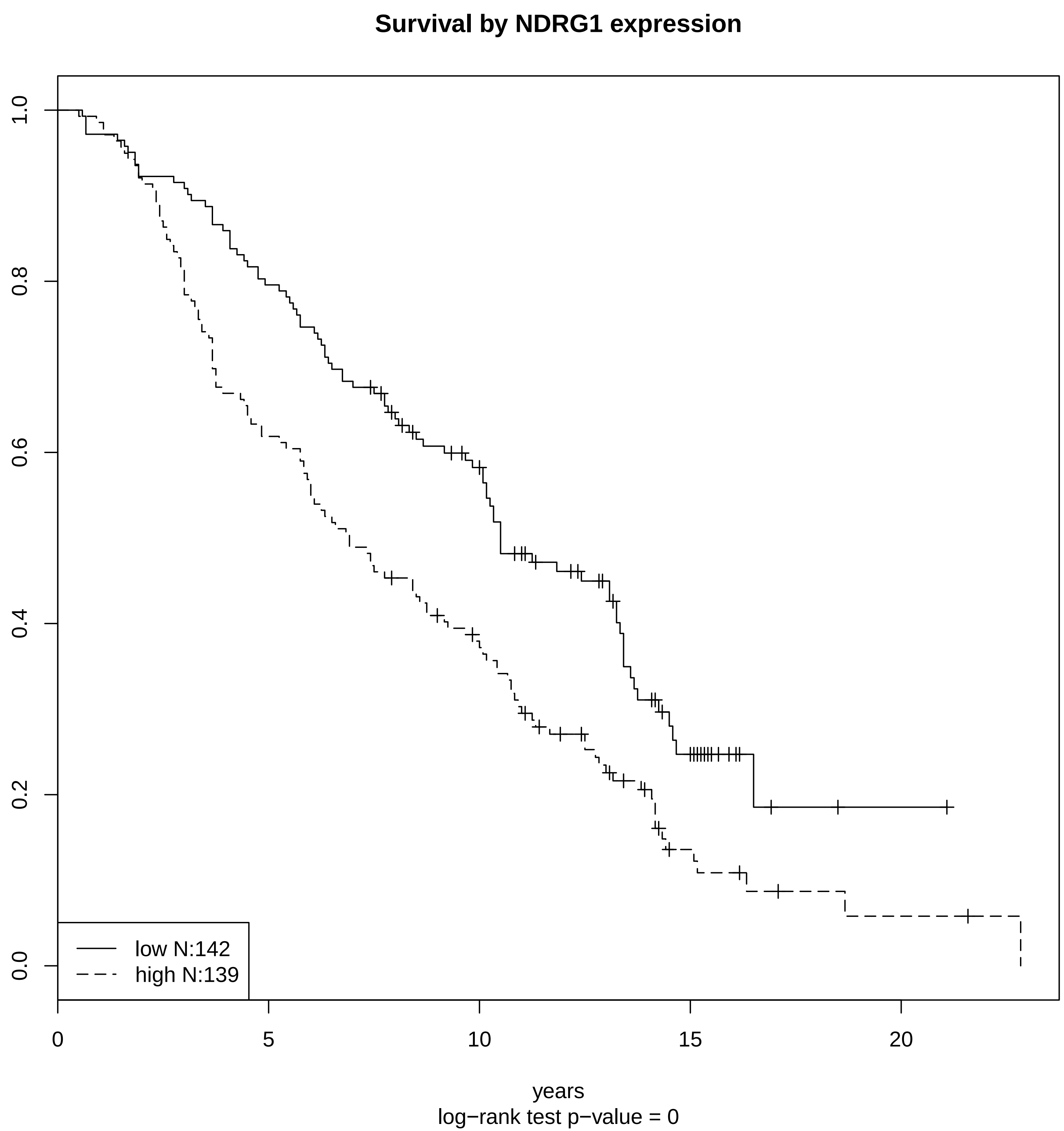
Survival by NDRG1 expression
1.0
0.8
0.6
0.4
0.2
0.0
0
5
10
15
20
years
log−rank test p−value = 0
low N:142
high N:139

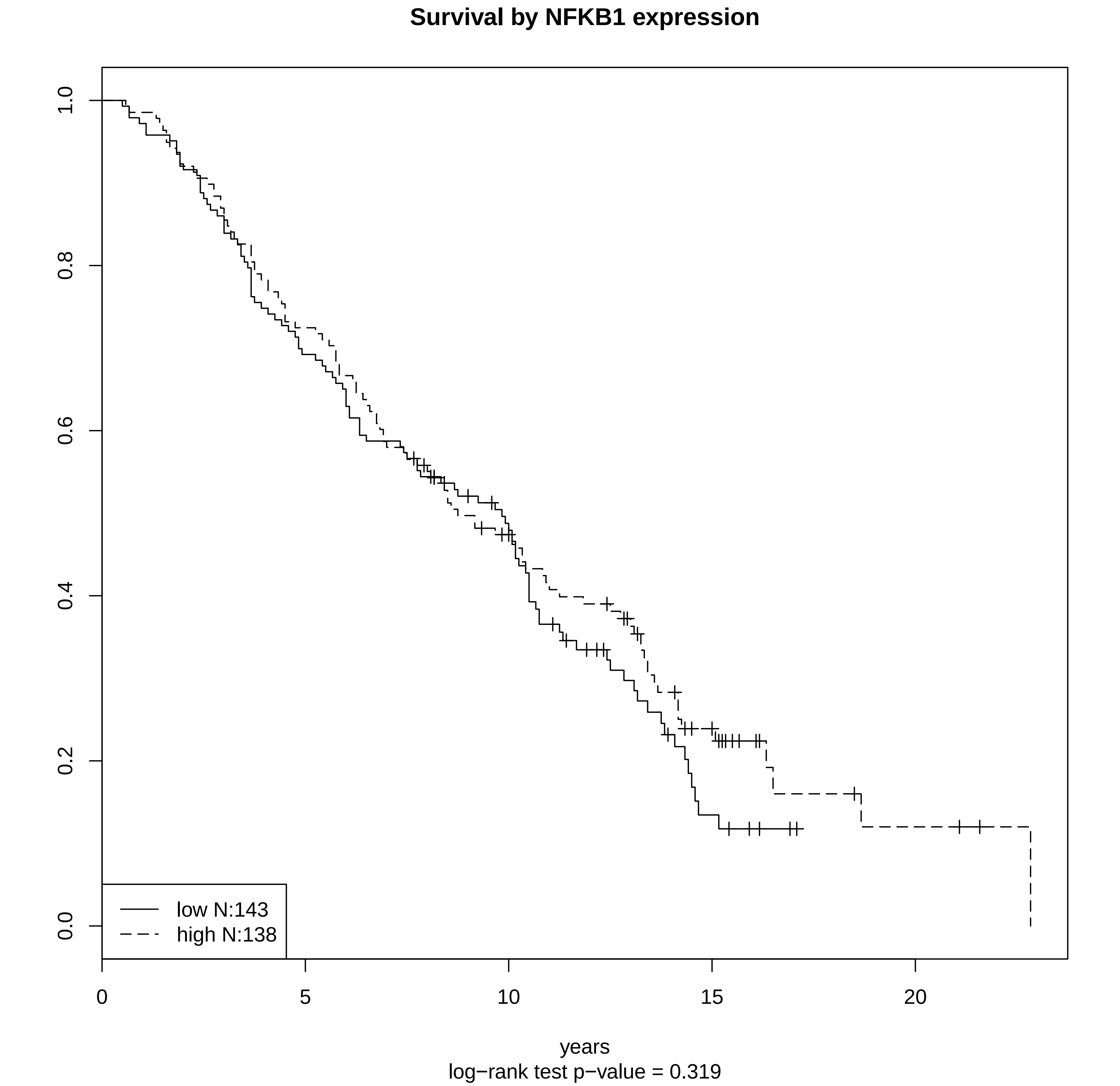

Survival by NFKB1 expression
1.0
0.8
0.6
0.4
0.2
0.0
0
5
10
15
20
low N:143
high N:138
years
log−rank test p−value = 0.319

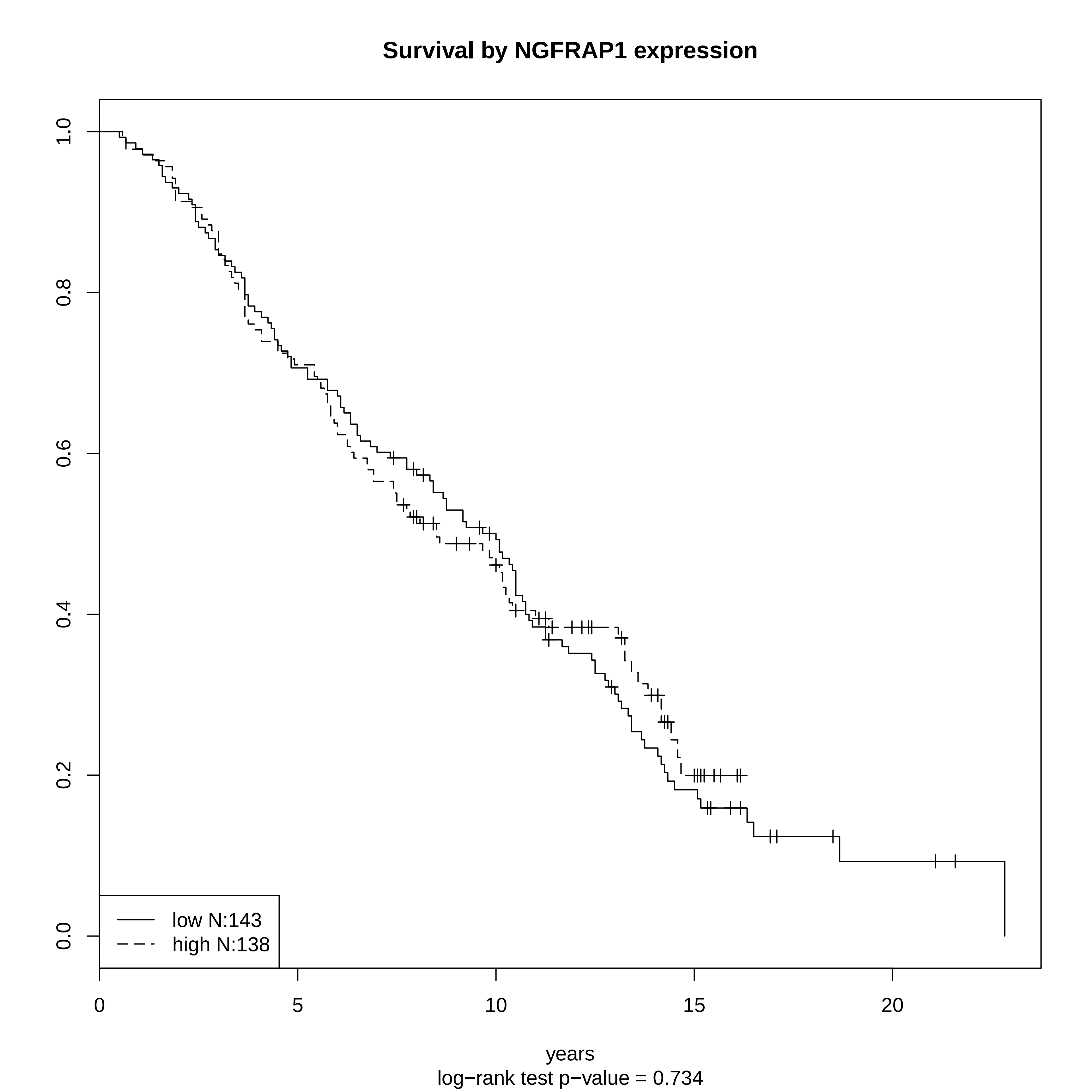
Survival by NGFRAP1 expression
1.0
0.8
0.6
0.4
0.2
0.0
0
5
10
15
20
low N:143
high N:138
years
log−rank test p−value = 0.734

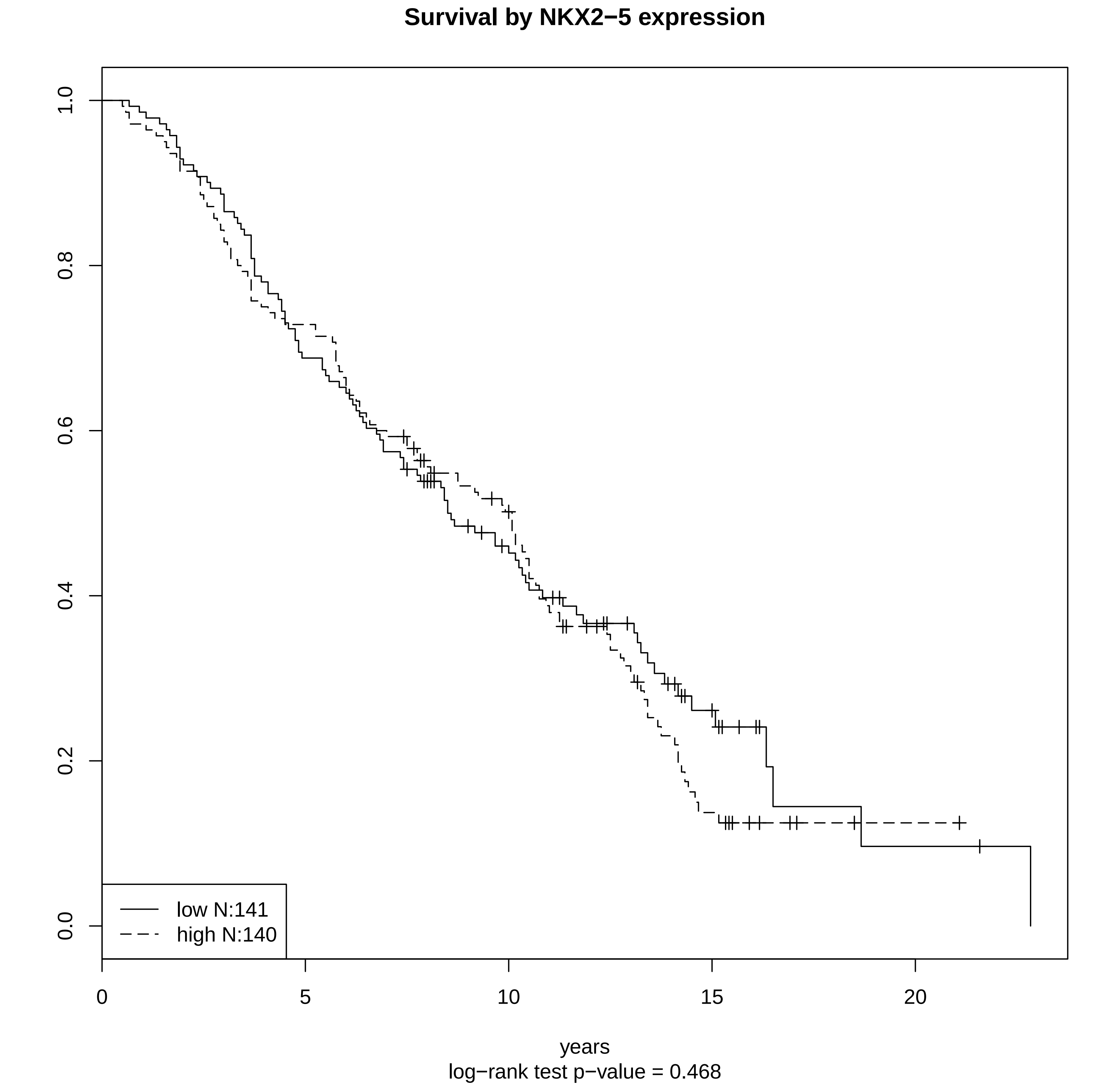

Survival by NKX2−5 expression
1.0
0.8
0.6
0.4
0.2
0.0
0
5
10
15
20
years
log−rank test p−value = 0.468
low N:141
high N:140

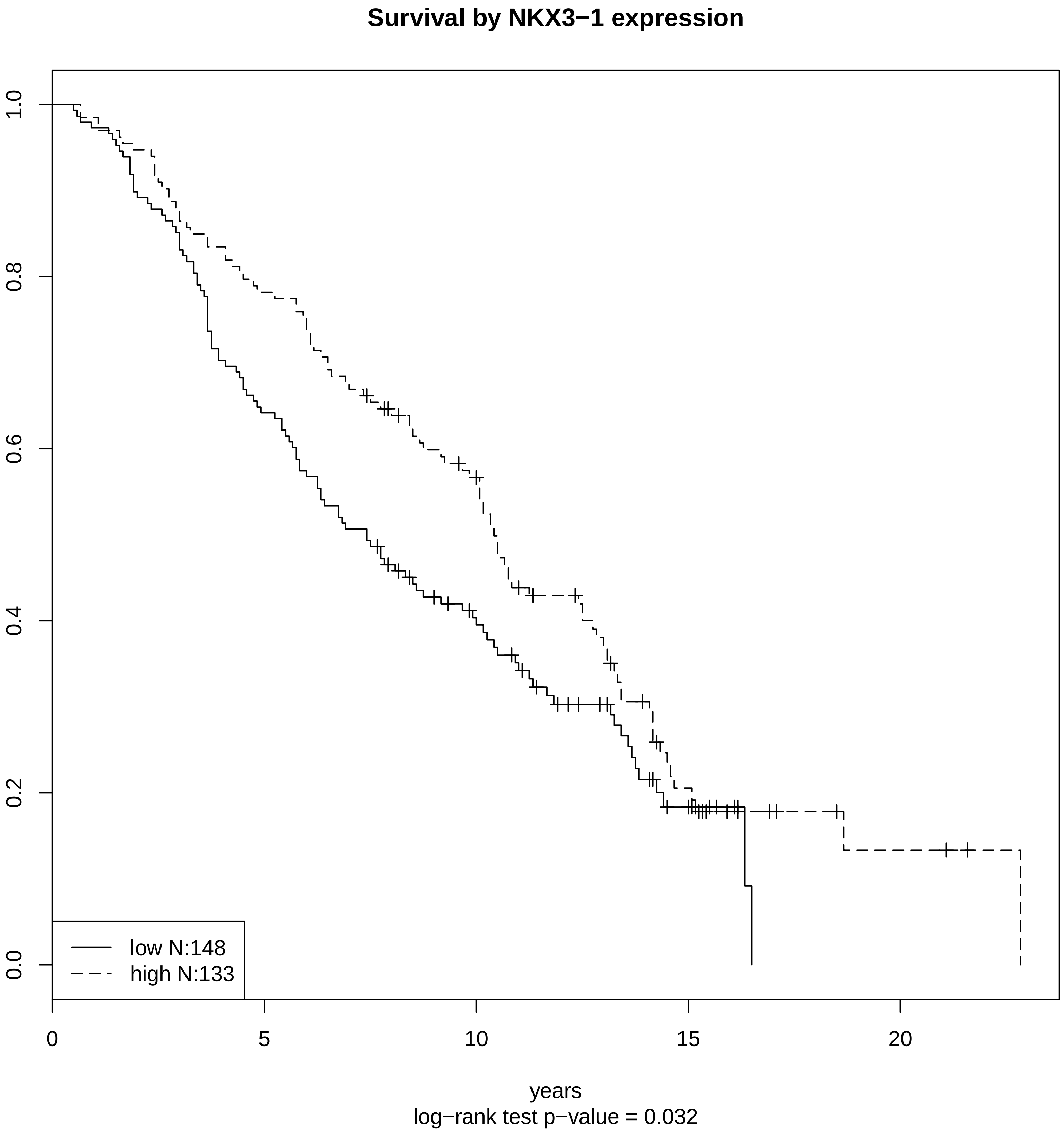
Survival by NKX3−1 expression
1.0
0.8
0.6
0.4
0.2
0.0
0
5
10
15
20
low N:148
high N:133
years
log−rank test p−value = 0.032

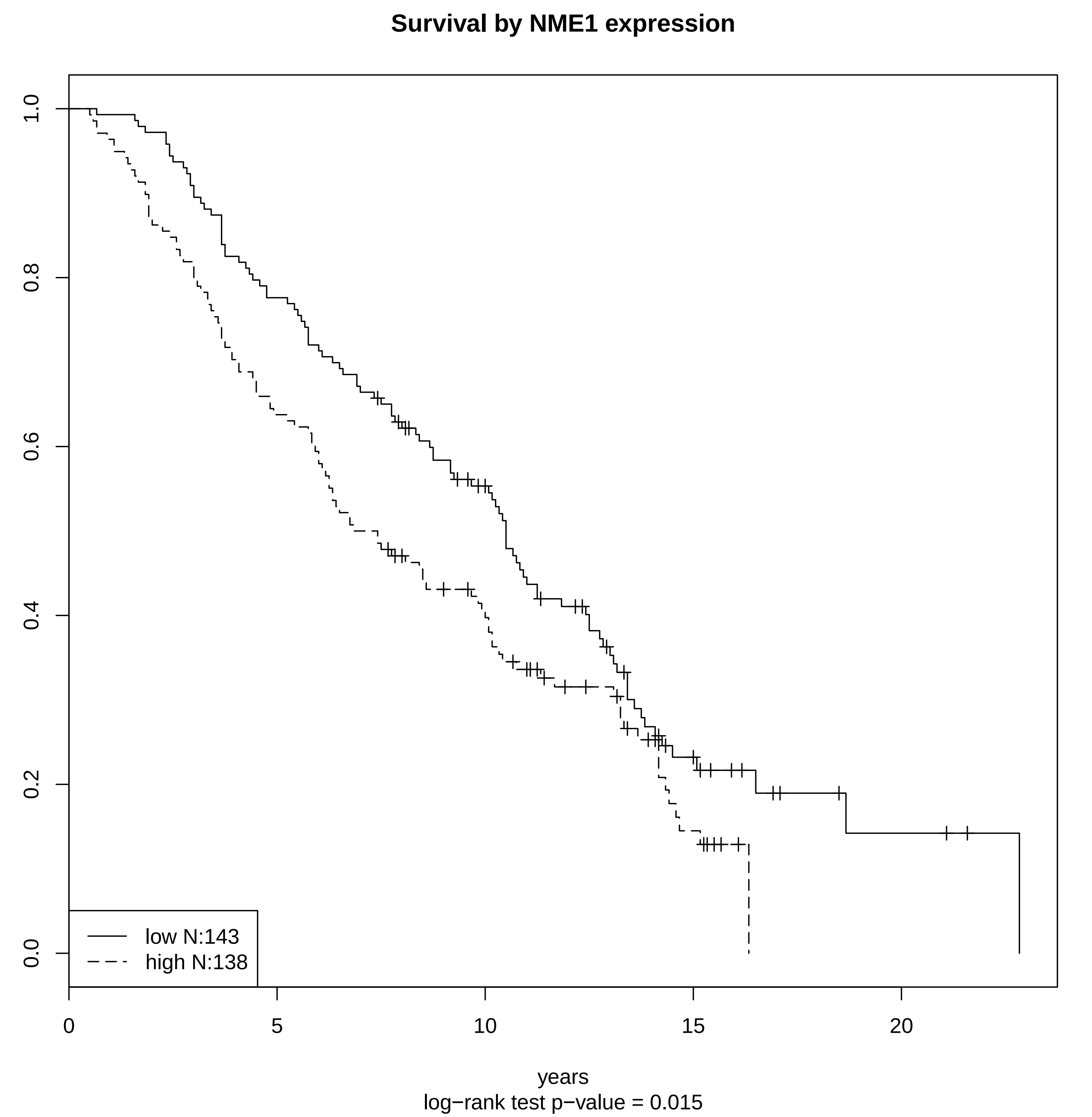
Survival by NME1 expression
1.0
0.8
0.6
0.4
0.2
0.0
0
5
10
15
20
low N:143
high N:138
years
log−rank test p−value = 0.015

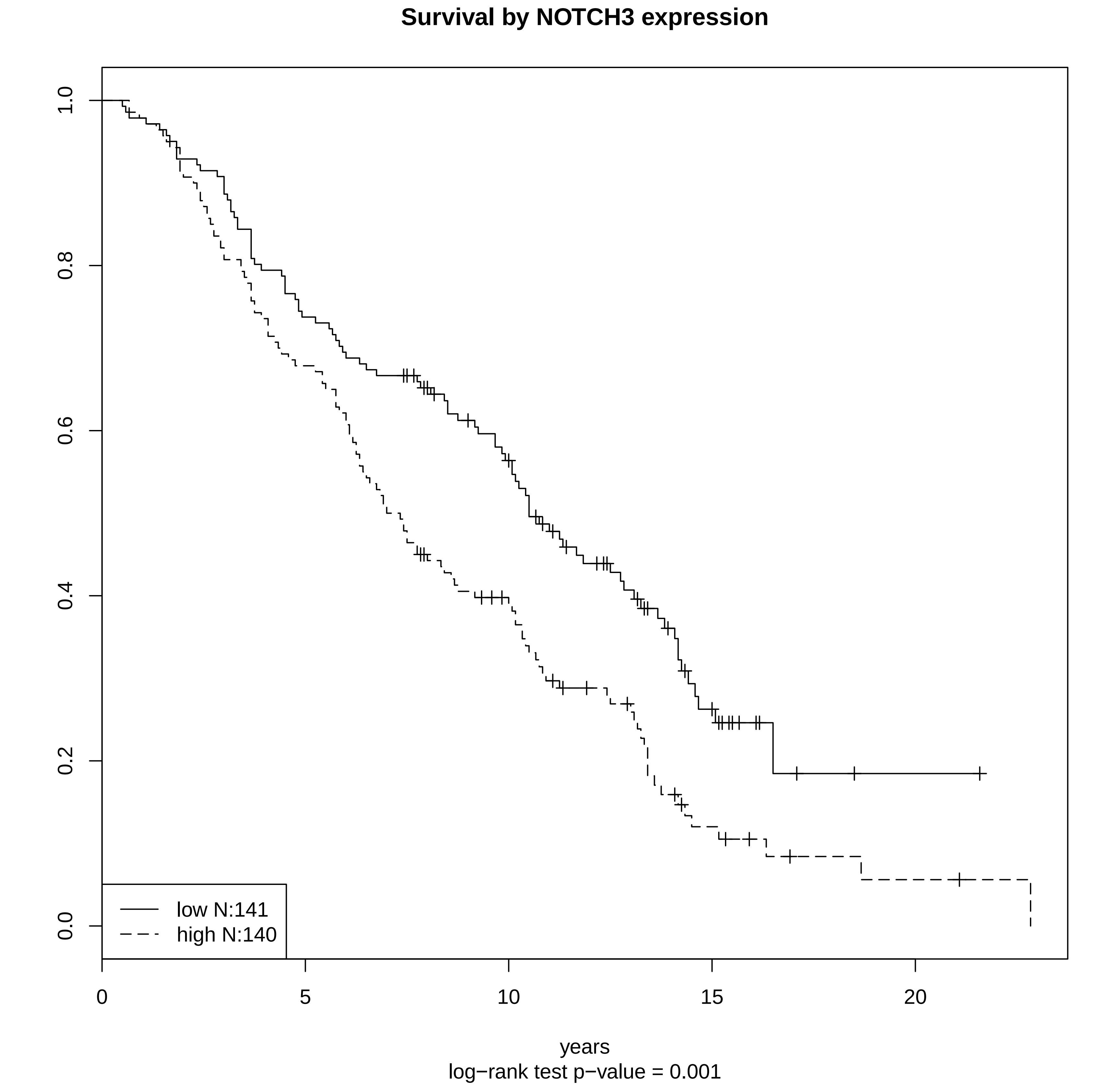
Survival by NOTCH3 expression
1.0
0.8
0.6
0.4
0.2
0.0
low N:141
high N:140
0
5
10
15
20
years
log−rank test p−value = 0.001

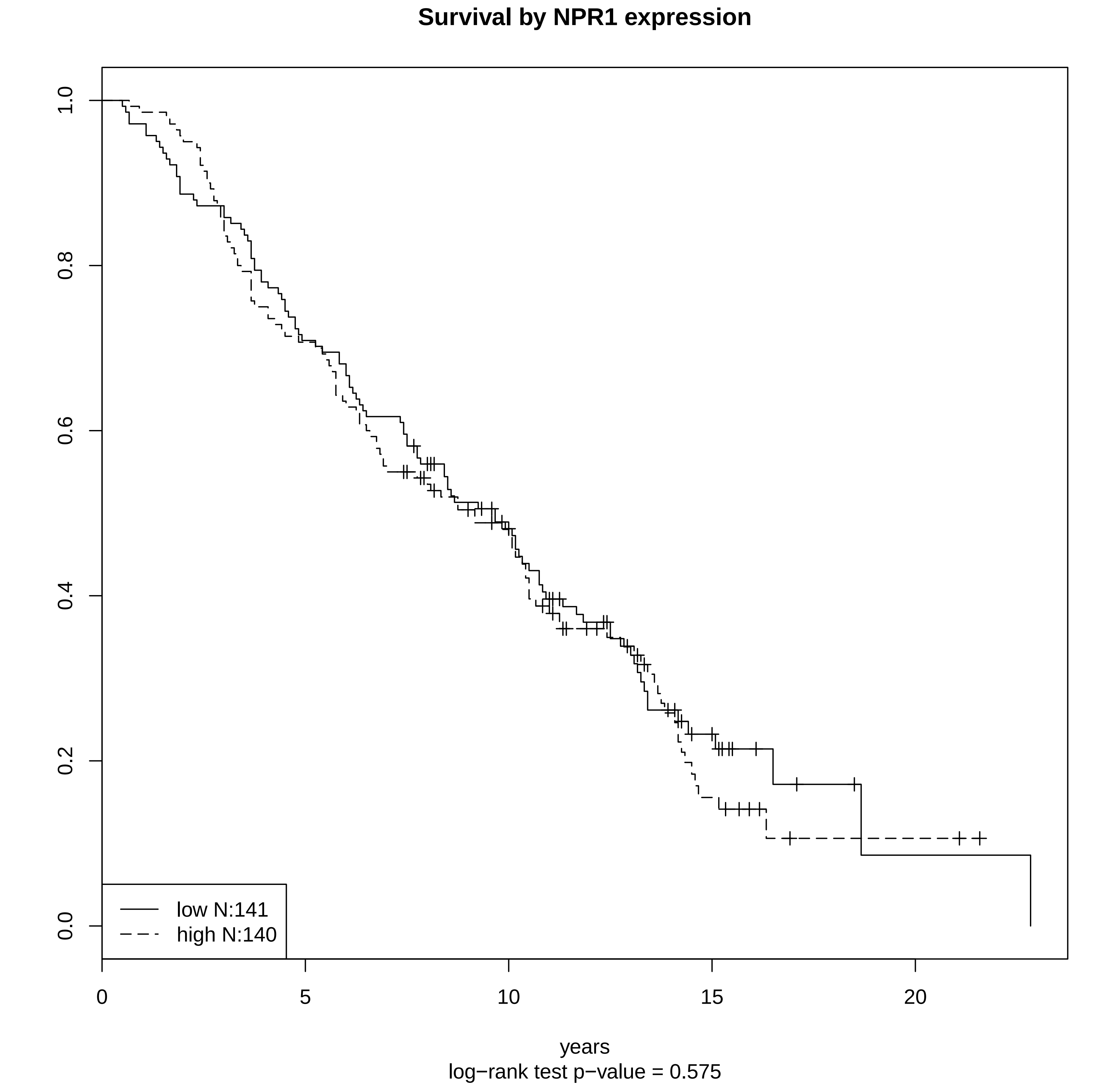

Survival by NPR1 expression
1.0
0.8
0.6
0.4
0.2
0.0
low N:141
high N:140
0
5
10
15
20
years
log−rank test p−value = 0.575

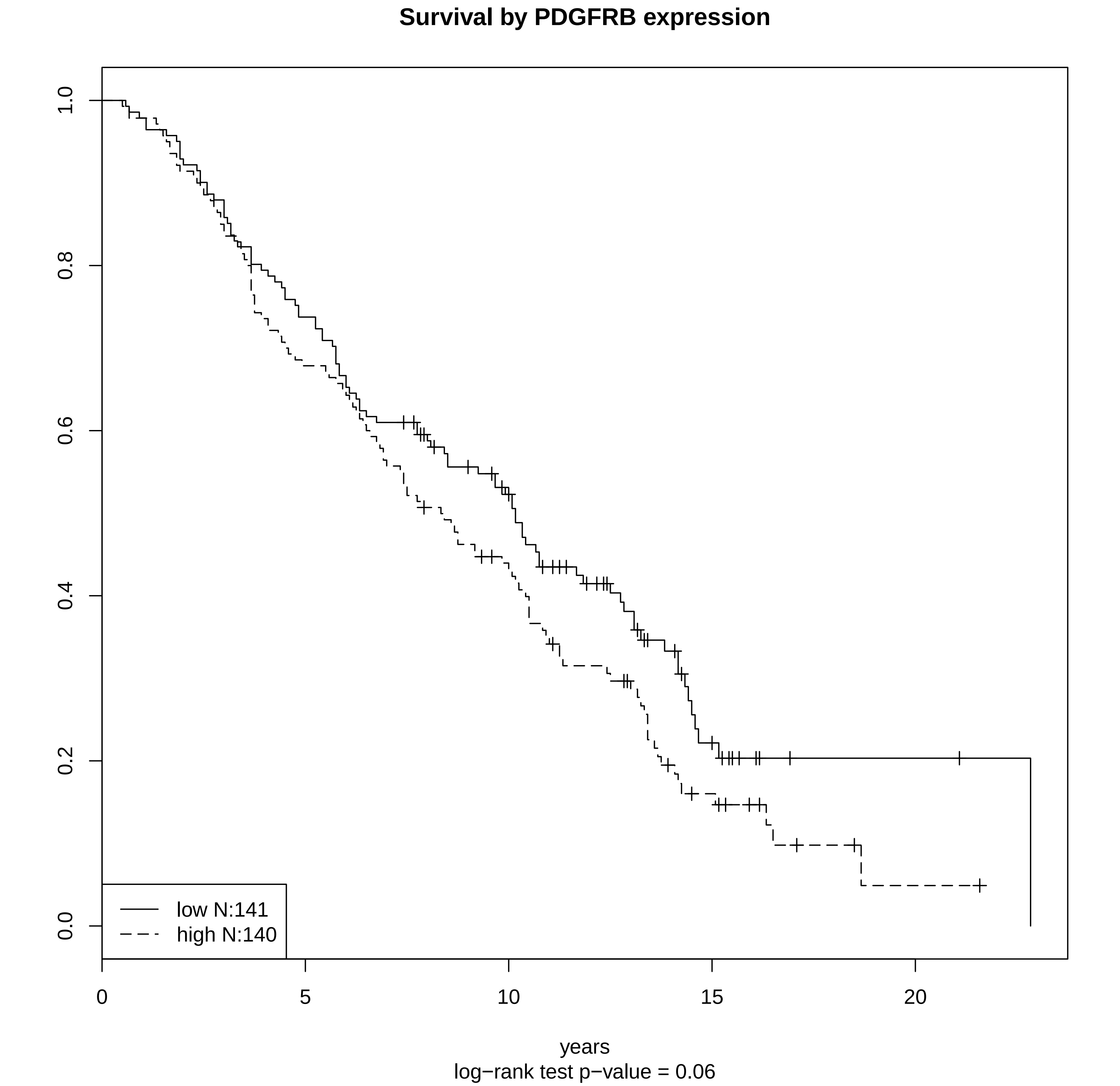
Survival by PDGFRB expression
1.0
0.8
0.6
0.4
0.2
0.0
0
5
10
15
20
low N:141
high N:140
years
log−rank test p−value = 0.06

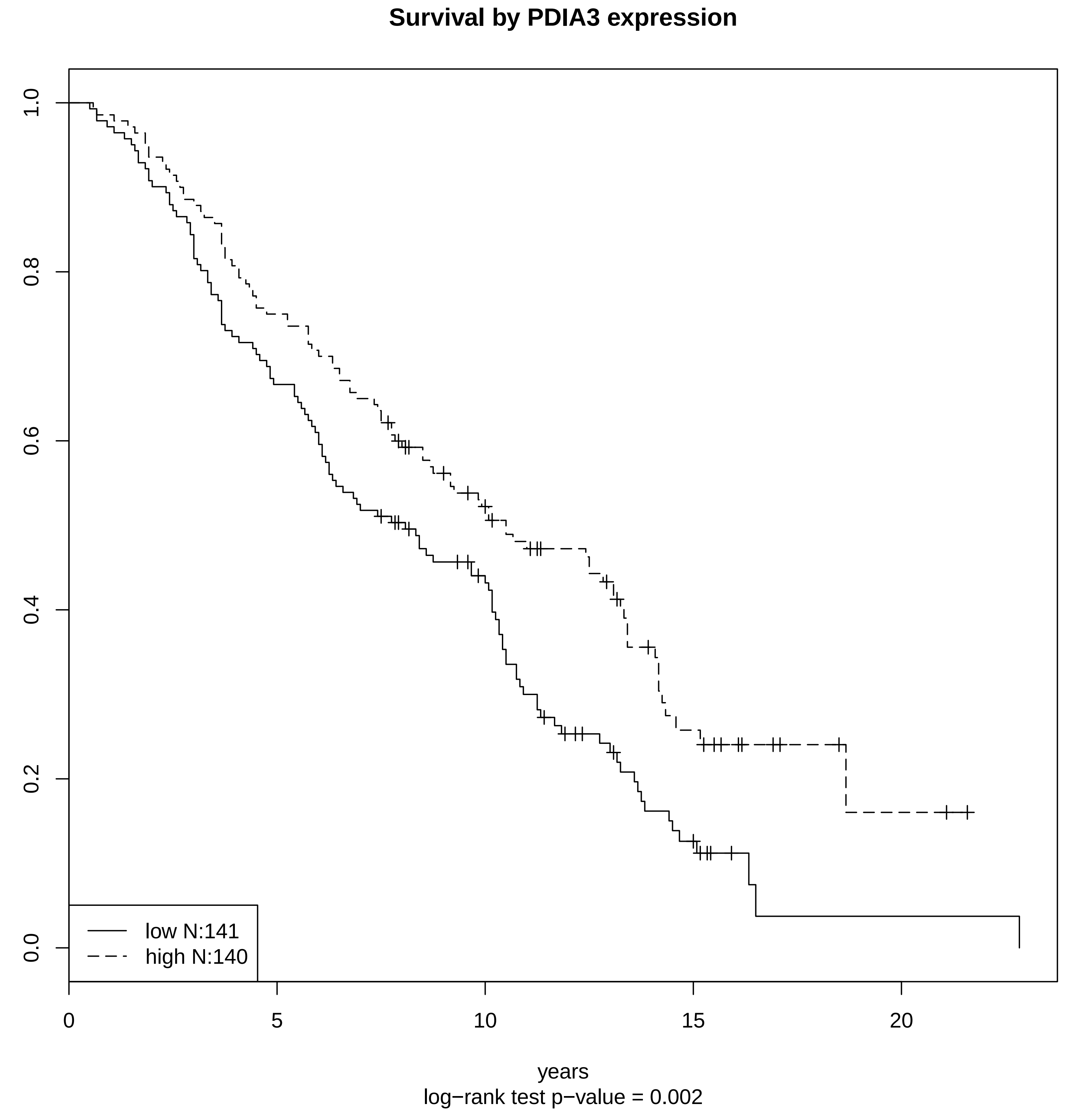
Survival by PDIA3 expression
1.0
0.8
0.6
0.4
0.2
0.0
0
5
10
15
20
low N:141
high N:140
years
log−rank test p−value = 0.002

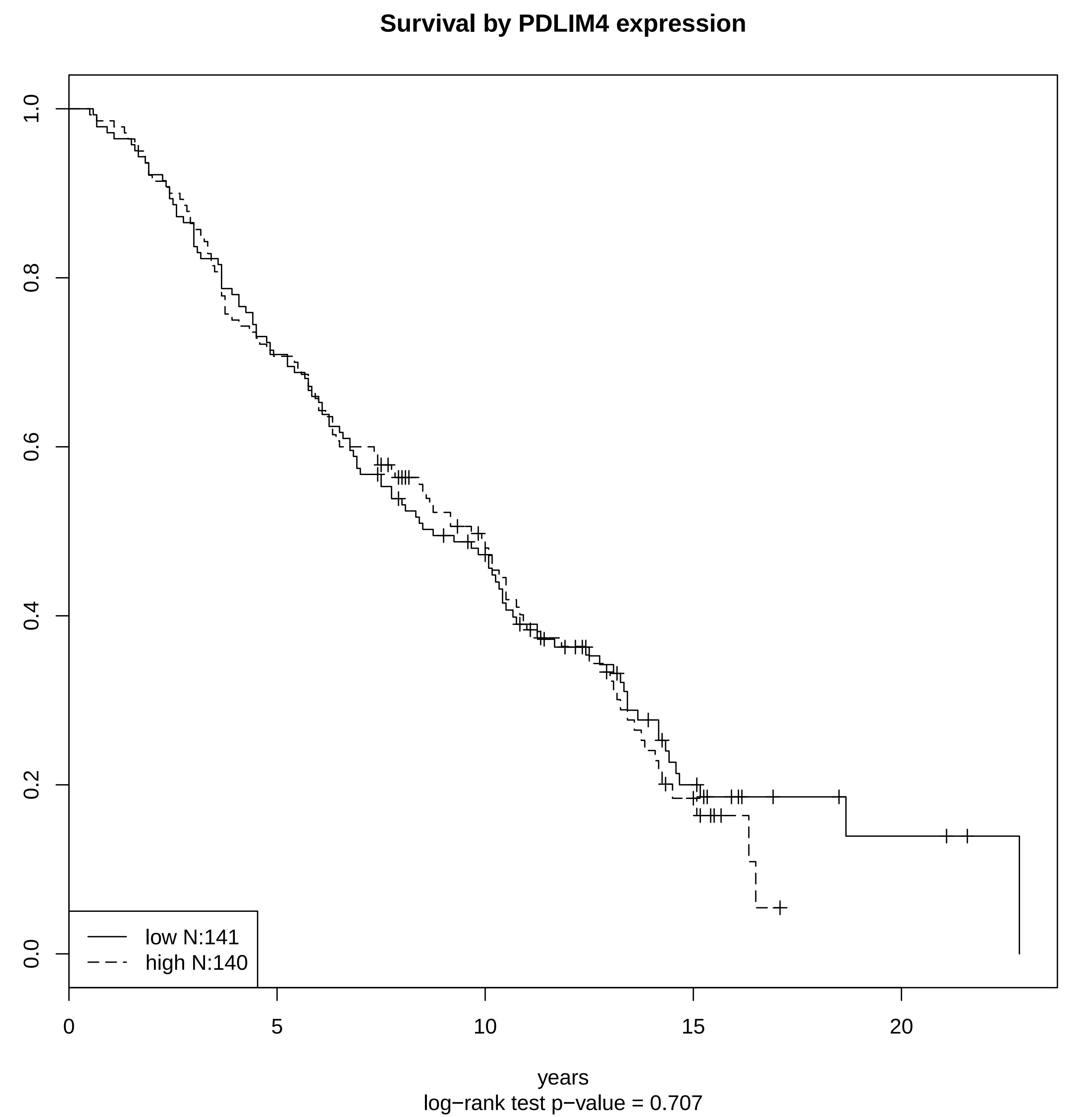
Survival by PDLIM4 expression
1.0
0.8
0.6
0.4
0.2
0.0
0
5
10
15
20
years
log−rank test p−value = 0.707
low N:141
high N:140

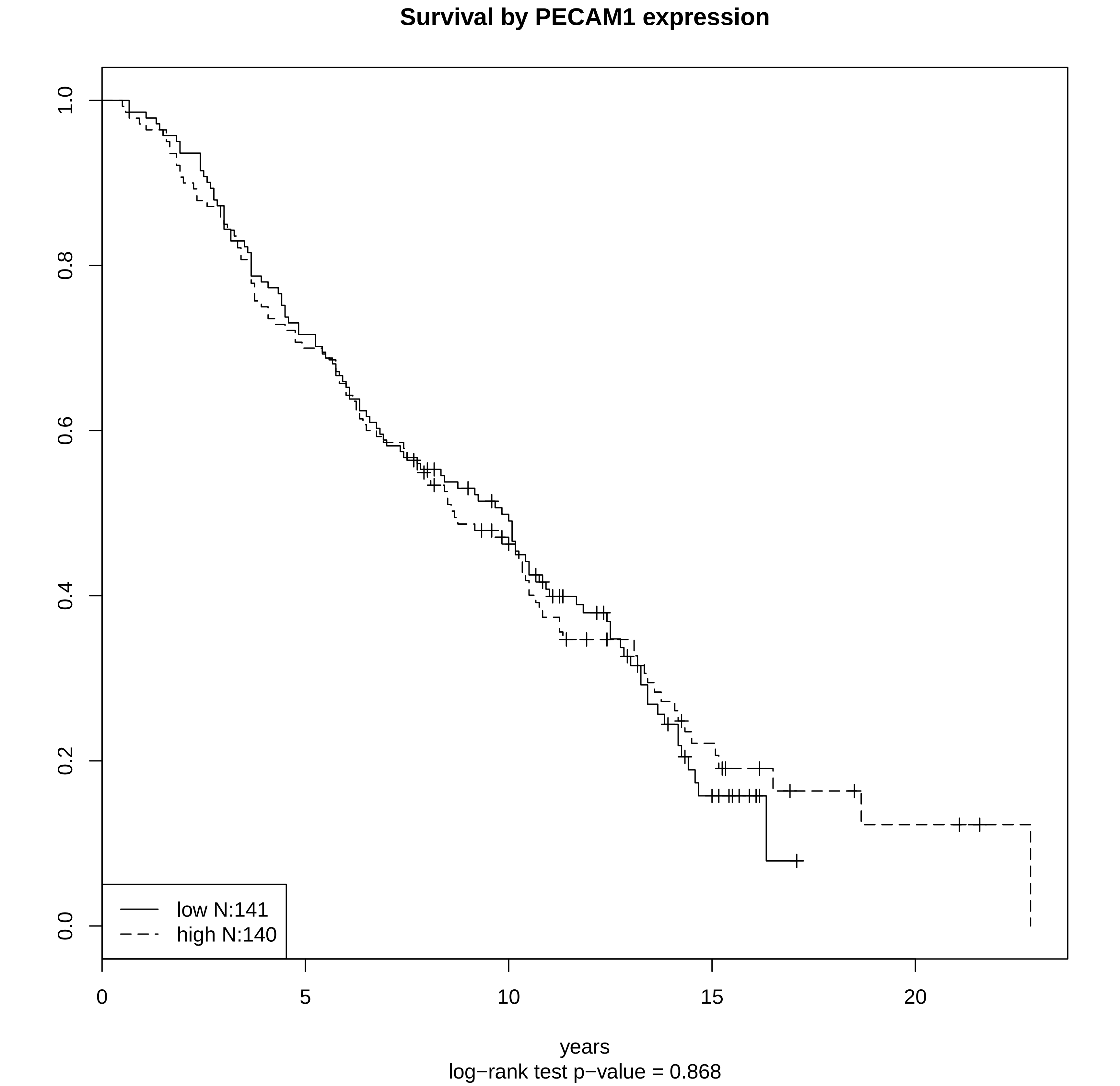

Survival by PECAM1 expression
1.0
0.8
0.6
0.4
0.2
0.0
0
5
10
15
20
low N:141
high N:140
years
log−rank test p−value = 0.868

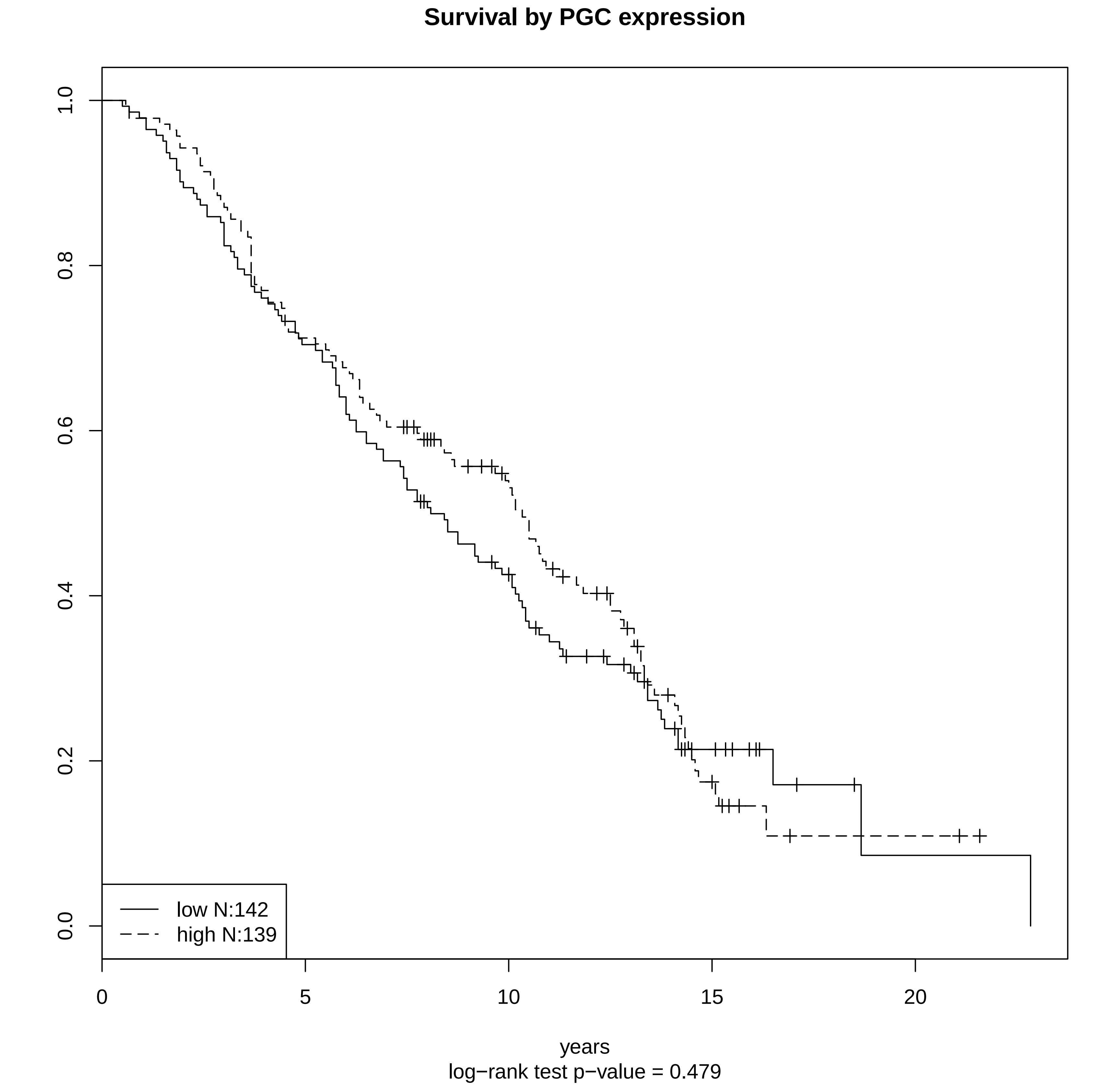
Survival by PGC expression
1.0
0.8
0.6
0.4
0.2
0.0
0
5
10
15
20
low N:142
high N:139
years
log−rank test p−value = 0.479

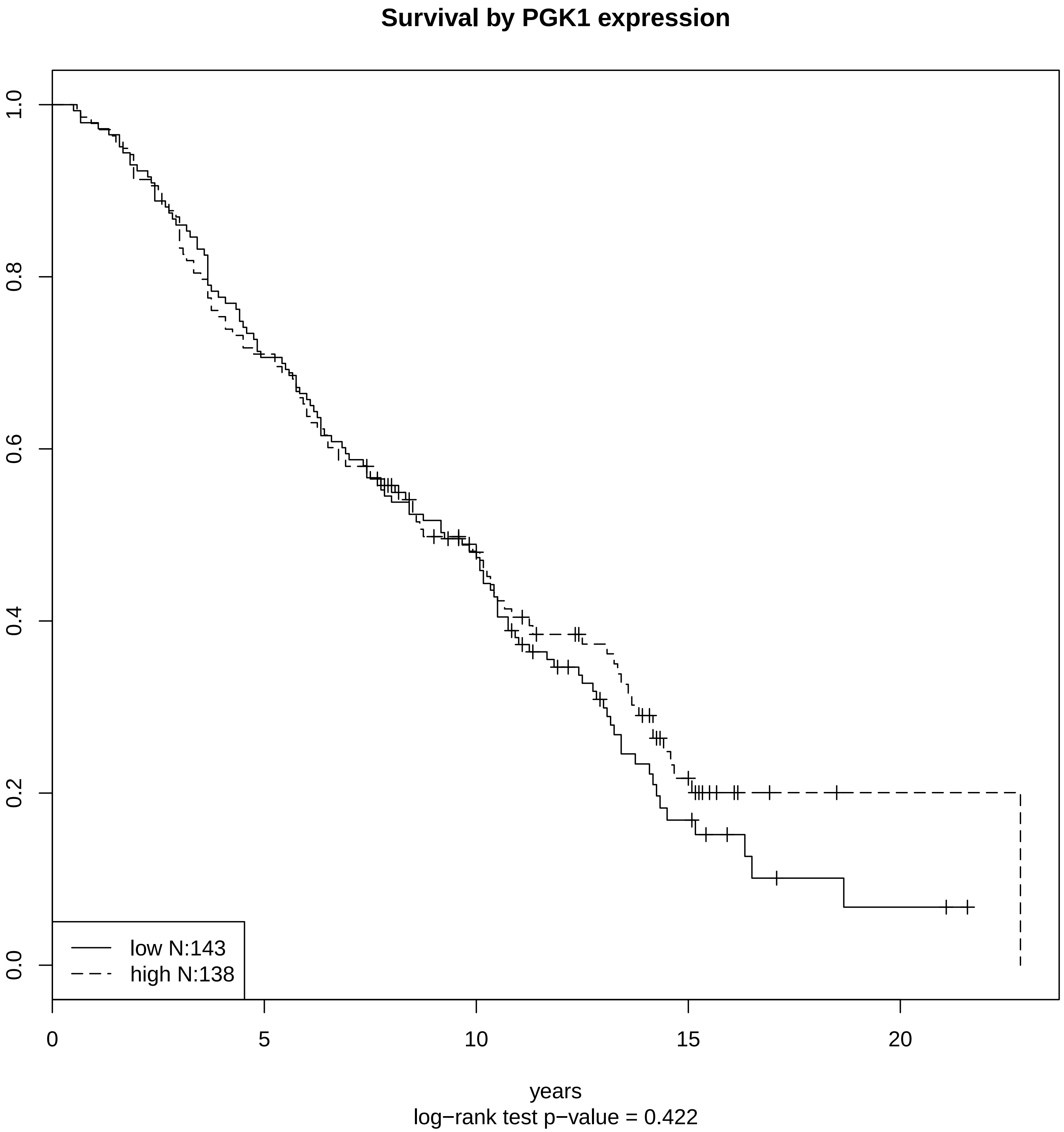

Survival by PGK1 expression
0.0
0.2
0.4
0.6
0.8
1.0
0
5
10
15
20
years
low N:143
high N:138
log−rank test p−value = 0.422

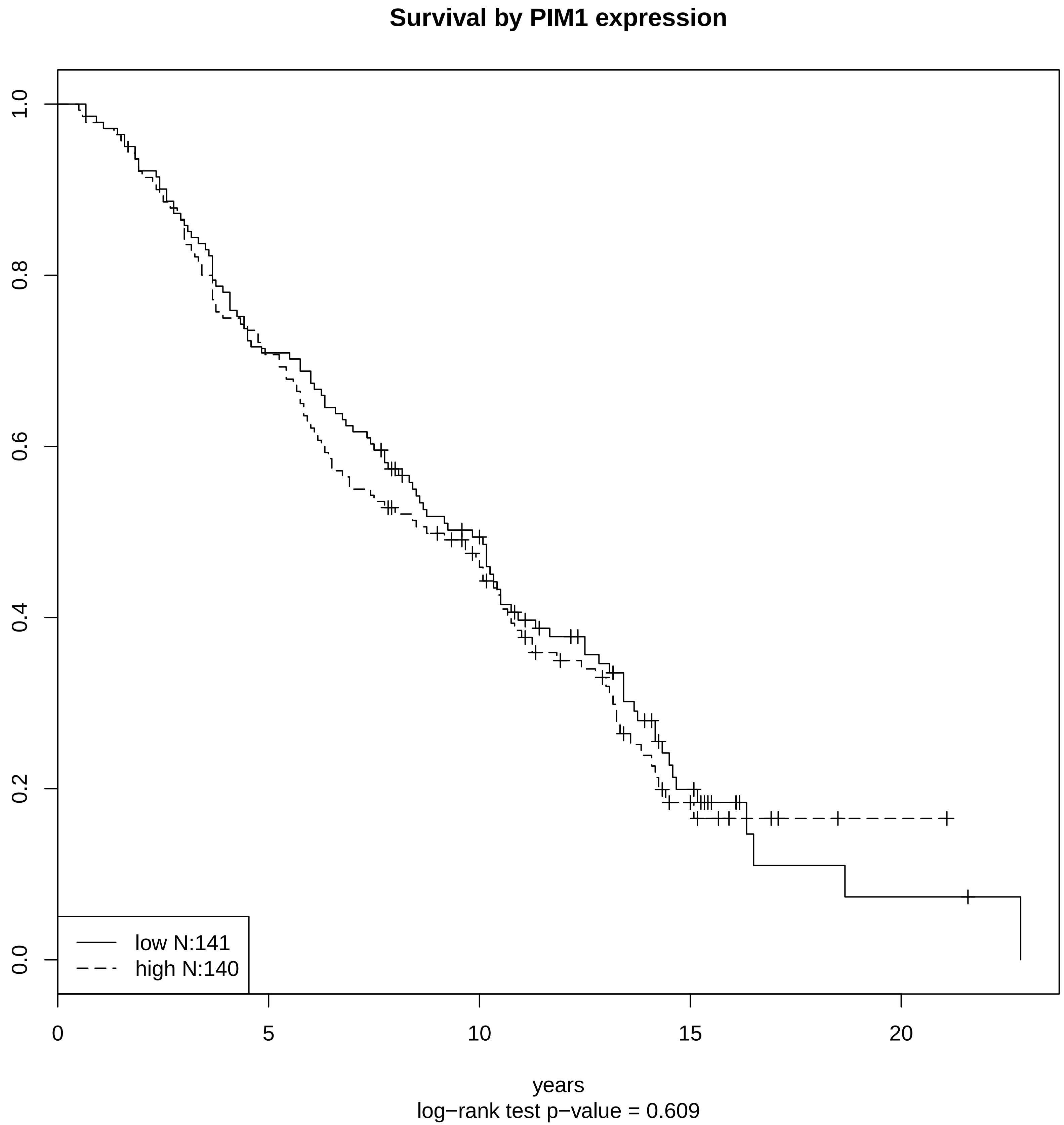
Survival by PIM1 expression
1.0
0.8
0.6
0.4
0.2
0.0
0
5
10
15
20
years
log−rank test p−value = 0.609
low N:141
high N:140

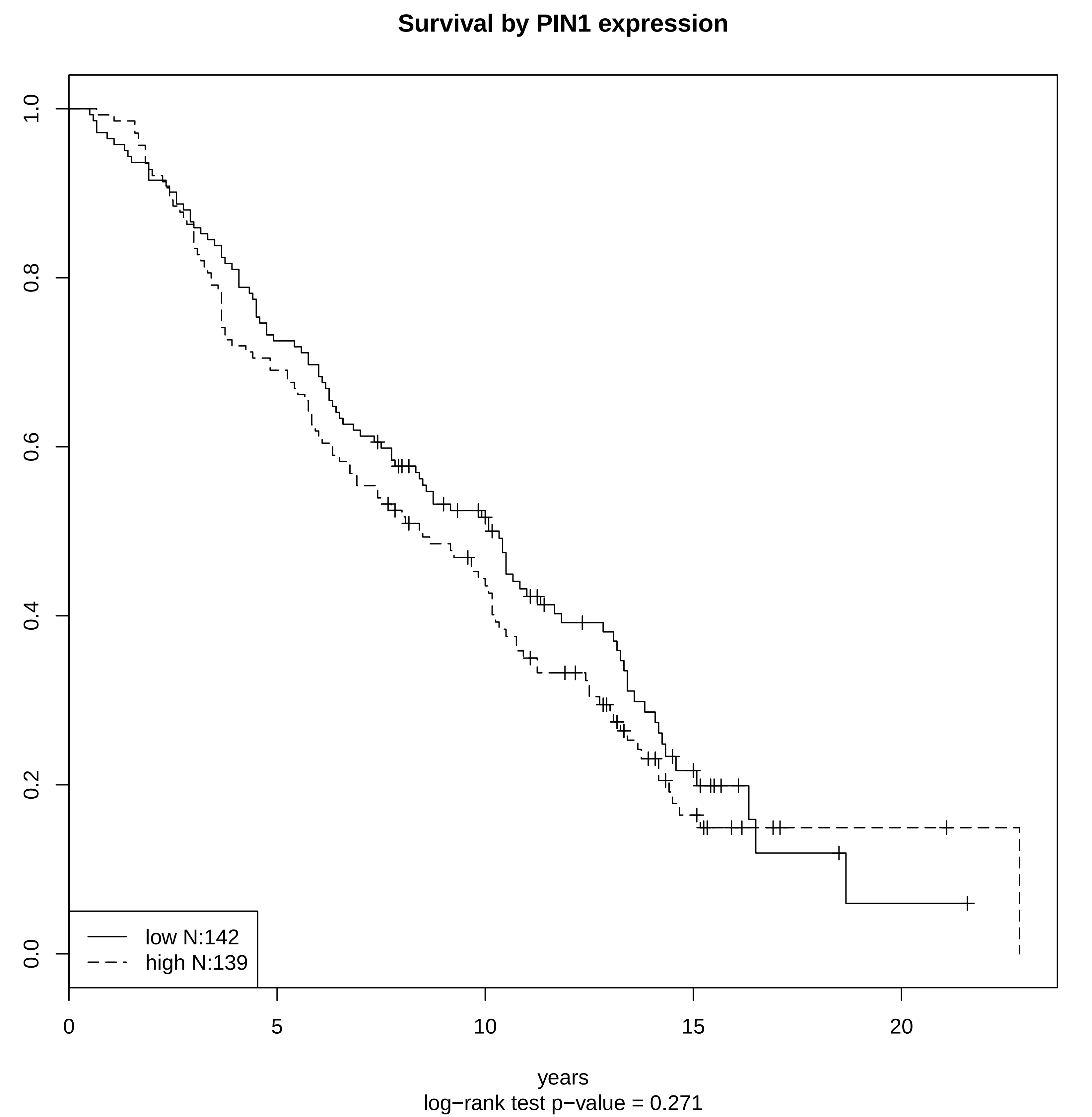
Survival by PIN1 expression
1.0
0.8
0.6
0.4
0.2
0.0
0
5
10
15
20
years
log−rank test p−value = 0.271
low N:142
high N:139

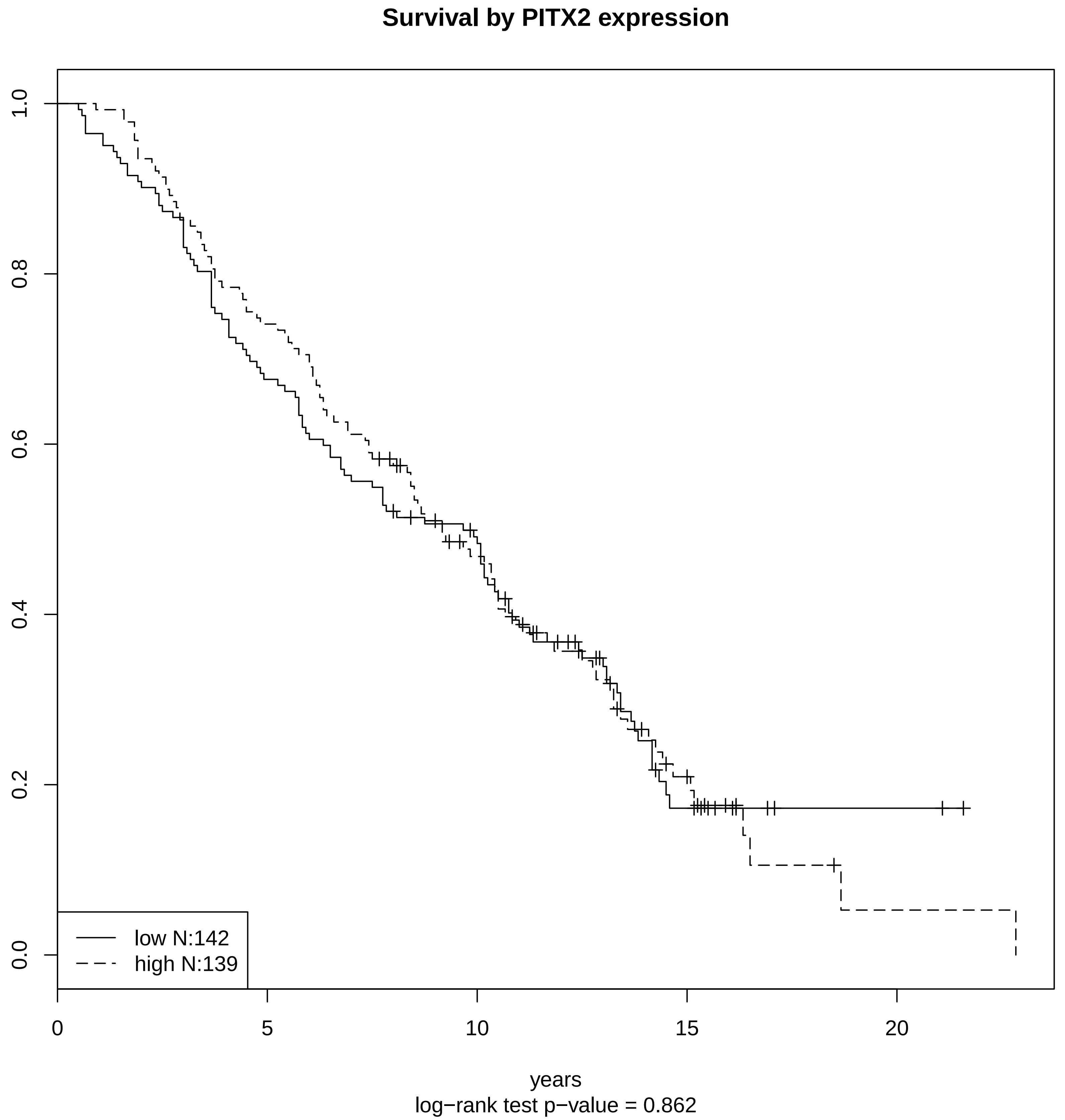
Survival by PITX2 expression
1.0
0.8
0.6
0.4
0.2
0.0
0
5
10
15
20
low N:142
high N:139
years
log−rank test p−value = 0.862

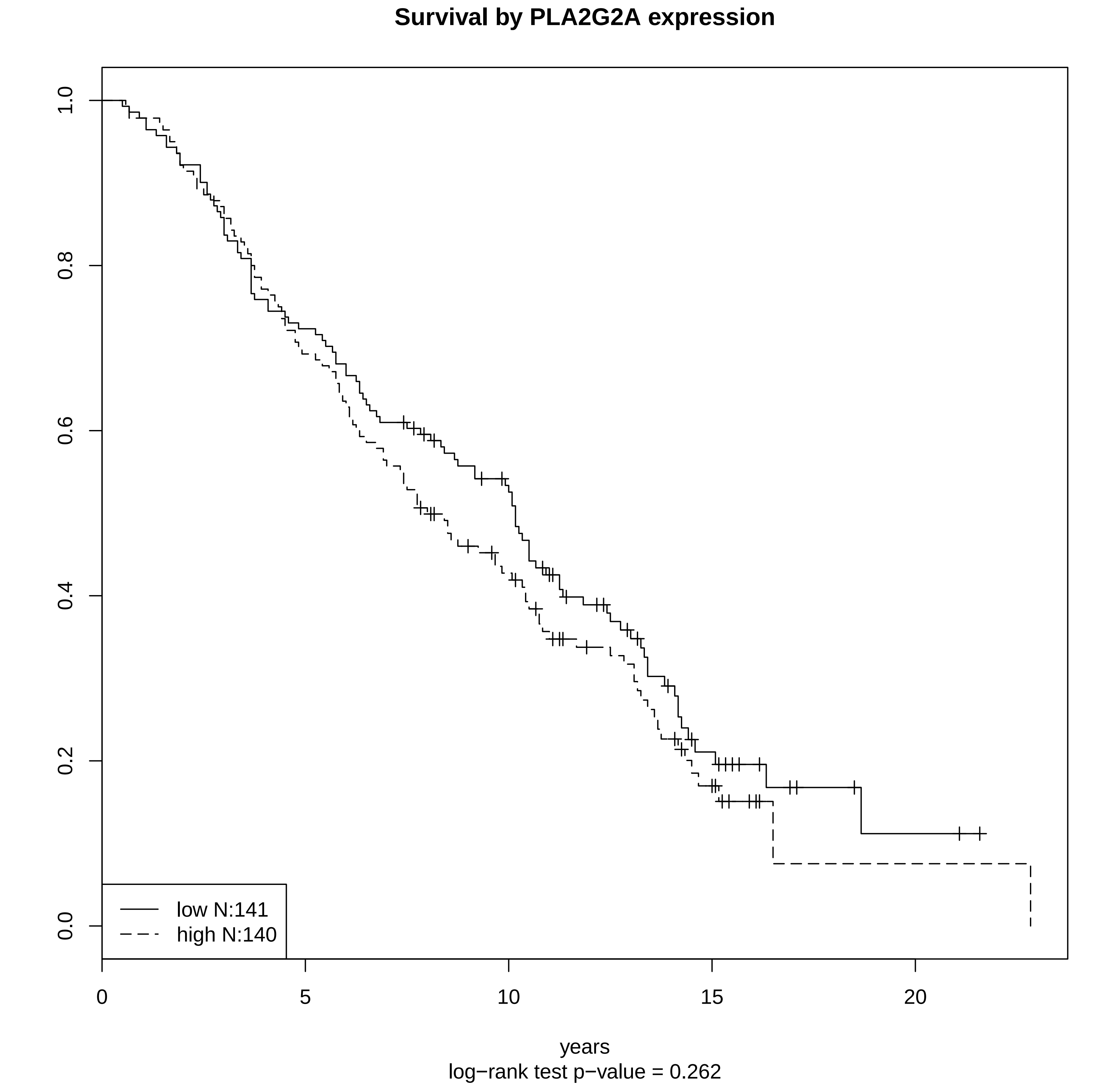

Survival by PLA2G2A expression
1.0
0.8
0.6
0.4
0.2
0.0
0
5
10
15
20
years
log−rank test p−value = 0.262
low N:141
high N:140

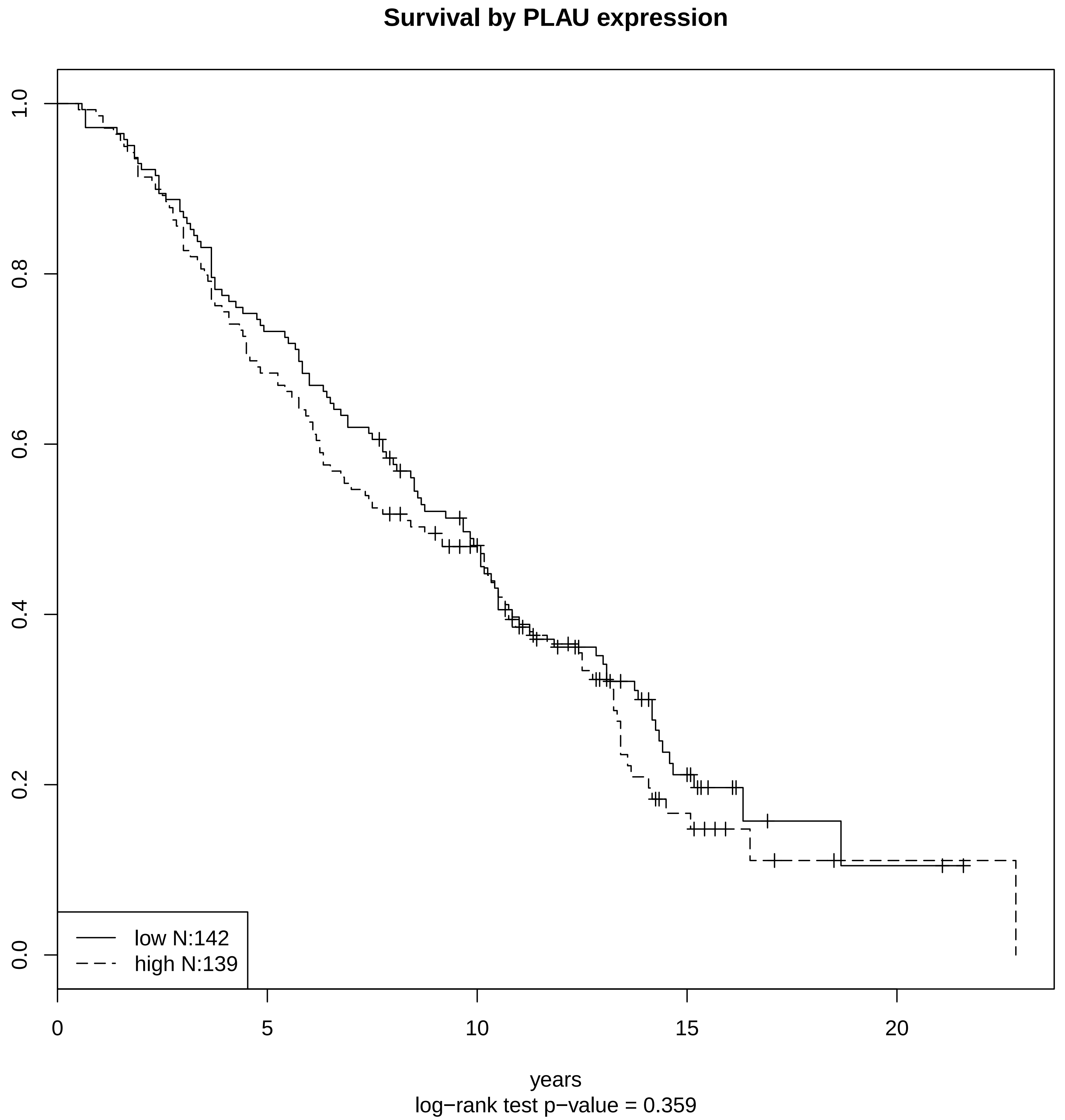
Survival by PLAU expression
1.0
0.8
0.6
0.4
0.2
0.0
0
5
10
15
20
years
low N:142
high N:139
log−rank test p−value = 0.359

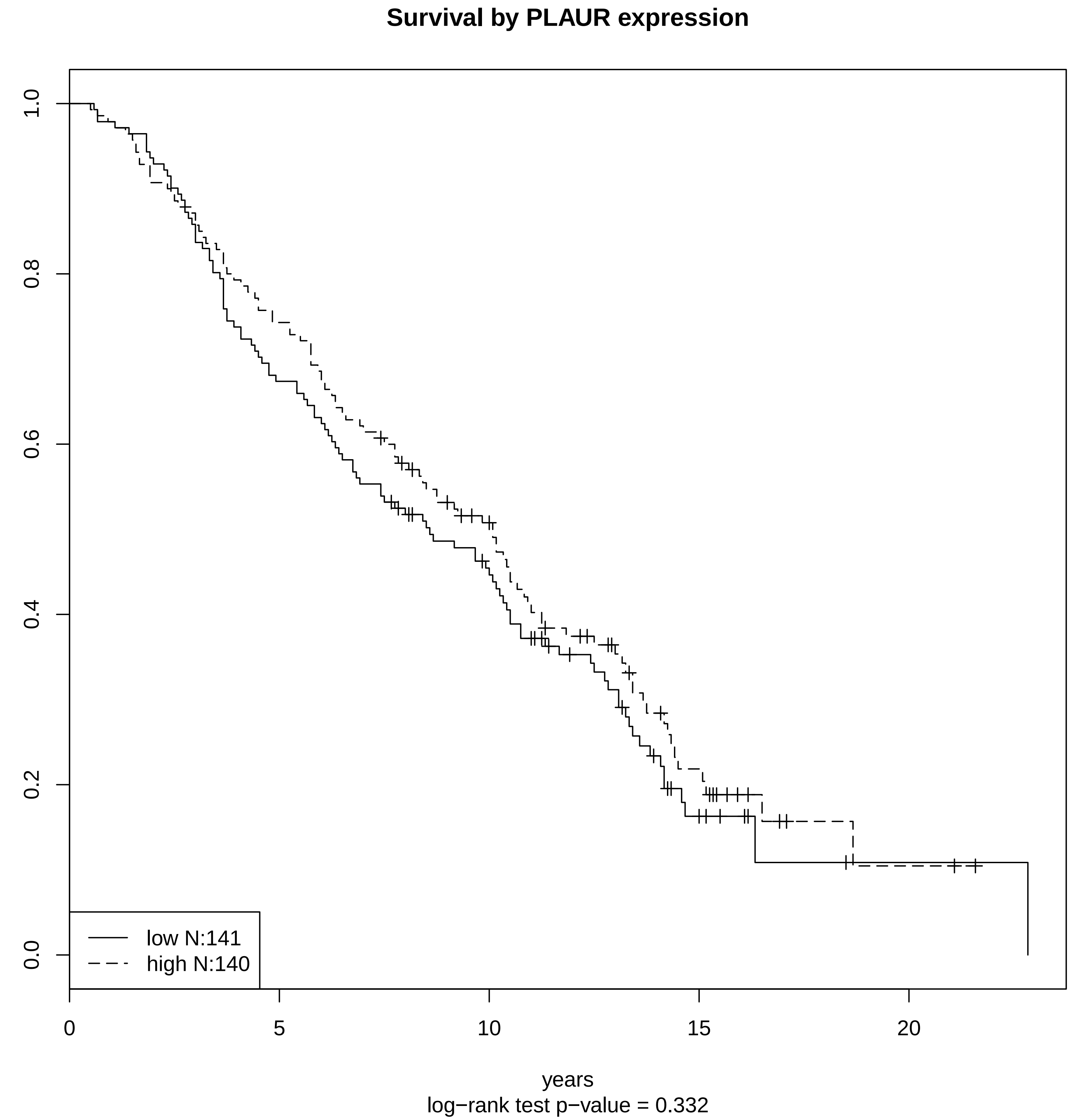

Survival by PLAUR expression
1.0
0.8
0.6
0.4
0.2
0.0
0
5
10
15
20
low N:141
high N:140
years
log−rank test p−value = 0.332

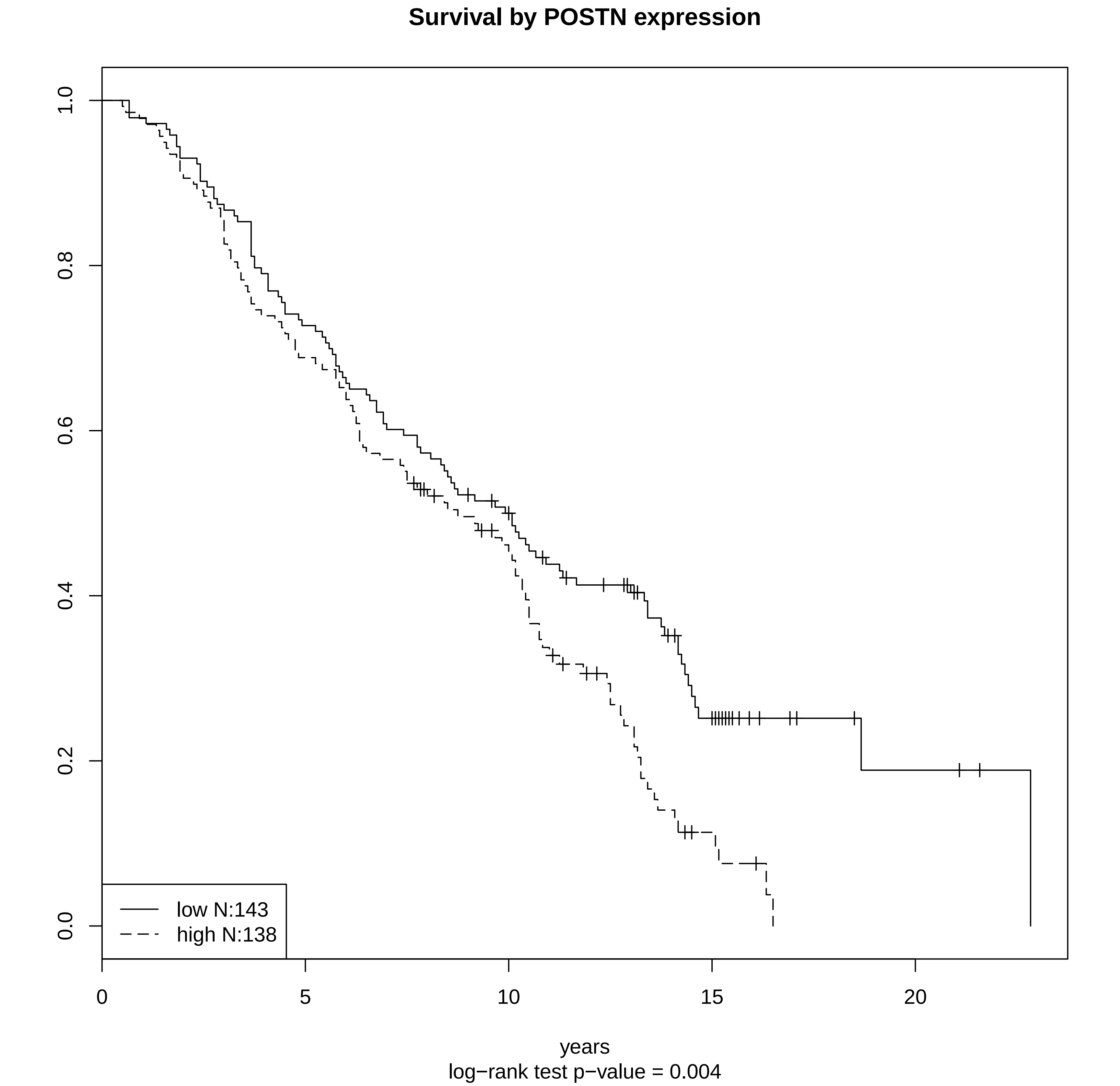
Survival by POSTN expression
1.0
0.8
0.6
0.4
0.2
0.0
0
5
10
15
20
years
log−rank test p−value = 0.004
low N:143
high N:138

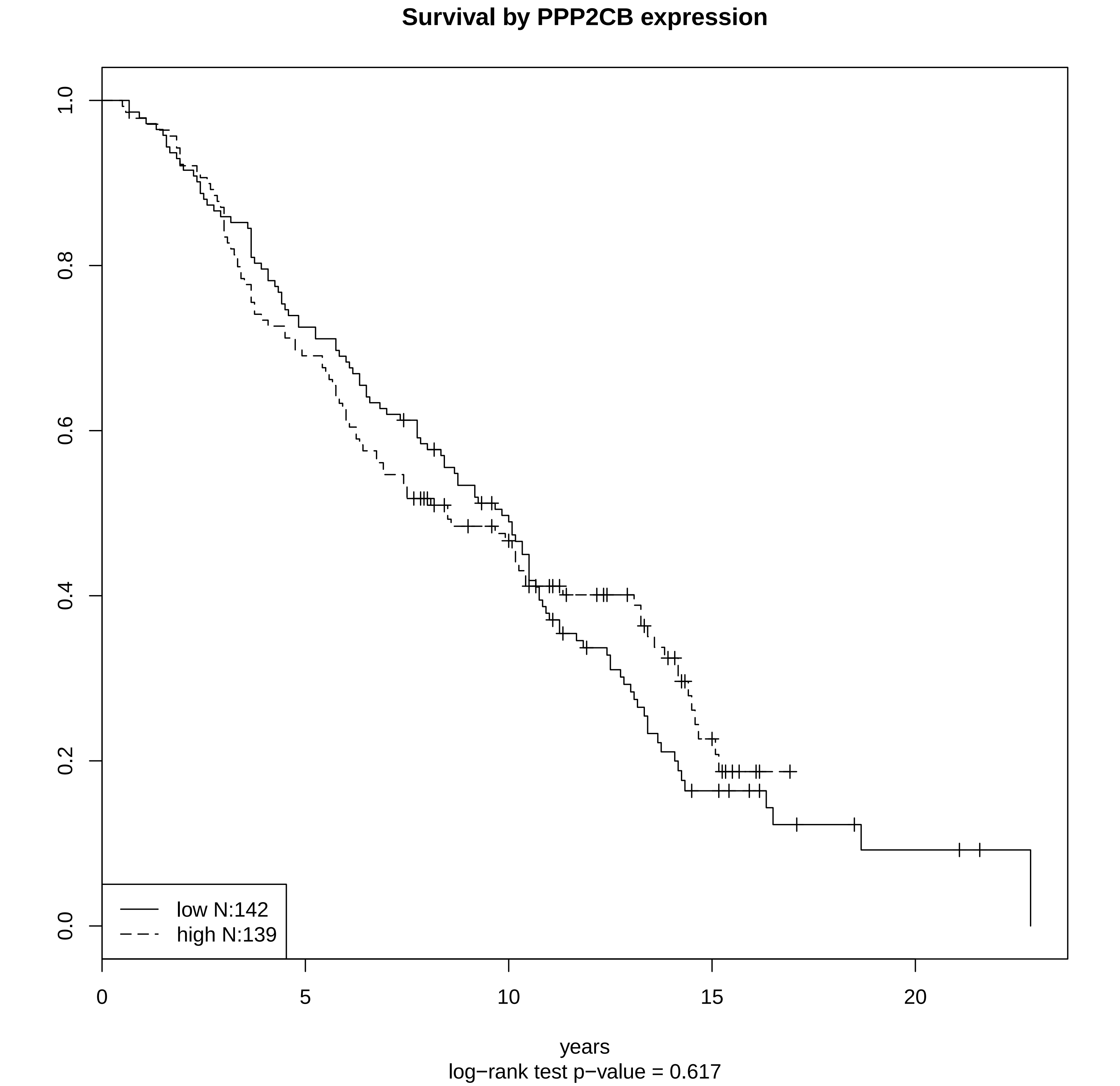

Survival by PPP2CB expression
1.0
0.8
0.6
0.4
0.2
0.0
0
5
10
15
20
low N:142
high N:139
years
log−rank test p−value = 0.617

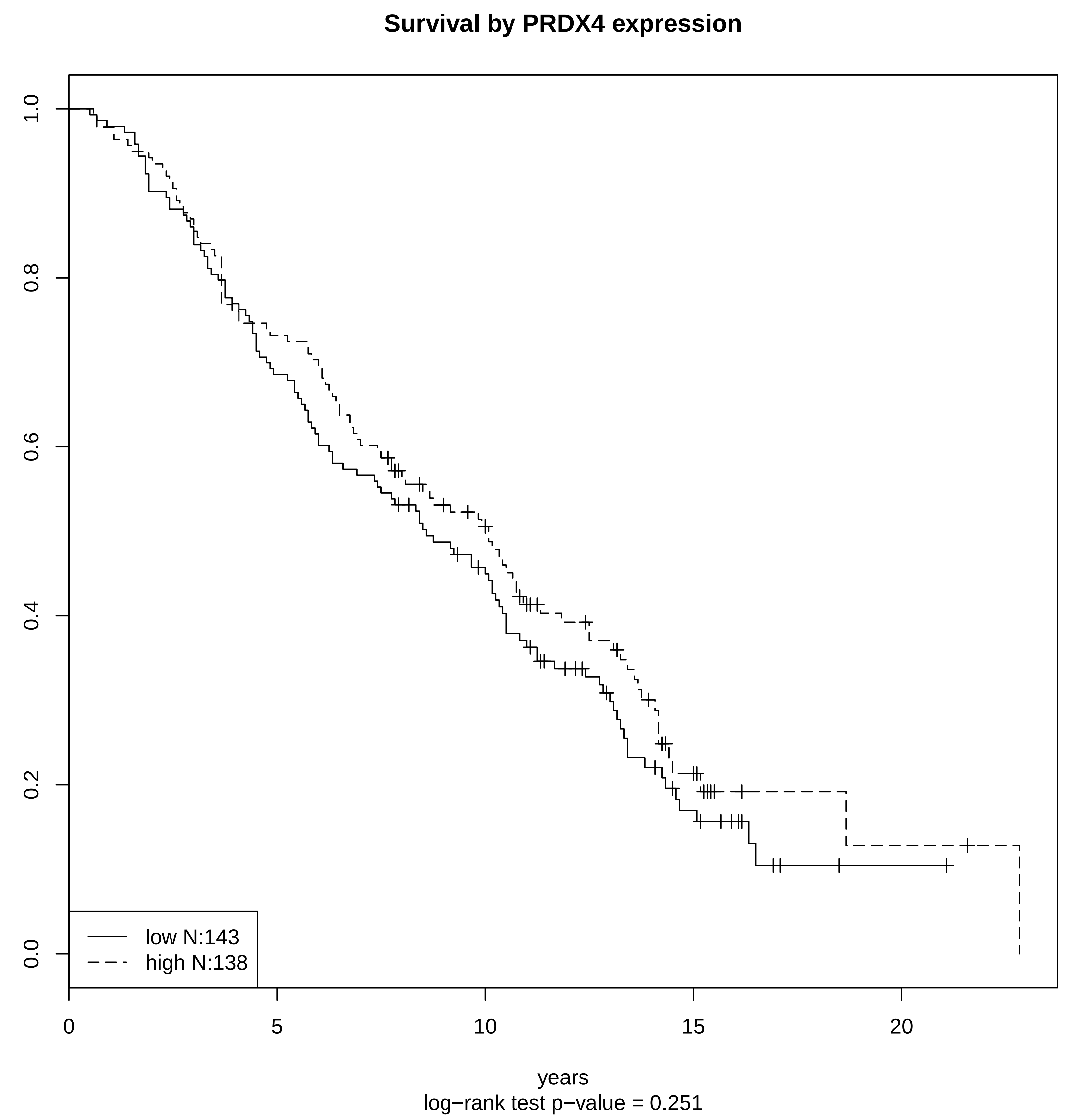

Survival by PRDX4 expression
1.0
0.8
0.6
0.4
0.2
0.0
0
5
10
15
20
years
log−rank test p−value = 0.251
low N:143
high N:138

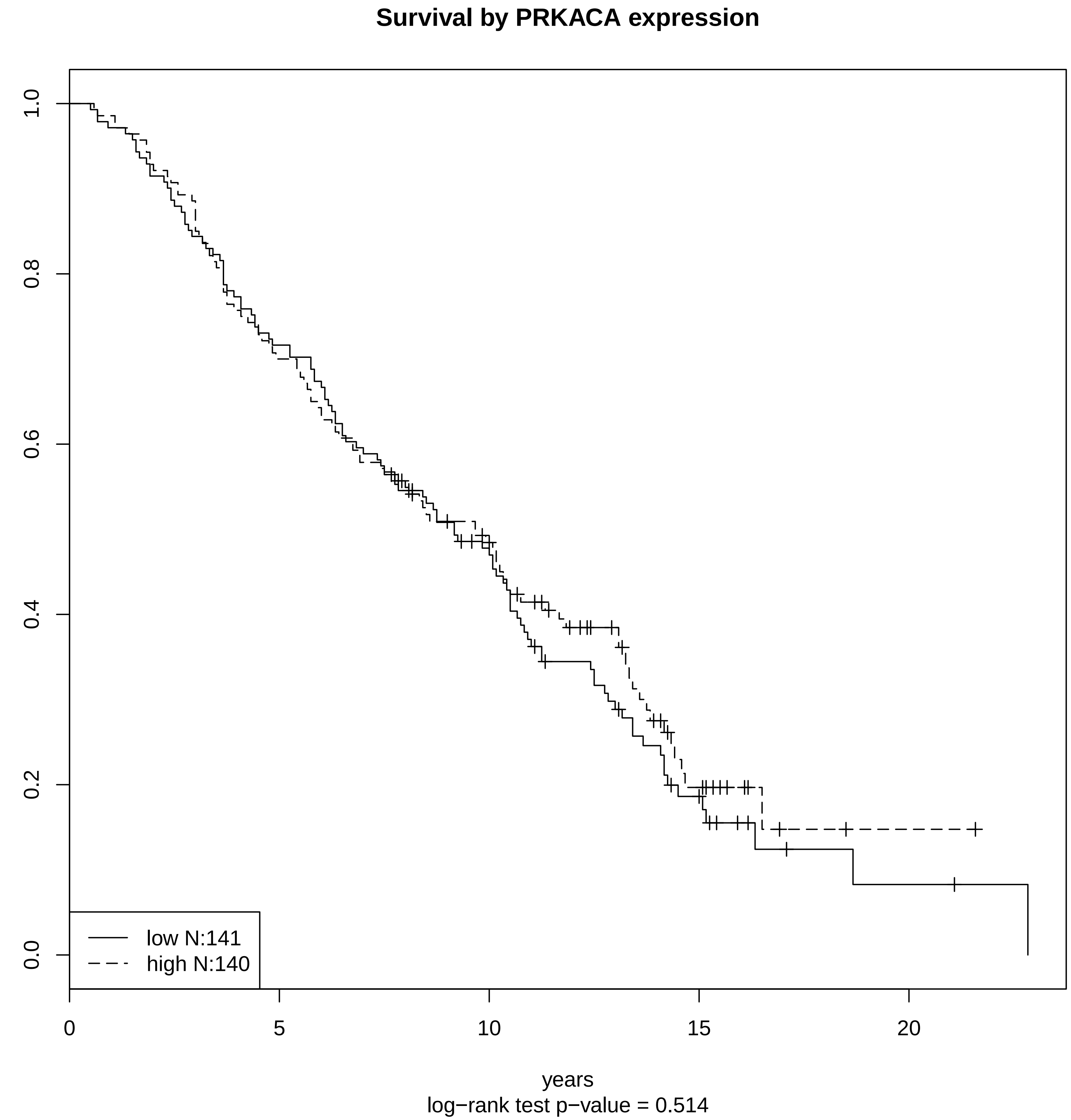

Survival by PRKACA expression
1.0
0.8
0.6
0.4
0.2
0.0
0
5
10
15
20
years
log−rank test p−value = 0.514
low N:141
high N:140

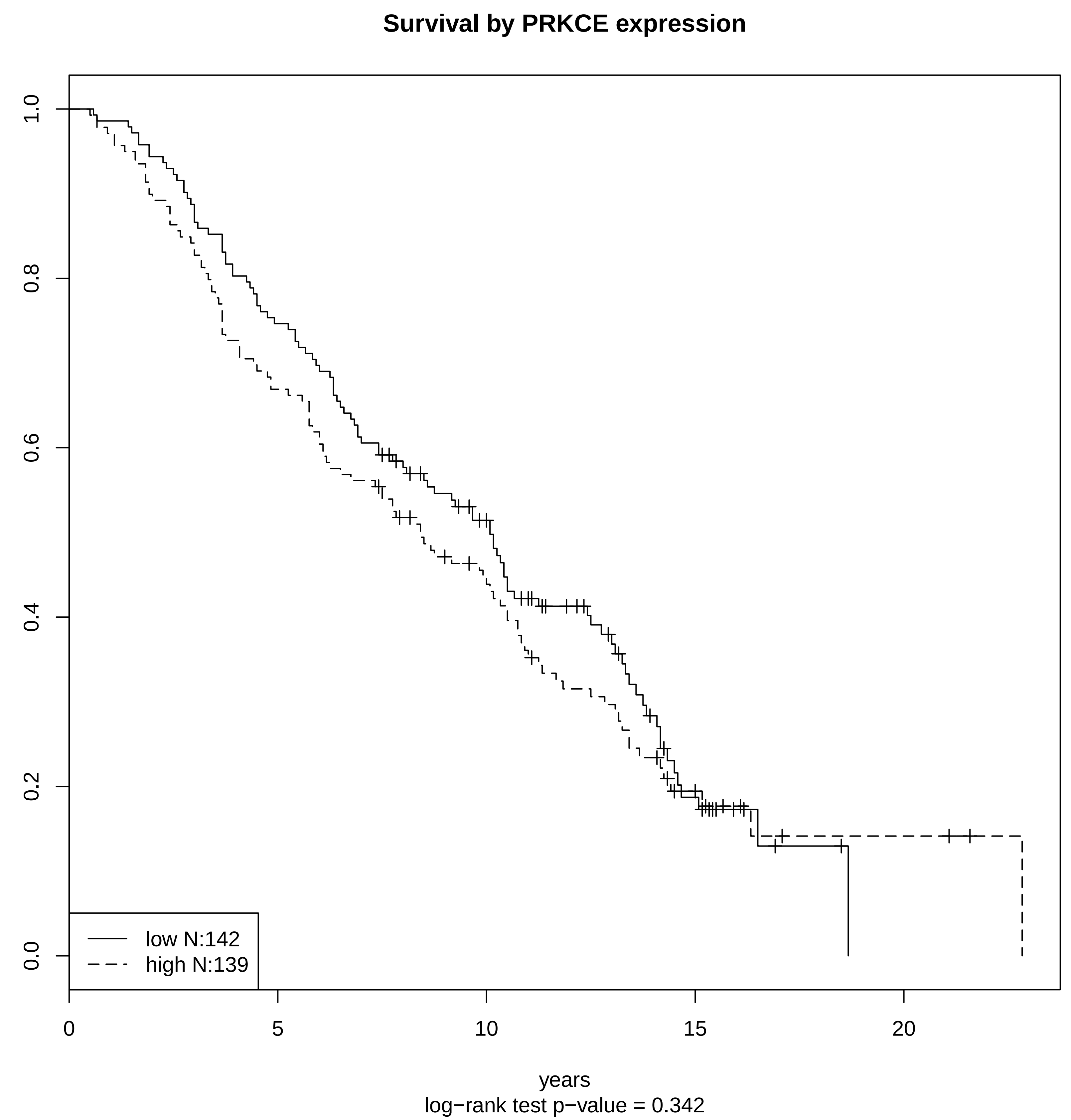
Survival by PRKCE expression
1.0
0.8
0.6
0.4
0.2
0.0
0
5
10
15
20
low N:142
high N:139
years
log−rank test p−value = 0.342

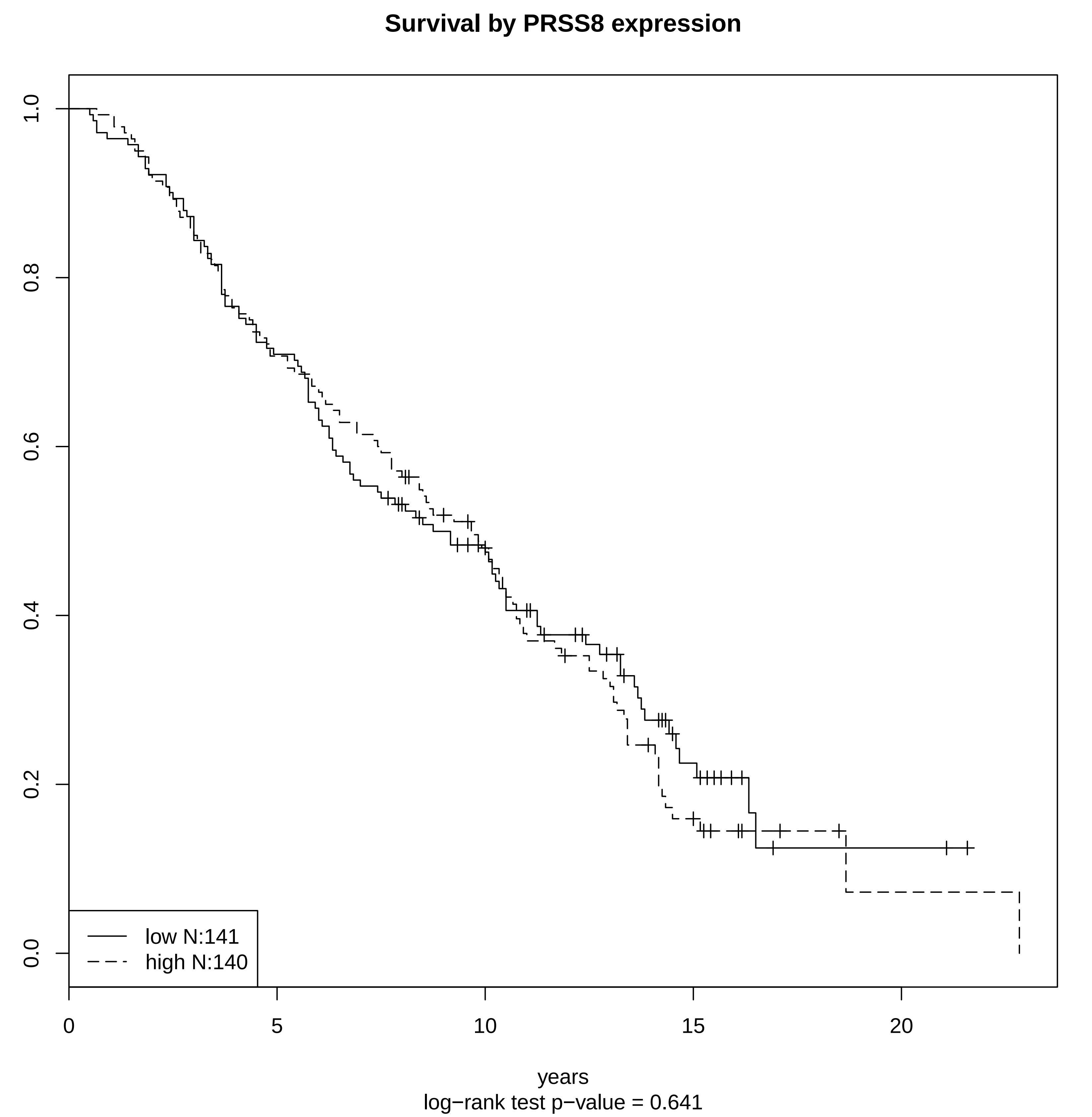

Survival by PRSS8 expression
1.0
0.8
0.6
0.4
0.2
0.0
0
5
10
15
20
years
log−rank test p−value = 0.641
low N:141
high N:140

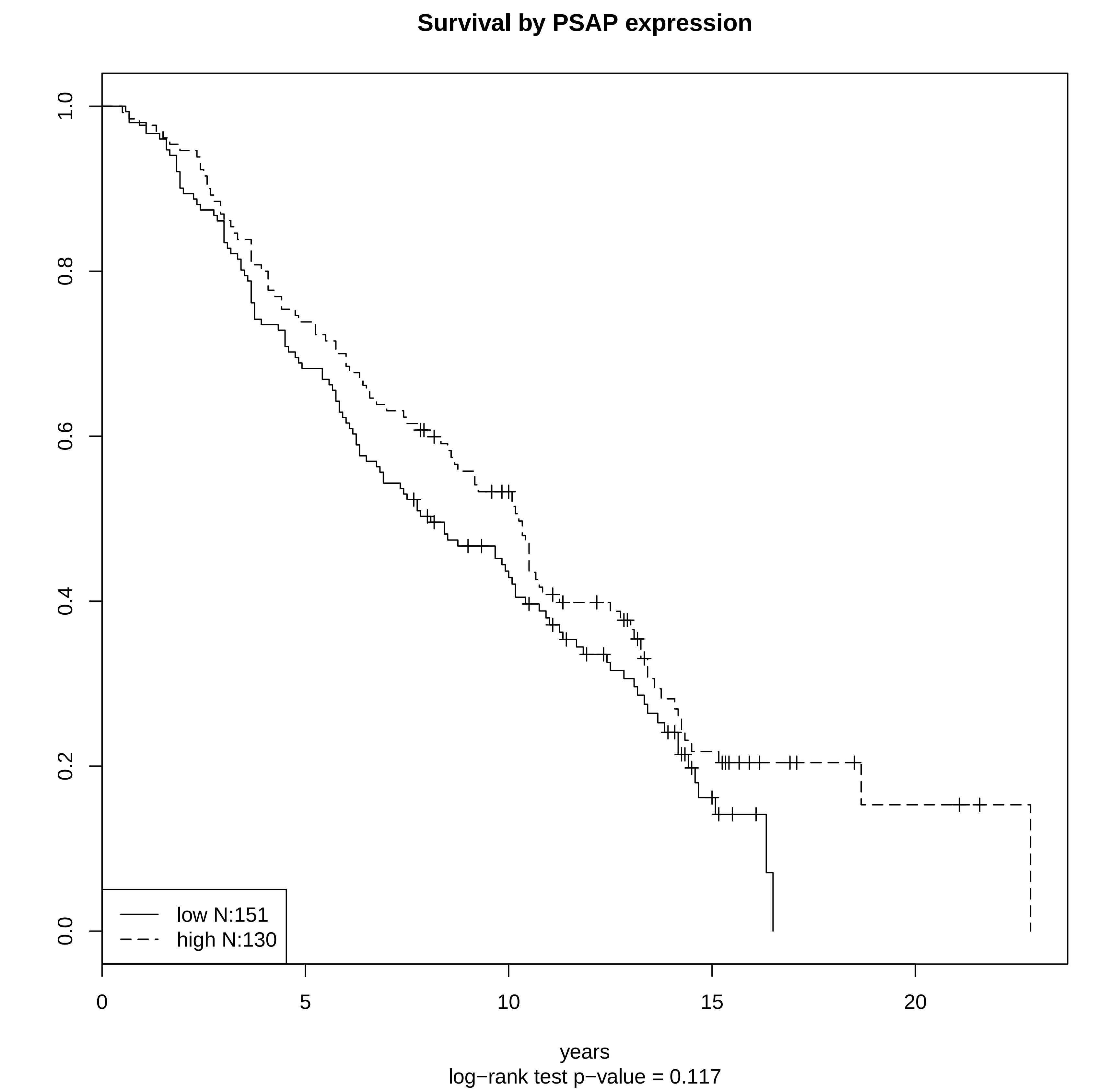
Survival by PSAP expression
1.0
0.8
0.6
0.4
0.2
0.0
0
5
10
15
20
low N:151
high N:130
years
log−rank test p−value = 0.117

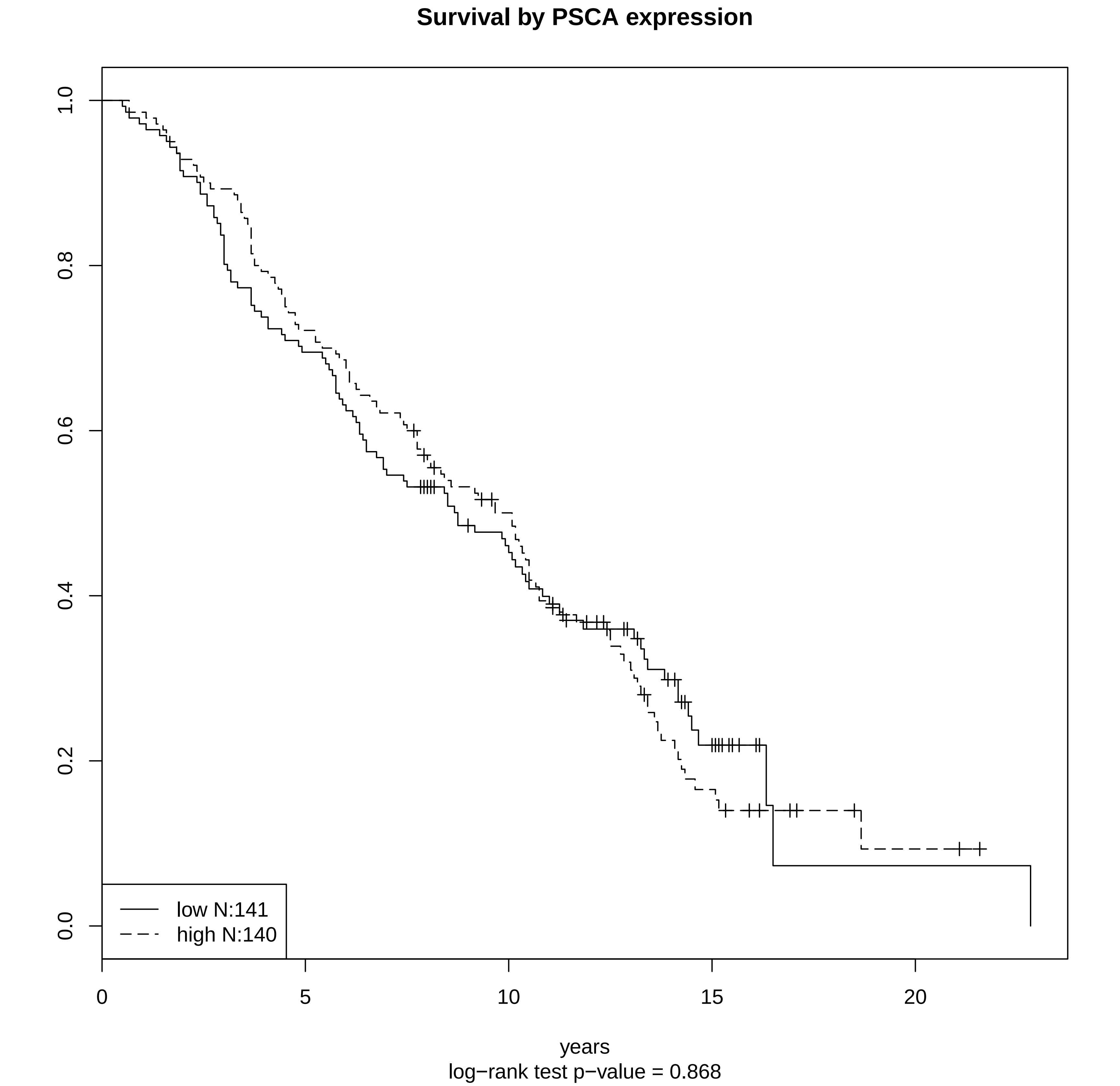

Survival by PSCA expression
1.0
0.8
0.6
0.4
0.2
0.0
0
5
10
15
20
low N:141
high N:140
years
log−rank test p−value = 0.868

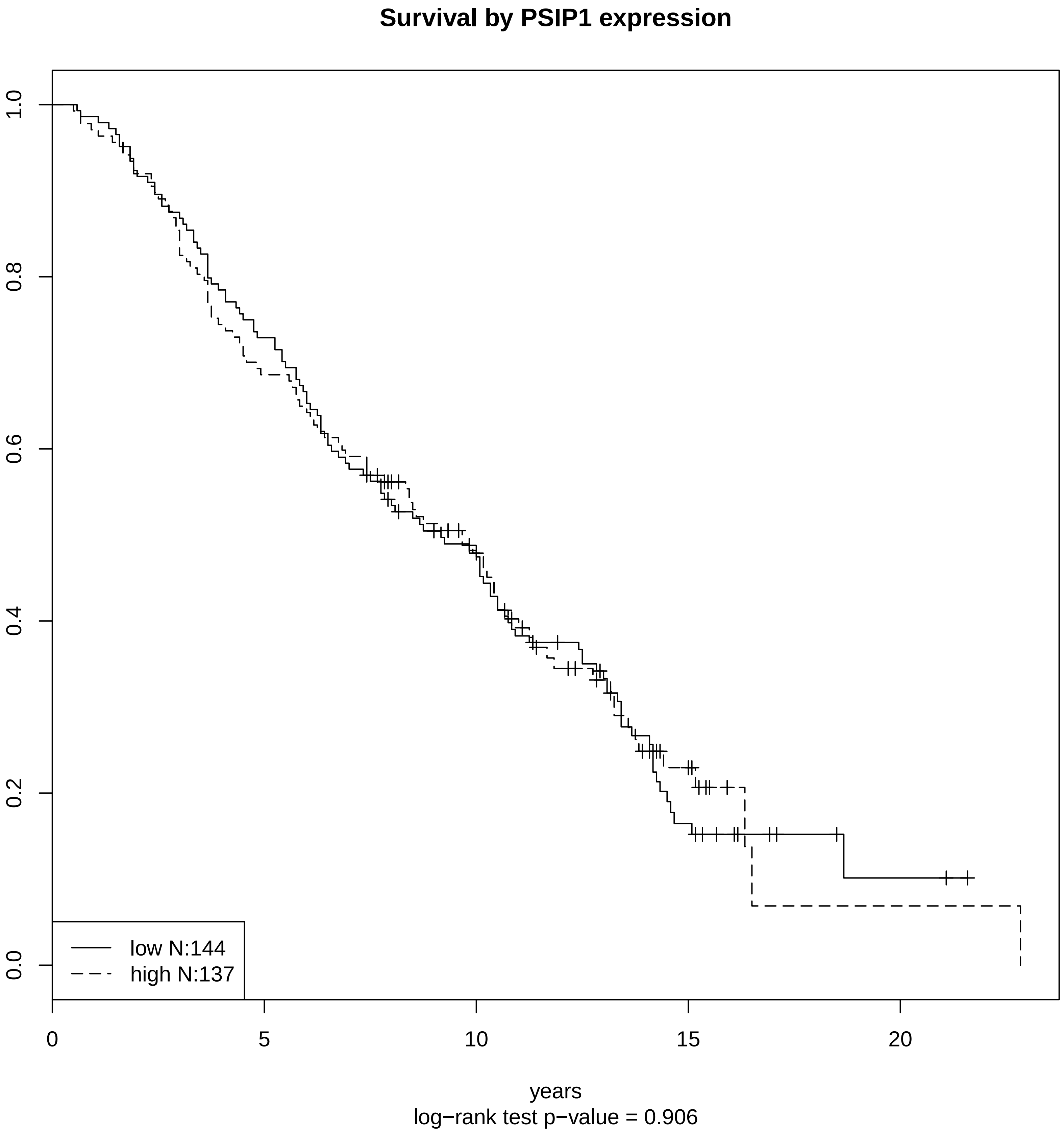
Survival by PSIP1 expression
1.0
0.8
0.6
0.4
0.2
0.0
0
5
10
15
20
low N:144
high N:137
years
log−rank test p−value = 0.906

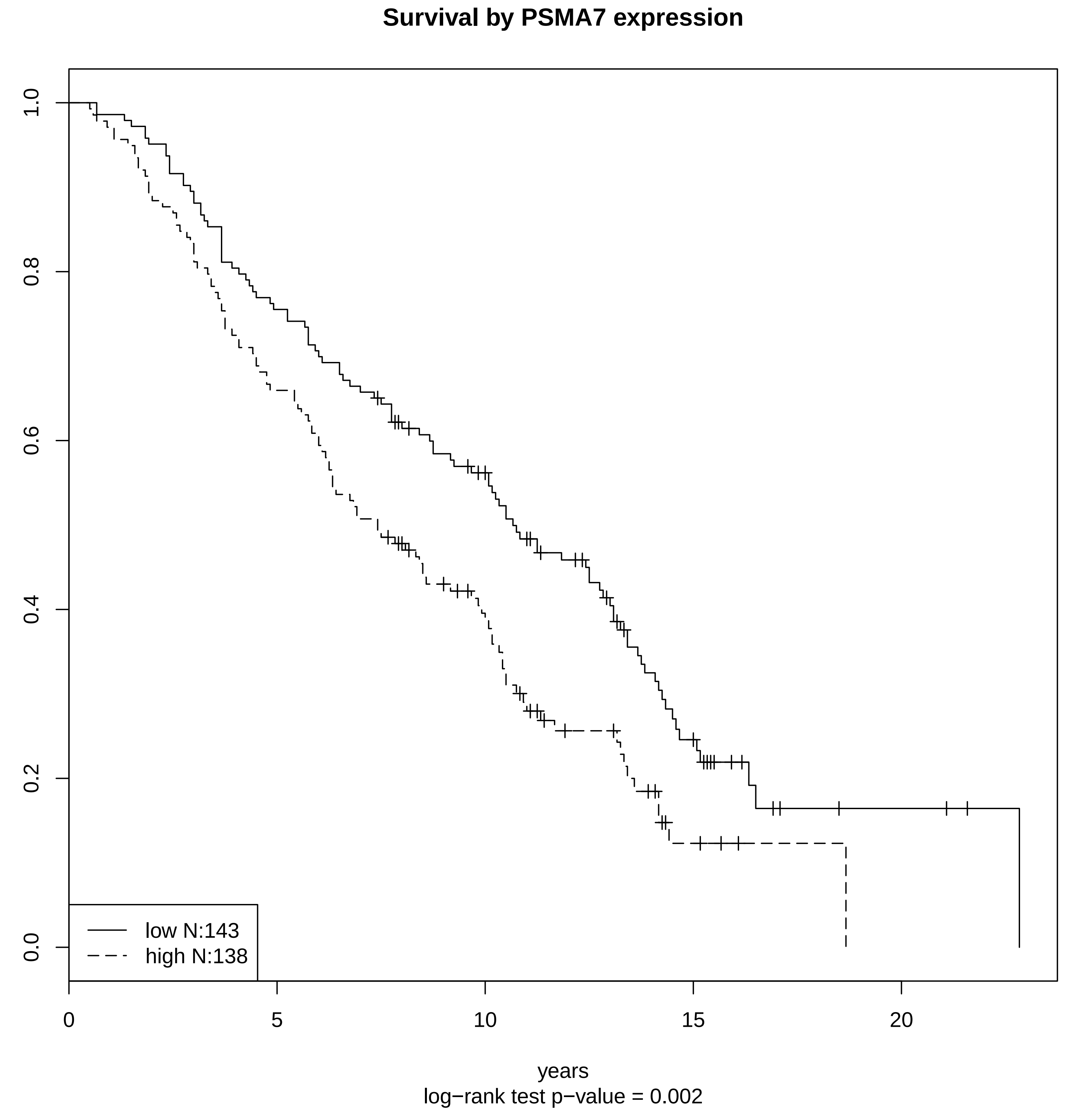

Survival by PSMA7 expression
1.0
0.8
0.6
0.4
0.2
0.0
0
5
10
15
20
low N:143
high N:138
years
log−rank test p−value = 0.002

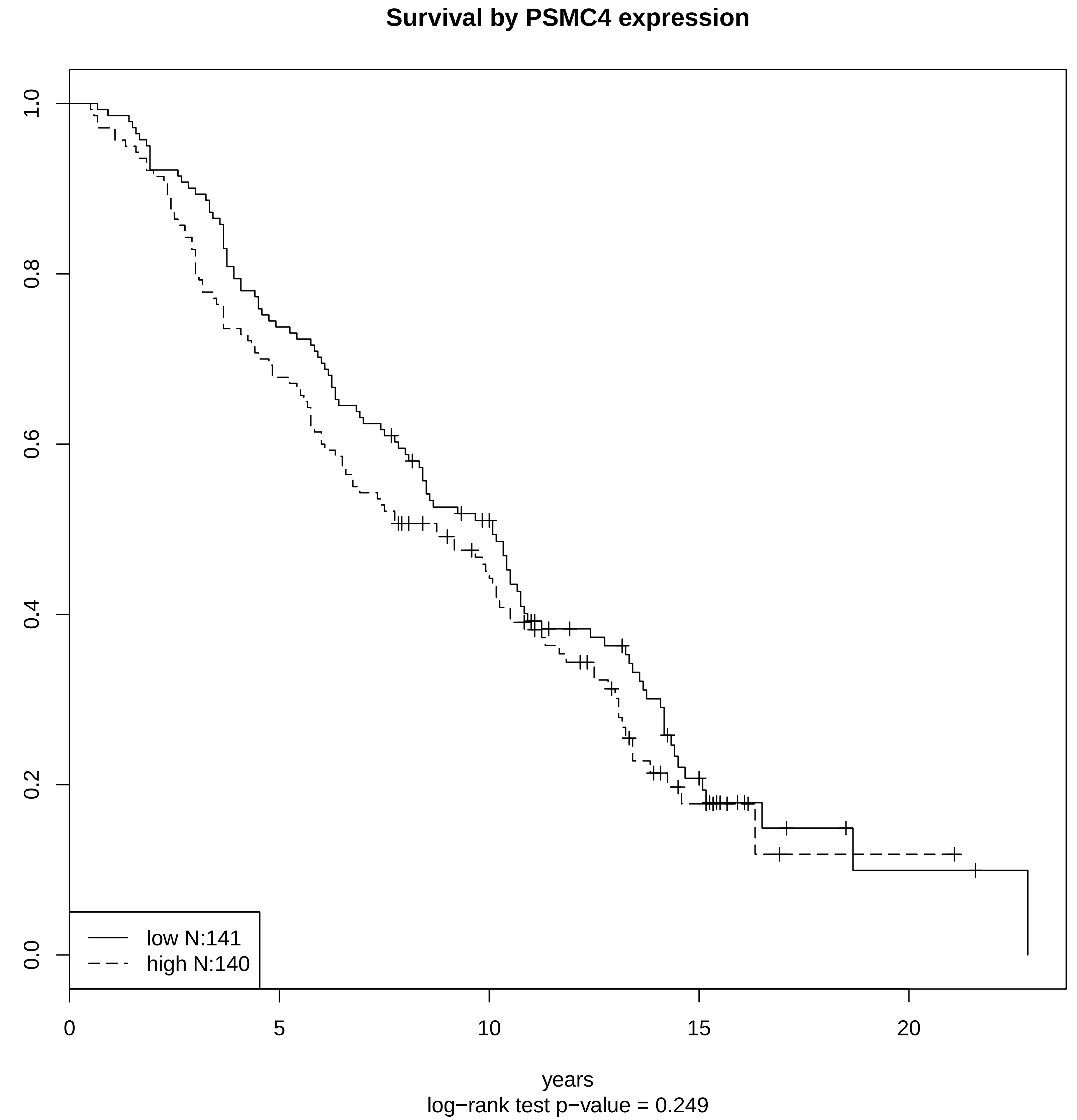
Survival by PSMC4 expression
1.0
0.8
0.6
0.4
0.2
0.0
0
5
10
15
20
years
log−rank test p−value = 0.249
low N:141
high N:140

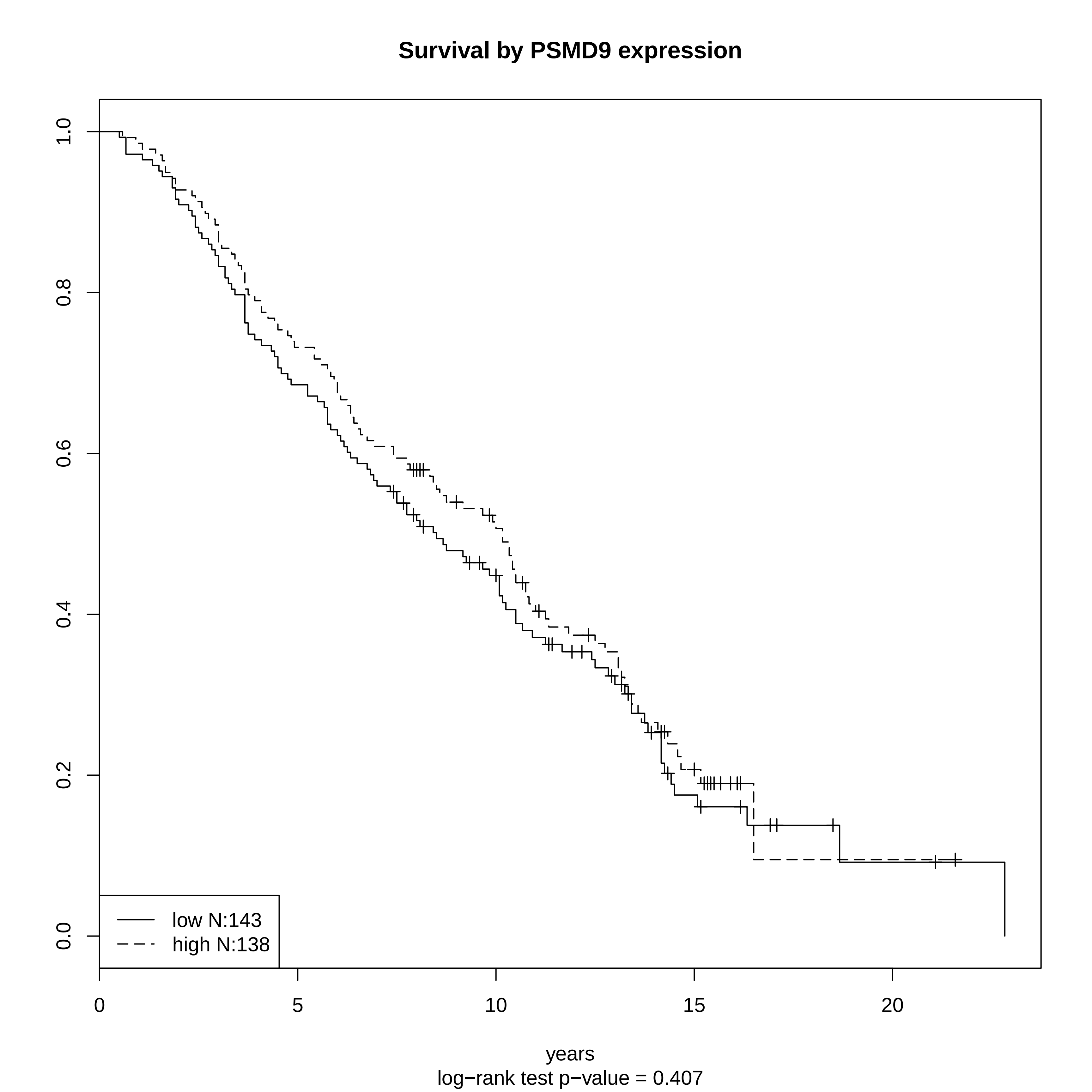
Survival by PSMD9 expression
1.0
0.8
0.6
0.4
0.2
0.0
0
5
10
15
20
low N:143
high N:138
years
log−rank test p−value = 0.407

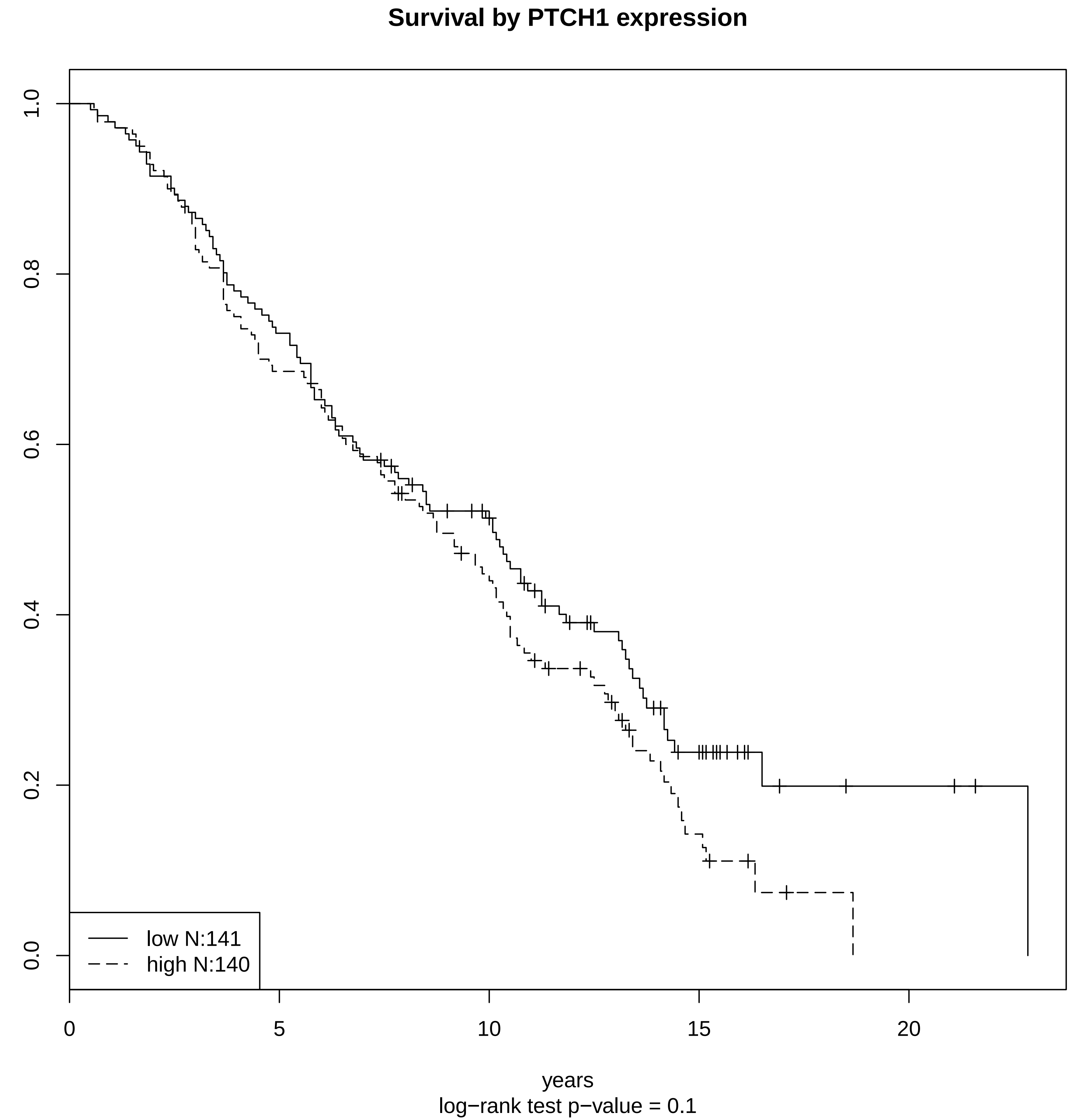

Survival by PTCH1 expression
1.0
0.8
0.6
0.4
0.2
0.0
0
5
10
15
20
years
log−rank test p−value = 0.1
low N:141
high N:140

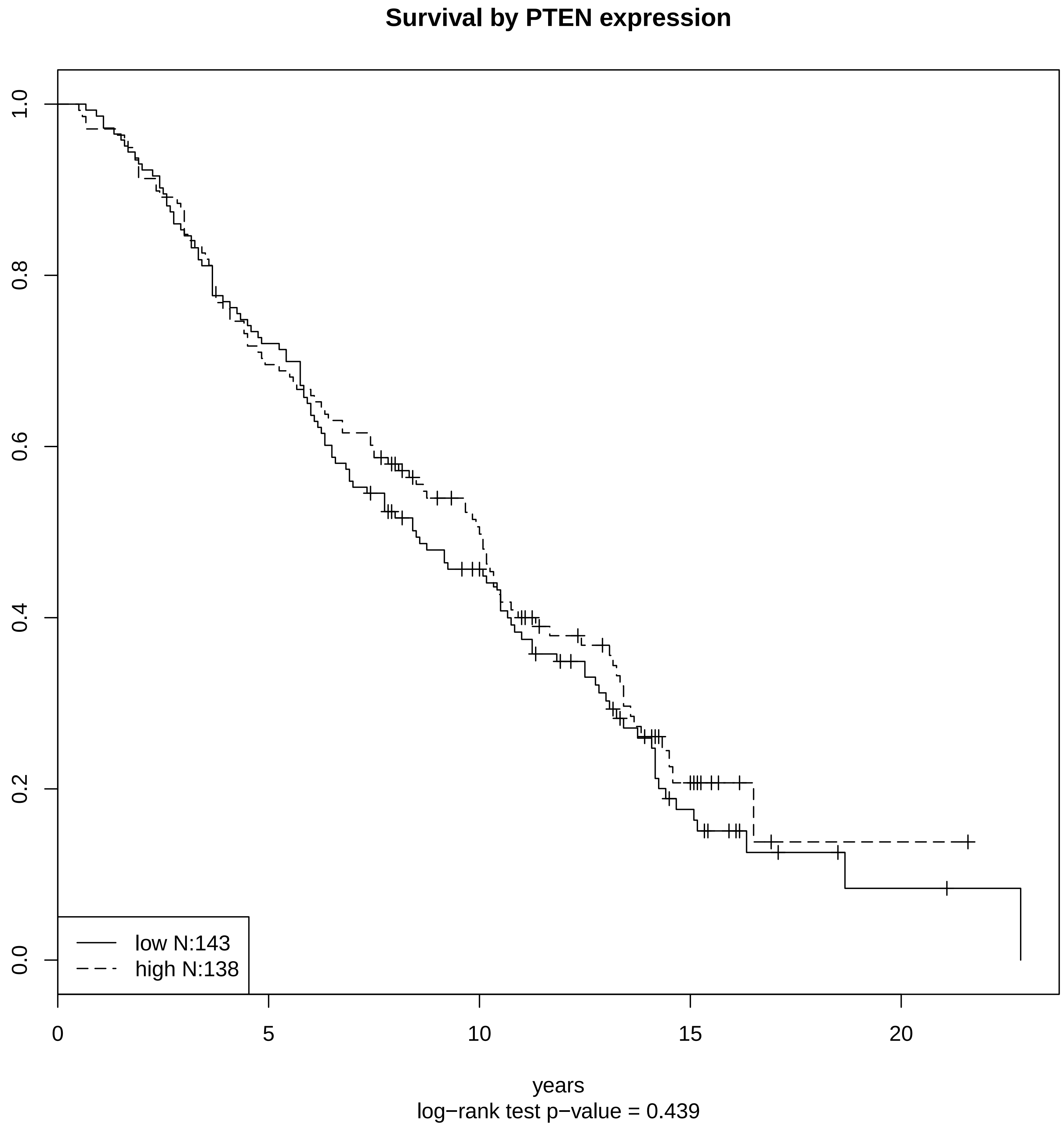
Survival by PTEN expression
1.0
0.8
0.6
0.4
0.2
0.0
0
5
10
15
20
years
low N:143
high N:138
log−rank test p−value = 0.439

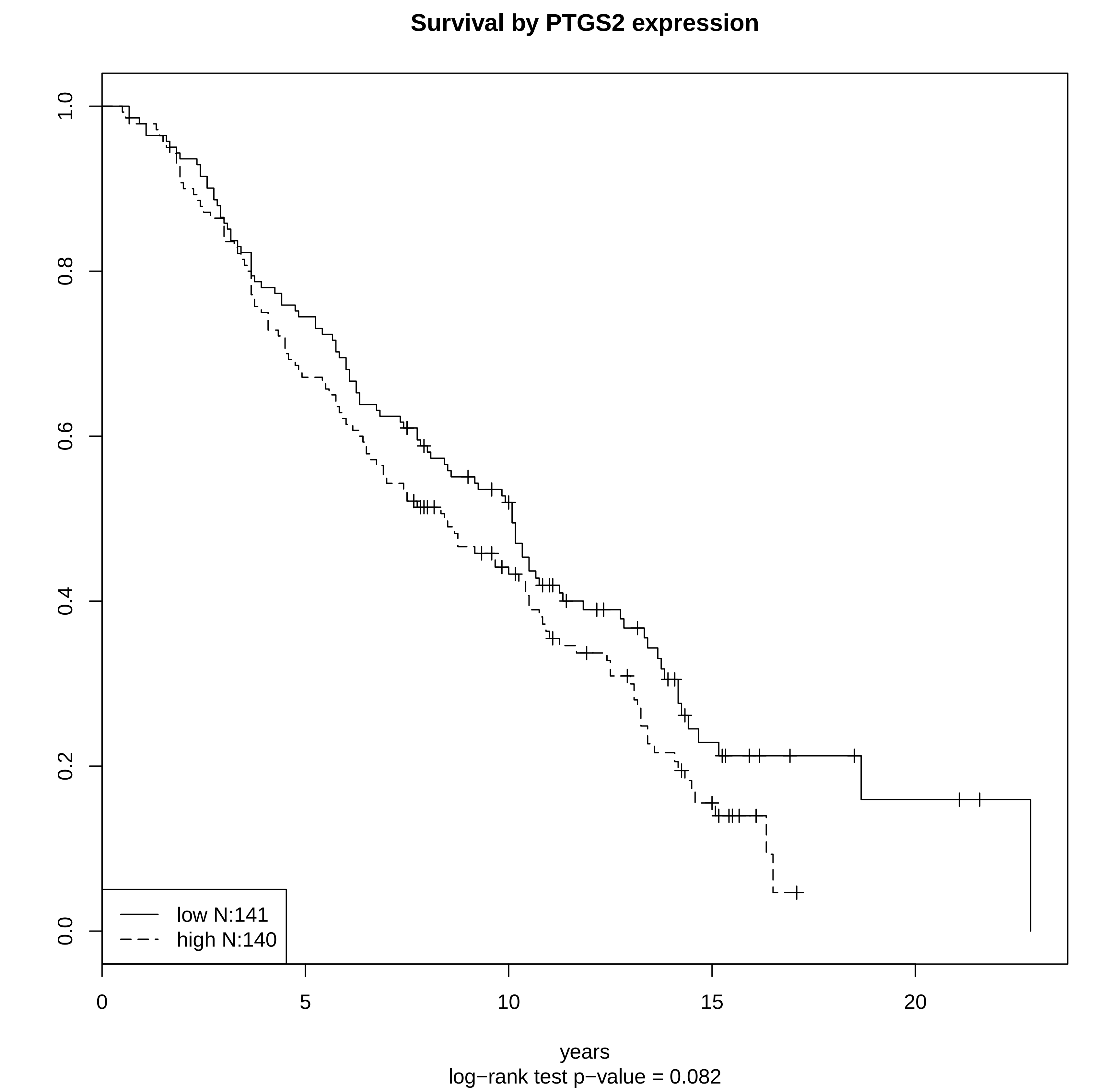

Survival by PTGS2 expression
1.0
0.8
0.6
0.4
0.2
0.0
0
5
10
15
20
low N:141
high N:140
years
log−rank test p−value = 0.082

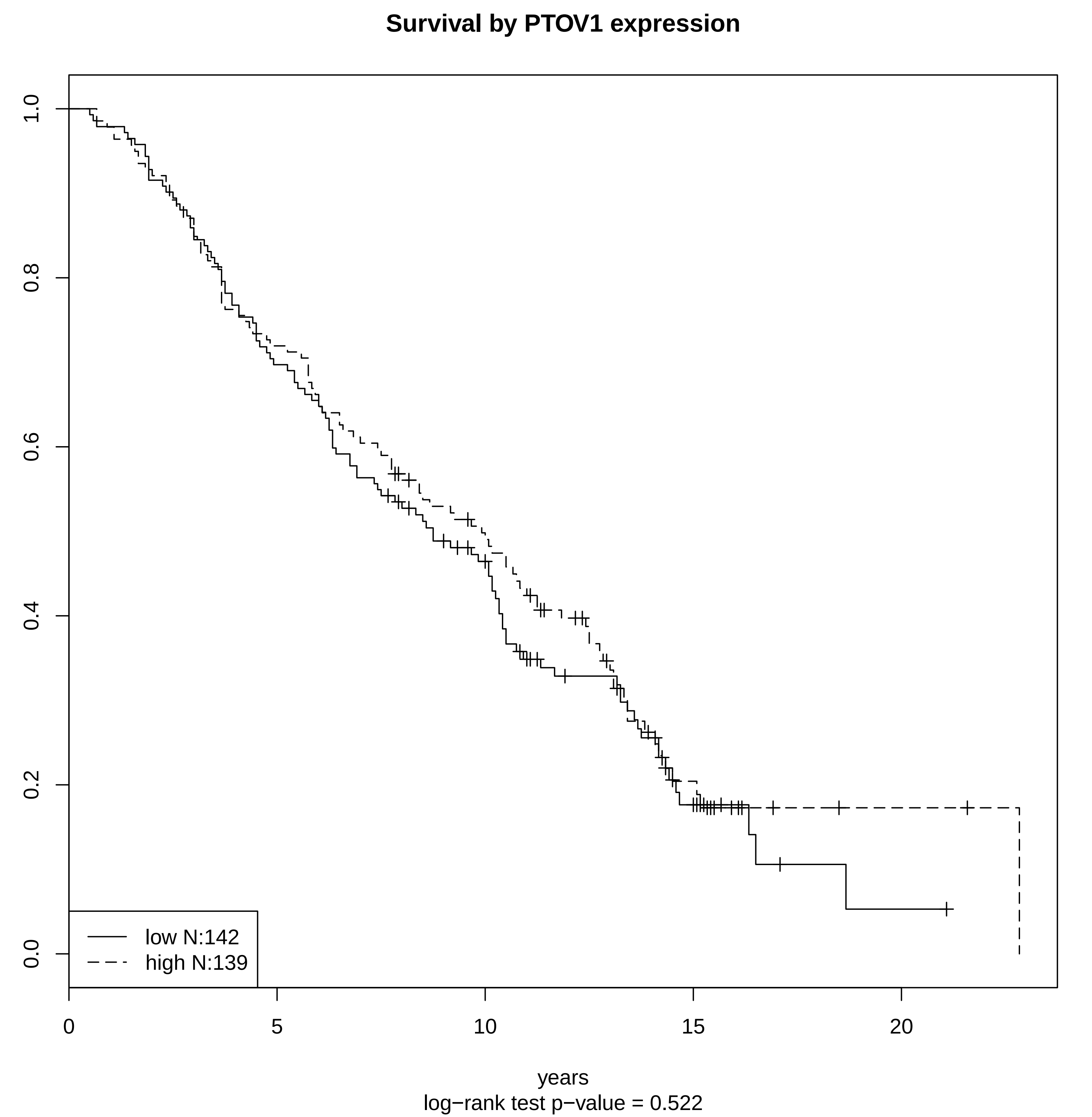

Survival by PTOV1 expression
1.0
0.8
0.6
0.4
0.2
0.0
0
5
10
15
20
years
low N:142
high N:139
log−rank test p−value = 0.522

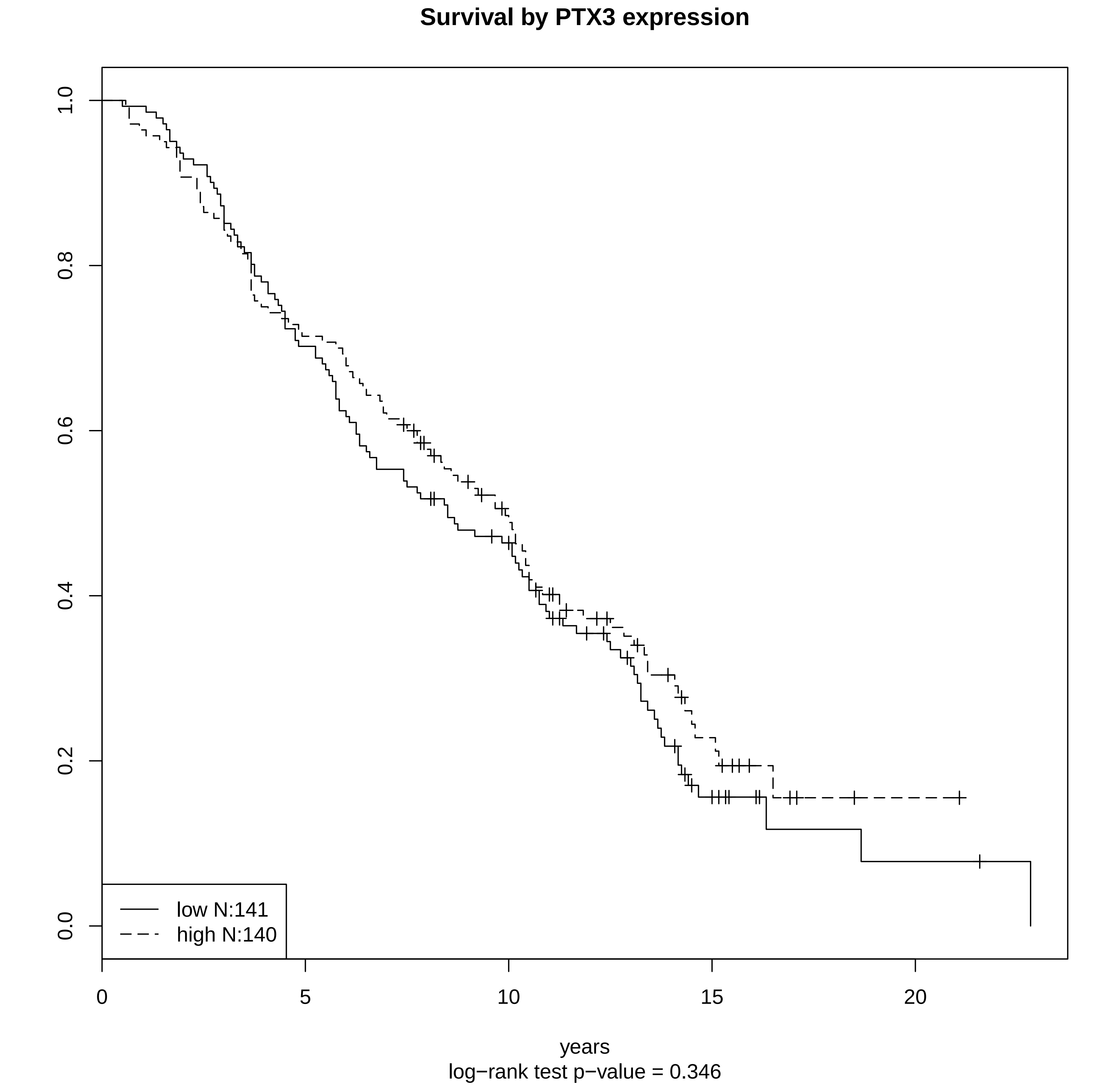
Survival by PTX3 expression
1.0
0.8
0.6
0.4
0.2
0.0
0
5
10
15
20
years
log−rank test p−value = 0.346
low N:141
high N:140

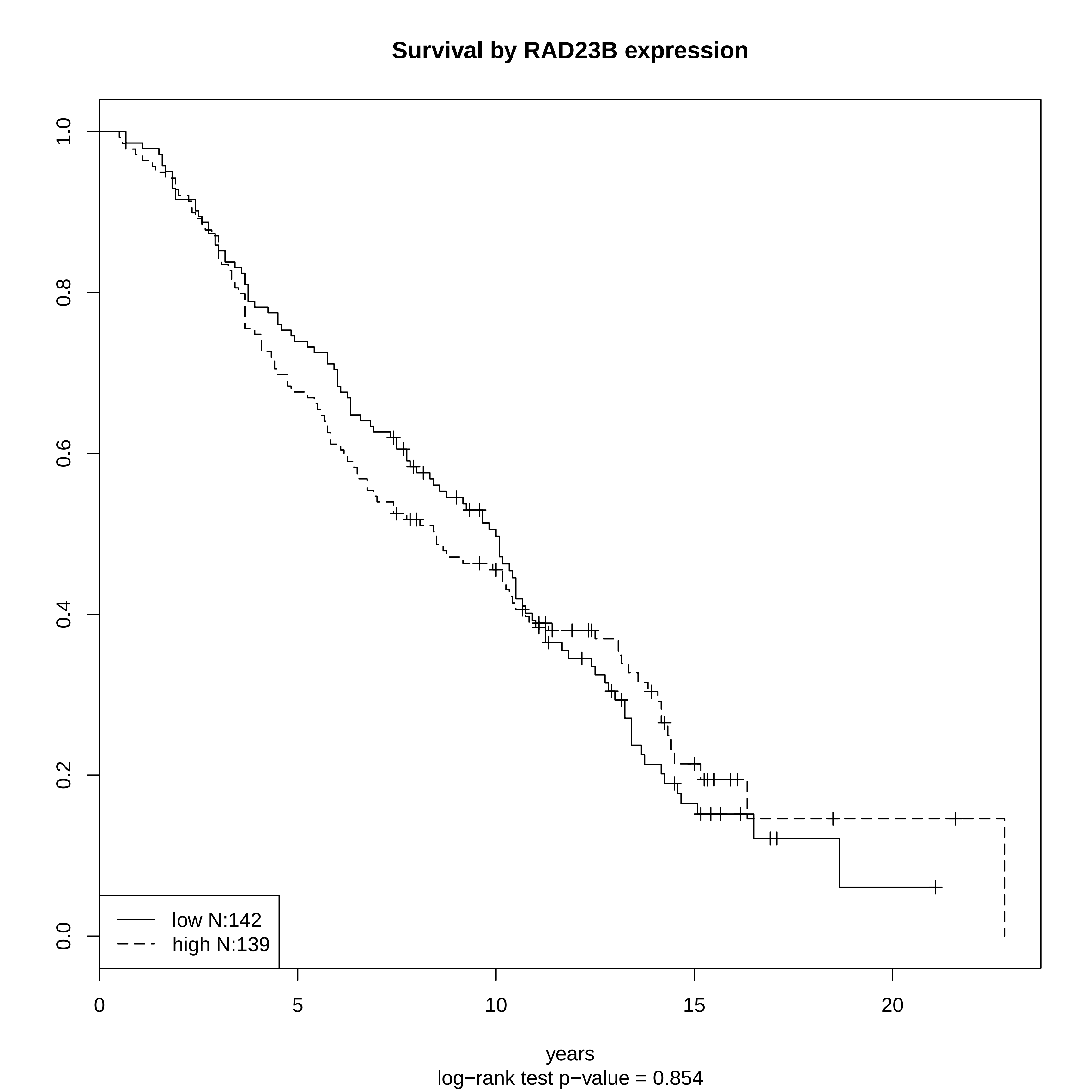
Survival by RAD23B expression
1.0
0.8
0.6
0.4
0.2
0.0
0
5
10
15
20
low N:142
high N:139
years
log−rank test p−value = 0.854

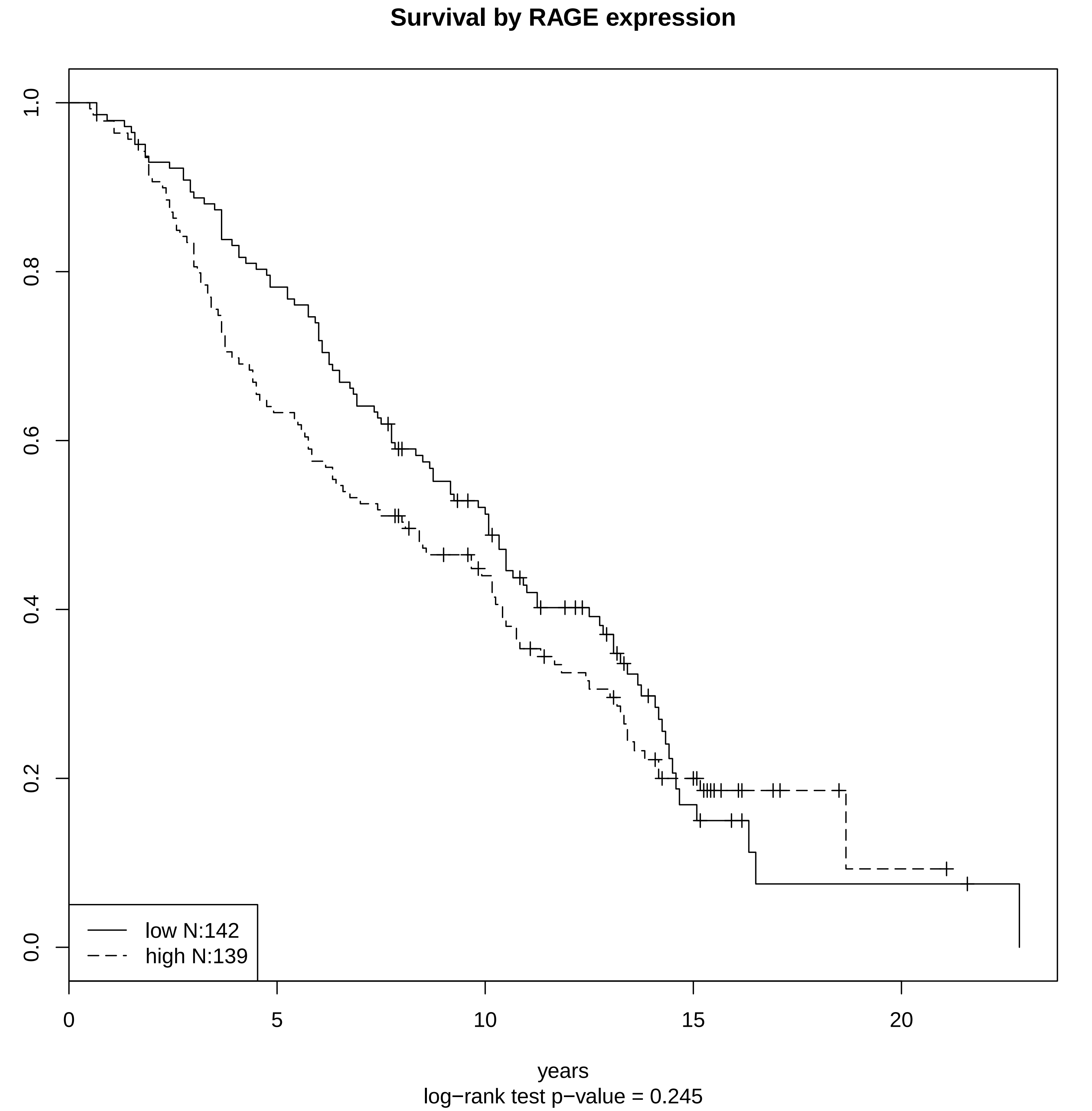
Survival by RAGE expression
1.0
0.8
0.6
0.4
0.2
0.0
0
5
10
15
20
low N:142
high N:139
years
log−rank test p−value = 0.245

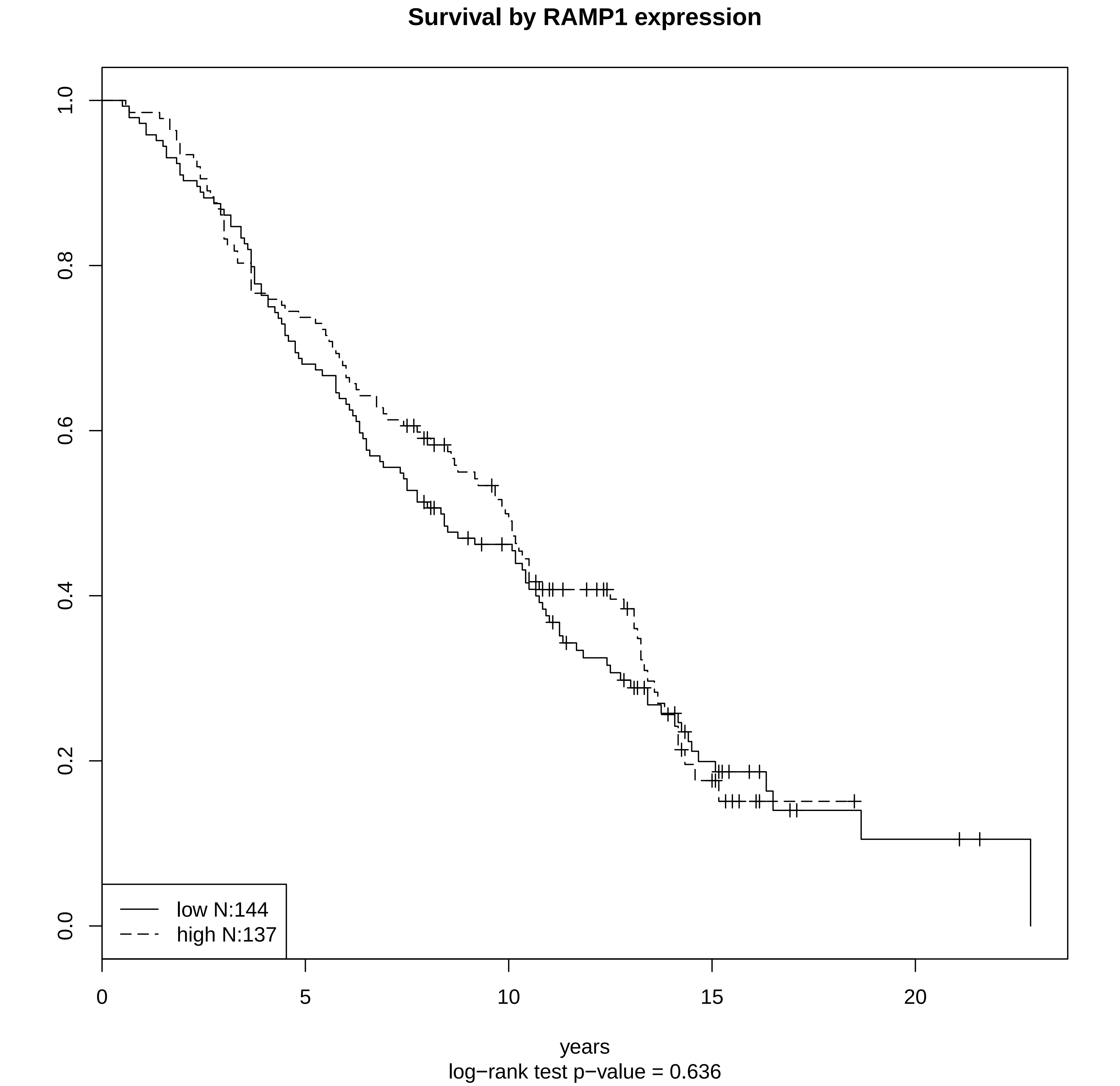
Survival by RAMP1 expression
0.0
0.2
0.4
0.6
0.8
1.0
0
5
10
15
20
years
low N:144
high N:137
log−rank test p−value = 0.636

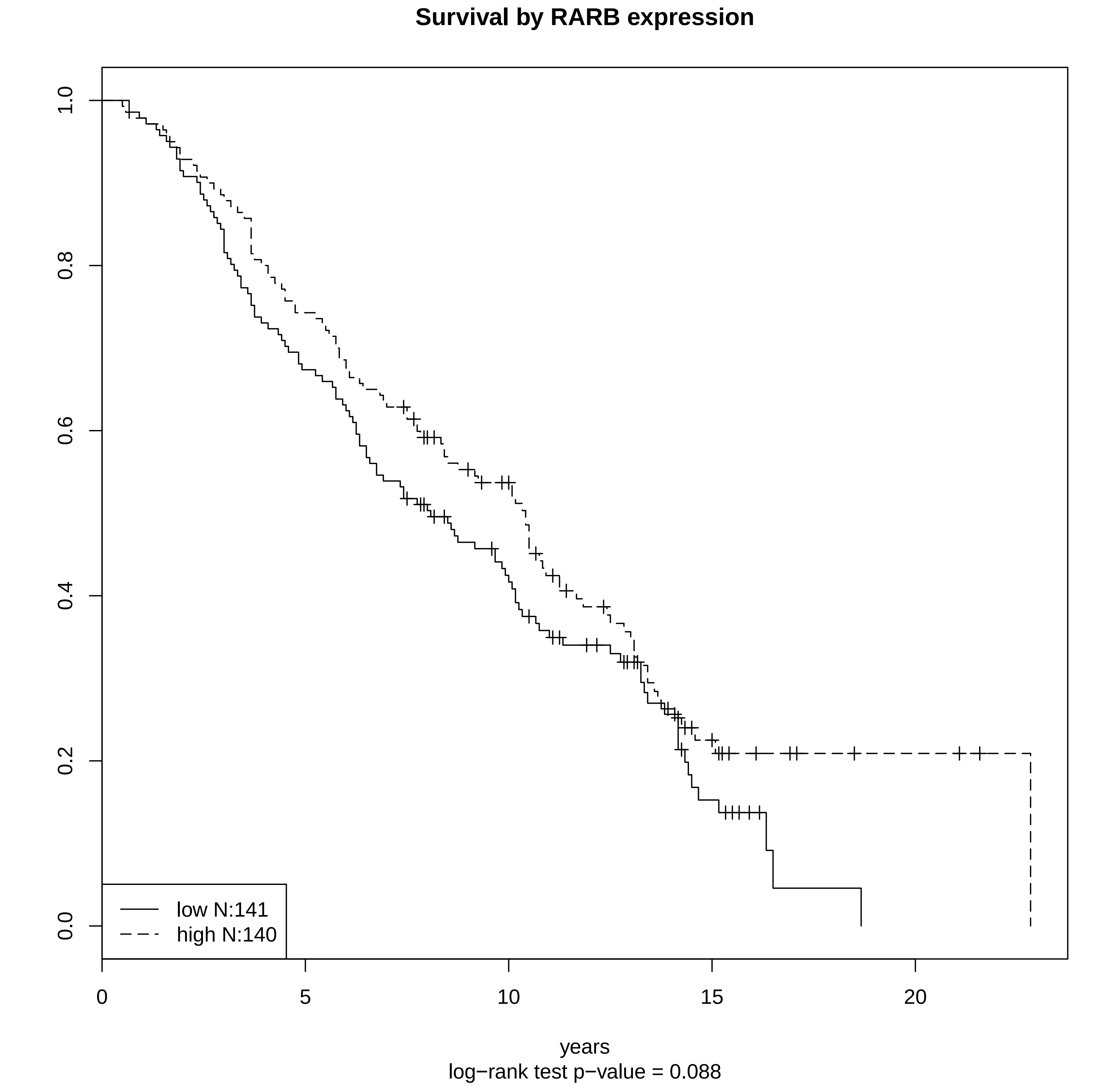
Survival by RARB expression
0.0
0.2
0.4
0.6
0.8
1.0
0
5
10
15
20
low N:141
high N:140
years
log−rank test p−value = 0.088

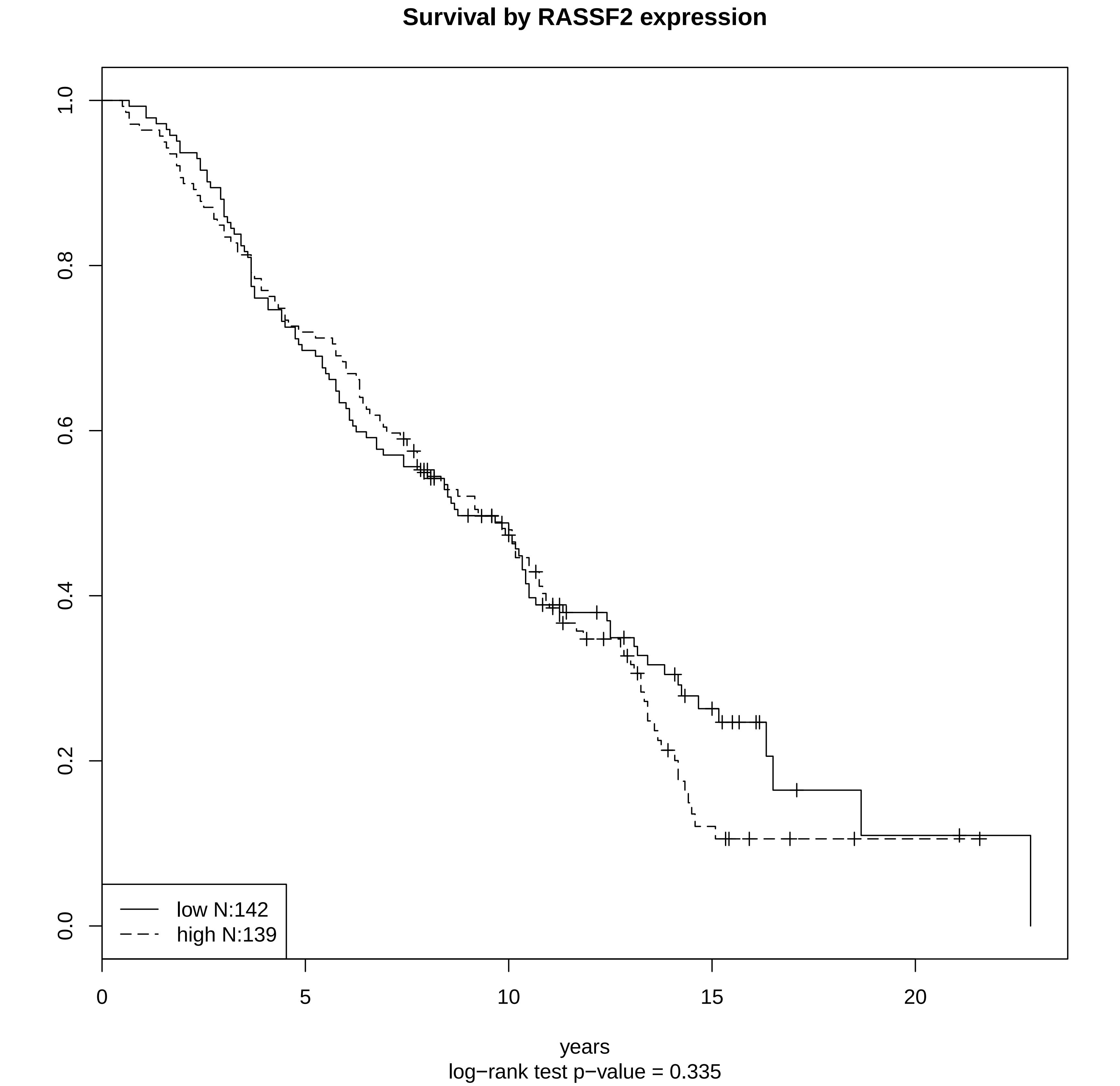

Survival by RASSF2 expression
1.0
0.8
0.6
0.4
0.2
0.0
0
5
10
15
20
low N:142
high N:139
years
log−rank test p−value = 0.335

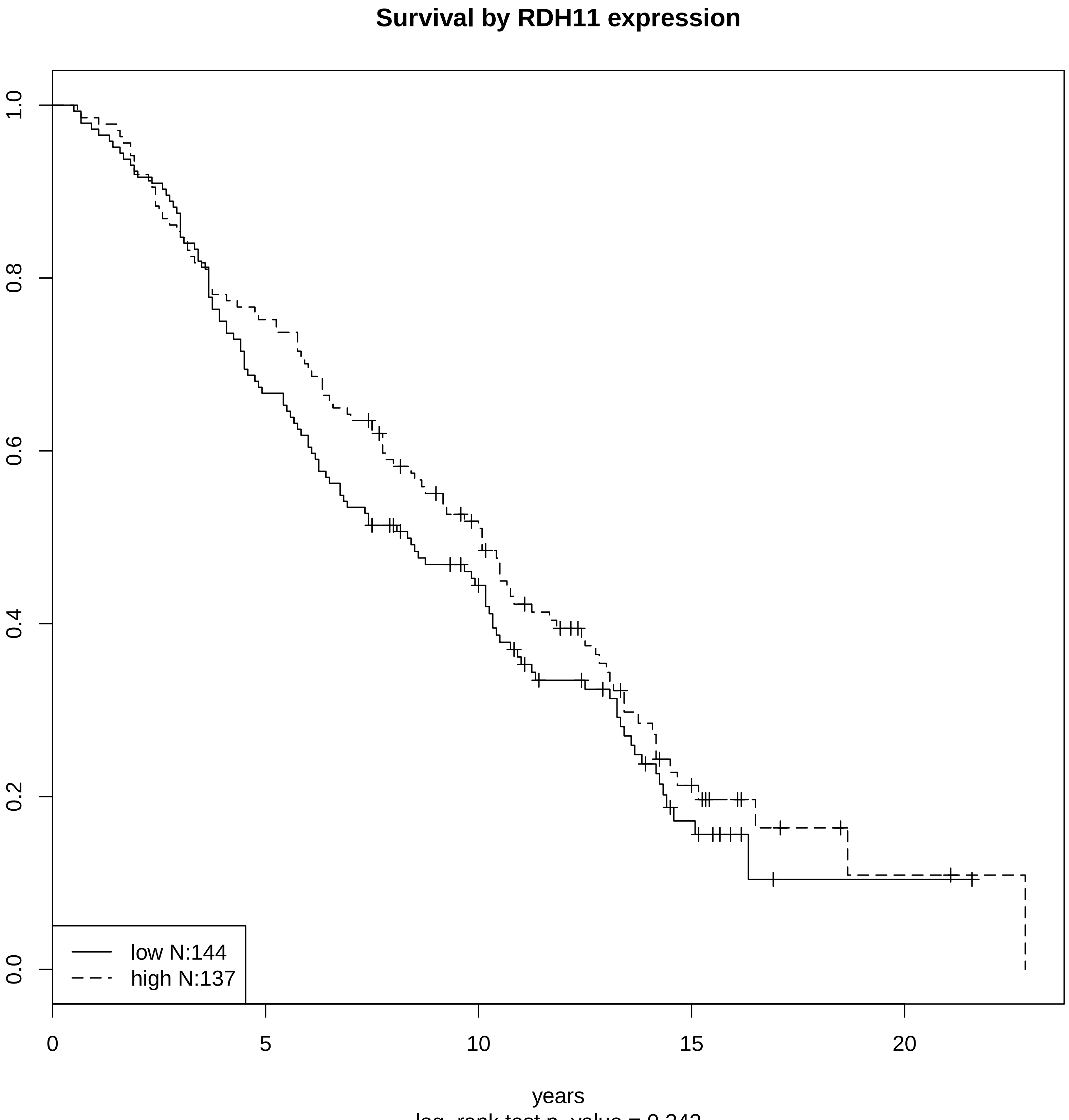
Survival by RDH11 expression
1.0
0.8
0.6
0.4
0.2
0.0
0
5
10
15
20
years
low N:144
high N:137
log−rank test p−value = 0.242

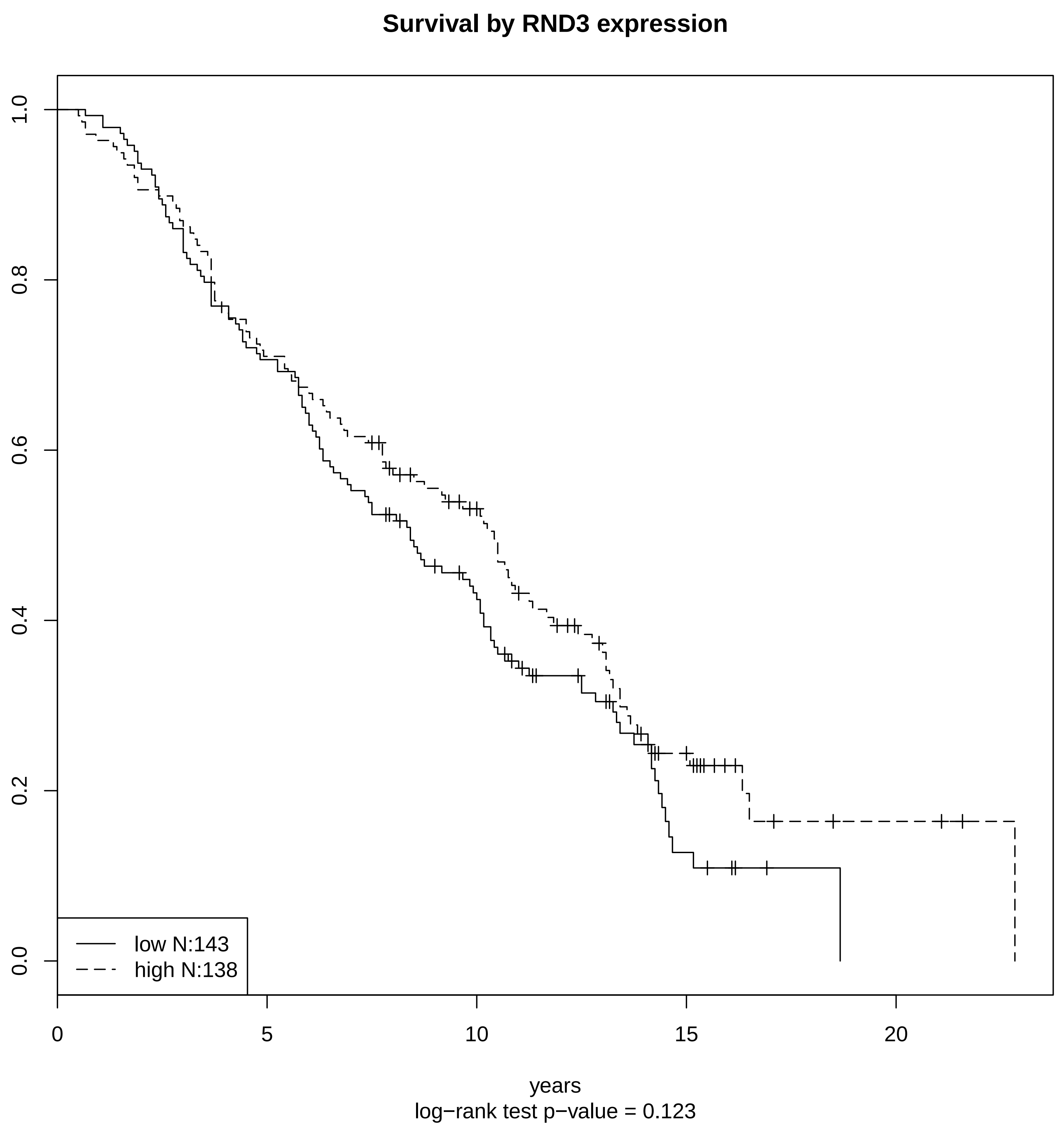

Survival by RND3 expression
1.0
0.8
0.6
0.4
0.2
0.0
0
5
10
15
20
years
log−rank test p−value = 0.123
low N:143
high N:138

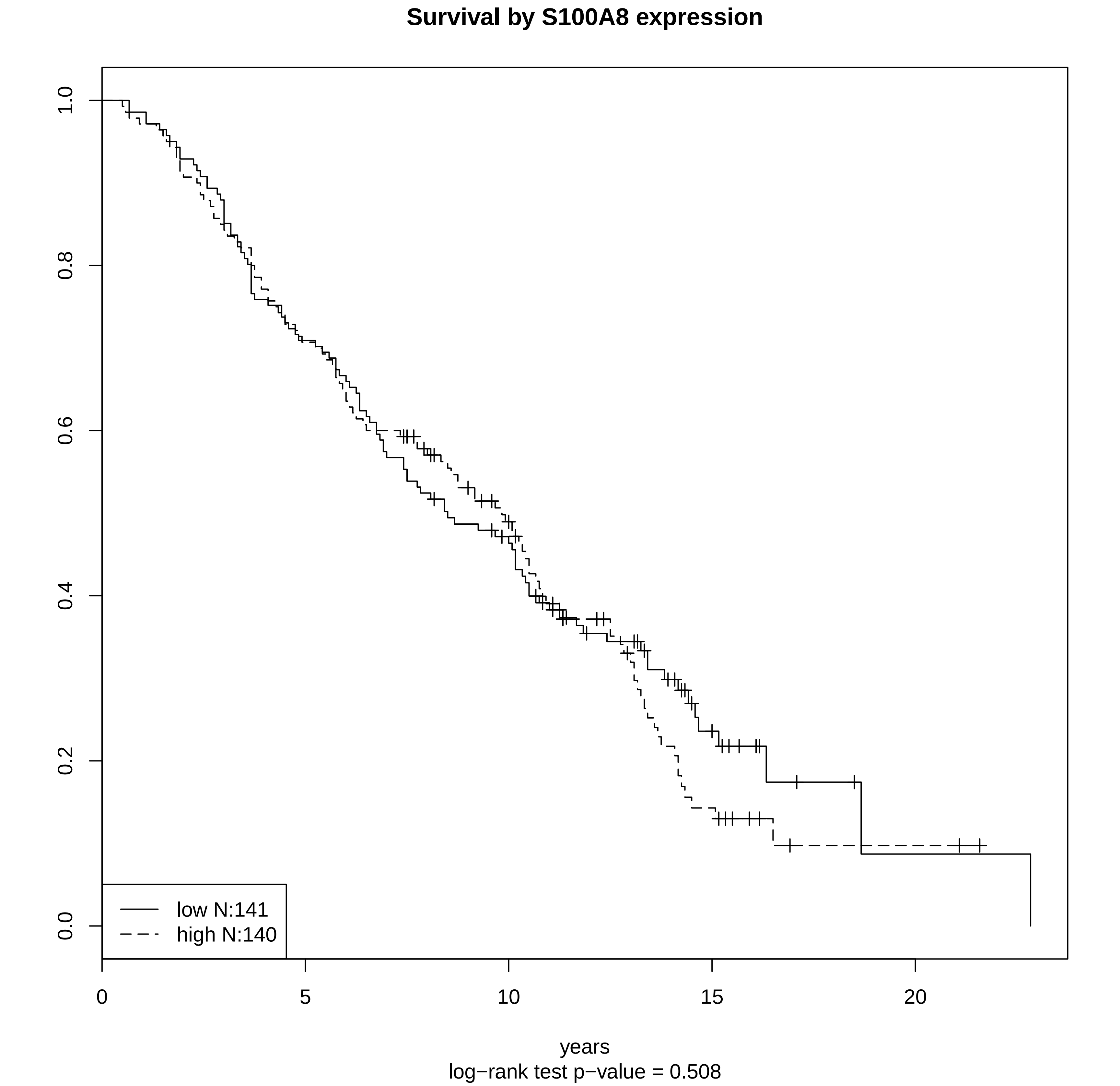

Survival by S100A8 expression
1.0
0.8
0.6
0.4
0.2
0.0
0
5
10
15
20
low N:141
high N:140
years
log−rank test p−value = 0.508

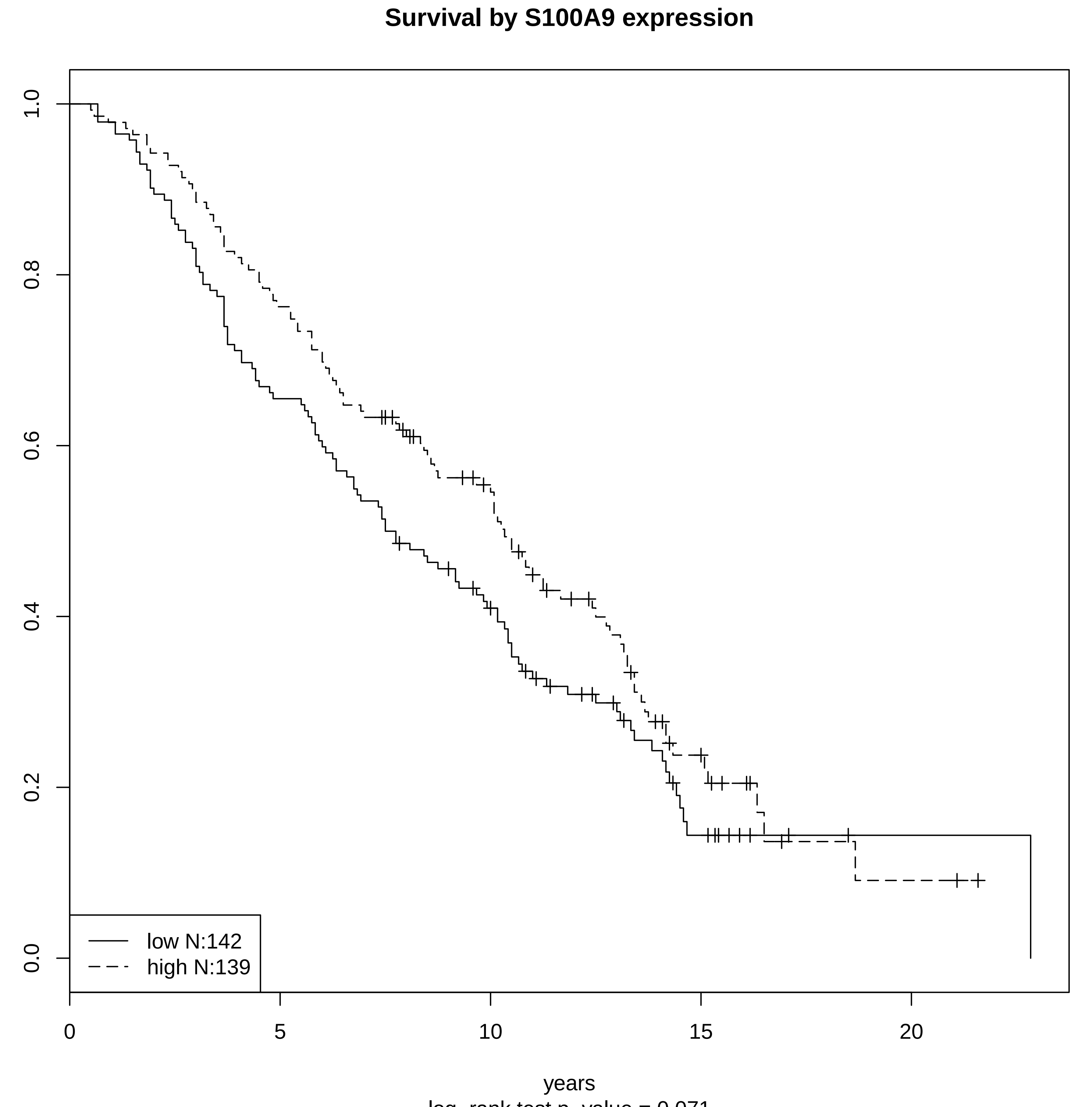

Survival by S100A9 expression
1.0
0.8
0.6
0.4
0.2
0.0
0
5
10
15
20
years
log−rank test p−value = 0.071
low N:142
high N:139

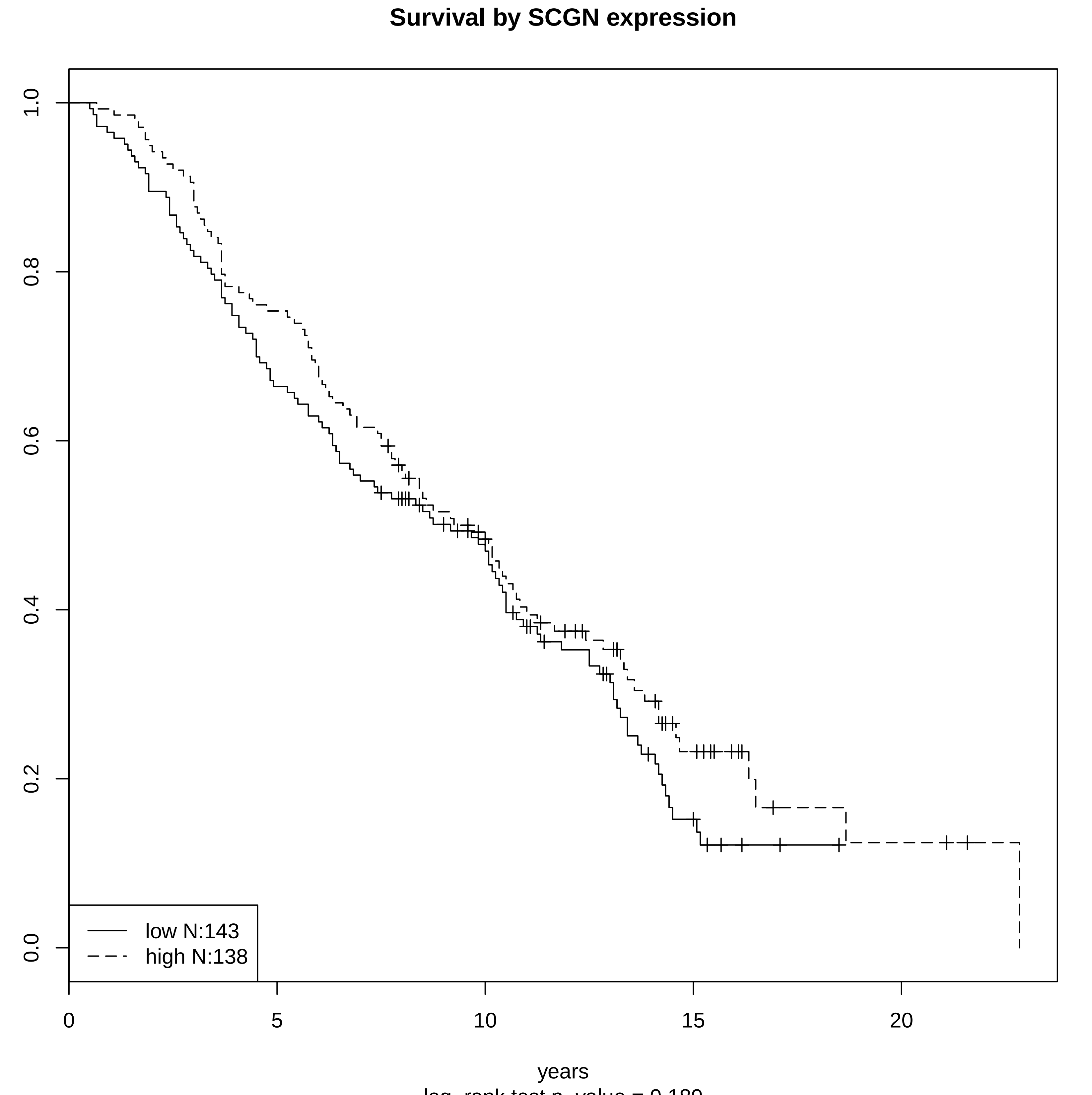
Survival by SCGN expression
1.0
0.8
0.6
0.4
0.2
0.0
0
5
10
15
20
low N:143
high N:138
years
log−rank test p−value = 0.189

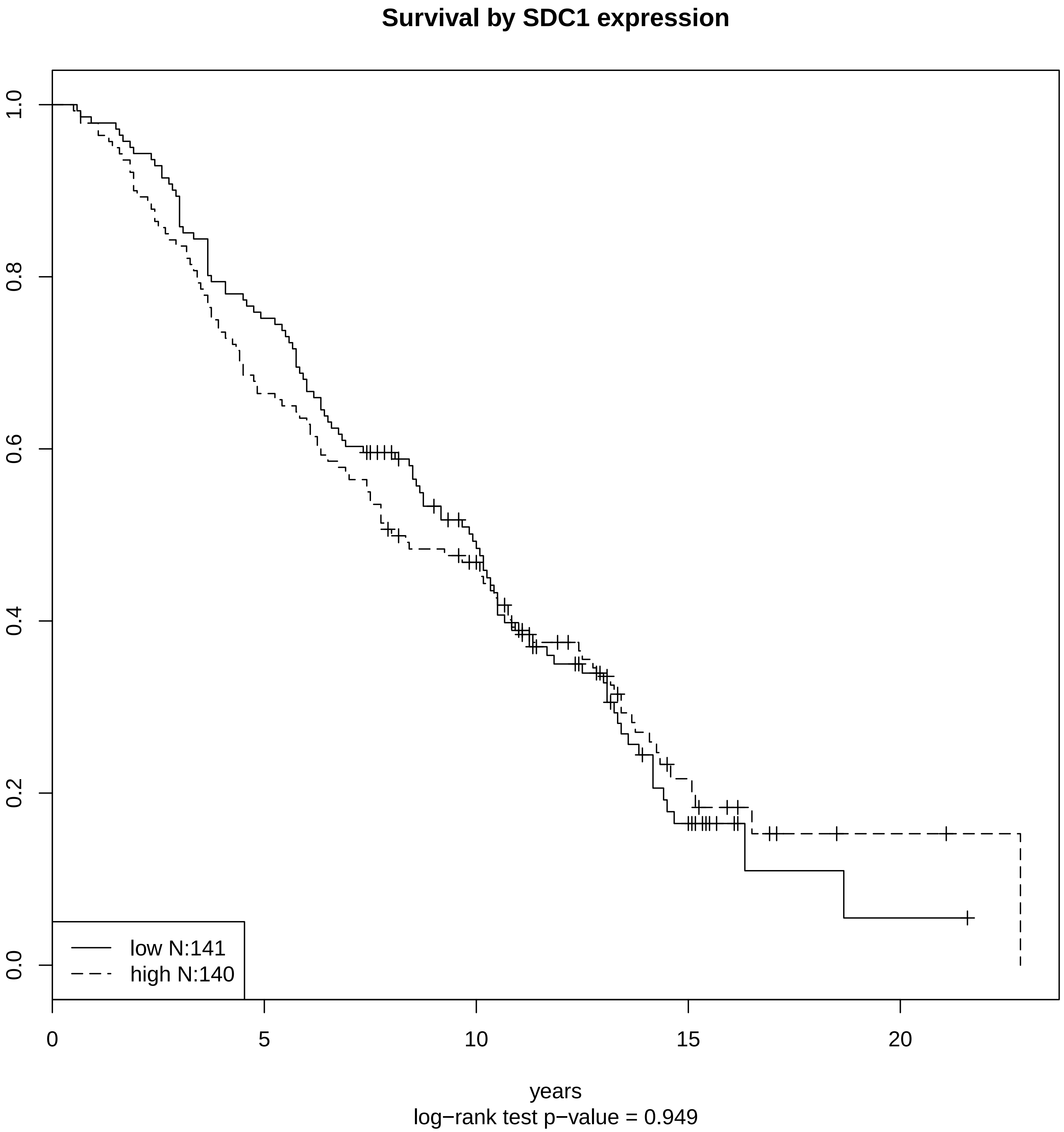

Survival by SDC1 expression
1.0
0.8
0.6
0.4
0.2
0.0
0
5
10
15
20
years
log−rank test p−value = 0.949
low N:141
high N:140

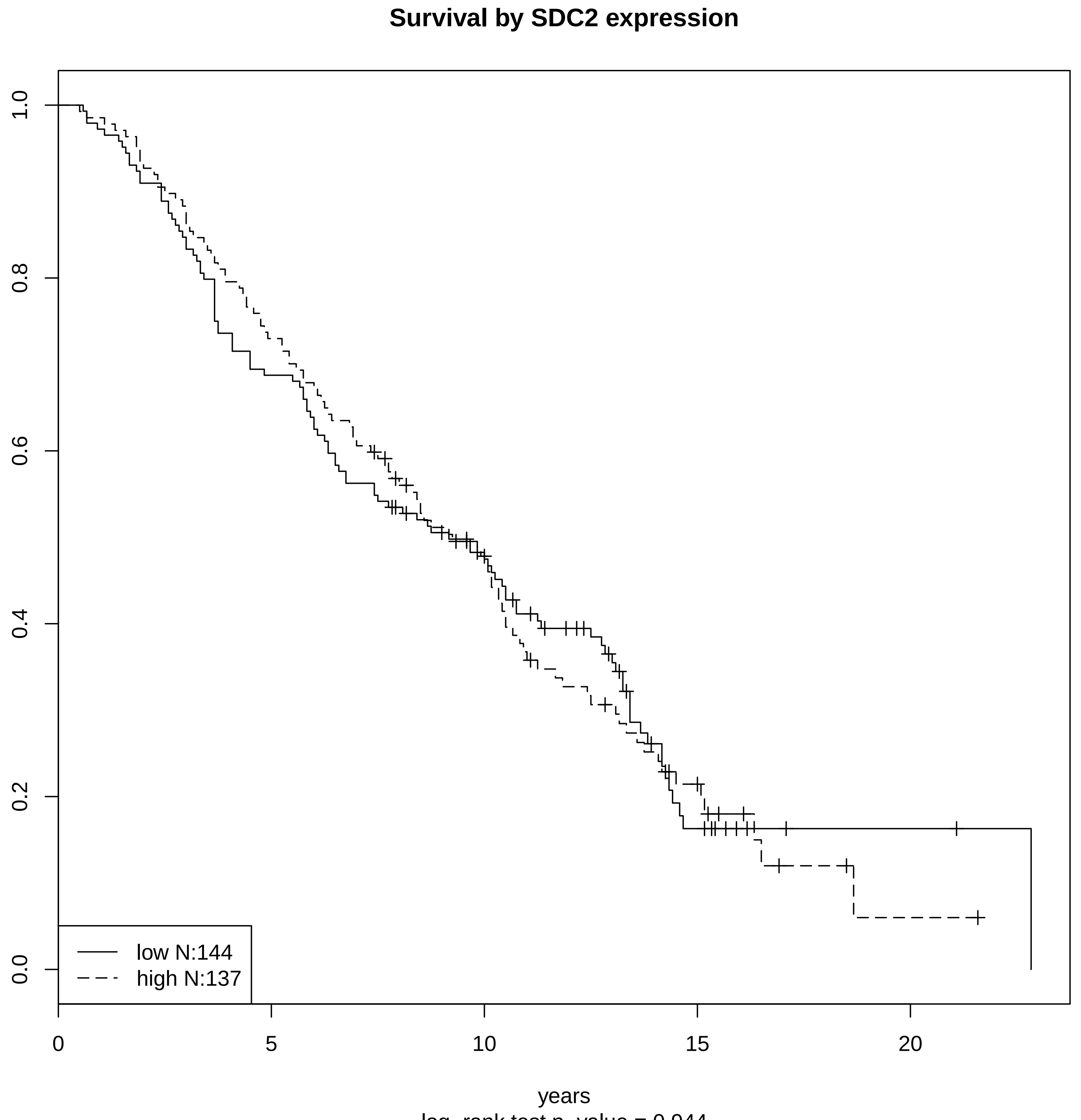
Survival by SDC2 expression
1.0
0.8
0.6
0.4
0.2
0.0
0
5
10
15
20
low N:144
high N:137
years
log−rank test p−value = 0.944

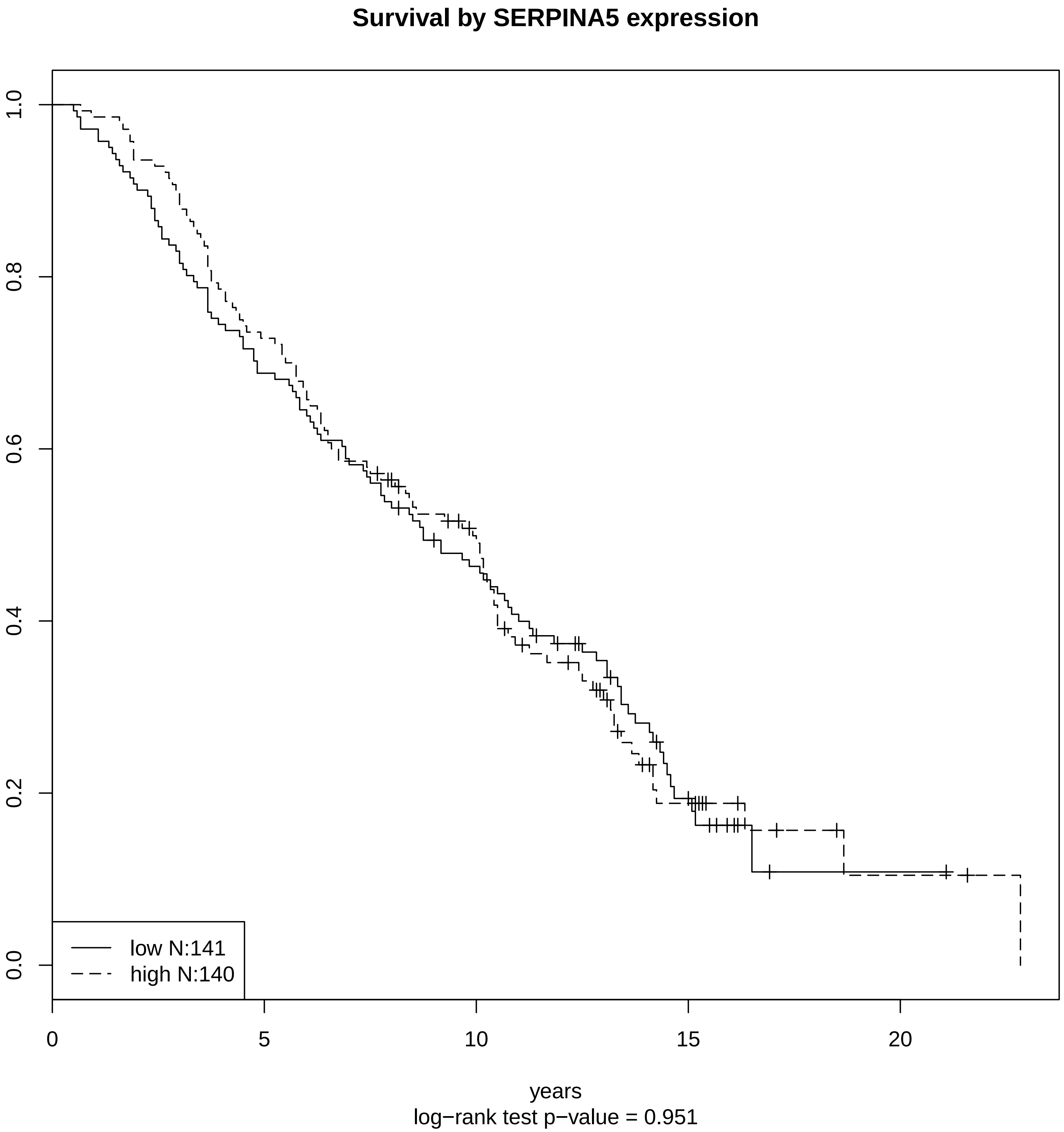
Survival by SERPINA5 expression
1.0
0.8
0.6
0.4
0.2
0.0
0
5
10
15
20
years
log−rank test p−value = 0.951
low N:141
high N:140

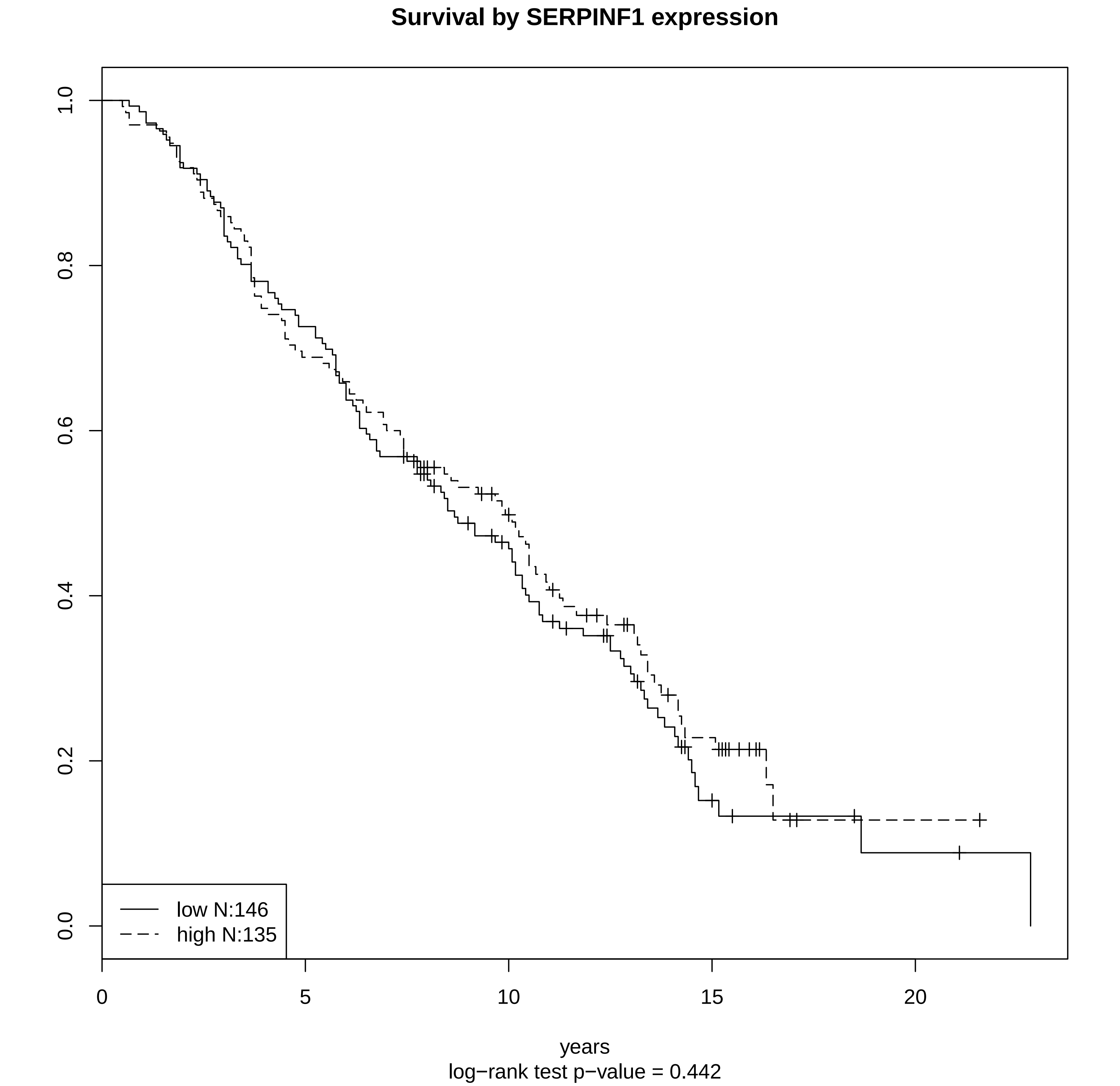

Survival by SERPINF1 expression
1.0
0.8
0.6
0.4
0.2
0.0
0
5
10
15
20
years
low N:146
high N:135
log−rank test p−value = 0.442

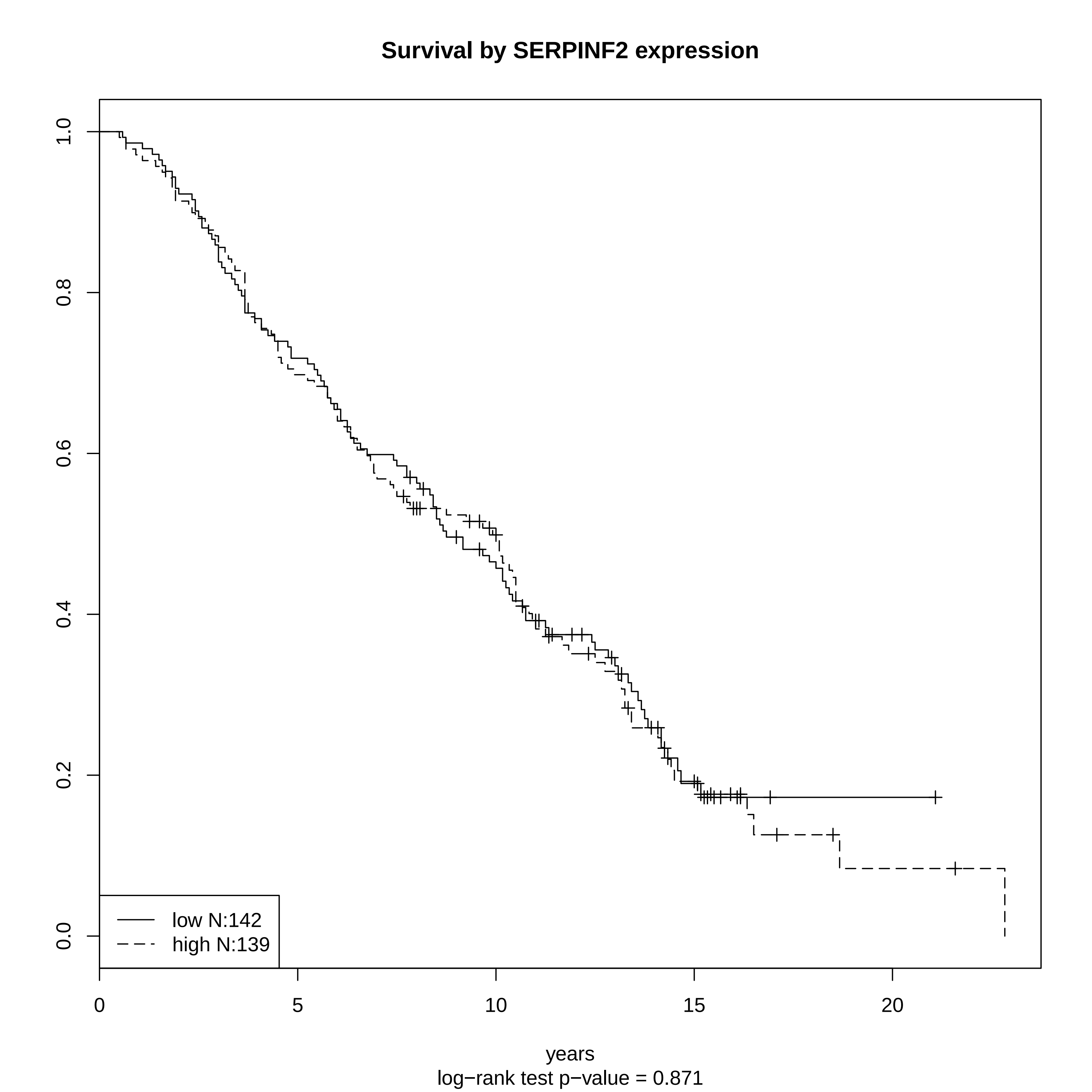

Survival by SERPINF2 expression
1.0
0.8
0.6
0.4
0.2
0.0
0
5
10
15
20
low N:142
high N:139
years
log−rank test p−value = 0.871

Survival by SHBG expression

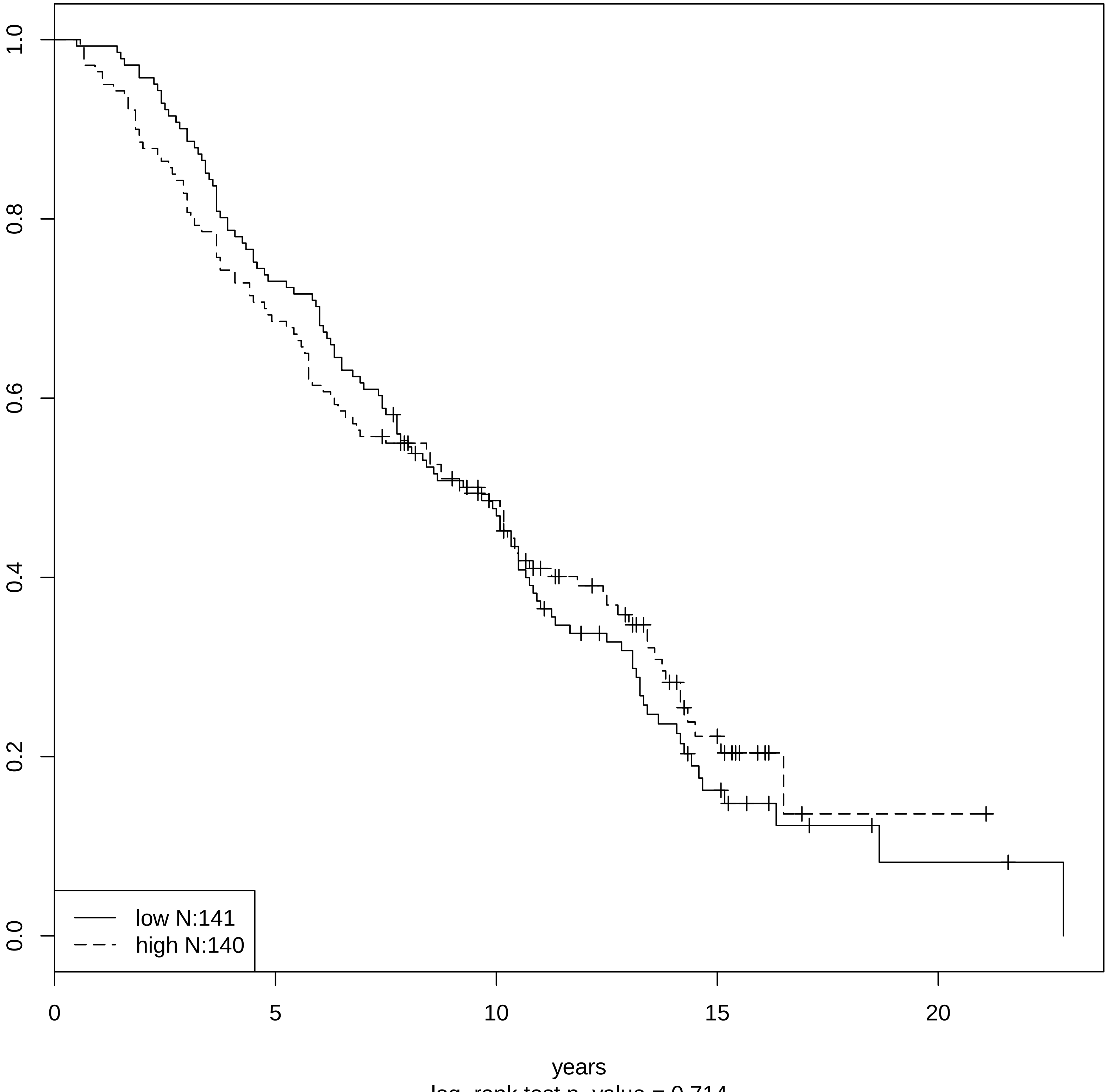

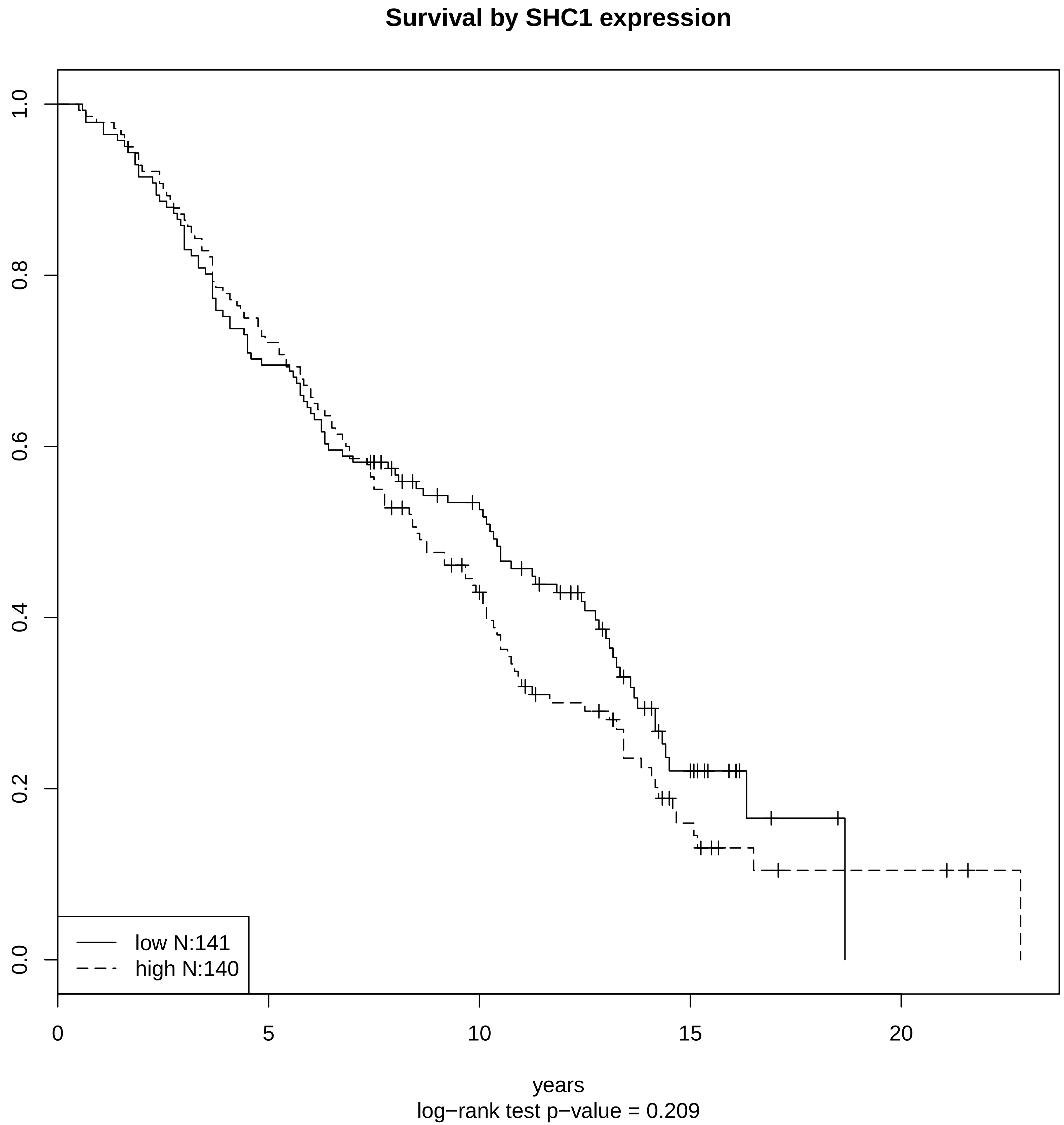
Survival by SHC1 expression
1.0
0.8
0.6
0.4
0.2
0.0
0
5
10
15
20
years
log−rank test p−value = 0.209
low N:141
high N:140

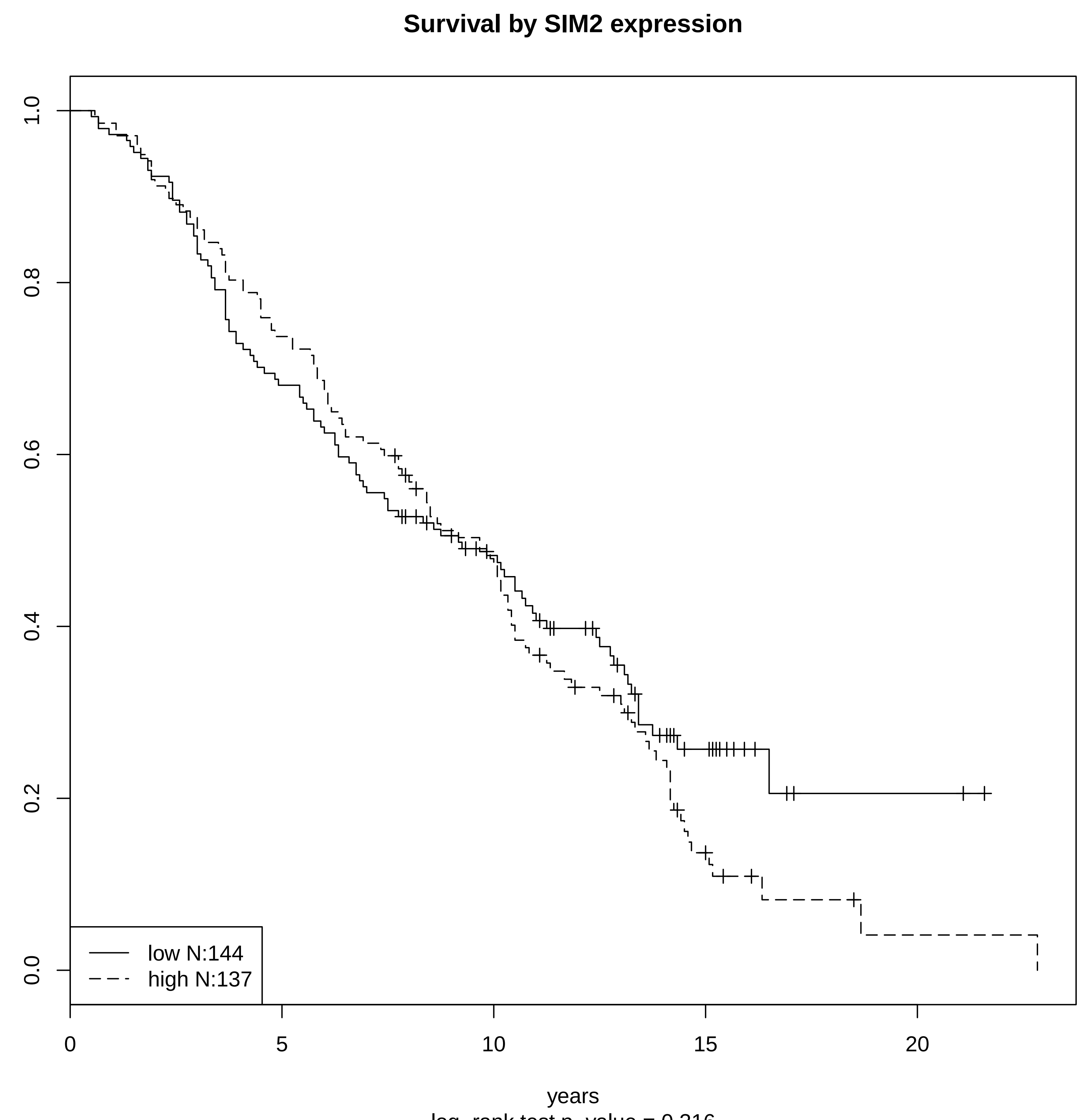

Survival by SIM2 expression
1.0
0.8
0.6
0.4
0.2
0.0
0
5
10
15
20
low N:144
high N:137
years
log−rank test p−value = 0.316

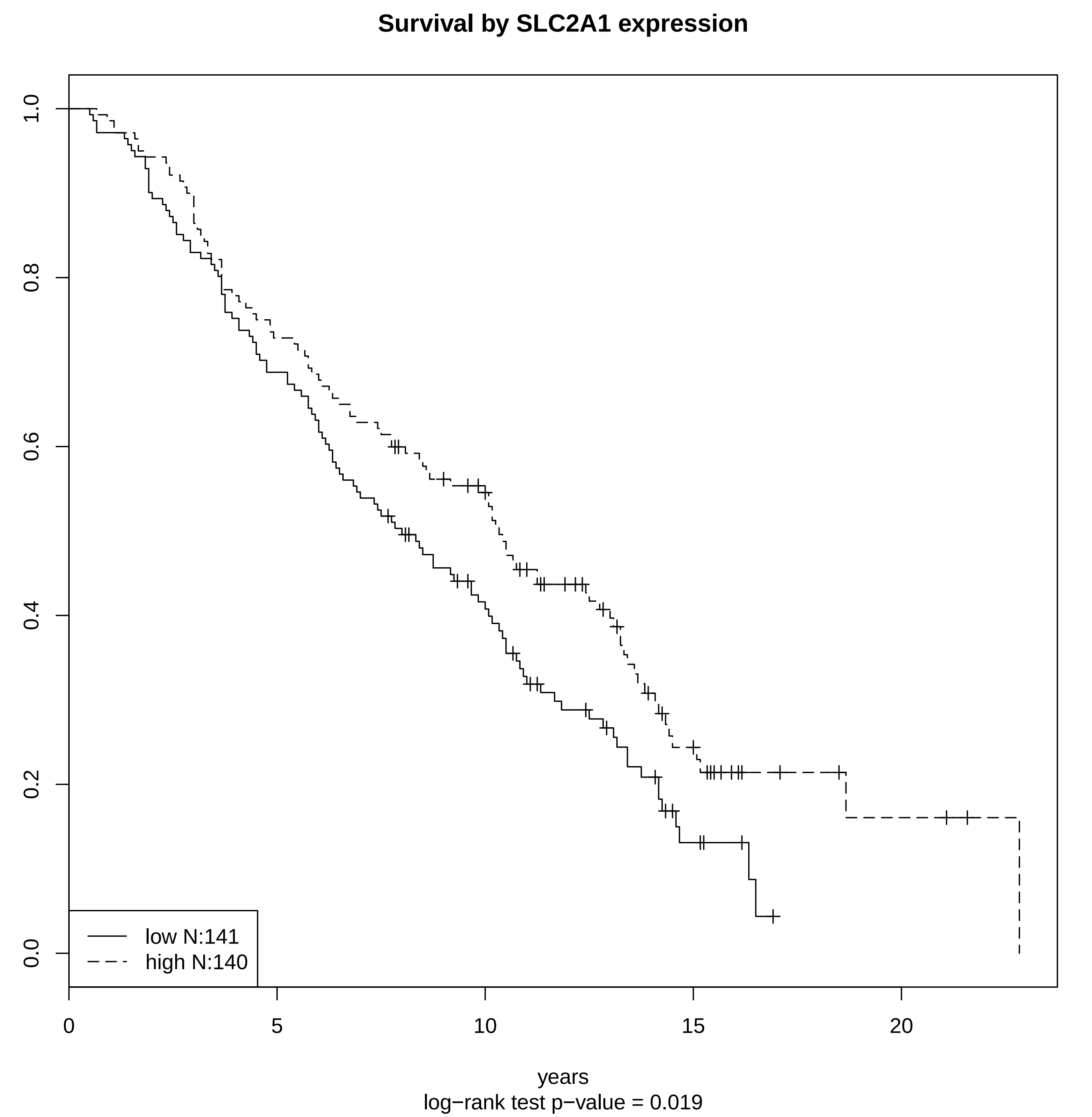
Survival by SLC2A1 expression
1.0
0.8
0.6
0.4
0.2
0.0
0
5
10
15
20
low N:141
high N:140
years
log−rank test p−value = 0.019

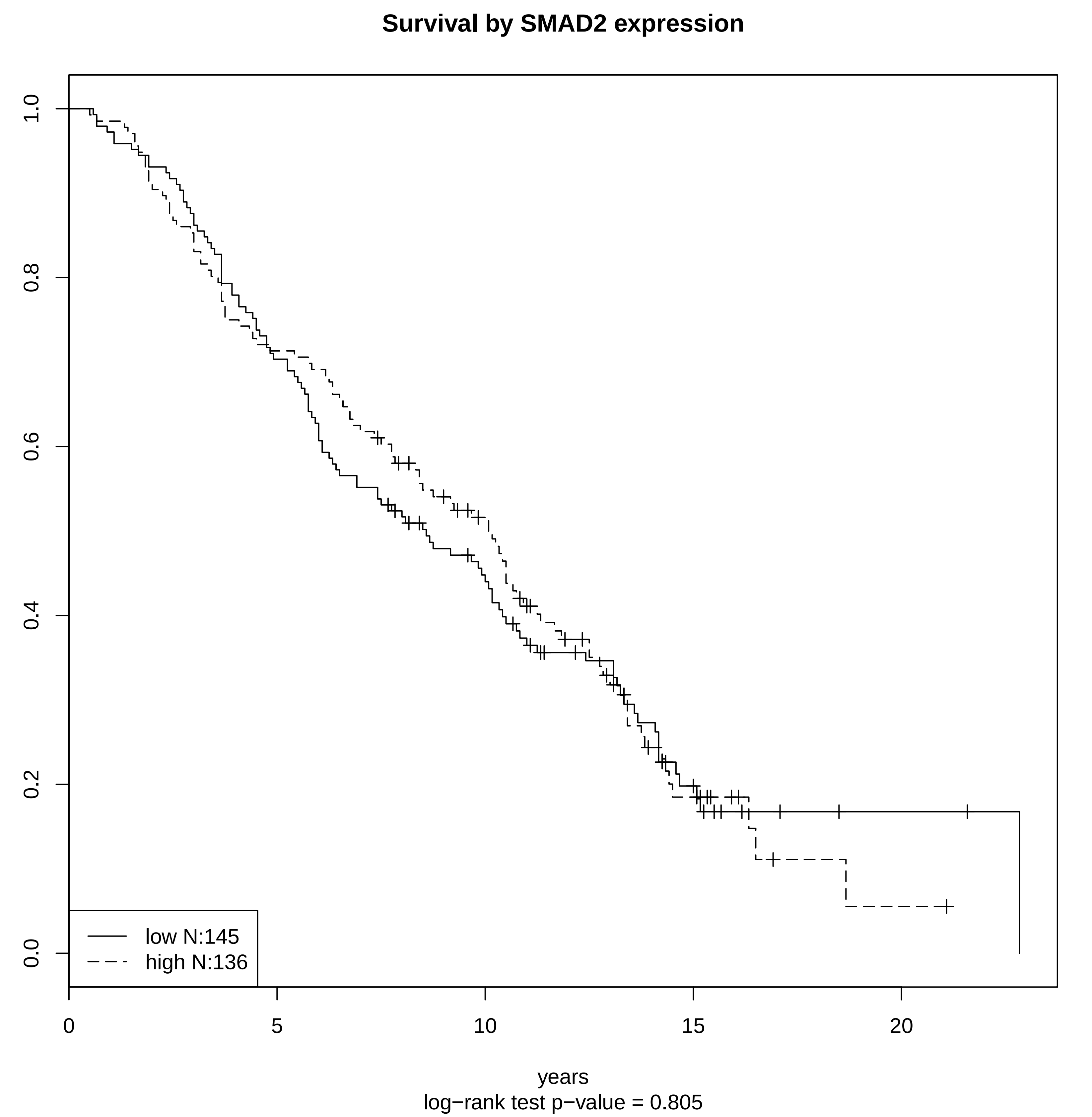

Survival by SMAD2 expression
1.0
0.8
0.6
0.4
0.2
0.0
0
5
10
15
20
years
log−rank test p−value = 0.805
low N:145
high N:136

Survival by SMAD3 expression

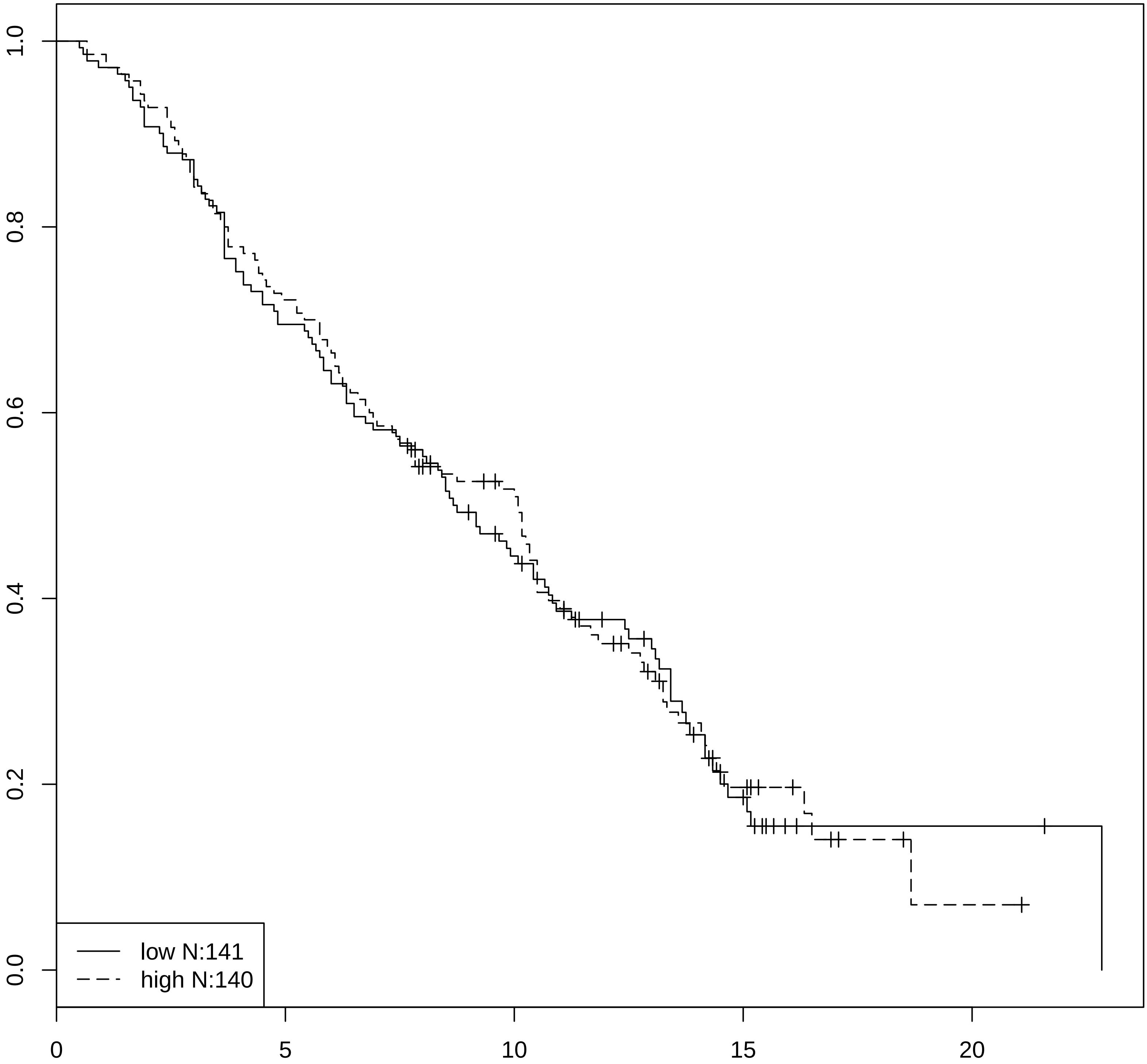

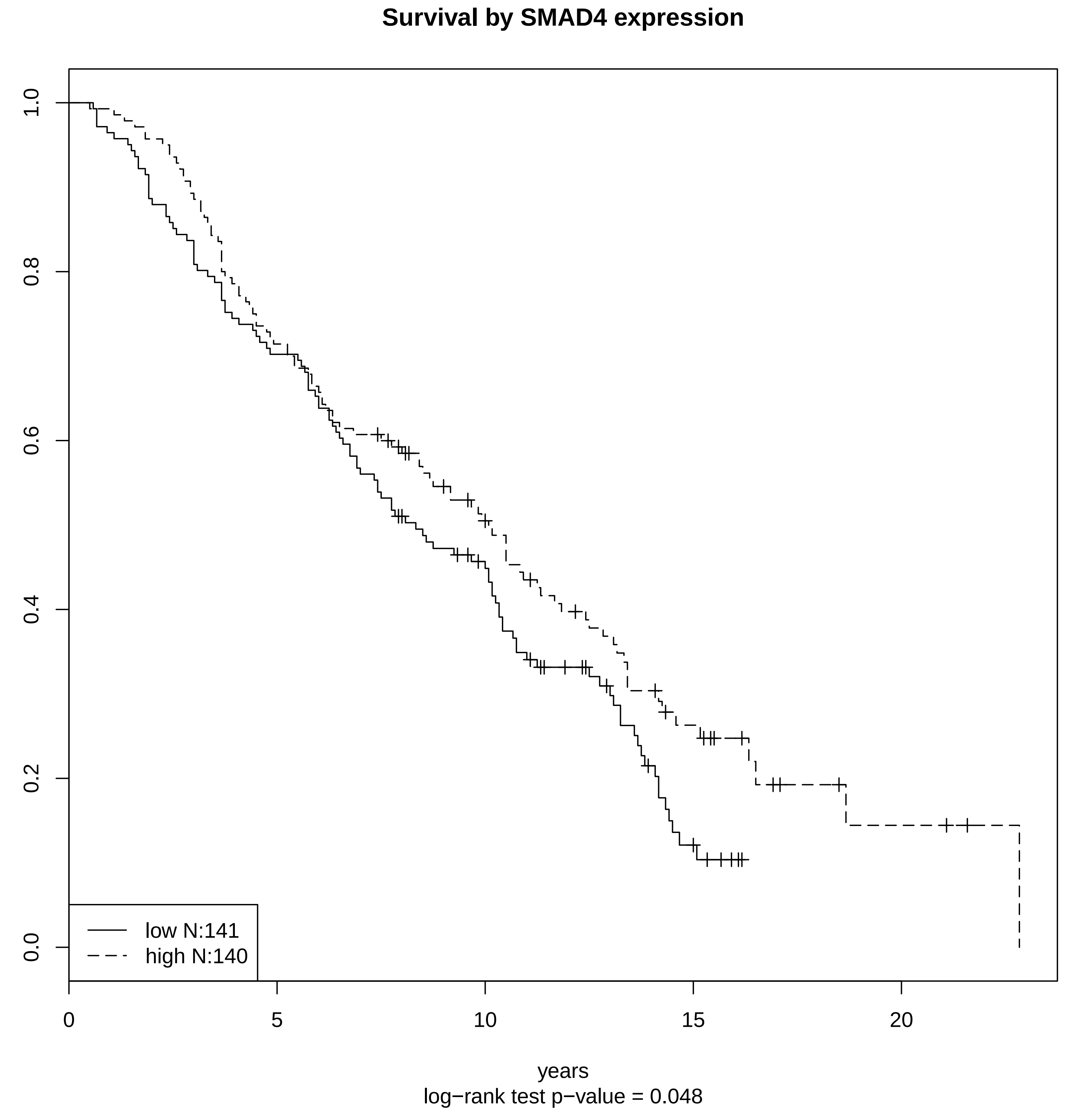

Survival by SMAD4 expression
1.0
0.8
0.6
0.4
0.2
0.0
0
5
10
15
20
low N:141
high N:140
years
log−rank test p−value = 0.048

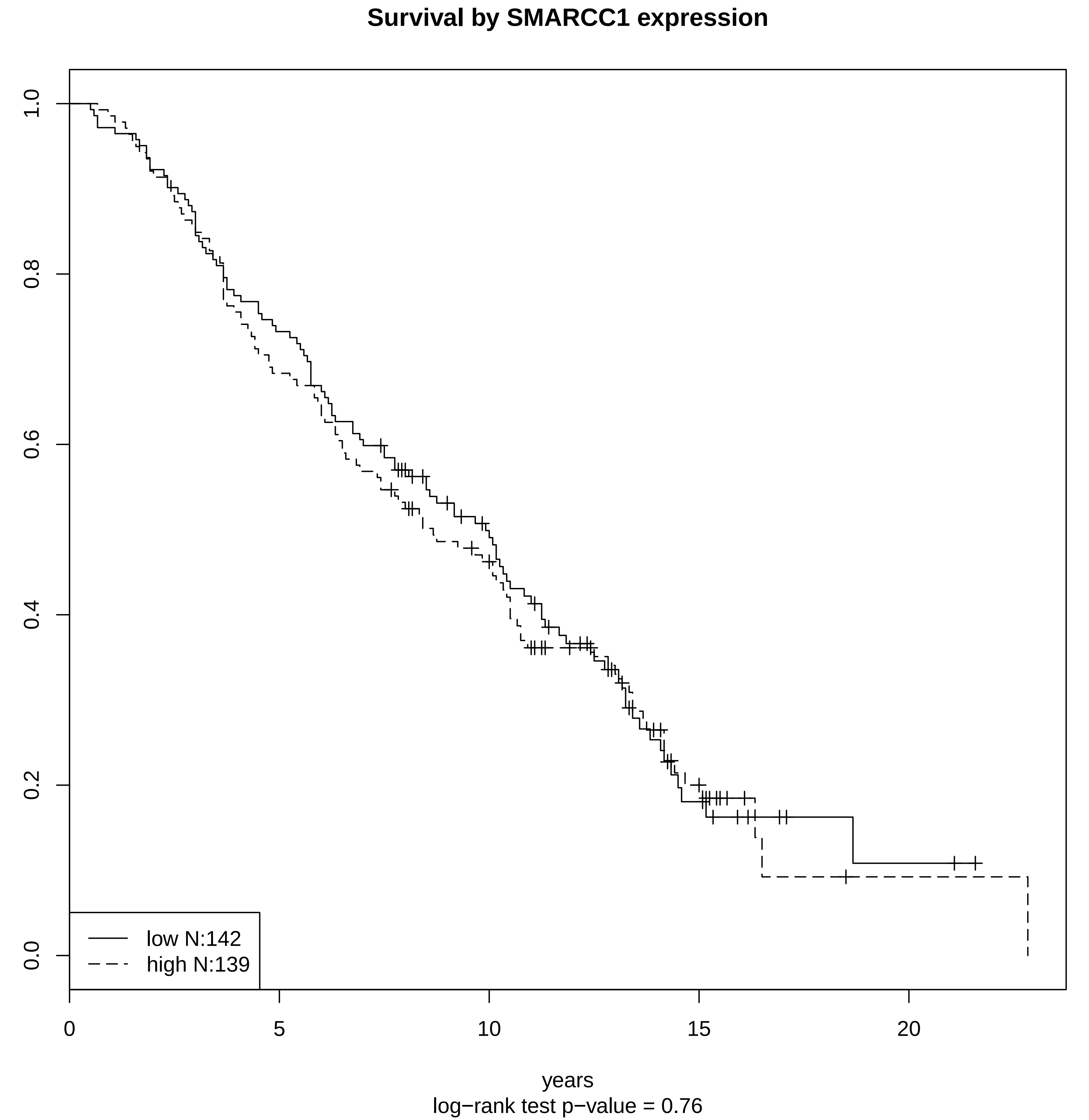
Survival by SMARCC1 expression
1.0
0.8
0.6
0.4
0.2
0.0
0
5
10
15
20
low N:142
high N:139
years
log−rank test p−value = 0.76

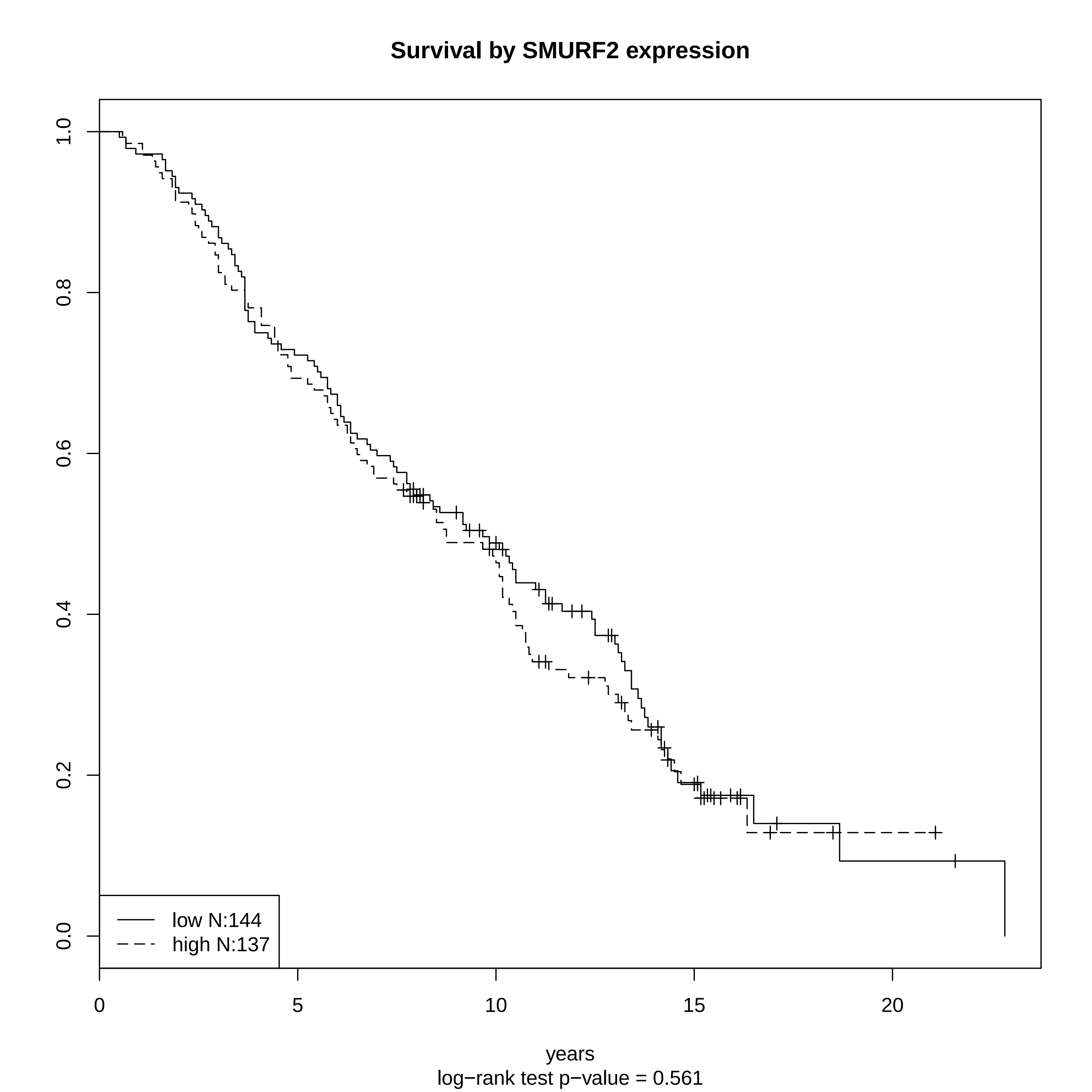
Survival by SMURF2 expression
1.0
0.8
0.6
0.4
0.2
0.0
0
5
10
15
20
years
log−rank test p−value = 0.561
low N:144
high N:137

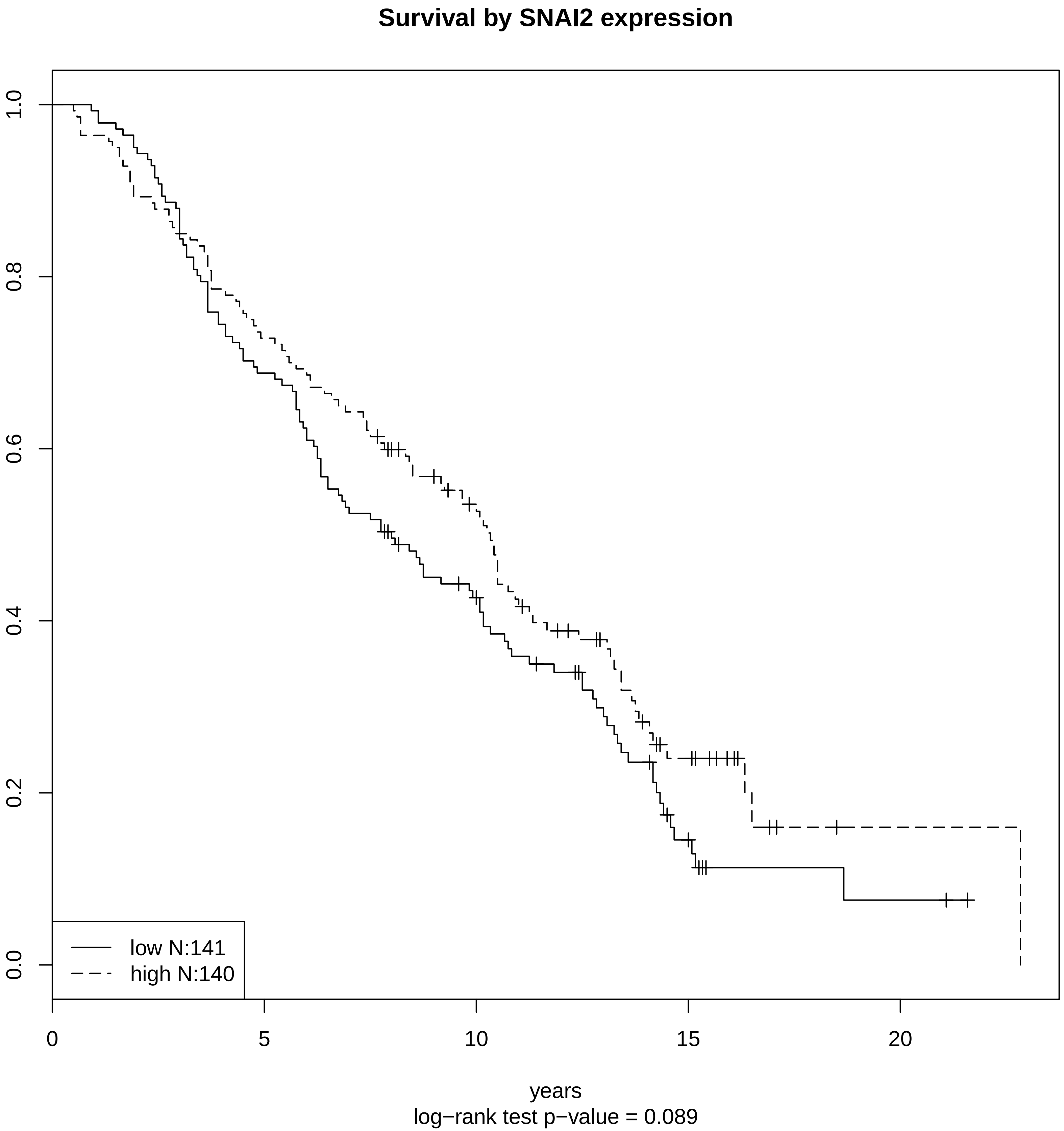
Survival by SNAI2 expression
1.0
0.8
0.6
0.4
0.2
0.0
0
5
10
15
20
low N:141
high N:140
years
log−rank test p−value = 0.089

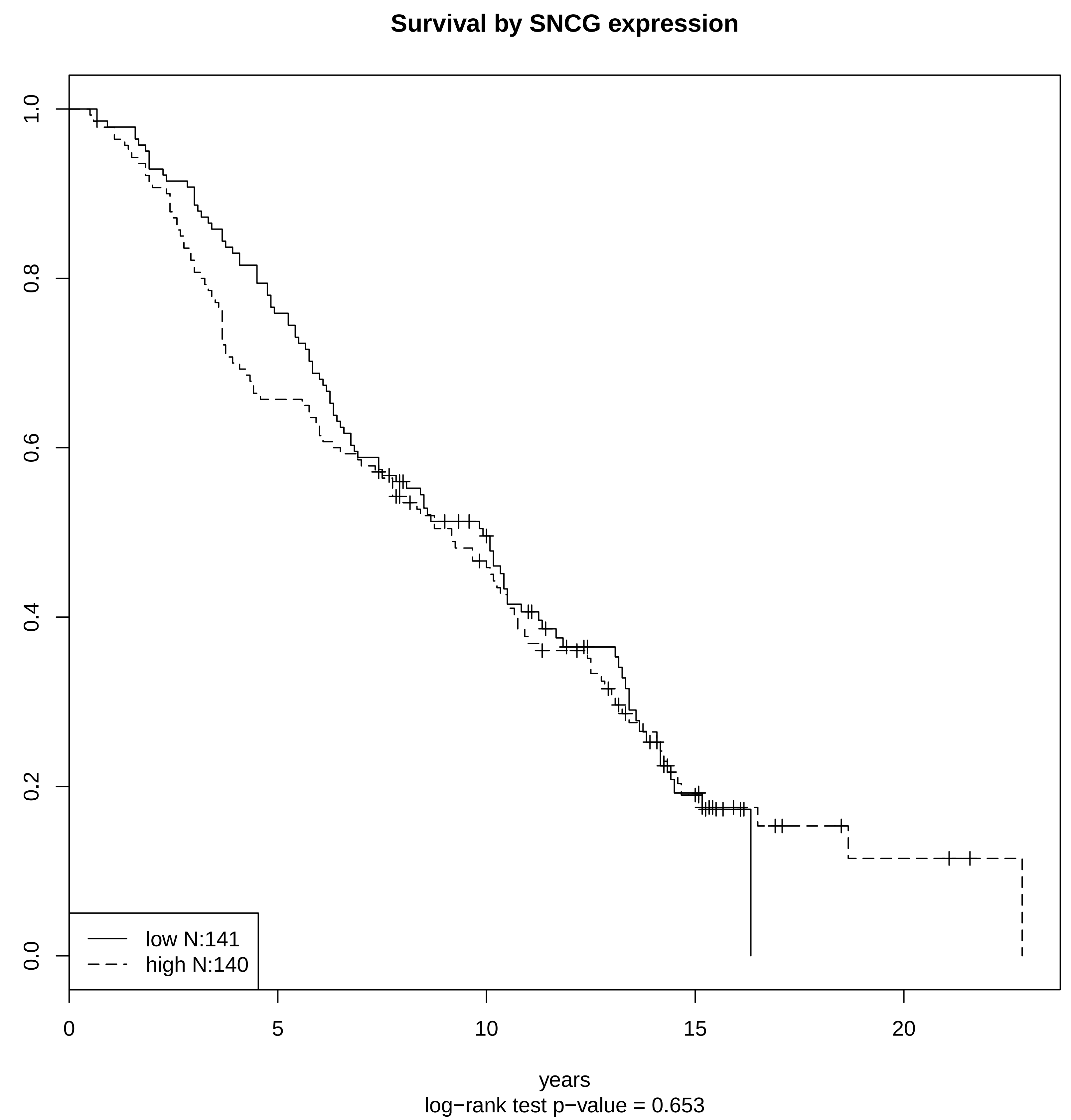
Survival by SNCG expression
1.0
0.8
0.6
0.4
0.2
0.0
0
5
10
15
20
years
log−rank test p−value = 0.653
low N:141
high N:140

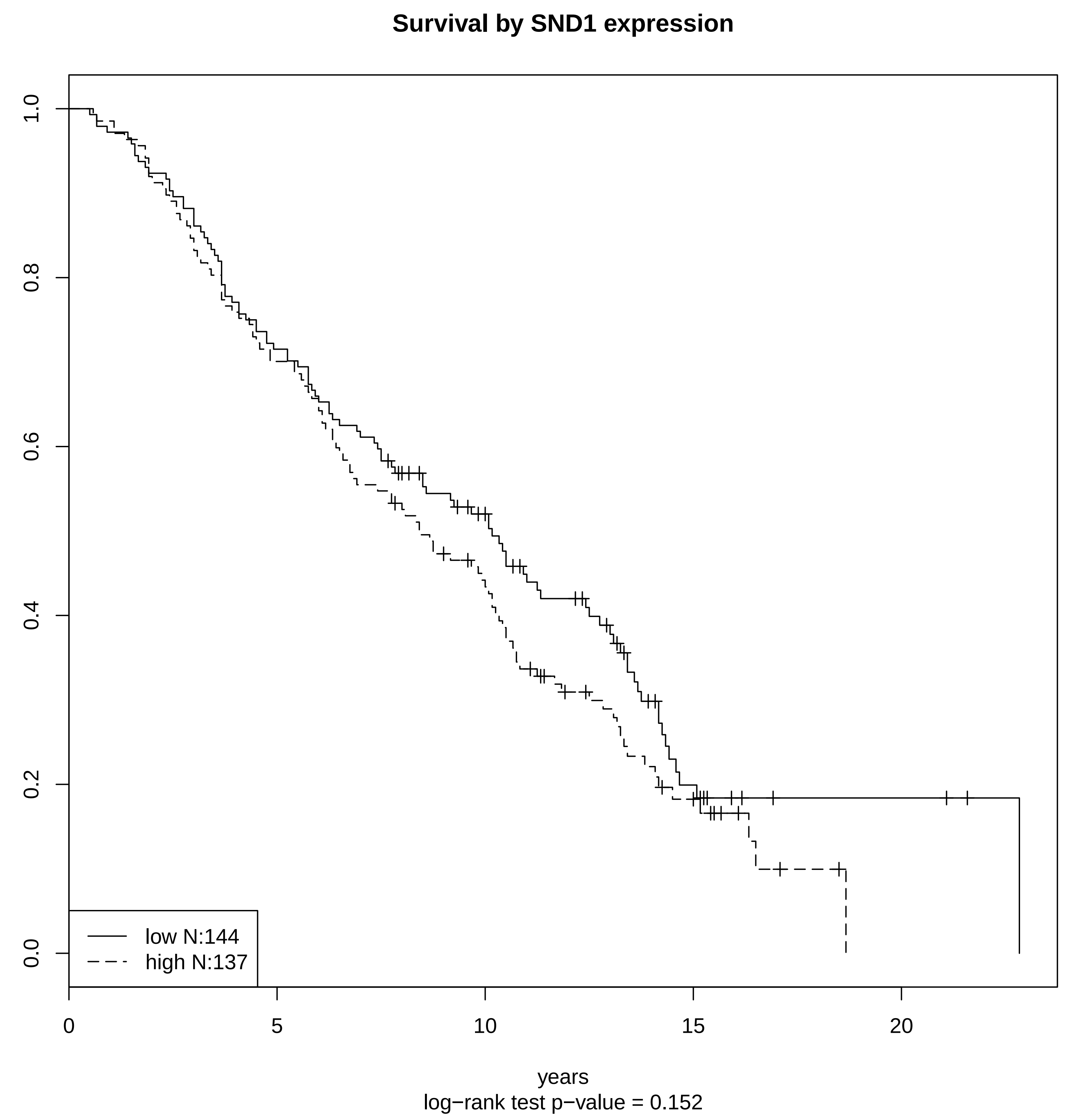
Survival by SND1 expression
1.0
0.8
0.6
0.4
0.2
0.0
0
5
10
15
20
years
log−rank test p−value = 0.152
low N:144
high N:137

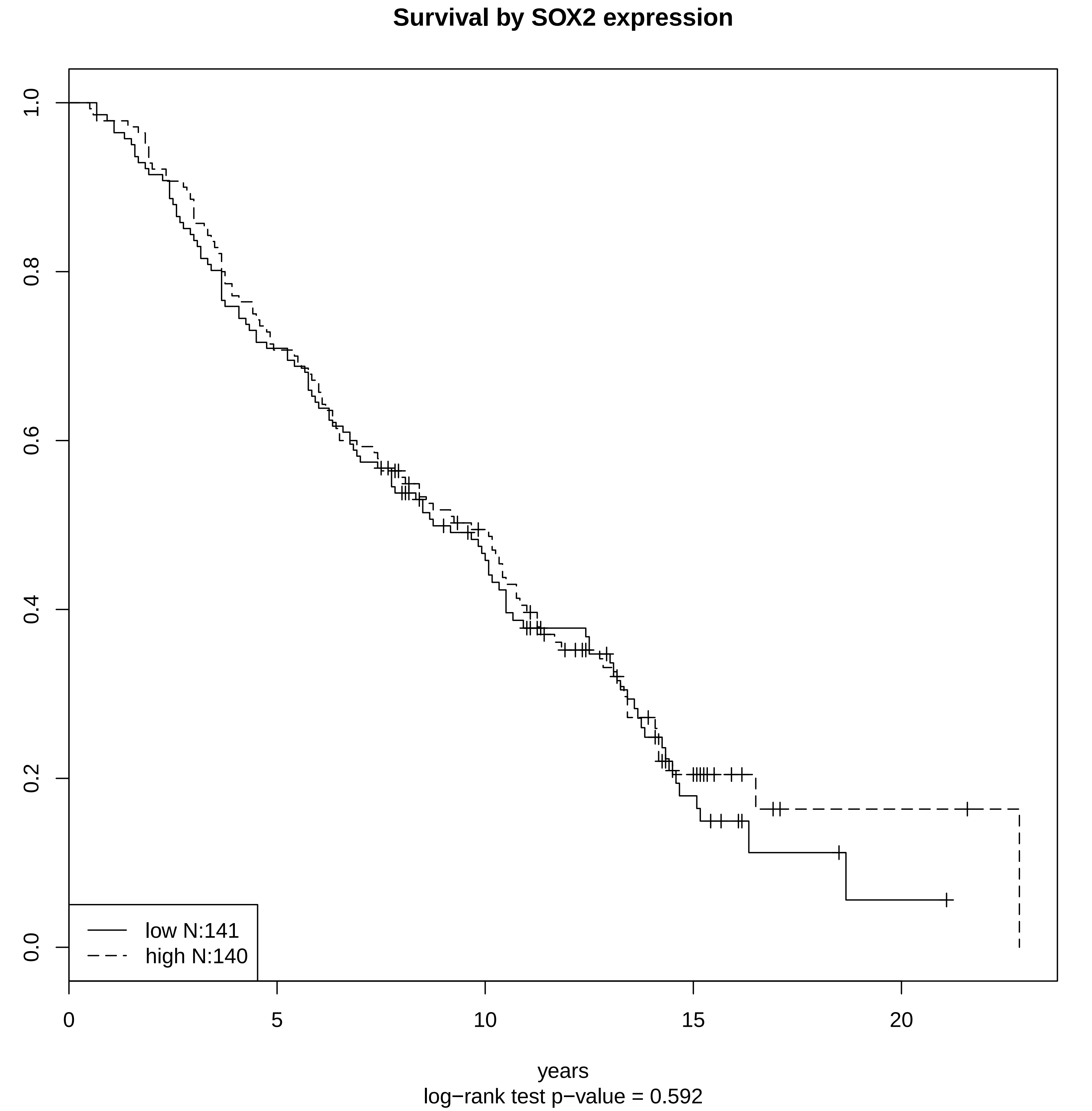

Survival by SOX2 expression
1.0
0.8
0.6
0.4
0.2
0.0
0
5
10
15
20
years
log−rank test p−value = 0.592
low N:141
high N:140

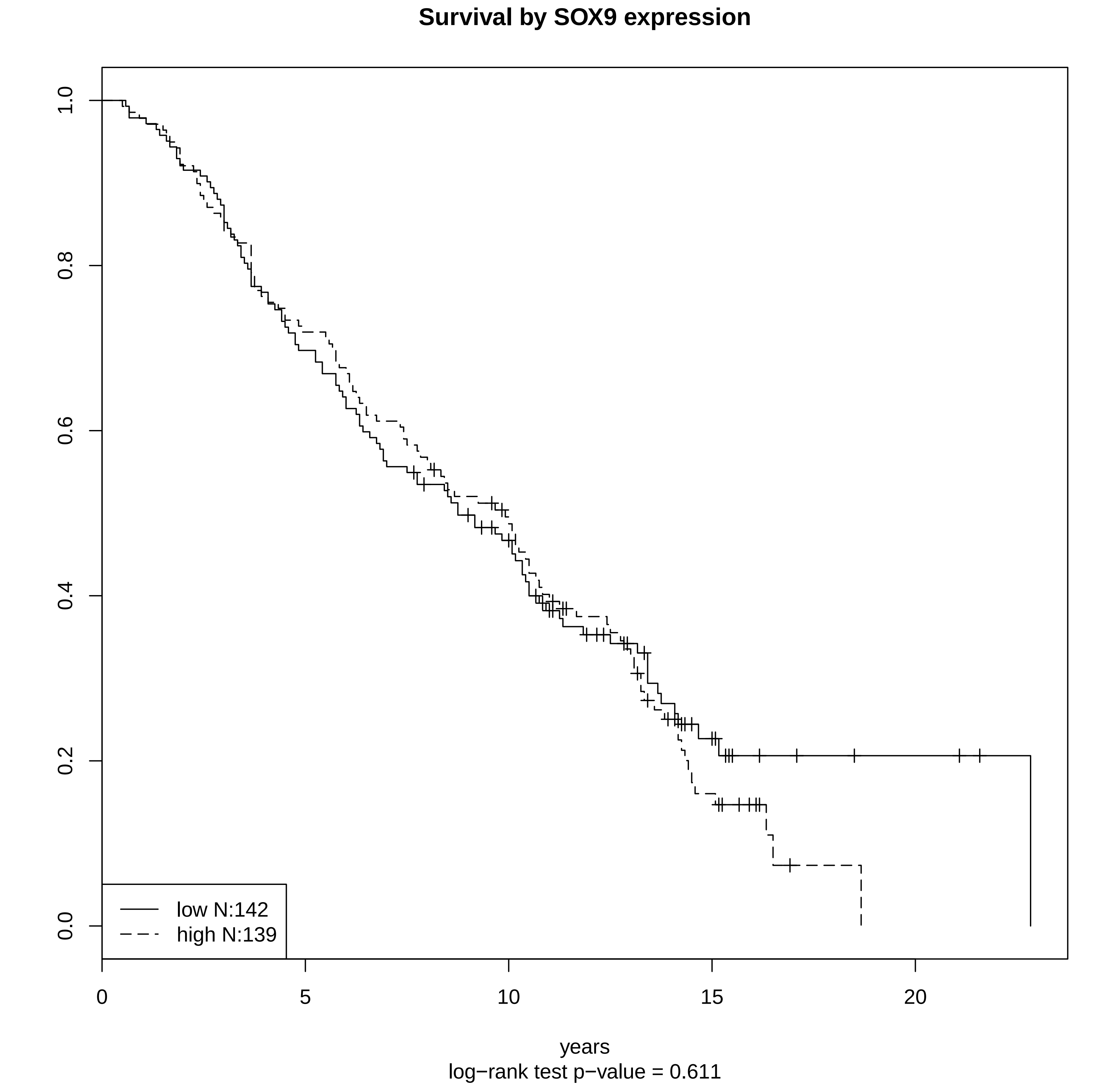

Survival by SOX9 expression
1.0
0.8
0.6
0.4
0.2
0.0
0
5
10
15
20
low N:142
high N:139
years
log−rank test p−value = 0.611

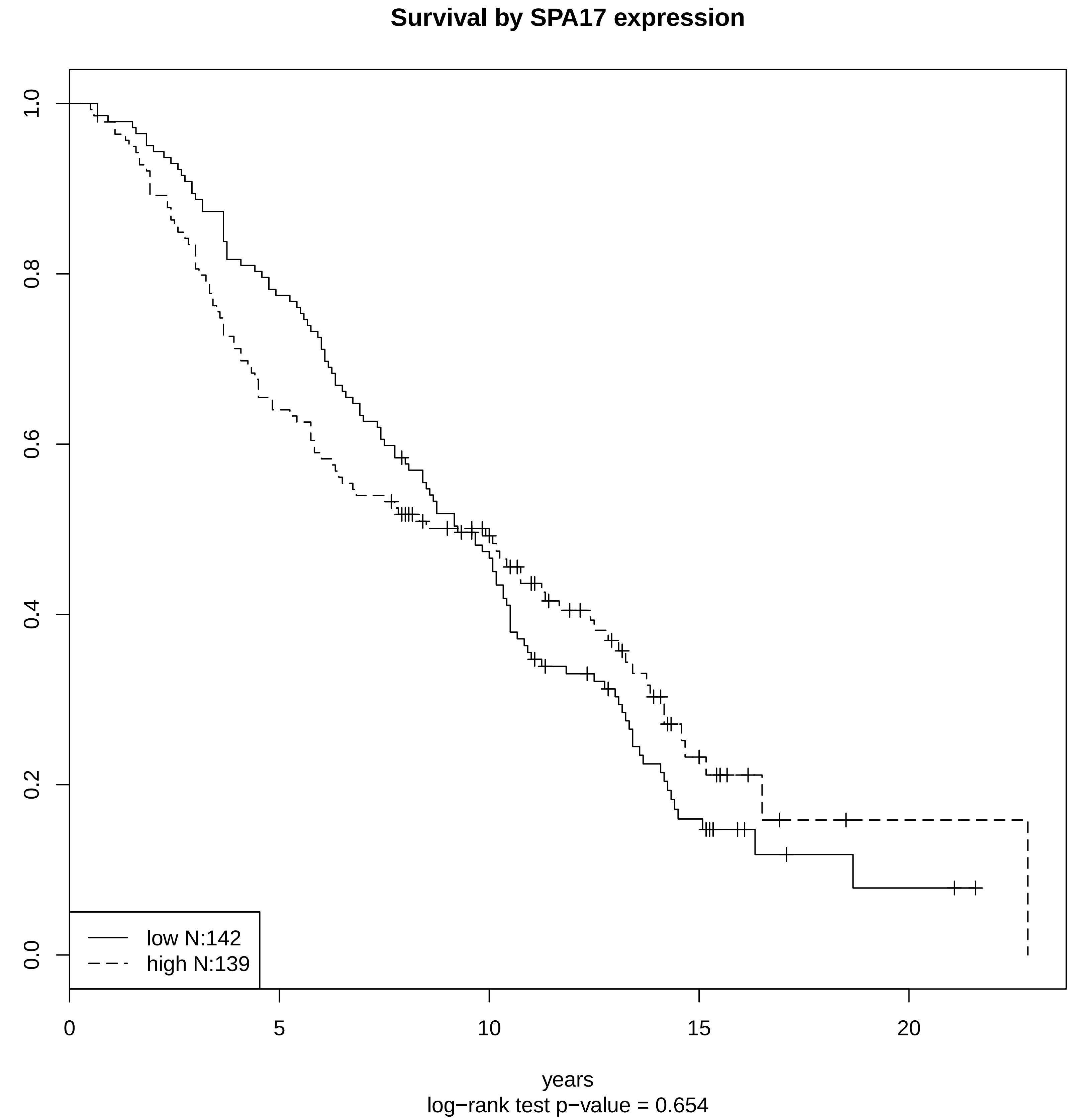
Survival by SPA17 expression
1.0
0.8
0.6
0.4
0.2
0.0
0
5
10
15
20
low N:142
high N:139
years
log−rank test p−value = 0.654

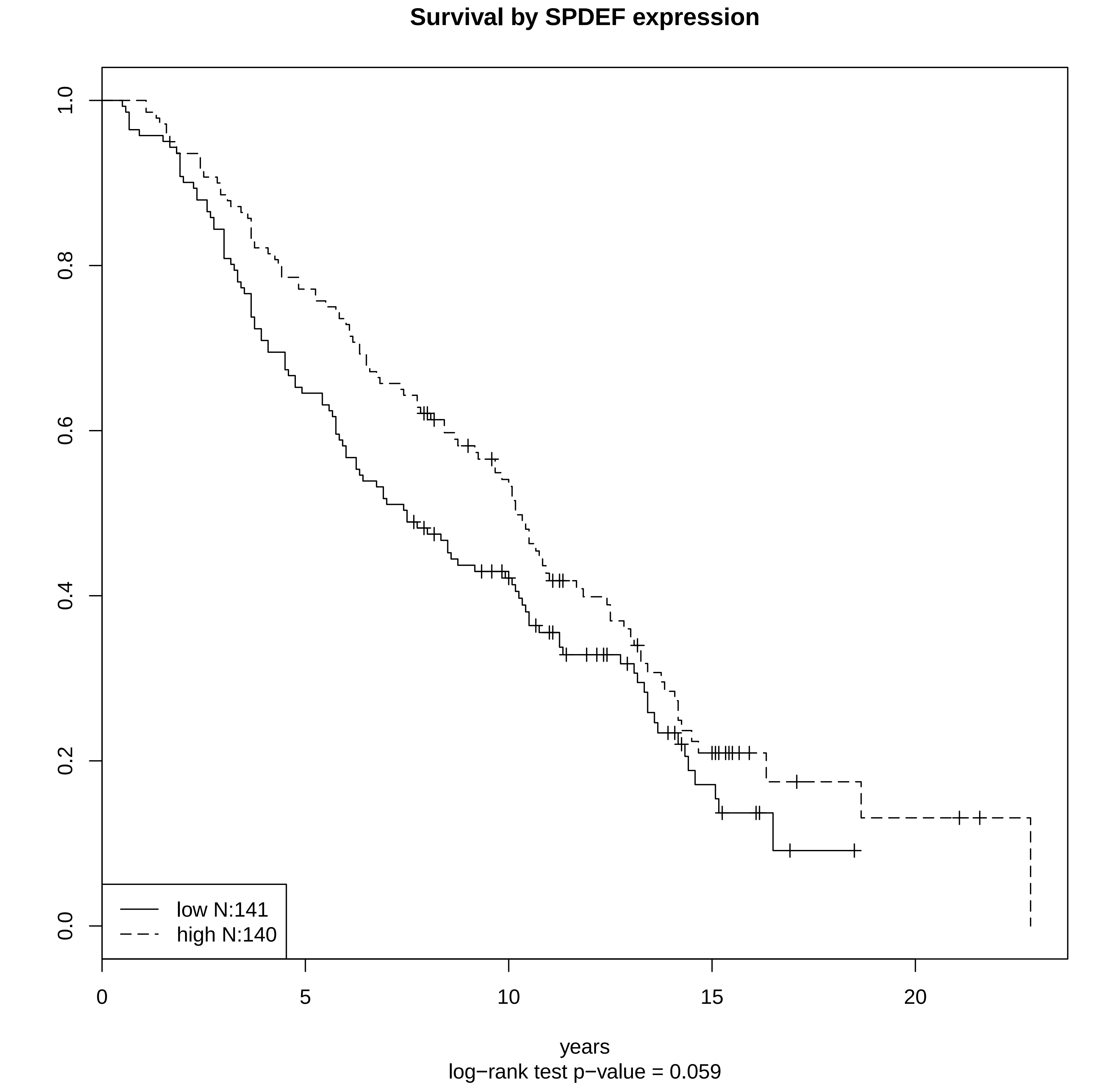
Survival by SPDEF expression
1.0
0.8
0.6
0.4
0.2
0.0
0
5
10
15
20
low N:141
high N:140
years
log−rank test p−value = 0.059

Survival by SPINK1 expression

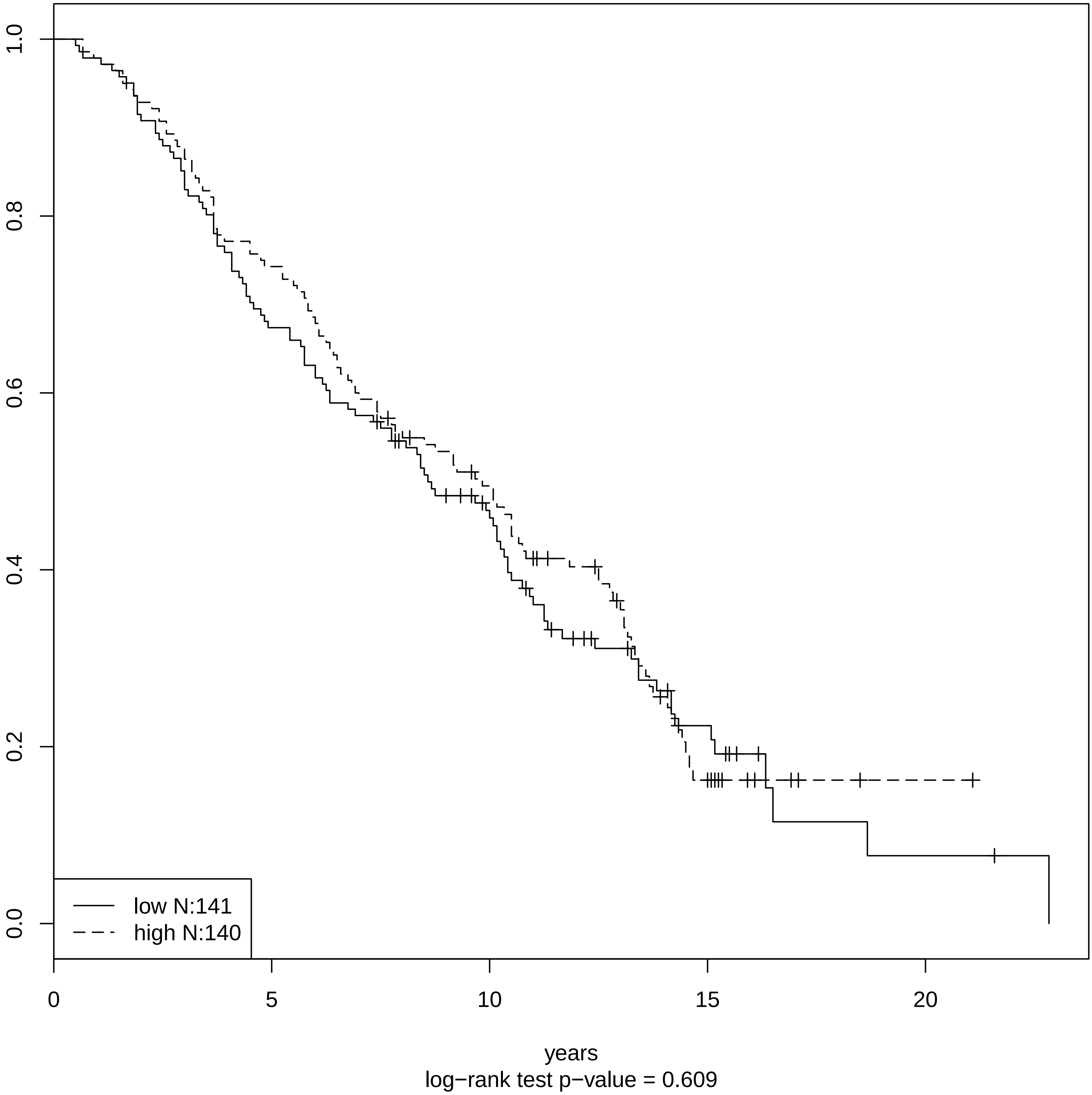

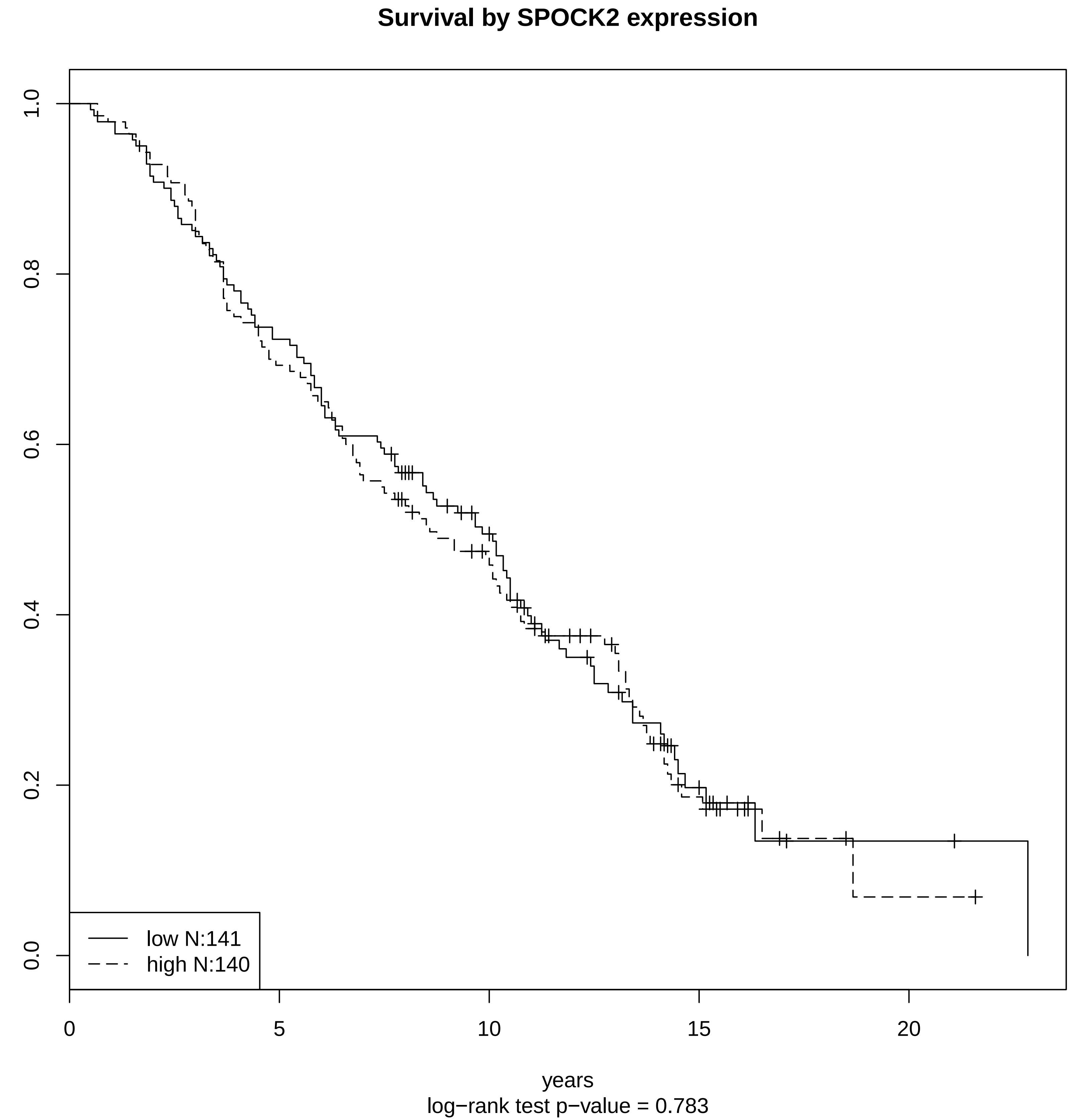
Survival by SPOCK2 expression
1.0
0.8
0.6
0.4
0.2
0.0
low N:141
high N:140
0
5
10
15
20
years
log−rank test p−value = 0.783

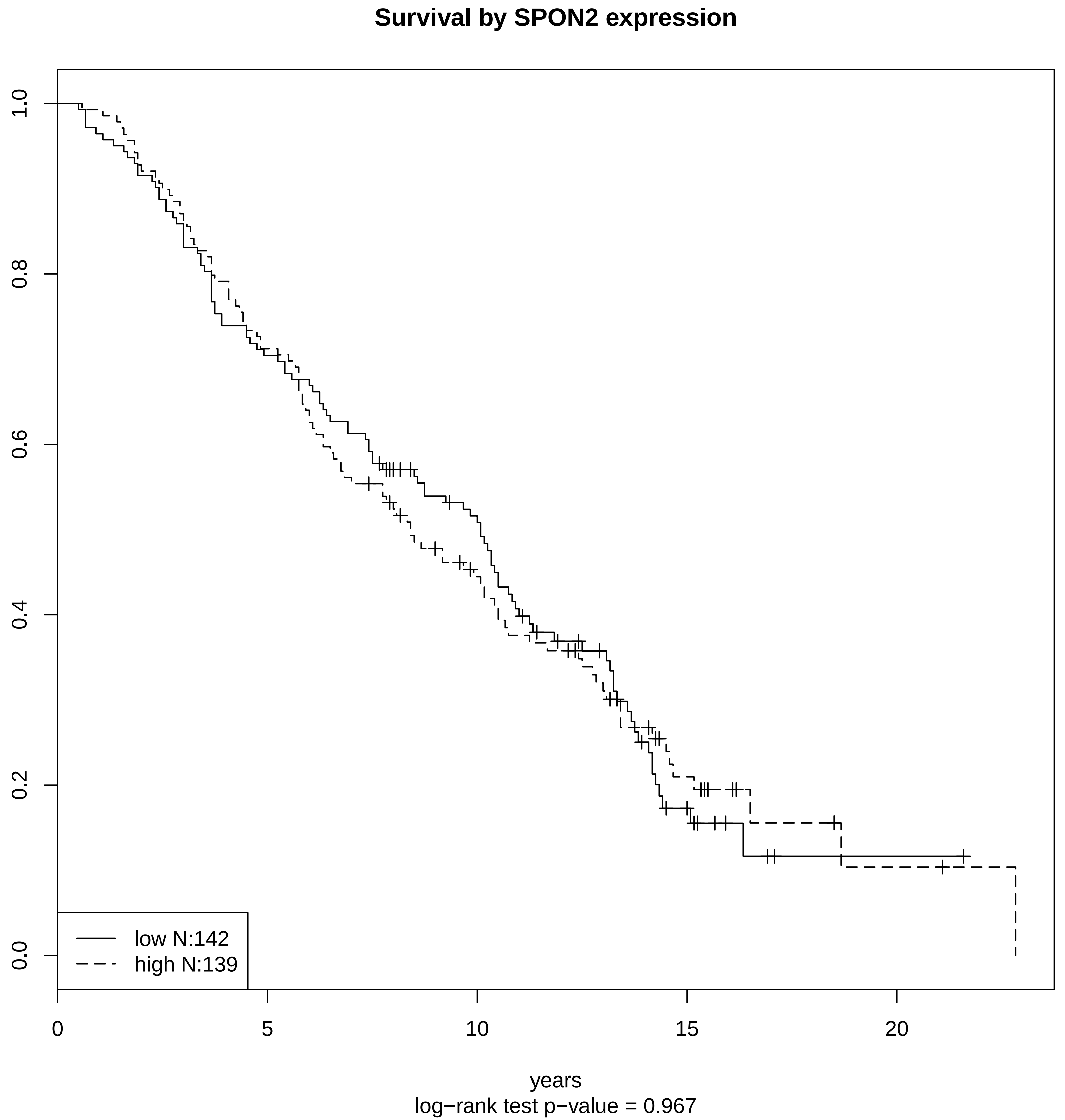
Survival by SPON2 expression
1.0
0.8
0.6
0.4
0.2
0.0
0
5
10
15
20
low N:142
high N:139
years
log−rank test p−value = 0.967

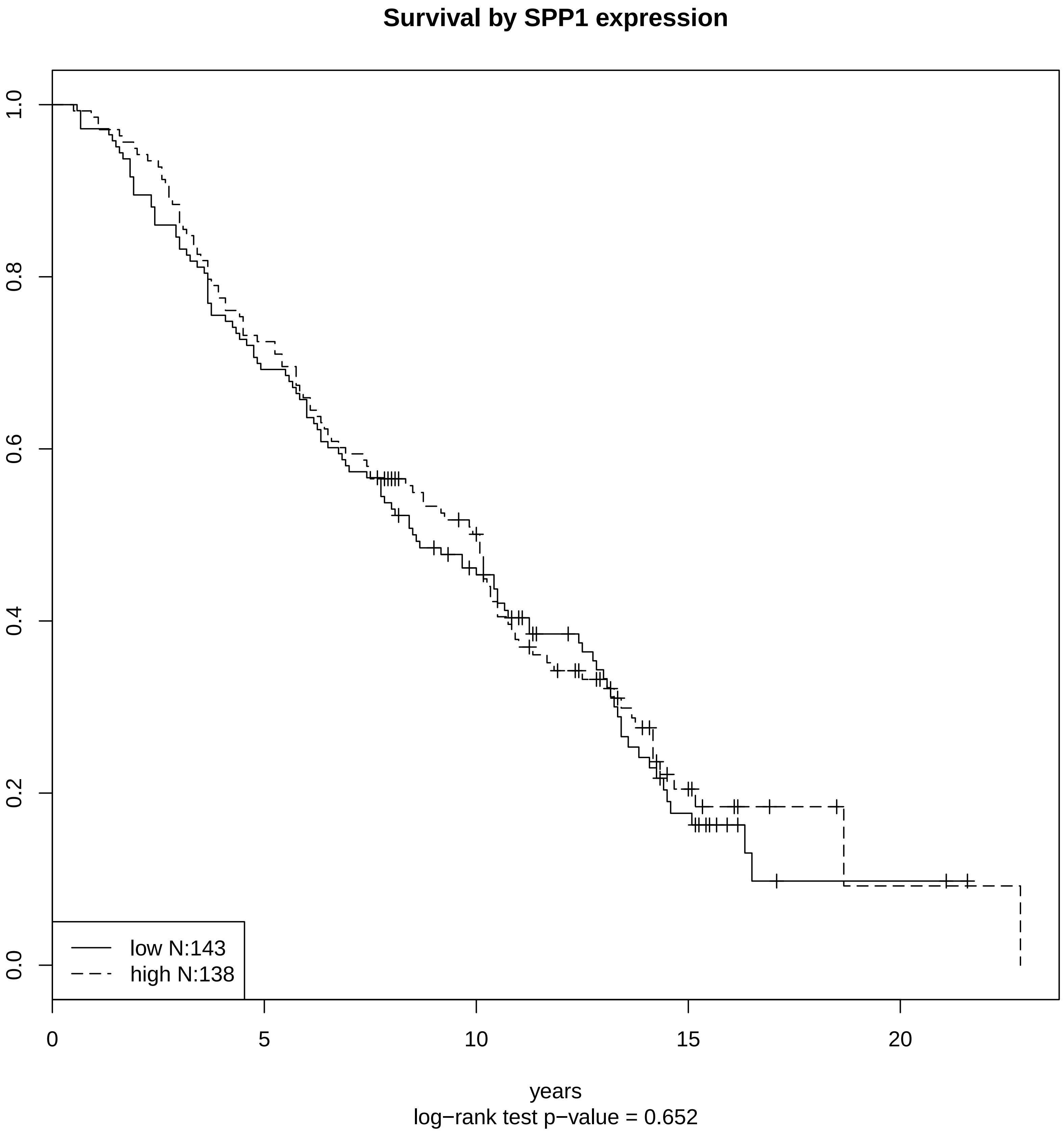
Survival by SPP1 expression
1.0
0.8
0.6
0.4
0.2
0.0
0
5
10
15
20
years
low N:143
high N:138
log−rank test p−value = 0.652

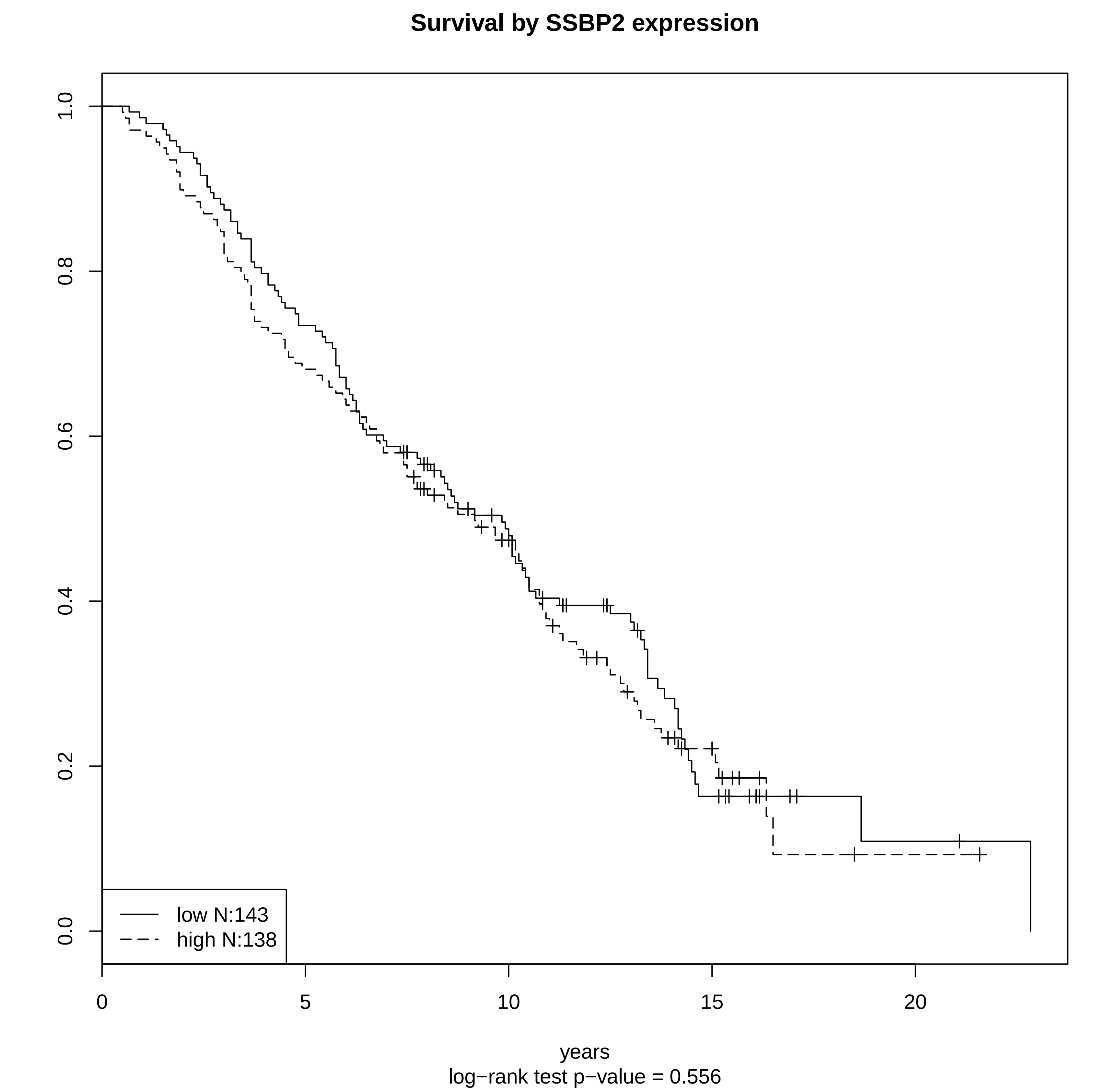

Survival by SSBP2 expression
1.0
0.8
0.6
0.4
0.2
0.0
0
5
10
15
20
years
log−rank test p−value = 0.556
low N:143
high N:138

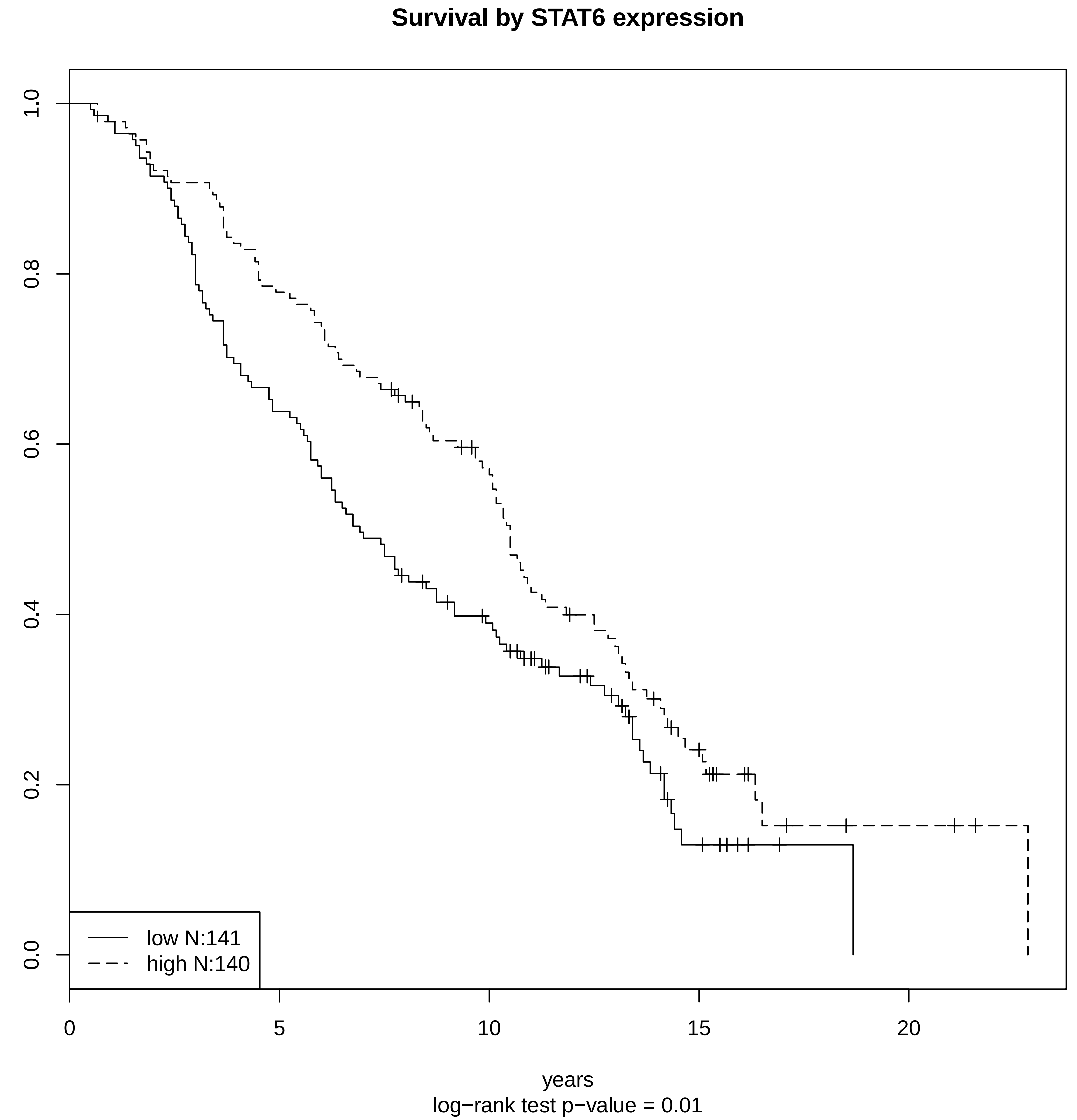

Survival by STAT6 expression
1.0
0.8
0.6
0.4
0.2
0.0
0
5
10
15
20
low N:141
high N:140
years
log−rank test p−value = 0.01

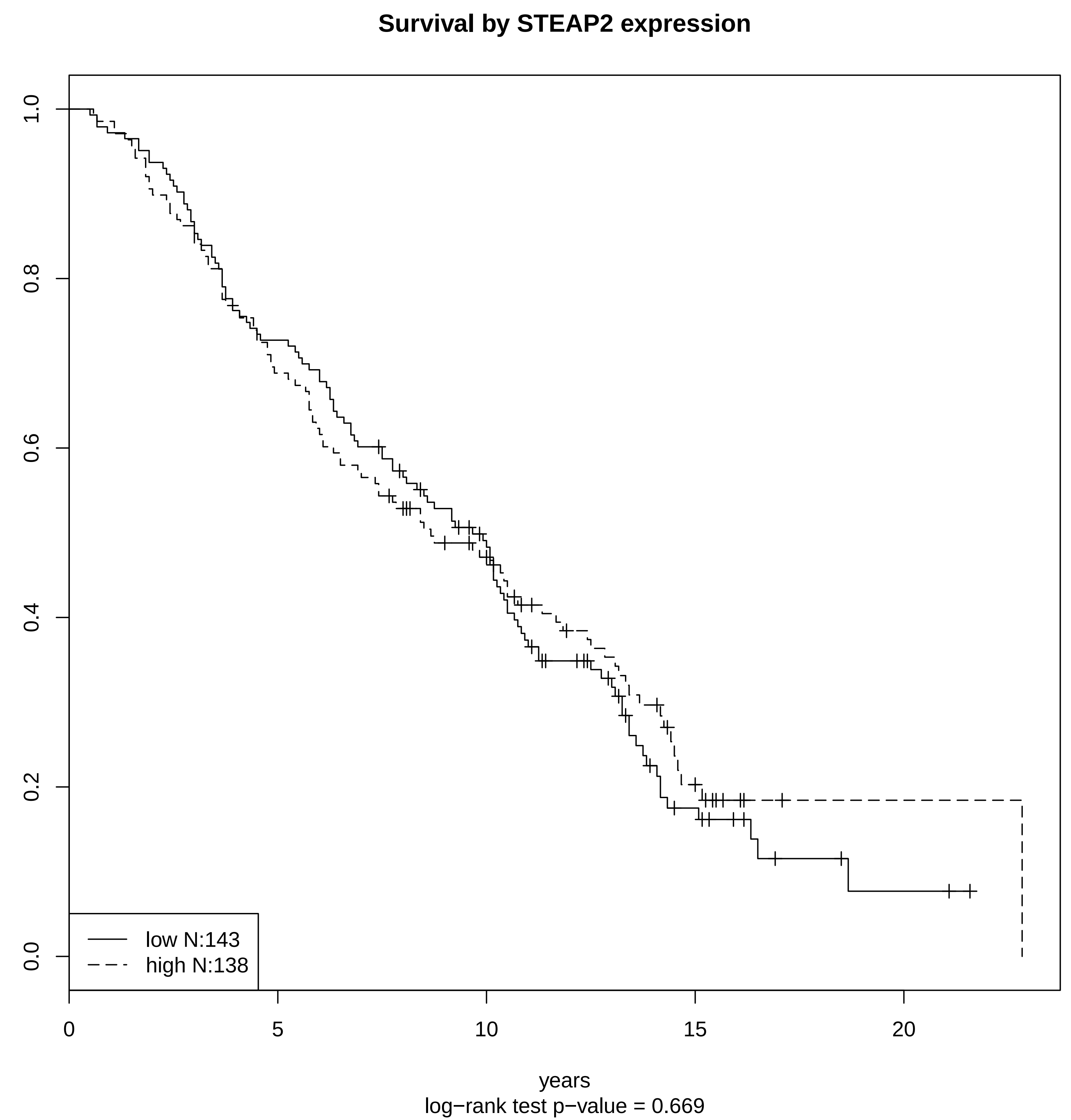
Survival by STEAP2 expression
1.0
0.8
0.6
0.4
0.2
0.0
0
5
10
15
20
years
log−rank test p−value = 0.669
low N:143
high N:138

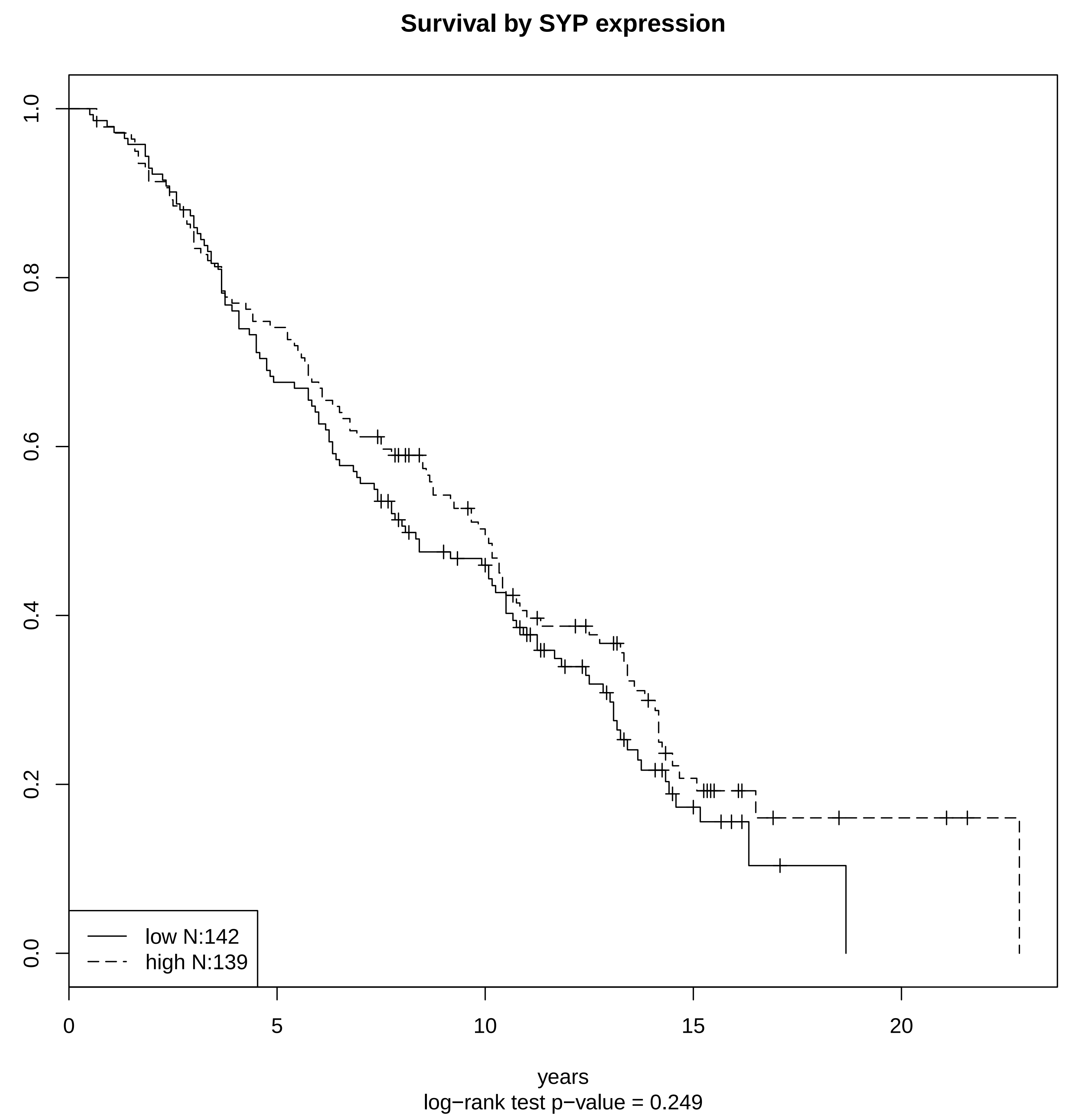

Survival by SYP expression
1.0
0.8
0.6
0.4
0.2
0.0
0
5
10
15
20
low N:142
high N:139
years
log−rank test p−value = 0.249

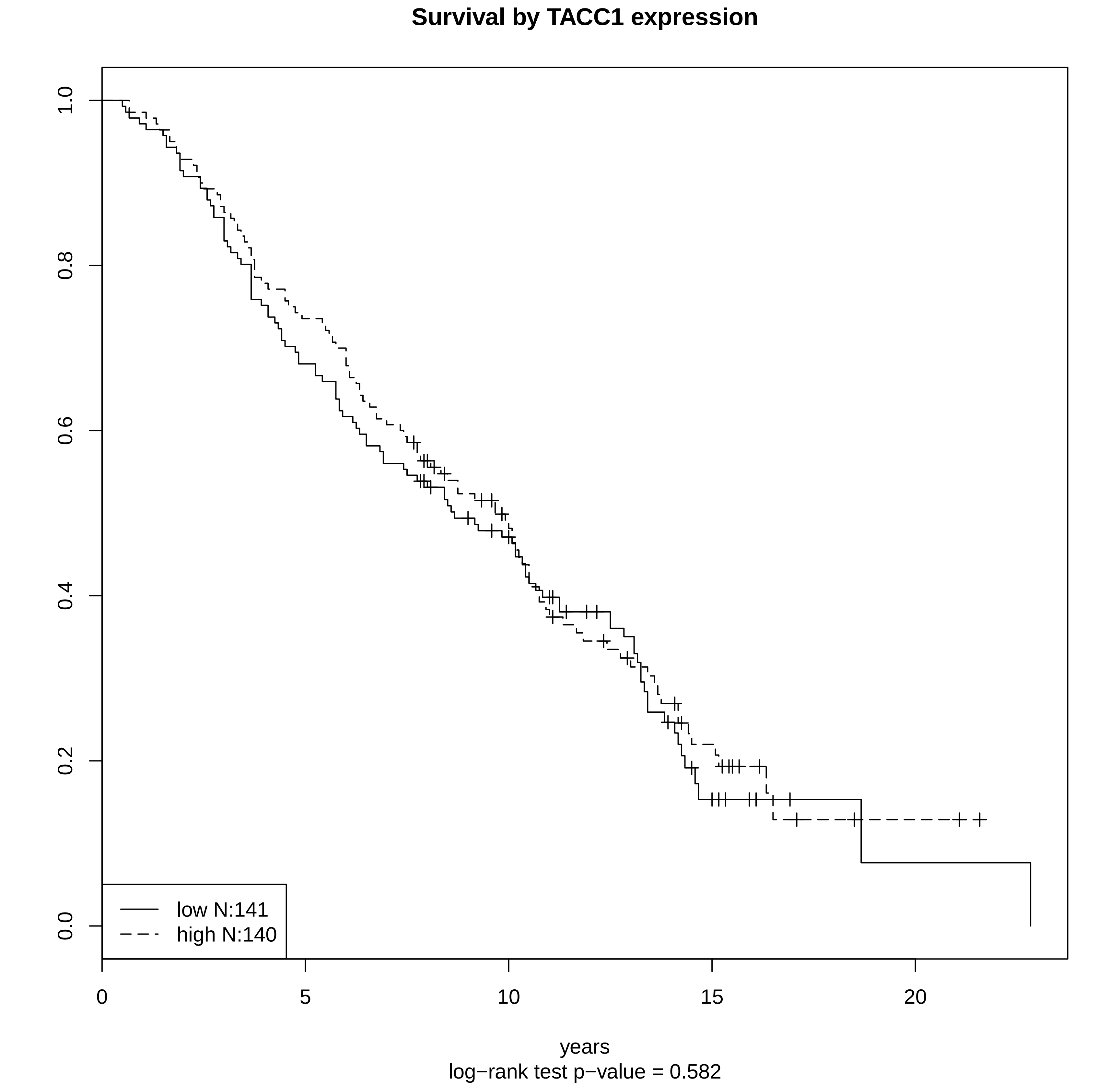
Survival by TACC1 expression
1.0
0.8
0.6
0.4
0.2
0.0
0
5
10
15
20
low N:141
high N:140
years
log−rank test p−value = 0.582

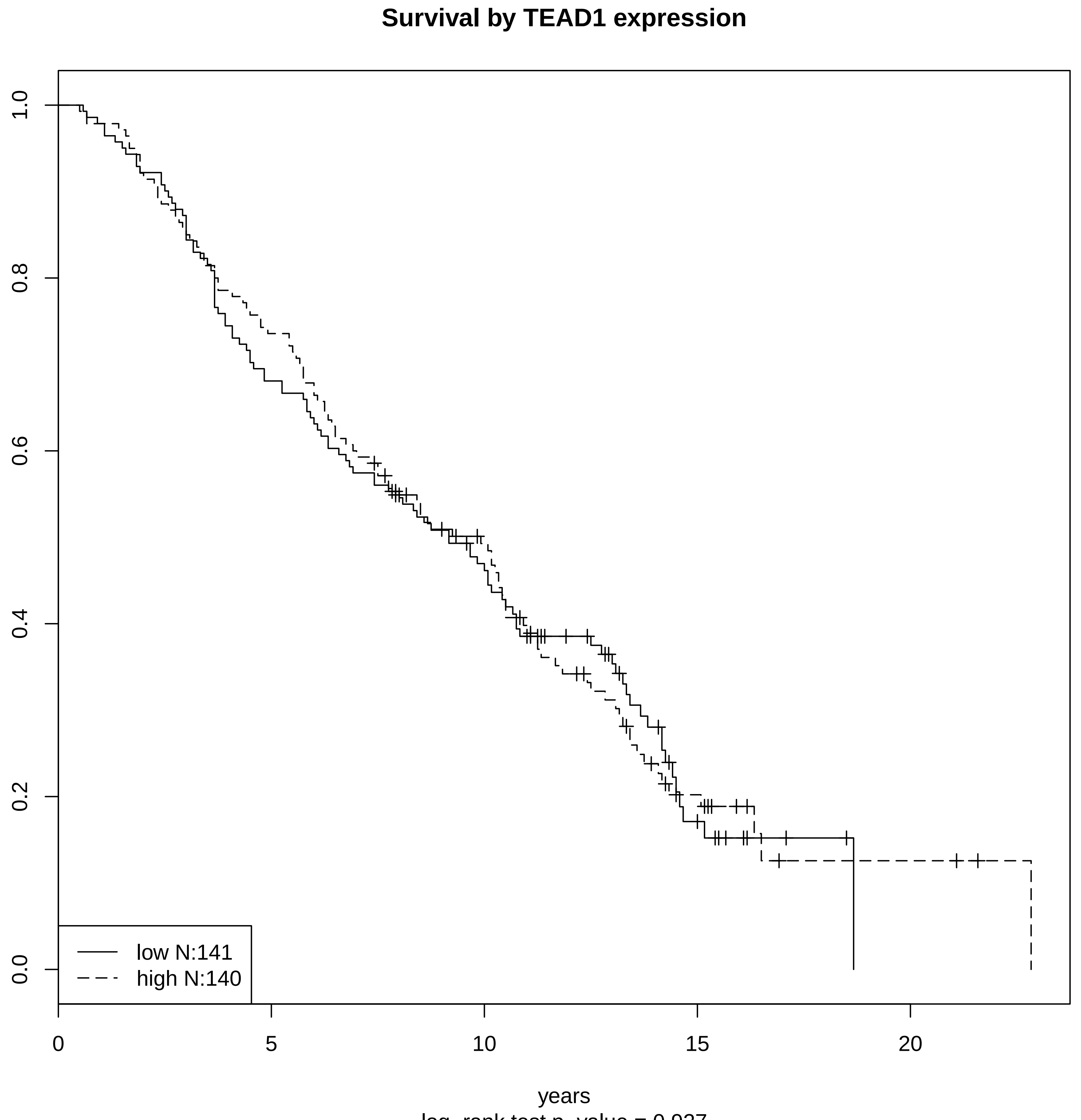

Survival by TEAD1 expression
1.0
0.8
0.6
0.4
0.2
0.0
0
5
10
15
20
low N:141
high N:140
years
log−rank test p−value = 0.927

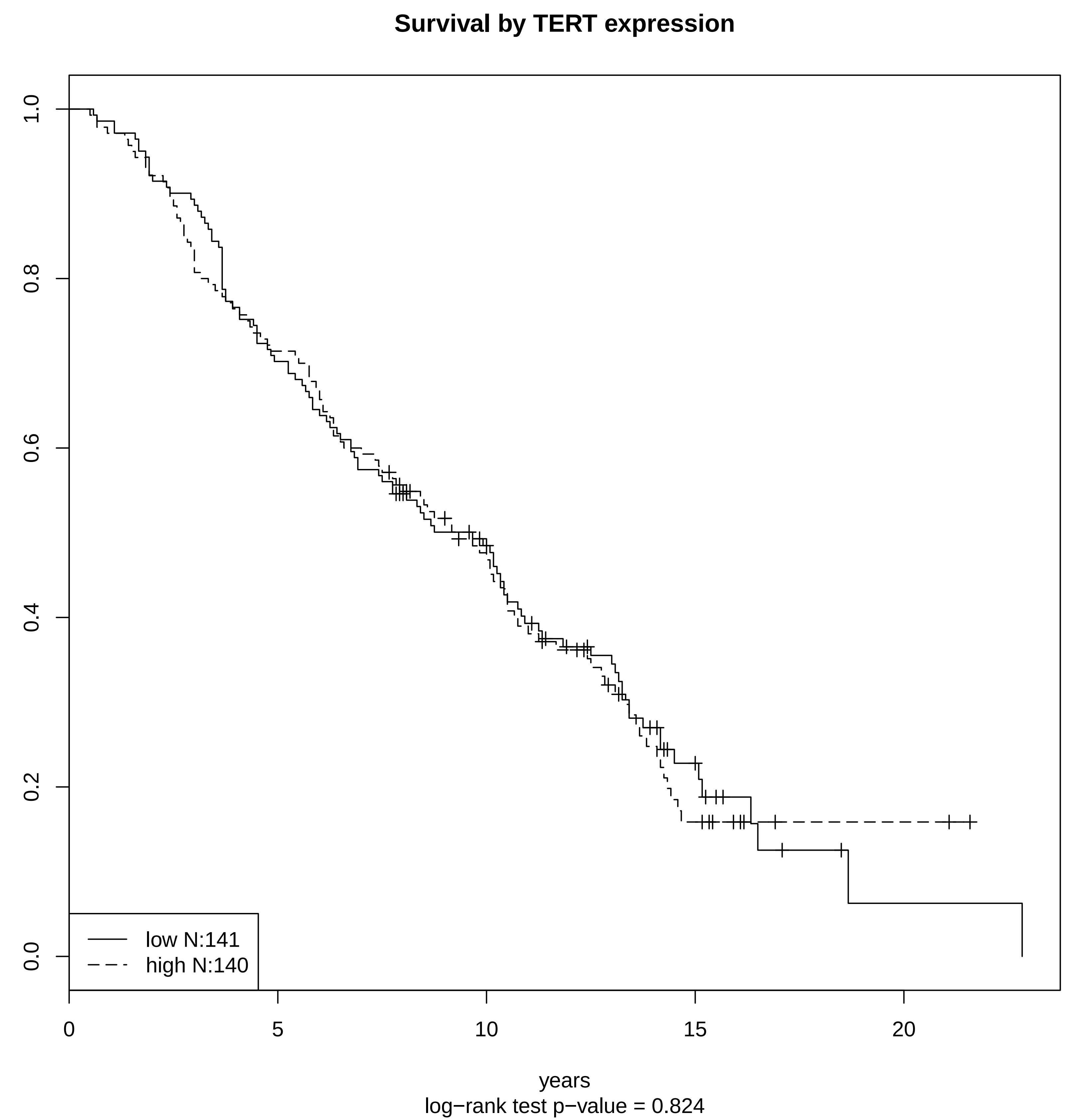

Survival by TERT expression
1.0
0.8
0.6
0.4
0.2
0.0
0
5
10
15
20
low N:141
high N:140
years
log−rank test p−value = 0.824

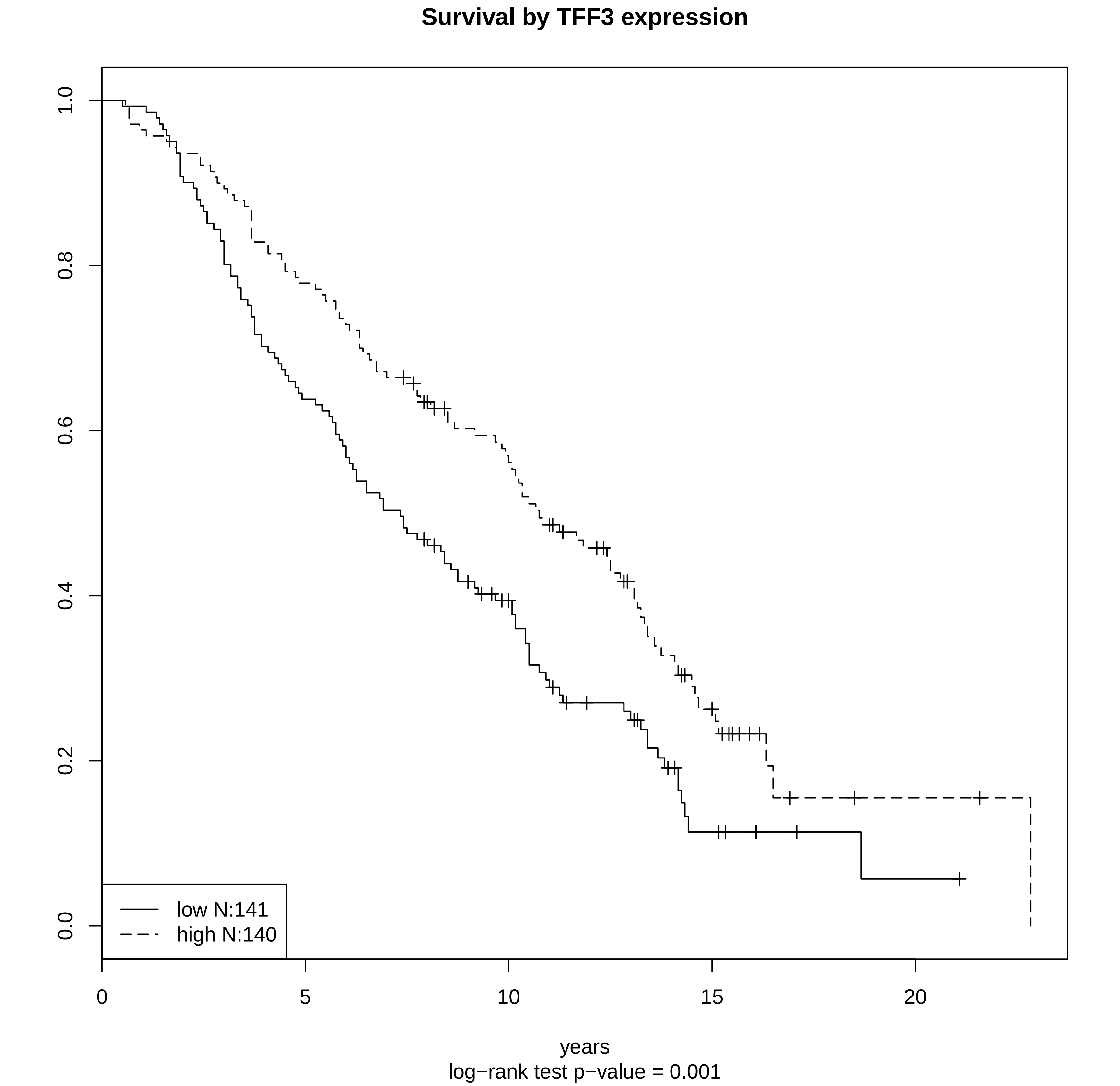
Survival by TFF3 expression
1.0
0.8
0.6
0.4
0.2
0.0
0
5
10
15
20
low N:141
high N:140
years
log−rank test p−value = 0.001

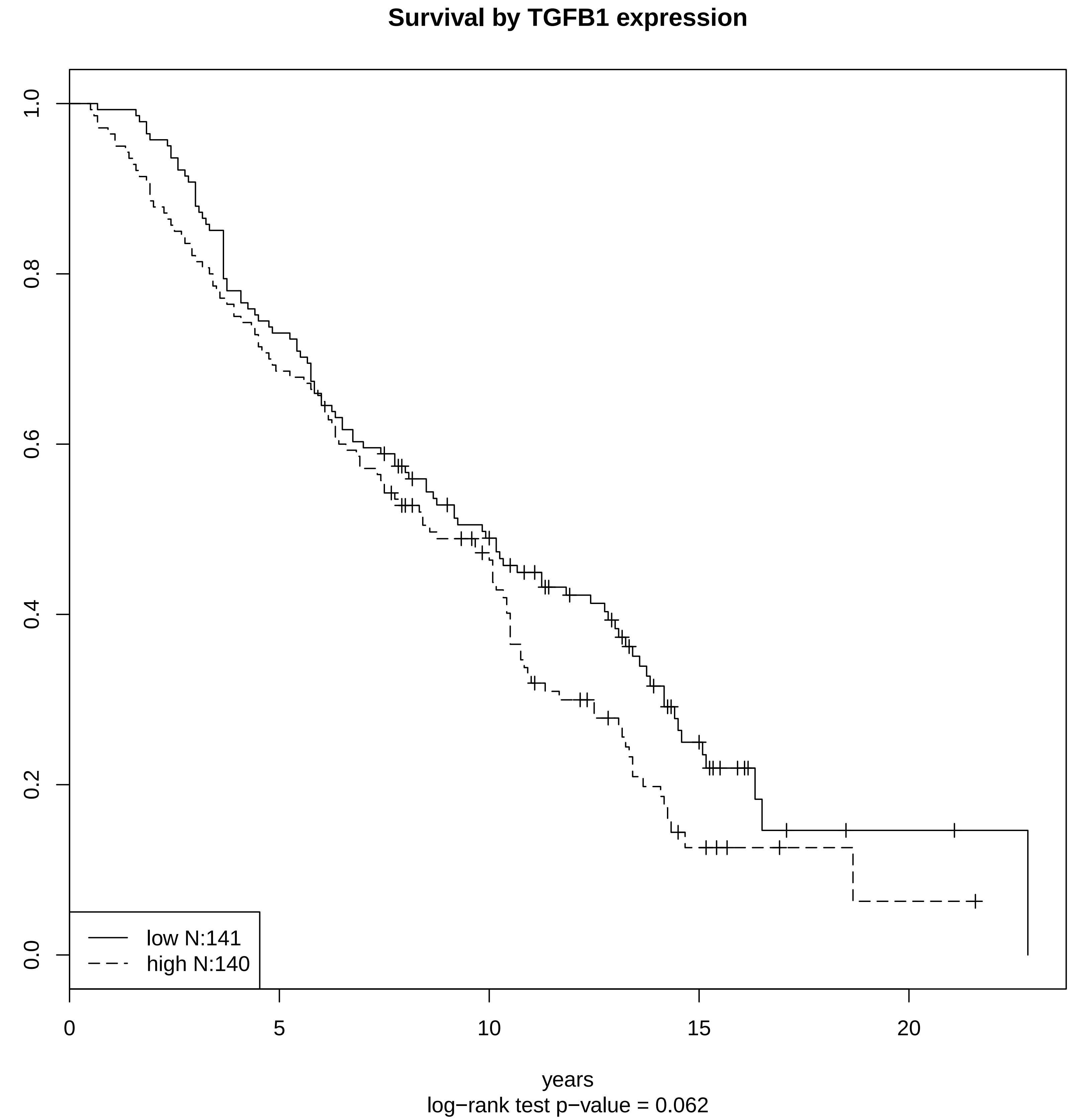
Survival by TGFB1 expression
1.0
0.8
0.6
0.4
0.2
0.0
0
5
10
15
20
years
low N:141
high N:140
log−rank test p−value = 0.062

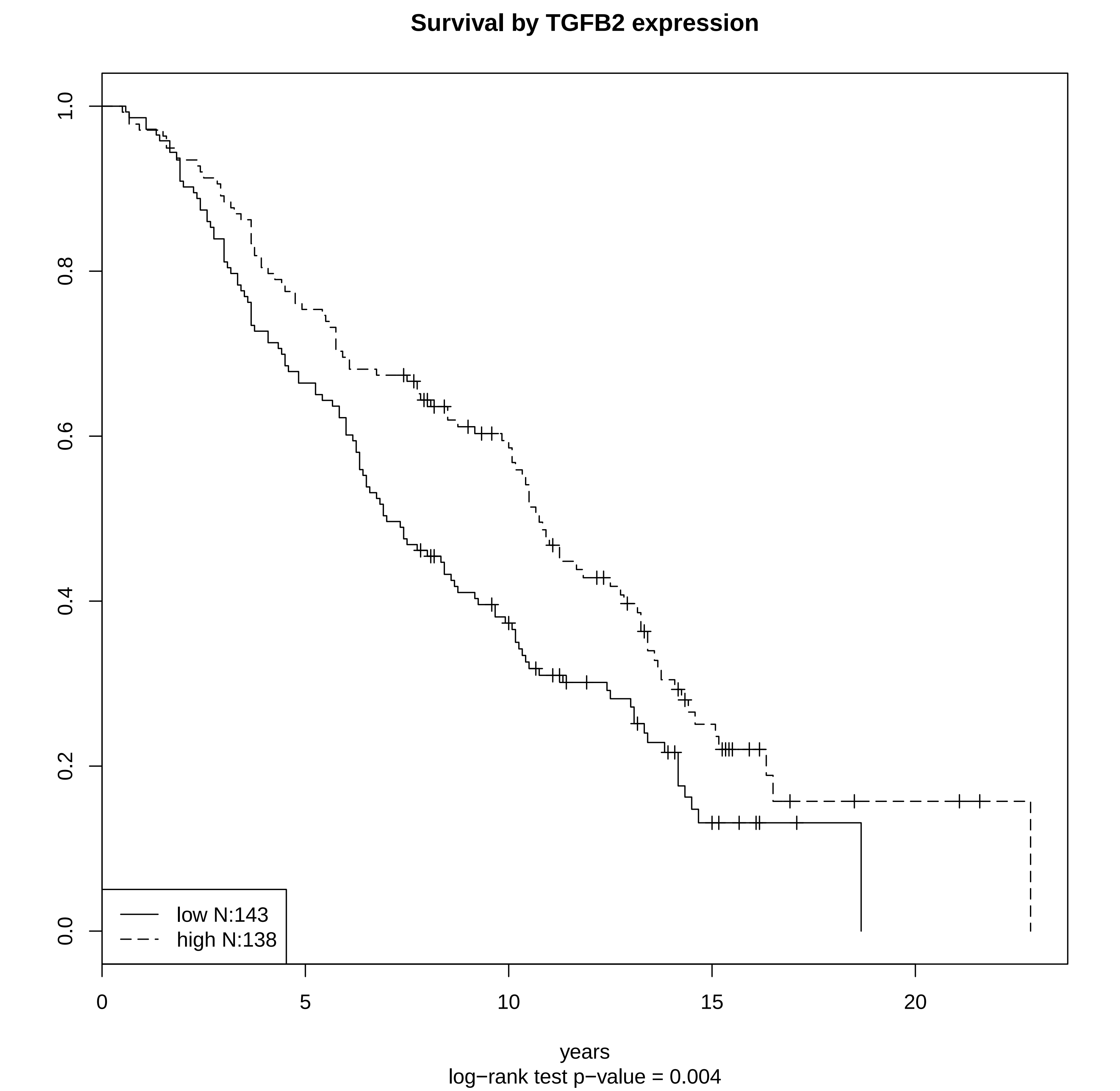

Survival by TGFB2 expression
1.0
0.8
0.6
0.4
0.2
0.0
0
5
10
15
20
years
log−rank test p−value = 0.004
low N:143
high N:138

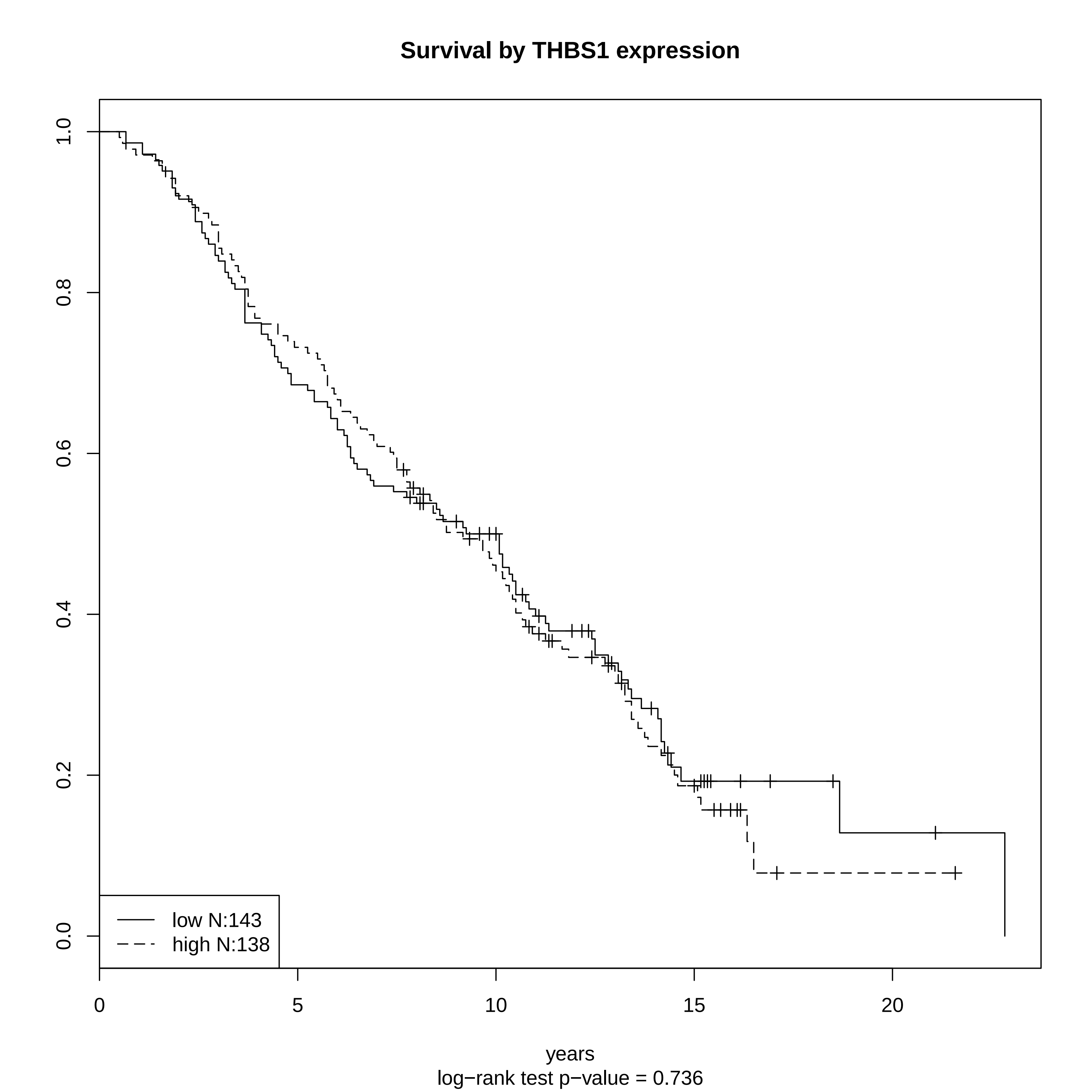
Survival by THBS1 expression
1.0
0.8
0.6
0.4
0.2
0.0
low N:143
high N:138
0
5
10
15
20
years
log−rank test p−value = 0.736

Survival by TIMP1 expression

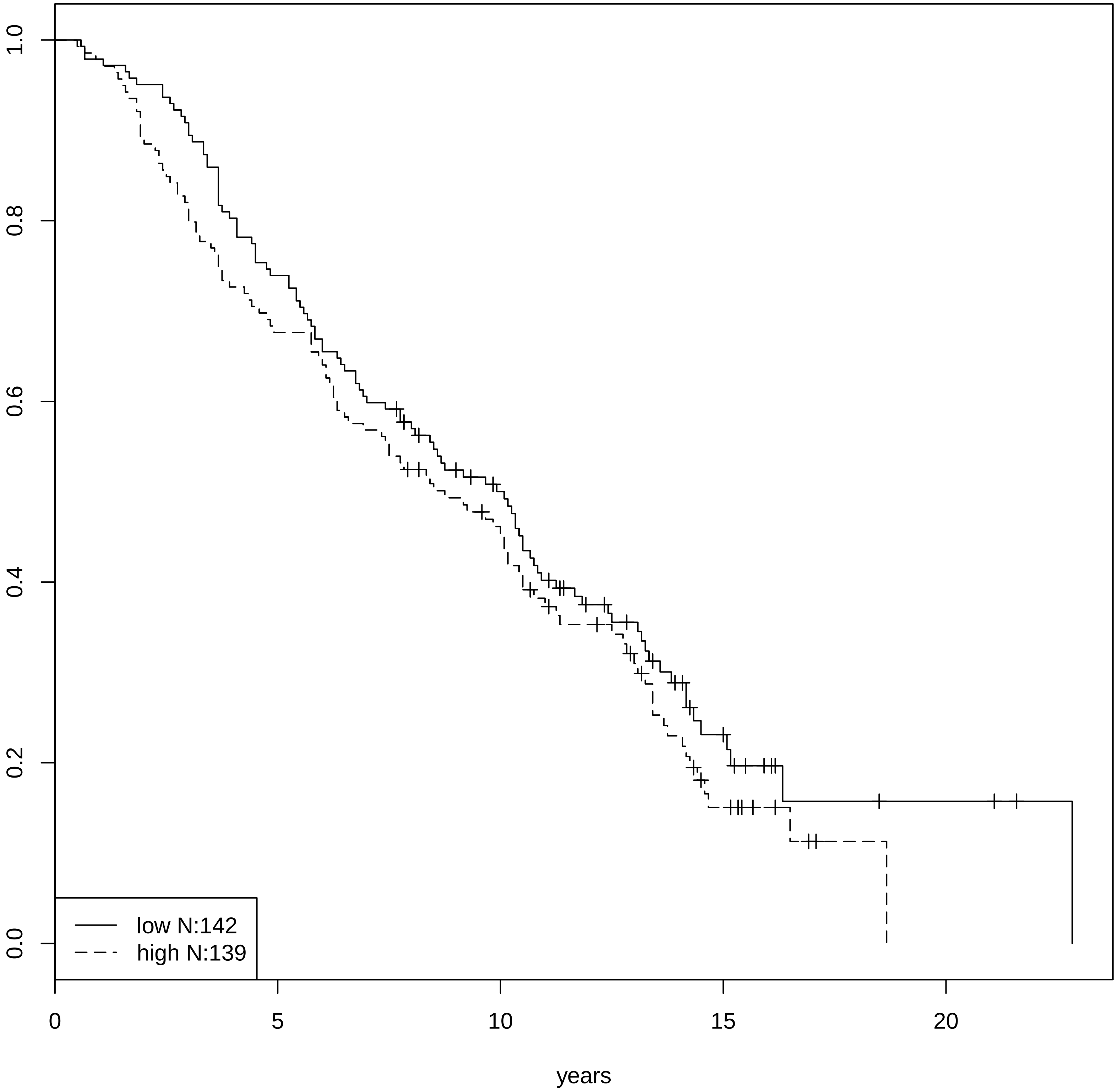

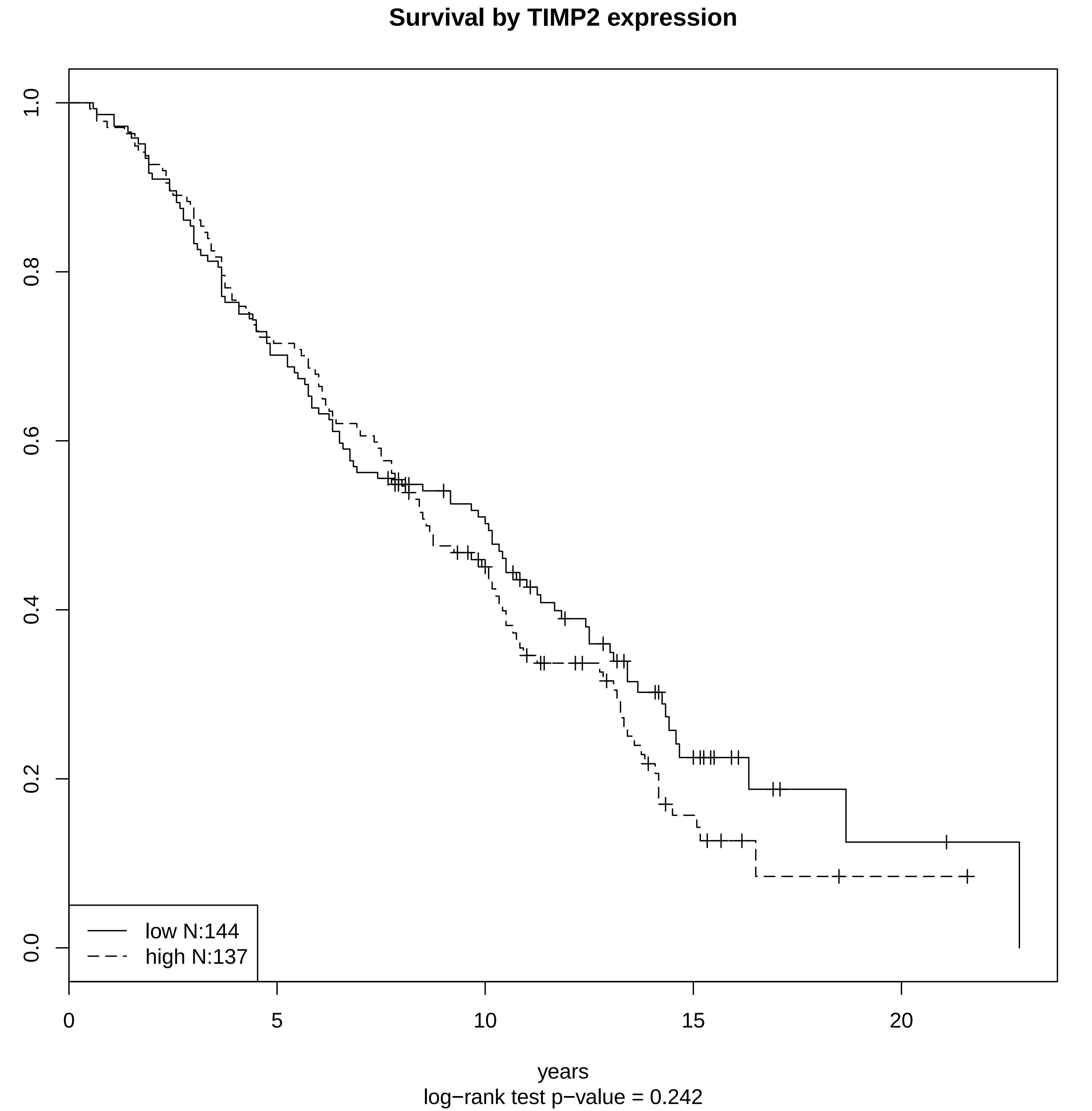
Survival by TIMP2 expression
1.0
0.8
0.6
0.4
0.2
0.0
0
5
10
15
20
low N:144
high N:137
years
log−rank test p−value = 0.242

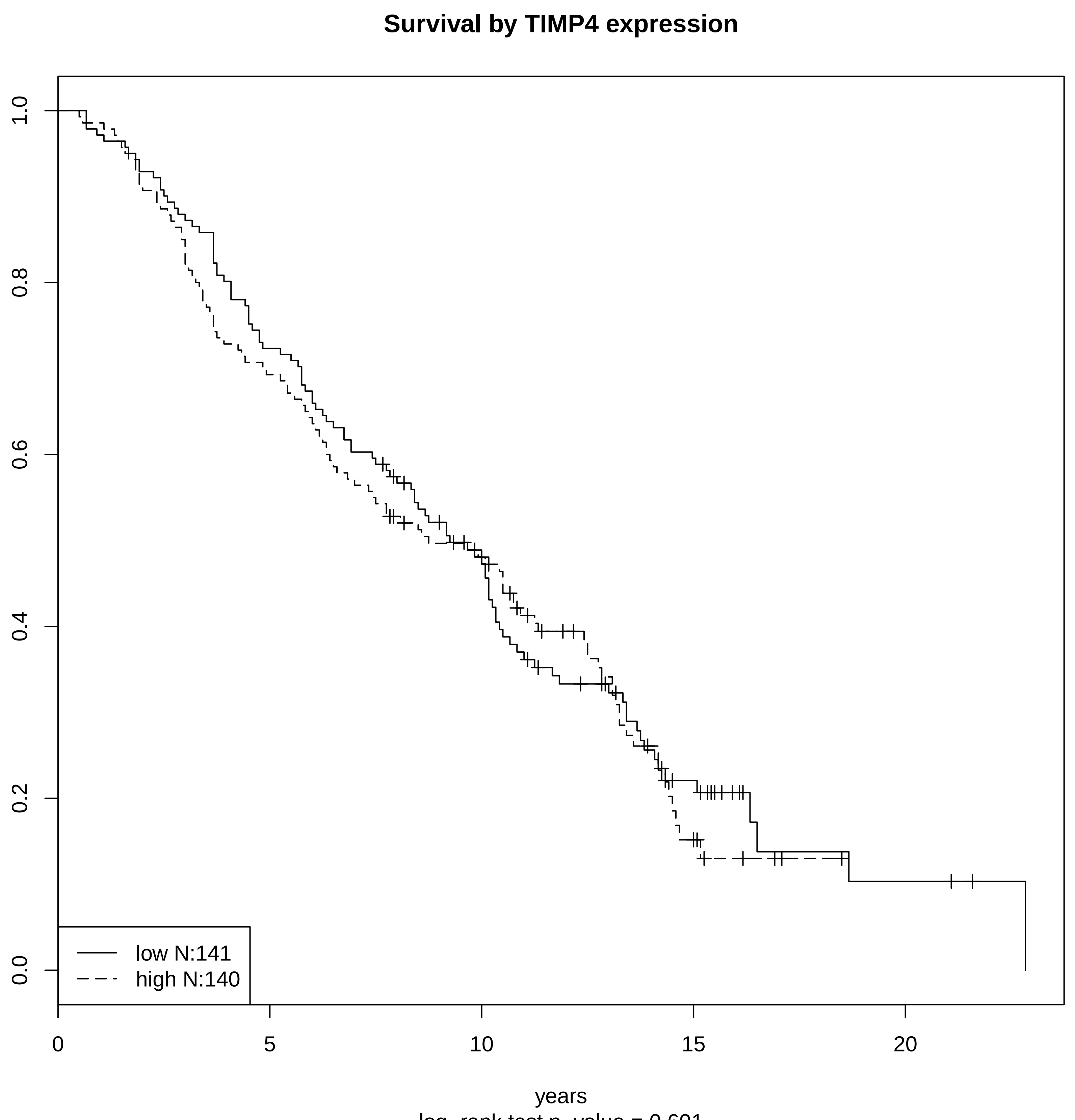
Survival by TIMP4 expression
1.0
0.8
0.6
0.4
0.2
0.0
0
5
10
15
20
years
log−rank test p−value = 0.691
low N:141
high N:140

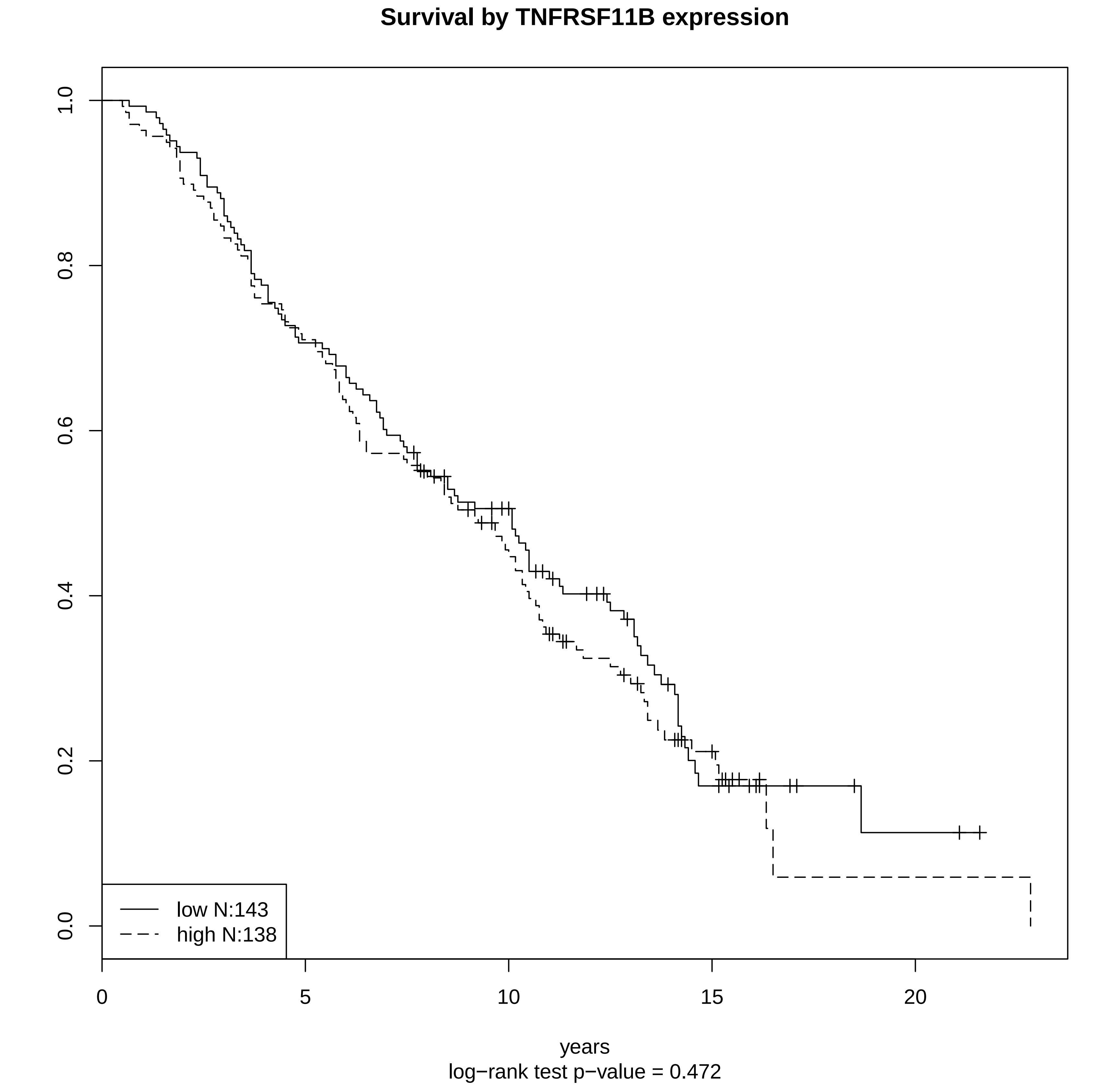
Survival by TNFRSF11B expression
1.0
0.8
0.6
0.4
0.2
0.0
0
5
10
15
20
low N:143
high N:138
years
log−rank test p−value = 0.472

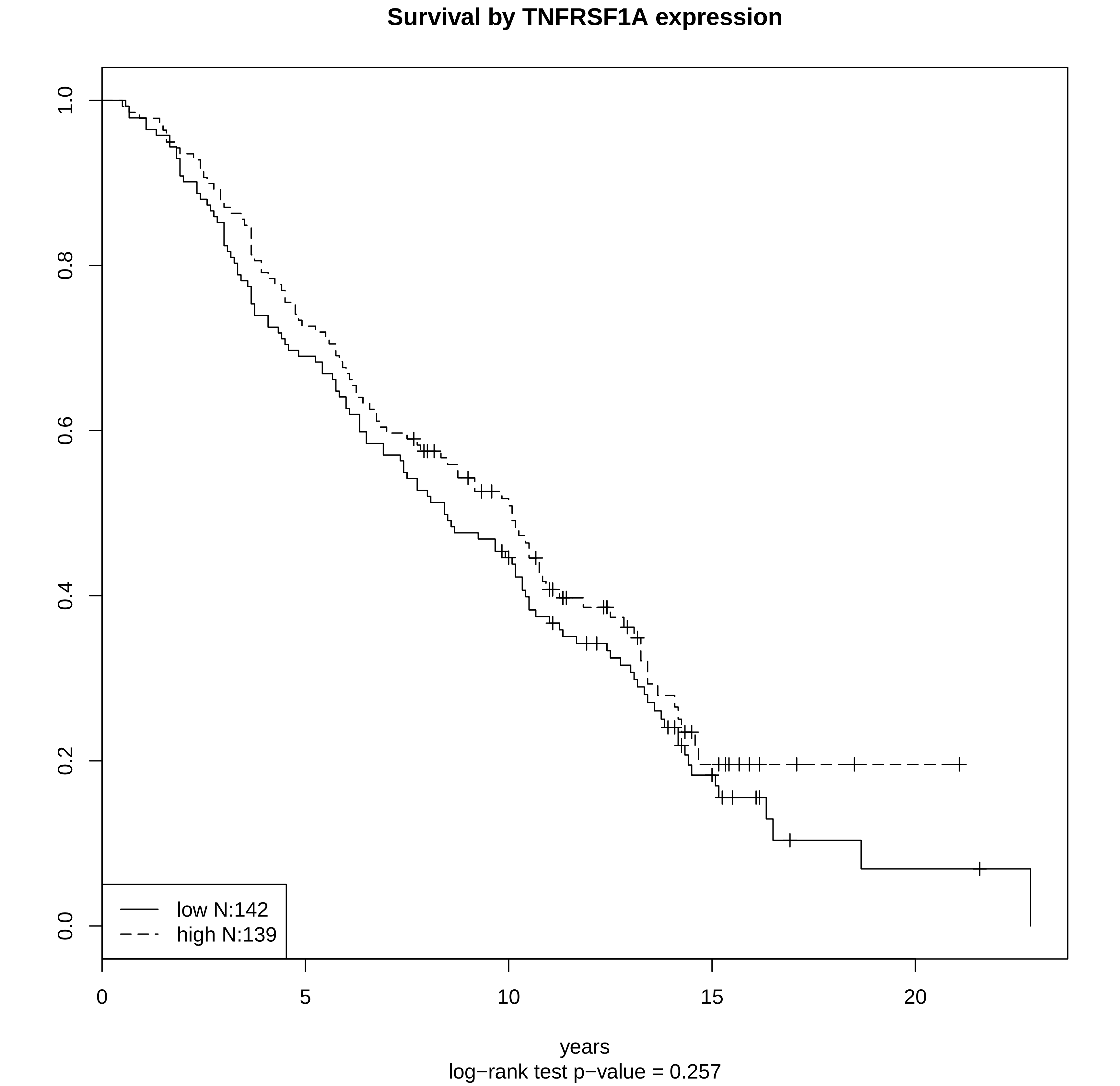
Survival by TNFRSF1A expression
0.0
0.2
0.4
0.6
0.8
1.0
0
5
10
15
20
low N:142
high N:139
years
log−rank test p−value = 0.257

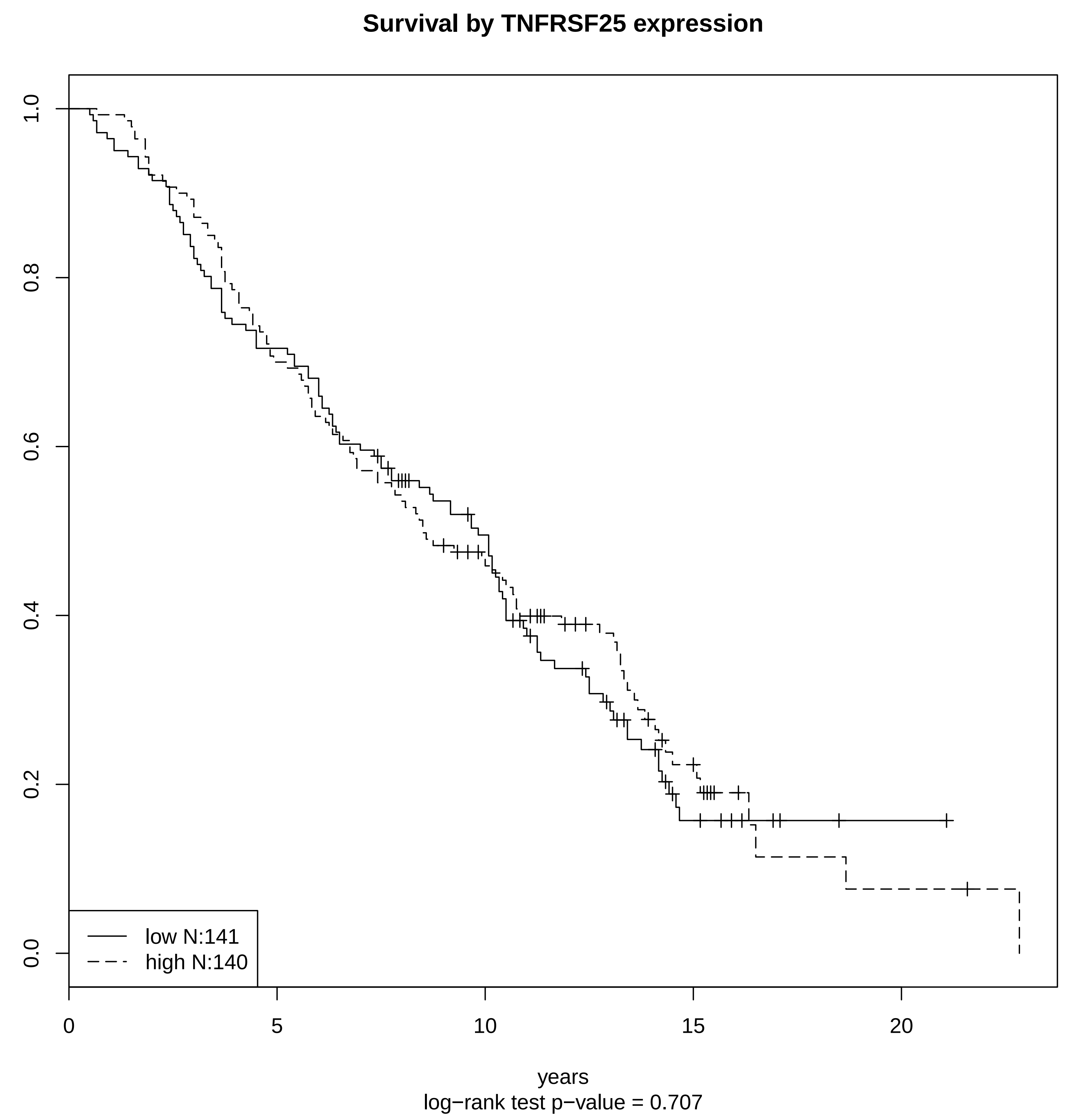

Survival by TNFRSF25 expression
1.0
0.8
0.6
0.4
0.2
0.0
0
5
10
15
20
years
log−rank test p−value = 0.707
low N:141
high N:140

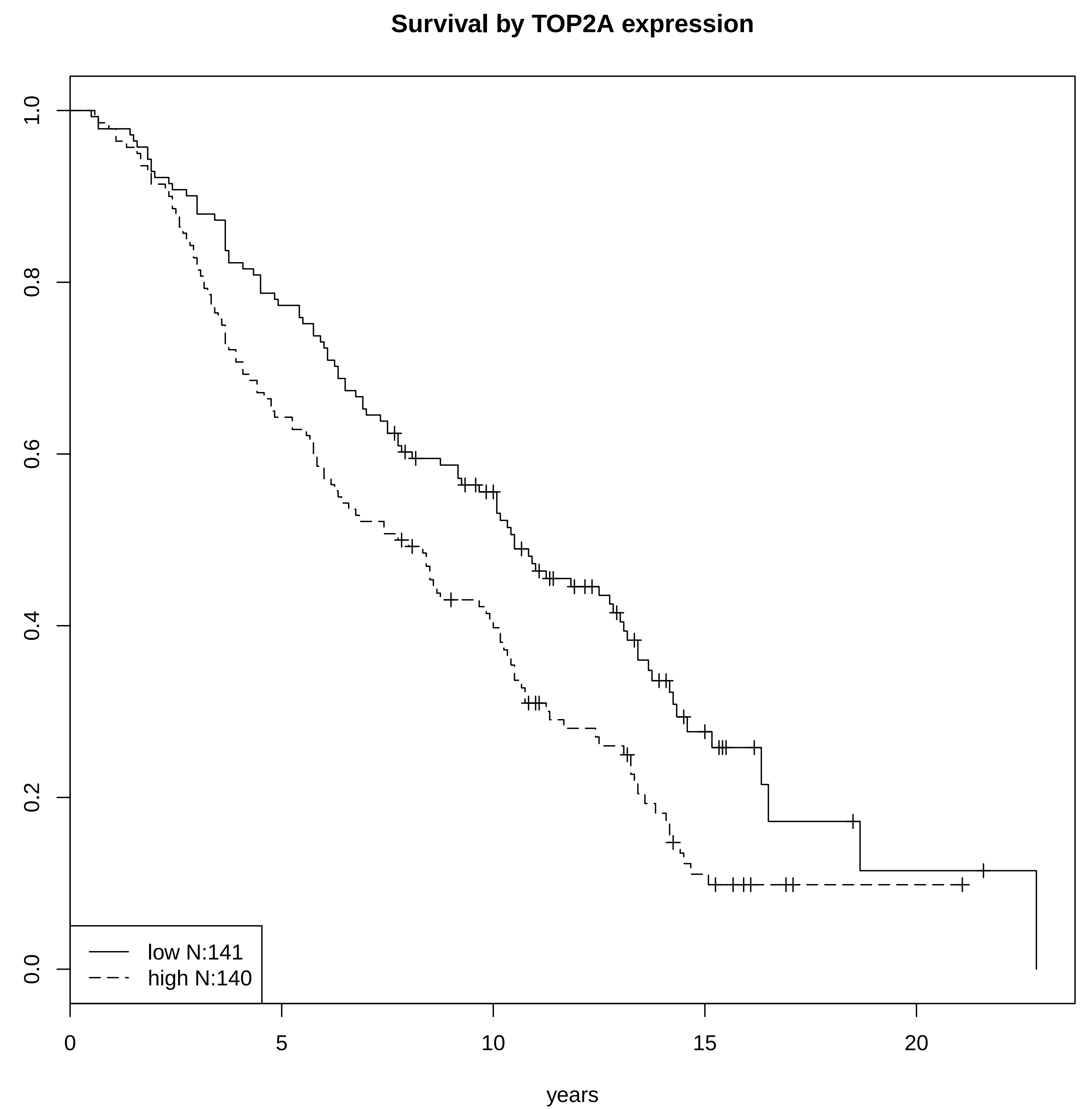
Survival by TOP2A expression
1.0
0.8
0.6
0.4
0.2
0.0
0
5
10
15
20
low N:141
high N:140
years
log−rank test p−value = 0.002

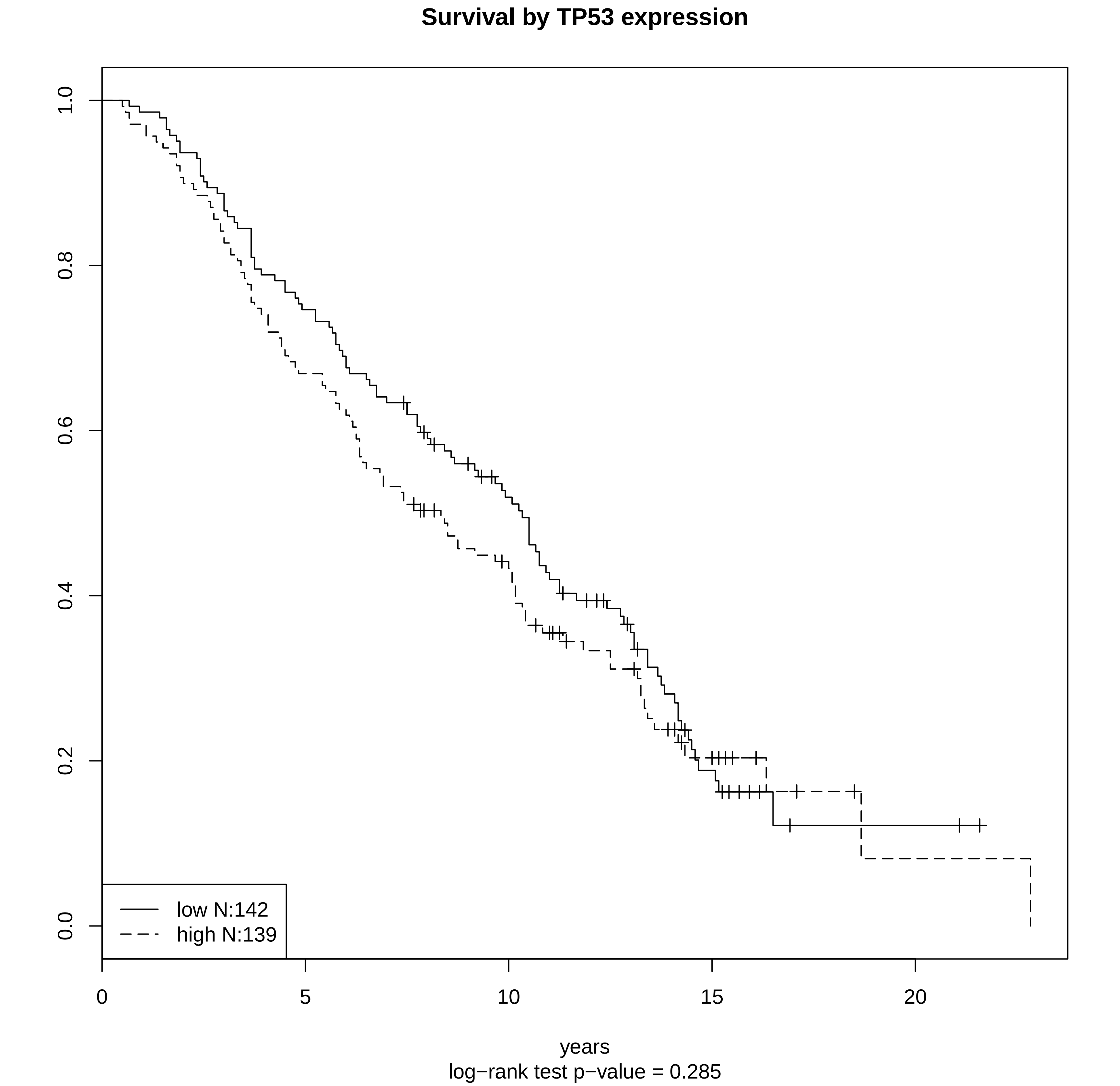

Survival by TP53 expression
1.0
0.8
0.6
0.4
0.2
0.0
0
5
10
15
20
low N:142
high N:139
years
log−rank test p−value = 0.285

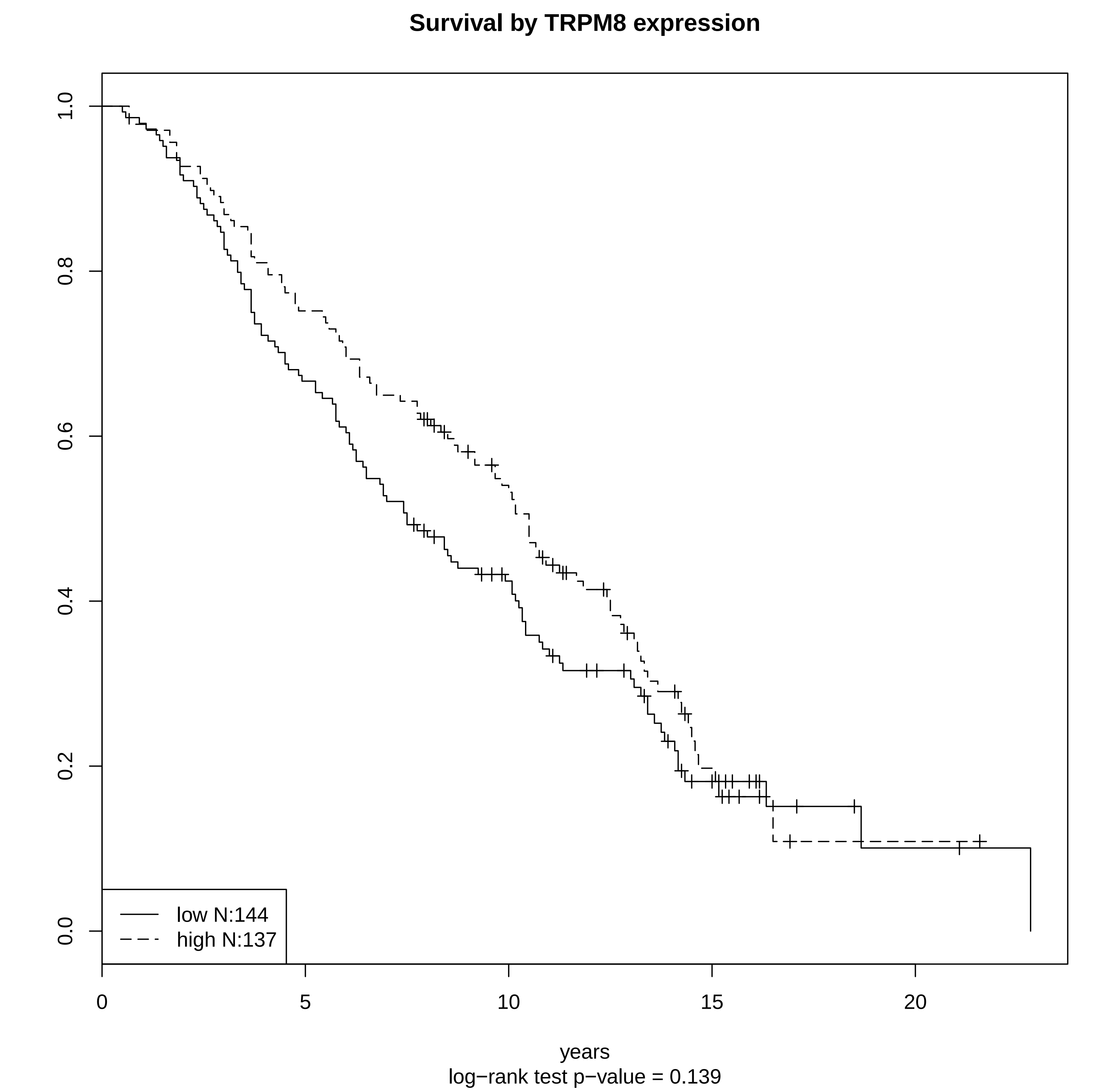
Survival by TRPM8 expression
1.0
0.8
0.6
0.4
0.2
0.0
0
5
10
15
20
low N:144
high N:137
years
log−rank test p−value = 0.139

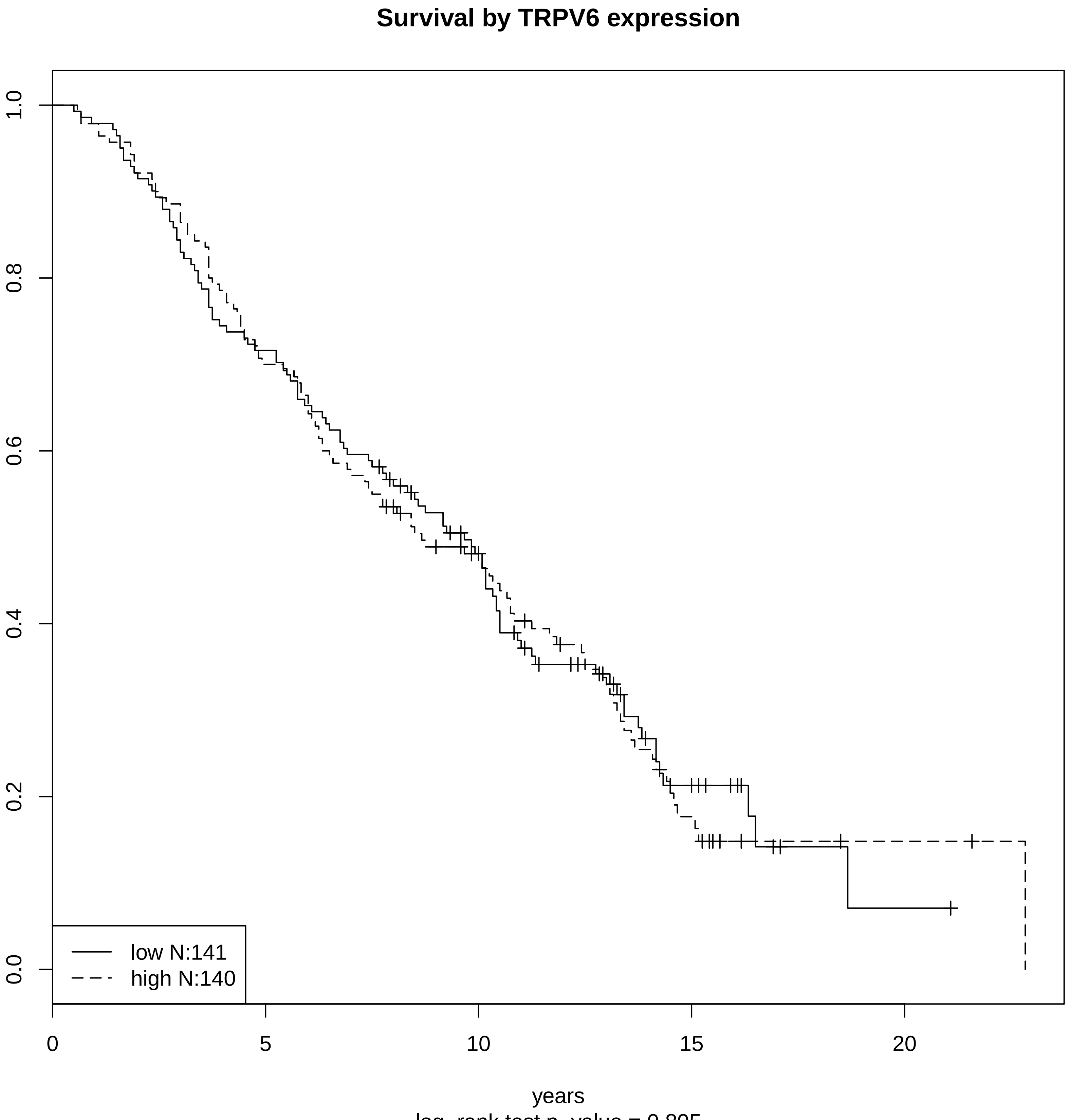

Survival by TRPV6 expression
1.0
0.8
0.6
0.4
0.2
0.0
0
5
10
15
20
years
log−rank test p−value = 0.895
low N:141
high N:140

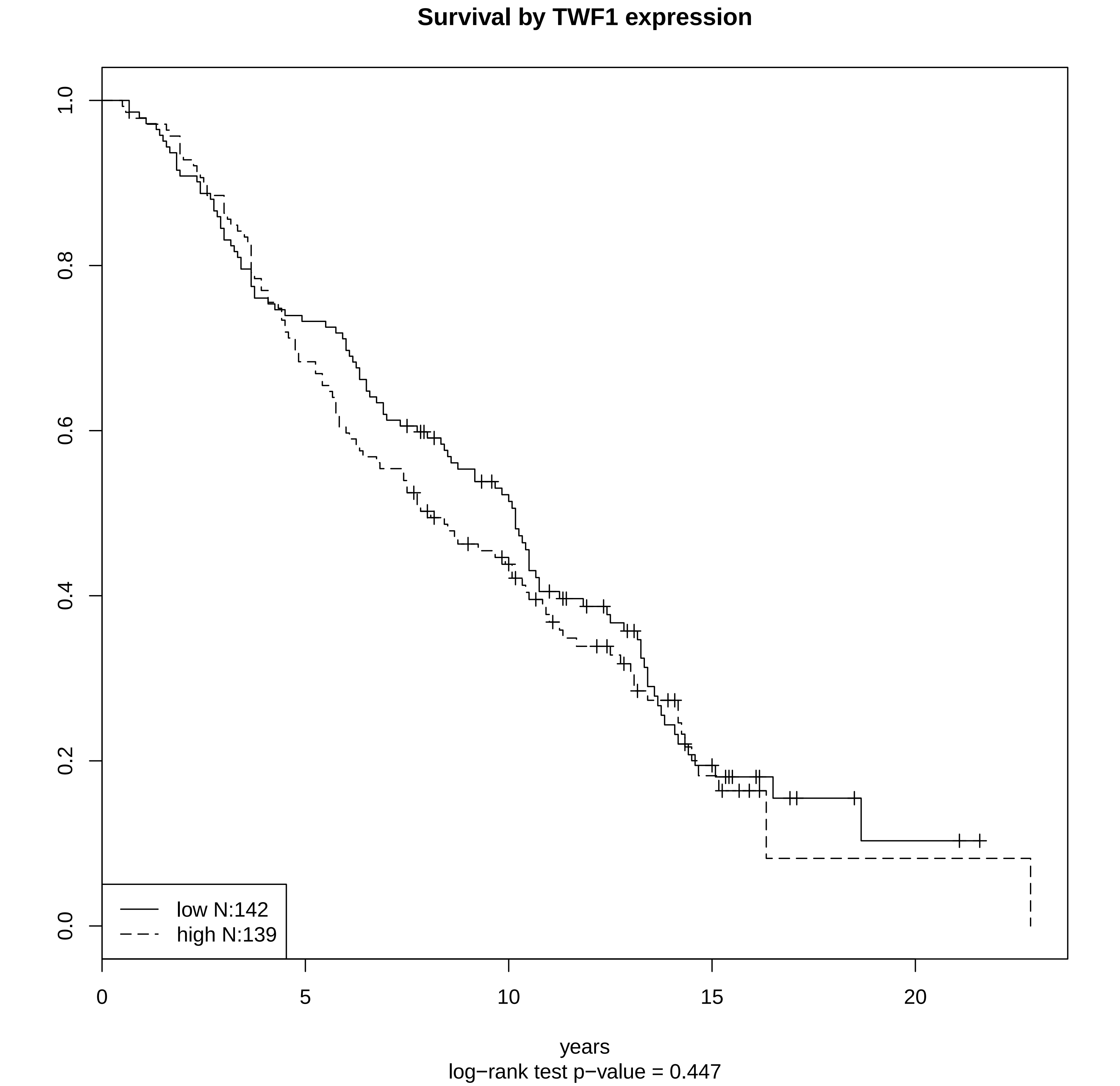

Survival by TWF1 expression
1.0
0.8
0.6
0.4
0.2
0.0
0
5
10
15
20
low N:142
high N:139
years
log−rank test p−value = 0.447

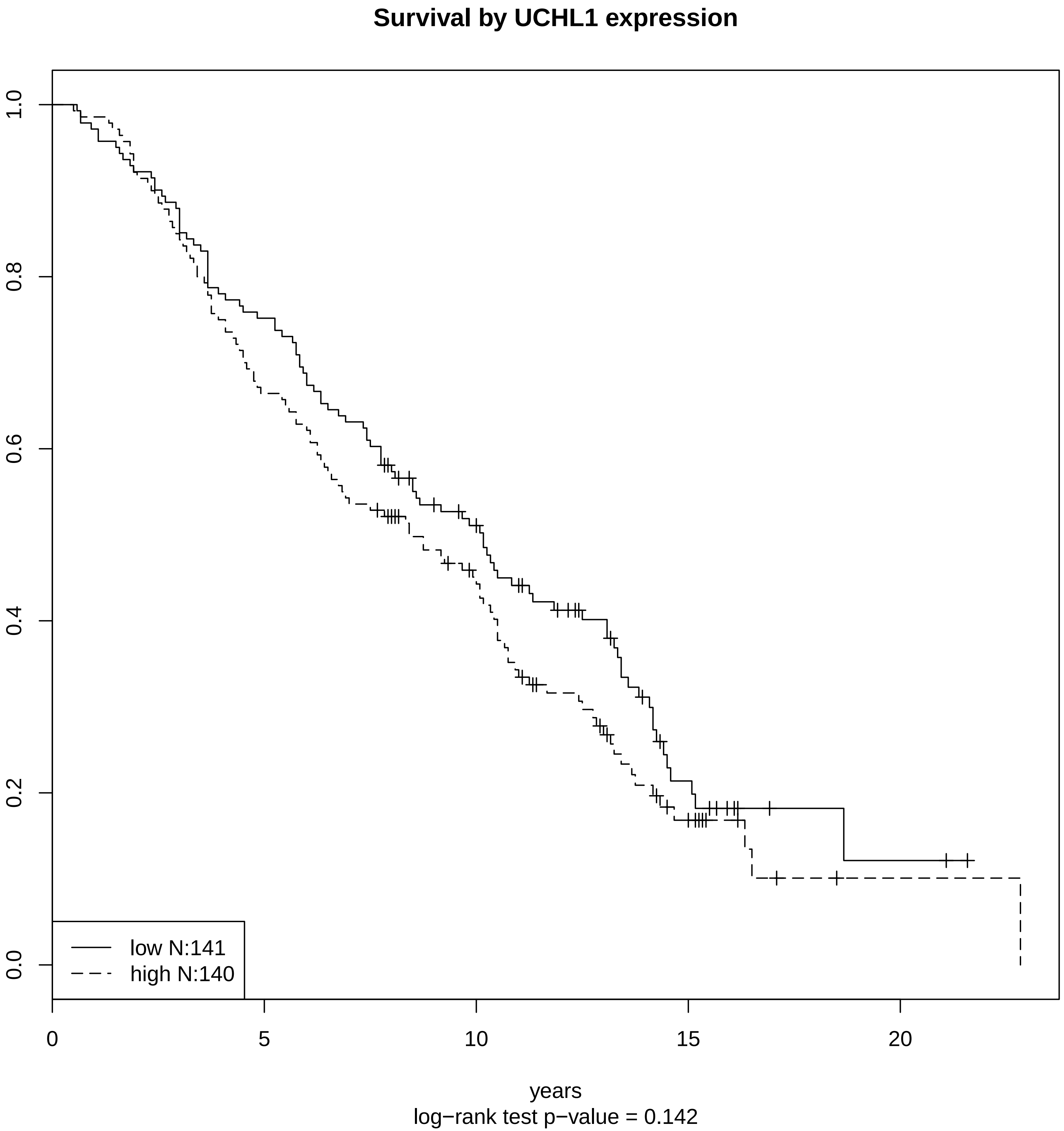
Survival by UCHL1 expression
1.0
0.8
0.6
0.4
0.2
0.0
0
5
10
15
20
years
log−rank test p−value = 0.142
low N:141
high N:140

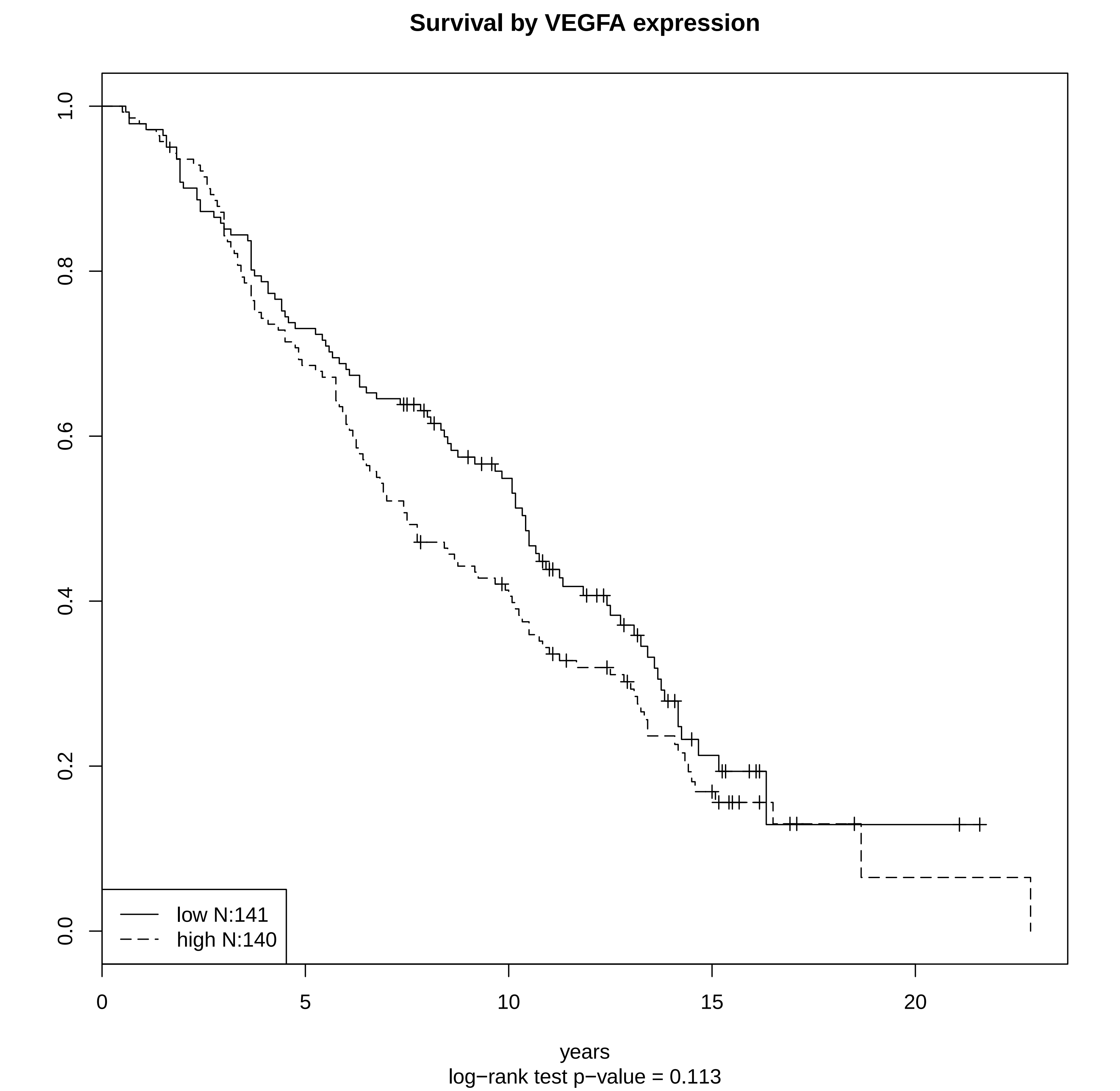

Survival by VEGFA expression
1.0
0.8
0.6
0.4
0.2
0.0
0
5
10
15
20
low N:141
high N:140
years
log−rank test p−value = 0.113

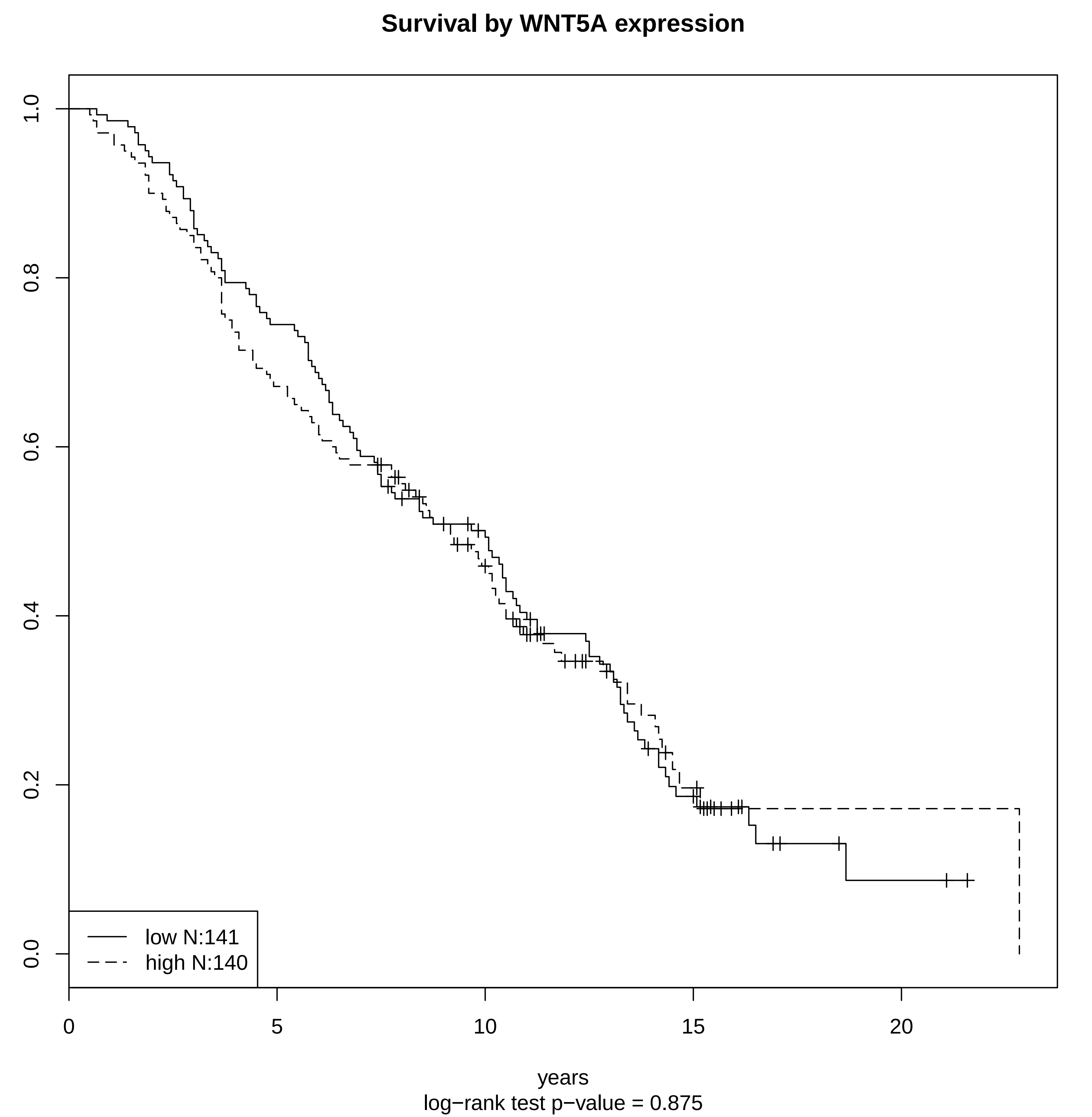

Survival by WNT5A expression
1.0
0.8
0.6
0.4
0.2
0.0
0
5
10
15
20
years
log−rank test p−value = 0.875
low N:141
high N:140

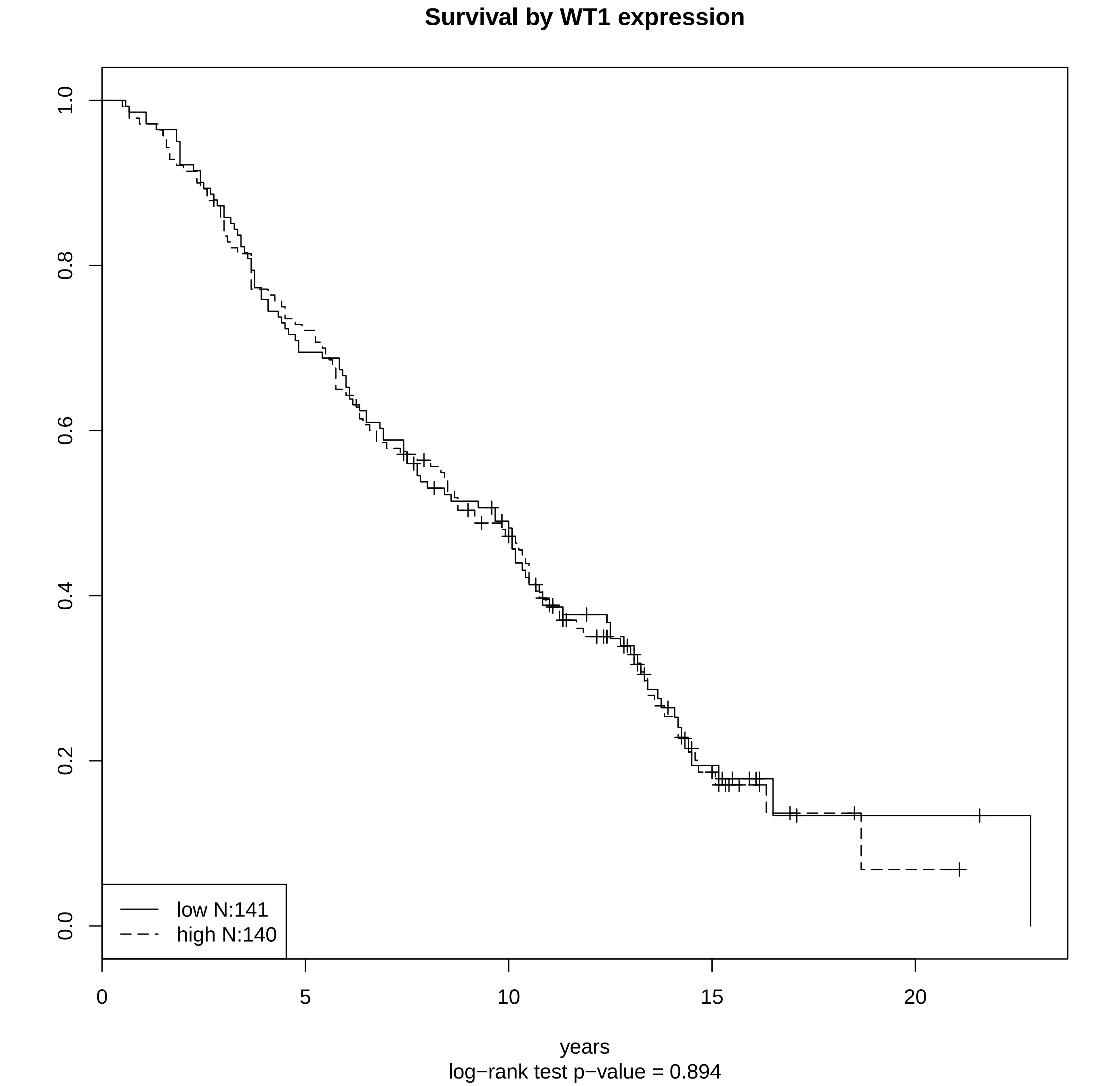

Survival by WT1 expression
1.0
0.8
0.6
0.4
0.2
0.0
0
5
10
15
20
low N:141
high N:140
years
log−rank test p−value = 0.894

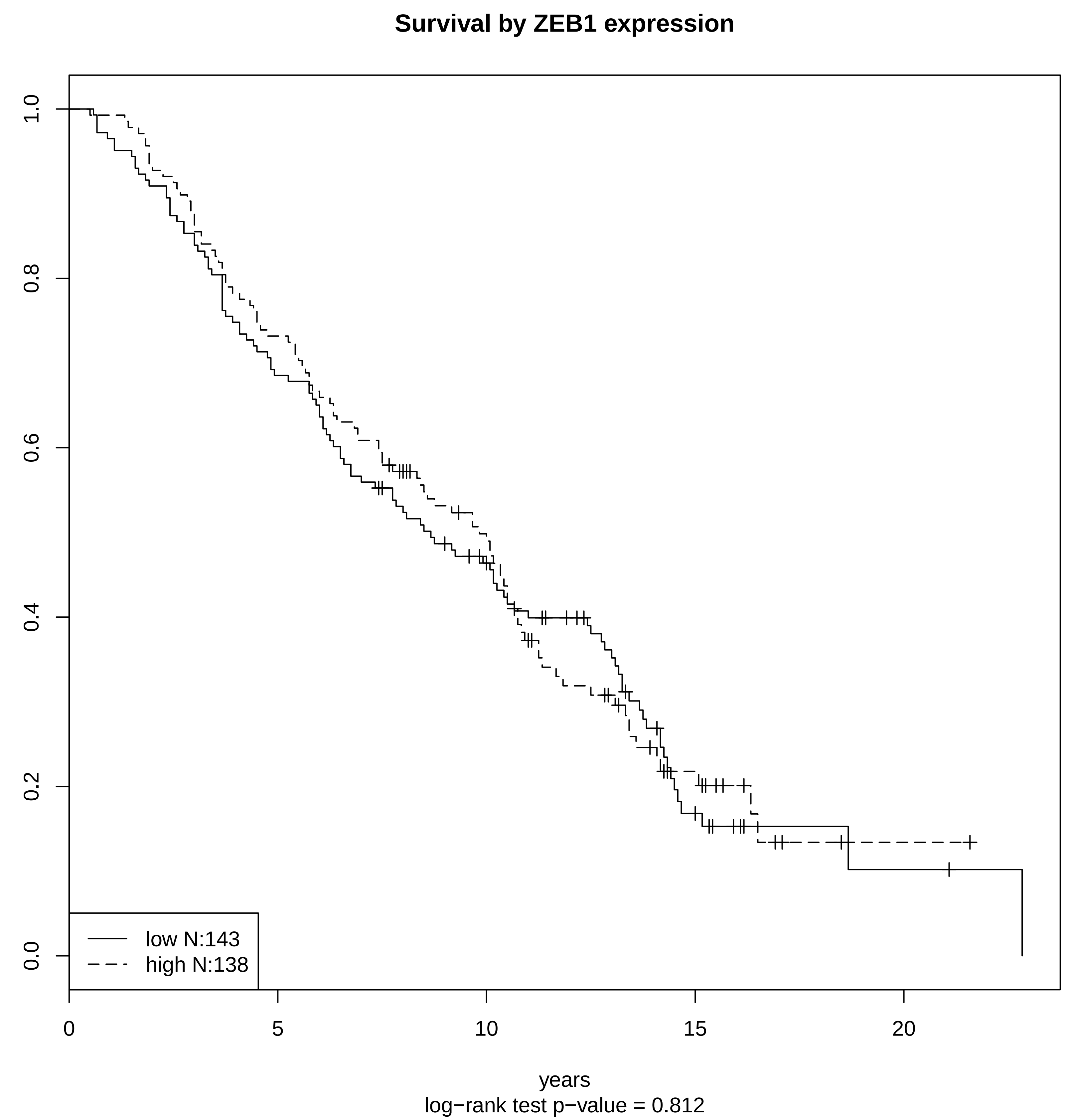
Survival by ZEB1 expression
1.0
0.8
0.6
0.4
0.2
0.0
0
5
10
15
20
low N:143
high N:138
years
log−rank test p−value = 0.812

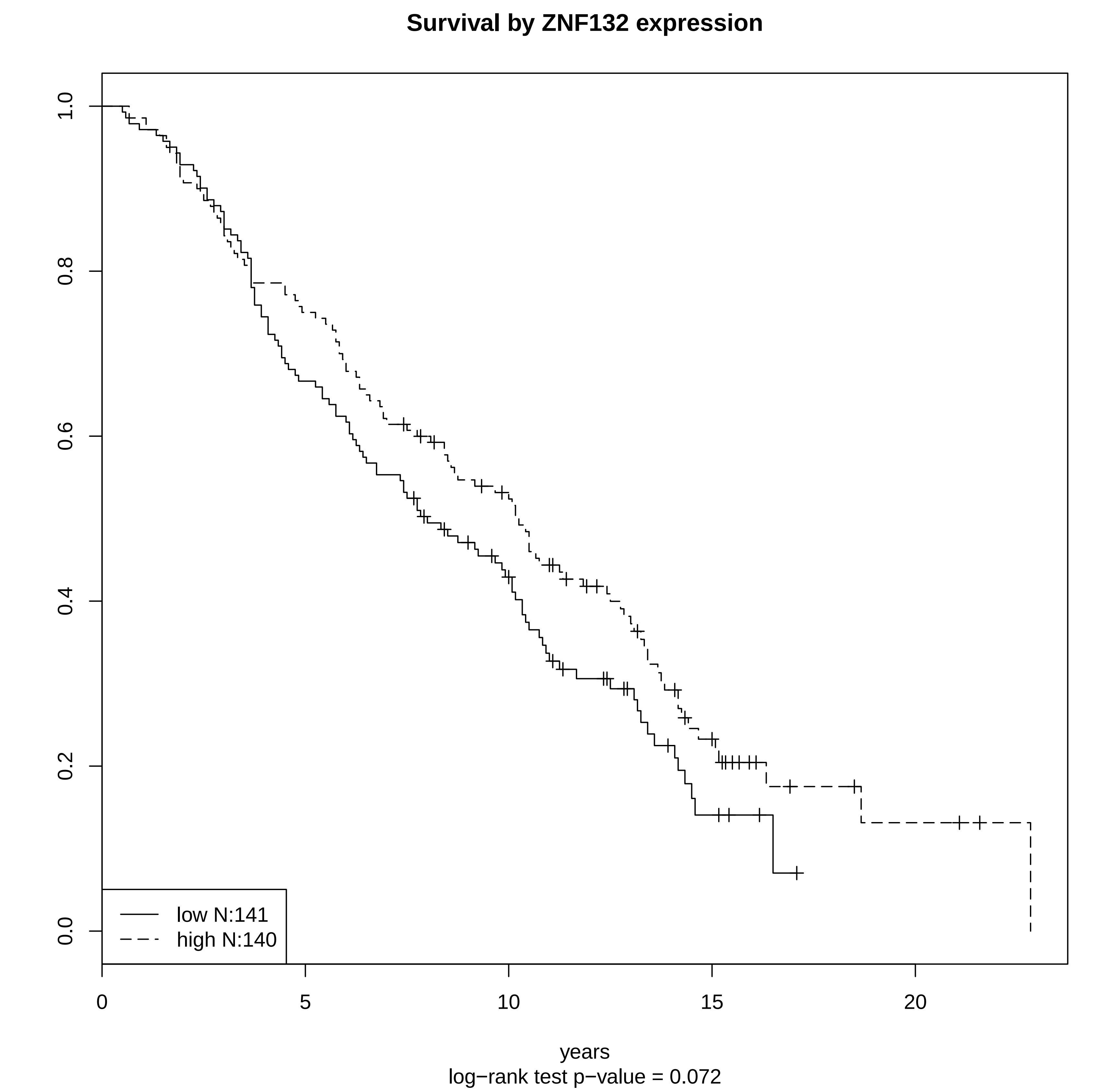
Survival by ZNF132 expression
1.0
0.8
0.6
0.4
0.2
0.0
0
5
10
15
20
years
log−rank test p−value = 0.072
low N:141
high N:140
