# Supplementary figures and images for: Validation of proposed prostate cancer biomarkers with gene expression data: a long road to travel
Source: Cancer Metastasis Rev. 2014 Jan 30;33(2):657–71. doi: 10.1007/s10555-013-9470-4 (PMC4113682; doi:10.1007/s10555-013-9470-4)

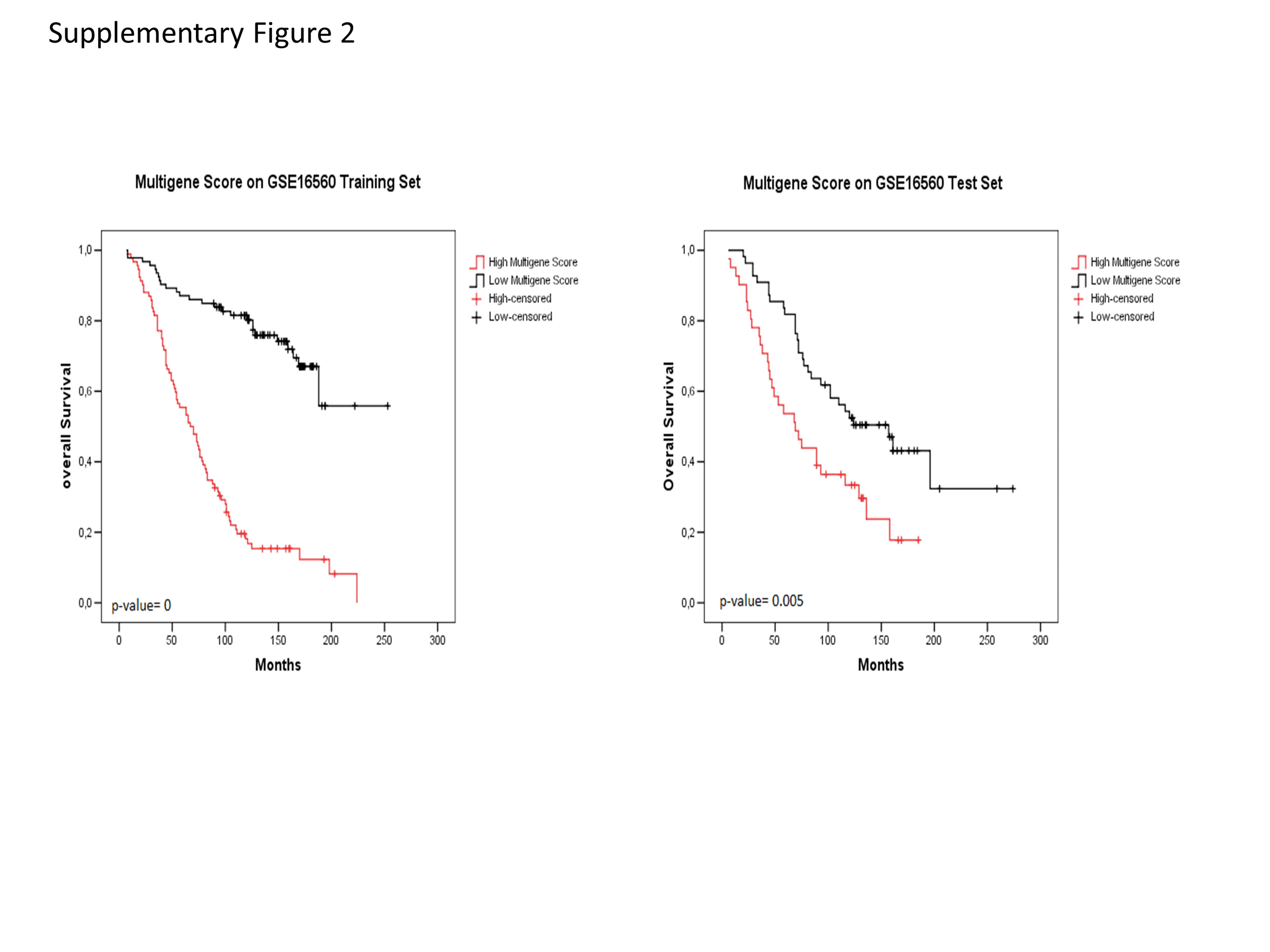

Supplement: Supplementary file 2 — Kaplan–Meier curves for the multigene score on a random split of the dataset GSE16560 to create training and test sets (JPEG 284 kb) [file 10555_2013_9470_Fig10_ESM.jpg]

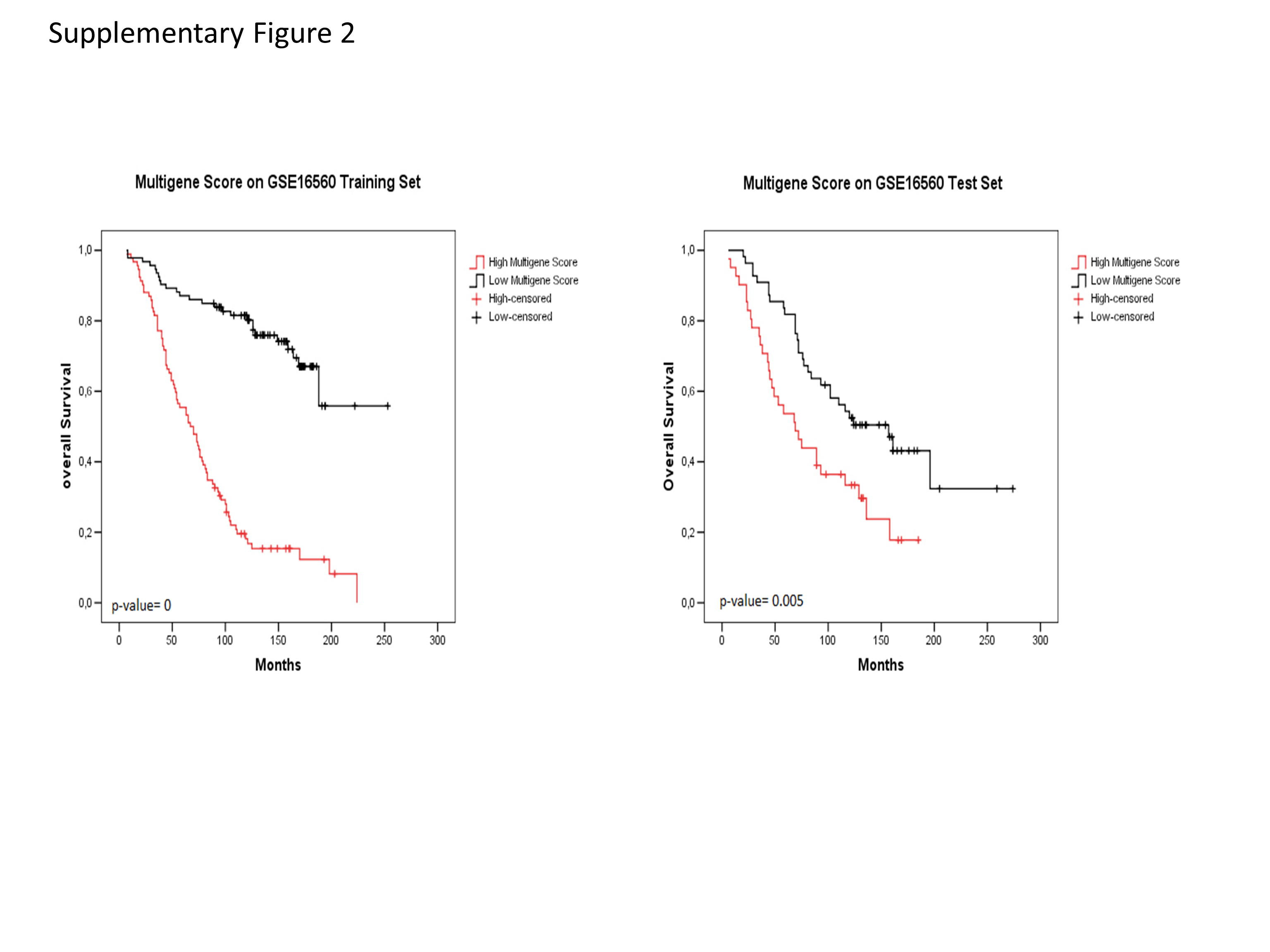

Supplement: Supplementary file 3 — High-resolution image (TIFF 573 kb) [file 10555_2013_9470_MOESM2_ESM.tif]
