# Supplementary material for: Validation of proposed prostate cancer biomarkers with gene expression data: a long road to travel
Source: Cancer Metastasis Rev. 2014 Jan 30;33(2):657–71. doi: 10.1007/s10555-013-9470-4 (PMC4113682; doi:10.1007/s10555-013-9470-4)
Supplement: Supplementary file 4 — List of prostate cancer biomarkers considered for the present study with bibliographic references (PDF 632 kb) [file 10555_2013_9470_MOESM3_ESM.pdf]

Marker	Type	Method	Sample	Reference
FKBP4	diagnosis	protein	tissue	(1)
ITGA3	prognosis	protein	tissue	(2)
ITGA2B	prognosis	rna/protein	cell lines	(3)
ETV1	prognosis	rna	tissue	(4)
IL32	prognosis	rna	cell lines	(5)
BRCA1	prognosis	protein	tissue	(6)
ELOVL5	diagnosis/prognosis	rna	tissue	(7)
BID	prognosis	rna	tissue	(4)
IGF1	prognosis	protein	tissue	(8)
SNAI2	diagnosis/prognosis	rna	cell lines	(9)
HGF	diagnosis/prognosis	protein	serum	(10)
CD44	prognosis	rna	tissue	(11)
FLT4	prognosis	rna	tissue	(12)
HSPA5	prognosis	protein	tissue	(13)
NEDD4L	diagnosis/ prognosis	rna	tissue	(14)
PSMC4	diagnosis/ prognosis	rna	tissue	(14)
LETMD1	prognosis	rna	tissue	(4)
COL23A1	prognosis	rna	tissue	(15)
PRSS8	prognosis	rna/protein	tissue	(16)
TNFRSF1A	prognosis	rna	tissue	(4)
RASSF1	prognosis	dna	tissue	(17)
RDH11	diagnosis/prognosis	rna	tissue	(18)
PRKACA	prognosis	protein	tissue	(19)
MCM2	prognosis	protein	tissue	(20)
TP63	diagnosis	protein	tissue	(21)
PTGS2	prognosis	protein	tissue	(22)
NOTCH3	prognosis	rna	tissue	(4)
FSCN1	prognosis	protein	tissue/cell lines	(23)
RARB	prognosis	dna	tissue	(17)
FGFR1	prognosis	dna/rna/protein	tissue	(24)
SCGN	diagnosis	protein	tissue	(25)
RAGE	diagnosis	protein	tissue/serum	(26)
GSTP1	diagnosis	dna	urine	(27)
CD82	diagnosis	protein	tissue	(28)
FOLH1	diagnosis/prognosis	rna	tissue	(18)
MMP2	prognosis	protein	serum	(29)
BIRC5	prognosis	protein	tissue	(30)
EZR	prognosis	protein	tissue	(31)
TGFB2	prognosis	rna/protein	cell lines	(32)
MSH2	prognosis	protein	tissue	(33)
IL11	prognosis	protein	tissue	(34)
PGC	diagnosis	rna	tissue	(35)
MAPK1	prognosis	protein	cell lines	(36)
HMOX1	prognosis	protein	tissue	(37)
EP300	prognosis	protein	tissue	(38)
CHGA	prognosis	protein	serum	(39)
HIF1A	diagnosis	protein	tissue	(40)
PSMB5	diagnosis/prognosis	rna	tissue	(14)
APEX1	diagnosis	protein	tissue	(41)
EFS	diagnosis	dna	tissue	(42)

MMP9	prognosis	rna	cell lines	(9)
PSMA7	diagnosis/prognosis	rna	tissue	(7)
RASSF2	diagnosis	dna	serum/urine	(43)
E2F1	prognosis	rna/protein	tissue	(44)
PGK1	prognosis	rna	tissue	(7)
ACP5	prognosis	protein	serum	(45)
PYCARD	diagnosis/prognosis	rna/protein	tissue/cell lines	(46)
CD276	prognosis	protein	tissue	(47)
NDRG1	diagnosis	protein	tissue	(28)
PPP2CB	diagnosis	protein	tissue	(48)
ADAM2	diagnosis/prognosis	rna	tissue	(7)
PTOV1	diagnosis	rna/protein	tissue	(49)
EPHX3	prognosis	dna	tissue	(50)
TGFB1	prognosis	protein	serum	(51)
ISYNA1	diagnosis	protein	tissue	(52)
HPN	prognosis	rna	tissue	(53)
CAV1	diagnosis/prognosis	protein	serum	(54)
EZH2	prognosis	rna/protein	cell lines	(55)
AGR2	prognosis	rna/protein	tissue/cell lines	(56)
GCK	prognosis	rna/protein	cell lines	(57)
ENG	prognosis	protein	urine	(58)
DVL1	diagnosis	rna/protein	tissue	(59)
CXCL12	prognosis	protein	tissue	(60)
SPOCK2	diagnosis	dna	tissue/cell lines	(61)
DKK1	prognosis	protein	tissue	(62)
RPL19	prognosis	rna	tissue/cell lines	(63)
COL1A1	prognosis	protein	serum	(64)
SMURF2	diagnosis/prognosis	rna	tissue	(14)
NFKB1	prognosis	protein	tissue	(65)
AREG	prognosis			(66)
CBL	prognosis	protein	In vitro study	(67)
PSMD9	prognosis	protein	tissue	(68)
CCR6	prognosis	protein	tissue	(69)
TMEM30A	diagnosis/prognosis	rna	tissue	(7)
VEGFA	prognosis	protein	tissue	(70)
LOX	prognosis	rna	tissue	(71)
PDGFRB	prognosis	protein	tissue	(72)
KNG1	diagnosis	protein	tissue	(73)
HYAL1	prognosis	rna/protein	tissue	(74)
ID2	prognosis	protein	tissue	(75)
SDC1	prognosis	protein	tissue	(76)
RND3	prognosis	rna	cell lines	(5)
DHCR24	diagnosis/prognosis	rna	tissue	(7)
TFAP2E	diagnosis	dna	serum/urine	(43)
STAG1	diagnosis/prognosis	rna	serum	(77)
CCND2	prognosis	dna	tissue	(17)
C2ORF40	diagnosis	dna	tissue	(42)
RAD23B	prognosis	rna	tissue	(4)
EPCAM	prognosis	protein	tissue	(78)
TNFSF11	prognosis	protein	tissue	(79)
EGR1	prognosis	protein	tissue	(80)

CSTA	prognosis	protein	tissue	(81)
PMS2	prognosis	protein	tissue	(82)
PRDX4	diagnosis/prognosis	protein	tissue	(1)
SPDEF	prognosis	rna/protein	tissue	(83)
GNMT	prognosis	protein	tissue	(84)
CDKN1A	prognosis	rna	cell lines	(5)
GLO1	diagnosis/prognosis	rna	tissue	(7)
MT1G	prognosis	dna	tissue	(85)
ABCC4	prognosis	rna	cell lines	(86)
SOX9	diagnosis/prognosis	rna	cell lines	(9)
ID1	prognosis	protein	tissue	(75)
SBDS	diagnosis/prognosis	rna	tissue	(7)
PIN1	prognosis	protein	tissue	(87)
HIP1	prognosis	protein	tissue	(88)
KDR	prognosis	rna	tissue	(12)
FLNC	diagnosis	dna	tissue	(42)
GAD1	diagnosis	rna/protein	tissue	(89)
SHBG	diagnosis	protein	serum	(90)
HSD17B3	prognosis	rna/protein	tissue	(91)
PDLIM4	diagnosis/prognosis	rna	tissue	(92)
TOP2A	prognosis	rna/protein	tissue	(44)
ZNF132	prognosis	rna/protein	tissue	(93)
LGALS3	diagnosis/prognosis	rna/protein	tissue/cell lines	(94)
RAMP1	diagnosis/prognosis	rna	tissue	(7)
NES	prognosis	rna/protein	tissue	(95)
CHI3L1	prognosis	protein	serum	(96)
CCNA1	invasion	rna/protein	tissue/cell lines	(97)
MICAL2	prognosis	rna/protein	tissue/cell lines	(98)
TMEM66	diagnosis/prognosis	rna	tissue	(7)
CDK7	prognosis	protein	serum	(99)
YWHAQ	diagnosis/prognosis	rna	tissue	(7)
GRP	prognosis	protein	serum	(100)
APC	diagnosis	dna	tissue	(17, 101)
ANXA1	prognosis	rna	tissue	(4)
C7ORF23	diagnosis	dna	tissue	(101)
KRT7	diagnosis	protein	tissue	(102)
IL6	prognosis	protein	serum	(51)
MYC	diagnosis	protein	tissue	(48)
PIM1	diagnosis/prognosis	rna/protein	tissue	(103)
MMP7	prognosis	protein	serum	(104)
MMP13	diagnosis	protein	serum	(105)
THBS1	diagnosis	protein	serum	(106)
CYP1B1	prognosis	dna	blood	(107)
ANXA7	prognosis	rna	tissue/cell lines	(108)
MSMB	prognosis	protein	serum	(109)
CCNG2	prognosis	rna	tissue	(4)
BRCA2	prognosis	dna	tissue	(110)
CYP11A1	prognosis	dna	blood	(107)
SMAD4	prognosis	protein	tissue	(111)
KLK3	diagnosis/prognosis	rna	tissue	(18)
SYTL1	diagnosis	protein	cell lines	(112)

CYR61	prognosis	protein	tissue	(113)
S100A8	diagnosis	protein	tissue/serum	(26)
TMEFF2	prognosis	rna	cell lines	(114)
HSPD1	prognosis	protein	cell lines	(115)
TRPM8	diagnosis	rna/ protein	tissue	(116)
LRIG1	prognosis	rna/ protein	tissue	(117)
EAF2	prognosis	rna/ protein	tissue/cell lines	(118)
SSBP2	prognosis	dna/rna/protein	tissue/cell lines	(119)
EGFR	prognosis			(120)
IGFBP3	prognosis	dna	tissue	(121)
TACC1	prognosis	dna/rna/protein	tissue	(24)
DPYS	diagnosis	dna	tissue/cell lines	(61)
EBAG9	prognosis	protein	tissue	(122)
CDKN2A	prognosis	protein	tissue	(123)
MKI67	prognosis	protein	tissue	(124)
CYP17A1	prognosis	protein	tissue	(8)
ATM	prognosis	protein	tissue	(125)
VEGFC	prognosis	protein	tissue	(126)
TWF1	diagnosis/prognosis	rna	cell lines	(9)
BAG3	prognosis	protein	tissue	(127)
DSPP	diagnosis	rna/ protein	serum	(128)
GJA1	prognosis	protein	tissue	(129)
SLC16A12	diagnosis	dna	tissue/cell lines	(61)
PART1	diagnosis	rna	tissue	(130)
BMP6	prognosis	protein	tissue	(131)
CD8A	prognosis	protein	tissue	(132)
ABCA5	diagnosis	rna/ protein	tissue/urine	(133)
UCHL1	prognosis	rna	cell lines	(114)
NCAM2	diagnosis/prognosis	rna	tissue	(7)
KCNMA1	diagnosis	dna	tissue	(42)
BUB1B	prognosis	protein	tissue	(134)
TIMP4	diagnosis	protein	tissue	(135, 136)
STEAP2	diagnosis/prognosis	rna	tissue	(18)
ERG	diagnosis	protein	tissue	(137-139)
SLC45A3	diagnosis/prognosis	rna	tissue	(18)
APOA2	diagnosis	protein	serum	(140)
PRAC	diagnosis/prognosis	rna	tissue	(18)
HOXB13	diagnosis	rna	tissue	(18)
SIM2	diagnosis	rna/ protein	tissue	(141)
HK2	prognosis	rna/protein	cell lines	(57)
SPON2	diagnosis/prognosis	rna	tissue	(7)
TFF3	diagnosis	rna	urine	(142)
CSTB	diagnosis	protein	tissue	(143)
CD3G	prognosis	protein	tissue	(144)
IL6R	prognosis	protein	serum	(51)
AZGP1	diagnosis	protein	tissue/serum	(145)
ALOX15	diagnosis	protein	tissue	(146)
CAMK2N1	diagnosis/prognosis	rna	tissue	(7)
FRZB	prognosis	rna/ protein	cell lines	(147)
S100A9	diagnosis	protein	tissue/serum	(26)
CLDN1	prognosis	protein	tissue	(148)

CXCR1	prognosis	protein	tissue	(149)
GUCY1A3	prognosis	rna	cell lines	(86)
SPINK1	prognosis	rna	urine	(142)
TERT	diagnosis	rna	tissue/urine	(150)
CSF2	diagnosis/prognosis	rna	cell lines	(9)
CTSB	prognosis	protein	serum	(151)
TNFRSF11B	prognosis	protein	serum	(152)
EN2	diagnosis	rna/protein	tissue/cell lines	(153)
ALDH1A1	prognosis	protein	tissue/cell lines	(154)
LOC644063	diagnosis/prognosis	protein	tissue	(155)
TRPV6	prognosis	rna/protein	tissue	(156)
FBP1	prognosis	rna	tissue	(4)
FIGF	prognosis	rna	tissue	(12)
CLDN3	diagnosis	protein	tissue	(157)
SPINT1	prognosis	protein	tissue/cell lines	(158)
ILK	diagnosis	rna/protein	tissue/cell lines	(159)
MCM7	prognosis	dna/protein	tissue	(160)
NGFRAP1	diagnosis/prognosis	rna	tissue	(7)
B2M	diagnosis	protein	serum	(161)
STAT6	prognosis	rna	tissue	(53)
SMAD3	diagnosis/prognosis	rna	cell lines	(9)
PDIA3	prognosis	rna/protein	tissue	(162)
NKX3-1	diagnosis	rna	tissue	(18)
OR51E2	diagnosis/prognosis	rna	tissue	(18)
MMP26	diagnosis	protein	tissue	(135, 136)
MVD	diagnosis	protein	tissue	(40)
PSCA	diagnosis/prognosis			(163)
KLK1	prognosis			(164)
KLK4	prognosis			(164)
KLK5	prognosis			(164)
KLK6	prognosis			(164)
KLK7	prognosis			(164)
KLK8	prognosis			(164)
KLK9	prognosis			(164)
KLK10	prognosis			(164)
KLK11	diagnosis/prognosis			(165)
KLK12	prognosis			(164)
KLK13	prognosis			(164)
KLK14	prognosis			(164)
KLK15	prognosis			(164)
KLK2	diagnosis/prognosis	rna	tissue	(18)
ARF4	diagnosis	dna	urine	(27)
FEN1	diagnosis	protein	tissue	(166)
NSMCE1	diagnosis	dna	tissue/cell lines	(61)
NPR1	prognosis	rna/protein	tissue/cell lines	(167)
IL8	prognosis	protein	cell lines	(149, 168)
SDC2	prognosis	protein	tissue	(169)
FASN	diagnosis/prognosis	rna	tissue	(18)
DCXR	diagnosis	rna	tissue/cell lines	(170)
ALCAM	diagnosis	protein	tissue	(171)

MGMT	diagnosis	dna	urine	(27)
CD14	diagnosis	protein	urine	(172)
PRKCE	diagnosis			(173)
KRT19	prognosis	rna	serum	(174)
CLSTN1	diagnosis	dna	tissue/cell lines	(61)
PTEN	prognosis	rna/protein	tissue	(175)
BSG	prognosis	protein	tissue	(176)
PPP1CA	diagnosis	protein	tissue	(48)
ADRBK1	diagnosis	protein	tissue	(48)
SMARCC1	prognosis	protein	tissue	(177)
CD34	prognosis	protein	tissue	(68)
LEP	prognosis	protein	serum	(178)
MARCKSL1	diagnosis/prognosis	rna	tissue	(7)
SMAD2	diagnosis	protein	tissue	(111)
A2M	diagnosis/prognosis	protein	serum	(179)
SPHK1	diagnosis/prognosis	rna	cell lines	(9)
CD151	prognosis	protein	tissue	(180)
CXCR2	prognosis	protein	tissue	(149)
SOX2	prognosis	rna/protein	cell lines	(181)
KPNA2	prognosis	rna/protein	tissue	(182)
CSF1R	prognosis	protein	tissue	(144)
ANXA2	diagnosis	protein	tissue	(183)
MTA1	prognosis	rna/protein		(184)
NKX2-5	diagnosis	dna	tissue/cell lines	(61)
NPBWR1	prognosis	dna	tissue	(50)
CHEK2	prognosis	protein	tissue	(125)
TMPRSS2	prognosis	rna	tissue	(185)
CSF1	prognosis	protein	tissue	(144)
WT1	prognosis	dna/rna/protein	tissue	(24)
MUC1	prognosis	protein	tissue	(186)
KRT5	diagnosis	protein	tissue	(187)
TEAD1	prognosis	rna/protein	tissue/cell lines	(188)
EIF4EBP1	prognosis	protein	tissue	(189)
PLA2G2A	prognosis	rna/protein	tissue	(190)
SCN8A	prognosis	rna/protein	tissue	(191)
SND1	diagnosis	rna/protein	tissue/cell lines	(192)
MAP3K5	prognosis	rna/protein	tissue	(162)
GSTK1	diagnosis	protein	tissue	(52)
MYH6	diagnosis	rna/protein	tissue	(193)
PSAP	diagnosis/prognosis	protein	tissue/serum	(194)
HIST1H4K	diagnosis	dna	serum/urine	(43)
MTOR	prognosis	protein	tissue	(189)
POU5F1	diagnosis	rna/protein	tissue	(195)
MIR647	prognosis	rna	tissue	(4)
MIR99A	prognosis	rna	tissue/cell lines	(196)
MIR519D	prognosis	rna	tissue	(4)
MIR100	prognosis	rna	tissue	(197)
TNFRSF25	prognosis	dna	tissue	(121)
CKMT1B	prognosis	protein	cell lines	(198)
NME1	diagnosis	protein	tissue	(48)
MIF	diagnosis/prognosis	protein	serum	(199)

AMACR	diagnosis	rna/protein	tissue	(200)
BAG1	prognosis	protein	tissue	(201)
CD24	prognosis	rna/protein	tissue	(202)
CLDN4	prognosis	protein	tissue	(148)
CTNNB1	prognosis	protein	tissue	(148)
SPA17	prognosis	rna	tissue/cell lines	(203)
ASAP2	prognosis	rna	tissue/cell lines	(203)
GLI2	prognosis	protein	tissue	(8)
SYP	prognosis	protein	tissue	(8)
GLI1	prognosis	protein	tissue	(8)
SMO	prognosis	protein	tissue	(8)
TP53	prognosis	protein	tissue	(8)
IGF1R	prognosis	protein	tissue	(8)
AR	prognosis	protein	tissue	(8)
BCL2	prognosis	protein	tissue	(8)
PECAM1	prognosis	protein	tissue	(8)
SHH	prognosis	protein	tissue	(8)
PTCH1	prognosis	protein	tissue	(8)
SLC2A1	prognosis	rna	tissue	(71)
SERPINF1	prognosis	protein	serum	(204)
SERPINF2	prognosis	protein	serum	(204)
ANXA3	diagnosis/prognosis	rna/protein	tissue	(205)
CRISP3	diagnosis/prognosis	rna/protein	tissue	(205)
CHIT1	prognosis	protein	serum	(96)
CXCL16	diagnosis/prognosis	protein	serum/urine	(206)
PTX3	diagnosis/prognosis	protein	serum/urine	(206)
FST	diagnosis/prognosis	protein	serum/urine	(206)
MIRLET7D	diagnosis/prognosis			(207)
Mir195	diagnosis/prognosis			(207)
Mir203	diagnosis/prognosis			(207)
MIR99B	prognosis	rna	tissue/cell lines	(196)
CXCR1	prognosis	protein	tissue	(149)
PCA3	prognosis	rna	tissue	(208)
SHC1	prognosis			(209)
PSIP1	prognosis			(209)
MT1A	prognosis			(210)
MT2A	prognosis			(210)
WNT5A	prognosis	rna	tissue	(211)
KLF6	prognosis	rna	tissue	(211)
ANGPT2	prognosis	rna/protein	tissue	(212)
CSNK2A1	diagnosis/prognosis	protein	tissue	(213)
GOLM1	diagnosis	rna/protein	cell lines	(214)
ADAM15	prognosis	protein	tissue	(215)
SLIT2	prognosis	rna/protein	cell lines	(216)
IGFBP2	prognosis	rna	tissue	(217)
ADAM9	prognosis	rna/protein	tissue	(218)
CXCR6	prognosis	rna	cell lines	(219)
REG4	diagnosis	protein	tissue	(220)
POSTN	prognosis	protein	tissue	(221)

CGA	prognosis	protein	tissue	(222)
IGF2BP3	diagnosis	protein	tissue	(223)
DAB2IP	prognosis	protein	cell lines	(224)
RPS2	diagnosis	protein	cell lines	(225)
BMP6	prognosis	protein	tissue	(131)
EEF1A1	prognosis	protein	tissue	(226)
BMX	diagnosis	rna	cell lines	(227)
ZEB1	prognosis	rna/protein	cell lines	(228)
PITX2	prognosis	dna	tissue	(229)
PCGEM1	diagnosis	rna	tissue	(230)
AKAP12	prognosis	dna	tissue	(231)
ADAM8	prognosis	rna/protein	tissue	(232)
OR51E1	diagnosis	rna	tissue	(233)
C4A	prognosis	protein	tissue	(234)
SERPINA5	prognosis	protein	tissue	(234)
NCOA3	prognosis			(235)
TIMP1	prognosis	protein	tissue	(236)
TIMP2	prognosis	protein	tissue	(236)
DPP4	prognosis	protein	cell lines	(237)
SPP1	prognosis	protein	tissue	(238)
SARDH	diagnosis	protein	urine	(150)
SNCG	prognosis	dna/protein	tissue	(239)
ENO2	prognosis	protein	tissue	(240)
PLAU	diagnosis/prognosis	protein	cell lines	(241)
PLAUR	diagnosis/prognosis	protein	cell lines	(241)
HTR3A	prognosis	protein	serum	(242)
BAP	prognosis	protein	serum	(243)
HAS1	prognostic	rna/protein	cell lines	(244)

1. Lin JF, Xu J, Tian HY*, et al.* Identification of candidate prostate cancer biomarkers in prostate needle biopsy specimens using proteomic analysis. Int J Cancer 2007;121:2596-605.
2. Pontes-Junior J, Reis ST, de Oliveira LC*, et al.* Association between integrin expression and prognosis in localized prostate cancer. Prostate;70:1189-95.
3. Ramirez NE, Zhang Z, Madamanchi A, *et al.* The α2β1 integrin is a metastasis suppressor in mouse models and human cancer J Clin Invest 2011;121:226-237.
4. Long Q, Johnson BA, Osunkoya AO*, et al.* Protein-coding and microRNA biomarkers of recurrence of prostate cancer following radical prostatectomy. Am J Pathol;179:46-54.
5. Trojan L, Schaaf A, Steidler A*, et al.* Identification of metastasis-associated genes in prostate cancer by genetic profiling of human prostate cancer cell lines. Anticancer Res 2005;25:183-91.
6. Fiorentino M, Judson G, Penney K*, et al.* Immunohistochemical expression of BRCA1 and lethal prostate cancer. Cancer Res;70:3136-9.
7. Romanuik TL, Ueda T, Le N*, et al.* Novel biomarkers for prostate cancer including noncoding transcripts. Am J Pathol 2009;175:2264-76.
8. Tzelepi V, Efstathiou E, Wen S*, et al.* Persistent, biologically meaningful prostate cancer after 1 year of androgen ablation and docetaxel treatment. J Clin Oncol;29:2574-81.
9. Baniwal SK, Khalid O, Gabet Y*, et al.* Runx2 transcriptome of prostate cancer cells: insights into invasiveness and bone metastasis. Mol Cancer;9:258.

10. Gupta A, Karakiewicz PI, Roehrborn CG, Lotan Y, Zlotta AR, Shariat SF. Predictive value of plasma hepatocyte growth factor/scatter factor levels in patients with clinically localized prostate cancer. Clin Cancer Res 2008;14:7385-90.
11. Ekici S, Ayhan A, Kendi S, Ozen H. Determination of prognosis in patients with prostate cancer treated with radical prostatectomy: prognostic value of CD44v6 score. J Urol 2002;167:2037-41.
12. Kaushal V, Mukunyadzi P, Dennis RA, Siegel ER, Johnson DE, Kohli M. Stage-specific characterization of the vascular endothelial growth factor axis in prostate cancer: expression of lymphangiogenic markers is associated with advanced-stage disease. Clin Cancer Res 2005;11:584-93.
13. Tan SS, Ahmad I, Bennett HL*, et al.* GRP78 up-regulation is associated with androgen receptor status, Hsp70-Hsp90 client proteins and castrate-resistant prostate cancer. J Pathol;223:81-7.
14. Hellwinkel OJ, Asong LE, Rogmann JP*, et al.* Transcription alterations of members of the ubiquitin-proteasome network in prostate carcinoma. Prostate Cancer Prostatic Dis;14:38-45.
15. Banyard J, Bao L, Hofer MD*, et al.* Collagen XXIII expression is associated with prostate cancer recurrence and distant metastases. Clin Cancer Res 2007;13:2634-42.
16. Takahashi S, Suzuki S, Inaguma S*, et al.* Down-regulated expression of prostasin in high-grade or hormone-refractory human prostate cancers. Prostate 2003;54:187-93.
17. Rosenbaum E, Hoque MO, Cohen Y*, et al.* Promoter hypermethylation as an independent prognostic factor for relapse in patients with prostate cancer following radical prostatectomy. Clin Cancer Res 2005;11:8321-5.
18. Edwards S, Campbell C, Flohr P*, et al.* Expression analysis onto microarrays of randomly selected cDNA clones highlights HOXB13 as a marker of human prostate cancer. Br J Cancer 2005;92:376-81.
19. Khor LY, Bae K, Al-Saleem T*, et al.* Protein kinase A RI-alpha predicts for prostate cancer outcome: analysis of radiation therapy oncology group trial 86-10. Int J Radiat Oncol Biol Phys 2008;71:1309-15.
20. Meng MV, Grossfeld GD, Williams GH*, et al.* Minichromosome maintenance protein 2 expression in prostate: characterization and association with outcome after therapy for cancer. Clin Cancer Res 2001;7:2712-8.
21. Weinstein MH, Signoretti S, Loda M. Diagnostic utility of immunohistochemical staining for p63, a sensitive marker of prostatic basal cells. Mod Pathol 2002;15:1302-8.
22. Wang W, Bergh A, Damber JE. Cyclooxygenase-2 expression correlates with local chronic inflammation and tumor neovascularization in human prostate cancer. Clin Cancer Res 2005;11:3250-6.
23. Darnel AD, Behmoaram E, Vollmer RT, *et al.* Fascin regulates prostate cancer cell invasion and is associated with metastasis and biochemical failure in prostate cancer. Clin Cancer Res. 2009 15:1376-83.
24. Devilard E, Bladou F, Ramuz O*, et al.* FGFR1 and WT1 are markers of human prostate cancer progression. BMC Cancer 2006;6:272.
25. Adolf K, Wagner L, Bergh A*, et al.* Secretagogin is a new neuroendocrine marker in the human prostate. Prostate 2007;67:472-84.
26. Hermani A, Hess J, De Servi B*, et al.* Calcium-binding proteins S100A8 and S100A9 as novel diagnostic markers in human prostate cancer. Clin Cancer Res 2005;11:5146-52.
27. Hoque MO, Topaloglu O, Begum S*, et al.* Quantitative methylation-specific polymerase chain reaction gene patterns in urine sediment distinguish prostate cancer patients from control subjects. J Clin Oncol 2005;23:6569-75.
28. Liu W, Iiizumi-Gairani M, Okuda H*, et al.* KAI1 gene is engaged in NDRG1 gene-mediated metastasis suppression through the ATF3-NFkappaB complex in human prostate cancer. J Biol Chem;286:18949-59.
29. Salminen EK, Kallioinen MJ, Ala-Houhala MA*, et al.* Survival markers related to bone metastases in prostate cancer. Anticancer Res 2006;26:4879-84.
30. Shariat SF, Ashfaq R, Roehrborn CG, Slawin KM, Lotan Y. Expression of survivin and apoptotic biomarkers in benign prostatic hyperplasia. J Urol 2005;174:2046-50.
31. Valdman A, Fang X, Pang ST, Nilsson B, Ekman P, Egevad L. Ezrin expression in prostate cancer and benign prostatic tissue. Eur Urol 2005;48:852-7.

32. Aalinkeel R, Nair MP, Sufrin G*, et al.* Gene expression of angiogenic factors correlates with metastatic potential of prostate cancer cells. Cancer Res 2004;64:5311-21.
33. Prtilo A, Leach FS, Markwalder R*, et al.* Tissue microarray analysis of hMSH2 expression predicts outcome in men with prostate cancer. J Urol 2005;174:1814-8; discussion 18.
34. Zurita AJ, Troncoso P, Cardo-Vila M, Logothetis CJ, Pasqualini R, Arap W. Combinatorial screenings in patients: the interleukin-11 receptor alpha as a candidate target in the progression of human prostate cancer. Cancer Res 2004;64:435-9.
35. Antunes AA, Leite KR, Sousa-Canavez JM, Camara-Lopes LH, Srougi M. The role of prostate specific membrane antigen and pepsinogen C tissue expression as an adjunctive method to prostate cancer diagnosis. J Urol 2009;181:594-600.
36. Oka H, Chatani Y, Kohno M, Kawakita M, Ogawa O. Constitutive activation of the 41- and 43-kDa mitogen-activated protein (MAP) kinases in the progression of prostate cancer to an androgen-independent state. Int J Urol 2005;12:899-905.
37. Li Y, Su J, DingZhang X*, et al.* PTEN deletion and heme oxygenase-1 overexpression cooperate in prostate cancer progression and are associated with adverse clinical outcome. J Pathol;224:90-100.
38. Isharwal S, Miller MC, Marlow C, Makarov DV, Partin AW, Veltri RW. p300 (histone acetyltransferase) biomarker predicts prostate cancer biochemical recurrence and correlates with changes in epithelia nuclear size and shape. Prostate 2008;68:1097-104.
39. Berruti A, Dogliotti L, Mosca A*, et al.* Effects of the somatostatin analog lanreotide on the circulating levels of chromogranin-A, prostate-specific antigen, and insulin-like growth factor-1 in advanced prostate cancer patients. Prostate 2001;47:205-11.
40. Lekas A, Lazaris AC, Deliveliotis C*, et al.* The expression of hypoxia-inducible factor-1alpha (HIF-1alpha) and angiogenesis markers in hyperplastic and malignant prostate tissue. Anticancer Res 2006;26:2989-93.
41. Kelley MR, Cheng L, Foster R*, et al.* Elevated and altered expression of the multifunctional DNA base excision repair and redox enzyme Ape1/ref-1 in prostate cancer. Clin Cancer Res 2001;7:824-30.
42. Vanaja DK, Ehrich M, Van den Boom D*, et al.* Hypermethylation of genes for diagnosis and risk stratification of prostate cancer. Cancer Invest 2009;27:549-60.
43. Payne SR, Serth J, Schostak M*, et al.* DNA methylation biomarkers of prostate cancer: confirmation of candidates and evidence urine is the most sensitive body fluid for non-invasive detection. Prostate 2009;69:1257-69.
44. Malhotra S, Lapointe J, Salari K*, et al.* A tri-marker proliferation index predicts biochemical recurrence after surgery for prostate cancer. PLoS One;6:e20293.
45. Chao TY, Wu YY, Janckila AJ. Tartrate-resistant acid phosphatase isoform 5b (TRACP 5b) as a serum maker for cancer with bone metastasis. Clin Chim Acta;411:1553-64.
46. Das PM, Ramachandran K, Vanwert J*, et al.* Methylation mediated silencing of TMS1/ASC gene in prostate cancer. Mol Cancer 2006;5:28.
47. Roth TJ, Sheinin Y, Lohse CM*, et al.* B7-H3 ligand expression by prostate cancer: a novel marker of prognosis and potential target for therapy. Cancer Res 2007;67:7893-900.
48. Prowatke I, Devens F, Benner A*, et al.* Expression analysis of imbalanced genes in prostate carcinoma using tissue microarrays. Br J Cancer 2007;96:82-8.
49. Benedit P, Paciucci R, Thomson TM*, et al.* PTOV1, a novel protein overexpressed in prostate cancer containing a new class of protein homology blocks. Oncogene 2001;20:1455-64.
50. Cottrell S, Jung K, Kristiansen G*, et al.* Discovery and validation of 3 novel DNA methylation markers of prostate cancer prognosis. J Urol 2007;177:1753-8.
51. Shariat SF, Semjonow A, Lilja H, Savage C, Vickers AJ, Bjartell A. Tumor markers in prostate cancer I: blood-based markers. Acta Oncol;50 Suppl 1:61-75.
52. Karaivanov M, Todorova K, Kuzmanov A, Hayrabedyan S. Quantitative immunohistochemical detection of the molecular expression patterns in proliferative inflammatory atrophy. J Mol Histol 2007;38:1-11.
53. Xu L, Tan AC, Naiman DQ, Geman D, Winslow RL. Robust prostate cancer marker genes emerge from direct integration of inter-study microarray data. Bioinformatics 2005;21:3905-11.

54. Tahir SA, Ren C, Timme TL*, et al.* Development of an immunoassay for serum caveolin-1: a novel biomarker for prostate cancer. Clin Cancer Res 2003;9:3653-9.
55. Karanikolas BD, Figueiredo ML, Wu L. Comprehensive evaluation of the role of EZH2 in the growth, invasion, and aggression of a panel of prostate cancer cell lines. Prostate;70:675-88.
56. Brychtova V, Vojtesek B, Hrstka R. Anterior gradient 2: a novel player in tumor cell biology. Cancer Lett;304:1-7.
57. Mize GJ, Wang W, Takayama TK. Prostate-specific kallikreins-2 and -4 enhance the proliferation of DU-145 prostate cancer cells through protease-activated receptors-1 and -2. Mol Cancer Res 2008;6:1043-51.
58. Fujita K, Ewing CM, Chan DY*, et al.* Endoglin (CD105) as a urinary and serum marker of prostate cancer. Int J Cancer 2009;124:664-9.
59. Mizutani K, Miyamoto S, Nagahata T, Konishi N, Emi M, Onda M. Upregulation and overexpression of DVL1, the human counterpart of the Drosophila dishevelled gene, in prostate cancer. Tumori 2005;91:546-51.
60. Liang JJ, Zhu S, Bruggeman R*, et al.* High levels of expression of human stromal cell-derived factor-1 are associated with worse prognosis in patients with stage II pancreatic ductal adenocarcinoma. Cancer Epidemiol Biomarkers Prev;19:2598-604.
61. Chung W, Kwabi-Addo B, Ittmann M*, et al.* Identification of novel tumor markers in prostate, colon and breast cancer by unbiased methylation profiling. PLoS One 2008;3:e2079.
62. Hall CL, Daignault SD, Shah RB, Pienta KJ, Keller ET. Dickkopf-1 expression increases early in prostate cancer development and decreases during progression from primary tumor to metastasis. Prostate 2008;68:1396-404.
63. Bee A, Ke Y, Forootan S*, et al.* Ribosomal protein l19 is a prognostic marker for human prostate cancer. Clin Cancer Res 2006;12:2061-5.
64. Klepzig M, Jonas D, Oremek GM. Procollagen type 1 amino-terminal propeptide: a marker for bone metastases in prostate carcinoma. Anticancer Res 2009;29:671-3.
65. Domingo-Domenech J, Mellado B, Ferrer B*, et al.* Activation of nuclear factor-kappaB in human prostate carcinogenesis and association to biochemical relapse. Br J Cancer 2005;93:1285-94.
66. Busser B, Sancey L, Brambilla E, Coll JL, Hurbin A. The multiple roles of amphiregulin in human cancer. Biochim Biophys Acta;1816:119-31.
67. Kumar EA, Charvet CD, Lokesh GL, Natarajan A. High-throughput fluorescence polarization assay to identify inhibitors of Cbl(TKB)-protein tyrosine kinase interactions. Anal Biochem;411:254-60.
68. Nassif AE, Tambara Filho R. Immunohistochemistry expression of tumor markers CD34 and P27 as a prognostic factor of clinically localized prostate adenocarcinoma after radical prostatectomy. Rev Col Bras Cir;37:338-44.
69. Ghadjar P, Loddenkemper C, Coupland SE*, et al.* Chemokine receptor CCR6 expression level and aggressiveness of prostate cancer. J Cancer Res Clin Oncol 2008;134:1181-9.
70. Gravdal K, Halvorsen OJ, Haukaas SA, Akslen LA. Proliferation of immature tumor vessels is a novel marker of clinical progression in prostate cancer. Cancer Res 2009;69:4708-15.
71. Stewart GD, Gray K, Pennington CJ*, et al.* Analysis of hypoxia-associated gene expression in prostate cancer: lysyl oxidase and glucose transporter-1 expression correlate with Gleason score. Oncol Rep 2008;20:1561-7.
72. Mathew P, Tannir N, Tu SM*, et al.* Accelerated disease progression in prostate cancer and bone metastases with platelet-derived growth factor receptor inhibition: observations with tandutinib. Cancer Chemother Pharmacol;68:889-96.
73. Kumaresan K, Kakkar N, Verma A, Mandal AK, Singh SK, Joshi K. Diagnostic utility of alpha-methylacyl CoA racemase (P504S) & HMWCK in morphologically difficult prostate cancer. Diagn Pathol;5:83.
74. Lokeshwar VB, Rubinowicz D, Schroeder GL*, et al.* Stromal and epithelial expression of tumor markers hyaluronic acid and HYAL1 hyaluronidase in prostate cancer. J Biol Chem 2001;276:11922-32.
75. Coppe JP, Itahana Y, Moore DH, Bennington JL, Desprez PY. Id-1 and Id-2 proteins as molecular markers for human prostate cancer progression. Clin Cancer Res 2004;10:2044-51.

76. Zellweger T, Ninck C, Mirlacher M*, et al.* Tissue microarray analysis reveals prognostic significance of syndecan-1 expression in prostate cancer. Prostate 2003;55:20-9.
77. Cardillo MR, Di Silverio F. Prostate--specific G protein couple receptor genes and STAG1/PMEPA1 in peripheral blood from patients with prostatic cancer. Int J Immunopathol Pharmacol 2006;19:871-8.
78. Benko G, Spajic B, Kruslin B, Tomas D. Impact of the EpCAM expression on biochemical recurrence-free survival in clinically localized prostate cancer. Urol Oncol.
79. Perez-Martinez FC, Alonso V, Sarasa JL*, et al.* Receptor activator of nuclear factor-kappaB ligand (RANKL) as a novel prognostic marker in prostate carcinoma. Histol Histopathol 2008;23:709-15.
80. Ahmed MM, Chendil D, Lele S*, et al.* Early growth response-1 gene: potential radiation response gene marker in prostate cancer. Am J Clin Oncol 2001;24:500-5.
81. Sinha AA, Quast BJ, Wilson MJ*, et al.* Ratio of cathepsin B to stefin A identifies heterogeneity within Gleason histologic scores for human prostate cancer. Prostate 2001;48:274-84.
82. Norris AM, Woodruff RD, D'Agostino RB, Jr., Clodfelter JE, Scarpinato KD. Elevated levels of the mismatch repair protein PMS2 are associated with prostate cancer. Prostate 2007;67:214-25.
83. Ghadersohi A, Sharma S, Zhang S*, et al.* Prostate-derived Ets transcription factor (PDEF) is a potential prognostic marker in patients with prostate cancer. Prostate 2011;71:1178-88.
84. Song YH, Shiota M, Kuroiwa K, Naito S, Oda Y. The important role of glycine N-methyltransferase in the carcinogenesis and progression of prostate cancer. Mod Pathol;24:1272-80.
85. Henrique R, Jeronimo C, Hoque MO*, et al.* MT1G hypermethylation is associated with higher tumor stage in prostate cancer. Cancer Epidemiol Biomarkers Prev 2005;14:1274-8.
86. Dong Y, Zhang H, Gao AC, Marshall JR, Ip C. Androgen receptor signaling intensity is a key factor in determining the sensitivity of prostate cancer cells to selenium inhibition of growth and cancer-specific biomarkers. Mol Cancer Ther 2005;4:1047-55.
87. Ayala G, Wang D, Wulf G*, et al.* The prolyl isomerase Pin1 is a novel prognostic marker in human prostate cancer. Cancer Res 2003;63:6244-51.
88. Rao DS, Hyun TS, Kumar PD*, et al.* Huntingtin-interacting protein 1 is overexpressed in prostate and colon cancer and is critical for cellular survival. J Clin Invest 2002;110:351-60.
89. Jaraj SJ, Augsten M, Haggarth L*, et al.* GAD1 is a biomarker for benign and malignant prostatic tissue. Scand J Urol Nephrol;45:39-45.
90. Gill JK, Wilkens LR, Pollak MN, Stanczyk FZ, Kolonel LN. Androgens, growth factors, and risk of prostate cancer: the Multiethnic Cohort. Prostate;70:906-15.
91. Montgomery RB, Mostaghel EA, Vessella R*, et al.* Maintenance of intratumoral androgens in metastatic prostate cancer: a mechanism for castration-resistant tumor growth. Cancer Res 2008;68:4447-54.
92. Vanaja DK, Ballman KV, Morlan BW*, et al.* PDLIM4 repression by hypermethylation as a potential biomarker for prostate cancer. Clin Cancer Res 2006;12:1128-36.
93. Abildgaard MO, Borre M, Mortensen MM*, et al.* Downregulation of zinc finger protein 132 in prostate cancer is associated with aberrant promoter hypermethylation and poor prognosis. Int J Cancer;130:885-95.
94. Wang Y, Nangia-Makker P, Tait L*, et al.* Regulation of prostate cancer progression by galectin-3. Am J Pathol 2009;174:1515-23.
95. Ishiwata T, Matsuda Y, Naito Z. Nestin in gastrointestinal and other cancers: effects on cells and tumor angiogenesis. World J Gastroenterol;17:409-18.
96. Kucur M, Isman FK, Balci C*, et al.* Serum YKL-40 levels and chitotriosidase activity as potential biomarkers in primary prostate cancer and benign prostatic hyperplasia. Urol Oncol 2008;26:47-52.
97. Wegiel B, Bjartell A, Tuomela J*, et al.* Multiple cellular mechanisms related to cyclin A1 in prostate cancer invasion and metastasis. J Natl Cancer Inst 2008;100:1022-36.
98. Ashida S, Furihata M, Katagiri T*, et al.* Expression of novel molecules, MICAL2-PV (MICAL2 prostate cancer variants), increases with high Gleason score and prostate cancer progression. Clin Cancer Res 2006;12:2767-73.
99. Huang S, Lin J, Guo N*, et al.* Elevated serum thymidine kinase 1 predicts risk of pre/early cancerous progression. Asian Pac J Cancer Prev;12:497-505.

100. Yashi M, Nukui A, Kurokawa S*, et al.* Elevated serum progastrin-releasing peptide (31-98) level is a predictor of short response duration after hormonal therapy in metastatic prostate cancer. Prostate 2003;56:305-12.
101. Enokida H, Shiina H, Urakami S*, et al.* Multigene methylation analysis for detection and staging of prostate cancer. Clin Cancer Res 2005;11:6582-8.
102. Kunju LP, Mehra R, Snyder M, Shah RB. Prostate-specific antigen, high-molecular-weight cytokeratin (clone 34betaE12), and/or p63: an optimal immunohistochemical panel to distinguish poorly differentiated prostate adenocarcinoma from urothelial carcinoma. Am J Clin Pathol 2006;125:675-81.
103. Xu Y, Zhang T, Tang H*, et al.* Overexpression of PIM-1 is a potential biomarker in prostate carcinoma. J Surg Oncol 2005;92:326-30.
104. Szarvas T, Becker M, Vom Dorp F*, et al.* Elevated serum matrix metalloproteinase 7 levels predict poor prognosis after radical prostatectomy. Int J Cancer;128:1486-92.
105. Morgia G, Falsaperla M, Malaponte G*, et al.* Matrix metalloproteinases as diagnostic (MMP-13) and prognostic (MMP-2, MMP-9) markers of prostate cancer. Urol Res 2005;33:44-50.
106. Shafer MW, Mangold L, Partin AW, Haab BB. Antibody array profiling reveals serum TSP-1 as a marker to distinguish benign from malignant prostatic disease. Prostate 2007;67:255-67.
107. Cicek MS, Liu X, Casey G, Witte JS. Role of androgen metabolism genes CYP1B1, PSA/KLK3, and CYP11alpha in prostate cancer risk and aggressiveness. Cancer Epidemiol Biomarkers Prev 2005;14:2173-7.
108. Srivastava M, Bubendorf L, Nolan L*, et al.* ANX7 as a bio-marker in prostate and breast cancer progression. Dis Markers 2001;17:115-20.
109. Reeves JR, Dulude H, Panchal C, Daigneault L, Ramnani DM. Prognostic value of prostate secretory protein of 94 amino acids and its binding protein after radical prostatectomy. Clin Cancer Res 2006;12:6018-22.
110. Mitra AV, Bancroft EK, Barbachano Y*, et al.* Targeted prostate cancer screening in men with mutations in BRCA1 and BRCA2 detects aggressive prostate cancer: preliminary analysis of the results of the IMPACT study. BJU Int;107:28-39.
111. Perttu MC, Martikainen PM, Huhtala HS*, et al.* Altered levels of Smad2 and Smad4 are associated with human prostate carcinogenesis. Prostate Cancer Prostatic Dis 2006;9:185-9.
112. Johnson JL, Ellis BA, Noack D, Seabra MC, Catz SD. The Rab27a-binding protein, JFC1, regulates androgen-dependent secretion of prostate-specific antigen and prostatic-specific acid phosphatase. Biochem J 2005;391:699-710.
113. D'Antonio KB, Schultz L, Albadine R*, et al.* Decreased expression of Cyr61 is associated with prostate cancer recurrence after surgical treatment. Clin Cancer Res;16:5908-13.
114. Chen Q, Watson JT, Marengo SR*, et al.* Gene expression in the LNCaP human prostate cancer progression model: progression associated expression in vitro corresponds to expression changes associated with prostate cancer progression in vivo. Cancer Lett 2006;244:274-88.
115. Johansson B, Pourian MR, Chuan YC*, et al.* Proteomic comparison of prostate cancer cell lines LNCaP-FGC and LNCaP-r reveals heatshock protein 60 as a marker for prostate malignancy. Prostate 2006;66:1235-44.
116. Tsavaler L, Shapero MH, Morkowski S, Laus R. Trp-p8, a novel prostate-specific gene, is up-regulated in prostate cancer and other malignancies and shares high homology with transient receptor potential calcium channel proteins. Cancer Res 2001;61:3760-9.
117. Thomasson M, Wang B, Hammarsten P*, et al.* LRIG1 and the liar paradox in prostate cancer: a study of the expression and clinical significance of LRIG1 in prostate cancer. Int J Cancer;128:2843-52.
118. Su F, Pascal LE, Xiao W, Wang Z. Tumor suppressor U19/EAF2 regulates thrombospondin-1 expression via p53. Oncogene;29:421-31.
119. Liu JW, Nagpal JK, Sun W*, et al.* ssDNA-binding protein 2 is frequently hypermethylated and suppresses cell growth in human prostate cancer. Clin Cancer Res 2008;14:3754-60.
120. Lu X, Kang Y. Epidermal growth factor signalling and bone metastasis. Br J Cancer;102:457-61.
121. Phe V, Cussenot O, Roupret M. Interest of methylated genes as biomarkers in urothelial cell carcinomas of the urinary tract. BJU Int 2009;104:896-901.

122. Takahashi S, Urano T, Tsuchiya F*, et al.* EBAG9/RCAS1 expression and its prognostic significance in prostatic cancer. Int J Cancer 2003;106:310-5.
123. Chakravarti A, DeSilvio M, Zhang M*, et al.* Prognostic value of p16 in locally advanced prostate cancer: a study based on Radiation Therapy Oncology Group Protocol 9202. J Clin Oncol 2007;25:3082-9.
124. Berney DM, Gopalan A, Kudahetti S*, et al.* Ki-67 and outcome in clinically localised prostate cancer: analysis of conservatively treated prostate cancer patients from the Trans-Atlantic Prostate Group study. Br J Cancer 2009;100:888-93.
125. Fan C, Quan R, Feng X*, et al.* ATM activation is accompanied with earlier stages of prostate tumorigenesis. Biochim Biophys Acta 2006;1763:1090-7.
126. Yang J, Wu HF, Qian LX*, et al.* Increased expressions of vascular endothelial growth factor (VEGF), VEGF-C and VEGF receptor-3 in prostate cancer tissue are associated with tumor progression. Asian J Androl 2006;8:169-75.
127. Staibano S, Mascolo M, Di Benedetto M*, et al.* BAG3 protein delocalisation in prostate carcinoma. Tumour Biol;31:461-9.
128. Jain A, McKnight DA, Fisher LW*, et al.* Small integrin-binding proteins as serum markers for prostate cancer detection. Clin Cancer Res 2009;15:5199-207.
129. Benko G, Spajic B, Demirovic A, Stimac G, Kru Sbreve Lin B, Tomas D. Prognostic value of connexin43 expression in patients with clinically localized prostate cancer. Prostate Cancer Prostatic Dis;14:90-5.
130. Sidiropoulos M, Chang A, Jung K, Diamandis EP. Expression and regulation of prostate androgen regulated transcript-1 (PART-1) and identification of differential expression in prostatic cancer. Br J Cancer 2001;85:393-7.
131. Yuen HF, Chan YP, Cheung WL, Wong YC, Wang X, Chan KW. The prognostic significance of BMP-6 signaling in prostate cancer. Mod Pathol 2008;21:1436-43.
132. Amamoto R, Yagi M, Song Y*, et al.* Mitochondrial p32/C1QBP is highly expressed in prostate cancer and is associated with shorter prostate-specific antigen relapse time after radical prostatectomy. Cancer Sci;102:639-47.
133. Hu Y, Wang M, Veverka K, Garcia FU, Stearns ME. The ABCA5 protein: a urine diagnostic marker for prostatic intraepithelial neoplasia. Clin Cancer Res 2007;13:929-38.
134. Nagao K, Yamamoto Y, Hara T*, et al.* Ki67 and BUBR1 may discriminate clinically insignificant prostate cancer in the PSA range <4 ng/ml. Jpn J Clin Oncol;41:555-64.
135. Li D. Peaking of MMP-26 and TIMP-4 marks invasive transition in prostate cancer. Cell Res 2006;16:741.
136. Lee S, Desai KK, Iczkowski KA*, et al.* Coordinated peak expression of MMP-26 and TIMP-4 in preinvasive human prostate tumor. Cell Res 2006;16:750-8.
137. Yaskiv O, Zhang X, Simmerman K*, et al.* The utility of ERG/P63 double immunohistochemical staining in the diagnosis of limited cancer in prostate needle biopsies. Am J Surg Pathol;35:1062-8.
138. He H, Magi-Galluzzi C, Li J*, et al.* The diagnostic utility of novel immunohistochemical marker ERG in the workup of prostate biopsies with "atypical glands suspicious for cancer". Am J Surg Pathol;35:608-14.
139. Miettinen M, Wang ZF, Paetau A*, et al.* ERG transcription factor as an immunohistochemical marker for vascular endothelial tumors and prostatic carcinoma. Am J Surg Pathol;35:432-41.
140. Malik G, Ward MD, Gupta SK*, et al.* Serum levels of an isoform of apolipoprotein A-II as a potential marker for prostate cancer. Clin Cancer Res 2005;11:1073-85.
141. Halvorsen OJ, Rostad K, Oyan AM*, et al.* Increased expression of SIM2-s protein is a novel marker of aggressive prostate cancer. Clin Cancer Res 2007;13:892-7.
142. Laxman B, Morris DS, Yu J*, et al.* A first-generation multiplex biomarker analysis of urine for the early detection of prostate cancer. Cancer Res 2008;68:645-9.
143. Mirtti T, Alanen K, Kallajoki M, Rinne A, Soderstrom KO. Expression of cystatins, high molecular weight cytokeratin, and proliferation markers in prostatic adenocarcinoma and hyperplasia. Prostate 2003;54:290-8.

144. Richardsen E, Uglehus RD, Due J, Busch C, Busund LT. The prognostic impact of M-CSF, CSF-1 receptor, CD68 and CD3 in prostatic carcinoma. Histopathology 2008;53:30-8.
145. Hale LP, Price DT, Sanchez LM, Demark-Wahnefried W, Madden JF. Zinc alpha-2-glycoprotein is expressed by malignant prostatic epithelium and may serve as a potential serum marker for prostate cancer. Clin Cancer Res 2001;7:846-53.
146. Sen M, McHugh K, Hutzley J*, et al.* Orthotopic expression of human 15-lipoxygenase (LO)-1 in the dorsolateral prostate of normal wild-type C57BL/6 mouse causes PIN-like lesions. Prostaglandins Other Lipid Mediat 2006;81:1-13.
147. Zi X, Guo Y, Simoneau AR*, et al.* Expression of Frzb/secreted Frizzled-related protein 3, a secreted Wnt antagonist, in human androgen-independent prostate cancer PC-3 cells suppresses tumor growth and cellular invasiveness. Cancer Res 2005;65:9762-70.
148. Szasz AM, Nyirady P, Majoros A*, et al.* beta-catenin expression and claudin expression pattern as prognostic factors of prostatic cancer progression. BJU Int;105:716-22.
149. Murphy C, McGurk M, Pettigrew J*, et al.* Nonapical and cytoplasmic expression of interleukin-8, CXCR1, and CXCR2 correlates with cell proliferation and microvessel density in prostate cancer. Clin Cancer Res 2005;11:4117-27.
150. Jamaspishvili T, Kral M, Khomeriki I, Student V, Kolar Z, Bouchal J. Urine markers in monitoring for prostate cancer. Prostate Cancer Prostatic Dis;13:12-9.
151. Miyake H, Hara I, Eto H. Serum level of cathepsin B and its density in men with prostate cancer as novel markers of disease progression. Anticancer Res 2004;24:2573-7.
152. Kamiya N, Suzuki H, Endo T*, et al.* Significance of serum osteoprotegerin and receptor activator of nuclear factor kappaB ligand in Japanese prostate cancer patients with bone metastasis. Int J Clin Oncol;16:366-72.
153. Morgan R, Boxall A, Bhatt A*, et al.* Engrailed-2 (EN2): a tumor specific urinary biomarker for the early diagnosis of prostate cancer. Clin Cancer Res;17:1090-8.
154. Li T, Su Y, Mei Y*, et al.* ALDH1A1 is a marker for malignant prostate stem cells and predictor of prostate cancer patients' outcome. Lab Invest;90:234-44.
155. Barboro P, Repaci E, Rubagotti A*, et al.* Heterogeneous nuclear ribonucleoprotein K: altered pattern of expression associated with diagnosis and prognosis of prostate cancer. Br J Cancer 2009;100:1608-16.
156. Fixemer T, Wissenbach U, Flockerzi V, Bonkhoff H. Expression of the Ca2+-selective cation channel TRPV6 in human prostate cancer: a novel prognostic marker for tumor progression. Oncogene 2003;22:7858-61.
157. Bartholow TL, Chandran UR, Becich MJ, Parwani AV. Immunohistochemical profiles of claudin-3 in primary and metastatic prostatic adenocarcinoma. Diagn Pathol;6:12.
158. Knudsen BS, Lucas JM, Fazli L*, et al.* Regulation of hepatocyte activator inhibitor-1 expression by androgen and oncogenic transformation in the prostate. Am J Pathol 2005;167:255-66.
159. Kieffer N, Schmitz M, Plancon S*, et al.* ILK as a potential marker gene to ascertain specific adenocarcinoma cell mRNA isolation from frozen prostate biopsy tissue sections. Int J Oncol 2005;26:1549-58.
160. Laitinen S, Martikainen PM, Tolonen T, Isola J, Tammela TL, Visakorpi T. EZH2, Ki-67 and MCM7 are prognostic markers in prostatectomy treated patients. Int J Cancer 2008;122:595-602.
161. Gross M, Top I, Laux I*, et al.* Beta-2-microglobulin is an androgen-regulated secreted protein elevated in serum of patients with advanced prostate cancer. Clin Cancer Res 2007;13:1979-86.
162. Pressinotti NC, Klocker H, Schafer G*, et al.* Differential expression of apoptotic genes PDIA3 and MAP3K5 distinguishes between low- and high-risk prostate cancer. Mol Cancer 2009;8:130.
163. Saeki N, Gu J, Yoshida T, Wu X. Prostate stem cell antigen: a Jekyll and Hyde molecule? Clin Cancer Res;16:3533-8.
164. Avgeris M, Mavridis K, Scorilas A. Kallikrein-related peptidase genes as promising biomarkers for prognosis and monitoring of human malignancies. Biol Chem;391:505-11.
165. Paliouras M, Borgono C, Diamandis EP. Human tissue kallikreins: the cancer biomarker family. Cancer Lett 2007;249:61-79.

166. Lam JS, Seligson DB, Yu H*, et al.* Flap endonuclease 1 is overexpressed in prostate cancer and is associated with a high Gleason score. BJU Int 2006;98:445-51.
167. Wang X, Raulji P, Mohapatra SS*, et al.* Natriuretic peptide receptor a as a novel target for prostate cancer. Mol Cancer;10:56.
168. Zhang F, Lee J, Lu S, Pettaway CA, Dong Z. Blockade of transforming growth factor-beta signaling suppresses progression of androgen-independent human prostate cancer in nude mice. Clin Cancer Res 2005;11:4512-20.
169. Popovic A, Demirovic A, Spajic B, Stimac G, Kruslin B, Tomas D. Expression and prognostic role of syndecan-2 in prostate cancer. Prostate Cancer Prostatic Dis;13:78-82.
170. Cho-Vega JH, Tsavachidis S, Do KA, Nakagawa J, Medeiros LJ, McDonnell TJ. Dicarbonyl/L-xylulose reductase: a potential biomarker identified by laser-capture microdissection-micro serial analysis of gene expression of human prostate adenocarcinoma. Cancer Epidemiol Biomarkers Prev 2007;16:2615-22.
171. Ruan W, Sassoon A, An F, Simko JP, Liu B. Identification of clinically significant tumor antigens by selecting phage antibody library on tumor cells in situ using laser capture microdissection. Mol Cell Proteomics 2006;5:2364-73.
172. Cheng HL, Huang HJ, Ou BY*, et al.* Urinary CD14 as a potential biomarker for benign prostatic hyperplasia - discovery by combining MALDI-TOF-based biostatistics and ESI-MS/MS-based stable-isotope labeling. Proteomics Clin Appl;5:121-32.
173. Toton E, Ignatowicz E, Skrzeczkowska K, Rybczynska M. Protein kinase Cepsilon as a cancer marker and target for anticancer therapy. Pharmacol Rep;63:19-29.
174. Tobias-Machado M, Fonseca F, Fantinato AP*, et al.* Cytokeratin 19 expression by reverse transcriptase-polymerase chain reaction in the peripheral blood of prostate cancer patients. Tumori 2005;91:248-52.
175. Schmitz M, Grignard G, Margue C*, et al.* Complete loss of PTEN expression as a possible early prognostic marker for prostate cancer metastasis. Int J Cancer 2007;120:1284-92.
176. Zhong WD, Liang YX, Lin SX*, et al.* Expression of CD147 is associated with prostate cancer progression. Int J Cancer;130:300-8.
177. Heeboll S, Borre M, Ottosen PD*, et al.* SMARCC1 expression is upregulated in prostate cancer and positively correlated with tumour recurrence and dedifferentiation. Histol Histopathol 2008;23:1069-76.
178. Singh SK, Grifson JJ, Mavuduru RS, Agarwal MM, Mandal AK, Jha V. Serum leptin: a marker of prostate cancer irrespective of obesity. Cancer Biomark;7:11-5.
179. Kanoh Y, Ohtani N, Mashiko T*, et al.* Levels of alpha 2 macroglobulin can predict bone metastases in prostate cancer. Anticancer Res 2001;21:551-6.
180. Ang J, Lijovic M, Ashman LK, Kan K, Frauman AG. CD151 protein expression predicts the clinical outcome of low-grade primary prostate cancer better than histologic grading: a new prognostic indicator? Cancer Epidemiol Biomarkers Prev 2004;13:1717-21.
181. Jia X, Li X, Xu Y*, et al.* SOX2 promotes tumorigenesis and increases the anti-apoptotic property of human prostate cancer cell. J Mol Cell Biol;3:230-8.
182. Mortezavi A, Hermanns T, Seifert HH*, et al.* KPNA2 expression is an independent adverse predictor of biochemical recurrence after radical prostatectomy. Clin Cancer Res;17:1111-21.
183. Stewart J, Fleshner N, Cole H, Sweet J. Comparison of annexin II, p63 and alpha-methylacyl-CoA racemase immunoreactivity in prostatic tissue: a tissue microarray study. J Clin Pathol 2007;60:773-80.
184. Kai L, Wang J, Ivanovic M*, et al.* Targeting prostate cancer angiogenesis through metastasis-associated protein 1 (MTA1). Prostate;71:268-80.
185. Barwick BG, Abramovitz M, Kodani M*, et al.* Prostate cancer genes associated with TMPRSS2-ERG gene fusion and prognostic of biochemical recurrence in multiple cohorts. Br J Cancer;102:570-6.
186. Andren O, Fall K, Andersson SO*, et al.* MUC-1 gene is associated with prostate cancer death: a 20-year follow-up of a population-based study in Sweden. Br J Cancer 2007;97:730-4.
187. Marin LV, Ferariu D, Mihailovici MS. [Immunohistochemic staining for CK5/6 and P63 significance in prostate premalignant lesions and adenocarcinoma]. Rev Med Chir Soc Med Nat Iasi;114:818-22.

188. Knight JF, Shepherd CJ, Rizzo S*, et al.* TEAD1 and c-Cbl are novel prostate basal cell markers that correlate with poor clinical outcome in prostate cancer. Br J Cancer 2008;99:1849-58.
189. Kremer CL, Klein RR, Mendelson J*, et al.* Expression of mTOR signaling pathway markers in prostate cancer progression. Prostate 2006;66:1203-12.
190. Mirtti T, Laine VJ, Hiekkanen H*, et al.* Group IIA phospholipase A as a prognostic marker in prostate cancer: relevance to clinicopathological variables and disease-specific mortality. Apmis 2009;117:151-61.
191. Hernandez-Plata E, Ortiz CS, Marquina-Castillo B*, et al.* Overexpression of NaV 1.6 channels is associated with the invasion capacity of human cervical cancer. Int J Cancer;130:2013-23.
192. Kuruma H, Kamata Y, Takahashi H*, et al.* Staphylococcal nuclease domain-containing protein 1 as a potential tissue marker for prostate cancer. Am J Pathol 2009;174:2044-50.
193. Dunn TA, Chen S, Faith DA*, et al.* A novel role of myosin VI in human prostate cancer. Am J Pathol 2006;169:1843-54.
194. Koochekpour S, Hu S, Vellasco-Gonzalez C*, et al.* Serum prosaposin levels are increased in patients with advanced prostate cancer. Prostate;72:253-69.
195. Sotomayor P, Godoy A, Smith GJ, Huss WJ. Oct4A is expressed by a subpopulation of prostate neuroendocrine cells. Prostate 2009;69:401-10.
196. Sun D, Lee YS, Malhotra A*, et al.* miR-99 family of MicroRNAs suppresses the expression of prostate-specific antigen and prostate cancer cell proliferation. Cancer Res;71:1313-24.
197. Leite KR, Tomiyama A, Reis ST*, et al.* MicroRNA-100 expression is independently related to biochemical recurrence of prostate cancer. J Urol;185:1118-22.
198. Pang B, Zhang H, Wang J*, et al.* Ubiquitous mitochondrial creatine kinase is overexpressed in the conditioned medium and the extract of LNCaP lineaged androgen independent cell lines and facilitates prostate cancer progression. Prostate 2009;69:1176-87.
199. Tang WM, Gou X, Liu QX. [Expression of macrophage migration inhibition factor (MIF) in serum of patients with prostate cancer and its clinical significance]. Xi Bao Yu Fen Zi Mian Yi Xue Za Zhi;27:97-8.
200. Kristiansen G, Fritzsche FR, Wassermann K*, et al.* GOLPH2 protein expression as a novel tissue biomarker for prostate cancer: implications for tissue-based diagnostics. Br J Cancer 2008;99:939-48.
201. Krajewska M, Turner BC, Shabaik A, Krajewski S, Reed JC. Expression of BAG-1 protein correlates with aggressive behavior of prostate cancers. Prostate 2006;66:801-10.
202. Kristiansen G, Pilarsky C, Wissmann C*, et al.* Expression profiling of microdissected matched prostate cancer samples reveals CD166/MEMD and CD24 as new prognostic markers for patient survival. J Pathol 2005;205:359-76.
203. Coutinho-Camillo CM, Salaorni S, Sarkis AS, Nagai MA. Differentially expressed genes in the prostate cancer cell line LNCaP after exposure to androgen and anti-androgen. Cancer Genet Cytogenet 2006;166:130-8.
204. Byrne JC, Downes MR, O'Donoghue N, *et al.* 2D-DIGE as a strategy to identify serum markers for the progression of prostate cancer. J Proteome Res 2009;8:942-57.
205. Sardana G, Dowell B, Diamandis EP. Emerging biomarkers for the diagnosis and prognosis of prostate cancer. Clin Chem 2008;54:1951-60.
206. Goo YA, Goodlett DR. Advances in proteomic prostate cancer biomarker discovery. J Proteomics;73:1839-50.
207. Heneghan HM, Miller N, Kerin MJ. MiRNAs as biomarkers and therapeutic targets in cancer. Curr Opin Pharmacol;10:543-50.
208. Schmidt U, Fuessel S, Koch R*, et al.* Quantitative multi-gene expression profiling of primary prostate cancer. Prostate 2006;66:1521-34.
209. Alam SM, Rajendran M, Ouyang S, Veeramani S, Zhang L, Lin MF. A novel role of Shc adaptor proteins in steroid hormone-regulated cancers. Endocr Relat Cancer 2009;16:1-16.
210. Pedersen MO, Larsen A, Stoltenberg M, Penkowa M. The role of metallothionein in oncogenesis and cancer prognosis. Prog Histochem Cytochem 2009;44:29-64.

211. Glinsky GV, Glinskii AB, Stephenson AJ, Hoffman RM, Gerald WL. Gene expression profiling predicts clinical outcome of prostate cancer. J Clin Invest 2004;113:913-23.
212. Tesan T, Gustavsson H, Welen K, Damber JE. Differential expression of angiopoietin-2 and vascular endothelial growth factor in androgen-independent prostate cancer models. BJU Int 2008;102:1034-9.
213. Laramas M, Pasquier D, Filhol O, Ringeisen F, Descotes JL, Cochet C. Nuclear localization of protein kinase CK2 catalytic subunit (CK2alpha) is associated with poor prognostic factors in human prostate cancer. Eur J Cancer 2007;43:928-34.
214. Varambally S, Laxman B, Mehra R, *et al.* Golgi protein GOLM1 is a tissue and urine biomarker of prostate cancer. Neoplasia 2008;10:1285-94.
215. Lucas N, Day ML. The role of the disintegrin metalloproteinase ADAM15 in prostate cancer progression. J Cell Biochem 2009;106:967-74.
216. Yu J, Cao Q, Yu J, *et al.* The neuronal repellent SLIT2 is a target for repression by EZH2 in prostate cancer. Oncogene;29:5370-80.
217. Schlomm T, Nakel E, Lubke A, *et al.* Marked gene transcript level alterations occur early during radical prostatectomy. Eur Urol 2008;53:333-44.
218. Fritzsche FR, Jung M, Tolle A, *et al.* ADAM9 expression is a significant and independent prognostic marker of PSA relapse in prostate cancer. Eur Urol 2008;54:1097-106.
219. Darash-Yahana M, Gillespie JW, Hewitt SM, *et al.* The chemokine CXCL16 and its receptor, CXCR6, as markers and promoters of inflammation-associated cancers. PLoS One 2009;4:e6695.
220. Gu Z, Rubin MA, Yang Y, *et al.* Reg IV: a promising marker of hormone refractory metastatic prostate cancer. Clin Cancer Res 2005;11:2237-43.
221. Sun C, Song C, Ma Z, *et al.* Periostin identified as a potential biomarker of prostate cancer by iTRAQ-proteomics analysis of prostate biopsy. Proteome Sci;9:22.
222. Grimaldi F, Valotto C, Barbina G, *et al.* The possible role of chromogranin A as a prognostic factor in organ-confined prostate cancer. Int J Biol Markers 2006;21:229-34.
223. Kobel M, Xu H, Bourne PA, *et al.* IGF2BP3 (IMP3) expression is a marker of unfavorable prognosis in ovarian carcinoma of clear cell subtype. Mod Pathol 2009;22:469-75.
224. Xie D, Gore C, Liu J, *et al.* Role of DAB2IP in modulating epithelial-to-mesenchymal transition and prostate cancer metastasis. Proc Natl Acad Sci U S A;107:2485-90.
225. Ohkia A, Hu Y, Wang M, Garcia FU, Stearns ME. Evidence for prostate cancer-associated diagnostic marker-1: immunohistochemistry and in situ hybridization studies. Clin Cancer Res 2004;10:2452-8.
226. Liu H, Ding J, Chen F, *et al.* Increased expression of elongation factor-1alpha is significantly correlated with poor prognosis of human prostate cancer. Scand J Urol Nephrol;44:277-83.
227. Dai B, Kim O, Xie Y, *et al.* Tyrosine kinase Etk/BMX is up-regulated in human prostate cancer and its overexpression induces prostate intraepithelial neoplasia in mouse. Cancer Res 2006;66:8058-64.
228. Graham TR, Zhau HE, Odero-Marah VA, *et al.* Insulin-like growth factor-I-dependent up-regulation of ZEB1 drives epithelial-to-mesenchymal transition in human prostate cancer cells. Cancer Res 2008;68:2479-88.
229. Steiner I, Jung K, Schatz P, *et al.* Gene promoter methylation and its potential relevance in early prostate cancer diagnosis. Pathobiology;77:260-6.
230. Bialkowska-Hobrzanska H, Driman DK, Fletcher R, Harry V, Razvi H. Expression of human telomerase reverse transcriptase, Survivin, DD3 and PCGEM1 messenger RNA in archival prostate carcinoma tissue. Can J Urol 2006;13:2967-74.
231. Liu W, Gong J, Hu J, *et al.* Quantitative assessment of AKAP12 promoter methylation in human prostate cancer using methylation-sensitive high-resolution melting: correlation with Gleason score. Urology;77:1006 e1-7.
232. Fritzsche FR, Jung M, Xu C, *et al.* ADAM8 expression in prostate cancer is associated with parameters of unfavorable prognosis. Virchows Arch 2006;449:628-36.
233. Wang J, Weng J, Cai Y, Penland R, Liu M, Ittmann M. The prostate-specific G-protein coupled receptors PSGR and PSGR2 are prostate cancer biomarkers that are complementary to alpha-methylacyl-CoA racemase. Prostate 2006;66:847-57.

234. Rosenzweig CN, Zhang Z, Sun X*, et al.* Predicting prostate cancer biochemical recurrence using a panel of serum proteomic biomarkers. J Urol 2009;181:1407-14.
235. Gojis O, Rudraraju B, Alifrangis C, Krell J, Libalova P, Palmieri C. The role of steroid receptor coactivator-3 (SRC-3) in human malignant disease. Eur J Surg Oncol;36:224-9.
236. Trudel D, Fradet Y, Meyer F, Harel F, Tetu B. Membrane-type-1 matrix metalloproteinase, matrix metalloproteinase 2, and tissue inhibitor of matrix proteinase 2 in prostate cancer: identification of patients with poor prognosis by immunohistochemistry. Hum Pathol 2008;39:731-9.
237. Gonzalez-Gronow M, Hershfield MS, Arredondo-Vega FX, Pizzo SV. Cell surface adenosine deaminase binds and stimulates plasminogen activation on 1-LN human prostate cancer cells. J Biol Chem 2004;279:20993-8.
238. Caruso DJ, Carmack AJ, Lokeshwar VB, Duncan RC, Soloway MS, Lokeshwar BL. Osteopontin and interleukin-8 expression is independently associated with prostate cancer recurrence. Clin Cancer Res 2008;14:4111-8.
239. Liu H, Liu W, Wu Y*, et al.* Loss of epigenetic control of synuclein-gamma gene as a molecular indicator of metastasis in a wide range of human cancers. Cancer Res 2005;65:7635-43.
240. Lilleby W, Paus E, Skovlund E, Fossa SD. Prognostic value of neuroendocrine serum markers and PSA in irradiated patients with pN0 localized prostate cancer. Prostate 2001;46:126-33.
241. Rabbani SA, Gladu J. Urokinase receptor antibody can reduce tumor volume and detect the presence of occult tumor metastases in vivo. Cancer Res 2002;62:2390-7.
242. Jungwirth N, Haeberle L, Schrott KM, Wullich B, Krause FS. Serotonin used as prognostic marker of urological tumors. World J Urol 2008;26:499-504.
243. Cook RJ, Coleman R, Brown J, *et al.* Markers of bone metabolism and survival in men with hormone-refractory metastatic prostate cancer. Clin Cancer Res. 2006;12:3361-7.
244. Simpson MA, Reiland J, Burger SR*, et al.* Hyaluronan synthase elevation in metastatic prostate carcinoma cells correlates with hyaluronan surface retention, a prerequisite for rapid adhesion to bone marrow endothelial cells. J Biol Chem 2001;276:17949-57.
